# Quo vadis? Microbial profiling revealed strong effects of cleanroom maintenance and routes of contamination in indoor environments

**DOI:** 10.1038/srep09156

**Published:** 2015-03-17

**Authors:** Christine Moissl-Eichinger, Anna K. Auerbach, Alexander J. Probst, Alexander Mahnert, Lauren Tom, Yvette Piceno, Gary L. Andersen, Kasthuri Venkateswaran, Petra Rettberg, Simon Barczyk, Rüdiger Pukall, Gabriele Berg

**Affiliations:** 1 Institute for Microbiology and Archaea Center, University of Regensburg, Universitaetsstrasse 31, 93053 Regensburg, Germany; 2 Medical University Graz, Department of Internal Medicine, Auenbruggerplatz 15, 8036 Graz, Austria; 3 BioTechMed Graz, Krenngasse 37, 8010 Graz, Austria; 4 Institute of Environmental Biotechnology, Graz University of Technology, Petersgasse 12, 8010 Graz, Austria; 5 Lawrence Berkeley National Laboratory, Earth Sciences Division, 1 Cyclotron Rd., Berkeley, CA 94720, USA; 6 Jet Propulsion Laboratory, 4800 Oak Grove Drive, Pasadena, CA 91109, USA; 7 German Aerospace Center, Institute of Aerospace Medicine and Radiation Biology, Linder Höhe, 51147 Köln, Germany; 8 Leibniz Institute DSMZ - Deutsche Sammlung von Mikroorganismen und Zellkulturen GmbH, Inhoffenstraβe 7 B, 38124 Braunschweig, Germany

## Abstract

Space agencies maintain highly controlled cleanrooms to ensure the demands of planetary protection. To study potential effects of microbiome control, we analyzed microbial communities in two particulate-controlled cleanrooms (ISO 5 and ISO 8) and two vicinal uncontrolled areas (office, changing room) by cultivation and 16S rRNA gene amplicon analysis (cloning, pyrotagsequencing, and PhyloChip G3 analysis). Maintenance procedures affected the microbiome on total abundance and microbial community structure concerning richness, diversity and relative abundance of certain taxa. Cleanroom areas were found to be mainly predominated by potentially human-associated bacteria; archaeal signatures were detected in every area. Results indicate that microorganisms were mainly spread from the changing room (68%) into the cleanrooms, potentially carried along with human activity. The numbers of colony forming units were reduced by up to ~400 fold from the uncontrolled areas towards the ISO 5 cleanroom, accompanied with a reduction of the living portion of microorganisms from 45% (changing area) to 1% of total 16S rRNA gene signatures as revealed via propidium monoazide treatment of the samples. Our results demonstrate the strong effects of cleanroom maintenance on microbial communities in indoor environments and can be used to improve the design and operation of biologically controlled cleanrooms.

The vast majority of microorganisms is known to play essential roles in natural ecosystem or eukaryote functioning^1^. However, the indoor microbiome is only at the beginning of being explored and could have severe impact on human health, well-being or living comfort^2^,^3^. Next generation sequencing and OMICS-technologies have tremendously contributed to the census of microbial diversity and enabled global projects analyzing terrestrial, marine, and human microbiomes^1^,^4^,^5^. These techniques opened up also new possibilities to study indoor microbiomes, which are an important component of everyday human health^6^,^7^,^8^. In general, uncontrolled indoor microbial communities are characterized by a high prokaryotic diversity and are comprised of diverse bacterial and archaeal phyla^7^,^8^,^9^,^10^,^11^. The microorganisms originate mainly from the human skin or from outside air and soil, and have even been known to include extremophiles^12^. In addition, the plant microbiome was suggested as important source for indoor microbiomes^13^. Although numerous developments and improvements have been reported during the last decade, the proper monitoring and control of microbial contamination remains one of the biggest challenges in pharmaceutical quality control, food industry, agriculture or maintenance of health-care associated buildings, including intensive care units^14^,^15^,^16^.

Another important research area dealing with indoor microbiomes is planetary protection, which aims to prevent biological contamination of both the target celestial body and the Earth^17^. Space missions that are intended to land on extraterrestrial bodies of elevated interest (in chemical and biological evolution and significant contamination risk) are subject to COSPAR (Committee on Space Research) regulations, which allow only extremely low levels of biological contamination. However, all space agencies that involve in life-detection and sample return missions should consider to catalogue microbial inventory associated with spacecraft using state-of-the art molecular techniques that enable not to compromise the science of such missions. At present, all space agencies enumerate heat-shock resistant microorganisms as a proxy for the general biological cleanliness of spacecraft surfaces that are bound to Mars (COSPAR planetary protection policy; ECSS (European Cooperation for Space Standardization)-Q-ST-70-55C^18^).

In order to avoid contaminants as much as possible, spacecraft are constructed in highly controlled cleanrooms that follow strict ISO and ECSS classifications (ISO 14644; ECSS-Q-ST-70-58C, http://esmat.esa.int/ecss-q-st-70-58c.pdf and https://www.iso.org/obp/ui/#iso:std:iso:14644:-1:ed-1:v1:en). Cleanrooms for spacecraft assembly were the first indoor environments, which were extensively studied with respect to their microbiome^10^,^19^,^20^,^21^,^22^,^23^. As expected, the detected microbial diversity and abundance strongly correlated with the applied sampling and detection methods, and during the last years a vast variety of bacterial contaminants was revealed^18^,^24^. The aforementioned studies gave rise that cleanroom microbiomes are mainly composed of human-associated microbes and hardy survival specialists and spore-forming bacteria as they can tolerate harsh cleanroom conditions^24^. However, cultivation assays that even included media for specialized microbes like anaerobic broths need to be complemented with molecular assays due to the vast majority of uncultivated microorganisms in general^24^. These molecular methods enabled the detection of archaea as a low but constant contamination in cleanrooms, and their presence was linked to human activity; the human body and in particular the skin was shown to function as a carrier of a variety of archaea and is therefore responsible for the transfer of these organisms into cleanrooms^25^. In contrast to general office or other indoor areas, controlled indoor environments, such as cleanrooms, represent an extraordinary, extreme habitat for microorganisms: the exchange with the outer environment is limited as much as possible, the air is constantly filtered, and particles are vastly reduced and frequent cleaning and/or disinfection of surfaces is performed. To date none of the previous research activities have focused on the real effect of cleanroom maintenance procedures on the diversity and abundance of microorganisms and compared the microbiome to typical indoor environments, such as an office facility.

To overcome this gap of knowledge, we have analyzed a cleanroom complex operated by Airbus Defence and Space GmbH in Friedrichshafen, Germany. The controlled environments at this complex are not monitored for biological contamination but provide an excellent research object in order to determine the baseline contamination level and possible contamination routes. The Airbus Defence and Space complex harbors uncontrolled rooms: an office (check-out room, CO), a changing room (UR) and two controlled cleanrooms of different ISO certification in very close vicinity (CR8, CR5; Fig. 1). We used four different methods, which were cultivation, classical 16S rRNA gene cloning, 454 pyrotagsequencing and PhyloChip G3™ technology, in order to analyze the microbial diversity and abundance of these four separated modules at a cleanroom facility. In addition, we performed network analyses to visualize the microbial contamination tracks within the entire facility.

## Results

### Abundance of microorganisms decreased from uncontrolled to controlled areas

In general, the distribution of cultivable microbes on facility floors was very heterogeneous. Wipe samples taken in one room revealed highly variable colony counts of up to three orders of magnitude. However, technical replicates from one wipe were comparably low in variation with respect to obtained colony counts (representatively, original data for oligotrophs and alkaliphiles are given in Table S1). As shown in Table 1, the changing room (UR) revealed the highest colony counts of cultivable oligotrophs (17.2 × 10^3^ colony forming units (CFU) per m^2^), alkaliphiles (1.9 × 10^3^ CFU per m^2^) and anaerobes (44.4 × 10^3^ CFU per m^2^), whereas the lowest numbers of cultivable microorganisms were detected in the CR5 cleanroom (0.4 × 10^3^, 0, 0.1 × 10^3^ CFU per m^2^, respectively). This corresponds to an at least 40-fold reduction of CFUs towards CR5. Bioburden determination according to ESA standard protocols revealed the highest number of CFU in CO samples (heat-shock resistant microbes: 0.2 × 10^3^ CFU per m^2^) and UR (without heat-shock).

These cultivation-based observations were confirmed by qPCR analyses of wipe samples, which revealed the highest contamination in the check-out as well as the changing room (both approx. 3 × 10^7^ gene copies per m^2^), corresponding to an estimated microbial contamination of about 7 × 10^6^ cells per m^2^ (in average 4.2 16S rRNA gene copies per bacterial genome^26^). The two cleanrooms revealed an order of magnitude lower gene copy numbers (Table 1). When samples were pre-treated with PMA to mask free DNA (i.e. DNA not enclosed in an intact cell membrane), detected copy numbers per m^2^ were even lower: 5.5 × 10^4^ (CR5), 2.6 × 10^5^ (CR8), 2.0 × 10^6^ (CO) and 1.3 × 10^7^ (UR; Table 1). The changing room (UR) thus revealed the highest portion of intact cells (45% of 16S rRNA gene copies).

### Cultivation approach revealed the omnipresence of *Staphylococcus*, *Bacillus* and *Micrococcus* in all areas and a great diversity overlap of changing room with cleanroom areas

Cultivation on alternative media (for oligotrophic, anaerobic, and alkalitolerant micro-organisms) revealed the presence of facultatively oligotrophic and facultatively anaerobic microorganisms in all rooms. Alkalitolerant microorganisms were not detected in CR8 and in very low abundance in CR5 (Table 1). The relative distribution of identified isolates across the facility rooms is depicted in Fig. 2. A complete list of all isolates is given in Table S2. The most prevalent microbes were staphylococci and *Microbacterium*, whereas *Staphylococcus* representatives were retrieved from each location and *Microbacterium* from CR8 and UR. The overwhelming majority of the isolates obtained from CR5 were identified as representatives of the genus *Staphylococcus* (*S. caprae, S. capitis, S. lugdunensis, S. pettenkoferi*), whereas most of the colonies were observed under nutrient-reduced conditions (oligotrophic; Fig. 2). *Erwinia* and *Cellulomonas* were only retrieved from cleanroom samples (CR8). The changing room shared four genera (*Acinetobacter, Propionibacterium, Rhodococcus, Microbacterium*) with the cleanroom environment. Except the three omnipresent cultivated genera *Staphylococcus*, *Bacillus* and *Micrococcus* no additional overlap was found for check-out room and cleanrooms, (Fig. 2). Overall, most CFU were obtained from phyla *Firmicutes* (98), *Actinobacteria* (49) and *Gammaproteobacteria* (10). Only two colonies of a *Bacteroidetes*-representative were obtained (*Chryseobacterium*; UR only).

### PMA-16S rRNA gene cloning identified *Corynebacterium* and *Staphylococcus* as intact contaminants in cleanroom areas

An overview of all samples processed, no. of clones analyzed and results from grouping into (cloned) operational taxonomic units (cOTUs) and coverage is given in Table S3. In total, 52 sequences (out of 257) were identified to be chimeric and therefore excluded from the subsequent analyses. Chimeric sequences were only detected in samples not treated with PMA. A detailed table of all analyzed recombinant sequences, their abundance and classification is given in Table S4. Fig. 3 displays the microbial diversity detected with respect to their presence in samples from the different facility areas. Sequences of *Corynebacterium, Staphylococcus*, *Propionibacterium*, *Chryseobacterium* and members of the order *Actinomycetales* were detected in each of the locations. The most restricted cleanroom (CR5) exclusively revealed the presence of *Aerococcaceae, Nostocaceae* and *Deinococcus* signatures.

PMA-treatment of samples allowed the detection of signatures from intact cells of *Corynebacterium* (UR, CR5), *Microbacterium, Propionibacterium, Streptococcus, Brevundimonas, Chroococcidiopsis, Ralstonia, Rickettsiella* (UR), *Propionicimonas, Paracoccus*, *Chitinophagaceae*, *Bacillus*, *Myxococcales*, *Tissierella* (CO) and *Staphylococcus* (UR, CR8). With the exception of omnipresent microorganisms (see above), no overlap occurred between sample diversity obtained from UR samples (changing room) and check-out facility (Fig. 3).

### Alpha diversity analysis of pyrotagsequencing suggested an opposed distribution of *Proteobacteria* and *Firmicutes* signatures in controlled and uncontrolled areas

On average, 1863 bacterial 16S rRNA gene sequences were obtained from each sample. Normalized data revealed the highest microbial diversity (see Table 1) in the checkout (6.0 H′) and the lowest in the changing room (4.76 H′). Nine bacterial phyla were detected after setting a threshold of 1% relative sequence abundance, whereas *Actinobacteria*, *Bacteroidetes*, *Cyanobacteria*, and in particular *Firmicutes* and *Proteobacteria* revealed the highest sequence abundance (see Table S5 and Fig. 4). Bacterial 16S rRNA genes belonging to the phylum *Actinobacteria* were most relative abundant in the checkout room and appeared lower in all other samples (10%). *Bacteriodetes* sequences could be detected in all rooms with a constant relative abundance, with *Wautersiella falsenii* signatures predominating in changing room (UR) amplicons. Sequences of the genus *Tessaracoccus* (*Actinobacteria*) were exclusively found in the checkout (CO). A detailed look at the phylum *Proteobacteria* revealed *Rhodocyclaceae* sequences as most abundant in CO samples, sequences affiliated to the genera *Stenotrophomonas* and *Comamonas* as the most abundant in UR, and *Paracoccus yeei* as the most abundant proteobacterial signature in CR8. Within the *Firmicutes*, 16S rRNA gene signatures of *Aerococcaceae* were predominant in CR5. On genus level, *Anaerococcus* sequences dominated in CR5 and *Paenibacillus* sequences in CR8. Signatures of the species *Finegoldia magna* could be detected in the changing and both cleanrooms with the highest abundance in CR5, whereas *Lactobacillales*-sequences (including *Lactobacillus* and *Lactococcus*) were predominant amongst *Firmicutes* signatures in UR and CO. Noteworthy, the relative abundance of *Firmicutes* sequences increased towards the cleaner areas (CR8, CR5; rel. abundance: 17–45%), whereas proteobacterial pyrosequenced operational taxonomic units (pOTUs) decreased (37–23%) compared to CO and UR areas (see Fig. 4 and Table S5). Overall, the largest portion of *Firmicutes* sequences was obtained from cleanroom samples.

In order to find microorganisms that increased or declined in cleanroom samples, read abundances were normalized to 5000 across each sample and then aggregated at genus level. Genera exhibiting at least 10 reads in one sample and showing a decrease or increase of at least 25% in cleanroom samples over non-cleanroom samples (tested individually) were filtered from the entire dataset and are depicted in Fig. 5.

Altogether 44 microbial genera were found to vary greatly between the two room categories, 17 of them decreased in non-cleanroom samples. These 17 included many Gram (−) bacteria like *Proteobacteria*-related taxa but also *Actinobacteria*. Most of the microbial taxa enriched in cleanroom samples were designated Gram (+), like *Firmicutes* (clostridia, *Paenibacillus*) and again *Actinobacteria*.

### PhyloChip G3™ DNA microarray revealed variations in microbial richness and a great reduction of *Staphylococcus* and other genera in cleanroom areas when considering signatures from intact and non-intact cells

Presence/absence calling of reference-based operational taxonomic units (rOTUs) produced values ranging from 2 to 1007 different microbial taxa with 2059 different rOTUs in total. All areas revealed the signatures of *Streptococcus*, *Microbacterium*, *Corynebacterium* and *Staphylococcus* with up to 361 detected rOTUs belonging to *Streptococcus* (non-PMA treated samples; Fig. 6). Considering the microbial diversity that was unique for each facility area, PhyloChip analyses revealed different compositions compared to pyrotagsequencing data with the exception of *Simplicispira* and *Helcococcus* sequences, which were found by both methods to be solely present in CO and CR, respectively. A complete list of all detected phylotypes (PhyloChip) is given in Table S6.

In order to detect the intact (and thus probably living portion of microbial contaminants), PhyloChip was combined with PMA-treatment prior to DNA extraction of each sample^29^. Non-PMA samples generally exhibited more than 500 different rOTUs (511 to 1007), whereas samples treated with PMA had a much lower microbial richness ranging from 2 to 190 different rOTUs. A statistical comparison (paired student's t-test) of PMA treated to non-PMA samples resulted in a p-value of <0.005 indicating a highly significant reduction of the microbial richness in PMA treated samples. On abundance level, OTUs were analyzed with regard to increase after PMA treatment. Here, 14 different rOTUs produced a significant p-value (<0.05, paired student's t-test), which all belonged to the phylum *Proteobacteria* in the class *Betaproteobacteria/Gammaproteobacteria*. The 14 rOTUs were classified as *Bradyrhizobiaceae*, *Phyllobacteriaceae*, *Erythrobacteraceae*, *Sphingomonadaceae*, and *Pseudomonadaceae*. Consequently, the abundance of these rOTUs was underestimated when non-PMA sample data were analyzed. Focusing on microorganisms that get selectively reduced due to cleanroom conditions, rOTU abundances were first rank-normalized across each array and then aggregated at genus level. Genera that decreased or increased at least 25% in their relative rank in both cleanroom samples compared to both non-cleanroom samples were filtered from the entire genus dataset. These genera are displayed in Fig. 7 and belonged to various phyla. Since PMA and non-PMA samples were treated separately, some rOTUs showed an increase in PMA samples but a decrease in non-PMA samples. This effect can be attributed to corresponding amount of DNA signatures from non-intact cells in the samples, which could have a masking effect. Fig. 7 depicts 48 different genera, which showed some congruence with the pyrotagsequencing predicted changes (e.g. *Paenibacillus*). However, when considering the PMA-treated samples, information regarding the reduction of microbial signatures due to potential cleaning efforts can be gained. For instance, when considering only the intact fraction of cells, staphylocci were enriched in the less controlled environments of the changing room and the checkout room. In contrast, the non-PMA samples exhibited similar aggregated ranks of *Staphylococcus* signatures in cleanroom and changing room samples, while only the checkout room exhibited less prominent signatures. Consequently, *Staphyloccus* appeared to get reduced due to the controlled environment of cleanrooms.

### The changing room revealed the lowest diversity but the highest abundance of microbial signatures

For a comparative analysis of 16S rRNA gene cloning, pyrotagsequencing and PhyloChip G3 technology representative sequences of OTUs were classified with the same taxonomic tool against the same database (see Methods for details). Measures of microbial diversity of pyrotagsequencing and PhyloChip G3 showed that the changing room harbored the lowest diversity in the cleanroom facility (Shannon-Wiener indices are provided in Table 1).

PMA pretreatment (detection of intact cells) was performed for experiments with PhyloChip G3 and 16S rRNA gene cloning. PMA treated samples, which were analyzed by PhyloChip G3, revealed a significant decrease in their diversity indices compared to the total microbial fraction (p-value 0.026, paired student's t-test). Concerning microbial richness measure, no correlation of number of OTUs in non-PMA treated samples was found when comparing the different methodologies (p-value > 0.05). However, when the OTUs were grouped at genus level, OTUs derived from PhyloChip G3 experiments (rOTUs) and pOTUs (OTUs obtained from pyrotagsequencing) showed a significant correlation of the microbial richness measure (p = 0.003, Pearson's r = 0.997, Fig. 8). A paired student's t-test testing for differences between genus richness of PMA and non-PMA samples produced a significant result for cloning (p = 0.011) and highly significant result for PhyloChip G3 data (p = 0.001). Thus, PMA-treated samples clearly show different richness than non-PMA samples. With regard to the agreement of PhyloChip G3™ and pyrosequencing, 62% of all genera detected by PhyloChip G3 technology were also detected via 454 pyrosequencing as depicted in Fig. 8. 16S rRNA gene cloning revealed seven genera, which were not detected by PhyloChip G3 or 454 pyrosequencing. Fig. 9 displays the microbial richness of genera detected in each sample grouped at phylum level (class level for *Proteobacteria*). The changing room (UR) generally showed the lowest amount of different genera detected by all three methods employed. However, as found with cultivation-dependent and -independent methods, the changing room (UR) revealed the highest contamination level with respect to colony forming units and detectable 16S rRNA genes after PMA treatment (Table 1).

### All methods revealed different microbiomes present in controlled and uncontrolled areas

Adonis testing (Refs. 27, 28) based on abundance metrics produced a significant p-value for PMA versus non-PMA samples (0.034 for cOTUs (cloning), 0.036 for rOTUs (PhyloChip G3); experiment was not performed for pyrosequencing) indicating that PMA treated samples harbored a different microbiome structure than non-PMA samples. Ordination analyses based on rank-normalized abundance scores of cOTUs and rOTUs (Fig. 10) showed a separation of PMA-treated samples. This is in accordance with the above mentioned significant p-value in the Adonis test. Moreover, ordination analysis showed for all three methods employed (16S rRNA gene cloning, pyrosequencing and Phylochip) that samples taken from cleanrooms (CR) group together apart from other samples (check out room (CO), changing room (UR)) considering PMA treated and non-PMA samples separately. Similar observations were made for HC-AN analysis with the exception of clone library data, which were, however, only based on few counts in the PMA-treated samples.

### The archaeal microbiome was predominated by *Thaumarchaeota* representatives

Archaeal 16S rRNA gene signatures were detected for each locations, whereas the CR5 facility revealed slightly higher qPCR signals than CR8 (1.7 × 10^5^ and 0.9 × 10^5^, respectively^25^). The archaeal diversity was investigated by pyrotagsequencing of 16S rRNA gene amplicons (Table S7 and included figure). OTU grouping revealed five (CR5) to 19 OTUs (CO), which were assigned to two archaeal phyla (*Thaumarchaeota* and *Euryarchaeota*, Table S7). The dominant lineage (*Candidatus* Nitrososphaera) accounted to 55–92% of all reads of each location. Signatures of halophilic archaea (*Halobacteriaceae*) were found in all sampled rooms, whereas *Halococcus* signatures appeared highly abundant in CR8 (43%). Signatures of *Methanocella* were detected in the check-out facility (CO). Cloning of 16S rRNA genes revealed the presence of signatures from unclassified (Eury)archaeota in CR8 as well as from Candidatus *Nitrososphaera* (both cleanrooms). Halophilic archaea have not been detected by 16S rRNA cloning^25^.

### Network analyses allowed tracking of the microbial routes and identified the changing room as most critical contamination source for the cleanrooms

All sampled rooms shared certain OTUs as presented in the network analyses (see supplementary Fig. S1 for pyrotagsequencing and supplementary Fig. S2 for PhyloChip analysis). Network tables were generated in QIIME (see Material and Methods and supplementary real node and edge tables S9.1, S9.2, S10.1 and S10.2) and visualized in Cytoscape. Lower amounts of pOTUs were shared outside the cleanroom (CO and UR, 18 pOTUs), than inside cleanrooms CR8 and CR5 (39 pOTUs). pOTUs detected in UR were spread to the highest relative proportion (68%) throughout the cleanroom facility. Although high in relative abundance and taxonomic resolution only a few pOTUs were common in all four sample locations (68 pOTUs), many were grouped at two (204 pOTUs) or three locations (115 pOTUs). The network revealed a similar portion of exclusive pOTUs in both cleanrooms (208 pOTUs in CR8 and 180 pOTUs in CR5), in contrast to CO and UR, where CO showed the highest (411 pOTUs) and UR the lowest number (76 pOTUs) of exclusive pOTUs. Similar patterns could be observed for rOTUs derived from PhyloChip data with the following exceptions: Most rOTUs were common in two sample locations (654 rOTUs). Portions of exclusive rOTUs were highest in CR8 (393 rOTUs) followed by CO (356 rOTUs) and lowest in CR5 (185 rOTUs) and UR (174 rOTUs). Beside UR, rOTUs were spread to the highest relative proportion in CR5 as well (~66% both rooms). Additional patterns were detected by the use of PMA treatment of samples. Hence, rOTUs from UR spread high, but only the smallest fraction (compared to all other samples) were derived from uncompromised cells (14% relative proportion). In contrast, almost all rOTUs from CR8 were represented by intact cells (59% CR8_PMA compared to 61% CR8).

## Discussion

HEPA air filtration, control of humidity and temperature, partial overpressure (ISO 5), frequent cleaning, limited number of persons working at the same time in a cleanroom and strict changing protocols – all these cleanroom maintenance procedures have strong impact on the abundance, viability and diversity of microorganisms therein. Such countermeasures, performed in order to decrease particulate contamination, result in the development of clearly distinct microbial communities in controlled and uncontrolled facility areas.

Firstly, the abundance of molecular microbial signatures and colony forming units was tremendously reduced within the cleanrooms compared to changing and office area. This has been proven true via four different methods. The changing room revealed the highest CFU numbers in all cultivation assays (except heat-shock resistant bioburden) and the highest number of 16S rRNA gene signatures per m^2^ (PMA-qPCR), whereas lowest numbers were detected in CR5 in these experiments (except cultivation of alkaliphiles, which also revealed 0 in CR8). The microbial abundance with respect to CFU thus decreased from UR to CR5 by a factor of 43 (oligotrophs), 431 (alkaliphiles), 444 (anaerobes), 10 (heat-shock resistant bioburden), and the 16S rRNA gene numbers by a factor of 6 (qPCR) and 40 (PMA-qPCR; Table 1). Secondly, the portion of intact cells decreased immensely: Only 10% (CR8) and 1% (CR5) of the qPCR signals obtained from the cleanroom samples were judged to be derived from intact, and thus possibly living cells. These values are in in the range of previously reported numbers for cleanroom facilities^29^. However, the ratio of these probably living cells was tremendously higher for the changing room (UR; 45%), which is in congruence with the cultivation-based experiments, revealing a decrease of the cultivable microbial portion towards cleanrooms by at least 10 fold. Thirdly, the cleanroom areas are most likely highly influenced by the human microbiome. Although each investigated room harbored its indigenous microbiome, a low, but general overlap of microbial diversity was found. In particular *Staphylococcus, Micrococcus, Corynebacterium, Propionibacterium, Clostridium*, and *Streptococcus* were detected by different methods in all facility areas, implying the major source of bacteria in these facilities: the human body. Fourthly, the cleanroom maintenance procedures clearly impacted the microbial diversity. Cultivation experiments revealed several microbial genera, which were exclusively found in the cleanrooms, including *Staphylococcus* (*S. lugdunensis, S. pettenkoferi*), *Erwinia* and *Cellulomonas*. Noteworthy, *S. lugdunensis*, a typical human skin commensal^25^,^30^, did not appear in any other area except CR5. This finding indicates the presence of a potential “hot spot” for these microorganisms and an increased contamination risk via human activity in this area. Although staphylococci are clearly human-associated and thus might not embody a risk for planetary protection considerations, their presence could have severe influence on planetary protection bioburden measurements: Cleanroom *Staphylococcus* species were shown to be able to survive heat-shock procedures which are the basis for contamination level estimations^31^. However, when comparing microarray data from intact versus non-intact cells, a strong decrease of *Staphylococcus* signatures was found for cleanroom samples, although their diversity was even higher in these areas.

The changing room represents the area of highest human activity and agitation, compared to office area and cleanrooms. In the changing area, particles and microorganisms, attached to human skin or cloths (also brought from the outer environment), are spread all over the place: into the air and onto the surfaces. Consequently, the highest abundance of 16S rRNA gene signatures from intact cells was detected in this area (1.3 × 10^7^ 16S rRNA gene copy numbers per m^2^). Noteworthy, this location also revealed the lowest microbial diversity when PhyloChip and pyrotagsequencing were applied. This finding, however, was not supported by cultivation-based experiments, pointing at a methodical problem of molecular techniques with microbial communities predominated by one or several species, which may arise from the various normalization procedures applied for these technologies. The central and important role of the changing area has been confirmed by network analyses, which revealed this location being the major source for microbial contamination possibly leaking into the cleanrooms. The changing procedure follows strict rules, thus being a completely defined and effective process to reduce the microbial (and particulate) contamination of cleanrooms. However, the microbial transport via this route, at least in our setting, could not completely be avoided. Interestingly a high portion of microbes transferred from the changing area into the cleanroom environment might be hampered to proliferate under these new extreme conditions, as revealed by network analyses of PhyloChip data. However after this selection process almost all microbes detected in the cleanroom environment (CR8) comprise intact cells (or spores), which now have a high potential to colonize new environments and products (e.g. spacecraft). As known from other studies, slight modifications in room architectures can have enormous impact on the indoor's microbiome und could help to further reduce the microbial contamination^8^. Thus, a two-step changing-room system, as it is generally established for cleanrooms of higher cleanliness levels, is certainly more effective in microbial contamination reduction. Studies of those systems, however, have not been conducted thus far.

To understand the introduction of contaminants and to estimate the risk of detected microorganisms for planetary protection or – under certain circumstances - even staff health, the natural origin as also the potential pathogenic character of the contaminants is of general interest. The genera detected via pyrotagsequencing in samples from uncontrolled environments were mostly assigned to natural environments. Particularly, soil-related genera were detected in the changing area. Noteworthy, 8% of the signatures detected in CO could be attributed to a food source. The cleanest area revealed sequences mostly from unknown sources (55%), and the lowest level of soil associated microorganisms (11%). Most bacteria with pathogenic potential were detected in UR (31%), followed by genera from the cleanroom environment (18%). The checkout room (CO) microbial community revealed the lowest pathogenic potential (13%). Relative proportions of potential beneficial microbes were higher in UR and the cleanroom CR8 (both 17%) than the checkout room CO (9%) and CR5 (7%). Interestingly, some beneficials belonging to the order of *Lactobacillales* like *Lactobacillus* and *Lactococcus* increased towards the cleanroom, and could also be associated with the human body^13^,^31^.

Members of *Bacillus*, *Staphylococcus* and *Deinococcus* (identified in the cleanroom area) are well-known for their capability to resist environmental stresses^32^,^33^. With regard to clinical environments, the reduced diversity within such areas could lead to a proliferation of bacterial species with pathogen potential and might increase the risk to acquire disease or allergic reactions^34^. This knowledge offers the possibility to use ecological knowledge to shape our buildings in a way that will select for an indoor microbiome that promotes our health and well-being. Biocontrol using beneficials like lactobacilli or the implementation of a highly diverse synthetic beneficial community would be an option, which should be evaluated for indoor areas besides cleanroomrooms^35^,^36^. Each human activity is correlated with microbial diversity; therefore sterility in cleanrooms is impossible. This requires new ways of thinking and is also important for cleanroom facilities for pharmaceutical and medical products but also for hospitals, especially intensive care units.

In our comprehensive study, using cultivation-dependent and cultivation-independent methods, we obtained further insights into the microbiology of cleanrooms. We were able to show a strong effect of cleanroom maintenance procedures on diversity, abundance and physiological status of microbial contaminants. All rooms belonging to the cleanroom facility, an office, a changing room and two cleanrooms of different ISO certification (ISO 5 and 8), harbored different microbial communities, including non-intact and intact (thus possibly living) cells. Additionally, we revealed also potential contamination sources and routes within the facility and thus identified the changing room as the area harboring the major risk for cleanroom contamination. Currently used countermeasures to avoid a severe contamination with outside- microorganisms seem to work properly, but potential risks could highly be reduced by a different architecture of the changing area.

## Methods

### Sampling sites and setting

**Figure 1:**
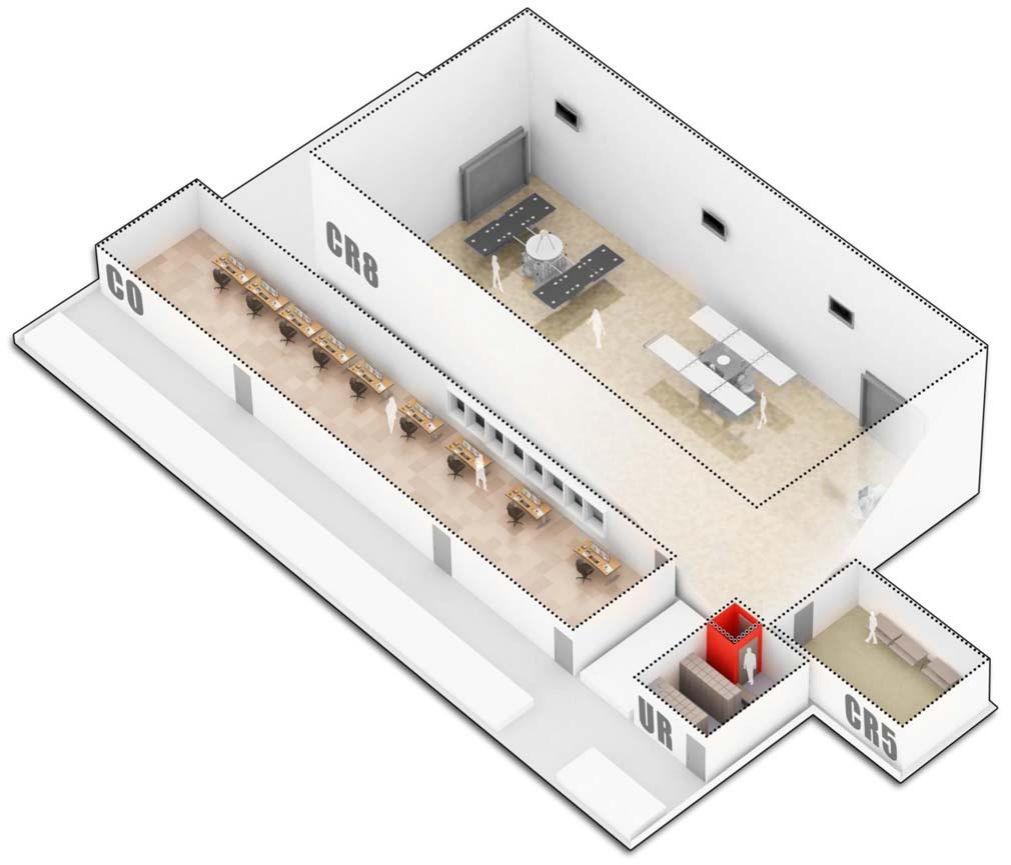
Illustration of the integration center at Airbus Defence and Space GmbH in Friedrichshafen, Germany. Sampled rooms were designated as follows: UR – changing room, CO – checkout room, CR8 – ISO 8 cleanroom, CR5 – ISO 5 cleanroom. Proportions reflect actual dimensions. Interieur decorations were abstracted and do not mirror real arrangement.

**Figure 2:**
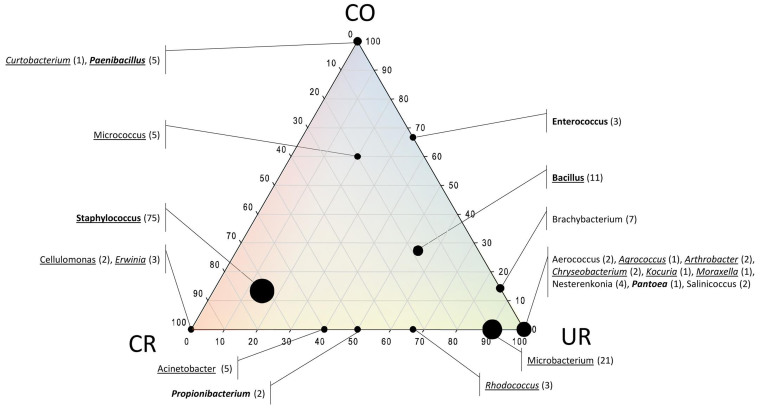
Ternary plot of isolates (genera) with respect to the sample origin (the two cleanrooms CR5 and CR8 were summarized: CR). Axes reflect the percentage of isolates detected in each location. Isolates obtained under oligotrophic conditions are underlined, isolates obtained under anaerobic conditions are printed bold, isolates obtained under alkaline conditions are printed non-italics. In brackets: number of retrieved colonies.

**Figure 3:**
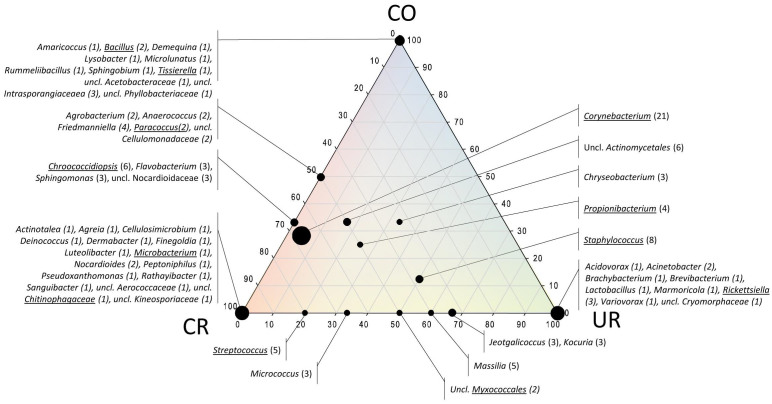
Ternary plot of detected cOTUs (bacterial 16S rRNA gene cloning) with respect to the sample origin (the two cleanrooms CR5 and CR8 were summarized: CR). Axes reflect the percentage of OTUs detected in each location; OTUs that could not be attributed to an order, family or genus were not considered. Size of dots reflects no. of detected OTUs summarized in one dot. Underlined genera were also detected when samples were treated with PMA ( = intact cells). Uncl.: unclassified.

**Figure 4:**
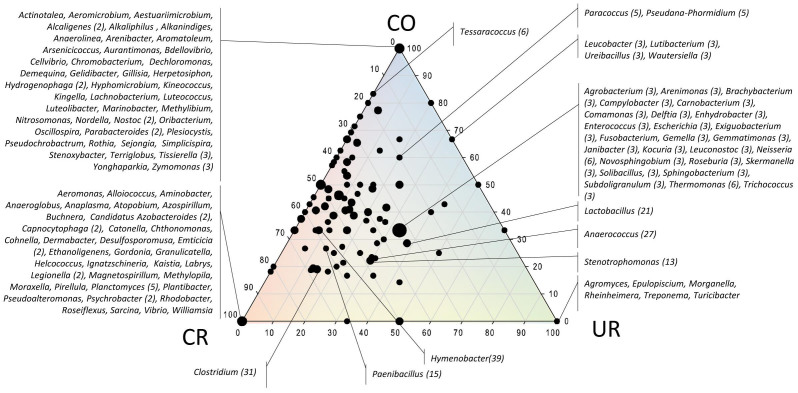
Ternary plots of detected pOTUs (454 pyrotag sequencing) with respect to the sample origin (the two cleanrooms CR5 and CR8 were summarized: CR). Axes reflect the percentage of OTUs detected in each location; OTUs that could not be attributed to a order, family or genus were not considered. Size of dots reflects no. of detected OTUs summarized in one dot (no. given in brackets if different from 1).

**Figure 5:**
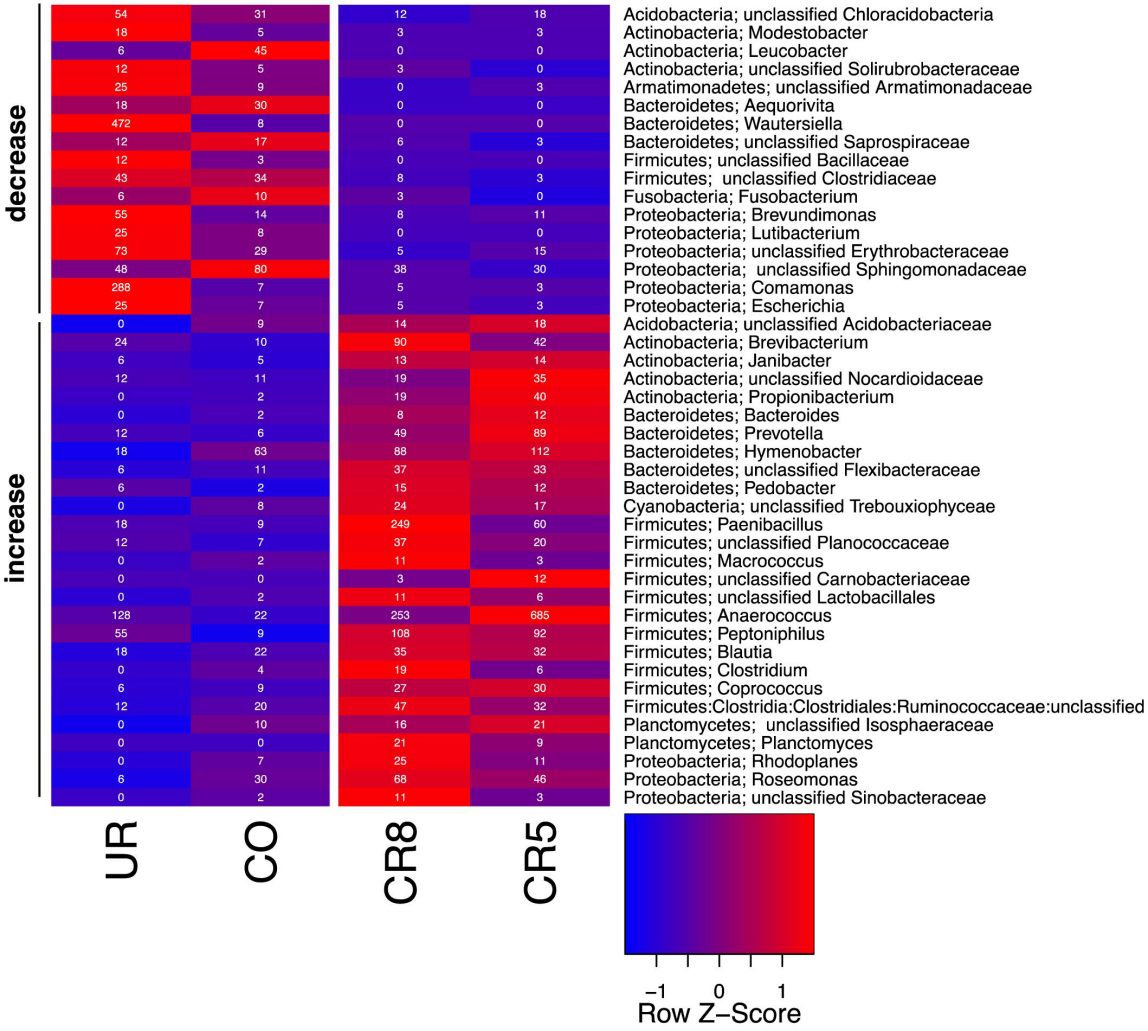
Heatmap based on 454 pyrotagsequencing data of aggregated read counts at genus level (reads were sum-normalized prior to aggregation). Displayed are genera that showed an at least 25% increase or decrease in both cleanroom samples compared to non-cleanroom samples and had a minimum number of reads of at least 10. Numbers in the cells give number of reads. For the color gradient, read scores were normalized for each genus and are presented as Z-scores.

**Figure 6:**
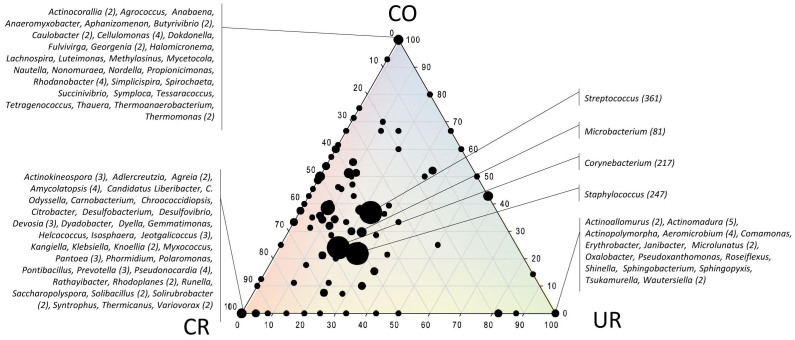
Ternary plots of detected rOTUs (PhyloChip, non-PMA treated sample) with respect to the sample origin (the two cleanrooms CR5 and CR8 were summarized: CR). Axes reflect the percentage of OTUs detected in each location; OTUs that could not be attributed to a order, family or genus were not considered for calculation. Size of dots reflects no. of detected OTUs summarized in one dot (no. given in brackets if different from 1).

**Figure 7:**
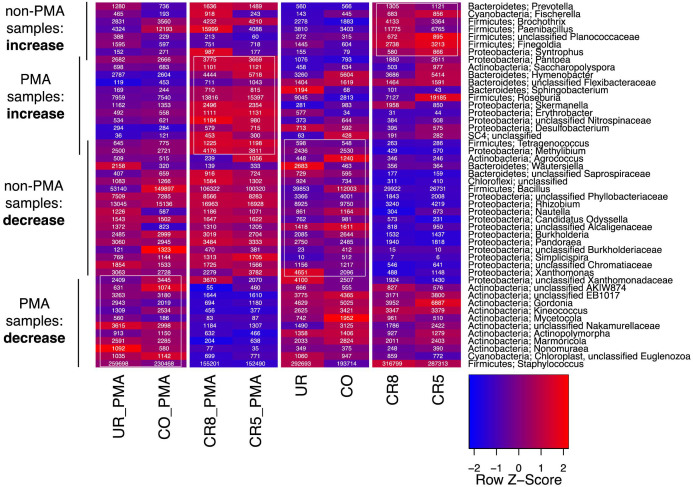
Heatmap based on summarized OTU trajectories (genus level) derived from PhyloChip G3™ data (after rank normalization of rOTUs). Displayed are genera that showed at least 25% increase or decrease in both cleanroom samples compared to non-cleanroom samples. White boxes indicate taxa that showed a 25% increase over other corresponding samples. Numbers in the cells give the summarized rank for each genus. For the color gradient, rank scores were normalized for each genus and are presented as Z-scores. Non-PMA and PMA treated samples are displayed individually.

**Figure 8:**
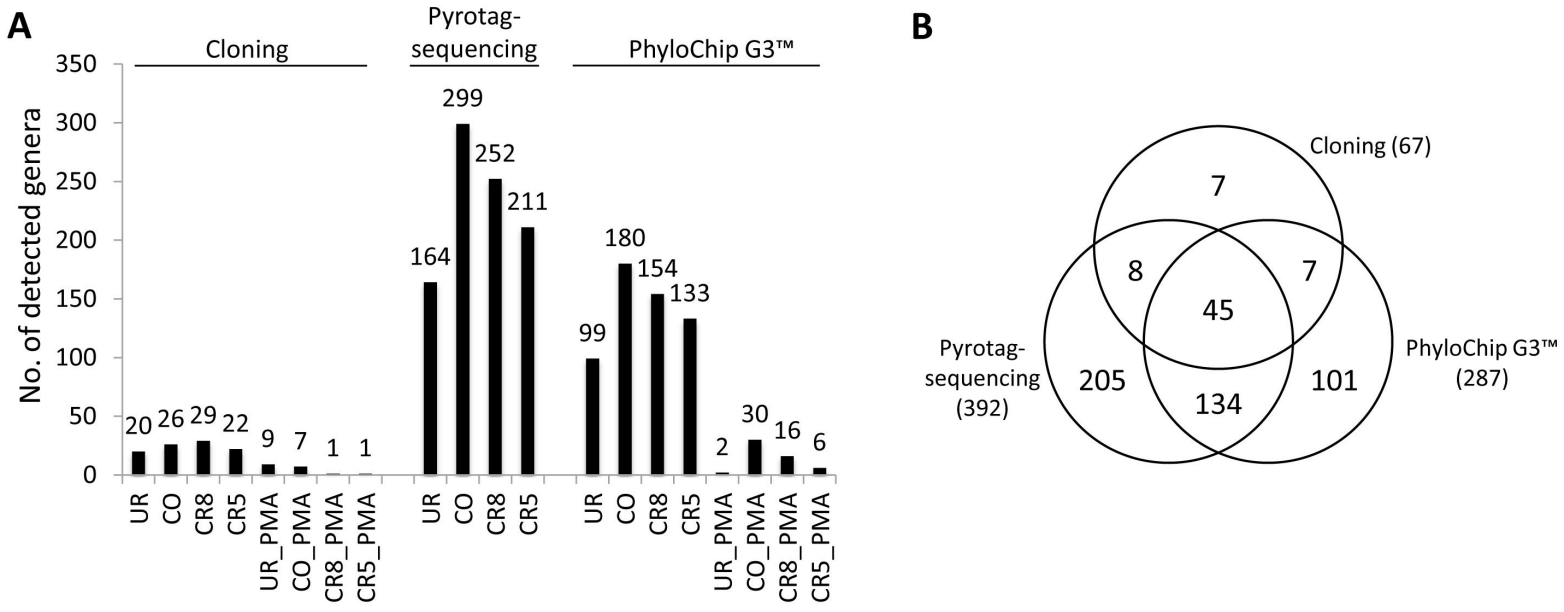
Comparison of molecular methods. All sequences were classified using the same method (Bayesian method in mothur, GreenGenes taxonomy) as indicated in Materials and Methods. (A): Richness comparison of genera detected in each sample via cloning, pyrotagsequencing, and PhyloChip G3. While no significant correlation was detected between cloning and pyrotagsequencing/PhyloChip richness, pyrosequencing and PhyloChip derived genus richness correlated highly significantly between samples (p-value = 0.003, Pearson's R = 0.997). No significant correlation was found for OTU based richness (data not shown). (B): Venn-Diagramm displaying the shared genera between the three techniques used in this study.

**Figure 9:**
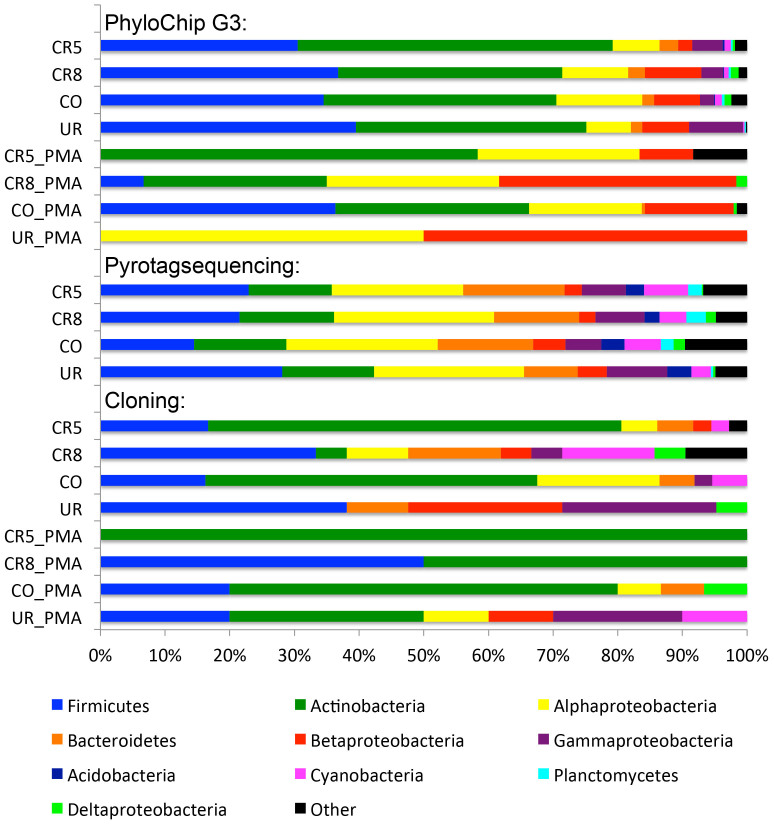
Barchart displaying the percent richness of c/p/rOTUs classified at higher taxa (phylum level, for Proteobacteria class level, incidence values of OTUs), for each sample and analysis method. All sequences were classified using the same method (Bayesian method in mothur, GreenGenes taxonomy) as indicated in the Methods section. The top ten most prominent higher taxa are shown ascending while the remaining taxa are grouped into category “other”.

**Figure 10:**
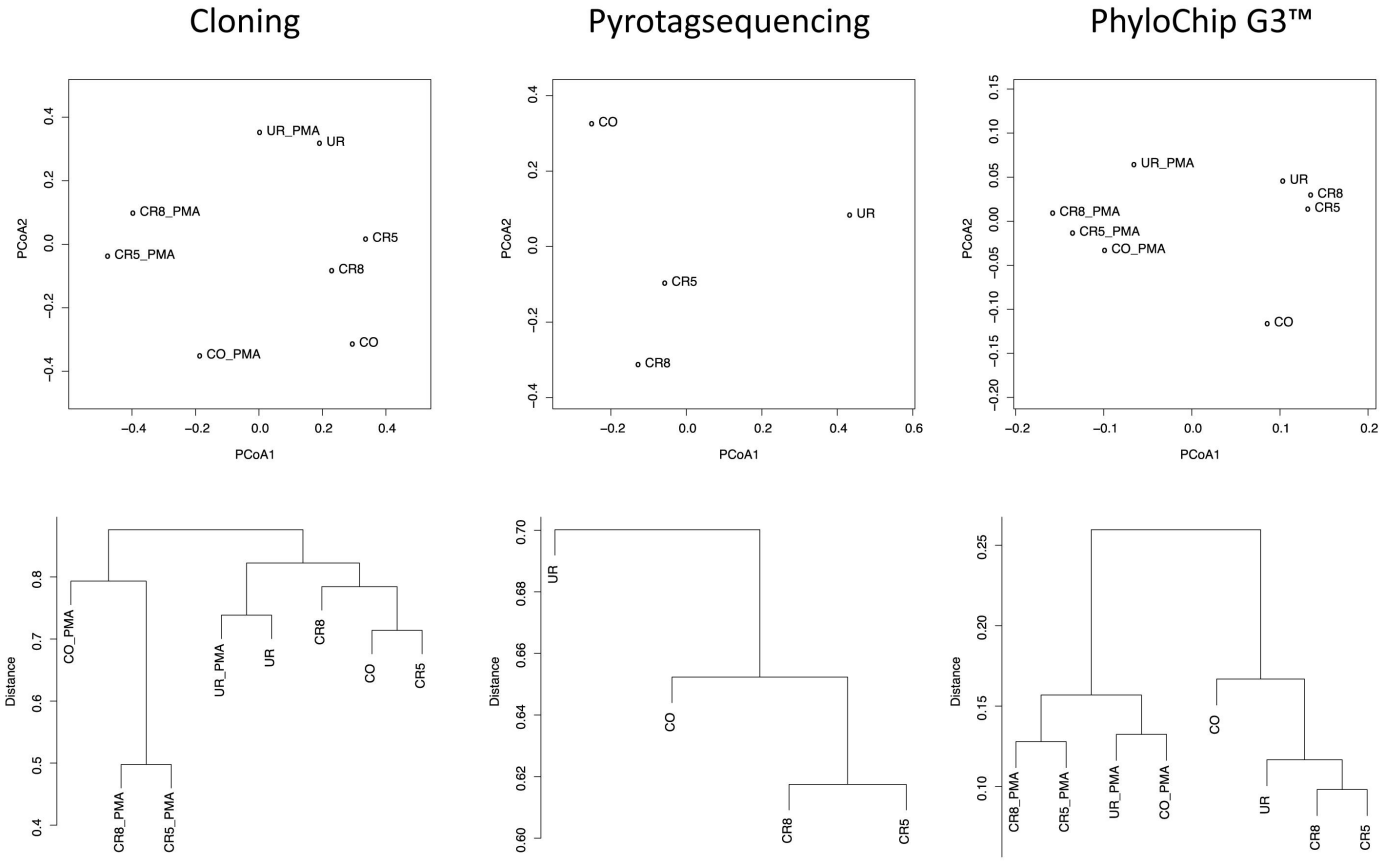
Ordination analysis and hierarchical clustering (average neighbour) of cloning, pyrosequencing and PhyloChip G3™ derived bacterial microbiomes. Analyses are based on Bray-Curtis indices of rank-normalized abundance scores of OTUs. Explained variances of PCoA axes were 29% (PCoA1) and 19% (PCoA2) for cloning, 40% (PCoA1) and 33% (PCoA1) for pyrosequencing, and 66% (PCoA1) and 13% (PCoA2) for PhyloChip data.

Sampling took place in September 2011 in Friedrichshafen, Germany. Samples were taken at various places within a cleanroom facility (integration center) maintained by the Airbus Defence and Space Division (the former European Aeronautic Defence and Space Company, EADS). In this facility, different types of indoor environments were located in close vicinity as depicted in Fig. 1. Check-out room (office and control room, CO), changing room (change room with lockers and bench, directly attached to the entrance (air lock) of the cleanrooms, UR), ISO 8 cleanroom (H-6048, CR8) and ISO 5 cleanroom (to be entered through the ISO 8 cleanroom, CR5). Both cleanrooms were maintained according to their classification (ISO 14644; HEPA air filtration, control of humidity and temperature) and were fully operating. Particulate counts in cleanroom ISO 8 determined within three days before sampling did not exceed 10.000 particles (0.5 μm) and 100 particles (5.0 μm) per ft^3^ (~0.028 m^3^), respectively, and therefore exhibited contamination levels well within specifications. Cleanroom ISO 5 was maintained with overpressure. These indoor environments reflect different levels of human activity, presence of particles (CO, UR: uncontrolled; CR8: 3.5 × 10^6^ and CR5: 3.5 × 10^3^ particles ≥ 0.5 μm), clothing (CO: streetwear; UR: changing area; CR8 cleanroom garment; CR5 complete covered cleanroom garment), entrance restrictions (CO to CR5 increasing restrictions), cleaning regimes (CO and UR household cleaning agents; CR8 and CR5 alkaline cleaning agents or alcohols) and environmental condition controls (CO and UR uncontrolled conditions; CR8: 0.5 air change per min, filter coverage 4–5%, filter efficiency 99.97%, vinyl composition tile on floors; CR5: 5–8 air change per min, filter coverage 60–70%, filter efficiency 99.997%, vinyl or epoxy on floors). As given above, sample abbreviations were as follows: CO (check-out room), UR (changing room), CR8 (ISO 8 cleanroom), CR5 (ISO 5 cleanroom).

### Sampling and sample processing

All areas (CO, UR, CR8, CR5) were sampled individually and in parallel. Samples were collected from floor (areas of 1 m^2^ maximum (1 sample) and 0.66 m^2^ (all other samples)) by using BiSKits (biological sampling kits, Quicksilver Analytics, Abingdon, MD, USA) for molecular-based analysis and wipes (TX3211 Sterile Wipe LP, polyester; Texwipe, Kernersville, NC, USA; 15 × 15 cm; wipes were premoistened with 4 ml of water before autoclaving) for cultivation-based assays. Overall, 74 samples were taken (see supplementary Fig. S3). BiSKit samples (four from each room) for molecular analyses were pooled according to the area sampled and immediately frozen on dry ice. Wipe samples (four per location for bioburden analysis, eight per room for alternative cultivation strategies) were stored on ice packs (4–8°C) and microbes were extracted immediately after return to the laboratory for inoculation of cultivation media (within 24 h after sampling). In sum, 10 field blanks were taken as process negative controls.

### Cultivation

Wipes were extracted in 40 ml PBS buffer (for sampling, extraction and cultivation procedures of anaerobes: please refer to Ref. 34. For the cultivation of oligotrophic microorganisms, 5 × 1 ml of the sample was plated on RAVAN agar (including 50 μg/ml nystatin; Ref. 34). Alkaliphilic or alkalitolerant microbes were grown on R2A medium, pH 10 as given earlier (Ref. 24). Facultatively or strictly anaerobic bacteria were cultivated on anoxic TSA plates^36^; 4 × 1 ml was plated and plates were incubated under nitrogen gas phase. Incubation was performed at 32°C for 8 (alkaliphiles), 11 (anaerobes) and 12 days (oligotrophs), respectively. Additionally, the microbial bioburden was determined following the ESA standard ECSS-Q-ST-7055C (wipe assay for bioburden (heat-shock resistant microbes) and vegetative microorganisms). Sampling and wipe-extraction details were also described earlier (Ref. 29). In brief, wipe samples (in 40 ml water) were split into two portions, whereas one aliquot was subjected to heat-shock treatment (80°C, 15 min). Sample was pour-plated in R2A medium (4 × 4 ml). Samples for vegetative microorganisms (not subjected to heat-shock) were pour-plated similarly. Cultivation was performed at 32°C for 72 hours (final count).

### Isolate processing and taxonomic classification

Isolates were purified by two subsequent streak-outs and sent to DSMZ (Leibniz institute DSMZ, Deutsche Sammlung von Mikroorganismen und Zellkulturen, Braunschweig, Germany). At DSMZ, strains were classified using MALDI-TOF MS (matrix assisted laser desorption/ionization time of flight mass spectrometry) or 16S rRNA gene sequencing. MALDI-TOF mass spectrometry was conducted using a Microflex L20 mass spectrometer (Bruker Daltonics) equipped with a N_2_ laser. A mass range of 2000–20.000 m/Z was used for analysis. MALDI-TOF mass spectra were compared by using the BioTyper (Bruker Daltonics) software package for identification of the isolates. Currently the MALDI Biotyper reference library covers more than 2,300 microbial species. Strains which could not be identified by MALDI-TOF, were identified by 16S rRNA gene sequence analysis.

### DNA extraction for molecular assays

Due to the low-biomass-nature of the samples and the recurrent observation of an inhomogeneous microbial distribution in cleanrooms (see also Ref. 31), the samples were pooled by facility room for molecular analyses (4 BiSKit samples per location) in order to allow a more accurate estimation of microbial diversity. Pooled BiSKits samples were thawed gently on ice over night and concentrated. 1/5 of each sample was treated with propidium monoazide (20 mM) as described elsewhere (Ref. 2) for masking free DNA. Covalent linkage was induced by light (3 min, 500 W). In general, all samples were subjected to bead-beating for DNA extraction (PowerBiofilm RNA Kit Bead Tubes, MO BIO, Carlsbad, CA, USA; 10 min vortex). Supernatant was harvested after centrifugation (5200× g, 4°C, 1 min) and bead-washing with 400 μl DNA-free water and subsequent additional centrifugation (100× g, 4°C, 1 min). DNA was extracted from PMA-treated and untreated samples using the XS-buffer method as described earlier^37^. The resulting pellet was solved in 15 μl DNA-free water.

### Quantitative real-time PCR

QPCR was performed as described earlier^10^. One microliter of extracted DNA was used as template and amplification was performed with Bacteria- and Archaea-targeted primers using the SYBR Green system. As a reference, 16S rRNA gene amplicons of *Methanosarcina barkeri* (archaeon) and *Bacillus safensis* (bacterium) were used for generation of a standard curve. QPCR was performed in triplicates for each sample.

### Cloning and sequencing of bacterial 16S rRNA gene amplicons

Cloning of archaeal and bacterial 16S rRNA genes was performed as described earlier (Ref. 31). For the analysis of bacterial 16S rRNA genes from PMA-untreated samples, each 96 clones were analyzed; additionally 48 and 72 clones were picked for samples from the cleanrooms (CR5 and CR8, respectively). 48 clones were analyzed from PMA-treated samples. Cloned 16S rRNA genes were RFLP analyzed (*Hinf*I, *Bsu*RI), representative inserts were fully sequenced and chimera-checked (Bellerophon^3^; Pintail^38^). The sequences were submitted to GenBank (accession nos: JQ855509-635) and grouped into operational taxonomic units (OTUs; later referred to as cOTUs). Coverage was calculated according to Good, 1953^39^.

### 454 pyrotagsequencing analysis of bacterial and archaeal 16S rRNA genes

For bacterial diversity analyses, DNA templates from all four rooms were amplified using the bacteria-directed 16S rRNA gene primers 27f and 1492r (5 μM each^40^), followed by a second (nested-) PCR with tagged primer Unibac-II-515f_MID and untagged Primer Unibac-II-927r_454 (10 μM each^41^). Polymerase chain reactions were accomplished with Taq & Go ™ (MP Biomedicals) in 10 μl 1^st^ PCR – and 30 μl 2^nd^ PCR reaction mix as follows: 95°C 5 min, 30 cycles of 95°C 30 s, 57°C 30 s, 72°C 90 s and 72°C for 5 min after the last cycle; 32 cycles were applied for the 2^nd^ PCR with the following parameters: 95°C 20 s, 66°C 15 s, 72°C 10 min. Archaeal PCR-products were obtained by nested PCR as described earlier^25^. The first PCR was performed using Archaea-directed primers 8aF and UA 1406R primers^42^,^43^. The second PCR included 5 μM primers (340F_454 and tagged 915R_MID^44^,^45^), 6 μl Taq-&Go™ [5×], 0.9 μl MgCl_2_ [50 mM] and 3 μl PCR product of the first archaeal PCR in a final volume of 30 μl. An optimized temperature program for primers with a 454 tag included the following steps: initial denaturation 95°C 7 min, 28 cycles with a denaturation at 95°C for 30 s, annealing at 71°C for 30 s, elongation at 72°C for 30 s, repetitive cycles were concluded with a final elongation at 72°C for 5 min. After PCR, amplified products were pooled respectively and purified using the Wizard® SV Gel and PCR Clean-Up System (Promega, Madison, USA) according to manufacturer's instructions. Pyrotagsequencing of equimolar PCR products was executed by Eurofins MWG Operon (Ebersberg, Germany) on a Roche 454 GS-FLX+ Titanium™ sequencer. Resulting 454 reads (submitted to QIIME-DB, http://www.microbio.me/qiime/ as Study 2558) were analyzed using the QIIME^46^ standard workflow as described in the 454 Overview Tutorial (http://qiime.org/tutorials/tutorial.html) and briefly summarized in the following: Denoising of pyrotagsequencing reads of the four samples (CO, UR, CR5 and CR8) resulted in 1003-5118 bacterial and 890-2725 archaeal sequences. OTUs (later referred to as pOTUs) were grouped at 97% similarity level using uclust^47^ picking the most abundant OTU as representative. Sequences were aligned using PyNAST^48^. An OTU table was created after removing chimeric sequences (561) via ChimeraSlayer (reference: greengenes 12_10 alignment) and filtering the PyNAST alignment. All pOTUs detected in the extraction blank were removed as potential contaminants from the entire sample set, which resulted in 816-2982 sequences (267–855 pOTUs) for bacteria and 47-229 sequences (5–14 pOTUs) for archaea. pOTU networks were visualized using Cytoscape 2.8.3 layout edge-weighted spring embedded eweights^49^.

### PhyloChip G3™ DNA microarray analysis of bacterial 16S rRNA gene amplicons

The basic of PhyloChip G3™ data acquisition and analysis can be found in the supplementary information of Hazen et al., 2010^50^. In brief, bacterial amplicons were generated as described above for 16S rRNA gene cloning with primer pair 9 bf and 1406ur^40^. After quantification, amplicons were spiked with a certain amount of non-16S rRNA genes for standardization, fragmented and biotin labeled as described in the abovementioned reference. After hybridization and washing, images were scanned. Raw data processing followed the principle of stage 1 and 2 analysis described in Hazen et al., but with modified parameters. First, an updated Greengenes taxonomy was used for assigning rOTUs (“reference-based OTUs”) to the probes^51^. Second, only those probes were included in the analysis that corresponded to the targeted 16S rRNA gene region of the amplicons generated with 9 bf and 1406ur primers. Third, at minimum seven probes were considered for an OTU and the positive fraction of scored versus counted probes was set to 0.92. The quartiles of the ranked *r* scores (response score to measure the potential that the probe pair is responding to a target and not the background) were set to *r*Q_1_ ≥ 0.80, *r*Q_2_ ≥ 0.93, and *r*Q_3_ ≥ 0.89 for stage 1 analysis. For stage 2, the *r*x values (cross-hybridization adjusted response score) was set to *r*xQ_1_ ≥ 0.22, *r*xQ_2_ ≥ 0.40, and *r*xQ_3_ ≥ 0.42. These adjusted parameters are considered sufficiently stringent for cleanroom diversity measures. Calculated hybridization values for each OTU were log_2_*1000 transformed. As different amounts of PCR product were loaded onto the chips (PCR reactions performed differently for each sample, particularly for those that were PMA treated) abundance values were rank normalized across each array and are referred to as hybridization scores/abundances.

### Taxonomic classification of 16S rRNA genes

16S rRNA gene amplicons were classified using the Bayesian method implemented in mothur (cutoff 80%, Refs. 52, 53) against an updated Greengenes taxonomy^51^, which was manually curated and in which OTUs were grouped at 98% similarity level. For taxonomic comparison of rOTUs (obtained from PhyloChip analysis) against amplicon generated OTUs (cOTUs, pOTUs), representative sequences of rOTUs were also classified with this method.

### Microbial diversity measure

Shannon-Wiener indices were computed of all samples using the R programming environment^54^. Phlyochip G3 abundance data was multiplied with binary data, i.e. using abundance data of only those rOTUs that were called present in a sample. Abundance data of clone libraries, pryotagsequencing libraries and microarray data were individually rarefied to the lowest number of OTU abundances in the sample set and the Shannon-Wiener index was calculated for each sample. To avoid statistical errors originating from rarefication, the procedure was performed 1000 times and the average Shannon-Wiener index of each sample was calculated.

### Cytoscape OTU networks

Node and edge tables (see supplementary Tables S9.1, S9.2, S10.1 and S10.2) for OTU networks were generated in QIIME and visualized in Cytoscape 2.8.3^49^. Shared OTUs were colored according to their presence in each sample (color mixtures were applied according to the color circle of Itten). OTUs as well as samples were displayed as nodes in a bipartite network. Both were connected via edges if their sequences were present in that sample. Edge weights (eweights) were calculated according to the sequence abundance in an OTU. For network clustering of OTUs and samples a stochastic spring-embedded algorithm was used with a spring constant and resting length. Nodes were organized as physical objects on which minimized force was applied to finalize the displayed networks.

### Statistical analysis

Multivariate statistics were employed for microbial community analysis^54^. Bray-Curtis distance was calculated from the clone library, the pyrotagsequencing and PhyloChip G3 abundance data, which were all rank-normalized. Principal Coordinate Analysis (PCoA) and Hierarchical Clustering based on Average Neighbour (HC-AN) was performed to analyze the microbiome relatedness of the samples. Adonis testing was used to investigate if PMA treatment of samples had a significant effect on the microbial community structure observed. Paired student's t-test was performed to find significant difference between qPCR data of PMA and non-PMA samples.

### Identification of enriched genera

HybScores of rank-normalized OTUs were aggregated at genus level for PhyloChip data. Considering pyrotagsequencing data, sum-normalized reads (5000 per sample) were summarized at genus level. In order to identify those genera that were enriched in cleanroom samples versus others, a 25% increase of aggregated scores was used as a threshold. In a similar manner, a 25% decrease of HybScores/sequencing reads was used as an indicator for genera that declined in cleanroom samples.

### Controls and blanks for molecular analyses and cultivation

Control samples were included in each step of the extractions and analyses. Field blanks (procedure see: Ref. 31), extraction blanks (for BiSKit samples: unopened PBS included in the kit was used for extraction), water blanks and no-template controls (for PCR), as well as media blanks were processed. If not stated otherwise no signal or positive cultivation result was obtained thereof. For bacterial 16S rRNA analyses, detected OTUs (for cloning, pyrotagsequencing and PhyloChip) were removed from the entire analysis (Table S8). Bacterial copies detected in qPCR negative controls were subtracted from sample values.

## Author Contributions

Conceived and designed the experiments: C.M.E., A.J.P. and G.B. Performed the experiments: A.K.A., A.J.P., A.M., L.T., Y.P., S.B. and R.P. Analyzed the data: A.J.P. and A.M. Contributed reagents/materials/analysis tools: G.A., K.V. and P.R. Wrote the paper: C.M.E., A.J.P., A.M. and G.B. All authors contributed to the manuscript revision.

## Supplementary Material

Supplementary Information Supplementary Information

## Figures and Tables

**Table 1:** Microbial abundance and diversity, determined by cultivation- and molecular-based methods

Cultivation dependent (abundance)		location			
		CO	UR	CR8	CR5
CFU per m^2^	Oligotrophs	2.3 × 10^3^(0.6–4.0 × 10^3^)	17.2 × 10^3^(10.4–23.9 × 10^3^)	15.7 × 10^3^(0.1–31.3 × 10^3^)	0.4 × 10^3^(0–0.7 × 10^3^)
	Alkaliphiles	0.6 × 10^3^(0–1.2 × 10^3^)	1.9 × 10^3^(0.7–3.0 × 10^3^)	BDL	0*(0–0.01 × 10^3^)
	Anaerobes	9.9 × 10^3^(6.2–13.6 × 10^3^)	44.4 × 10^3^	4.1 × 10^3^(3.3–4.9 × 10^3^)	0.1 × 10^3^(0–0.3 × 10^3^)
	Spore Bioburden (heat-shock: 80°C; 15 min)	0.2 × 10^3^(0–0.3 × 10^3^)	0.1 × 10^3^(0–0.2 × 10^3^)	0.08 × 10^3^(0–0.02 × 10^3^)	0.01 × 10^3^(0–0.01 × 10^3^)
	Bioburden (cultivable counts without heat-shock)	3.2 × 10^3^	TNTC	0.7 × 10^3^(0.3–1.3 × 10^3^)	0.3 × 10^3^(0.2–0.6 × 10^3^)
		(0.8–4.5 × 10^3^)	(4.3 × 10^3^- TNTC)		

Cultivation independent (abundance)		CO	UR	CR8	CR5
Total bacterial population (16S rRNA gene copies)	qPCR	3.1 × 10^7^	2.9 × 10^7^	0.3 × 10^7^	0.6 × 10^7^
	PMA-qPCR	0.2 × 10^7^	1.3 × 10^7^	0.03 × 10^7^	0.006 × 10^7^

Cultivation independent (diversity)		CO	UR	CR8	CR5
Bacterial diversity (Shannon-Wiener index)	cloning	2.39	2.21	2.15	2.19
	Cloning PMA	1.70	1.60		
	Pyrotagsequencing	6.04	4.76	5.72	5.43
	PhyloChip G3	6.83	6.22	6.89	6.44
	PhyloChip G3 PMA	5.24	0.69	4.09	2.48

*4,4.

TNTC: Too Numerous To Count.

BDL: below detection limit
