# Supplementary material for: Quo vadis? Microbial profiling revealed strong effects of cleanroom maintenance and routes of contamination in indoor environments
Source: Sci Rep. 2015 Mar 17;5:9156. doi: 10.1038/srep09156 (PMC4361859; doi:10.1038/srep09156)
Supplement: Supplementary Information [file srep09156-s1.pdf]

Supplementary Figures to:

Quo vadis? Microbial profiling of vicinal office, changing and clean rooms reveal clearly different microbiomes and routes of contamination

Christine Moissl-Eichinger[1*†], Anna K Auerbach[1†], Alexander J Probst[1†], Alexander Mahnert[2†], Lauren Tom[3], Yvette Piceno[3], Gary L Andersen[3], Kasthuri Venkateswaran[4], Petra Rettberg[5], Simon Barczyk[5], Rüdiger Pukall[6], Gabriele Berg[2]

[1]Institute for Microbiology and Archaea Center, University of Regensburg, Universitaetsstrasse 31, 93053 Regensburg, Germany

[2] Institute of Environmental Biotechnology, Graz University of Technology, Petersgasse 12/I, 8010 Graz, Austria

[3] Lawrence Berkeley National Laboratory, Earth Sciences Division, 1 Cyclotron Rd., Berkeley, CA 94720, USA

[4] Jet Propulsion Laboratory, 4800 Oak Grove Drive, Pasadena, CA 91109, USA

[5] German Aerospace Center, Institute of Aerospace Medicine and Radiation Biology, Linder Höhe, 51147 Köln, Germany

[6] Leibniz Institute DSMZ - Deutsche Sammlung von Mikroorganismen und Zellkulturen GmbH, Inhoffenstraße 7 B, 38124 Braunschweig, Germany

Supplementary Fig. S1:

Network analysis of pOTUs from pyrotagsequencing data visualized with the Cytoscape 2.8.3 layout edge-weighted spring embedded eweights. Samples from four different rooms are displayed as hexagonal nodes on an abstracted map of the clean room facility in colors yellow (CO – check out room), red (UR – changing room), light blue (CR8 – ISO8 clean room) and dark blue (CR5 – ISO5 clean room). pOTUs are displayed as nodes. Node color is defined by sample source and respective color mixtures (according to Itten's color circle) are used for shared pOTUs. pOTUs resolved till genus level are labelled. Node and font size represents abdundance of pOTUs (score is indicated in the legend below). Edge width and opacity correlates with respective eweights (edge weights represent sequence abundance in respective pOTUs). Network layout (pOTUs distance) was slightly modified to fit on the abstracted map.

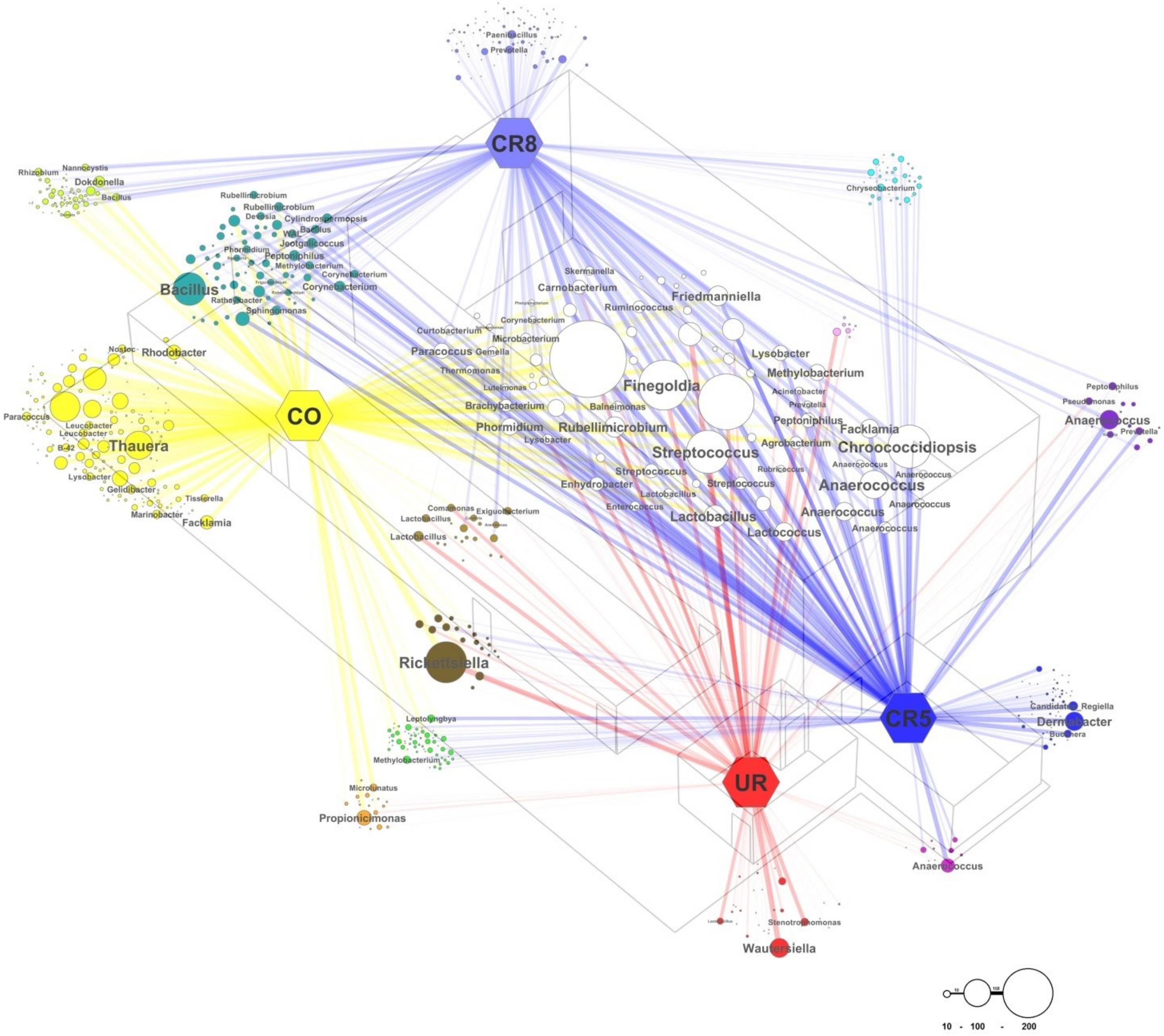

Supplementary Fig. S2:

Network analysis of rOTUs from PhyloChip data (based on binary incidences table) using Cytoscape 2.8.3 layout edge-weighted spring embedded eweights. Samples from four different rooms are displayed as octagonal nodes on an abstracted map of the clean room facility in colors yellow (CO – check out room), red (UR – changing room), light blue (CR8 – ISO8 clean room) and dark blue (CR5 – ISO5 clean room) and their corresponding PMA treated samples are shown as squares in green (CO_PMA), magenta (UR_PMA) and cyan (CR8_PMA and CR5_PMA). rOTUs are displayed as nodes. Node color is defined by sample source and respective color mixtures (according to Itten's color circle) are used for shared rOTUs. rOTUs resolved till genus level are labelled. Node and font size represents abdundance of rOTUs (score is indicated in the legend below). Edge width and opacity correlates with respective eweights (edge weights represent sequence abundance in respective rOTUs). Network layout (rOTUs distance) was slightly modified to fit on the abstracted map.

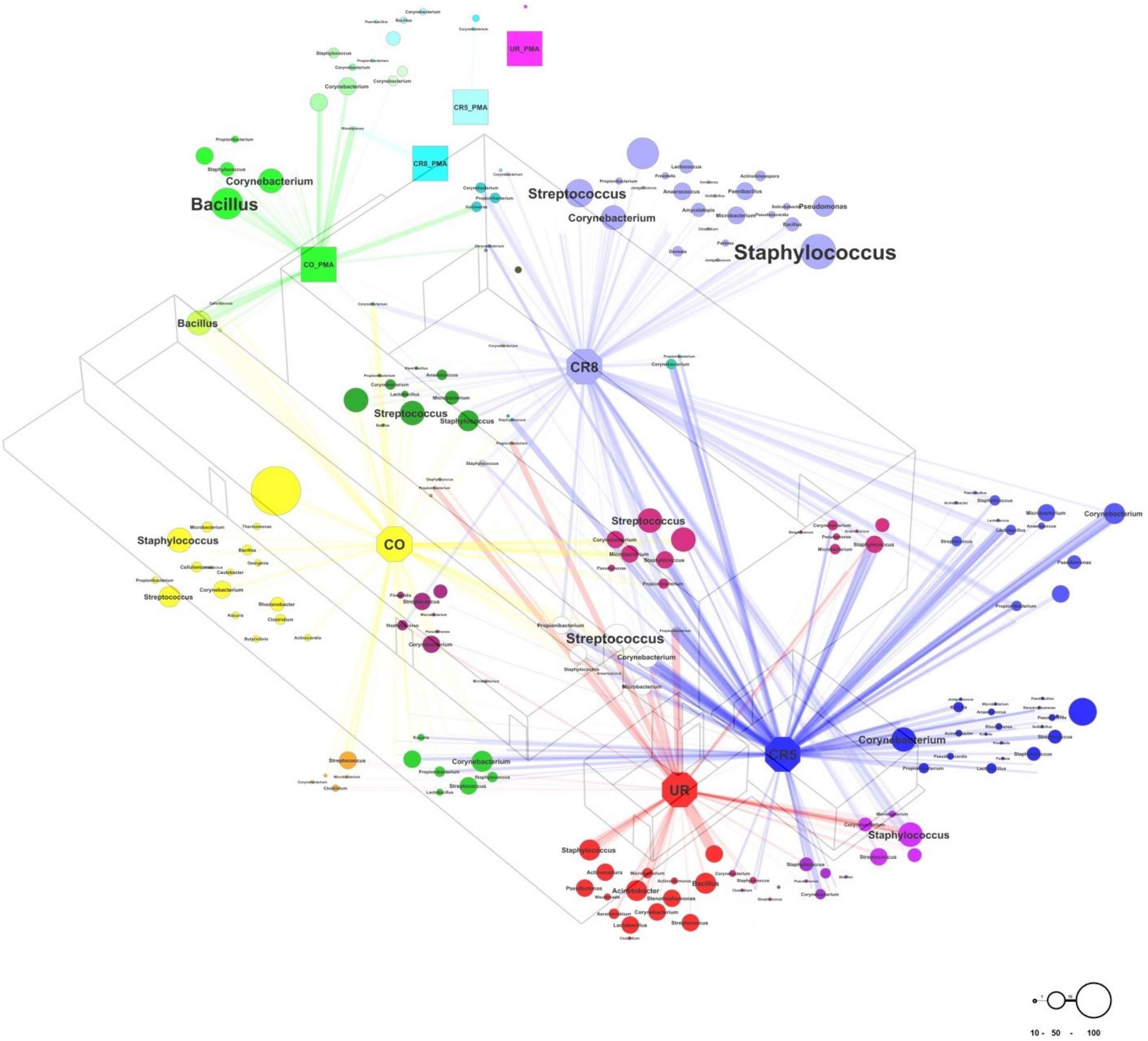

Supplementary Fig. S3:

Layout of the integration center at Airbus Defence and Space GmbH in Friedrichshafen, Germany. Sampled rooms are highlighted in red and designated as follows: UR – changing room, CO – checkout room, CR8 – ISO 8 clean room, CR5 – ISO 5 clean room. Sample location were marked as follows: Circles in yellow represent BiSKit samples, which were used for molecular analysis (cloning – cOTUs, pyrotagsequencing – pOTUs, PhyloChip – rOTUs). Circles in green represent wipe samples, which were used for cultivation of oligotrophs and alkaliphiles. Circles in blue represent wipe samples, which were used for cultivation of halophiles. Overall 16 BiSKits, 16 wipes for bioburden analysis (not displayed), 32 wipes for alternative cultivation strategies and 10 field blanks (not displayed) were taken.

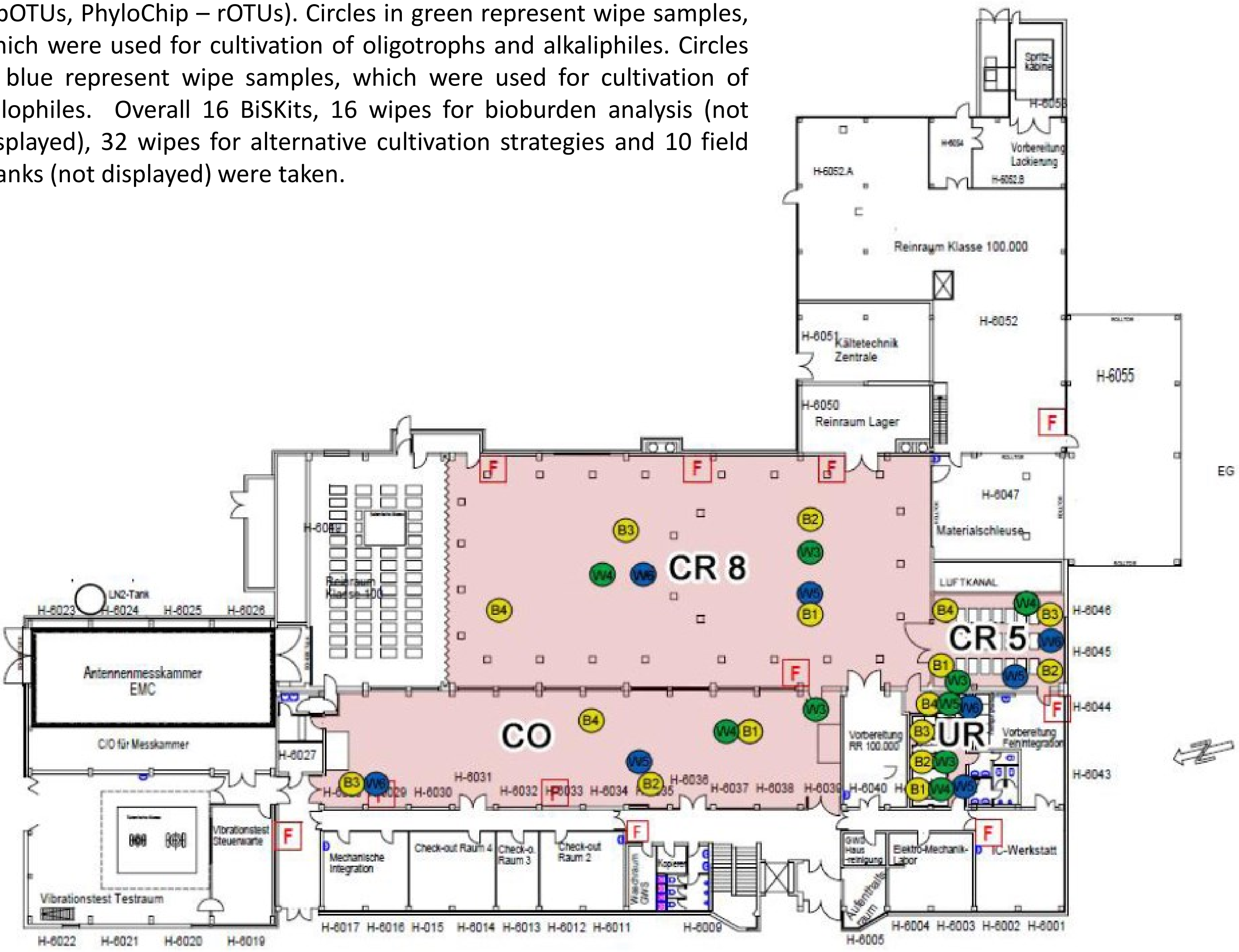

Supplementary Tables to:

Quo vadis? Microbial profiling of vicinal office, changing and clean rooms reveal clearly different microbiomes and routes of contamination

Christine Moissl-Eichinger[1*†], Anna K Auerbach[1†], Alexander J Probst[1†], Alexander Mahnert[2†], Lauren Tom[3], Yvette Piceno[3], Gary L Andersen[3], Kasthuri Venkateswaran[4], Petra Rettberg[5], Simon Barczyk[5], Rüdiger Pukall[6], Gabriele Berg[2]

[1]Institute for Microbiology and Archaea Center, University of Regensburg, Universitaetsstrasse 31, 93053 Regensburg, Germany

[2] Institute of Environmental Biotechnology, Graz University of Technology, Petersgasse 12/I, 8010 Graz, Austria

[3] Lawrence Berkeley National Laboratory, Earth Sciences Division, 1 Cyclotron Rd., Berkeley, CA 94720, USA

[4] Jet Propulsion Laboratory, 4800 Oak Grove Drive, Pasadena, CA 91109, USA

[5] German Aerospace Center, Institute of Aerospace Medicine and Radiation Biology, Linder Höhe, 51147 Köln, Germany

[6] Leibniz Institute DSMZ - Deutsche Sammlung von Mikroorganismen und Zellkulturen GmbH, Inhoffenstraße 7 B, 38124 Braunschweig, Germany

Table S1: Colony forming units (CFUs) obtained from wipe samples (alternative cultivation with respect to oligotrophs and alkaliphiles). Sd: standard deviation. Rsd: relative standard deviation.

Oligotrophs

CO	technical replicate	CFUs	CFU/m^2	mean	sd	rsd	total mean	sd	rsd
Wipe CO_W3	CO_W3a	4							
	CO_W3b	2							
	CO_W3c	3							
	CO_W3d	6							
	CO_W3e	14	644	5.80	4.31	0.74			
Wipe CO_W4	CO_W4a	26							
	CO_W4b	47							
	CO_W4c	45							
	CO_W4d	19							
	CO_W4e	43	4000	36.00	11.31	0.31	20.90	17.36	0.83
**UR**	*technical replicate*	*CFUs*	*CFU/m^2*	*mean*	*sd*	*rsd*	*total mean*	*sd*	*rsd*
Wipe UR_W3	UR_W3a	67							
	UR_W3b	76							
	UR_W3c	81							
	UR_W3d	122							
	UR_W3e	123	10422	93.80	23.86	0.25			
Wipe UR_W4	UR_W4a	208							
	UR_W4b	214							
	UR_W4c	229							
	UR_W4d	207							
	UR_W4e	218	23911	215.20	7.98	0.04	154.50	63.25	0.41
**CR8**	*technical replicate*	*CFUs*	*CFU/m^2*	*mean*	*sd*	*rsd*	*total mean*	*sd*	*rsd*
Wipe CR8_W3	CR8_W3a	1							
	CR8_W3b	0							
	CR8_W3c	2							
	CR8_W3d	2							
	CR8_W3e	1	133	1.20	0.75	0.62			
Wipe CR8_W4	CR8_W4a	353							
	CR8_W4b	227							
	CR8_W4c	437							
	CR8_W4d	282							
	CR8_W4e	192	33133	298.20	88.18	0.30	149.70	161.06	1.08
**CR5**	*technical replicate*	*CFUs*	*CFU/m^2*	*mean*	*sd*	*rsd*	*total mean*	*sd*	*rsd*
Wipe CR5_W3	CR5_W3a	0							
	CR5_W3b	2							
	CR5_W3c	0							
	CR5_W3d	0							
	CR5_W3e	0	44	0.40	0.80	2.00			
Wipe CR5_W4	CR5_W4a	13							
	CR5_W4b	2							
	CR5_W4c	2							
	CR5_W4d	8							
	CR5_W4e	8	733	6.60	4.18	0.63	3.50	4.32	1.23

pH10

CO	technical replicate	CFUs	CFU/m^2	mean	sd	rsd	total mean	sd	rsd
Wipe CO_W3	CO_W3a	0							
	CO_W3b	0							
	CO_W3c	0							
	CO_W3d	0							
	CO_W3e	0	0	0	0				
Wipe CO_W4	CO_W4a	12							
	CO_W4b	11							
	CO_W4c	12							
	CO_W4d	11							
	CO_W4e	9	1222	11	1.095445115	0.10	5.50	5.55	1.01
**UR**	*technical replicate*	*CFUs*	*CFU/m^2*	*mean*	*sd*	*rsd*	*total mean*	*sd*	*rsd*
Wipe UR_W3	UR_W3a	24							
	UR_W3b	36							
	UR_W3c	28							
	UR_W3d	27							
	UR_W3e	21	3022	27.2	5.035871325	0.19			
Wipe UR_W4	UR_W4a	6							
	UR_W4b	9							
	UR_W4c	11							
	UR_W4d	3							
	UR_W4e	2	689	6.2	3.42928564	0.55	16.70	11.35	0.68
**CR8**	*technical replicate*	*CFUs*	*CFU/m^2*	*mean*	*sd*	*rsd*	*total mean*	*sd*	*rsd*
Wipe CR8_W3	CR8_W3a	0							
	CR8_W3b	0							
	CR8_W3c	0							
	CR8_W3d	0							
	CR8_W3e	0	0	0	0				
Wipe CR8_W4	CR8_W4a	0							
	CR8_W4b	0							
	CR8_W4c	0							
	CR8_W4d	0							
	CR8_W4e	0	0	0	0		0.00	0.00	
**CR5**	*technical replicate*	*CFUs*	*CFU/m^2*	*mean*	*sd*	*rsd*	*total mean*	*sd*	*rsd*
Wipe CR5_W3	CR5_W3a	0							
	CR5_W3b	0							
	CR5_W3c	0							
	CR5_W3d	0							
	CR5_W3e	0	0	0	0				
Wipe CR5_W4	CR5_W4a	0							
	CR5_W4b	2							
	CR5_W4c	2							
	CR5_W4d	0							
	CR5_W4e	0	89	0.8	0.979795897	1.22	0.40	0.80	2.00

Table S2: Number of isolated microorganisms under different cultivation conditions and from different samples

cultivation target	oligotrophs				alkaliphiles/alkalitolerants				(facultatively) anaerobs			
isolated species / sample	CR5	CR8	CO	UR	CR5	CR8	CO	UR	CR5	CR8	CO	UR
Acinetobacter lwoffii	0	0	0	1	2	0	0	0	0	0	0	0
Acinetobacter pittii	0	0	0	1	1	0	0	0	0	0	0	0
Aerococcus sp.	0	0	0	0	0	0	0	2	0	0	0	0
Agrococcus versicolor	0	0	0	1	0	0	0	0	0	0	0	0
Arthrobacter sp.	0	0	0	2	0	0	0	0	0	0	0	0
Bacillus cereus	0	0	0	0	0	0	0	0	0	0	1	0
Bacillus licheniformis	0	0	0	3	0	0	0	0	0	2	1	1
Bacillus sp.	0	0	0	0	0	0	0	1	0	0	0	0
Bacillus thuringiensis	0	0	1	0	0	0	0	0	0	0	0	1
Brachybacterium faecium	0	0	0	0	0	0	0	4	0	0	0	0
Brachybacterium paraconglomeratum	0	0	0	0	0	0	1	2	0	0	0	0
Cellulomonas sp.	0	1	0	0	0	1	0	0	0	0	0	0
Chryseobacterium sp.	0	0	0	2	0	0	0	0	0	0	0	0
Curtobacterium flaccumfaciens	0	0	1	0	0	0	0	0	0	0	0	0
Enterococcus mundtii	0	0	0	0	0	0	2	0	0	0	0	1
Erwinia persica	0	3	0	0	0	0	0	0	0	0	0	0
Kocuria sp.	0	0	0	1	0	0	0	0	0	0	0	0
Microbacterium oxydans	0	0	0	1	0	0	0	0	0	0	0	0
MicRobacterium sp.	0	1	0	18	0	1	0	0	0	0	0	0
Micrococcus luteus	0	0	3	1	0	1	0	0	0	0	0	0
Moraxella osloensis	0	0	0	1	0	0	0	0	0	0	0	0
Nesterenkonia lutea	0	0	0	0	0	0	0	4	0	0	0	0
Paenibacillus glucanolyticus	0	0	1	0	0	0	0	0	0	0	0	0
Paenibacillus hunanensis	0	0	0	0	0	0	0	0	0	0	1	0
Paenibacillus pasadenensis	0	0	3	0	0	0	0	0	0	0	0	0
Pantoea agglomerans	0	0	0	0	0	0	0	0	0	0	0	1
Propionibacterium avidum	0	0	0	0	0	0	0	0	0	1	0	1
Rhodococcus fascians	0	1	0	0	0	0	0	0	0	0	0	0
Rhodococcus sp.	0	0	0	2	0	0	0	0	0	0	0	0
Salinicoccus qingdaonensis	0	0	0	0	0	0	0	2	0	0	0	0
Staphylococcus aureus	0	0	0	1	0	0	0	1	0	1	1	0
Staphylococcus capitis	0	0	0	1	0	0	0	0	3	3	1	0
Staphylococcus caprae	1	5	0	0	0	0	0	0	0	1	1	0
Staphylococcus epidermidis	0	0	0	0	0	0	0	0	0	0	0	4
Staphylococcus haemolyticus	0	0	2	0	0	0	0	0	0	0	0	0
Staphylococcus hominis	0	0	0	0	0	0	0	0	0	6	5	1
Staphylococcus lugdunensis	21	0	0	0	3	0	0	0	0	0	0	0
Staphylococcus pettenkoferi	0	0	0	0	0	0	0	0	2	0	0	0
Staphylococcus saphrophyticus	0	6	0	0	0	1	0	1	0	0	0	1
Staphylococcus sp.	0	0	0	0	0	0	0	0	0	1	0	0
Staphylococcus warneri	0	0	0	0	0	0	0	0	0	0	0	1

Table S3: Number of clones processed from each sample, number of singular OTUs and coverage.									
**sample**	**CR 5**	**CR 8**	**CO**	**UR**	**CR 5-PMA**	**CR 8-PMA**	**CO-PMA**	**UR-PMA**	**EB**
**No. of clones picked**	144	168	96	96	48	48	48	48	96
**Chimera**	9	12	15	16	0	0	0	0	0
**No. of clones processed after quality filtering**	80	71	52	51	43	47	44	45	86
**No. of singular OTUs**	25	30	27	22	1	1	5	8	4
**Coverage**	0.69	0.58	0.48	0.57	0.98	0.98	0.87	0.82	0.95

Table S4 (part A): Accession numbers, abundance and taxonomic affiliation of detected bacterial 16S rRNA genes by classical gene cloning of samples from different locations, with or without PMA treatment

	samples				samples, PMA treated				taxonomic classification of bacterial 16S rRNA genes				
Acc. No.	UR	CO	CR8	CR5	UR_PMA	CO_PMA	CR8_PMA	CR5_PMA	phylum	class	order	family	genus
JQ855629	0	1	0	0	0	0	0	0	Actinobacteria	Actinobacteria	Acidimicrobiales	CL500-29	unclassified
JQ855568	2	0	0	0	0	0	0	0	Actinobacteria	Actinobacteria	Actinomycetales	Brevibacteriaceae	Brevibacterium
JQ855550	0	0	1	0	0	0	0	0	Actinobacteria	Actinobacteria	Actinomycetales	Cellulomonadaceae	Actinotalea
JQ855601	0	1	0	0	0	0	0	0	Actinobacteria	Actinobacteria	Actinomycetales	Cellulomonadaceae	Demequina
JQ855613	0	2	0	1	0	0	0	0	Actinobacteria	Actinobacteria	Actinomycetales	Cellulomonadaceae	unclassified
JQ855529	0	1	0	4	0	0	0	0	Actinobacteria	Actinobacteria	Actinomycetales	Corynebacteriaceae	Corynebacterium
JQ855530	0	0	0	2	0	0	0	0	Actinobacteria	Actinobacteria	Actinomycetales	Corynebacteriaceae	Corynebacterium
JQ855541	1	1	0	0	0	0	0	0	Actinobacteria	Actinobacteria	Actinomycetales	Corynebacteriaceae	Corynebacterium
JQ855545	0	3	0	1	0	0	0	0	Actinobacteria	Actinobacteria	Actinomycetales	Corynebacteriaceae	Corynebacterium
JQ855561	0	0	2	10	0	0	0	0	Actinobacteria	Actinobacteria	Actinomycetales	Corynebacteriaceae	Corynebacterium
JQ855563	0	0	1	0	0	0	0	1	Actinobacteria	Actinobacteria	Actinomycetales	Corynebacteriaceae	Corynebacterium
JQ855564	0	0	0	1	0	0	0	0	Actinobacteria	Actinobacteria	Actinomycetales	Corynebacteriaceae	Corynebacterium
JQ855592	0	1	0	0	0	0	0	0	Actinobacteria	Actinobacteria	Actinomycetales	Corynebacteriaceae	Corynebacterium
JQ855600	0	0	0	6	1	0	0	0	Actinobacteria	Actinobacteria	Actinomycetales	Corynebacteriaceae	Corynebacterium
JQ855608	0	0	0	1	0	0	0	0	Actinobacteria	Actinobacteria	Actinomycetales	Corynebacteriaceae	Corynebacterium
JQ855609	0	0	0	1	0	0	0	0	Actinobacteria	Actinobacteria	Actinomycetales	Corynebacteriaceae	Corynebacterium
JQ855614	0	1	0	0	0	0	0	0	Actinobacteria	Actinobacteria	Actinomycetales	Corynebacteriaceae	Corynebacterium
JQ855615	0	0	1	0	0	0	0	0	Actinobacteria	Actinobacteria	Actinomycetales	Corynebacteriaceae	Corynebacterium
JQ855619	0	0	1	0	0	0	0	0	Actinobacteria	Actinobacteria	Actinomycetales	Corynebacteriaceae	Corynebacterium
JQ855620	0	1	0	2	0	0	0	0	Actinobacteria	Actinobacteria	Actinomycetales	Corynebacteriaceae	Corynebacterium
JQ855622	0	0	0	10	0	0	0	0	Actinobacteria	Actinobacteria	Actinomycetales	Corynebacteriaceae	Corynebacterium
JQ855630	0	0	0	1	0	0	0	0	Actinobacteria	Actinobacteria	Actinomycetales	Corynebacteriaceae	Corynebacterium
JQ855560	1	0	0	0	0	0	0	0	Actinobacteria	Actinobacteria	Actinomycetales	Dermabacteraceae	Brachybacterium
JQ855531	0	0	1	4	0	0	0	0	Actinobacteria	Actinobacteria	Actinomycetales	Dermabacteraceae	Dermabacter
JQ855548	0	2	0	0	0	0	0	0	Actinobacteria	Actinobacteria	Actinomycetales	Intrasporangiaceae	unclassified
JQ855599	0	1	0	0	0	0	0	0	Actinobacteria	Actinobacteria	Actinomycetales	Intrasporangiaceae	unclassified
JQ855611	0	1	0	0	0	0	0	0	Actinobacteria	Actinobacteria	Actinomycetales	Intrasporangiaceae	unclassified
JQ855596	0	0	0	1	0	0	0	0	Actinobacteria	Actinobacteria	Actinomycetales	Kineosporiaceae	unclassified
JQ855570	0	0	1	0	0	0	0	0	Actinobacteria	Actinobacteria	Actinomycetales	Microbacteriaceae	Agreia
JQ855583	0	0	0	0	1	0	0	0	Actinobacteria	Actinobacteria	Actinomycetales	Microbacteriaceae	Microbacterium
JQ855626	0	0	0	1	0	0	0	0	Actinobacteria	Actinobacteria	Actinomycetales	Microbacteriaceae	Microbacterium
JQ855593	0	0	1	0	0	0	0	0	Actinobacteria	Actinobacteria	Actinomycetales	Microbacteriaceae	Rathayibacter
JQ855576	1	0	0	0	0	0	0	0	Actinobacteria	Actinobacteria	Actinomycetales	Micrococcaceae	Kocuria
JQ855632	1	0	0	1	0	0	0	0	Actinobacteria	Actinobacteria	Actinomycetales	Micrococcaceae	Kocuria
JQ855612	0	0	1	0	0	0	0	0	Actinobacteria	Actinobacteria	Actinomycetales	Micrococcaceae	Micrococcus
JQ855635	3	0	2	1	0	0	0	0	Actinobacteria	Actinobacteria	Actinomycetales	Micrococcaceae	Micrococcus
JQ855509	0	1	1	0	0	0	0	0	Actinobacteria	Actinobacteria	Actinomycetales	Nocardioidaceae	Friedmanniella
JQ855597	0	0	0	1	0	0	0	0	Actinobacteria	Actinobacteria	Actinomycetales	Nocardioidaceae	Friedmanniella
JQ855617	0	1	0	0	0	0	0	0	Actinobacteria	Actinobacteria	Actinomycetales	Nocardioidaceae	Friedmanniella
JQ855551	1	0	0	0	0	0	0	0	Actinobacteria	Actinobacteria	Actinomycetales	Nocardioidaceae	Marmoricola
JQ855536	0	0	1	0	0	0	0	0	Actinobacteria	Actinobacteria	Actinomycetales	Nocardioidaceae	Nocardioides
JQ855565	0	0	1	0	0	0	0	0	Actinobacteria	Actinobacteria	Actinomycetales	Nocardioidaceae	Nocardioides
JQ855587	0	0	0	0	0	1	0	0	Actinobacteria	Actinobacteria	Actinomycetales	Nocardioidaceae	Propionicimonas
JQ855532	0	0	1	0	0	0	0	0	Actinobacteria	Actinobacteria	Actinomycetales	Nocardioidaceae	unclassified
JQ855578	0	1	0	0	0	0	0	0	Actinobacteria	Actinobacteria	Actinomycetales	Nocardioidaceae	unclassified
JQ855633	0	0	0	1	0	0	0	0	Actinobacteria	Actinobacteria	Actinomycetales	Nocardioidaceae	unclassified
JQ855579	0	0	1	0	0	0	0	0	Actinobacteria	Actinobacteria	Actinomycetales	Promicromonosporaceae	Cellulosimicrobium
JQ855605	0	3	0	0	0	0	0	0	Actinobacteria	Actinobacteria	Actinomycetales	Propionibacteriaceae	Microlunatus
JQ855520	0	0	2	0	0	0	0	0	Actinobacteria	Actinobacteria	Actinomycetales	Propionibacteriaceae	Propionibacterium
JQ855543	5	2	12	4	2	0	0	0	Actinobacteria	Actinobacteria	Actinomycetales	Propionibacteriaceae	Propionibacterium
JQ855627	0	0	1	0	0	0	0	0	Actinobacteria	Actinobacteria	Actinomycetales	Sanguibacteraceae	Sanguibacter
JQ855526	0	1	2	1	0	0	0	0	Actinobacteria	Actinobacteria	Actinomycetales	unclassified	unclassified
JQ855590	1	0	0	0	0	0	0	0	Actinobacteria	Actinobacteria	Actinomycetales	unclassified	unclassified
JQ855598	0	0	0	1	0	0	0	0	Actinobacteria	Actinobacteria	Actinomycetales	unclassified	unclassified
JQ855604	0	0	0	1	0	0	0	0	Actinobacteria	Actinobacteria	Actinomycetales	unclassified	unclassified
JQ855628	0	1	0	0	0	0	0	0	Actinobacteria	Actinobacteria	Actinomycetales	unclassified	unclassified
JQ855562	1	0	0	0	0	0	0	0	Bacteroidetes	Flavobacteria	Flavobacteriales	Cryomorphaceae	unclassified
JQ855621	1	0	0	0	0	0	0	0	Bacteroidetes	Flavobacteria	Flavobacteriales	Flavobacteriaceae	Chryseobacterium
JQ855623	0	1	1	0	0	0	0	0	Bacteroidetes	Flavobacteria	Flavobacteriales	Flavobacteriaceae	Chryseobacterium
JQ855555	0	1	0	2	0	0	0	0	Bacteroidetes	Flavobacteria	Flavobacteriales	Flavobacteriaceae	Flavobacterium
JQ855616	0	0	2	0	0	0	0	0	Bacteroidetes	Flavobacteria	Flavobacteriales	Flavobacteriaceae	Flavobacterium
JQ855534	0	0	1	1	0	1	0	0	Bacteroidetes	Sphingobacteria	Sphingobacteriale	Chitinophagaceae	unclassified
JQ855571	0	2	1	0	0	0	0	0	Cyanobacteria	Oscillatoriophycidea	Chroococcales	Xenococcaceae	Chroococcidiopsis
JQ855573	0	2	2	0	0	0	0	0	Cyanobacteria	Oscillatoriophycidea	Chroococcales	Xenococcaceae	Chroococcidiopsis
JQ855580	0	0	1	0	1	0	0	0	Cyanobacteria	Oscillatoriophycidea	Chroococcales	Xenococcaceae	Chroococcidiopsis
JQ855618	0	0	0	1	0	0	0	0	Cyanobacteria	Oscillatoriophycidea	Chroococcales	Xenococcaceae	Chroococcidiopsis

Table S4 (part B): Accession numbers, abundance and taxonomic affiliation of detected bacterial 16S rRNA genes by classical gene cloning of samples from different locations, with or without PMA treatment

	samples				samples, PMA treated				taxonomic classification of bacterial 16S rRNA genes				
Acc. No.	UR	CO	CR8	CR5	UR_PMA	CO_PMA	CR8_PMA	CR5_PMA	phylum	class	order	family	genus
JQ855558	0	2	0	0	0	5	0	0	Firmicutes	Bacilli	Bacillales	Bacillaceae	Bacillus
JQ855594	0	3	0	0	0	0	0	0	Firmicutes	Bacilli	Bacillales	Bacillaceae	Bacillus
JQ855634	0	1	0	0	0	0	0	0	Firmicutes	Bacilli	Bacillales	Planococcaceae	Rummeliibacillus
JQ855535	2	0	0	0	0	0	0	0	Firmicutes	Bacilli	Bacillales	Staphylococcaceae	Jeotgalicoccus
JQ855539	1	0	1	0	0	0	0	0	Firmicutes	Bacilli	Bacillales	Staphylococcaceae	Jeotgalicoccus
JQ855510	2	1	11	4	1	0	1	0	Firmicutes	Bacilli	Bacillales	Staphylococcaceae	Staphylococcus
JQ855511	1	0	0	0	0	0	0	0	Firmicutes	Bacilli	Bacillales	Staphylococcaceae	Staphylococcus
JQ855518	0	0	0	1	0	0	0	0	Firmicutes	Bacilli	Bacillales	Staphylococcaceae	Staphylococcus
JQ855553	1	0	0	0	0	0	0	0	Firmicutes	Bacilli	Bacillales	Staphylococcaceae	Staphylococcus
JQ855567	1	0	0	0	0	0	0	0	Firmicutes	Bacilli	Bacillales	Staphylococcaceae	Staphylococcus
JQ855610	0	0	1	0	0	0	0	0	Firmicutes	Bacilli	Bacillales	Staphylococcaceae	Staphylococcus
JQ855606	0	0	0	3	0	0	0	0	Firmicutes	Bacilli	Lactobacillales	Aerococcaceae	unclassified
JQ855544	1	0	0	0	0	0	0	0	Firmicutes	Bacilli	Lactobacillales	Lactobacillaceae	Lactobacillus
JQ855521	0	0	1	0	0	0	0	0	Firmicutes	Bacilli	Lactobacillales	Streptococcaceae	Streptococcus
JQ855522	1	0	0	2	1	0	0	0	Firmicutes	Bacilli	Lactobacillales	Streptococcaceae	Streptococcus
JQ855528	0	0	0	1	0	0	0	0	Firmicutes	Bacilli	Lactobacillales	Streptococcaceae	Streptococcus
JQ855572	0	0	1	0	0	0	0	0	Firmicutes	Bacilli	Lactobacillales	Streptococcaceae	Streptococcus
JQ855595	0	0	0	0	0	2	0	0	Firmicutes	Clostridia	Clostridiales	Clostridiaceae	Clostridium
JQ855538	0	2	0	2	0	0	0	0	Firmicutes	Clostridia	Clostridiales	Clostridiales_Family_XI._Incertae_Se	Anaerococcus
JQ855631	0	0	2	0	0	0	0	0	Firmicutes	Clostridia	Clostridiales	Clostridiales_Family_XI._Incertae_Se	Finegoldia
JQ855525	0	0	2	0	0	0	0	0	Firmicutes	Clostridia	Clostridiales	Clostridiales_Family_XI._Incertae_Se	Peptoniphilus
JQ855527	0	0	0	0	0	1	0	0	Firmicutes	Clostridia	Clostridiales	Clostridiales_Family_XI._Incertae_Se	Tissierella
JQ855537	0	1	0	0	0	0	0	0	Firmicutes	Clostridia	Clostridiales	Clostridiales_Family_XI._Incertae_Se	Tissierella
JQ855584	0	0	0	0	1	0	0	0	Proteobacteria	Alphaproteobacteria	Caulobacterales	Caulobacteraceae	Brevundimonas
JQ855588	0	1	0	0	0	0	0	0	Proteobacteria	Alphaproteobacteria	Rhizobiales	Phyllobacteriaceae	unclassified
JQ855566	0	1	0	1	0	0	0	0	Proteobacteria	Alphaproteobacteria	Rhizobiales	Rhizobiaceae	Agrobacterium
JQ855585	0	1	0	0	0	0	0	0	Proteobacteria	Alphaproteobacteria	Rhodobacterales	Rhodobacteraceae	Amaricoccus
JQ855513	0	0	1	0	0	0	0	0	Proteobacteria	Alphaproteobacteria	Rhodobacterales	Rhodobacteraceae	Paracoccus
JQ855559	0	1	0	0	0	1	0	0	Proteobacteria	Alphaproteobacteria	Rhodobacterales	Rhodobacteraceae	Paracoccus
JQ855554	0	1	0	0	0	0	0	0	Proteobacteria	Alphaproteobacteria	Rhodospirillales	Acetobacteraceae	unclassified
JQ855625	0	2	0	0	0	0	0	0	Proteobacteria	Alphaproteobacteria	Sphingomonadale	Sphingomonadaceae	Sphingobium
JQ855517	0	0	1	0	0	0	0	0	Proteobacteria	Alphaproteobacteria	Sphingomonadale	Sphingomonadaceae	Sphingomonas
JQ855577	0	0	0	1	0	0	0	0	Proteobacteria	Alphaproteobacteria	Sphingomonadale	Sphingomonadaceae	Sphingomonas
JQ855586	0	1	0	0	0	0	0	0	Proteobacteria	Alphaproteobacteria	Sphingomonadale	Sphingomonadaceae	Sphingomonas
JQ855542	0	0	0	0	1	0	0	0	Proteobacteria	Betaproteobacteria	Burkholderiales	Burkholderiaceae	Ralstonia
JQ855582	1	0	0	0	0	0	0	0	Proteobacteria	Betaproteobacteria	Burkholderiales	Comamonadaceae	Acidovorax
JQ855552	1	0	0	0	0	0	0	0	Proteobacteria	Betaproteobacteria	Burkholderiales	Comamonadaceae	Variovorax
JQ855512	1	0	0	1	0	0	0	0	Proteobacteria	Betaproteobacteria	Burkholderiales	Oxalobacteraceae	Massilia
JQ855575	6	0	0	0	0	0	0	0	Proteobacteria	Betaproteobacteria	Burkholderiales	Oxalobacteraceae	Massilia
JQ855581	1	0	0	0	0	0	0	0	Proteobacteria	Betaproteobacteria	Burkholderiales	Oxalobacteraceae	Massilia
JQ855624	0	0	2	0	0	0	0	0	Proteobacteria	Betaproteobacteria	Burkholderiales	Oxalobacteraceae	Massilia
JQ855540	1	0	1	0	0	1	0	0	Proteobacteria	Deltaproteobacteria	Myxococcales	unclassified	unclassified
JQ855549	2	0	0	0	0	0	0	0	Proteobacteria	Gammaproteobacter	Legionellales	Coxiellaceae	Rickettsiella
JQ855556	7	0	0	0	9	0	0	0	Proteobacteria	Gammaproteobacter	Legionellales	Coxiellaceae	Rickettsiella
JQ855569	1	0	0	0	0	0	0	0	Proteobacteria	Gammaproteobacter	Legionellales	Coxiellaceae	Rickettsiella
JQ855591	0	0	0	0	1	0	0	0	Proteobacteria	Gammaproteobacter	Legionellales	Coxiellaceae	Rickettsiella
JQ855547	1	0	0	0	0	0	0	0	Proteobacteria	Gammaproteobacter	Pseudomonadales	Moraxellaceae	Acinetobacter
JQ855603	1	0	0	0	0	0	0	0	Proteobacteria	Gammaproteobacter	Pseudomonadales	Moraxellaceae	Acinetobacter
JQ855546	0	1	0	0	0	0	0	0	Proteobacteria	Gammaproteobacter	Xanthomonadales	Xanthomonadaceae	Lysobacter
JQ855589	0	0	1	0	0	0	0	0	Proteobacteria	Gammaproteobacter	Xanthomonadales	Xanthomonadaceae	Pseudoxanthomonas
JQ855533	0	0	1	0	0	0	0	0	SC4	unclassified	unclassified	unclassified	unclassified
JQ855514	0	0	0	1	0	0	0	0	Thermi	Deinococci	Deinococcales	Deinococcaceae	Deinococcus
JQ855607	0	0	1	0	0	0	0	0	Verrucomicrobi	Verrucomicrobiae	Verrucomicrobiale	Verrucomicrobiaceae	Luteolibacter
	51	51	69	78	19	12	1	1					

Table S5 (part A): OTUs, no. of reads and corresponding taxonomic affiliation of detected bacterial 16S rRNA amplicons by 454 pyrotag sequencing. Please note, the data shown here was either rank normalized or sum normlaized (to 5000 reads per sample) before performing statisical analysis as depicted in the manuscript.

	Samples and no. of reads				taxonomic classification of bacterial 16S rRNA gene amplicons				
OTU	UR	CO	CR8	CR5	phylum	class	order	family	genus
50	0	1	0	2	Acidobacteria	Acidobacteria	Acidobacteriales	Acidobacteriaceae	unclassified
176	0	0	2	0	Acidobacteria	Solibacteres	Solibacterales	Solibacteraceae	Candidatus_Solibacter
204	2	0	0	0	Acidobacteria	Chloracidobacteria	unclassified	unclassified	unclassified
258	0	0	1	0	Acidobacteria	Acidobacteria	Acidobacteriales	Acidobacteriaceae	unclassified
303	0	1	0	0	Acidobacteria	Solibacteres	Solibacterales	Solibacteraceae	Candidatus_Solibacter
341	1	1	0	2	Acidobacteria	Chloracidobacteria	unclassified	unclassified	unclassified
367	0	0	1	0	Acidobacteria	Chloracidobacteria	unclassified	unclassified	unclassified
390	0	1	1	0	Acidobacteria	Chloracidobacteria	unclassified	unclassified	unclassified
397	1	0	0	0	Acidobacteria	Sva0725	Sva0725	unclassified	unclassified
409	0	0	3	0	Acidobacteria	Solibacteres	Solibacterales	Solibacteraceae	Candidatus_Solibacter
419	0	1	0	0	Acidobacteria	Chloracidobacteria	unclassified	unclassified	unclassified
510	0	0	1	0	Acidobacteria	Solibacteres	Solibacterales	Solibacteraceae	Candidatus_Solibacter
520	0	0	6	0	Acidobacteria	iii1-8	DS-18	unclassified	unclassified
541	0	1	0	1	Acidobacteria	Acidobacteria	Acidobacteriales	Acidobacteriaceae	unclassified
581	0	1	0	1	Acidobacteria	Chloracidobacteria	unclassified	unclassified	unclassified
617	0	2	0	0	Acidobacteria	Solibacteres	Solibacterales	Solibacteraceae	Candidatus_Solibacter
620	0	0	1	0	Acidobacteria	Solibacteres	Solibacterales	Solibacteraceae	Candidatus_Solibacter
645	0	1	2	0	Acidobacteria	Solibacteres	Solibacterales	Solibacteraceae	Candidatus_Solibacter
706	0	0	0	1	Acidobacteria	Chloracidobacteria	unclassified	unclassified	unclassified
725	0	2	0	0	Acidobacteria	Chloracidobacteria	unclassified	unclassified	unclassified
731	1	0	0	0	Acidobacteria	Chloracidobacteria	unclassified	unclassified	unclassified
732	1	2	0	0	Acidobacteria	Chloracidobacteria	unclassified	unclassified	unclassified
735	0	1	0	0	Acidobacteria	Chloracidobacteria	unclassified	unclassified	unclassified
771	1	1	0	0	Acidobacteria	Chloracidobacteria	unclassified	unclassified	unclassified
785	0	1	0	0	Acidobacteria	Chloracidobacteria	unclassified	unclassified	unclassified
815	0	1	0	0	Acidobacteria	Solibacteres	Solibacterales	Solibacteraceae	Candidatus_Solibacter
836	2	1	0	0	Acidobacteria	Solibacteres	Solibacterales	Solibacteraceae	Candidatus_Solibacter
857	0	1	0	1	Acidobacteria	Solibacteres	Solibacterales	Solibacteraceae	Candidatus_Solibacter
973	0	0	0	3	Acidobacteria	Solibacteres	Solibacterales	Solibacteraceae	Candidatus_Solibacter
1050	0	0	0	1	Acidobacteria	Chloracidobacteria	unclassified	unclassified	unclassified
1089	1	0	0	0	Acidobacteria	Chloracidobacteria	unclassified	unclassified	unclassified
1137	0	0	1	1	Acidobacteria	Chloracidobacteria	unclassified	unclassified	unclassified
1175	0	1	1	1	Acidobacteria	Acidobacteria	Acidobacteriales	Acidobacteriaceae	unclassified
1196	0	0	0	1	Acidobacteria	Solibacteres	Solibacterales	Solibacteraceae	Candidatus_Solibacter
1215	0	1	0	0	Acidobacteria	Solibacteres	Solibacterales	Solibacteraceae	Candidatus_Solibacter
1223	0	0	0	1	Acidobacteria	Acidobacteria	Acidobacteriales	Acidobacteriaceae	unclassified
1323	0	1	0	0	Acidobacteria	Chloracidobacteria	unclassified	unclassified	unclassified
1349	1	1	0	0	Acidobacteria	Chloracidobacteria	unclassified	unclassified	unclassified
1376	1	2	1	0	Acidobacteria	Chloracidobacteria	unclassified	unclassified	unclassified
1388	0	1	0	0	Acidobacteria	Solibacteres	Solibacterales	Solibacteraceae	Candidatus_Solibacter
1435	0	1	0	0	Acidobacteria	Solibacteres	Solibacterales	Solibacteraceae	Candidatus_Solibacter
1487	0	2	0	0	Acidobacteria	Acidobacteria	Acidobacteriales	Acidobacteriaceae	unclassified
1507	0	1	0	0	Acidobacteria	Acidobacteria	Acidobacteriales	Acidobacteriaceae	Terriglobus
1537	0	1	0	0	Acidobacteria	Chloracidobacteria	unclassified	unclassified	unclassified
1559	0	0	3	1	Acidobacteria	Acidobacteria	Acidobacteriales	Acidobacteriaceae	unclassified
1570	0	1	0	1	Acidobacteria	iii1-8	32-20	unclassified	unclassified
1573	0	1	0	0	Acidobacteria	Solibacteres	Solibacterales	Solibacteraceae	Candidatus_Solibacter
1592	0	0	0	1	Acidobacteria	iii1-8	32-20	unclassified	unclassified
1628	0	1	0	0	Acidobacteria	Solibacteres	Solibacterales	Solibacteraceae	Candidatus_Solibacter
1644	0	1	0	0	Acidobacteria	Chloracidobacteria	unclassified	unclassified	unclassified
10	0	1	1	0	Actinobacteria	Actinobacteria	Acidimicrobiales	CL500-29	unclassified
21	0	1	0	0	Actinobacteria	Actinobacteria	Acidimicrobiales	CL500-29	unclassified
31	0	0	1	0	Actinobacteria	Actinobacteria	0319-7L14	unclassified	unclassified
37	0	1	4	8	Actinobacteria	Actinobacteria	Actinomycetales	Propionibacteriaceae	Propionibacterium
38	0	3	0	0	Actinobacteria	Actinobacteria	Actinomycetales	Propionibacteriaceae	Tessaracoccus
42	0	0	1	0	Actinobacteria	Actinobacteria	Acidimicrobiales	Iamiaceae	Iamia
59	0	37	0	0	Actinobacteria	Actinobacteria	Actinomycetales	Intrasporangiaceae	unclassified
63	0	2	1	0	Actinobacteria	Actinobacteria	unclassified	unclassified	unclassified
66	0	0	0	1	Actinobacteria	Actinobacteria	Acidimicrobiales	CL500-29	unclassified
81	0	2	0	0	Actinobacteria	Actinobacteria	Acidimicrobiales	Iamiaceae	Iamia
82	0	1	0	0	Actinobacteria	Actinobacteria	Acidimicrobiales	CL500-29	unclassified
86	0	0	2	5	Actinobacteria	Actinobacteria	Actinomycetales	Corynebacteriaceae	Corynebacterium
88	1	3	5	5	Actinobacteria	Actinobacteria	Actinomycetales	Intrasporangiaceae	Janibacter
90	0	0	1	0	Actinobacteria	Actinobacteria	Solirubrobacterales	Conexibacteraceae	unclassified
95	0	1	1	0	Actinobacteria	Actinobacteria	Actinomycetales	Microbacteriaceae	Microbacterium
99	0	1	3	2	Actinobacteria	Actinobacteria	MC47	unclassified	unclassified
100	3	10	9	3	Actinobacteria	Actinobacteria	Actinomycetales	Microbacteriaceae	Microbacterium
146	0	2	0	3	Actinobacteria	Actinobacteria	Actinomycetales	Micrococcaceae	unclassified
152	0	1	1	1	Actinobacteria	Actinobacteria	Actinomycetales	Propionibacteriaceae	Tessaracoccus
163	2	0	1	2	Actinobacteria	Actinobacteria	Actinomycetales	Corynebacteriaceae	Corynebacterium
171	0	1	0	0	Actinobacteria	Actinobacteria	Acidimicrobiales	Acidimicrobiaceae	unclassified
177	0	1	2	0	Actinobacteria	Actinobacteria	Actinomycetales	Mycobacteriaceae	Mycobacterium
192	0	1	0	0	Actinobacteria	Actinobacteria	Actinomycetales	Microbacteriaceae	unclassified
205	0	0	0	3	Actinobacteria	Actinobacteria	Actinomycetales	Nocardioidaceae	unclassified
225	0	1	0	0	Actinobacteria	Actinobacteria	Actinomycetales	Nocardioidaceae	Nocardioides
232	0	5	2	3	Actinobacteria	Actinobacteria	Actinomycetales	Microbacteriaceae	unclassified
237	0	0	1	0	Actinobacteria	Actinobacteria	Actinomycetales	Propionibacteriaceae	Propionibacterium
249	0	2	0	0	Actinobacteria	Actinobacteria	Acidimicrobiales	CL500-29	unclassified
251	2	0	0	0	Actinobacteria	Actinobacteria	unclassified	unclassified	unclassified
257	0	0	1	0	Actinobacteria	Actinobacteria	Actinomycetales	Nocardioidaceae	unclassified
259	0	1	2	0	Actinobacteria	Actinobacteria	Actinomycetales	Frankiaceae	unclassified
261	0	1	1	0	Actinobacteria	Actinobacteria	Actinomycetales	Nocardioidaceae	Propionicimonas
264	0	1	0	0	Actinobacteria	Actinobacteria	Solirubrobacterales	Conexibacteraceae	unclassified
280	0	0	0	4	Actinobacteria	Actinobacteria	Coriobacteriales	Coriobacteriaceae	unclassified
284	0	1	0	0	Actinobacteria	Actinobacteria	Actinomycetales	Propionibacteriaceae	Luteococcus
294	0	0	1	0	Actinobacteria	Actinobacteria	MC47	unclassified	unclassified
302	0	3	0	0	Actinobacteria	Actinobacteria	Actinomycetales	Cellulomonadaceae	unclassified
312	0	1	0	0	Actinobacteria	Actinobacteria	Solirubrobacterales	Patulibacteraceae	unclassified
316	0	5	0	0	Actinobacteria	Actinobacteria	Actinomycetales	Propionibacteriaceae	Tessaracoccus
321	0	2	0	0	Actinobacteria	Actinobacteria	Actinomycetales	Cellulomonadaceae	Demequina
328	1	0	0	0	Actinobacteria	Actinobacteria	Actinomycetales	Kineosporiaceae	unclassified
345	0	0	1	0	Actinobacteria	Actinobacteria	Solirubrobacterales	Conexibacteraceae	unclassified
346	0	1	0	0	Actinobacteria	Actinobacteria	Actinomycetales	Intrasporangiaceae	unclassified
365	0	1	1	0	Actinobacteria	Actinobacteria	Actinomycetales	Corynebacteriaceae	Corynebacterium
371	2	0	2	2	Actinobacteria	Actinobacteria	Coriobacteriales	Coriobacteriaceae	Collinsella
382	0	1	0	0	Actinobacteria	Actinobacteria	MC47	unclassified	unclassified
398	1	2	1	0	Actinobacteria	Actinobacteria	Actinomycetales	unclassified	unclassified
404	0	3	0	0	Actinobacteria	Actinobacteria	Actinomycetales	Nocardioidaceae	Propionicimonas
407	0	5	0	0	Actinobacteria	Actinobacteria	Acidimicrobiales	unclassified	unclassified

Table S5 (part B): OTUs, no. of reads and corresponding taxonomic affiliation of detected bacterial 16S rRNA amplicons by 454 pyrotag sequencing. Please note, the data shown here was either rank normalized or sum normlaized (to 5000 reads per sample) before performing statisical analysis as depicted in the manuscript.

	Samples and no. of reads				taxonomic classification of bacterial 16S rRNA gene amplicons				
OTU	UR	CO	CR8	CR5	phylum	class	order	family	genus
408	0	13	0	0	Actinobacteria	Actinobacteria	Actinomycetales	Microbacteriaceae	Leucobacter
410	0	1	3	1	Actinobacteria	Actinobacteria	Actinomycetales	Nocardioidaceae	Propionicimonas
412	0	1	1	0	Actinobacteria	Actinobacteria	Actinomycetales	Dermacoccaceae	Dermacoccus
418	0	0	3	0	Actinobacteria	Actinobacteria	Actinomycetales	Actinomycetaceae	Actinomyces
441	3	2	1	5	Actinobacteria	Actinobacteria	Actinomycetales	Corynebacteriaceae	Corynebacterium
450	1	6	1	0	Actinobacteria	Actinobacteria	Actinomycetales	Microbacteriaceae	unclassified
452	0	5	1	0	Actinobacteria	Actinobacteria	Actinomycetales	unclassified	unclassified
462	0	0	0	1	Actinobacteria	Actinobacteria	MC47	unclassified	unclassified
465	0	2	0	0	Actinobacteria	Actinobacteria	Actinomycetales	Nocardioidaceae	Propionicimonas
470	0	0	0	3	Actinobacteria	Actinobacteria	Actinomycetales	Gordoniaceae	Gordonia
472	0	1	1	1	Actinobacteria	Actinobacteria	Solirubrobacterales	Conexibacteraceae	unclassified
478	1	2	2	1	Actinobacteria	Actinobacteria	Solirubrobacterales	Conexibacteraceae	unclassified
485	2	0	0	0	Actinobacteria	Actinobacteria	Solirubrobacterales	Patulibacteraceae	unclassified
493	0	3	2	2	Actinobacteria	Actinobacteria	Actinomycetales	Microbacteriaceae	Agrococcus
499	0	0	2	0	Actinobacteria	Actinobacteria	Actinomycetales	Propionibacteriaceae	unclassified
529	0	1	0	0	Actinobacteria	Actinobacteria	Acidimicrobiales	AKIW874	unclassified
533	0	1	0	0	Actinobacteria	Actinobacteria	Actinomycetales	Cellulomonadaceae	Actinotalea
537	0	0	1	1	Actinobacteria	Actinobacteria	Acidimicrobiales	unclassified	unclassified
557	0	1	0	0	Actinobacteria	Actinobacteria	Actinomycetales	Microbacteriaceae	Yonghaparkia
559	0	0	0	2	Actinobacteria	Actinobacteria	Actinomycetales	unclassified	unclassified
563	0	5	0	0	Actinobacteria	Actinobacteria	Acidimicrobiales	Microthrixaceae	unclassified
566	0	28	0	0	Actinobacteria	Actinobacteria	Acidimicrobiales	Iamiaceae	unclassified
575	0	1	0	2	Actinobacteria	Actinobacteria	Actinomycetales	Pseudonocardiaceae	Pseudonocardia
587	1	0	0	0	Actinobacteria	Actinobacteria	Acidimicrobiales	unclassified	unclassified
596	0	0	4	0	Actinobacteria	Actinobacteria	Actinomycetales	Corynebacteriaceae	Corynebacterium
612	0	2	0	0	Actinobacteria	Actinobacteria	Actinomycetales	Actinomycetaceae	Actinomyces
624	2	0	0	1	Actinobacteria	Actinobacteria	Actinomycetales	Actinomycetaceae	Actinomyces
632	0	0	1	1	Actinobacteria	Actinobacteria	Actinomycetales	unclassified	unclassified
638	0	0	1	0	Actinobacteria	Actinobacteria	Actinomycetales	unclassified	unclassified
660	0	1	0	0	Actinobacteria	Actinobacteria	Acidimicrobiales	Acidimicrobiaceae	unclassified
661	0	16	1	0	Actinobacteria	Actinobacteria	Actinomycetales	Propionibacteriaceae	unclassified
678	0	4	1	0	Actinobacteria	Actinobacteria	Solirubrobacterales	Conexibacteraceae	unclassified
680	0	1	1	1	Actinobacteria	Actinobacteria	Actinomycetales	unclassified	unclassified
686	0	1	0	1	Actinobacteria	Actinobacteria	Actinomycetales	Nocardioidaceae	unclassified
694	0	1	0	0	Actinobacteria	Actinobacteria	Actinomycetales	unclassified	unclassified
696	0	1	1	0	Actinobacteria	Actinobacteria	Acidimicrobiales	CL500-29	unclassified
734	0	1	0	0	Actinobacteria	Actinobacteria	Actinomycetales	Geodermatophilaceae	Modestobacter
752	1	0	6	5	Actinobacteria	Actinobacteria	Actinomycetales	Brevibacteriaceae	Brevibacterium
754	0	0	1	1	Actinobacteria	Actinobacteria	Actinomycetales	Geodermatophilaceae	Geodermatophilus
766	1	1	1	0	Actinobacteria	Actinobacteria	Actinomycetales	Propionibacteriaceae	unclassified
767	0	0	2	0	Actinobacteria	Actinobacteria	Solirubrobacterales	Patulibacteraceae	unclassified
775	0	1	0	0	Actinobacteria	Actinobacteria	Actinomycetales	Microbacteriaceae	Agrococcus
781	6	3	0	1	Actinobacteria	Actinobacteria	MC47	unclassified	unclassified
813	0	1	0	0	Actinobacteria	Actinobacteria	Actinomycetales	unclassified	unclassified
837	0	3	0	1	Actinobacteria	Actinobacteria	Actinomycetales	Nakamurellaceae	unclassified
840	0	1	0	0	Actinobacteria	Actinobacteria	Solirubrobacterales	Conexibacteraceae	unclassified
843	0	1	0	0	Actinobacteria	Actinobacteria	Actinomycetales	Micrococcaceae	Rothia
845	1	7	4	2	Actinobacteria	Actinobacteria	Actinomycetales	Geodermatophilaceae	unclassified
848	0	0	0	1	Actinobacteria	Actinobacteria	Solirubrobacterales	Conexibacteraceae	unclassified
863	2	4	6	7	Actinobacteria	Actinobacteria	Actinomycetales	Nocardioidaceae	unclassified
876	0	0	0	1	Actinobacteria	Actinobacteria	Actinomycetales	Microbacteriaceae	Microbacterium
884	0	3	0	0	Actinobacteria	Actinobacteria	Actinomycetales	Micromonosporaceae	unclassified
910	0	3	0	0	Actinobacteria	Actinobacteria	Actinomycetales	Nocardioidaceae	Aeromicrobium
921	0	3	0	0	Actinobacteria	Actinobacteria	Actinomycetales	unclassified	unclassified
938	0	4	0	0	Actinobacteria	Actinobacteria	Actinomycetales	Propionibacteriaceae	Aestuariimicrobium
942	2	3	1	0	Actinobacteria	Actinobacteria	Solirubrobacterales	Solirubrobacteraceae	unclassified
944	3	38	13	14	Actinobacteria	Actinobacteria	Actinomycetales	Nocardioidaceae	Friedmanniella
981	1	1	0	0	Actinobacteria	Actinobacteria	Acidimicrobiales	Acidimicrobiaceae	unclassified
984	0	0	0	1	Actinobacteria	Actinobacteria	MC47	unclassified	unclassified
985	3	4	2	4	Actinobacteria	Actinobacteria	Acidimicrobiales	CL500-29	unclassified
986	0	0	1	0	Actinobacteria	Actinobacteria	Actinomycetales	Williamsiaceae	Williamsia
992	0	2	0	0	Actinobacteria	Actinobacteria	Actinomycetales	unclassified	unclassified
1009	2	17	6	14	Actinobacteria	Actinobacteria	Actinomycetales	Dermabacteraceae	Brachybacterium
1014	0	1	1	1	Actinobacteria	Actinobacteria	Solirubrobacterales	Conexibacteraceae	unclassified
1027	0	0	1	0	Actinobacteria	Actinobacteria	Coriobacteriales	Coriobacteriaceae	Atopobium
1028	0	1	0	0	Actinobacteria	Actinobacteria	Actinomycetales	Nocardioidaceae	Nocardioides
1034	0	4	0	0	Actinobacteria	Actinobacteria	Acidimicrobiales	Microthrixaceae	unclassified
1103	9	0	3	5	Actinobacteria	Actinobacteria	Actinomycetales	Dermacoccaceae	Kytococcus
1111	0	1	0	0	Actinobacteria	Actinobacteria	Solirubrobacterales	Conexibacteraceae	unclassified
1115	0	1	0	0	Actinobacteria	Actinobacteria	Solirubrobacterales	Conexibacteraceae	unclassified
1127	1	1	0	1	Actinobacteria	Actinobacteria	Actinomycetales	Cellulomonadaceae	unclassified
1141	0	1	0	0	Actinobacteria	Actinobacteria	Actinomycetales	Propionibacteriaceae	Tessaracoccus
1142	0	1	0	0	Actinobacteria	Actinobacteria	Actinomycetales	Kineosporiaceae	unclassified
1144	0	3	0	0	Actinobacteria	Actinobacteria	Acidimicrobiales	Iamiaceae	unclassified
1146	0	7	0	0	Actinobacteria	Actinobacteria	Actinomycetales	unclassified	unclassified
1150	0	10	1	1	Actinobacteria	Actinobacteria	Actinomycetales	Nocardioidaceae	Nocardioides
1154	0	1	0	0	Actinobacteria	Actinobacteria	Actinomycetales	Nocardioidaceae	unclassified
1164	15	11	23	10	Actinobacteria	Actinobacteria	Actinomycetales	Micrococcaceae	Kocuria
1165	0	1	0	0	Actinobacteria	Actinobacteria	Actinomycetales	Nocardioidaceae	Nocardioides
1168	0	4	0	0	Actinobacteria	Actinobacteria	Actinomycetales	Nocardioidaceae	Friedmanniella
1179	1	0	0	0	Actinobacteria	Actinobacteria	Solirubrobacterales	Patulibacteraceae	unclassified
1193	0	0	2	4	Actinobacteria	Actinobacteria	Actinomycetales	Propionibacteriaceae	Propionibacterium
1202	1	14	0	0	Actinobacteria	Actinobacteria	Actinomycetales	Microbacteriaceae	Leucobacter
1204	0	0	0	1	Actinobacteria	Actinobacteria	MC47	unclassified	unclassified
1205	0	0	1	1	Actinobacteria	Actinobacteria	Actinomycetales	Mycobacteriaceae	Mycobacterium
1222	0	0	0	2	Actinobacteria	Actinobacteria	Actinomycetales	Propionibacteriaceae	Propionibacterium
1228	0	0	1	0	Actinobacteria	Actinobacteria	0319-7L14	unclassified	unclassified
1230	0	1	1	0	Actinobacteria	Actinobacteria	Actinomycetales	unclassified	unclassified
1243	1	0	0	0	Actinobacteria	Actinobacteria	Actinomycetales	Microbacteriaceae	Agromyces
1310	1	6	16	5	Actinobacteria	Actinobacteria	Actinomycetales	Brevibacteriaceae	Brevibacterium
1321	0	1	3	0	Actinobacteria	Actinobacteria	Actinomycetales	Sanguibacteraceae	unclassified
1325	0	0	0	1	Actinobacteria	Actinobacteria	Actinomycetales	unclassified	unclassified
1327	0	0	0	1	Actinobacteria	Actinobacteria	Solirubrobacterales	Conexibacteraceae	unclassified
1329	0	48	0	0	Actinobacteria	Actinobacteria	Actinomycetales	Propionibacteriaceae	Tessaracoccus
1331	0	1	0	0	Actinobacteria	Actinobacteria	Acidimicrobiales	EB1017	unclassified
1378	0	4	2	1	Actinobacteria	Actinobacteria	Actinomycetales	Geodermatophilaceae	Geodermatophilus
1404	0	26	3	2	Actinobacteria	Actinobacteria	Actinomycetales	Micrococcaceae	Arthrobacter
1410	0	0	1	0	Actinobacteria	Actinobacteria	Actinomycetales	Microbacteriaceae	Plantibacter
1412	0	0	1	0	Actinobacteria	Actinobacteria	Actinomycetales	unclassified	unclassified

Table S5 (part C): OTUs, no. of reads and corresponding taxonomic affiliation of detected bacterial 16S rRNA amplicons by 454 pyrotag sequencing. Please note, the data shown here was either rank normalized or sum normlaized (to 5000 reads per sample) before performing statisical analysis as depicted in the manuscript.

	Samples and no. of reads				taxonomic classification of bacterial 16S rRNA gene amplicons				
OTU	UR	CO	CR8	CR5	phylum	class	order	family	genus
1425	3	2	1	1	Actinobacteria	Actinobacteria	Actinomycetales	Geodermatophilaceae	Modestobacter
1427	0	3	1	0	Actinobacteria	Actinobacteria	Acidimicrobiales	Microthrixaceae	unclassified
1456	0	0	0	1	Actinobacteria	Actinobacteria	MC47	unclassified	unclassified
1468	0	1	0	0	Actinobacteria	Actinobacteria	Actinomycetales	Kineosporiaceae	Kineococcus
1488	0	13	0	0	Actinobacteria	Actinobacteria	Acidimicrobiales	CL500-29	unclassified
1492	0	0	0	1	Actinobacteria	Actinobacteria	Actinomycetales	Nocardioidaceae	unclassified
1495	2	0	12	5	Actinobacteria	Actinobacteria	Actinomycetales	Brevibacteriaceae	Brevibacterium
1511	0	0	1	0	Actinobacteria	Actinobacteria	Acidimicrobiales	CL500-29	unclassified
1517	0	1	0	0	Actinobacteria	Actinobacteria	Actinomycetales	Jonesiaceae	unclassified
1523	0	1	0	0	Actinobacteria	Actinobacteria	Acidimicrobiales	Acidimicrobiaceae	unclassified
1524	0	1	2	0	Actinobacteria	Actinobacteria	Actinomycetales	unclassified	unclassified
1529	0	2	0	0	Actinobacteria	Actinobacteria	Acidimicrobiales	CL500-29	unclassified
1531	0	0	3	1	Actinobacteria	Actinobacteria	Actinomycetales	Dermabacteraceae	Dermabacter
1532	0	0	0	1	Actinobacteria	Actinobacteria	Acidimicrobiales	CL500-29	unclassified
1534	0	1	0	3	Actinobacteria	Actinobacteria	Actinomycetales	Kineosporiaceae	unclassified
1536	0	1	1	1	Actinobacteria	Actinobacteria	Actinomycetales	unclassified	unclassified
1539	0	1	0	0	Actinobacteria	Actinobacteria	Actinomycetales	Micromonosporaceae	unclassified
1545	0	1	0	0	Actinobacteria	Actinobacteria	Solirubrobacterales	Conexibacteraceae	unclassified
1578	1	0	0	0	Actinobacteria	Actinobacteria	MC47	unclassified	unclassified
1599	0	29	4	1	Actinobacteria	Actinobacteria	Actinomycetales	Intrasporangiaceae	Terracoccus
1601	0	0	0	1	Actinobacteria	Actinobacteria	Actinomycetales	unclassified	unclassified
1634	0	0	2	0	Actinobacteria	Actinobacteria	Solirubrobacterales	Conexibacteraceae	unclassified
1638	1	0	3	1	Actinobacteria	Actinobacteria	Actinomycetales	Corynebacteriaceae	Corynebacterium
1649	0	1	1	2	Actinobacteria	Actinobacteria	Actinomycetales	Pseudonocardiaceae	Pseudonocardia
1656	0	1	0	0	Actinobacteria	Actinobacteria	Actinomycetales	Intrasporangiaceae	Arsenicicoccus
1660	1	5	0	0	Actinobacteria	Actinobacteria	Actinomycetales	Bogoriellaceae	Georgenia
1671	1	0	0	0	Actinobacteria	Actinobacteria	Solirubrobacterales	Conexibacteraceae	unclassified
1676	0	8	2	1	Actinobacteria	Actinobacteria	Actinomycetales	Microbacteriaceae	Frigoribacterium
1678	0	1	0	0	Actinobacteria	Actinobacteria	Acidimicrobiales	EB1017	unclassified
1689	1	45	0	0	Actinobacteria	Actinobacteria	Actinomycetales	Nocardioidaceae	Propionicimonas
1696	0	0	2	5	Actinobacteria	Actinobacteria	Actinomycetales	Corynebacteriaceae	Corynebacterium
1705	3	17	2	2	Actinobacteria	Actinobacteria	Actinomycetales	unclassified	unclassified
1717	0	14	0	0	Actinobacteria	Actinobacteria	Actinomycetales	unclassified	unclassified
18	0	1	0	0	Armatimonadetes	Armatimonadia	Armatimonadales	WD294	unclassified
29	0	0	1	0	Armatimonadetes	CH21	unclassified	unclassified	unclassified
101	0	0	0	1	Armatimonadetes	Armatimonadia	Armatimonadales	Armatimonadaceae	unclassified
197	0	1	0	0	Armatimonadetes	Armatimonadia	Armatimonadales	Armatimonadaceae	unclassified
447	0	1	0	0	Armatimonadetes	Armatimonadia	Armatimonadales	Armatimonadaceae	unclassified
647	1	0	0	0	Armatimonadetes	CH21	unclassified	unclassified	unclassified
796	4	0	0	0	Armatimonadetes	Armatimonadia	Armatimonadales	Armatimonadaceae	unclassified
893	0	2	0	0	Armatimonadetes	Armatimonadia	Armatimonadales	Armatimonadaceae	unclassified
902	0	1	0	0	Armatimonadetes	Armatimonadia	Armatimonadales	Armatimonadaceae	unclassified
1031	0	0	0	1	Armatimonadetes	CH21	unclassified	unclassified	unclassified
1549	0	0	0	2	Armatimonadetes	Chthonomonadetes	Chthonomonadales	Chthonomonadaceae	Chthonomonas
1672	0	0	0	1	Armatimonadetes	S1a-1H	unclassified	unclassified	unclassified
2	0	1	0	0	Bacteroidetes	Sphingobacteria	Sphingobacteriales	Chitinophagaceae	unclassified
5	0	1	0	0	Bacteroidetes	Sphingobacteria	Sphingobacteriales	Chitinophagaceae	unclassified
13	0	0	1	0	Bacteroidetes	Flavobacteria	Flavobacteriales	Flavobacteriaceae	Flavobacterium
22	1	0	1	0	Bacteroidetes	Flavobacteria	Flavobacteriales	Flavobacteriaceae	Chryseobacterium
30	0	3	1	0	Bacteroidetes	Flavobacteria	Flavobacteriales	Flavobacteriaceae	unclassified
41	0	1	0	0	Bacteroidetes	Bacteroidia	Bacteroidales	Bacteroidaceae	Bacteroides
45	0	2	0	0	Bacteroidetes	Flavobacteria	Flavobacteriales	Flavobacteriaceae	Chryseobacterium
53	0	1	0	0	Bacteroidetes	Sphingobacteria	Sphingobacteriales	Flammeovirgaceae	unclassified
57	0	1	0	0	Bacteroidetes	Flavobacteria	Flavobacteriales	Flavobacteriaceae	Flavobacterium
77	0	1	0	0	Bacteroidetes	Flavobacteria	Flavobacteriales	Flavobacteriaceae	Aequorivita
80	1	4	2	5	Bacteroidetes	Bacteroidia	Bacteroidales	Porphyromonadaceae	Porphyromonas
84	0	4	0	0	Bacteroidetes	Bacteroidia	Bacteroidales	RikenellaceaeII	unclassified
89	0	0	5	6	Bacteroidetes	Flavobacteria	Flavobacteriales	Flavobacteriaceae	Chryseobacterium
96	0	0	2	0	Bacteroidetes	Bacteroidia	Bacteroidales	Porphyromonadaceae	Candidatus_Azobacteroides
110	0	1	0	0	Bacteroidetes	Flavobacteria	Flavobacteriales	Flavobacteriaceae	Flavobacterium
124	0	1	0	0	Bacteroidetes	Sphingobacteria	Sphingobacteriales	Flexibacteraceae	Spirosoma
126	0	1	0	0	Bacteroidetes	Bacteroidia	Bacteroidales	RikenellaceaeII	unclassified
128	0	1	0	0	Bacteroidetes	Sphingobacteria	Sphingobacteriales	Flexibacteraceae	Spirosoma
133	0	1	0	0	Bacteroidetes	unclassified	unclassified	unclassified	unclassified
136	0	2	0	0	Bacteroidetes	Sphingobacteria	Sphingobacteriales	Chitinophagaceae	unclassified
137	0	1	0	0	Bacteroidetes	Flavobacteria	Flavobacteriales	Flavobacteriaceae	Flavobacterium
142	0	8	0	0	Bacteroidetes	Flavobacteria	Flavobacteriales	Flavobacteriaceae	Aequorivita
154	0	1	1	0	Bacteroidetes	Sphingobacteria	Sphingobacteriales	Chitinophagaceae	Flavisolibacter
160	0	1	0	0	Bacteroidetes	Bacteroidia	Bacteroidales	RikenellaceaeII	unclassified
166	0	1	0	0	Bacteroidetes	Sphingobacteria	Sphingobacteriales	Flexibacteraceae	unclassified
168	0	0	1	0	Bacteroidetes	Bacteroidia	Bacteroidales	Bacteroidaceae	Bacteroides
182	0	0	0	1	Bacteroidetes	Bacteroidia	Bacteroidales	Porphyromonadaceae	Porphyromonas
188	0	2	5	0	Bacteroidetes	Sphingobacteria	Sphingobacteriales	Flexibacteraceae	Hymenobacter
193	0	1	0	0	Bacteroidetes	Sphingobacteria	Sphingobacteriales	Flexibacteraceae	Spirosoma
200	0	0	1	0	Bacteroidetes	Sphingobacteria	Sphingobacteriales	Flexibacteraceae	Hymenobacter
203	0	0	4	0	Bacteroidetes	Sphingobacteria	Sphingobacteriales	Flexibacteraceae	unclassified
215	0	0	2	2	Bacteroidetes	Sphingobacteria	Sphingobacteriales	Chitinophagaceae	unclassified
218	0	3	0	0	Bacteroidetes	Bacteroidia	Bacteroidales	Porphyromonadaceae	Porphyromonas
220	0	0	2	0	Bacteroidetes	Sphingobacteria	Sphingobacteriales	Sphingobacteriaceae	Pedobacter
239	0	0	0	1	Bacteroidetes	Sphingobacteria	Sphingobacteriales	Rhodothermaceae	unclassified
240	0	0	1	0	Bacteroidetes	Flavobacteria	Flavobacteriales	Flavobacteriaceae	Flavobacterium
243	0	1	0	0	Bacteroidetes	Sphingobacteria	Sphingobacteriales	Chitinophagaceae	unclassified
244	0	4	0	0	Bacteroidetes	Sphingobacteria	Sphingobacteriales	Flexibacteraceae	Spirosoma
247	0	1	0	0	Bacteroidetes	Flavobacteria	Flavobacteriales	Flavobacteriaceae	Flavobacterium
248	0	1	0	0	Bacteroidetes	Sphingobacteria	Sphingobacteriales	Chitinophagaceae	unclassified
253	0	0	1	0	Bacteroidetes	Sphingobacteria	Sphingobacteriales	Flexibacteraceae	Hymenobacter
268	0	2	0	0	Bacteroidetes	Sphingobacteria	Sphingobacteriales	Chitinophagaceae	unclassified
281	0	0	2	2	Bacteroidetes	Bacteroidia	Bacteroidales	unclassified	unclassified
305	0	0	1	1	Bacteroidetes	Sphingobacteria	Sphingobacteriales	Flexibacteraceae	Hymenobacter
308	0	1	0	2	Bacteroidetes	Sphingobacteria	Sphingobacteriales	Chitinophagaceae	unclassified
309	0	3	0	1	Bacteroidetes	Sphingobacteria	Sphingobacteriales	Chitinophagaceae	Segetibacter
324	0	22	0	0	Bacteroidetes	Bacteroidia	Bacteroidales	unclassified	unclassified
326	0	0	0	3	Bacteroidetes	Flavobacteria	Flavobacteriales	Flavobacteriaceae	unclassified
334	1	9	6	6	Bacteroidetes	Sphingobacteria	Sphingobacteriales	Flexibacteraceae	Hymenobacter
354	0	2	0	0	Bacteroidetes	Sphingobacteria	Sphingobacteriales	Flexibacteraceae	Dyadobacter
380	0	0	0	1	Bacteroidetes	Bacteroidia	Bacteroidales	Prevotellaceae	Prevotella
388	0	0	2	0	Bacteroidetes	Sphingobacteria	Sphingobacteriales	Chitinophagaceae	unclassified
389	0	1	0	0	Bacteroidetes	Sphingobacteria	Sphingobacteriales	Sphingobacteriaceae	Sphingobacterium
391	0	0	1	2	Bacteroidetes	Sphingobacteria	Sphingobacteriales	Chitinophagaceae	unclassified
393	2	0	0	1	Bacteroidetes	Flavobacteria	Flavobacteriales	Flavobacteriaceae	unclassified

Table S5 (part D): OTUs, no. of reads and corresponding taxonomic affiliation of detected bacterial 16S rRNA amplicons by 454 pyrotag sequencing. Please note, the data shown here was either rank normalized or sum normlaized (to 5000 reads per sample) before performing statisical analysis as depicted in the manuscript.

	Samples and no. of reads				taxonomic classification of bacterial 16S rRNA gene amplicons				
OTU	UR	CO	CR8	CR5	phylum	class	order	family	genus
400	0	2	0	0	Bacteroidetes	Sphingobacteria	Sphingobacteriales	Flexibacteraceae	Spirosoma
417	0	2	0	0	Bacteroidetes	Sphingobacteria	Sphingobacteriales	Chitinophagaceae	unclassified
425	1	0	2	1	Bacteroidetes	Sphingobacteria	Sphingobacteriales	Sphingobacteriaceae	Pedobacter
432	0	19	0	0	Bacteroidetes	Sphingobacteria	Sphingobacteriales	Chitinophagaceae	unclassified
434	0	1	0	0	Bacteroidetes	Sphingobacteria	Sphingobacteriales	Chitinophagaceae	Segetibacter
436	0	0	0	3	Bacteroidetes	Bacteroidia	Bacteroidales	Prevotellaceae	unclassified
438	0	0	0	1	Bacteroidetes	Bacteroidia	Bacteroidales	Prevotellaceae	Prevotella
439	0	0	0	1	Bacteroidetes	Bacteroidia	Bacteroidales	RikenellaceaeII	unclassified
442	0	1	3	0	Bacteroidetes	Sphingobacteria	Sphingobacteriales	Flexibacteraceae	unclassified
443	0	0	2	0	Bacteroidetes	Sphingobacteria	Sphingobacteriales	Flexibacteraceae	Hymenobacter
445	0	0	0	2	Bacteroidetes	Flavobacteria	Flavobacteriales	Flavobacteriaceae	Flavobacterium
455	0	0	0	1	Bacteroidetes	Sphingobacteria	Sphingobacteriales	Chitinophagaceae	unclassified
461	0	1	0	0	Bacteroidetes	Sphingobacteria	Sphingobacteriales	unclassified	unclassified
481	0	1	0	1	Bacteroidetes	Sphingobacteria	Sphingobacteriales	Flexibacteraceae	Hymenobacter
487	0	0	0	2	Bacteroidetes	Sphingobacteria	Sphingobacteriales	Flexibacteraceae	Hymenobacter
502	0	0	1	0	Bacteroidetes	Sphingobacteria	Sphingobacteriales	Flexibacteraceae	Hymenobacter
507	0	0	0	2	Bacteroidetes	Sphingobacteria	Sphingobacteriales	Flexibacteraceae	Hymenobacter
516	0	0	3	0	Bacteroidetes	Sphingobacteria	Sphingobacteriales	Chitinophagaceae	unclassified
526	0	1	0	0	Bacteroidetes	Sphingobacteria	Sphingobacteriales	Flexibacteraceae	Spirosoma
534	0	1	0	0	Bacteroidetes	Sphingobacteria	Sphingobacteriales	Flexibacteraceae	Hymenobacter
545	0	0	0	2	Bacteroidetes	Sphingobacteria	Sphingobacteriales	Flexibacteraceae	Hymenobacter
546	0	2	0	1	Bacteroidetes	Sphingobacteria	Sphingobacteriales	Flexibacteraceae	Hymenobacter
551	0	0	0	1	Bacteroidetes	Sphingobacteria	Sphingobacteriales	Chitinophagaceae	unclassified
554	0	0	0	2	Bacteroidetes	Sphingobacteria	Sphingobacteriales	Flexibacteraceae	unclassified
555	0	3	0	0	Bacteroidetes	Flavobacteria	Flavobacteriales	Flavobacteriaceae	Wautersiella
569	2	2	5	2	Bacteroidetes	Sphingobacteria	Sphingobacteriales	Rhodothermaceae	unclassified
572	0	2	0	0	Bacteroidetes	Sphingobacteria	Sphingobacteriales	Flexibacteraceae	Spirosoma
573	0	0	0	2	Bacteroidetes	Sphingobacteria	Sphingobacteriales	Flexibacteraceae	unclassified
586	1	0	0	0	Bacteroidetes	Sphingobacteria	Sphingobacteriales	Saprospiraceae	unclassified
589	0	1	0	0	Bacteroidetes	Sphingobacteria	Sphingobacteriales	Flexibacteraceae	Hymenobacter
593	0	7	0	0	Bacteroidetes	Bacteroidia	Bacteroidales	RikenellaceaeII	unclassified
599	0	0	1	0	Bacteroidetes	Flavobacteria	Flavobacteriales	Flavobacteriaceae	Capnocytophaga
615	0	0	2	0	Bacteroidetes	Bacteroidia	Bacteroidales	RikenellaceaeII	unclassified
625	0	0	0	1	Bacteroidetes	Sphingobacteria	Sphingobacteriales	Rhodothermaceae	unclassified
635	0	2	0	1	Bacteroidetes	Sphingobacteria	Sphingobacteriales	Flexibacteraceae	Hymenobacter
637	0	0	1	0	Bacteroidetes	Sphingobacteria	Sphingobacteriales	Flexibacteraceae	Spirosoma
648	0	2	6	2	Bacteroidetes	Sphingobacteria	Sphingobacteriales	Flexibacteraceae	Hymenobacter
671	0	0	3	0	Bacteroidetes	Bacteroidia	Bacteroidales	Porphyromonadaceae	Candidatus_Azobacteroides
679	0	0	2	3	Bacteroidetes	Bacteroidia	Bacteroidales	unclassified	unclassified
689	0	2	0	0	Bacteroidetes	Sphingobacteria	Sphingobacteriales	Flexibacteraceae	Cytophaga
693	1	3	0	1	Bacteroidetes	Sphingobacteria	Sphingobacteriales	unclassified	unclassified
702	0	0	0	1	Bacteroidetes	Sphingobacteria	Sphingobacteriales	Flexibacteraceae	Spirosoma
704	0	0	0	1	Bacteroidetes	Sphingobacteria	Sphingobacteriales	Chitinophagaceae	Segetibacter
710	0	0	3	0	Bacteroidetes	Sphingobacteria	Sphingobacteriales	Flexibacteraceae	Cytophaga
723	0	1	0	0	Bacteroidetes	Sphingobacteria	Sphingobacteriales	Flexibacteraceae	Dyadobacter
737	0	0	0	1	Bacteroidetes	Bacteroidia	Bacteroidales	RikenellaceaeII	unclassified
741	0	1	5	1	Bacteroidetes	Sphingobacteria	Sphingobacteriales	Flexibacteraceae	Dyadobacter
764	0	0	2	0	Bacteroidetes	Sphingobacteria	Sphingobacteriales	Chitinophagaceae	unclassified
778	0	0	2	0	Bacteroidetes	Sphingobacteria	Sphingobacteriales	unclassified	unclassified
783	3	0	0	0	Bacteroidetes	Flavobacteria	Flavobacteriales	Flavobacteriaceae	Aequorivita
792	0	0	2	0	Bacteroidetes	Bacteroidia	Bacteroidales	Bacteroidaceae	Bacteroides
799	0	1	2	0	Bacteroidetes	Flavobacteria	Flavobacteriales	Flavobacteriaceae	Flavobacterium
802	0	1	0	0	Bacteroidetes	Flavobacteria	Flavobacteriales	Flavobacteriaceae	Flavobacterium
805	0	0	0	2	Bacteroidetes	Sphingobacteria	Sphingobacteriales	Sphingobacteriaceae	unclassified
817	0	0	0	1	Bacteroidetes	Bacteroidia	Bacteroidales	Prevotellaceae	Prevotella
818	0	1	0	3	Bacteroidetes	Sphingobacteria	Sphingobacteriales	Flexibacteraceae	unclassified
829	0	8	0	8	Bacteroidetes	Sphingobacteria	Sphingobacteriales	Flexibacteraceae	Hymenobacter
846	0	0	2	0	Bacteroidetes	Sphingobacteria	Sphingobacteriales	Chitinophagaceae	unclassified
860	0	0	8	1	Bacteroidetes	Sphingobacteria	Sphingobacteriales	Flexibacteraceae	Cytophaga
861	0	7	0	0	Bacteroidetes	Sphingobacteria	Sphingobacteriales	Saprospiraceae	unclassified
891	0	1	0	0	Bacteroidetes	Bacteroidia	Bacteroidales	RikenellaceaeII	unclassified
908	0	0	2	0	Bacteroidetes	Sphingobacteria	Sphingobacteriales	Flexibacteraceae	Hymenobacter
916	0	5	0	1	Bacteroidetes	Sphingobacteria	Sphingobacteriales	Chitinophagaceae	unclassified
918	2	0	0	0	Bacteroidetes	Sphingobacteria	Sphingobacteriales	Chitinophagaceae	unclassified
922	0	22	0	0	Bacteroidetes	Bacteroidia	Bacteroidales	Porphyromonadaceae	unclassified
931	0	1	0	0	Bacteroidetes	Sphingobacteria	Sphingobacteriales	Flexibacteraceae	Cytophaga
932	0	0	0	2	Bacteroidetes	Flavobacteria	Flavobacteriales	Flavobacteriaceae	Flavobacterium
936	1	1	1	7	Bacteroidetes	Bacteroidia	Bacteroidales	Prevotellaceae	Prevotella
937	0	1	4	1	Bacteroidetes	Sphingobacteria	Sphingobacteriales	unclassified	unclassified
940	0	1	0	0	Bacteroidetes	Flavobacteria	Flavobacteriales	Flavobacteriaceae	Gelidibacter
941	0	0	1	0	Bacteroidetes	Sphingobacteria	Sphingobacteriales	Chitinophagaceae	unclassified
959	0	0	0	1	Bacteroidetes	Sphingobacteria	Sphingobacteriales	Flexibacteraceae	unclassified
963	0	1	0	0	Bacteroidetes	unclassified	unclassified	unclassified	unclassified
976	0	0	2	0	Bacteroidetes	Sphingobacteria	Sphingobacteriales	Chitinophagaceae	unclassified
977	0	2	0	3	Bacteroidetes	Sphingobacteria	Sphingobacteriales	Flexibacteraceae	unclassified
987	0	0	5	0	Bacteroidetes	Sphingobacteria	Sphingobacteriales	Flexibacteraceae	Cytophaga
991	0	2	0	0	Bacteroidetes	Flavobacteria	Flavobacteriales	Flavobacteriaceae	Wautersiella
1000	0	0	1	0	Bacteroidetes	Sphingobacteria	Sphingobacteriales	Sphingobacteriaceae	Sphingobacterium
1007	0	1	0	0	Bacteroidetes	Bacteroidia	Bacteroidales	Prevotellaceae	Prevotella
1011	0	1	0	0	Bacteroidetes	Flavobacteria	Flavobacteriales	Flavobacteriaceae	unclassified
1019	0	0	2	0	Bacteroidetes	Sphingobacteria	Sphingobacteriales	Chitinophagaceae	unclassified
1033	0	1	0	0	Bacteroidetes	Sphingobacteria	Sphingobacteriales	Sphingobacteriaceae	unclassified
1041	0	1	0	0	Bacteroidetes	unclassified	unclassified	unclassified	unclassified
1045	0	0	1	0	Bacteroidetes	Sphingobacteria	Sphingobacteriales	Saprospiraceae	unclassified
1064	0	2	0	0	Bacteroidetes	Bacteroidia	Bacteroidales	Porphyromonadaceae	Parabacteroides
1083	0	0	0	1	Bacteroidetes	Flavobacteria	Flavobacteriales	Flavobacteriaceae	Flavobacterium
1088	0	6	0	0	Bacteroidetes	Flavobacteria	Flavobacteriales	Corymorphaceae	unclassified
1090	0	0	0	1	Bacteroidetes	Flavobacteria	Flavobacteriales	Flavobacteriaceae	Flavobacterium
1091	0	1	2	3	Bacteroidetes	Sphingobacteria	Sphingobacteriales	Sphingobacteriaceae	Pedobacter
1095	0	1	0	0	Bacteroidetes	Flavobacteria	Flavobacteriales	Flavobacteriaceae	Chryseobacterium
1096	0	3	1	0	Bacteroidetes	Sphingobacteria	Sphingobacteriales	Chitinophagaceae	unclassified
1116	0	1	0	0	Bacteroidetes	Sphingobacteria	Sphingobacteriales	Chitinophagaceae	unclassified
1121	0	0	0	1	Bacteroidetes	Flavobacteria	Flavobacteriales	Flavobacteriaceae	Flavobacterium
1123	0	0	0	1	Bacteroidetes	Bacteroidia	Bacteroidales	Prevotellaceae	Prevotella
1131	2	0	0	0	Bacteroidetes	Sphingobacteria	Sphingobacteriales	Chitinophagaceae	unclassified
1152	0	5	0	0	Bacteroidetes	unclassified	unclassified	unclassified	unclassified
1156	0	1	0	1	Bacteroidetes	Sphingobacteria	Sphingobacteriales	Flexibacteraceae	Hymenobacter
1157	0	1	0	0	Bacteroidetes	Sphingobacteria	Sphingobacteriales	Flexibacteraceae	unclassified
1166	0	1	0	0	Bacteroidetes	Flavobacteria	Flavobacteriales	Flavobacteriaceae	Gillisia
1173	0	0	2	0	Bacteroidetes	Sphingobacteria	Sphingobacteriales	Flexibacteraceae	unclassified

Table S5 (part E): OTUs, no. of reads and corresponding taxonomic affiliation of detected bacterial 16S rRNA amplicons by 454 pyrotag sequencing. Please note, the data shown here was either rank normalized or sum normlaized (to 5000 reads per sample) before performing statisical analysis as depicted in the manuscript.

	Samples and no. of reads				taxonomic classification of bacterial 16S rRNA gene amplicons				
OTU	UR	CO	CR8	CR5	phylum	class	order	family	genus
1180	0	2	0	0	Bacteroidetes	Sphingobacteria	Sphingobacteriales	Flexibacteraceae	Spirosoma
1182	0	0	3	5	Bacteroidetes	Bacteroidia	Bacteroidales	Prevotellaceae	Prevotella
1184	0	0	1	0	Bacteroidetes	Sphingobacteria	Sphingobacteriales	Sphingobacteriaceae	unclassified
1188	0	0	0	1	Bacteroidetes	Flavobacteria	Flavobacteriales	Flavobacteriaceae	Flavobacterium
1200	0	0	0	2	Bacteroidetes	Sphingobacteria	Sphingobacteriales	Flexibacteraceae	Hymenobacter
1213	0	1	0	1	Bacteroidetes	Sphingobacteria	Sphingobacteriales	Flexibacteraceae	Spirosoma
1217	0	1	0	0	Bacteroidetes	Sphingobacteria	Sphingobacteriales	Saprospiraceae	unclassified
1218	0	1	0	0	Bacteroidetes	Flavobacteria	Flavobacteriales	Flavobacteriaceae	Sejongia
1224	0	0	5	0	Bacteroidetes	Sphingobacteria	Sphingobacteriales	Flexibacteraceae	unclassified
1231	0	1	0	0	Bacteroidetes	Flavobacteria	Flavobacteriales	Flavobacteriaceae	Aequorivita
1233	0	0	0	1	Bacteroidetes	Bacteroidia	Bacteroidales	Bacteroidaceae	Bacteroides
1234	0	1	0	0	Bacteroidetes	Flavobacteria	Flavobacteriales	Corymorphaceae	unclassified
1250	0	0	1	1	Bacteroidetes	Bacteroidia	Bacteroidales	Prevotellaceae	Prevotella
1252	1	0	0	0	Bacteroidetes	Sphingobacteria	Sphingobacteriales	Flexibacteraceae	unclassified
1257	0	1	0	0	Bacteroidetes	Flavobacteria	Flavobacteriales	Flavobacteriaceae	Arenibacter
1258	0	0	1	0	Bacteroidetes	Sphingobacteria	Sphingobacteriales	Flexibacteraceae	Hymenobacter
1262	0	0	1	0	Bacteroidetes	Flavobacteria	Flavobacteriales	Flavobacteriaceae	Flavobacterium
1264	0	1	0	1	Bacteroidetes	Sphingobacteria	Sphingobacteriales	Sphingobacteriaceae	unclassified
1273	0	1	2	0	Bacteroidetes	Bacteroidia	Bacteroidales	unclassified	unclassified
1274	1	0	0	2	Bacteroidetes	Sphingobacteria	Sphingobacteriales	Flexibacteraceae	Hymenobacter
1286	0	2	0	0	Bacteroidetes	Sphingobacteria	Sphingobacteriales	Cyclobacteriaceae	unclassified
1287	0	3	0	0	Bacteroidetes	Sphingobacteria	Sphingobacteriales	Flexibacteraceae	Spirosoma
1297	0	0	2	0	Bacteroidetes	Sphingobacteria	Sphingobacteriales	Chitinophagaceae	unclassified
1298	0	1	0	0	Bacteroidetes	Sphingobacteria	Sphingobacteriales	unclassified	unclassified
1300	0	0	0	1	Bacteroidetes	Sphingobacteria	Sphingobacteriales	Flexibacteraceae	Spirosoma
1303	2	2	1	1	Bacteroidetes	Flavobacteria	Flavobacteriales	Flavobacteriaceae	Chryseobacterium
1320	0	0	0	1	Bacteroidetes	Sphingobacteria	Sphingobacteriales	Flexibacteraceae	Emticicia
1330	0	0	0	1	Bacteroidetes	Bacteroidia	Bacteroidales	unclassified	unclassified
1333	0	6	0	0	Bacteroidetes	Sphingobacteria	Sphingobacteriales	Chitinophagaceae	unclassified
1345	0	0	1	0	Bacteroidetes	Sphingobacteria	Sphingobacteriales	unclassified	unclassified
1351	1	0	0	0	Bacteroidetes	Sphingobacteria	Sphingobacteriales	Flexibacteraceae	Spirosoma
1352	1	0	0	0	Bacteroidetes	Sphingobacteria	Sphingobacteriales	Sphingobacteriaceae	Sphingobacterium
1356	0	0	0	4	Bacteroidetes	Sphingobacteria	Sphingobacteriales	Chitinophagaceae	unclassified
1359	0	0	0	1	Bacteroidetes	Bacteroidia	Bacteroidales	Prevotellaceae	Prevotella
1363	0	1	0	0	Bacteroidetes	unclassified	unclassified	unclassified	unclassified
1364	0	5	0	0	Bacteroidetes	Bacteroidia	Bacteroidales	Marinilabiaceae	unclassified
1366	1	6	0	3	Bacteroidetes	Sphingobacteria	Sphingobacteriales	Flexibacteraceae	Hymenobacter
1382	0	2	7	2	Bacteroidetes	Sphingobacteria	Sphingobacteriales	Flexibacteraceae	Hymenobacter
1393	0	2	1	0	Bacteroidetes	Sphingobacteria	Sphingobacteriales	Saprospiraceae	unclassified
1396	0	8	0	0	Bacteroidetes	Flavobacteria	Flavobacteriales	Flavobacteriaceae	Aequorivita
1400	0	0	1	0	Bacteroidetes	Bacteroidia	Bacteroidales	Porphyromonadaceae	unclassified
1401	0	0	6	0	Bacteroidetes	Sphingobacteria	Sphingobacteriales	Flexibacteraceae	Cytophaga
1424	0	5	0	0	Bacteroidetes	Flavobacteria	Flavobacteriales	Flavobacteriaceae	unclassified
1434	0	1	0	0	Bacteroidetes	Sphingobacteria	Sphingobacteriales	Flammeovirgaceae	unclassified
1444	0	4	0	0	Bacteroidetes	Sphingobacteria	Sphingobacteriales	Sphingobacteriaceae	unclassified
1452	0	1	0	2	Bacteroidetes	Sphingobacteria	Sphingobacteriales	Flexibacteraceae	Hymenobacter
1473	1	3	0	2	Bacteroidetes	Flavobacteria	Flavobacteriales	Flavobacteriaceae	Chryseobacterium
1480	0	0	0	1	Bacteroidetes	Sphingobacteria	Sphingobacteriales	Chitinophagaceae	unclassified
1489	77	0	0	0	Bacteroidetes	Flavobacteria	Flavobacteriales	Flavobacteriaceae	Wautersiella
1490	0	0	2	0	Bacteroidetes	Sphingobacteria	Sphingobacteriales	Sphingobacteriaceae	unclassified
1494	0	2	0	0	Bacteroidetes	Sphingobacteria	Sphingobacteriales	Chitinophagaceae	Flavisolibacter
1498	0	1	0	0	Bacteroidetes	Sphingobacteria	Sphingobacteriales	Chitinophagaceae	unclassified
1499	0	1	0	0	Bacteroidetes	Bacteroidia	Bacteroidales	Prevotellaceae	Prevotella
1500	0	3	0	0	Bacteroidetes	Bacteroidia	Bacteroidales	Porphyromonadaceae	unclassified
1502	0	2	0	0	Bacteroidetes	Sphingobacteria	Sphingobacteriales	unclassified	unclassified
1525	0	0	0	1	Bacteroidetes	Sphingobacteria	Sphingobacteriales	Saprospiraceae	unclassified
1544	0	1	0	0	Bacteroidetes	Sphingobacteria	Sphingobacteriales	Flexibacteraceae	Spirosoma
1547	0	0	2	0	Bacteroidetes	Sphingobacteria	Sphingobacteriales	Flexibacteraceae	Spirosoma
1557	0	0	0	1	Bacteroidetes	Flavobacteria	Flavobacteriales	Flavobacteriaceae	Capnocytophaga
1568	0	0	0	1	Bacteroidetes	Sphingobacteria	Sphingobacteriales	Chitinophagaceae	unclassified
1574	0	11	0	0	Bacteroidetes	Flavobacteria	Flavobacteriales	Flavobacteriaceae	unclassified
1576	1	0	0	0	Bacteroidetes	Sphingobacteria	Sphingobacteriales	Saprospiraceae	unclassified
1585	0	0	2	0	Bacteroidetes	Sphingobacteria	Sphingobacteriales	Chitinophagaceae	unclassified
1596	0	1	0	0	Bacteroidetes	Bacteroidia	Bacteroidales	Porphyromonadaceae	Parabacteroides
1610	0	1	0	0	Bacteroidetes	unclassified	unclassified	unclassified	unclassified
1615	0	0	3	0	Bacteroidetes	Sphingobacteria	Sphingobacteriales	Flexibacteraceae	Emticicia
1616	0	3	0	0	Bacteroidetes	Sphingobacteria	Sphingobacteriales	Chitinophagaceae	unclassified
1622	0	0	12	0	Bacteroidetes	Bacteroidia	Bacteroidales	Prevotellaceae	Prevotella
1626	0	2	0	0	Bacteroidetes	unclassified	unclassified	unclassified	unclassified
1630	0	1	0	0	Bacteroidetes	Sphingobacteria	Sphingobacteriales	Flexibacteraceae	Dyadobacter
1635	0	2	0	0	Bacteroidetes	unclassified	unclassified	unclassified	unclassified
1653	0	0	0	4	Bacteroidetes	Bacteroidia	Bacteroidales	Prevotellaceae	Prevotella
1663	0	0	1	0	Bacteroidetes	Sphingobacteria	Sphingobacteriales	Chitinophagaceae	unclassified
1668	0	0	0	1	Bacteroidetes	Sphingobacteria	Sphingobacteriales	Sphingobacteriaceae	unclassified
1669	1	0	1	9	Bacteroidetes	Bacteroidia	Bacteroidales	Prevotellaceae	Prevotella
1677	0	0	0	3	Bacteroidetes	Bacteroidia	Bacteroidales	Bacteroidaceae	Bacteroides
1699	0	1	0	1	Bacteroidetes	Sphingobacteria	Sphingobacteriales	Flexibacteraceae	Spirosoma
1700	0	0	0	1	Bacteroidetes	Sphingobacteria	Sphingobacteriales	Flexibacteraceae	Spirosoma
1720	0	1	1	0	Bacteroidetes	Sphingobacteria	Sphingobacteriales	unclassified	unclassified
1721	0	3	0	0	Bacteroidetes	Sphingobacteria	Sphingobacteriales	Chitinophagaceae	unclassified
955	0	1	0	0	BRC1	PRR-11	unclassified	unclassified	unclassified
19	0	2	0	0	Chlorobi	Chlorobia	Chlorobiales	unclassified	unclassified
373	0	2	1	0	Chlorobi	Chlorobia	Chlorobiales	SJA-28	unclassified
703	0	0	0	1	Chlorobi	Ignavibacteria	Ignavibacteriales	Ignavibacteriaceae	unclassified
3	0	0	2	0	Chloroflexi	Anaerolineae	Anaerolineales	Anaerolinaceae	A4b
196	0	2	0	1	Chloroflexi	SOGA31	unclassified	unclassified	unclassified
298	0	1	0	0	Chloroflexi	Chloroflexi	Herpetosiphonales	Herpetosiphonaceae	Herpetosiphon
356	0	1	0	0	Chloroflexi	Chloroflexi	Chloroflexales	unclassified	unclassified
623	0	2	0	0	Chloroflexi	Anaerolineae	Anaerolineales	Anaerolinaceae	unclassified
674	0	1	0	0	Chloroflexi	Thermomicrobia	Sphaerobacterales	Sphaerobacterineae	HN1-15
856	0	0	1	0	Chloroflexi	SOGA31	unclassified	unclassified	unclassified
957	0	4	0	0	Chloroflexi	Thermomicrobia	Sphaerobacterales	Sphaerobacterineae	HN1-15
1084	0	0	0	1	Chloroflexi	Chloroflexi	Chloroflexales	Chloroflexaceae	Roseiflexus
1107	0	1	1	0	Chloroflexi	Thermomicrobia	Sphaerobacterales	Sphaerobacterineae	HN1-15
1113	0	1	0	0	Chloroflexi	SOGA31	unclassified	unclassified	unclassified
1171	0	8	0	0	Chloroflexi	Thermomicrobia	Sphaerobacterales	Sphaerobacterineae	HN1-15
1305	0	1	0	0	Chloroflexi	Bljii12	B07_WMSP1	FFCH4570	unclassified
1423	0	0	2	0	Chloroflexi	Anaerolineae	Anaerolineales	Anaerolinaceae	WCHB1-50
1453	0	0	0	1	Chloroflexi	Thermomicrobia	Sphaerobacterales	Sphaerobacterineae	HN1-15
1476	0	1	0	0	Chloroflexi	Anaerolineae	Anaerolineales	Anaerolinaceae	A4b

Table S5 (part F): OTUs, no. of reads and corresponding taxonomic affiliation of detected bacterial 16S rRNA amplicons by 454 pyrotag sequencing. Please note, the data shown here was either rank normalized or sum normlaized (to 5000 reads per sample) before performing statisical analysis as depicted in the manuscript.

	Samples and no. of reads				taxonomic classification of bacterial 16S rRNA gene amplicons				
OTU	UR	CO	CR8	CR5	phylum	class	order	family	genus
1520	0	1	0	0	Chloroflexi	Anaerolineae	Anaerolineales	Anaerolinaceae	Anaerolinea
1583	0	0	0	1	Chloroflexi	TK17	mle1-48	unclassified	unclassified
1603	0	0	2	0	Chloroflexi	unclassified	unclassified	unclassified	unclassified
102	10	0	0	0	Cyanobacteria	Nostocophycideae	Nostocales	unclassified	unclassified
148	0	0	0	3	Cyanobacteria	Synechococcophycideae	Pseudanabaenales	Pseudanabaenaceae	Leptolyngbya
164	0	1	0	1	Cyanobacteria	Oscillatoriophycideae	Chroococcales	Xenococcaceae	Chroococcidiopsis
170	0	3	0	5	Cyanobacteria	unclassified	unclassified	unclassified	unclassified
173	0	0	1	2	Cyanobacteria	Oscillatoriophycideae	Chroococcales	Xenococcaceae	Chroococcidiopsis
207	0	1	0	0	Cyanobacteria	Synechococcophycideae	Pseudanabaenales	Pseudanabaenaceae	unclassified
230	0	4	0	0	Cyanobacteria	unclassified	unclassified	unclassified	unclassified
306	0	0	0	1	Cyanobacteria	Oscillatoriophycideae	Chroococcales	Xenococcaceae	unclassified
314	0	20	2	6	Cyanobacteria	Oscillatoriophycideae	Chroococcales	Xenococcaceae	Chroococcidiopsis
351	0	1	0	0	Cyanobacteria	Chloroplast	Streptophyta	unclassified	unclassified
387	0	3	0	0	Cyanobacteria	Synechococcophycideae	Pseudanabaenales	Pseudanabaenaceae	Leptolyngbya
403	0	0	0	2	Cyanobacteria	Synechococcophycideae	Pseudanabaenales	Pseudanabaenaceae	Leptolyngbya
426	0	1	1	0	Cyanobacteria	Synechococcophycideae	Pseudanabaenales	Pseudanabaenaceae	Leptolyngbya
430	0	0	1	0	Cyanobacteria	Chloroplast	unclassified	unclassified	unclassified
437	0	0	0	1	Cyanobacteria	unclassified	unclassified	unclassified	unclassified
444	0	1	0	2	Cyanobacteria	Synechococcophycideae	Pseudanabaenales	Pseudanabaenaceae	Leptolyngbya
477	1	4	0	2	Cyanobacteria	Synechococcophycideae	Pseudanabaenales	Pseudanabaenaceae	Pseudana-Phormidium
496	0	3	0	0	Cyanobacteria	Oscillatoriophycideae	unclassified	unclassified	unclassified
524	0	2	0	0	Cyanobacteria	unclassified	unclassified	unclassified	unclassified
527	0	13	0	0	Cyanobacteria	Nostocophycideae	Nostocales	Nostocaceae	Nostoc
530	0	5	2	4	Cyanobacteria	Oscillatoriophycideae	Oscillatoriales	Phormidiaceae	Phormidium
542	0	2	0	0	Cyanobacteria	Oscillatoriophycideae	Oscillatoriales	Phormidiaceae	Phormidium
543	0	1	0	0	Cyanobacteria	Synechococcophycideae	Pseudanabaenales	Pseudanabaenaceae	Pseudana-Phormidium
584	0	0	0	3	Cyanobacteria	Oscillatoriophycideae	unclassified	unclassified	unclassified
591	0	1	0	0	Cyanobacteria	Oscillatoriophycideae	Chroococcales	Xenococcaceae	Chroococcidiopsis
594	0	8	0	0	Cyanobacteria	Oscillatoriophycideae	Oscillatoriales	Phormidiaceae	Phormidium
604	0	0	3	5	Cyanobacteria	Chloroplast	Chlorophyta	Trebouxiophyceae	unclassified
614	0	1	0	0	Cyanobacteria	Synechococcophycideae	Pseudanabaenales	Pseudanabaenaceae	Leptolyngbya
641	0	0	1	0	Cyanobacteria	4C0d-2	SM1D11	unclassified	unclassified
646	0	0	0	7	Cyanobacteria	Oscillatoriophycideae	Chroococcales	Xenococcaceae	Chroococcidiopsis
676	0	1	0	0	Cyanobacteria	Oscillatoriophycideae	Chroococcales	Xenococcaceae	Chroococcidiopsis
744	0	2	0	0	Cyanobacteria	Synechococcophycideae	Pseudanabaenales	Pseudanabaenaceae	Pseudana-Phormidium
761	0	0	3	0	Cyanobacteria	4C0d-2	mle1-12	unclassified	unclassified
774	0	1	0	0	Cyanobacteria	Oscillatoriophycideae	Chroococcales	Xenococcaceae	Chroococcidiopsis
793	0	0	2	0	Cyanobacteria	Chloroplast	Stramenopiles	unclassified	unclassified
808	0	0	0	3	Cyanobacteria	unclassified	unclassified	unclassified	unclassified
821	0	4	0	2	Cyanobacteria	Synechococcophycideae	Pseudanabaenales	Pseudanabaenaceae	Leptolyngbya
828	0	3	0	0	Cyanobacteria	Nostocophycideae	Nostocales	Nostocaceae	Nostoc
847	0	1	0	0	Cyanobacteria	unclassified	unclassified	unclassified	unclassified
872	0	3	4	1	Cyanobacteria	Chloroplast	Chlorophyta	Trebouxiophyceae	unclassified
1062	0	0	2	0	Cyanobacteria	Chloroplast	Chlorophyta	Trebouxiophyceae	unclassified
1099	0	1	0	0	Cyanobacteria	unclassified	unclassified	unclassified	unclassified
1101	1	16	8	6	Cyanobacteria	Oscillatoriophycideae	unclassified	unclassified	unclassified
1106	0	2	0	3	Cyanobacteria	unclassified	unclassified	unclassified	unclassified
1108	0	4	9	7	Cyanobacteria	unclassified	unclassified	unclassified	unclassified
1140	2	0	0	0	Cyanobacteria	Chloroplast	Stramenopiles	unclassified	unclassified
1145	0	1	0	0	Cyanobacteria	unclassified	unclassified	unclassified	unclassified
1163	0	0	0	1	Cyanobacteria	Oscillatoriophycideae	unclassified	unclassified	unclassified
1235	0	0	0	1	Cyanobacteria	Synechococcophycideae	Pseudanabaenales	Pseudanabaenaceae	unclassified
1236	1	4	0	0	Cyanobacteria	Oscillatoriophycideae	Chroococcales	Microcystaceae	unclassified
1245	0	0	1	0	Cyanobacteria	Oscillatoriophycideae	Oscillatoriales	Phormidiaceae	unclassified
1290	0	2	3	1	Cyanobacteria	Oscillatoriophycideae	Oscillatoriales	Phormidiaceae	Phormidium
1324	0	2	0	0	Cyanobacteria	Synechococcophycideae	Pseudanabaenales	Pseudanabaenaceae	Leptolyngbya
1326	0	0	2	5	Cyanobacteria	Chloroplast	Streptophyta	unclassified	unclassified
1337	1	6	2	0	Cyanobacteria	Nostocophycideae	Nostocales	Rivulariaceae	Calothrix
1340	0	0	0	2	Cyanobacteria	unclassified	unclassified	unclassified	unclassified
1383	0	0	2	0	Cyanobacteria	S15B-MN24	unclassified	unclassified	unclassified
1415	0	0	0	1	Cyanobacteria	Chloroplast	Streptophyta	unclassified	unclassified
1416	0	1	0	9	Cyanobacteria	Synechococcophycideae	Pseudanabaenales	Pseudanabaenaceae	unclassified
1429	0	1	0	1	Cyanobacteria	Oscillatoriophycideae	Chroococcales	Xenococcaceae	Chroococcidiopsis
1436	0	0	0	2	Cyanobacteria	Synechococcophycideae	Pseudanabaenales	Pseudanabaenaceae	Leptolyngbya
1481	0	1	0	1	Cyanobacteria	Oscillatoriophycideae	Chroococcales	Xenococcaceae	Chroococcidiopsis
1508	0	1	0	0	Cyanobacteria	Synechococcophycideae	Pseudanabaenales	Pseudanabaenaceae	unclassified
1521	0	2	0	0	Cyanobacteria	Chloroplast	Chlorophyta	Trebouxiophyceae	unclassified
1533	0	2	0	5	Cyanobacteria	Synechococcophycideae	Pseudanabaenales	Pseudanabaenaceae	unclassified
1542	0	3	3	0	Cyanobacteria	Nostocophycideae	Nostocales	Rivulariaceae	Calothrix
1571	1	2	0	0	Cyanobacteria	Oscillatoriophycideae	Chroococcales	Xenococcaceae	Chroococcidiopsis
1580	0	5	7	14	Cyanobacteria	Chloroplast	Streptophyta	unclassified	unclassified
1608	0	0	1	0	Cyanobacteria	unclassified	unclassified	unclassified	unclassified
1611	0	2	1	3	Cyanobacteria	Chloroplast	unclassified	unclassified	unclassified
1625	0	10	2	1	Cyanobacteria	Chloroplast	Streptophyta	unclassified	unclassified
1636	0	3	0	1	Cyanobacteria	Oscillatoriophycideae	Oscillatoriales	Phormidiaceae	Phormidium
1658	3	0	0	0	Cyanobacteria	Synechococcophycideae	Pseudanabaenales	Pseudanabaenaceae	Leptolyngbya
1710	0	1	0	0	Cyanobacteria	unclassified	unclassified	unclassified	unclassified
1711	0	0	0	2	Cyanobacteria	Oscillatoriophycideae	Chroococcales	Xenococcaceae	unclassified
0	1	2	0	0	Firmicutes	Bacilli	Bacillales	Bacillaceae	Geobacillus
7	0	0	9	0	Firmicutes	Clostridia	Clostridiales	Ruminococcaceae	unclassified
8	0	2	0	0	Firmicutes	Clostridia	Clostridiales	Clostridiaceae	Alkaliphilus
12	0	10	7	7	Firmicutes	Bacilli	Bacillales	Staphylococcaceae	Jeotgalicoccus
14	0	0	1	0	Firmicutes	Clostridia	Clostridiales	Clostridiales_Family_XI._Incertae_Sedis	Finegoldia
24	0	0	0	1	Firmicutes	Bacilli	Lactobacillales	Lactobacillaceae	Lactobacillus
26	0	0	1	0	Firmicutes	Clostridia	Clostridiales	Clostridiales_Family_XI._Incertae_Sedis	Peptoniphilus
27	3	9	6	4	Firmicutes	Bacilli	Gemellales	Gemellaceae	Gemella
32	0	1	0	0	Firmicutes	Clostridia	Clostridiales	Ruminococcaceae	unclassified
33	0	0	1	0	Firmicutes	Clostridia	Clostridiales	unclassified	unclassified
55	0	0	0	1	Firmicutes	Bacilli	Lactobacillales	Aerococcaceae	unclassified
65	3	0	0	37	Firmicutes	Clostridia	Clostridiales	Clostridiales_Family_XI._Incertae_Sedis	Anaerococcus
67	0	0	1	2	Firmicutes	Bacilli	Lactobacillales	Streptococcaceae	Streptococcus
73	0	0	1	0	Firmicutes	Clostridia	Clostridiales	Lachnospiraceae	unclassified
76	0	0	0	1	Firmicutes	Clostridia	Clostridiales	Clostridiaceae	Clostridium
94	0	2	0	0	Firmicutes	Clostridia	Clostridiales	Ruminococcaceae	Faecalibacterium
97	0	1	0	0	Firmicutes	Bacilli	Bacillales	Planococcaceae	Ureibacillus
112	0	0	76	0	Firmicutes	Bacilli	Bacillales	Paenibacillaceae	Paenibacillus
114	4	5	11	12	Firmicutes	Clostridia	Clostridiales	Clostridiales_Family_XI._Incertae_Sedis	Anaerococcus
116	3	6	2	45	Firmicutes	Bacilli	Lactobacillales	Aerococcaceae	unclassified
139	0	2	0	0	Firmicutes	Bacilli	Bacillales	Paenibacillaceae	Paenibacillus
140	0	0	1	0	Firmicutes	Clostridia	Clostridiales	Clostridiales_Family_XI._Incertae_Sedis	Anaerococcus

Table S5 (part G): OTUs, no. of reads and corresponding taxonomic affiliation of detected bacterial 16S rRNA amplicons by 454 pyrotag sequencing. Please note, the data shown here was either rank normalized or sum normlaized (to 5000 reads per sample) before performing statisical analysis as depicted in the manuscript.

	Samples and no. of reads				taxonomic classification of bacterial 16S rRNA gene amplicons				
OTU	UR	CO	CR8	CR5	phylum	class	order	family	genus
149	3	20	13	2	Firmicutes	Clostridia	Clostridiales	Veillonellaceae	Veillonella
150	2	1	0	3	Firmicutes	Bacilli	Bacillales	Planococcaceae	Solibacillus
151	3	3	15	1	Firmicutes	Bacilli	Lactobacillales	Lactobacillaceae	Lactobacillus
153	0	0	2	0	Firmicutes	Clostridia	Clostridiales	Veillonellaceae	Desulfosporomusa
156	2	0	0	0	Firmicutes	Clostridia	Clostridiales	Clostridiaceae	Clostridium
165	2	6	0	4	Firmicutes	Clostridia	Clostridiales	Ruminococcaceae	Subdoligranulum
167	0	2	0	0	Firmicutes	Bacilli	Bacillales	Bacillaceae	Bacillus
174	0	0	2	0	Firmicutes	Clostridia	Clostridiales	unclassified	unclassified
181	6	1	23	12	Firmicutes	Clostridia	Clostridiales	Clostridiales_Family_XI._Incertae_Sedis	Peptoniphilus
198	1	0	0	0	Firmicutes	Clostridia	Clostridiales	Clostridiales_Family_XIII._Incertae_Sedis	unclassified
201	0	0	1	0	Firmicutes	Clostridia	Clostridiales	Ruminococcaceae	unclassified
202	1	0	0	0	Firmicutes	Bacilli	Bacillales	Bacillaceae	Bacillus
209	0	3	3	9	Firmicutes	Clostridia	Clostridiales	Clostridiales_Family_XI._Incertae_Sedis	unclassified
211	0	1	0	0	Firmicutes	Clostridia	Clostridiales	Lachnospiraceae	Oribacterium
212	0	0	0	1	Firmicutes	Clostridia	Clostridiales	Ruminococcaceae	Ruminococcus
214	0	2	0	0	Firmicutes	Bacilli	Bacillales	Bacillaceae	unclassified
217	2	3	11	91	Firmicutes	Clostridia	Clostridiales	Clostridiales_Family_XI._Incertae_Sedis	Anaerococcus
219	0	1	0	0	Firmicutes	Clostridia	Clostridiales	Lachnospiraceae	unclassified
223	0	0	1	0	Firmicutes	Clostridia	Clostridiales	Ruminococcaceae	unclassified
231	0	1	0	0	Firmicutes	Clostridia	Clostridiales	Catabacteriaceae	unclassified
234	0	0	0	1	Firmicutes	Clostridia	Clostridiales	Ruminococcaceae	Ethanoligenens
252	1	1	8	4	Firmicutes	Clostridia	Clostridiales	Clostridiales_Family_XI._Incertae_Sedis	Anaerococcus
254	0	1	1	1	Firmicutes	Clostridia	Clostridiales	unclassified	unclassified
262	1	1	0	0	Firmicutes	Bacilli	Bacillales	Planococcaceae	Ureibacillus
263	2	17	18	4	Firmicutes	Bacilli	Lactobacillales	Streptococcaceae	Streptococcus
266	0	0	1	0	Firmicutes	Clostridia	Clostridiales	Lachnospiraceae	Clostridium
267	0	3	4	54	Firmicutes	Bacilli	Lactobacillales	Aerococcaceae	unclassified
270	0	1	0	0	Firmicutes	Bacilli	Bacillales	Bacillaceae	Bacillus
272	0	0	3	0	Firmicutes	Bacilli	Lactobacillales	Streptococcaceae	Streptococcus
282	0	0	2	0	Firmicutes	Clostridia	MBA08	unclassified	unclassified
285	0	1	0	0	Firmicutes	Bacilli	Bacillales	unclassified	unclassified
287	0	3	8	0	Firmicutes	Bacilli	Bacillales	Listeriaceae	Brochothrix
288	0	0	1	0	Firmicutes	Bacilli	Bacillales	unclassified	unclassified
299	0	17	0	0	Firmicutes	Clostridia	Clostridiales	Clostridiales_Family_XI._Incertae_Sedis	Tissierella
313	0	1	1	0	Firmicutes	Clostridia	Clostridiales	Lachnospiraceae	unclassified
333	0	4	0	3	Firmicutes	Clostridia	Clostridiales	Lachnospiraceae	Eubacterium
337	0	0	0	1	Firmicutes	Clostridia	Clostridiales	Lachnospiraceae	Clostridium
347	0	4	0	0	Firmicutes	Clostridia	Clostridiales	Lachnospiraceae	unclassified
348	0	1	0	0	Firmicutes	Clostridia	Clostridiales	Lachnospiraceae	Bacteroides
352	0	0	0	2	Firmicutes	Clostridia	Clostridiales	Lachnospiraceae	Coprococcus
353	0	0	0	1	Firmicutes	Clostridia	Clostridiales	Lachnospiraceae	unclassified
361	0	1	1	0	Firmicutes	Clostridia	Clostridiales	Lachnospiraceae	Clostridium
378	0	0	0	1	Firmicutes	Clostridia	Clostridiales	Lachnospiraceae	unclassified
394	0	0	0	1	Firmicutes	unclassified	unclassified	unclassified	unclassified
399	0	0	2	0	Firmicutes	Bacilli	Lactobacillales	Streptococcaceae	Streptococcus
401	1	2	3	1	Firmicutes	Bacilli	Lactobacillales	Leuconostocaceae	Leuconostoc
405	0	1	0	0	Firmicutes	Clostridia	Clostridiales	Lachnospiraceae	unclassified
414	1	1	1	0	Firmicutes	Clostridia	Clostridiales	Lachnospiraceae	Blautia
427	0	3	10	12	Firmicutes	Clostridia	Clostridiales	Clostridiales_Family_XI._Incertae_Sedis	Peptoniphilus
428	3	1	0	0	Firmicutes	Bacilli	Lactobacillales	Lactobacillaceae	Lactobacillus
431	0	1	2	0	Firmicutes	Clostridia	Clostridiales	Clostridiales_Family_XIII._Incertae_Sedis	unclassified
456	0	0	0	1	Firmicutes	Bacilli	Bacillales	Paenibacillaceae	Paenibacillus
458	0	0	0	1	Firmicutes	Bacilli	Bacillales	Paenibacillaceae	Paenibacillus
459	0	0	0	2	Firmicutes	Clostridia	Clostridiales	Clostridiales_Family_XI._Incertae_Sedis	unclassified
460	1	0	0	0	Firmicutes	Clostridia	Clostridiales	Lachnospiraceae	Blautia
466	0	58	0	0	Firmicutes	Clostridia	Clostridiales	unclassified	unclassified
471	0	1	4	1	Firmicutes	Bacilli	Bacillales	Staphylococcaceae	Macrococcus
476	2	10	9	27	Firmicutes	Clostridia	Clostridiales	Ruminococcaceae	Ruminococcus
482	0	0	5	0	Firmicutes	Bacilli	Bacillales	Bacillaceae	Bacillus
500	0	0	0	2	Firmicutes	Bacilli	Lactobacillales	Carnobacteriaceae	unclassified
506	3	0	0	0	Firmicutes	Clostridia	Clostridiales	Clostridiaceae	unclassified
513	1	0	0	0	Firmicutes	Clostridia	Clostridiales	Clostridiales_Family_XI._Incertae_Sedis	unclassified
515	0	14	7	2	Firmicutes	Bacilli	Lactobacillales	Leuconostocaceae	Weissella
517	0	0	2	0	Firmicutes	Clostridia	Clostridiales	Clostridiaceae	Clostridium
521	0	0	0	1	Firmicutes	Clostridia	Clostridiales	Veillonellaceae	Megasphaera
547	0	0	0	1	Firmicutes	Clostridia	Clostridiales	Lachnospiraceae	Clostridium
553	0	0	2	0	Firmicutes	Clostridia	Clostridiales	Lachnospiraceae	Catonella
562	0	0	0	1	Firmicutes	Clostridia	Clostridiales	Veillonellaceae	Dialister
577	25	9	49	125	Firmicutes	Clostridia	Clostridiales	Clostridiales_Family_XI._Incertae_Sedis	Finegoldia
578	0	0	2	0	Firmicutes	Clostridia	Clostridiales	Lachnospiraceae	Clostridium
592	0	0	1	0	Firmicutes	Bacilli	Bacillales	Paenibacillaceae	Cohnella
595	4	1	25	32	Firmicutes	Clostridia	Clostridiales	Clostridiales_Family_XI._Incertae_Sedis	Anaerococcus
597	1	3	0	1	Firmicutes	Bacilli	Lactobacillales	Lactobacillaceae	Lactobacillus
602	0	2	0	0	Firmicutes	Clostridia	unclassified	unclassified	unclassified
603	0	1	0	0	Firmicutes	Clostridia	Clostridiales	Eubacteriaceae	unclassified
606	0	0	2	0	Firmicutes	Bacilli	Lactobacillales	Carnobacteriaceae	Alloiococcus
610	1	0	0	0	Firmicutes	Clostridia	Clostridiales	Lachnospiraceae	Epulopiscium
616	0	0	0	1	Firmicutes	Bacilli	Lactobacillales	Carnobacteriaceae	unclassified
618	0	10	0	0	Firmicutes	Clostridia	Clostridiales	Clostridiales_Family_XI._Incertae_Sedis	unclassified
621	0	0	2	0	Firmicutes	Bacilli	Lactobacillales	Lactobacillaceae	Lactobacillus
640	0	0	1	0	Firmicutes	Clostridia	Clostridiales	Veillonellaceae	unclassified
643	0	0	1	1	Firmicutes	Clostridia	Clostridiales	Lachnospiraceae	Coprococcus
650	0	1	0	0	Firmicutes	Clostridia	Clostridiales	Lachnospiraceae	unclassified
657	5	4	4	0	Firmicutes	Bacilli	Lactobacillales	Lactobacillaceae	Lactobacillus
658	0	1	0	0	Firmicutes	Clostridia	Clostridiales	unclassified	unclassified
659	0	1	0	0	Firmicutes	Bacilli	Bacillales	Bacillaceae	Bacillus
677	0	4	0	0	Firmicutes	Clostridia	Clostridiales	Clostridiales_Family_XI._Incertae_Sedis	Tissierella
682	0	0	2	0	Firmicutes	Clostridia	Clostridiales	unclassified	unclassified
701	0	0	0	1	Firmicutes	Clostridia	Clostridiales	Ruminococcaceae	Clostridium
711	0	0	2	0	Firmicutes	Bacilli	Bacillales	Paenibacillaceae	Paenibacillus
713	0	0	1	0	Firmicutes	Bacilli	Bacillales	Staphylococcaceae	Staphylococcus
733	0	1	0	0	Firmicutes	Clostridia	Clostridiales	Lachnospiraceae	unclassified
743	1	0	2	0	Firmicutes	Clostridia	Clostridiales	Peptostreptococcaceae	Peptostreptococcus
747	0	0	0	1	Firmicutes	Clostridia	Clostridiales	Lachnospiraceae	unclassified
750	0	1	4	2	Firmicutes	Bacilli	Lactobacillales	unclassified	unclassified
751	0	0	2	0	Firmicutes	Clostridia	Clostridiales	Clostridiaceae	Clostridium
772	0	3	0	0	Firmicutes	Clostridia	Clostridiales	Clostridiales_Family_XI._Incertae_Sedis	Tissierella
777	0	0	1	0	Firmicutes	Clostridia	Clostridiales	Clostridiaceae	Clostridium
788	0	0	3	0	Firmicutes	Clostridia	Clostridiales	unclassified	unclassified
797	1	0	0	0	Firmicutes	Bacilli	Bacillales	Bacillaceae	unclassified

Table S5 (part H): OTUs, no. of reads and corresponding taxonomic affiliation of detected bacterial 16S rRNA amplicons by 454 pyrotag sequencing. Please note, the data shown here was either rank normalized or sum normlaized (to 5000 reads per sample) before performing statisical analysis as depicted in the manuscript.

	Samples and no. of reads				taxonomic classification of bacterial 16S rRNA gene amplicons				
OTU	UR	CO	CR8	CR5	phylum	class	order	family	genus
798	1	0	0	0	Firmicutes	Bacilli	Bacillales	Bacillaceae	unclassified
800	0	6	10	7	Firmicutes	Clostridia	Clostridiales	Lachnospiraceae	Blautia
801	0	0	0	3	Firmicutes	Clostridia	Clostridiales	Ruminococcaceae	unclassified
806	0	0	0	1	Firmicutes	Clostridia	Clostridiales	Clostridiales_Family_XI._Incertae_Sedis	unclassified
810	1	0	0	1	Firmicutes	Clostridia	Clostridiales	Ruminococcaceae	Ruminococcus
820	0	0	0	1	Firmicutes	Bacilli	Bacillales	Paenibacillaceae	Paenibacillus
824	11	0	1	0	Firmicutes	Bacilli	Bacillales	Planococcaceae	Rummeliibacillus
830	0	0	0	1	Firmicutes	Clostridia	Clostridiales	Veillonellaceae	Selenomonas
835	0	1	0	0	Firmicutes	Clostridia	Clostridiales	Veillonellaceae	Selenomonas
864	0	0	1	0	Firmicutes	Bacilli	Bacillales	Bacillaceae	Bacillus
865	0	0	3	0	Firmicutes	Clostridia	Clostridiales	Lachnospiraceae	Clostridium
868	0	1	0	0	Firmicutes	Clostridia	Clostridiales	Clostridiaceae	Clostridium
875	6	9	47	15	Firmicutes	Bacilli	Lactobacillales	Lactobacillaceae	Lactobacillus
879	0	1	0	0	Firmicutes	Bacilli	Bacillales	Paenibacillaceae	Paenibacillus
886	0	0	1	1	Firmicutes	Clostridia	Clostridiales	Ruminococcaceae	unclassified
887	1	0	1	0	Firmicutes	Bacilli	Bacillales	Paenibacillaceae	Paenibacillus
903	5	7	0	3	Firmicutes	Clostridia	Clostridiales	Clostridiaceae	Clostridium
912	0	0	1	0	Firmicutes	Clostridia	Clostridiales	Catabacteriaceae	unclassified
927	0	1	0	0	Firmicutes	Clostridia	Clostridiales	Lachnospiraceae	Lachnobacterium
928	1	1	0	0	Firmicutes	Clostridia	Clostridiales	Clostridiaceae	Clostridium
946	3	3	5	0	Firmicutes	Bacilli	Exiguobacterales	Exiguobacteraceae	Exiguobacterium
949	1	0	6	1	Firmicutes	Clostridia	Clostridiales	Lachnospiraceae	Coprococcus
960	0	1	0	0	Firmicutes	Clostridia	Clostridiales	Clostridiales_Family_XI._Incertae_Sedis	Parvimonas
964	0	1	0	0	Firmicutes	Clostridia	Clostridiales	Lachnospiraceae	Coprococcus
971	0	7	1	3	Firmicutes	Clostridia	Clostridiales	Ruminococcaceae	unclassified
980	1	0	1	1	Firmicutes	Clostridia	Clostridiales	Lachnospiraceae	Blautia
993	0	1	0	0	Firmicutes	Clostridia	Clostridiales	unclassified	unclassified
997	0	0	0	1	Firmicutes	Clostridia	Clostridiales	Lachnospiraceae	unclassified
1001	0	2	0	1	Firmicutes	Clostridia	Clostridiales	Lachnospiraceae	unclassified
1002	0	0	1	0	Firmicutes	Clostridia	Clostridiales	Ruminococcaceae	unclassified
1003	0	0	1	0	Firmicutes	Bacilli	Lactobacillales	Streptococcaceae	Lactococcus
1020	0	0	2	1	Firmicutes	Clostridia	Clostridiales	Clostridiales_Family_XI._Incertae_Sedis	Anaerococcus
1022	2	4	14	7	Firmicutes	Bacilli	Bacillales	Planococcaceae	unclassified
1024	0	1	0	0	Firmicutes	Clostridia	Clostridiales	Lachnospiraceae	Clostridium
1029	0	1	0	0	Firmicutes	Clostridia	Clostridiales	unclassified	unclassified
1039	3	9	12	10	Firmicutes	Bacilli	Lactobacillales	Streptococcaceae	Streptococcus
1049	0	0	0	1	Firmicutes	Bacilli	Bacillales	Bacillaceae	Bacillus
1053	2	1	14	17	Firmicutes	Bacilli	Bacillales	Paenibacillaceae	Paenibacillus
1054	0	1	0	0	Firmicutes	Clostridia	Clostridiales	Lachnospiraceae	unclassified
1058	0	0	0	1	Firmicutes	Bacilli	Bacillales	Paenibacillaceae	Paenibacillus
1069	0	0	1	1	Firmicutes	Bacilli	Lactobacillales	Carnobacteriaceae	unclassified
1072	0	1	0	0	Firmicutes	Bacilli	Bacillales	Paenibacillaceae	Paenibacillus
1074	0	0	0	1	Firmicutes	Clostridia	Clostridiales	Lachnospiraceae	unclassified
1077	0	1	0	0	Firmicutes	Clostridia	Clostridiales	Ruminococcaceae	Oscillospira
1085	1	0	0	2	Firmicutes	Clostridia	Clostridiales	Veillonellaceae	Dialister
1098	3	3	5	2	Firmicutes	Bacilli	Bacillales	Staphylococcaceae	unclassified
1112	9	0	0	0	Firmicutes	Bacilli	Lactobacillales	Lactobacillaceae	Lactobacillus
1117	0	2	0	0	Firmicutes	Clostridia	Clostridiales	Lachnospiraceae	Blautia
1118	3	2	8	5	Firmicutes	Clostridia	Clostridiales	Lachnospiraceae	Roseburia
1119	0	2	0	0	Firmicutes	Clostridia	Clostridiales	unclassified	unclassified
1120	0	0	0	1	Firmicutes	Clostridia	Clostridiales	Lachnospiraceae	unclassified
1126	0	0	2	0	Firmicutes	Clostridia	Clostridiales	Clostridiaceae	Clostridium
1128	2	0	0	0	Firmicutes	Clostridia	Clostridiales	Ruminococcaceae	unclassified
1138	0	0	2	0	Firmicutes	Clostridia	Clostridiales	Ruminococcaceae	unclassified
1139	1	1	2	0	Firmicutes	Bacilli	Lactobacillales	Carnobacteriaceae	Trichococcus
1149	0	0	0	2	Firmicutes	Clostridia	Clostridiales	Lachnospiraceae	unclassified
1161	0	1	1	0	Firmicutes	Clostridia	Clostridiales	Veillonellaceae	Selenomonas
1167	0	1	0	0	Firmicutes	Clostridia	unclassified	unclassified	unclassified
1169	6	18	7	10	Firmicutes	Bacilli	Lactobacillales	Carnobacteriaceae	Carnobacterium
1178	1	0	0	0	Firmicutes	Clostridia	Clostridiales	Ruminococcaceae	Ruminococcus
1183	2	2	7	4	Firmicutes	Clostridia	Clostridiales	Clostridiales_Family_XI._Incertae_Sedis	Anaerococcus
1190	0	0	0	2	Firmicutes	Clostridia	Clostridiales	Clostridiales_Family_XI._Incertae_Sedis	Peptoniphilus
1192	1	1	0	1	Firmicutes	Clostridia	Clostridiales	Clostridiales_Family_XI._Incertae_Sedis	Peptoniphilus
1195	0	0	0	1	Firmicutes	Clostridia	Clostridiales	Clostridiaceae	Clostridium
1216	0	0	0	1	Firmicutes	Clostridia	Clostridiales	Clostridiales_Family_XI._Incertae_Sedis	Parvimonas
1221	0	1	0	1	Firmicutes	Clostridia	Clostridiales	Lachnospiraceae	unclassified
1227	0	0	1	0	Firmicutes	Clostridia	1B07	unclassified	unclassified
1239	1	0	0	0	Firmicutes	Clostridia	Clostridiales	Veillonellaceae	Megasphaera
1242	0	0	2	0	Firmicutes	Clostridia	Clostridiales	Lachnospiraceae	unclassified
1244	1	0	0	0	Firmicutes	Bacilli	Bacillales	Staphylococcaceae	Staphylococcus
1247	0	4	0	3	Firmicutes	Clostridia	Clostridiales	Lachnospiraceae	Blautia
1265	0	0	1	0	Firmicutes	Clostridia	Clostridiales	Lachnospiraceae	unclassified
1275	0	0	0	1	Firmicutes	Clostridia	Clostridiales	Clostridiales_Family_XI._Incertae_Sedis	Peptoniphilus
1281	2	2	0	0	Firmicutes	Clostridia	Clostridiales	Lachnospiraceae	unclassified
1289	0	0	0	1	Firmicutes	Clostridia	Clostridiales	Lachnospiraceae	unclassified
1291	0	1	0	0	Firmicutes	Bacilli	Bacillales	Bacillaceae	Bacillus
1294	1	0	0	0	Firmicutes	Bacilli	unclassified	unclassified	unclassified
1299	0	0	5	0	Firmicutes	Clostridia	Clostridiales	Veillonellaceae	Veillonella
1315	0	0	1	0	Firmicutes	Clostridia	Clostridiales	Clostridiales_Family_XI._Incertae_Sedis	Anaerococcus
1318	0	0	0	4	Firmicutes	Clostridia	Clostridiales	Ruminococcaceae	unclassified
1335	0	0	1	0	Firmicutes	Clostridia	Clostridiales	Lachnospiraceae	Blautia
1336	0	1	0	3	Firmicutes	Clostridia	Clostridiales	Lachnospiraceae	unclassified
1341	0	0	0	1	Firmicutes	Clostridia	Clostridiales	Clostridiaceae	Clostridium
1342	2	0	0	0	Firmicutes	Bacilli	Turicibacterales	Turicibacteraceae	Turicibacter
1357	0	0	0	2	Firmicutes	Clostridia	Clostridiales	Clostridiales_Family_XI._Incertae_Sedis	Anaerococcus
1360	0	0	1	0	Firmicutes	Bacilli	Bacillales	Paenibacillaceae	Paenibacillus
1368	0	0	1	0	Firmicutes	Clostridia	Clostridiales	Ruminococcaceae	unclassified
1371	0	0	0	2	Firmicutes	Bacilli	Lactobacillales	Aerococcaceae	Facklamia
1372	1	0	2	3	Firmicutes	Bacilli	Lactobacillales	Aerococcaceae	Aerococcus
1374	0	1	2	0	Firmicutes	Clostridia	Clostridiales	Lachnospiraceae	Coprococcus
1375	0	0	0	5	Firmicutes	Clostridia	Clostridiales	Veillonellaceae	Anaeroglobus
1379	3	0	16	46	Firmicutes	Clostridia	Clostridiales	Clostridiales_Family_XI._Incertae_Sedis	Anaerococcus
1381	0	0	1	0	Firmicutes	Bacilli	Lactobacillales	Streptococcaceae	Streptococcus
1384	0	0	2	0	Firmicutes	Bacilli	Bacillales	Bacillaceae	Bacillus
1391	0	0	4	0	Firmicutes	Clostridia	Clostridiales	Clostridiaceae	Clostridium
1392	2	1	13	8	Firmicutes	Clostridia	Clostridiales	Clostridiales_Family_XI._Incertae_Sedis	Anaerococcus
1402	0	3	3	6	Firmicutes	Bacilli	Bacillales	Bacillaceae	Bacillus
1405	0	0	0	1	Firmicutes	Clostridia	Clostridiales	Lachnospiraceae	unclassified
1406	0	6	0	0	Firmicutes	Bacilli	Lactobacillales	Enterococcaceae	unclassified
1407	0	0	0	1	Firmicutes	Clostridia	Clostridiales	Veillonellaceae	unclassified

Table S5 (part I): OTUs, no. of reads and corresponding taxonomic affiliation of detected bacterial 16S rRNA amplicons by 454 pyrotag sequencing. Please note, the data shown here was either rank normalized or sum normlaized (to 5000 reads per sample) before performing statisical analysis as depicted in the manuscript.

	Samples and no. of reads				taxonomic classification of bacterial 16S rRNA gene amplicons				
OTU	UR	CO	CR8	CR5	phylum	class	order	family	genus
1411	0	0	0	3	Firmicutes	Clostridia	Clostridiales	Lachnospiraceae	Coprococcus
1418	0	0	1	0	Firmicutes	Clostridia	Clostridiales	Clostridiaceae	Clostridium
1421	4	9	4	5	Firmicutes	Bacilli	Lactobacillales	Enterococcaceae	Enterococcus
1422	0	1	0	0	Firmicutes	Clostridia	Clostridiales	unclassified	unclassified
1433	0	2	0	0	Firmicutes	Clostridia	Clostridiales	Ruminococcaceae	unclassified
1437	0	1	0	7	Firmicutes	Clostridia	Clostridiales	Lachnospiraceae	Ruminococcus
1438	3	0	0	4	Firmicutes	Clostridia	Clostridiales	Clostridiaceae	Clostridium
1440	0	4	3	1	Firmicutes	Bacilli	Bacillales	Alicyclobacillaceae	Alicyclobacillus
1441	0	1	0	0	Firmicutes	Bacilli	Bacillales	Planococcaceae	Bacillus
1442	2	40	11	10	Firmicutes	Bacilli	Lactobacillales	Streptococcaceae	Lactococcus
1451	0	0	0	2	Firmicutes	Clostridia	Clostridiales	Lachnospiraceae	unclassified
1483	0	0	0	1	Firmicutes	Clostridia	Clostridiales	Clostridiaceae	Clostridium
1485	0	0	1	0	Firmicutes	Bacilli	Lactobacillales	Carnobacteriaceae	Granulicatella
1491	2	0	7	3	Firmicutes	Clostridia	Clostridiales	Clostridiales_Family_XI._Incertae_Sedis	Peptoniphilus
1518	0	2	0	0	Firmicutes	Clostridia	Clostridiales	Ruminococcaceae	unclassified
1526	0	0	1	0	Firmicutes	Clostridia	OPB54	unclassified	unclassified
1555	0	0	1	4	Firmicutes	Clostridia	Clostridiales	Clostridiales_Family_XI._Incertae_Sedis	Anaerococcus
1556	5	0	0	0	Firmicutes	Bacilli	Lactobacillales	Lactobacillaceae	Lactobacillus
1558	0	0	3	0	Firmicutes	Clostridia	Clostridiales	Lachnospiraceae	unclassified
1562	4	20	3	1	Firmicutes	Clostridia	Clostridiales	Clostridiaceae	unclassified
1575	0	3	0	0	Firmicutes	Clostridia	Clostridiales	Lachnospiraceae	Ruminococcus
1579	0	0	0	1	Firmicutes	Clostridia	Clostridiales	Ruminococcaceae	Eubacterium
1581	0	0	0	1	Firmicutes	Clostridia	Clostridiales	Clostridiales_Family_XI._Incertae_Sedis	Peptoniphilus
1584	13	6	0	2	Firmicutes	Bacilli	Bacillales	Bacillaceae	Bacillus
1587	1	0	0	0	Firmicutes	Clostridia	Clostridiales	Clostridiales_Family_XIII._Incertae_Sedis	unclassified
1590	1	14	11	12	Firmicutes	Clostridia	Clostridiales	Ruminococcaceae	Faecalibacterium
1591	0	0	2	0	Firmicutes	unclassified	unclassified	unclassified	unclassified
1614	0	2	0	0	Firmicutes	unclassified	unclassified	unclassified	unclassified
1617	0	0	5	2	Firmicutes	Clostridia	Clostridiales	Veillonellaceae	Veillonella
1629	0	1	0	0	Firmicutes	Clostridia	Clostridiales	Eubacteriaceae	unclassified
1642	0	2	0	2	Firmicutes	Bacilli	Lactobacillales	Carnobacteriaceae	Desemzia
1645	0	0	0	1	Firmicutes	Clostridia	Clostridiales	Clostridiaceae	Clostridium
1650	0	10	7	3	Firmicutes	Clostridia	Clostridiales	Clostridiaceae	Clostridium
1651	0	1	0	0	Firmicutes	Clostridia	Clostridiales	Lachnospiraceae	unclassified
1652	1	0	0	0	Firmicutes	Clostridia	MBA08	unclassified	unclassified
1657	0	1	0	0	Firmicutes	Clostridia	Clostridiales	Lachnospiraceae	Coprococcus
1664	0	0	0	1	Firmicutes	unclassified	unclassified	unclassified	unclassified
1665	0	0	1	0	Firmicutes	Clostridia	Clostridiales	Clostridiales_Family_XI._Incertae_Sedis	Helcococcus
1688	0	0	1	0	Firmicutes	Clostridia	Clostridiales	Clostridiaceae	Sarcina
1693	0	0	0	1	Firmicutes	Bacilli	Lactobacillales	Streptococcaceae	Streptococcus
1694	2	0	0	2	Firmicutes	Bacilli	Lactobacillales	Aerococcaceae	Facklamia
1697	1	1	0	3	Firmicutes	Clostridia	Clostridiales	Veillonellaceae	Dialister
1704	0	2	1	3	Firmicutes	Clostridia	Clostridiales	Lachnospiraceae	Coprococcus
1707	20	2	1	0	Firmicutes	Bacilli	Lactobacillales	Lactobacillaceae	Lactobacillus
1709	0	1	0	0	Firmicutes	Clostridia	Clostridiales	Lachnospiraceae	unclassified
1722	0	1	0	0	Firmicutes	Clostridia	Clostridiales	Clostridiales_Family_XIII._Incertae_Sedis	Eubacterium
611	1	0	0	0	Fusobacteria	Fusobacteria	Fusobacteriales	Fusobacteriaceae	Fusobacterium
1207	0	6	1	0	Fusobacteria	Fusobacteria	Fusobacteriales	Fusobacteriaceae	Fusobacterium
222	0	1	0	0	Gemmatimonadetes	Gemmatimonadetes	Gemmatimonadales	Gemmatimonadaceae	unclassified
329	0	1	0	0	Gemmatimonadetes	Gemmatimonadetes	Gemmatimonadales	Gemmatimonadaceae	unclassified
355	0	1	0	0	Gemmatimonadetes	Gemmatimonadetes	Gemmatimonadales	Gemmatimonadaceae	unclassified
372	0	1	0	0	Gemmatimonadetes	Gemmatimonadetes	Gemmatimonadales	Gemmatimonadaceae	unclassified
421	0	0	1	0	Gemmatimonadetes	Gemmatimonadetes	Gemmatimonadales	Gemmatimonadaceae	unclassified
579	0	1	0	1	Gemmatimonadetes	Gemmatimonadetes	unclassified	unclassified	unclassified
619	0	2	2	0	Gemmatimonadetes	Gemmatimonadetes	Gemmatimonadales	Gemmatimonadaceae	Gemmatimonas
712	0	0	1	0	Gemmatimonadetes	Gemmatimonadetes	Gemmatimonadales	Gemmatimonadaceae	unclassified
769	1	0	0	0	Gemmatimonadetes	Gemmatimonadetes	Gemmatimonadales	Gemmatimonadaceae	unclassified
834	0	1	0	0	Gemmatimonadetes	Gemmatimonadetes	Gemmatimonadales	Gemmatimonadaceae	unclassified
841	0	3	0	0	Gemmatimonadetes	Gemmatimonadetes	Gemmatimonadales	Gemmatimonadaceae	unclassified
867	2	0	0	0	Gemmatimonadetes	Gemmatimonadetes	Gemmatimonadales	Gemmatimonadaceae	Gemmatimonas
896	0	6	0	0	Gemmatimonadetes	Gemmatimonadetes	Gemmatimonadales	Gemmatimonadaceae	unclassified
972	0	2	0	1	Gemmatimonadetes	Gemmatimonadetes	Gemmatimonadales	Gemmatimonadaceae	unclassified
1008	0	0	1	0	Gemmatimonadetes	Gemmatimonadetes	unclassified	unclassified	unclassified
1013	0	1	0	0	Gemmatimonadetes	Gemmatimonadetes	Gemmatimonadales	Gemmatimonadaceae	unclassified
1026	0	4	0	0	Gemmatimonadetes	Gemmatimonadetes	unclassified	unclassified	unclassified
1038	0	1	0	0	Gemmatimonadetes	Gemmatimonadetes	Gemmatimonadales	Gemmatimonadaceae	unclassified
1051	0	3	0	0	Gemmatimonadetes	Gemmatimonadetes	Gemmatimonadales	Gemmatimonadaceae	unclassified
1203	0	1	0	0	Gemmatimonadetes	Gemmatimonadetes	Gemmatimonadales	Gemmatimonadaceae	unclassified
1211	0	0	0	3	Gemmatimonadetes	Gemmatimonadetes	Gemmatimonadales	Gemmatimonadaceae	unclassified
1237	0	4	0	1	Gemmatimonadetes	Gemmatimonadetes	Gemmatimonadales	Gemmatimonadaceae	unclassified
1246	0	1	0	0	Gemmatimonadetes	Gemmatimonadetes	Gemmatimonadales	Gemmatimonadaceae	unclassified
1255	0	5	3	0	Gemmatimonadetes	Gemmatimonadetes	Gemmatimonadales	Gemmatimonadaceae	unclassified
1259	0	3	0	2	Gemmatimonadetes	Gemmatimonadetes	Gemmatimonadales	Gemmatimonadaceae	unclassified
1355	0	0	0	1	Gemmatimonadetes	Gemmatimonadetes	Gemmatimonadales	Gemmatimonadaceae	unclassified
1454	0	0	0	1	Gemmatimonadetes	Gemmatimonadetes	unclassified	unclassified	unclassified
1516	0	1	0	0	Gemmatimonadetes	Gemmatimonadetes	Gemmatimonadales	Gemmatimonadaceae	unclassified
134	0	0	0	1	Nitrospirae	Nitrospira	Nitrospirales	Nitrospiraceae	Nitrospira
286	0	0	1	1	Nitrospirae	Nitrospira	Nitrospirales	Nitrospiraceae	Nitrospira
947	1	0	0	0	Nitrospirae	Nitrospira	Nitrospirales	Nitrospiraceae	Nitrospira
9	0	0	1	0	Planctomycetes	Planctomycea	Gemmatales	Isosphaeraceae	unclassified
23	0	2	0	0	Planctomycetes	Planctomycea	Planctomycetales	Planctomycetaceae	unclassified
70	1	0	0	0	Planctomycetes	Planctomycea	Gemmatales	Gemmataceae	unclassified
158	0	0	1	0	Planctomycetes	Planctomycea	Planctomycetales	Planctomycetaceae	Planctomyces
300	0	0	1	0	Planctomycetes	Planctomycea	Gemmatales	Gemmataceae	Gemmata
311	0	1	0	0	Planctomycetes	Planctomycea	Gemmatales	Gemmataceae	unclassified
362	0	0	0	1	Planctomycetes	Planctomycea	Planctomycetales	Planctomycetaceae	A17
413	0	0	2	0	Planctomycetes	Planctomycea	Gemmatales	Isosphaeraceae	unclassified
415	0	0	1	0	Planctomycetes	Planctomycea	Planctomycetales	Planctomycetaceae	unclassified
422	0	7	0	0	Planctomycetes	Planctomycea	Planctomycetales	Planctomycetaceae	Rhodopirellula
503	0	0	0	1	Planctomycetes	Planctomycea	Planctomycetales	Planctomycetaceae	unclassified
571	0	0	0	1	Planctomycetes	Planctomycea	Planctomycetales	Planctomycetaceae	Planctomyces
598	0	0	0	1	Planctomycetes	Planctomycea	Gemmatales	Gemmataceae	Gemmata
700	0	1	0	0	Planctomycetes	Planctomycea	Gemmatales	Gemmataceae	unclassified
717	0	0	1	0	Planctomycetes	Planctomycea	Gemmatales	Gemmataceae	unclassified
724	0	10	0	0	Planctomycetes	Planctomycea	Planctomycetales	Planctomycetaceae	Rhodopirellula
728	0	1	0	0	Planctomycetes	Planctomycea	Gemmatales	Gemmataceae	Gemmata
729	0	1	0	0	Planctomycetes	Planctomycea	Planctomycetales	Planctomycetaceae	unclassified
748	0	0	0	1	Planctomycetes	Planctomycea	Gemmatales	Isosphaeraceae	unclassified
803	0	3	0	0	Planctomycetes	Planctomycea	Planctomycetales	Planctomycetaceae	unclassified
814	0	0	0	2	Planctomycetes	Planctomycea	Planctomycetales	Planctomycetaceae	Planctomyces

Table S5 (part J): OTUs, no. of reads and corresponding taxonomic affiliation of detected bacterial 16S rRNA amplicons by 454 pyrotag sequencing. Please note, the data shown here was either rank normalized or sum normlaized (to 5000 reads per sample) before performing statisical analysis as depicted in the manuscript.

	Samples and no. of reads				taxonomic classification of bacterial 16S rRNA gene amplicons				
OTU	UR	CO	CR8	CR5	phylum	class	order	family	genus
862	0	0	2	0	Planctomycetes	Planctomycea	Gemmatales	Gemmataceae	Gemmata
998	0	5	0	2	Planctomycetes	Planctomycea	Gemmatales	Isosphaeraceae	unclassified
999	0	0	1	0	Planctomycetes	Planctomycea	Gemmatales	Gemmataceae	unclassified
1060	0	2	0	0	Planctomycetes	Planctomycea	Gemmatales	unclassified	unclassified
1181	0	1	0	0	Planctomycetes	Planctomycea	Planctomycetales	Planctomycetaceae	unclassified
1225	0	0	2	0	Planctomycetes	Planctomycea	Gemmatales	Gemmataceae	Gemmata
1241	0	0	1	0	Planctomycetes	Planctomycea	Gemmatales	Gemmataceae	unclassified
1249	0	1	1	0	Planctomycetes	Planctomycea	Gemmatales	Gemmataceae	Gemmata
1276	0	1	0	0	Planctomycetes	Planctomycea	Gemmatales	Gemmataceae	Gemmata
1278	0	1	0	2	Planctomycetes	Planctomycea	Gemmatales	Isosphaeraceae	unclassified
1296	0	1	0	0	Planctomycetes	Planctomycea	Planctomycetales	Planctomycetaceae	unclassified
1361	0	0	2	0	Planctomycetes	Planctomycea	Planctomycetales	Planctomycetaceae	Planctomyces
1386	0	0	1	0	Planctomycetes	Planctomycea	Gemmatales	Isosphaeraceae	unclassified
1389	0	0	1	0	Planctomycetes	Planctomycea	Planctomycetales	Planctomycetaceae	Pirellula
1445	0	1	0	1	Planctomycetes	Planctomycea	Gemmatales	Gemmataceae	Gemmata
1496	0	0	1	0	Planctomycetes	Planctomycea	Planctomycetales	Planctomycetaceae	Rhodopirellula
1506	0	0	5	0	Planctomycetes	Planctomycea	Planctomycetales	Planctomycetaceae	Planctomyces
1550	0	1	0	0	Planctomycetes	Planctomycea	Gemmatales	Gemmataceae	Gemmata
1606	0	0	2	0	Planctomycetes	Planctomycea	Gemmatales	Isosphaeraceae	unclassified
1618	0	0	0	1	Planctomycetes	Planctomycea	Gemmatales	Isosphaeraceae	unclassified
1681	0	0	0	1	Planctomycetes	Planctomycea	Gemmatales	Isosphaeraceae	unclassified
4	0	0	1	0	Proteobacteria	Alphaproteobacteria	unclassified	unclassified	unclassified
6	1	0	0	0	Proteobacteria	Betaproteobacteria	unclassified	unclassified	unclassified
11	4	2	0	0	Proteobacteria	Alphaproteobacteria	Sphingomonadales	Erythrobacteraceae	Lutibacterium
16	0	0	1	2	Proteobacteria	Alphaproteobacteria	Rhizobiales	Aurantimonadaceae	unclassified
17	0	1	0	0	Proteobacteria	Alphaproteobacteria	Rhizobiales	Phyllobacteriaceae	unclassified
20	0	8	2	8	Proteobacteria	Alphaproteobacteria	Rhodobacterales	Rhodobacteraceae	Rubellimicrobium
28	0	3	1	0	Proteobacteria	Alphaproteobacteria	Sphingomonadales	Sphingomonadaceae	unclassified
34	0	0	1	0	Proteobacteria	Alphaproteobacteria	Rhodospirillales	Rhodospirillaceae	unclassified
35	1	2	0	1	Proteobacteria	Alphaproteobacteria	Sphingomonadales	Sphingomonadaceae	unclassified
39	1	0	0	0	Proteobacteria	Alphaproteobacteria	unclassified	unclassified	unclassified
40	0	2	0	0	Proteobacteria	Alphaproteobacteria	Sphingomonadales	Sphingomonadaceae	unclassified
43	0	0	1	0	Proteobacteria	Alphaproteobacteria	Rhizobiales	unclassified	unclassified
46	0	0	1	0	Proteobacteria	Betaproteobacteria	unclassified	unclassified	unclassified
47	0	0	2	0	Proteobacteria	Alphaproteobacteria	Rhizobiales	unclassified	unclassified
51	0	0	0	1	Proteobacteria	Alphaproteobacteria	Rhodobacterales	Rhodobacteraceae	Rubellimicrobium
52	0	4	0	0	Proteobacteria	Alphaproteobacteria	Rhodobacterales	Rhodobacteraceae	unclassified
54	1	1	0	0	Proteobacteria	Alphaproteobacteria	Rhodospirillales	Acetobacteraceae	Roseomonas
56	0	2	4	0	Proteobacteria	Alphaproteobacteria	Rhizobiales	Methylobacteriaceae	Methylobacterium
58	0	0	0	2	Proteobacteria	Alphaproteobacteria	Rhodobacterales	Rhodobacteraceae	Amaricoccus
60	0	1	0	0	Proteobacteria	Deltaproteobacteria	Syntrophobacterales	Syntrophobacteraceae	unclassified
61	0	0	1	0	Proteobacteria	Alphaproteobacteria	Rhodospirillales	Acetobacteraceae	unclassified
62	0	0	3	0	Proteobacteria	Alphaproteobacteria	Rhizobiales	Bradyrhizobiaceae	unclassified
69	0	1	0	0	Proteobacteria	unclassified	unclassified	unclassified	unclassified
74	0	0	0	1	Proteobacteria	Alphaproteobacteria	Sphingomonadales	Sphingomonadaceae	unclassified
75	1	0	0	0	Proteobacteria	Alphaproteobacteria	Rhodospirillales	Acetobacteraceae	unclassified
83	0	1	0	0	Proteobacteria	Alphaproteobacteria	Rhizobiales	Brucellaceae	Pseudochrobactrum
85	0	1	0	0	Proteobacteria	Gammaproteobacteria	Oceanospirillales	HTCC2188	unclassified
91	1	3	1	2	Proteobacteria	Alphaproteobacteria	Rhodobacterales	Rhodobacteraceae	Rubellimicrobium
93	0	1	0	0	Proteobacteria	Alphaproteobacteria	Rhodospirillales	Acetobacteraceae	unclassified
98	2	1	0	0	Proteobacteria	Alphaproteobacteria	Caulobacterales	Caulobacteraceae	unclassified
103	0	0	1	0	Proteobacteria	Alphaproteobacteria	Rhizobiales	Phyllobacteriaceae	Aminobacter
104	0	0	0	1	Proteobacteria	Alphaproteobacteria	Sphingomonadales	Erythrobacteraceae	unclassified
106	47	4	2	1	Proteobacteria	Betaproteobacteria	Burkholderiales	Comamonadaceae	Comamonas
107	11	0	6	8	Proteobacteria	Gammaproteobacteria	Pseudomonadales	Pseudomonadaceae	Pseudomonas
108	1	0	0	0	Proteobacteria	Alphaproteobacteria	Rhodobacterales	Rhodobacteraceae	Amaricoccus
109	0	0	1	0	Proteobacteria	Alphaproteobacteria	Rhodospirillales	Rhodospirillaceae	unclassified
111	0	1	0	0	Proteobacteria	Alphaproteobacteria	Caulobacterales	Caulobacteraceae	unclassified
113	0	2	0	0	Proteobacteria	Alphaproteobacteria	Rhizobiales	Brucellaceae	unclassified
115	0	13	0	0	Proteobacteria	Gammaproteobacteria	Xanthomonadales	Xanthomonadaceae	Lysobacter
117	2	2	4	10	Proteobacteria	Alphaproteobacteria	Sphingomonadales	Sphingomonadaceae	Sphingomonas
118	0	0	0	1	Proteobacteria	unclassified	unclassified	unclassified	unclassified
119	0	1	0	0	Proteobacteria	Alphaproteobacteria	Rhodobacterales	Rhodobacteraceae	unclassified
120	0	1	0	0	Proteobacteria	Gammaproteobacteria	Xanthomonadales	Xanthomonadaceae	unclassified
121	0	0	7	0	Proteobacteria	Gammaproteobacteria	Pseudomonadales	Moraxellaceae	Moraxella
129	0	2	0	0	Proteobacteria	Gammaproteobacteria	Xanthomonadales	Xanthomonadaceae	unclassified
131	0	2	0	0	Proteobacteria	Gammaproteobacteria	Xanthomonadales	Sinobacteraceae	unclassified
132	1	0	0	0	Proteobacteria	Alphaproteobacteria	Caulobacterales	Caulobacteraceae	Caulobacter
135	0	3	0	0	Proteobacteria	Alphaproteobacteria	Sphingomonadales	Sphingomonadaceae	unclassified
138	0	0	1	0	Proteobacteria	Alphaproteobacteria	Rhizobiales	Hyphomicrobiaceae	Pedomicrobium
144	0	0	2	0	Proteobacteria	Alphaproteobacteria	Rhizobiales	Rhizobiaceae	Kaistia
161	0	0	1	0	Proteobacteria	Alphaproteobacteria	Rickettsiales	Anaplasmataceae	Anaplasma
162	0	0	2	1	Proteobacteria	Alphaproteobacteria	Caulobacterales	Caulobacteraceae	unclassified
172	0	1	0	0	Proteobacteria	Alphaproteobacteria	Rhodospirillales	Rhodospirillaceae	unclassified
175	0	1	0	0	Proteobacteria	Alphaproteobacteria	Rhizobiales	unclassified	unclassified
179	0	5	0	0	Proteobacteria	Alphaproteobacteria	Sphingomonadales	Erythrobacteraceae	unclassified
180	0	1	0	0	Proteobacteria	Betaproteobacteria	Rhodocyclales	Rhodocyclaceae	Dechloromonas
183	4	7	0	1	Proteobacteria	Alphaproteobacteria	Rhizobiales	unclassified	unclassified
184	0	3	1	2	Proteobacteria	Alphaproteobacteria	Rhizobiales	Rhizobiaceae	unclassified
186	0	0	0	1	Proteobacteria	Alphaproteobacteria	Rhizobiales	Xanthobacteraceae	Labrys
187	0	1	0	0	Proteobacteria	Alphaproteobacteria	Sphingomonadales	Sphingomonadaceae	unclassified
191	0	2	0	0	Proteobacteria	Alphaproteobacteria	Caulobacterales	Caulobacteraceae	Brevundimonas
195	0	0	0	1	Proteobacteria	Gammaproteobacteria	Xanthomonadales	Xanthomonadaceae	unclassified
199	0	1	0	0	Proteobacteria	Betaproteobacteria	Burkholderiales	unclassified	unclassified
206	0	14	0	0	Proteobacteria	Betaproteobacteria	Burkholderiales	Burkholderiaceae	unclassified
208	0	0	2	1	Proteobacteria	Betaproteobacteria	Methylophilales	Methylophilaceae	unclassified
213	0	0	0	1	Proteobacteria	Alphaproteobacteria	Rhodospirillales	Acetobacteraceae	Roseomonas
216	0	5	0	2	Proteobacteria	Betaproteobacteria	Burkholderiales	unclassified	unclassified
226	0	0	0	2	Proteobacteria	Alphaproteobacteria	Rhodospirillales	Acetobacteraceae	unclassified
227	0	109	0	0	Proteobacteria	Betaproteobacteria	Rhodocyclales	Rhodocyclaceae	unclassified
228	0	1	0	0	Proteobacteria	Gammaproteobacteria	Xanthomonadales	Xanthomonadaceae	unclassified
229	0	1	0	0	Proteobacteria	Betaproteobacteria	Burkholderiales	Alcaligenaceae	unclassified
233	0	2	0	0	Proteobacteria	Deltaproteobacteria	Myxococcales	unclassified	unclassified
235	1	0	0	0	Proteobacteria	Alphaproteobacteria	Rhodobacterales	Rhodobacteraceae	Amaricoccus
236	0	0	1	0	Proteobacteria	Alphaproteobacteria	Rhizobiales	unclassified	unclassified
238	0	0	1	0	Proteobacteria	Alphaproteobacteria	Rickettsiales	unclassified	unclassified
241	0	0	0	1	Proteobacteria	Alphaproteobacteria	Sphingomonadales	Sphingomonadaceae	unclassified
242	0	1	3	0	Proteobacteria	Deltaproteobacteria	CTD005-82B-02	unclassified	unclassified
245	0	2	0	0	Proteobacteria	Gammaproteobacteria	Xanthomonadales	Xanthomonadaceae	unclassified
246	0	1	0	0	Proteobacteria	Alphaproteobacteria	Sphingomonadales	Erythrobacteraceae	unclassified

Table S5 (part K): OTUs, no. of reads and corresponding taxonomic affiliation of detected bacterial 16S rRNA amplicons by 454 pyrotag sequencing. Please note, the data shown here was either rank normalized or sum normlaized (to 5000 reads per sample) before performing statisical analysis as depicted in the manuscript.

	Samples and no. of reads				taxonomic classification of bacterial 16S rRNA gene amplicons				
OTU	UR	CO	CR8	CR5	phylum	class	order	family	genus
255	0	1	0	0	Proteobacteria	unclassified	unclassified	unclassified	unclassified
256	0	0	1	0	Proteobacteria	Alphaproteobacteria	Rhizobiales	Methylobacteriaceae	Methylobacterium
265	0	2	0	0	Proteobacteria	Betaproteobacteria	Burkholderiales	Alcaligenaceae	unclassified
273	0	0	2	0	Proteobacteria	Gammaproteobacteria	Legionellales	Legionellaceae	Legionella
275	0	2	0	0	Proteobacteria	Alphaproteobacteria	Caulobacterales	Caulobacteraceae	Brevundimonas
277	0	0	0	1	Proteobacteria	Betaproteobacteria	unclassified	unclassified	unclassified
278	0	0	2	0	Proteobacteria	Alphaproteobacteria	Sphingomonadales	Sphingomonadaceae	Sphingomonas
279	0	1	1	1	Proteobacteria	Alphaproteobacteria	Caulobacterales	Caulobacteraceae	unclassified
283	0	2	0	0	Proteobacteria	Alphaproteobacteria	Sphingomonadales	Sphingomonadaceae	Zymomonas
289	0	0	2	0	Proteobacteria	Alphaproteobacteria	Rhodobacterales	Rhodobacteraceae	Rubellimicrobium
291	0	1	0	0	Proteobacteria	Gammaproteobacteria	unclassified	unclassified	unclassified
292	0	0	1	0	Proteobacteria	Gammaproteobacteria	Enterobacteriales	Enterobacteriaceae	unclassified
296	0	1	0	0	Proteobacteria	Alphaproteobacteria	Rhizobiales	Bradyrhizobiaceae	unclassified
297	0	0	1	0	Proteobacteria	Alphaproteobacteria	Rhodospirillales	Acetobacteraceae	unclassified
301	0	1	0	0	Proteobacteria	Gammaproteobacteria	unclassified	unclassified	unclassified
304	0	15	13	12	Proteobacteria	Betaproteobacteria	Burkholderiales	Oxalobacteraceae	unclassified
310	0	1	0	0	Proteobacteria	Alphaproteobacteria	Rhodospirillales	Rhodospirillaceae	unclassified
318	1	5	0	0	Proteobacteria	Gammaproteobacteria	Xanthomonadales	Xanthomonadaceae	Rhodanobacter
320	0	1	0	0	Proteobacteria	Alphaproteobacteria	Sphingomonadales	unclassified	unclassified
323	0	0	0	1	Proteobacteria	Alphaproteobacteria	Sphingomonadales	Sphingomonadaceae	unclassified
327	0	0	0	2	Proteobacteria	Alphaproteobacteria	Rhodospirillales	Acetobacteraceae	unclassified
330	0	1	1	0	Proteobacteria	Gammaproteobacteria	Xanthomonadales	Xanthomonadaceae	Xanthomonas
331	0	1	1	1	Proteobacteria	Gammaproteobacteria	Xanthomonadales	Xanthomonadaceae	Stenotrophomonas
332	0	1	0	1	Proteobacteria	Alphaproteobacteria	Rhodospirillales	Rhodospirillaceae	unclassified
335	3	13	7	30	Proteobacteria	Alphaproteobacteria	Sphingomonadales	Sphingomonadaceae	Novosphingobium
336	0	0	4	0	Proteobacteria	Alphaproteobacteria	Rhodospirillales	Rhodospirillaceae	unclassified
340	0	0	0	2	Proteobacteria	Gammaproteobacteria	Vibrionales	Vibrionaceae	Vibrio
350	2	5	2	1	Proteobacteria	Gammaproteobacteria	Xanthomonadales	Xanthomonadaceae	unclassified
358	0	1	0	0	Proteobacteria	Alphaproteobacteria	Rhodospirillales	Acetobacteraceae	Roseomonas
360	0	1	0	0	Proteobacteria	Gammaproteobacteria	Xanthomonadales	Xanthomonadaceae	Lysobacter
363	0	0	0	2	Proteobacteria	Gammaproteobacteria	Xanthomonadales	Xanthomonadaceae	Stenotrophomonas
364	1	0	0	0	Proteobacteria	Alphaproteobacteria	Sphingomonadales	Sphingomonadaceae	Sphingomonas
369	0	1	0	0	Proteobacteria	Betaproteobacteria	unclassified	unclassified	unclassified
374	0	6	7	6	Proteobacteria	Alphaproteobacteria	Rhodospirillales	Acetobacteraceae	unclassified
375	0	15	0	0	Proteobacteria	Betaproteobacteria	Burkholderiales	Alcaligenaceae	Alcaligenes
376	0	2	0	0	Proteobacteria	Alphaproteobacteria	Rhodobacterales	Rhodobacteraceae	unclassified
377	1	0	0	0	Proteobacteria	Alphaproteobacteria	Rhodobacterales	Rhodobacteraceae	unclassified
379	0	2	3	1	Proteobacteria	Alphaproteobacteria	Rhizobiales	Hyphomicrobiaceae	Devosia
381	1	0	1	1	Proteobacteria	Alphaproteobacteria	Rhodobacterales	Rhodobacteraceae	Amaricoccus
383	2	0	1	0	Proteobacteria	Gammaproteobacteria	Xanthomonadales	Xanthomonadaceae	Pseudoxanthomonas
384	0	2	37	0	Proteobacteria	Betaproteobacteria	Burkholderiales	Oxalobacteraceae	unclassified
385	0	1	0	0	Proteobacteria	Alphaproteobacteria	Rhizobiales	unclassified	unclassified
386	0	1	0	0	Proteobacteria	Betaproteobacteria	Burkholderiales	Oxalobacteraceae	unclassified
392	0	1	0	0	Proteobacteria	Betaproteobacteria	Neisseriales	Neisseriaceae	Kingella
396	0	0	31	0	Proteobacteria	Gammaproteobacteria	Legionellales	Legionellaceae	unclassified
402	0	0	2	0	Proteobacteria	Betaproteobacteria	Burkholderiales	unclassified	unclassified
406	0	0	1	0	Proteobacteria	Alphaproteobacteria	Rhodospirillales	Rhodospirillaceae	unclassified
420	0	1	0	2	Proteobacteria	Alphaproteobacteria	Rhodospirillales	Acetobacteraceae	Roseomonas
423	0	1	1	0	Proteobacteria	Alphaproteobacteria	Rhodospirillales	Acetobacteraceae	unclassified
424	1	1	7	0	Proteobacteria	Gammaproteobacteria	Xanthomonadales	Xanthomonadaceae	Arenimonas
435	0	1	0	0	Proteobacteria	Alphaproteobacteria	Rhizobiales	Bradyrhizobiaceae	unclassified
440	0	1	0	1	Proteobacteria	Alphaproteobacteria	Rhodobacterales	Rhodobacteraceae	Rubellimicrobium
446	0	7	0	0	Proteobacteria	Alphaproteobacteria	Rhodobacterales	Rhodobacteraceae	unclassified
448	0	1	1	0	Proteobacteria	Alphaproteobacteria	Rhizobiales	Bradyrhizobiaceae	unclassified
467	0	2	0	0	Proteobacteria	Alphaproteobacteria	Rhodobacterales	Rhodobacteraceae	Paracoccus
468	0	0	0	1	Proteobacteria	unclassified	unclassified	unclassified	unclassified
469	0	0	0	1	Proteobacteria	Alphaproteobacteria	Sphingomonadales	Sphingomonadaceae	Sphingomonas
479	0	0	1	0	Proteobacteria	Deltaproteobacteria	CTD005-82B-02	unclassified	unclassified
484	0	0	3	0	Proteobacteria	Gammaproteobacteria	Xanthomonadales	Xanthomonadaceae	unclassified
486	0	3	0	0	Proteobacteria	Betaproteobacteria	unclassified	unclassified	unclassified
488	0	2	0	0	Proteobacteria	Gammaproteobacteria	unclassified	unclassified	unclassified
490	0	6	0	5	Proteobacteria	Alphaproteobacteria	Rhizobiales	Methylobacteriaceae	Methylobacterium
491	2	2	0	1	Proteobacteria	Alphaproteobacteria	Rhodobacterales	Rhodobacteraceae	Amaricoccus
494	0	1	0	0	Proteobacteria	Gammaproteobacteria	Enterobacteriales	Enterobacteriaceae	unclassified
495	0	1	0	0	Proteobacteria	Alphaproteobacteria	Sphingomonadales	Sphingomonadaceae	unclassified
497	0	1	0	0	Proteobacteria	Deltaproteobacteria	Myxococcales	Polyangiaceae	unclassified
501	0	0	1	0	Proteobacteria	Gammaproteobacteria	Chromatiales	Sinobacteraceae	unclassified
505	0	0	0	1	Proteobacteria	Deltaproteobacteria	Myxococcales	Polyangiaceae	unclassified
508	0	0	1	1	Proteobacteria	Alphaproteobacteria	Rhizobiales	Methylobacteriaceae	Methylobacterium
509	0	0	3	0	Proteobacteria	Alphaproteobacteria	Sphingomonadales	Sphingomonadaceae	Kaistobacter
511	0	1	3	0	Proteobacteria	Alphaproteobacteria	Rhizobiales	Hyphomicrobiaceae	Rhodoplanes
512	1	0	2	0	Proteobacteria	Alphaproteobacteria	Rhizobiales	Bradyrhizobiaceae	Bosea
522	0	0	1	0	Proteobacteria	Alphaproteobacteria	Rhizobiales	Phyllobacteriaceae	unclassified
523	0	1	0	0	Proteobacteria	Betaproteobacteria	Rhodocyclales	Rhodocyclaceae	Aromatoleum
531	0	2	2	0	Proteobacteria	Alphaproteobacteria	Rickettsiales	Rickettsiaceae	Rickettsia
538	0	1	0	0	Proteobacteria	Deltaproteobacteria	Desulfobacterales	Desulfobulbaceae	unclassified
539	0	11	0	0	Proteobacteria	Alphaproteobacteria	Rhizobiales	Phyllobacteriaceae	unclassified
540	0	1	0	0	Proteobacteria	Betaproteobacteria	Gallionellales	Gallionellaceae	unclassified
544	0	13	4	6	Proteobacteria	Alphaproteobacteria	Sphingomonadales	Sphingomonadaceae	Sphingomonas
549	0	0	4	0	Proteobacteria	Alphaproteobacteria	Rhizobiales	Aurantimonadaceae	unclassified
550	0	1	0	1	Proteobacteria	Alphaproteobacteria	Rhodobacterales	Rhodobacteraceae	Amaricoccus
552	0	0	1	0	Proteobacteria	Alphaproteobacteria	unclassified	unclassified	unclassified
556	1	3	1	0	Proteobacteria	Alphaproteobacteria	Sphingomonadales	Sphingomonadaceae	unclassified
558	0	0	4	0	Proteobacteria	Gammaproteobacteria	Xanthomonadales	Xanthomonadaceae	Lysobacter
561	0	2	0	0	Proteobacteria	Alphaproteobacteria	Rhizobiales	Aurantimonadaceae	unclassified
564	0	1	0	0	Proteobacteria	Deltaproteobacteria	MIZ46	unclassified	unclassified
570	0	1	0	0	Proteobacteria	Deltaproteobacteria	Bdellovibrionales	Bdellovibrionaceae	Bdellovibrio
576	0	1	0	0	Proteobacteria	Alphaproteobacteria	Rhodospirillales	Acetobacteraceae	unclassified
582	0	8	1	0	Proteobacteria	Alphaproteobacteria	Rhizobiales	Hyphomicrobiaceae	Devosia
590	0	0	1	0	Proteobacteria	Gammaproteobacteria	Chromatiales	Sinobacteraceae	unclassified
600	5	6	34	5	Proteobacteria	Alphaproteobacteria	Rhodobacterales	Rhodobacteraceae	Paracoccus
605	0	0	2	2	Proteobacteria	Alphaproteobacteria	Rhizobiales	unclassified	unclassified
607	0	4	0	0	Proteobacteria	Alphaproteobacteria	Sphingomonadales	Sphingomonadaceae	unclassified
608	0	0	0	1	Proteobacteria	Gammaproteobacteria	Pseudomonadales	Moraxellaceae	Psychrobacter
609	0	8	9	11	Proteobacteria	Alphaproteobacteria	Rhodospirillales	Acetobacteraceae	Roseomonas
627	0	0	0	2	Proteobacteria	Gammaproteobacteria	Pseudomonadales	Pseudomonadaceae	Pseudomonas
628	1	0	0	0	Proteobacteria	Betaproteobacteria	Rhodocyclales	unclassified	unclassified
629	1	0	0	0	Proteobacteria	Alphaproteobacteria	Sphingomonadales	Sphingomonadaceae	unclassified
630	0	1	0	0	Proteobacteria	Alphaproteobacteria	Sphingomonadales	Sphingomonadaceae	Kaistobacter
631	0	1	0	0	Proteobacteria	Alphaproteobacteria	Rhodospirillales	Acetobacteraceae	Roseomonas

Table S5 (part L): OTUs, no. of reads and corresponding taxonomic affiliation of detected bacterial 16S rRNA amplicons by 454 pyrotag sequencing. Please note, the data shown here was either rank normalized or sum normlaized (to 5000 reads per sample) before performing statisical analysis as depicted in the manuscript.

	Samples and no. of reads				taxonomic classification of bacterial 16S rRNA gene amplicons				
OTU	UR	CO	CR8	CR5	phylum	class	order	family	genus
633	0	0	2	0	Proteobacteria	Betaproteobacteria	unclassified	unclassified	unclassified
634	0	1	0	0	Proteobacteria	Gammaproteobacteria	Enterobacteriales	Enterobacteriaceae	unclassified
639	0	0	1	0	Proteobacteria	Alphaproteobacteria	Rhizobiales	unclassified	unclassified
642	0	0	1	0	Proteobacteria	Alphaproteobacteria	Rhizobiales	unclassified	unclassified
652	2	10	0	8	Proteobacteria	Alphaproteobacteria	Sphingomonadales	Sphingomonadaceae	Sphingomonas
654	0	1	4	0	Proteobacteria	Alphaproteobacteria	Rhizobiales	unclassified	unclassified
655	0	0	0	1	Proteobacteria	Gammaproteobacteria	Chromatiales	Sinobacteraceae	unclassified
662	0	1	0	0	Proteobacteria	Alphaproteobacteria	Rhodobacterales	Rhodobacteraceae	unclassified
664	0	0	2	2	Proteobacteria	Alphaproteobacteria	Sphingomonadales	Erythrobacteraceae	unclassified
665	0	0	0	1	Proteobacteria	Alphaproteobacteria	Rhodospirillales	Rhodospirillaceae	unclassified
666	0	5	0	0	Proteobacteria	Betaproteobacteria	Burkholderiales	Burkholderiaceae	unclassified
668	0	0	0	3	Proteobacteria	Alphaproteobacteria	Rhodospirillales	Acetobacteraceae	unclassified
670	0	1	1	0	Proteobacteria	Alphaproteobacteria	Sphingomonadales	Sphingomonadaceae	Sphingomonas
672	2	2	13	1	Proteobacteria	Alphaproteobacteria	Rhodospirillales	Rhodospirillaceae	Skermanella
675	1	0	0	0	Proteobacteria	Betaproteobacteria	unclassified	unclassified	unclassified
683	0	0	0	1	Proteobacteria	Gammaproteobacteria	unclassified	unclassified	unclassified
687	1	0	0	0	Proteobacteria	Gammaproteobacteria	Alteromonadales	Chromatiaceae	Rheinheimera
690	0	2	0	0	Proteobacteria	Alphaproteobacteria	Rhizobiales	Hyphomicrobiaceae	Rhodoplanes
691	0	1	0	0	Proteobacteria	Gammaproteobacteria	Pseudomonadales	Pseudomonadaceae	Pseudomonas
692	0	0	1	0	Proteobacteria	Alphaproteobacteria	Rhizobiales	Hyphomicrobiaceae	Rhodoplanes
695	0	2	0	0	Proteobacteria	Deltaproteobacteria	Myxococcales	unclassified	unclassified
697	0	1	0	0	Proteobacteria	Alphaproteobacteria	Sphingomonadales	Sphingomonadaceae	unclassified
698	1	0	0	0	Proteobacteria	Gammaproteobacteria	Enterobacteriales	Enterobacteriaceae	Morganella
707	0	0	0	2	Proteobacteria	Alphaproteobacteria	Rhodobacterales	Rhodobacteraceae	Rhodobacter
709	0	1	0	0	Proteobacteria	Alphaproteobacteria	Rhodobacterales	Rhodobacteraceae	Amaricoccus
715	0	0	0	1	Proteobacteria	Alphaproteobacteria	Rhodospirillales	Acetobacteraceae	Roseomonas
716	1	0	0	1	Proteobacteria	Gammaproteobacteria	Xanthomonadales	Xanthomonadaceae	Stenotrophomonas
720	4	4	0	0	Proteobacteria	Alphaproteobacteria	Rhodospirillales	Acetobacteraceae	unclassified
721	1	0	0	0	Proteobacteria	Alphaproteobacteria	Sphingomonadales	Erythrobacteraceae	unclassified
722	0	1	0	0	Proteobacteria	Betaproteobacteria	Burkholderiales	Comamonadaceae	unclassified
727	0	2	0	0	Proteobacteria	Alphaproteobacteria	Rhizobiales	Hyphomicrobiaceae	Hyphomicrobium
730	0	2	0	0	Proteobacteria	Alphaproteobacteria	Rhizobiales	unclassified	unclassified
736	0	0	0	12	Proteobacteria	Gammaproteobacteria	Enterobacteriales	Enterobacteriaceae	Buchnera
738	0	2	0	1	Proteobacteria	Betaproteobacteria	Burkholderiales	Alcaligenaceae	unclassified
740	0	1	0	0	Proteobacteria	Gammaproteobacteria	Oceanospirillales	Alteromonadaceae	Cellvibrio
742	10	1	1	1	Proteobacteria	Gammaproteobacteria	Xanthomonadales	Xanthomonadaceae	Stenotrophomonas
745	0	1	0	0	Proteobacteria	Alphaproteobacteria	Rhodobacterales	Rhodobacteraceae	unclassified
749	0	1	0	1	Proteobacteria	Alphaproteobacteria	Rhizobiales	Phyllobacteriaceae	unclassified
753	0	0	1	0	Proteobacteria	Alphaproteobacteria	Rhizobiales	Phyllobacteriaceae	Mesorhizobium
759	0	1	0	0	Proteobacteria	Alphaproteobacteria	Rhodospirillales	unclassified	unclassified
760	0	0	1	0	Proteobacteria	Deltaproteobacteria	Myxococcales	unclassified	unclassified
762	0	0	5	2	Proteobacteria	Alphaproteobacteria	Rhodobacterales	Rhodobacteraceae	Amaricoccus
765	0	1	0	1	Proteobacteria	Alphaproteobacteria	Rhodobacterales	Rhodobacteraceae	unclassified
770	0	4	0	0	Proteobacteria	Deltaproteobacteria	Myxococcales	Haliangiaceae	unclassified
773	0	1	0	0	Proteobacteria	Deltaproteobacteria	Myxococcales	unclassified	unclassified
776	0	1	2	0	Proteobacteria	Gammaproteobacteria	Chromatiales	Sinobacteraceae	unclassified
779	0	1	0	0	Proteobacteria	Betaproteobacteria	Burkholderiales	Oxalobacteraceae	unclassified
780	0	7	2	0	Proteobacteria	Gammaproteobacteria	Pasteurellales	Pasteurellaceae	Haemophilus
782	6	5	3	4	Proteobacteria	Alphaproteobacteria	Caulobacterales	Caulobacteraceae	Brevundimonas
784	0	0	0	1	Proteobacteria	Alphaproteobacteria	Rhizobiales	Beijerinckiaceae	unclassified
787	1	11	2	1	Proteobacteria	Gammaproteobacteria	Xanthomonadales	Xanthomonadaceae	unclassified
790	0	4	1	0	Proteobacteria	Alphaproteobacteria	Sphingomonadales	Sphingomonadaceae	Sphingomonas
794	0	0	1	0	Proteobacteria	Alphaproteobacteria	Rhizobiales	Phyllobacteriaceae	unclassified
795	4	4	2	1	Proteobacteria	Gammaproteobacteria	Enterobacteriales	Enterobacteriaceae	Escherichia
804	0	2	5	0	Proteobacteria	Alphaproteobacteria	Sphingomonadales	Sphingomonadaceae	unclassified
811	1	0	0	0	Proteobacteria	Alphaproteobacteria	Sphingomonadales	unclassified	unclassified
812	3	1	7	0	Proteobacteria	Alphaproteobacteria	Rhizobiales	Phyllobacteriaceae	Mesorhizobium
816	0	0	0	1	Proteobacteria	Gammaproteobacteria	Xanthomonadales	Xanthomonadaceae	Ignatzschineria
819	0	3	1	6	Proteobacteria	Alphaproteobacteria	Rhizobiales	unclassified	unclassified
822	0	1	0	0	Proteobacteria	Alphaproteobacteria	Rhizobiales	Hyphomicrobiaceae	Devosia
823	0	0	2	0	Proteobacteria	Gammaproteobacteria	Xanthomonadales	Xanthomonadaceae	unclassified
825	1	3	0	2	Proteobacteria	Betaproteobacteria	Burkholderiales	unclassified	unclassified
826	0	1	1	0	Proteobacteria	Gammaproteobacteria	Xanthomonadales	Sinobacteraceae	unclassified
831	0	0	0	1	Proteobacteria	Alphaproteobacteria	Rhodobacterales	Rhodobacteraceae	Amaricoccus
832	0	0	1	0	Proteobacteria	Alphaproteobacteria	Rhizobiales	Methylocystaceae	Methylopila
833	0	1	0	0	Proteobacteria	Betaproteobacteria	Burkholderiales	Oxalobacteraceae	unclassified
838	0	1	0	0	Proteobacteria	Alphaproteobacteria	Caulobacterales	Caulobacteraceae	unclassified
842	0	6	1	3	Proteobacteria	Alphaproteobacteria	Sphingomonadales	Sphingomonadaceae	unclassified
849	0	0	2	0	Proteobacteria	Alphaproteobacteria	Rhizobiales	unclassified	unclassified
850	0	0	1	0	Proteobacteria	Alphaproteobacteria	Rhodospirillales	Rhodospirillaceae	unclassified
851	0	6	7	0	Proteobacteria	Alphaproteobacteria	Rhizobiales	Rhizobiaceae	Rhizobium
852	0	0	2	0	Proteobacteria	Gammaproteobacteria	Alteromonadales	Pseudoalteromonadaceae	Pseudoalteromonas
853	0	0	1	0	Proteobacteria	Deltaproteobacteria	Myxococcales	unclassified	unclassified
854	0	1	1	0	Proteobacteria	Gammaproteobacteria	Pseudomonadales	Pseudomonadaceae	Pseudomonas
858	0	1	5	0	Proteobacteria	Alphaproteobacteria	Caulobacterales	Caulobacteraceae	unclassified
866	1	0	0	0	Proteobacteria	Betaproteobacteria	unclassified	unclassified	unclassified
869	0	1	0	0	Proteobacteria	Betaproteobacteria	Burkholderiales	Comamonadaceae	unclassified
870	0	1	0	0	Proteobacteria	Alphaproteobacteria	unclassified	unclassified	unclassified
871	0	1	0	0	Proteobacteria	Alphaproteobacteria	Rhizobiales	Phyllobacteriaceae	unclassified
873	0	4	5	4	Proteobacteria	Gammaproteobacteria	Enterobacteriales	Enterobacteriaceae	unclassified
874	1	0	0	0	Proteobacteria	Alphaproteobacteria	Rhizobiales	Methylobacteriaceae	Methylobacterium
877	0	1	0	0	Proteobacteria	Alphaproteobacteria	Sphingomonadales	Sphingomonadaceae	unclassified
878	0	1	0	0	Proteobacteria	Deltaproteobacteria	Syntrophobacterales	Syntrophobacteraceae	unclassified
880	0	3	4	3	Proteobacteria	Alphaproteobacteria	Sphingomonadales	Sphingomonadaceae	Sphingomonas
881	1	1	1	0	Proteobacteria	Betaproteobacteria	Burkholderiales	Oxalobacteraceae	Massilia
883	0	2	1	0	Proteobacteria	Alphaproteobacteria	unclassified	unclassified	unclassified
888	0	2	1	0	Proteobacteria	Alphaproteobacteria	Sphingomonadales	Sphingomonadaceae	unclassified
889	0	0	3	1	Proteobacteria	Alphaproteobacteria	Rhodospirillales	Rhodospirillaceae	unclassified
890	0	1	0	0	Proteobacteria	Deltaproteobacteria	Myxococcales	Nannocystaceae	Plesiocystis
892	0	2	0	0	Proteobacteria	Alphaproteobacteria	Caulobacterales	Caulobacteraceae	Phenylobacterium
895	0	0	1	0	Proteobacteria	Alphaproteobacteria	Sphingomonadales	Sphingomonadaceae	Kaistobacter
897	2	0	0	0	Proteobacteria	Alphaproteobacteria	Caulobacterales	Caulobacteraceae	Brevundimonas
898	12	11	4	7	Proteobacteria	Alphaproteobacteria	Rhizobiales	Rhizobiaceae	Agrobacterium
899	2	0	0	0	Proteobacteria	Alphaproteobacteria	Sphingomonadales	Sphingomonadaceae	unclassified
904	0	1	0	0	Proteobacteria	Betaproteobacteria	Nitrosomonadales	Nitrosomonadaceae	Nitrosomonas
909	0	0	3	0	Proteobacteria	Alphaproteobacteria	Rickettsiales	Holosporaceae	unclassified
911	0	28	0	0	Proteobacteria	Gammaproteobacteria	Xanthomonadales	Xanthomonadaceae	unclassified
913	0	0	1	0	Proteobacteria	Alphaproteobacteria	Rhodospirillales	Acetobacteraceae	Roseomonas
914	3	0	0	0	Proteobacteria	Alphaproteobacteria	Rhodospirillales	Rhodospirillaceae	unclassified
915	0	7	0	0	Proteobacteria	Alphaproteobacteria	Sphingomonadales	Sphingomonadaceae	unclassified

Table S5 (part M): OTUs, no. of reads and corresponding taxonomic affiliation of detected bacterial 16S rRNA amplicons by 454 pyrotag sequencing. Please note, the data shown here was either rank normalized or sum normlaized (to 5000 reads per sample) before performing statisical analysis as depicted in the manuscript.

	Samples and no. of reads				taxonomic classification of bacterial 16S rRNA gene amplicons				
OTU	UR	CO	CR8	CR5	phylum	class	order	family	genus
917	0	3	4	0	Proteobacteria	Alphaproteobacteria	Sphingomonadales	Sphingomonadaceae	Sphingomonas
919	1	0	2	0	Proteobacteria	Alphaproteobacteria	Rhodospirillales	Rhodospirillaceae	unclassified
923	0	0	0	1	Proteobacteria	Alphaproteobacteria	Sphingomonadales	Sphingomonadaceae	Sphingomonas
924	0	0	0	3	Proteobacteria	Alphaproteobacteria	Sphingomonadales	Sphingomonadaceae	unclassified
926	0	0	0	1	Proteobacteria	Alphaproteobacteria	Rhizobiales	Phyllobacteriaceae	Mesorhizobium
930	0	1	0	3	Proteobacteria	Alphaproteobacteria	Sphingomonadales	unclassified	unclassified
945	0	0	2	0	Proteobacteria	Gammaproteobacteria	Legionellales	Legionellaceae	Legionella
950	2	0	0	0	Proteobacteria	Alphaproteobacteria	Sphingomonadales	Sphingomonadaceae	Sphingobium
951	0	0	0	3	Proteobacteria	Alphaproteobacteria	Caulobacterales	Caulobacteraceae	unclassified
952	1	0	0	0	Proteobacteria	Deltaproteobacteria	Myxococcales	unclassified	unclassified
954	0	0	0	1	Proteobacteria	Alphaproteobacteria	Rhizobiales	Bradyrhizobiaceae	unclassified
956	0	1	0	0	Proteobacteria	Gammaproteobacteria	Xanthomonadales	Xanthomonadaceae	Lysobacter
958	0	1	0	0	Proteobacteria	Alphaproteobacteria	Sphingomonadales	Sphingomonadaceae	Sphingobium
961	0	1	0	0	Proteobacteria	Alphaproteobacteria	Rhodobacterales	Rhodobacteraceae	Rubellimicrobium
965	0	8	0	0	Proteobacteria	Alphaproteobacteria	Rhizobiales	Hyphomicrobiaceae	Devosia
966	0	1	0	0	Proteobacteria	Gammaproteobacteria	Oceanospirillales	OM60	unclassified
967	0	1	0	0	Proteobacteria	Alphaproteobacteria	Rhizobiales	Methylobacteriaceae	Methylobacterium
974	1	1	0	0	Proteobacteria	Epsilonproteobacteria	Campylobacterales	Campylobacteraceae	Campylobacter
975	0	4	7	1	Proteobacteria	Alphaproteobacteria	Rhizobiales	Hyphomicrobiaceae	Devosia
979	1	5	2	1	Proteobacteria	Alphaproteobacteria	Sphingomonadales	Sphingomonadaceae	unclassified
982	0	1	0	0	Proteobacteria	Alphaproteobacteria	Rhizobiales	Bradyrhizobiaceae	unclassified
983	2	0	0	0	Proteobacteria	Alphaproteobacteria	Sphingomonadales	Sphingomonadaceae	unclassified
988	0	1	1	0	Proteobacteria	Alphaproteobacteria	Rhodobacterales	Rhodobacteraceae	unclassified
989	0	0	1	1	Proteobacteria	Alphaproteobacteria	Caulobacterales	Caulobacteraceae	unclassified
990	0	1	0	0	Proteobacteria	Alphaproteobacteria	Rhizobiales	unclassified	unclassified
1004	0	1	0	1	Proteobacteria	Alphaproteobacteria	unclassified	unclassified	unclassified
1005	0	3	0	0	Proteobacteria	Alphaproteobacteria	Sphingomonadales	Sphingomonadaceae	Sphingomonas
1006	0	0	4	1	Proteobacteria	Alphaproteobacteria	Rhizobiales	Phyllobacteriaceae	unclassified
1010	0	0	1	0	Proteobacteria	Gammaproteobacteria	Pseudomonadales	Moraxellaceae	Psychrobacter
1012	0	1	0	0	Proteobacteria	Alphaproteobacteria	Caulobacterales	Caulobacteraceae	unclassified
1016	0	16	0	0	Proteobacteria	Alphaproteobacteria	Rhodobacterales	Rhodobacteraceae	Paracoccus
1017	0	0	1	0	Proteobacteria	Alphaproteobacteria	Rhodospirillales	Rhodospirillaceae	Azospirillum
1018	0	0	3	2	Proteobacteria	Alphaproteobacteria	Rhodobacterales	Rhodobacteraceae	Rubellimicrobium
1023	0	0	4	0	Proteobacteria	Betaproteobacteria	unclassified	unclassified	unclassified
1025	0	0	1	0	Proteobacteria	Deltaproteobacteria	Myxococcales	Haliangiaceae	unclassified
1035	0	0	1	0	Proteobacteria	Alphaproteobacteria	Rhodospirillales	Acetobacteraceae	Roseomonas
1037	0	3	0	0	Proteobacteria	Gammaproteobacteria	Xanthomonadales	Xanthomonadaceae	unclassified
1040	0	1	0	0	Proteobacteria	Alphaproteobacteria	Rhizobiales	unclassified	unclassified
1042	0	1	0	0	Proteobacteria	Alphaproteobacteria	unclassified	unclassified	unclassified
1043	0	0	0	3	Proteobacteria	Alphaproteobacteria	Sphingomonadales	Sphingomonadaceae	Sphingomonas
1044	0	0	0	1	Proteobacteria	Gammaproteobacteria	Xanthomonadales	Xanthomonadaceae	unclassified
1046	0	0	1	0	Proteobacteria	Alphaproteobacteria	Rhodospirillales	Acetobacteraceae	unclassified
1047	0	1	0	0	Proteobacteria	Alphaproteobacteria	Rhizobiales	Hyphomicrobiaceae	unclassified
1048	0	0	0	1	Proteobacteria	Alphaproteobacteria	Rhodobacterales	Rhodobacteraceae	Rubellimicrobium
1052	0	1	0	0	Proteobacteria	Alphaproteobacteria	Rhizobiales	Hyphomicrobiaceae	Pedomicrobium
1055	0	1	1	1	Proteobacteria	Alphaproteobacteria	Rhizobiales	Bradyrhizobiaceae	Rhodopseudomonas
1056	0	6	0	0	Proteobacteria	Betaproteobacteria	Burkholderiales	unclassified	unclassified
1059	0	1	0	0	Proteobacteria	Alphaproteobacteria	Rhodospirillales	Acetobacteraceae	unclassified
1061	0	2	0	0	Proteobacteria	Alphaproteobacteria	Rhizobiales	Phyllobacteriaceae	unclassified
1063	1	1	3	1	Proteobacteria	Alphaproteobacteria	Rhizobiales	Methylobacteriaceae	Methylobacterium
1065	0	16	0	0	Proteobacteria	Gammaproteobacteria	Oceanospirillales	Alteromonadaceae	Marinobacter
1066	0	1	0	0	Proteobacteria	Alphaproteobacteria	Rhizobiales	unclassified	unclassified
1067	1	0	4	0	Proteobacteria	Alphaproteobacteria	Rhodospirillales	Rhodospirillaceae	unclassified
1075	0	0	0	1	Proteobacteria	Gammaproteobacteria	Xanthomonadales	Xanthomonadaceae	Xanthomonas
1082	0	0	0	1	Proteobacteria	Alphaproteobacteria	Rhodospirillales	Acetobacteraceae	unclassified
1086	0	1	0	1	Proteobacteria	Alphaproteobacteria	Rhizobiales	Methylobacteriaceae	Methylobacterium
1087	0	2	1	1	Proteobacteria	Alphaproteobacteria	Rhodobacterales	Rhodobacteraceae	unclassified
1092	0	0	0	1	Proteobacteria	Betaproteobacteria	Burkholderiales	Oxalobacteraceae	Massilia
1093	1	13	9	2	Proteobacteria	Alphaproteobacteria	Rhodospirillales	Acetobacteraceae	unclassified
1097	1	0	0	0	Proteobacteria	Alphaproteobacteria	Caulobacterales	Caulobacteraceae	Brevundimonas
1100	1	4	4	1	Proteobacteria	Betaproteobacteria	Burkholderiales	unclassified	unclassified
1104	3	0	0	0	Proteobacteria	Alphaproteobacteria	Sphingomonadales	Sphingomonadaceae	Kaistobacter
1110	3	2	0	1	Proteobacteria	Alphaproteobacteria	Caulobacterales	Caulobacteraceae	unclassified
1114	0	1	0	0	Proteobacteria	Alphaproteobacteria	Rhodobacterales	Rhodobacteraceae	Rubellimicrobium
1122	0	5	1	0	Proteobacteria	Alphaproteobacteria	Rhodobacterales	Rhodobacteraceae	Amaricoccus
1124	0	0	6	0	Proteobacteria	Alphaproteobacteria	Rhodospirillales	Rhodospirillaceae	unclassified
1125	0	0	5	0	Proteobacteria	Gammaproteobacteria	Enterobacteriales	Enterobacteriaceae	Pantoea
1129	0	4	0	1	Proteobacteria	Alphaproteobacteria	Rhizobiales	unclassified	unclassified
1130	0	0	1	0	Proteobacteria	Alphaproteobacteria	Rhodospirillales	Acetobacteraceae	unclassified
1134	0	1	5	0	Proteobacteria	Alphaproteobacteria	Rhodospirillales	Acetobacteraceae	unclassified
1136	0	2	0	0	Proteobacteria	Gammaproteobacteria	Xanthomonadales	Xanthomonadaceae	unclassified
1143	0	1	0	0	Proteobacteria	Deltaproteobacteria	CTD005-82B-02	unclassified	unclassified
1147	0	0	1	0	Proteobacteria	Alphaproteobacteria	Caulobacterales	Caulobacteraceae	unclassified
1151	0	1	0	0	Proteobacteria	Alphaproteobacteria	Rhizobiales	Phyllobacteriaceae	unclassified
1153	0	0	1	0	Proteobacteria	Alphaproteobacteria	Rhizobiales	Methylocystaceae	unclassified
1155	2	4	0	1	Proteobacteria	Alphaproteobacteria	Sphingomonadales	Erythrobacteraceae	unclassified
1159	0	5	0	0	Proteobacteria	Alphaproteobacteria	Sphingomonadales	unclassified	unclassified
1160	0	3	0	1	Proteobacteria	Alphaproteobacteria	Caulobacterales	Caulobacteraceae	unclassified
1162	1	0	0	1	Proteobacteria	Alphaproteobacteria	Sphingomonadales	unclassified	unclassified
1170	0	3	0	0	Proteobacteria	Alphaproteobacteria	Rhodobacterales	Rhodobacteraceae	unclassified
1172	0	3	0	0	Proteobacteria	Alphaproteobacteria	Sphingomonadales	Erythrobacteraceae	Lutibacterium
1176	44	0	0	1	Proteobacteria	Gammaproteobacteria	Xanthomonadales	Xanthomonadaceae	Stenotrophomonas
1177	1	0	0	0	Proteobacteria	Betaproteobacteria	Burkholderiales	unclassified	unclassified
1185	0	2	0	0	Proteobacteria	Betaproteobacteria	Burkholderiales	Comamonadaceae	Hydrogenophaga
1186	0	0	0	1	Proteobacteria	Alphaproteobacteria	Rhizobiales	Bradyrhizobiaceae	unclassified
1187	0	1	0	0	Proteobacteria	Alphaproteobacteria	Sphingomonadales	Sphingomonadaceae	Zymomonas
1189	0	3	0	0	Proteobacteria	Alphaproteobacteria	Sphingomonadales	Sphingomonadaceae	unclassified
1194	0	31	0	0	Proteobacteria	Betaproteobacteria	Nitrosomonadales	Nitrosomonadaceae	unclassified
1199	0	0	1	0	Proteobacteria	Alphaproteobacteria	Rhodospirillales	Acetobacteraceae	unclassified
1201	0	5	0	0	Proteobacteria	Alphaproteobacteria	Rhizobiales	Aurantimonadaceae	Aurantimonas
1206	0	3	5	0	Proteobacteria	Gammaproteobacteria	Xanthomonadales	Xanthomonadaceae	Lysobacter
1208	8	40	9	14	Proteobacteria	Alphaproteobacteria	Rhodobacterales	Rhodobacteraceae	Rubellimicrobium
1212	0	0	0	1	Proteobacteria	Gammaproteobacteria	Xanthomonadales	Xanthomonadaceae	unclassified
1214	1	0	1	0	Proteobacteria	Alphaproteobacteria	Rhizobiales	Beijerinckiaceae	unclassified
1226	0	1	0	0	Proteobacteria	Alphaproteobacteria	Sphingomonadales	Sphingomonadaceae	Sphingomonas
1229	0	0	1	0	Proteobacteria	Alphaproteobacteria	Rhizobiales	unclassified	unclassified
1232	0	0	3	0	Proteobacteria	Alphaproteobacteria	Rhodospirillales	Rhodospirillaceae	unclassified
1238	0	0	1	0	Proteobacteria	Alphaproteobacteria	Sphingomonadales	Sphingomonadaceae	unclassified
1248	0	1	9	0	Proteobacteria	Deltaproteobacteria	Myxococcales	Nannocystaceae	Nannocystis
1251	1	3	2	6	Proteobacteria	Betaproteobacteria	Burkholderiales	Comamonadaceae	Delftia

Table S5 (part N): OTUs, no. of reads and corresponding taxonomic affiliation of detected bacterial 16S rRNA amplicons by 454 pyrotag sequencing. Please note, the data shown here was either rank normalized or sum normlaized (to 5000 reads per sample) before performing statisical analysis as depicted in the manuscript.

	Samples and no. of reads				taxonomic classification of bacterial 16S rRNA gene amplicons				
OTU	UR	CO	CR8	CR5	phylum	class	order	family	genus
1254	0	1	0	0	Proteobacteria	Betaproteobacteria	Burkholderiales	Methylibiaceae	Methylibium
1260	3	3	8	3	Proteobacteria	Gammaproteobacteria	Pseudomonadales	Pseudomonadaceae	Pseudomonas
1261	1	1	2	0	Proteobacteria	Gammaproteobacteria	Pseudomonadales	Pseudomonadaceae	Pseudomonas
1263	0	3	0	0	Proteobacteria	Alphaproteobacteria	Rhizobiales	unclassified	unclassified
1266	0	0	0	1	Proteobacteria	Alphaproteobacteria	Rhodospirillales	Rhodospirillaceae	unclassified
1267	0	1	0	0	Proteobacteria	Alphaproteobacteria	Rickettsiales	Holosporaceae	unclassified
1268	0	0	1	4	Proteobacteria	Alphaproteobacteria	Rhizobiales	Hyphomicrobiaceae	Rhodoplanes
1269	0	4	0	2	Proteobacteria	Alphaproteobacteria	Rhodobacterales	Rhodobacteraceae	Rubellimicrobium
1271	0	1	0	0	Proteobacteria	Betaproteobacteria	Burkholderiales	Comamonadaceae	unclassified
1277	0	1	0	0	Proteobacteria	Alphaproteobacteria	Rhizobiales	unclassified	unclassified
1279	0	0	1	0	Proteobacteria	Alphaproteobacteria	Rhodospirillales	Acetobacteraceae	Roseomonas
1280	1	5	3	4	Proteobacteria	Gammaproteobacteria	Xanthomonadales	Xanthomonadaceae	Thermomonas
1282	2	0	0	0	Proteobacteria	Gammaproteobacteria	Xanthomonadales	Xanthomonadaceae	Pseudoxanthomonas
1283	0	0	2	0	Proteobacteria	Betaproteobacteria	Neisseriales	Neisseriaceae	unclassified
1285	0	1	0	0	Proteobacteria	Alphaproteobacteria	Rhodobacterales	Rhodobacteraceae	unclassified
1292	0	8	8	6	Proteobacteria	Gammaproteobacteria	Enterobacteriales	Enterobacteriaceae	Pantoea
1295	0	1	0	0	Proteobacteria	Alphaproteobacteria	Rhodobacterales	Rhodobacteraceae	unclassified
1301	0	1	0	0	Proteobacteria	Alphaproteobacteria	Rhizobiales	Hyphomicrobiaceae	Devosia
1304	0	32	0	0	Proteobacteria	Alphaproteobacteria	Rhodobacterales	Rhodobacteraceae	unclassified
1307	0	0	1	0	Proteobacteria	Alphaproteobacteria	Sphingomonadales	Sphingomonadaceae	Kaistobacter
1309	0	0	1	0	Proteobacteria	unclassified	unclassified	unclassified	unclassified
1312	0	1	0	0	Proteobacteria	Alphaproteobacteria	Rhizobiales	Hyphomicrobiaceae	Devosia
1313	0	1	0	0	Proteobacteria	Betaproteobacteria	Burkholderiales	Comamonadaceae	unclassified
1314	0	1	0	0	Proteobacteria	Alphaproteobacteria	Rhodospirillales	Acetobacteraceae	unclassified
1317	0	0	0	2	Proteobacteria	Alphaproteobacteria	Caulobacterales	Caulobacteraceae	unclassified
1319	0	0	0	1	Proteobacteria	Alphaproteobacteria	Rhizobiales	Rhodobiaceae	unclassified
1334	0	0	1	0	Proteobacteria	Alphaproteobacteria	Rhodospirillales	Rhodospirillaceae	unclassified
1338	0	1	0	0	Proteobacteria	Alphaproteobacteria	Rhizobiales	Aurantimonadaceae	unclassified
1343	1	1	0	3	Proteobacteria	Alphaproteobacteria	Rhodobacterales	Rhodobacteraceae	Rubellimicrobium
1344	0	0	2	0	Proteobacteria	Alphaproteobacteria	Sphingomonadales	unclassified	unclassified
1346	2	0	0	0	Proteobacteria	Alphaproteobacteria	Rhodobacterales	Rhodobacteraceae	Amaricoccus
1347	0	0	3	0	Proteobacteria	Alphaproteobacteria	Rhodospirillales	Acetobacteraceae	Roseomonas
1348	0	0	0	1	Proteobacteria	Gammaproteobacteria	unclassified	unclassified	unclassified
1353	1	2	0	0	Proteobacteria	Alphaproteobacteria	Rhizobiales	Methylobacteriaceae	Methylobacterium
1362	3	0	0	0	Proteobacteria	Gammaproteobacteria	Pseudomonadales	Pseudomonadaceae	Pseudomonas
1365	1	3	3	2	Proteobacteria	Alphaproteobacteria	Caulobacterales	Caulobacteraceae	Phenylobacterium
1369	0	0	1	1	Proteobacteria	Alphaproteobacteria	Rhizobiales	Phyllobacteriaceae	Mesorhizobium
1377	0	0	1	0	Proteobacteria	Deltaproteobacteria	Myxococcales	Cystobacteraceae	unclassified
1385	0	0	1	0	Proteobacteria	Alphaproteobacteria	Rhodospirillales	Rhodospirillaceae	Magnetospirillum
1387	0	0	1	0	Proteobacteria	Alphaproteobacteria	Sphingomonadales	Sphingomonadaceae	Sphingomonas
1390	0	0	1	0	Proteobacteria	Gammaproteobacteria	Xanthomonadales	Xanthomonadaceae	Pseudoxanthomonas
1395	1	0	0	0	Proteobacteria	Gammaproteobacteria	Pseudomonadales	Moraxellaceae	Acinetobacter
1397	0	1	0	0	Proteobacteria	Gammaproteobacteria	Pseudomonadales	Moraxellaceae	Alkanindiges
1398	1	0	0	0	Proteobacteria	Alphaproteobacteria	Rhizobiales	Rhizobiaceae	unclassified
1403	0	5	0	1	Proteobacteria	Alphaproteobacteria	Rhodobacterales	Rhodobacteraceae	Rubellimicrobium
1409	0	1	0	0	Proteobacteria	Deltaproteobacteria	Myxococcales	unclassified	unclassified
1413	0	3	0	0	Proteobacteria	Alphaproteobacteria	Rhizobiales	Beijerinckiaceae	unclassified
1419	0	1	1	0	Proteobacteria	Alphaproteobacteria	Rhodobacterales	Rhodobacteraceae	Rubellimicrobium
1426	0	0	1	0	Proteobacteria	Alphaproteobacteria	Rhodobacterales	Rhodobacteraceae	Rubellimicrobium
1428	0	23	0	0	Proteobacteria	Betaproteobacteria	Burkholderiales	Comamonadaceae	Simplicispira
1430	0	4	0	0	Proteobacteria	Alphaproteobacteria	Rhodospirillales	Rhodospirillaceae	unclassified
1431	0	1	0	0	Proteobacteria	Alphaproteobacteria	Sphingomonadales	unclassified	unclassified
1439	2	0	2	1	Proteobacteria	Gammaproteobacteria	Xanthomonadales	Xanthomonadaceae	unclassified
1443	0	1	0	3	Proteobacteria	Alphaproteobacteria	Rhizobiales	Hyphomicrobiaceae	Devosia
1447	0	1	1	0	Proteobacteria	Alphaproteobacteria	Rhodospirillales	unclassified	unclassified
1448	1	6	0	4	Proteobacteria	Alphaproteobacteria	Caulobacterales	Caulobacteraceae	unclassified
1455	1	18	2	1	Proteobacteria	Gammaproteobacteria	Xanthomonadales	Xanthomonadaceae	Thermomonas
1458	0	0	0	1	Proteobacteria	Betaproteobacteria	Rhodocyclales	unclassified	unclassified
1459	0	1	0	0	Proteobacteria	Alphaproteobacteria	Rhizobiales	Hyphomicrobiaceae	unclassified
1460	0	0	1	0	Proteobacteria	Deltaproteobacteria	Myxococcales	unclassified	unclassified
1463	0	1	0	0	Proteobacteria	Betaproteobacteria	Neisseriales	Neisseriaceae	Chromobacterium
1464	0	3	0	0	Proteobacteria	Alphaproteobacteria	Rhodobacterales	Rhodobacteraceae	unclassified
1465	0	1	0	0	Proteobacteria	Betaproteobacteria	Neisseriales	Neisseriaceae	Stenoxybacter
1466	0	0	0	1	Proteobacteria	Epsilonproteobacteria	Campylobacterales	Campylobacteraceae	Campylobacter
1471	0	0	1	0	Proteobacteria	Alphaproteobacteria	Sphingomonadales	unclassified	unclassified
1472	0	1	2	0	Proteobacteria	Gammaproteobacteria	Xanthomonadales	Xanthomonadaceae	Dokdonella
1477	0	1	0	0	Proteobacteria	Alphaproteobacteria	Sphingomonadales	Erythrobacteraceae	unclassified
1479	8	3	2	2	Proteobacteria	Gammaproteobacteria	Pseudomonadales	Moraxellaceae	Acinetobacter
1484	0	1	7	0	Proteobacteria	Alphaproteobacteria	Rhizobiales	unclassified	unclassified
1501	1	1	0	0	Proteobacteria	Alphaproteobacteria	Rhodobacterales	Rhodobacteraceae	Rubellimicrobium
1504	0	1	0	0	Proteobacteria	Gammaproteobacteria	Legionellales	Legionellaceae	unclassified
1505	0	0	4	0	Proteobacteria	Alphaproteobacteria	Rhizobiales	Hyphomicrobiaceae	Rhodoplanes
1509	0	1	0	1	Proteobacteria	Alphaproteobacteria	Rhizobiales	Phyllobacteriaceae	Mesorhizobium
1510	0	3	13	3	Proteobacteria	Alphaproteobacteria	Rhodospirillales	Acetobacteraceae	unclassified
1513	0	3	0	0	Proteobacteria	Alphaproteobacteria	Rhodospirillales	Acetobacteraceae	unclassified
1519	0	3	4	2	Proteobacteria	Alphaproteobacteria	Rhodobacterales	Rhodobacteraceae	Rubellimicrobium
1535	0	1	0	0	Proteobacteria	Alphaproteobacteria	Rhodobacterales	Rhodobacteraceae	unclassified
1538	1	2	1	0	Proteobacteria	Alphaproteobacteria	Rhizobiales	Bradyrhizobiaceae	unclassified
1540	0	1	0	0	Proteobacteria	Alphaproteobacteria	Rhodospirillales	Acetobacteraceae	Roseomonas
1546	0	1	0	0	Proteobacteria	unclassified	unclassified	unclassified	unclassified
1548	0	1	0	0	Proteobacteria	Betaproteobacteria	Burkholderiales	Comamonadaceae	Hydrogenophaga
1551	0	0	0	2	Proteobacteria	Alphaproteobacteria	Rhodospirillales	Acetobacteraceae	unclassified
1552	0	1	0	0	Proteobacteria	Alphaproteobacteria	Rhizobiales	Hyphomicrobiaceae	Rhodoplanes
1553	0	1	4	1	Proteobacteria	Alphaproteobacteria	Rhodospirillales	Acetobacteraceae	Roseomonas
1554	2	0	0	1	Proteobacteria	Gammaproteobacteria	Enterobacteriales	Enterobacteriaceae	Ewingella
1560	0	0	0	1	Proteobacteria	Gammaproteobacteria	Enterobacteriales	Enterobacteriaceae	unclassified
1567	0	1	0	0	Proteobacteria	Alphaproteobacteria	Sphingomonadales	Sphingomonadaceae	Sphingomonas
1569	0	0	0	1	Proteobacteria	Gammaproteobacteria	Pseudomonadales	Pseudomonadaceae	Pseudomonas
1586	0	17	0	0	Proteobacteria	Alphaproteobacteria	Rhodobacterales	Rhodobacteraceae	unclassified
1588	0	1	0	0	Proteobacteria	Alphaproteobacteria	Rhizobiales	Nordellaceae	Nordella
1595	0	1	0	0	Proteobacteria	Alphaproteobacteria	Rhodospirillales	Acetobacteraceae	Roseomonas
1597	0	0	3	0	Proteobacteria	Gammaproteobacteria	Oceanospirillales	211ds20	unclassified
1598	0	1	0	0	Proteobacteria	Alphaproteobacteria	Rhodobacterales	Rhodobacteraceae	unclassified
1600	0	2	2	3	Proteobacteria	Alphaproteobacteria	Rhodospirillales	Acetobacteraceae	unclassified
1602	0	1	0	0	Proteobacteria	Gammaproteobacteria	Xanthomonadales	Xanthomonadaceae	unclassified
1604	0	2	6	0	Proteobacteria	Alphaproteobacteria	Rhodospirillales	Acetobacteraceae	Roseomonas
1605	2	14	29	7	Proteobacteria	Alphaproteobacteria	Rhizobiales	Methylobacteriaceae	Methylobacterium
1607	0	0	12	0	Proteobacteria	Deltaproteobacteria	Myxococcales	Haliangiaceae	unclassified
1619	9	0	0	0	Proteobacteria	Alphaproteobacteria	Sphingomonadales	Erythrobacteraceae	unclassified
1620	0	2	0	3	Proteobacteria	Alphaproteobacteria	Rickettsiales	Rickettsiaceae	Rickettsia

Table S5 (part O): OTUs, no. of reads and corresponding taxonomic affiliation of detected bacterial 16S rRNA amplicons by 454 pyrotag sequencing. Please note, the data shown here was either rank normalized or sum normlaized (to 5000 reads per sample) before performing statisical analysis as depicted in the manuscript.

	Samples and no. of reads				taxonomic classification of bacterial 16S rRNA gene amplicons				
OTU	UR	CO	CR8	CR5	phylum	class	order	family	genus
1621	0	0	1	0	Proteobacteria	Gammaproteobacteria	Xanthomonadales	Xanthomonadaceae	unclassified
1624	0	5	2	3	Proteobacteria	Betaproteobacteria	Burkholderiales	Burkholderiaceae	unclassified
1627	0	1	0	0	Proteobacteria	Alphaproteobacteria	Rhizobiales	Hyphomicrobiaceae	Devosia
1633	0	0	2	0	Proteobacteria	unclassified	unclassified	unclassified	unclassified
1637	2	5	4	12	Proteobacteria	Alphaproteobacteria	Sphingomonadales	Sphingomonadaceae	Sphingomonas
1639	2	1	1	1	Proteobacteria	Betaproteobacteria	Neisseriales	Neisseriaceae	Neisseria
1640	0	0	0	2	Proteobacteria	Alphaproteobacteria	Rhodobacterales	Rhodobacteraceae	Amaricoccus
1641	0	0	0	1	Proteobacteria	Gammaproteobacteria	Xanthomonadales	Xanthomonadaceae	unclassified
1643	15	6	11	7	Proteobacteria	Gammaproteobacteria	Pseudomonadales	Moraxellaceae	Enhydrobacter
1646	0	0	2	1	Proteobacteria	Alphaproteobacteria	Caulobacterales	Caulobacteraceae	Caulobacter
1647	0	1	0	0	Proteobacteria	unclassified	unclassified	unclassified	unclassified
1648	0	2	0	0	Proteobacteria	Betaproteobacteria	Burkholderiales	Alcaligenaceae	Alcaligenes
1654	0	3	2	0	Proteobacteria	Alphaproteobacteria	Rhizobiales	Bradyrhizobiaceae	unclassified
1655	0	1	0	1	Proteobacteria	Alphaproteobacteria	Rhizobiales	unclassified	unclassified
1661	30	1	14	6	Proteobacteria	Gammaproteobacteria	Xanthomonadales	Xanthomonadaceae	Stenotrophomonas
1662	0	0	2	0	Proteobacteria	Alphaproteobacteria	Sphingomonadales	Sphingomonadaceae	unclassified
1666	0	6	0	0	Proteobacteria	Betaproteobacteria	Burkholderiales	Burkholderiaceae	unclassified
1667	0	1	0	0	Proteobacteria	Alphaproteobacteria	Sphingomonadales	Sphingomonadaceae	Sphingobium
1673	1	0	0	0	Proteobacteria	Gammaproteobacteria	unclassified	unclassified	unclassified
1674	0	0	0	1	Proteobacteria	Gammaproteobacteria	Pasteurellales	Pasteurellaceae	Haemophilus
1675	0	2	1	0	Proteobacteria	Alphaproteobacteria	Rhizobiales	unclassified	unclassified
1679	0	0	1	1	Proteobacteria	Gammaproteobacteria	Aeromonadales	Aeromonadaceae	Aeromonas
1682	0	0	2	0	Proteobacteria	Alphaproteobacteria	Rhizobiales	Methylobacteriaceae	Methylobacterium
1683	0	0	1	0	Proteobacteria	Alphaproteobacteria	Rhodobacterales	Rhodobacteraceae	unclassified
1690	3	17	9	6	Proteobacteria	Alphaproteobacteria	Sphingomonadales	unclassified	unclassified
1692	0	1	0	0	Proteobacteria	Alphaproteobacteria	Sphingomonadales	unclassified	unclassified
1695	0	1	0	0	Proteobacteria	Alphaproteobacteria	unclassified	unclassified	unclassified
1702	2	2	0	1	Proteobacteria	Alphaproteobacteria	Sphingomonadales	Sphingomonadaceae	Sphingomonas
1703	5	0	2	0	Proteobacteria	Alphaproteobacteria	Sphingomonadales	Sphingomonadaceae	Sphingobium
1712	0	2	0	0	Proteobacteria	Alphaproteobacteria	Sphingomonadales	Sphingomonadaceae	Zymomonas
1713	0	6	0	1	Proteobacteria	Alphaproteobacteria	Sphingomonadales	Erythrobacteraceae	unclassified
1714	0	2	0	0	Proteobacteria	Alphaproteobacteria	Sphingomonadales	Sphingomonadaceae	unclassified
1715	1	1	0	2	Proteobacteria	Betaproteobacteria	Neisseriales	Neisseriaceae	Neisseria
1716	0	2	1	4	Proteobacteria	Alphaproteobacteria	Rhodobacterales	Rhodobacteraceae	Rubellimicrobium
1718	1	3	7	0	Proteobacteria	Alphaproteobacteria	Rhizobiales	Phyllobacteriaceae	Mesorhizobium
1719	0	0	2	0	Proteobacteria	Gammaproteobacteria	Legionellales	Coxiellaceae	unclassified
339	0	2	0	0	SC4	unclassified	unclassified	unclassified	unclassified
536	0	3	1	0	SC4	unclassified	unclassified	unclassified	unclassified
565	0	7	0	4	SC4	unclassified	unclassified	unclassified	unclassified
626	1	0	0	0	SC4	unclassified	unclassified	unclassified	unclassified
1015	0	2	0	0	SC4	unclassified	unclassified	unclassified	unclassified
1133	1	0	0	0	SC4	unclassified	unclassified	unclassified	unclassified
1328	0	1	0	0	SC4	unclassified	unclassified	unclassified	unclassified
322	2	0	0	0	Spirochaetes	Spirochaetes	Spirochaetales	Spirochaetaceae	Treponema
483	0	0	2	0	Tenericutes	Erysipelotrichi	Erysipelotrichales	Erysipelotrichaceae	ATCC_27806
130	0	5	0	3	Thermi	Deinococci	Deinococcales	Deinococcaceae	Deinococcus
189	1	27	4	14	Thermi	Deinococci	Deinococcales	Deinococcaceae	Deinococcus
433	0	7	3	2	Thermi	Deinococci	Deinococcales	Deinococcaceae	Deinococcus
613	0	1	0	0	Thermi	Deinococci	Deinococcales	Deinococcaceae	Deinococcus
746	0	1	0	0	Thermi	Deinococci	Deinococcales	Deinococcaceae	Deinococcus
758	0	21	0	0	Thermi	Deinococci	Deinococcales	Deinococcaceae	Deinococcus
995	0	1	0	0	Thermi	Deinococci	Deinococcales	Deinococcaceae	Deinococcus
1191	0	3	0	2	Thermi	Deinococci	Deinococcales	Deinococcaceae	Deinococcus
1197	0	1	0	0	Thermi	Deinococci	Deinococcales	Deinococcaceae	Deinococcus
1220	0	0	0	2	Thermi	Deinococci	Deinococcales	Deinococcaceae	Deinococcus
1449	0	5	1	0	Thermi	Deinococci	Deinococcales	Deinococcaceae	Deinococcus
1461	0	2	0	1	Thermi	Deinococci	Deinococcales	Deinococcaceae	Deinococcus
1527	0	1	1	0	Thermi	Deinococci	Deinococcales	Deinococcaceae	Deinococcus
1623	0	2	0	0	Thermi	Deinococci	Deinococcales	Deinococcaceae	Deinococcus
1670	0	1	0	1	Thermi	Deinococci	Deinococcales	Deinococcaceae	Deinococcus
756	0	0	0	1	TM6	SJA-4	unclassified	unclassified	unclassified
68	0	0	0	1	unclassified	unclassified	unclassified	unclassified	unclassified
71	0	0	0	3	unclassified	unclassified	unclassified	unclassified	unclassified
143	0	0	1	0	unclassified	unclassified	unclassified	unclassified	unclassified
157	0	0	3	0	unclassified	unclassified	unclassified	unclassified	unclassified
178	0	1	0	0	unclassified	unclassified	unclassified	unclassified	unclassified
290	0	0	1	0	unclassified	unclassified	unclassified	unclassified	unclassified
498	0	1	0	0	unclassified	unclassified	unclassified	unclassified	unclassified
525	0	1	0	0	unclassified	unclassified	unclassified	unclassified	unclassified
548	0	2	0	2	unclassified	unclassified	unclassified	unclassified	unclassified
567	0	1	0	0	unclassified	unclassified	unclassified	unclassified	unclassified
894	0	2	0	0	unclassified	unclassified	unclassified	unclassified	unclassified
906	0	1	0	0	unclassified	unclassified	unclassified	unclassified	unclassified
996	0	0	2	0	unclassified	unclassified	unclassified	unclassified	unclassified
1021	0	1	0	1	unclassified	unclassified	unclassified	unclassified	unclassified
1057	0	0	0	1	unclassified	unclassified	unclassified	unclassified	unclassified
1132	0	1	0	0	unclassified	unclassified	unclassified	unclassified	unclassified
1148	0	1	1	0	unclassified	unclassified	unclassified	unclassified	unclassified
1174	1	0	0	0	unclassified	unclassified	unclassified	unclassified	unclassified
1370	0	1	0	0	unclassified	unclassified	unclassified	unclassified	unclassified
1474	0	2	0	0	unclassified	unclassified	unclassified	unclassified	unclassified
1512	0	1	0	0	unclassified	unclassified	unclassified	unclassified	unclassified
1514	1	1	0	0	unclassified	unclassified	unclassified	unclassified	unclassified
317	0	1	0	0	Verrucomicrobia	Verrucomicrobiae	Verrucomicrobiales	Verrucomicrobiaceae	Luteolibacter
663	0	1	0	0	Verrucomicrobia	Verrucomicrobiae	Verrucomicrobiales	unclassified	unclassified
1240	0	2	0	0	Verrucomicrobia	Verrucomicrobiae	Verrucomicrobiales	unclassified	unclassified
1306	0	0	0	1	WPS-2	unclassified	unclassified	unclassified	unclassified

Table S6 (part A): HybScores of OTUs that were called present in at least one of the samples. Taxonomic classifications are based on PhyloChip taxonomy (derived from GreenGenes). Please note, that data presented here was rank normalized before performing statisical analyses presented in the manuscript.

	Sample ID (PMA)				Sample ID				taxonomic classification of representative rOTU cluster						
OTU	UR_PMA	CO_PMA	CR8_PMA	CR5_PMA	UR	CO	CR8	CR5	kingdom	phylum	class	order	family	genus	species
51048	11074	9537	7473	7676	12119	12905	11946	12182	Bacteria	Acidobacteria	Acidobacteria	Acidobacteriales	Acidobacteriaceae	unclassified	unclassified
51676	9105	9538	8885	8077	12423	13335	13082	12945	Bacteria	Acidobacteria	Acidobacteria	Acidobacteriales	Acidobacteriaceae	unclassified	unclassified
58588	8247	8294	7605	7117	10952	12084	11550	12352	Bacteria	Acidobacteria	Acidobacteria	Acidobacteriales	Acidobacteriaceae	unclassified	unclassified
51431	8664	11058	7198	7362	10498	12040	11381	11562	Bacteria	Acidobacteria	BPC102	unclassified	unclassified	sfF	unclassified
41119	9394	9812	9028	8572	11446	12421	11719	11963	Bacteria	Actinobacteria	Actinobacteria	0319-7L14	unclassified	sfA	unclassified
41305	8548	9407	8024	7271	12209	13139	12291	12637	Bacteria	Actinobacteria	Actinobacteria	0319-7L14	unclassified	sfA	unclassified
42582	8870	8815	6768	7747	11632	12518	11794	12400	Bacteria	Actinobacteria	Actinobacteria	Acidimicrobiales	Acidimicrobiaceae	unclassified	unclassified
42676	10082	11138	10157	9552	12768	13557	13089	13113	Bacteria	Actinobacteria	Actinobacteria	Acidimicrobiales	Acidimicrobiaceae	unclassified	unclassified
42818	10167	11183	9558	8888	12932	13801	13205	13240	Bacteria	Actinobacteria	Actinobacteria	Acidimicrobiales	Acidimicrobiaceae	unclassified	unclassified
42997	9160	9210	6608	7664	11848	12404	11648	11608	Bacteria	Actinobacteria	Actinobacteria	Acidimicrobiales	Acidimicrobiaceae	unclassified	unclassified
43927	9217	9087	6794	7809	11092	12129	11092	11774	Bacteria	Actinobacteria	Actinobacteria	Acidimicrobiales	Acidimicrobiaceae	unclassified	unclassified
44151	8746	9822	6076	6581	12048	12932	12229	12162	Bacteria	Actinobacteria	Actinobacteria	Acidimicrobiales	Acidimicrobiaceae	unclassified	unclassified
42622	8864	9836	6081	7187	11634	12211	12064	11875	Bacteria	Actinobacteria	Actinobacteria	Acidimicrobiales	AKIW874	unclassified	unclassified
42505	8724	7349	6302	6803	11460	12750	11563	11448	Bacteria	Actinobacteria	Actinobacteria	Acidimicrobiales	CL500-29	unclassified	unclassified
42479	9891	9898	8323	7478	12610	13126	12218	12754	Bacteria	Actinobacteria	Actinobacteria	Acidimicrobiales	EB1017	unclassified	unclassified
42497	9314	8853	6689	7591	11849	12442	11753	12259	Bacteria	Actinobacteria	Actinobacteria	Acidimicrobiales	EB1017	unclassified	unclassified
42959	9438	10753	5703	6514	12747	13637	13052	13063	Bacteria	Actinobacteria	Actinobacteria	Acidimicrobiales	EB1017	unclassified	unclassified
43227	9437	9593	8323	7922	12432	12790	12694	12399	Bacteria	Actinobacteria	Actinobacteria	Acidimicrobiales	Microthrixaceae	unclassified	unclassified
40921	8773	9084	7343	6540	12056	12976	12043	12406	Bacteria	Actinobacteria	Actinobacteria	Actinomycetales	ACK-M1	unclassified	unclassified
40164	9491	9625	7421	8357	11704	11639	11679	11878	Bacteria	Actinobacteria	Actinobacteria	Actinomycetales	Actinomycetaceae	Actinomyces	Actinomyces oris
40286	9279	9772	7326	8325	12002	11710	12091	12078	Bacteria	Actinobacteria	Actinobacteria	Actinomycetales	Actinomycetaceae	Actinomyces	Actinomyces oris
40334	9764	9699	8250	8768	12126	12535	12362	12826	Bacteria	Actinobacteria	Actinobacteria	Actinomycetales	Actinomycetaceae	Actinomyces	Actinomyces oris
40651	8960	7950	7649	8314	11540	11376	11917	11156	Bacteria	Actinobacteria	Actinobacteria	Actinomycetales	Actinomycetaceae	Actinomyces	Actinomyces oris
40779	10193	9968	7661	8476	12720	12515	12727	12842	Bacteria	Actinobacteria	Actinobacteria	Actinomycetales	Actinomycetaceae	Actinomyces	Actinomyces oris
39415	8376	9079	6249	7222	11282	12764	12279	12456	Bacteria	Actinobacteria	Actinobacteria	Actinomycetales	Actinomycetaceae	Actinomyces	unclassified
39607	9396	10711	8133	7743	12624	13024	12548	13020	Bacteria	Actinobacteria	Actinobacteria	Actinomycetales	Actinomycetaceae	Actinomyces	unclassified
40407	9409	8817	8061	8801	11817	11765	12035	11644	Bacteria	Actinobacteria	Actinobacteria	Actinomycetales	Actinomycetaceae	Actinomyces	unclassified
40442	8297	9205	6854	7600	11861	12854	12115	12462	Bacteria	Actinobacteria	Actinobacteria	Actinomycetales	Actinomycetaceae	Actinomyces	unclassified
40443	9397	8451	7079	7914	11586	11429	11844	11176	Bacteria	Actinobacteria	Actinobacteria	Actinomycetales	Actinomycetaceae	Actinomyces	unclassified
40492	8452	9830	6650	8121	11669	11759	12089	11720	Bacteria	Actinobacteria	Actinobacteria	Actinomycetales	Actinomycetaceae	Actinomyces	unclassified
40549	8102	7004	6119	6585	11306	12071	11718	11997	Bacteria	Actinobacteria	Actinobacteria	Actinomycetales	Actinomycetaceae	Actinomyces	unclassified
40733	8217	8399	7963	8268	12059	12107	12296	12090	Bacteria	Actinobacteria	Actinobacteria	Actinomycetales	Actinomycetaceae	Actinomyces	unclassified
40774	8679	7404	6608	7624	11444	11203	11831	10999	Bacteria	Actinobacteria	Actinobacteria	Actinomycetales	Actinomycetaceae	Actinomyces	unclassified
40838	8679	7404	6608	7624	11444	11203	11831	10999	Bacteria	Actinobacteria	Actinobacteria	Actinomycetales	Actinomycetaceae	Actinomyces	unclassified
40892	8692	10148	6712	8176	11990	12319	12370	12109	Bacteria	Actinobacteria	Actinobacteria	Actinomycetales	Actinomycetaceae	Actinomyces	unclassified
40929	9151	10014	9338	9315	12070	12261	12289	11972	Bacteria	Actinobacteria	Actinobacteria	Actinomycetales	Actinomycetaceae	Actinomyces	unclassified
40958	8277	8751	6791	7599	11764	11861	12130	11704	Bacteria	Actinobacteria	Actinobacteria	Actinomycetales	Actinomycetaceae	Actinomyces	unclassified
37393	9594	8836	6959	7390	12110	12918	12410	13181	Bacteria	Actinobacteria	Actinobacteria	Actinomycetales	Actinosynnemataceae	Actinokineospora	Actinokineospora diospyrosa
37407	8258	8441	6571	7391	12315	13061	12491	12941	Bacteria	Actinobacteria	Actinobacteria	Actinomycetales	Actinosynnemataceae	Actinokineospora	Actinokineospora diospyrosa
37619	9520	11856	6986	7510	12947	13811	13158	13621	Bacteria	Actinobacteria	Actinobacteria	Actinomycetales	Actinosynnemataceae	Actinokineospora	Actinokineospora diospyrosa
41015	8003	7830	6291	6853	11595	11989	11655	12826	Bacteria	Actinobacteria	Actinobacteria	Actinomycetales	Beutenbergiaceae	unclassified	unclassified
40375	9522	9854	6806	8167	11034	12426	11443	11694	Bacteria	Actinobacteria	Actinobacteria	Actinomycetales	Bogoriellaceae	Georgenia	unclassified
40890	8457	8577	6604	6913	10995	12212	11543	11710	Bacteria	Actinobacteria	Actinobacteria	Actinomycetales	Bogoriellaceae	Georgenia	unclassified
40983	8132	7860	6485	7092	11502	12313	12702	12432	Bacteria	Actinobacteria	Actinobacteria	Actinomycetales	Brevibacteriaceae	Brevibacterium	Brevibacterium paucivorans
40719	9426	10686	6956	7610	13102	13080	13209	13115	Bacteria	Actinobacteria	Actinobacteria	Actinomycetales	Brevibacteriaceae	Brevibacterium	unclassified
40792	8560	10614	7189	7526	13036	13134	13220	13011	Bacteria	Actinobacteria	Actinobacteria	Actinomycetales	Brevibacteriaceae	Brevibacterium	unclassified
40882	9463	10346	7924	8655	12329	12704	12325	12537	Bacteria	Actinobacteria	Actinobacteria	Actinomycetales	Brevibacteriaceae	Brevibacterium	unclassified
40611	10103	11102	7501	8998	12542	13356	12605	12847	Bacteria	Actinobacteria	Actinobacteria	Actinomycetales	Cellulomonadaceae	Cellulomonas	unclassified
40619	8615	8921	6867	7660	10866	12366	11033	10933	Bacteria	Actinobacteria	Actinobacteria	Actinomycetales	Cellulomonadaceae	Cellulomonas	unclassified
40917	9715	11373	6521	7469	12012	13240	12229	12510	Bacteria	Actinobacteria	Actinobacteria	Actinomycetales	Cellulomonadaceae	Cellulomonas	unclassified
40925	10814	11566	7567	9452	12986	13649	13031	13159	Bacteria	Actinobacteria	Actinobacteria	Actinomycetales	Cellulomonadaceae	Cellulomonas	unclassified
37633	10534	10332	10194	9033	12245	13029	12133	12562	Bacteria	Actinobacteria	Actinobacteria	Actinomycetales	Cellulomonadaceae	unclassified	unclassified
37779	9084	10288	6604	8216	11430	12834	11609	11821	Bacteria	Actinobacteria	Actinobacteria	Actinomycetales	Cellulomonadaceae	unclassified	unclassified
37924	8637	9336	6735	7314	11335	12647	11731	12101	Bacteria	Actinobacteria	Actinobacteria	Actinomycetales	Cellulomonadaceae	unclassified	unclassified
38165	8701	8974	8028	8187	11253	12163	11330	11737	Bacteria	Actinobacteria	Actinobacteria	Actinomycetales	Cellulomonadaceae	unclassified	unclassified
38303	8774	9442	7130	6944	12520	13332	12511	12851	Bacteria	Actinobacteria	Actinobacteria	Actinomycetales	Cellulomonadaceae	unclassified	unclassified
39019	8868	7959	7191	8092	11259	12365	11609	11374	Bacteria	Actinobacteria	Actinobacteria	Actinomycetales	Cellulomonadaceae	unclassified	unclassified
39588	9023	9086	7155	7584	13079	13558	12826	13429	Bacteria	Actinobacteria	Actinobacteria	Actinomycetales	Cellulomonadaceae	unclassified	unclassified
39602	8448	8524	7193	8218	10886	12378	11271	11739	Bacteria	Actinobacteria	Actinobacteria	Actinomycetales	Cellulomonadaceae	unclassified	unclassified
40117	8772	9017	7267	7425	11368	12507	11676	12099	Bacteria	Actinobacteria	Actinobacteria	Actinomycetales	Cellulomonadaceae	unclassified	unclassified
40148	9532	11002	6511	7464	12473	13758	12601	12805	Bacteria	Actinobacteria	Actinobacteria	Actinomycetales	Cellulomonadaceae	unclassified	unclassified
40335	8342	9214	6637	6701	11972	12774	12541	11922	Bacteria	Actinobacteria	Actinobacteria	Actinomycetales	Cellulomonadaceae	unclassified	unclassified
40412	8754	8702	6528	6975	11713	12619	11437	11791	Bacteria	Actinobacteria	Actinobacteria	Actinomycetales	Cellulomonadaceae	unclassified	unclassified
40419	9619	9385	6770	8143	11392	12962	12157	12129	Bacteria	Actinobacteria	Actinobacteria	Actinomycetales	Cellulomonadaceae	unclassified	unclassified
40988	9362	10246	7635	8317	12410	13545	12718	12930	Bacteria	Actinobacteria	Actinobacteria	Actinomycetales	Cellulomonadaceae	unclassified	unclassified
41745	9505	11141	6747	8448	12456	13357	12294	12836	Bacteria	Actinobacteria	Actinobacteria	Actinomycetales	Cellulomonadaceae	unclassified	unclassified
41851	8438	9648	6508	6892	12446	13386	12798	13003	Bacteria	Actinobacteria	Actinobacteria	Actinomycetales	Cellulomonadaceae	unclassified	unclassified
42097	9561	9406	8650	8023	11928	12392	12149	12212	Bacteria	Actinobacteria	Actinobacteria	Actinomycetales	Cellulomonadaceae	unclassified	unclassified
36899	9258	9982	7377	7780	12809	13098	13108	13800	Bacteria	Actinobacteria	Actinobacteria	Actinomycetales	Corynebacteriaceae	Corynebacterium	Corynebacterium flavescens
37247	8676	7711	6992	7603	12226	12298	12362	13381	Bacteria	Actinobacteria	Actinobacteria	Actinomycetales	Corynebacteriaceae	Corynebacterium	Corynebacterium jeikeium
37280	10312	9674	6906	8037	13437	13406	13671	14228	Bacteria	Actinobacteria	Actinobacteria	Actinomycetales	Corynebacteriaceae	Corynebacterium	Corynebacterium riegelii
37312	9859	9573	9186	9012	12206	12668	12570	12776	Bacteria	Actinobacteria	Actinobacteria	Actinomycetales	Corynebacteriaceae	Corynebacterium	Corynebacterium variabile
28504	12552	13345	12771	12431	14209	14192	13956	14258	Bacteria	Actinobacteria	Actinobacteria	Actinomycetales	Corynebacteriaceae	Corynebacterium	unclassified
28641	13072	13073	12236	11584	14387	14187	14188	14399	Bacteria	Actinobacteria	Actinobacteria	Actinomycetales	Corynebacteriaceae	Corynebacterium	unclassified
28655	8300	8544	5900	6775	11792	13045	12271	13034	Bacteria	Actinobacteria	Actinobacteria	Actinomycetales	Corynebacteriaceae	Corynebacterium	unclassified
28664	8464	8987	6315	7030	11151	12106	11388	12234	Bacteria	Actinobacteria	Actinobacteria	Actinomycetales	Corynebacteriaceae	Corynebacterium	unclassified
28691	11759	12293	10928	11004	14348	14223	14164	14501	Bacteria	Actinobacteria	Actinobacteria	Actinomycetales	Corynebacteriaceae	Corynebacterium	unclassified
28754	12343	12589	12320	12237	14175	13923	13853	14249	Bacteria	Actinobacteria	Actinobacteria	Actinomycetales	Corynebacteriaceae	Corynebacterium	unclassified
28768	8657	8964	7262	6955	11745	12310	11988	12409	Bacteria	Actinobacteria	Actinobacteria	Actinomycetales	Corynebacteriaceae	Corynebacterium	unclassified
28819	10634	11062	10508	10204	13608	13739	13820	14009	Bacteria	Actinobacteria	Actinobacteria	Actinomycetales	Corynebacteriaceae	Corynebacterium	unclassified
28952	9229	9204	8989	7774	12920	13373	13251	13824	Bacteria	Actinobacteria	Actinobacteria	Actinomycetales	Corynebacteriaceae	Corynebacterium	unclassified
28961	11175	11590	11102	10857	13628	13714	13429	13770	Bacteria	Actinobacteria	Actinobacteria	Actinomycetales	Corynebacteriaceae	Corynebacterium	unclassified
28989	9403	9046	7497	8525	11204	12163	11553	12157	Bacteria	Actinobacteria	Actinobacteria	Actinomycetales	Corynebacteriaceae	Corynebacterium	unclassified
29061	9906	9892	8886	7878	12804	13064	12779	13450	Bacteria	Actinobacteria	Actinobacteria	Actinomycetales	Corynebacteriaceae	Corynebacterium	unclassified
29693	10827	11892	10004	9225	14000	13985	14188	14343	Bacteria	Actinobacteria	Actinobacteria	Actinomycetales	Corynebacteriaceae	Corynebacterium	unclassified
29743	12199	12633	11640	11449	14461	14219	14250	14644	Bacteria	Actinobacteria	Actinobacteria	Actinomycetales	Corynebacteriaceae	Corynebacterium	unclassified
29901	9067	8180	6946	7092	11727	12451	12004	13055	Bacteria	Actinobacteria	Actinobacteria	Actinomycetales	Corynebacteriaceae	Corynebacterium	unclassified
29909	9162	8756	6483	7702	12114	12436	12293	13635	Bacteria	Actinobacteria	Actinobacteria	Actinomycetales	Corynebacteriaceae	Corynebacterium	unclassified
30016	9337	8342	7502	8444	11286	12261	11581	12156	Bacteria	Actinobacteria	Actinobacteria	Actinomycetales	Corynebacteriaceae	Corynebacterium	unclassified
30017	9952	10194	10002	8165	13203	13291	13344	13905	Bacteria	Actinobacteria	Actinobacteria	Actinomycetales	Corynebacteriaceae	Corynebacterium	unclassified
30142	11163	11929	11221	11255	14136	14001	14015	14184	Bacteria	Actinobacteria	Actinobacteria	Actinomycetales	Corynebacteriaceae	Corynebacterium	unclassified
30177	12363	12916	12085	11832	14646	14335	14249	14580	Bacteria	Actinobacteria	Actinobacteria	Actinomycetales	Corynebacteriaceae	Corynebacterium	unclassified
30421	9869	10099	9308	8424	13186	13207	13010	13719	Bacteria	Actinobacteria	Actinobacteria	Actinomycetales	Corynebacteriaceae	Corynebacterium	unclassified
30430	12459	13197	12391	12101	14641	14100	14063	14366	Bacteria	Actinobacteria	Actinobacteria	Actinomycetales	Corynebacteriaceae	Corynebacterium	unclassified
30513	12352	12573	11975	11870	14090	14040	13870	14200	Bacteria	Actinobacteria	Actinobacteria	Actinomycetales	Corynebacteriaceae	Corynebacterium	unclassified
30645	9566	10333	7524	8545	12769	12918	12818	13577	Bacteria	Actinobacteria	Actinobacteria	Actinomycetales	Corynebacteriaceae	Corynebacterium	unclassified
30652	12469	13013	11920	12021	14911	14411	14487	14772	Bacteria	Actinobacteria	Actinobacteria	Actinomycetales	Corynebacteriaceae	Corynebacterium	unclassified
30734	12034	12555	12153	11063	14186	14186	14066	14425	Bacteria	Actinobacteria	Actinobacteria	Actinomycetales	Corynebacteriaceae	Corynebacterium	unclassified
30849	12491	13287	12554	12411	14654	14331	14406	14675	Bacteria	Actinobacteria	Actinobacteria	Actinomycetales	Corynebacteriaceae	Corynebacterium	unclassified
30882	9793	10297	9779	8184	12450	12617	12043	13194	Bacteria	Actinobacteria	Actinobacteria	Actinomycetales	Corynebacteriaceae	Corynebacterium	unclassified

Table S6 (part B): HybScores of OTUs that were called present in at least one of the samples. Taxonomic classifications are based on PhyloChip taxonomy (derived from GreenGenes). Please note, that data presented here was rank normalized before performing statisical analyses presented in the manuscript.

	Sample ID (PMA)				Sample ID				taxonomic classification of representative rOTU cluster						
OTU	UR_PMA	CO_PMA	CR8_PMA	CR5_PMA	UR	CO	CR8	CR5	kingdom	phylum	class	order	family	genus	species
30922	10373	10545	8707	8517	12392	12691	12452	12886	Bacteria	Actinobacteria	Actinobacteria	Actinomycetales	Corynebacteriaceae	Corynebacterium	unclassified
30996	12222	12760	12292	10988	14214	14191	14124	14459	Bacteria	Actinobacteria	Actinobacteria	Actinomycetales	Corynebacteriaceae	Corynebacterium	unclassified
31080	9314	8137	6294	7015	12274	12638	12367	13365	Bacteria	Actinobacteria	Actinobacteria	Actinomycetales	Corynebacteriaceae	Corynebacterium	unclassified
31163	10500	11612	8983	7816	13198	13358	13327	13901	Bacteria	Actinobacteria	Actinobacteria	Actinomycetales	Corynebacteriaceae	Corynebacterium	unclassified
31205	8507	9169	6453	6700	12331	12816	12185	12316	Bacteria	Actinobacteria	Actinobacteria	Actinomycetales	Corynebacteriaceae	Corynebacterium	unclassified
31218	11793	12448	12025	12087	13933	13883	14078	14047	Bacteria	Actinobacteria	Actinobacteria	Actinomycetales	Corynebacteriaceae	Corynebacterium	unclassified
31307	12659	13217	12838	12695	14141	14081	14040	14276	Bacteria	Actinobacteria	Actinobacteria	Actinomycetales	Corynebacteriaceae	Corynebacterium	unclassified
31479	9451	9962	7306	7278	13363	13645	13557	13974	Bacteria	Actinobacteria	Actinobacteria	Actinomycetales	Corynebacteriaceae	Corynebacterium	unclassified
31616	10603	11130	10605	9112	13126	13280	13095	13564	Bacteria	Actinobacteria	Actinobacteria	Actinomycetales	Corynebacteriaceae	Corynebacterium	unclassified
31655	10422	10609	10208	8642	13148	13277	13168	13869	Bacteria	Actinobacteria	Actinobacteria	Actinomycetales	Corynebacteriaceae	Corynebacterium	unclassified
31702	11315	12178	10364	9869	13972	14034	14027	14349	Bacteria	Actinobacteria	Actinobacteria	Actinomycetales	Corynebacteriaceae	Corynebacterium	unclassified
31823	12676	13325	12880	12074	14217	14321	14346	14566	Bacteria	Actinobacteria	Actinobacteria	Actinomycetales	Corynebacteriaceae	Corynebacterium	unclassified
32200	11515	11894	11410	9988	12724	13021	12706	13230	Bacteria	Actinobacteria	Actinobacteria	Actinomycetales	Corynebacteriaceae	Corynebacterium	unclassified
32344	12760	13429	12618	11830	14622	14455	14479	14816	Bacteria	Actinobacteria	Actinobacteria	Actinomycetales	Corynebacteriaceae	Corynebacterium	unclassified
32357	9158	8017	5839	6806	13063	12974	13053	14157	Bacteria	Actinobacteria	Actinobacteria	Actinomycetales	Corynebacteriaceae	Corynebacterium	unclassified
32411	11495	12582	11489	11283	14391	14385	14426	14482	Bacteria	Actinobacteria	Actinobacteria	Actinomycetales	Corynebacteriaceae	Corynebacterium	unclassified
32607	9150	9009	7498	7676	12736	12763	12646	13698	Bacteria	Actinobacteria	Actinobacteria	Actinomycetales	Corynebacteriaceae	Corynebacterium	unclassified
32857	10302	10739	9588	8219	13623	13435	13228	14067	Bacteria	Actinobacteria	Actinobacteria	Actinomycetales	Corynebacteriaceae	Corynebacterium	unclassified
33058	9596	10293	7924	7846	13189	13426	13207	13828	Bacteria	Actinobacteria	Actinobacteria	Actinomycetales	Corynebacteriaceae	Corynebacterium	unclassified
33189	8287	7098	7029	6452	11653	12198	11408	12703	Bacteria	Actinobacteria	Actinobacteria	Actinomycetales	Corynebacteriaceae	Corynebacterium	unclassified
33258	12061	12806	11219	10985	14626	14402	14540	14812	Bacteria	Actinobacteria	Actinobacteria	Actinomycetales	Corynebacteriaceae	Corynebacterium	unclassified
33300	9380	9599	9276	8510	12013	12823	12142	12993	Bacteria	Actinobacteria	Actinobacteria	Actinomycetales	Corynebacteriaceae	Corynebacterium	unclassified
33488	10117	10731	9727	8705	13740	13750	13276	14094	Bacteria	Actinobacteria	Actinobacteria	Actinomycetales	Corynebacteriaceae	Corynebacterium	unclassified
33570	8889	7768	7373	7996	11803	12598	11937	12860	Bacteria	Actinobacteria	Actinobacteria	Actinomycetales	Corynebacteriaceae	Corynebacterium	unclassified
33576	9333	9679	7246	8438	13185	13328	13463	13959	Bacteria	Actinobacteria	Actinobacteria	Actinomycetales	Corynebacteriaceae	Corynebacterium	unclassified
33646	9162	9190	7287	7727	12350	12673	12764	13269	Bacteria	Actinobacteria	Actinobacteria	Actinomycetales	Corynebacteriaceae	Corynebacterium	unclassified
33688	10768	11096	10387	9381	13413	13525	13395	13879	Bacteria	Actinobacteria	Actinobacteria	Actinomycetales	Corynebacteriaceae	Corynebacterium	unclassified
33700	9764	9571	7701	8393	13796	13858	13777	14185	Bacteria	Actinobacteria	Actinobacteria	Actinomycetales	Corynebacteriaceae	Corynebacterium	unclassified
33847	9439	9823	9046	7341	12809	13057	13074	13736	Bacteria	Actinobacteria	Actinobacteria	Actinomycetales	Corynebacteriaceae	Corynebacterium	unclassified
33873	12069	12643	12338	11191	13843	13908	13846	14084	Bacteria	Actinobacteria	Actinobacteria	Actinomycetales	Corynebacteriaceae	Corynebacterium	unclassified
33892	9209	8728	8433	7564	11878	11920	12006	13248	Bacteria	Actinobacteria	Actinobacteria	Actinomycetales	Corynebacteriaceae	Corynebacterium	unclassified
33895	11661	12201	11485	10972	14261	14026	14021	14320	Bacteria	Actinobacteria	Actinobacteria	Actinomycetales	Corynebacteriaceae	Corynebacterium	unclassified
34124	10599	11253	10483	9446	13330	13446	13352	13744	Bacteria	Actinobacteria	Actinobacteria	Actinomycetales	Corynebacteriaceae	Corynebacterium	unclassified
34240	10319	10367	9868	8243	12755	12813	12536	13491	Bacteria	Actinobacteria	Actinobacteria	Actinomycetales	Corynebacteriaceae	Corynebacterium	unclassified
34246	9471	9897	7917	8008	13436	13371	13293	14082	Bacteria	Actinobacteria	Actinobacteria	Actinomycetales	Corynebacteriaceae	Corynebacterium	unclassified
34266	11633	12121	11950	11279	13315	13638	13311	13725	Bacteria	Actinobacteria	Actinobacteria	Actinomycetales	Corynebacteriaceae	Corynebacterium	unclassified
34318	10559	11700	9328	8406	13727	13778	14195	14177	Bacteria	Actinobacteria	Actinobacteria	Actinomycetales	Corynebacteriaceae	Corynebacterium	unclassified
34389	12411	12911	12439	11149	14537	14472	14436	14753	Bacteria	Actinobacteria	Actinobacteria	Actinomycetales	Corynebacteriaceae	Corynebacterium	unclassified
34438	11142	12071	11068	10083	13718	13811	13659	13979	Bacteria	Actinobacteria	Actinobacteria	Actinomycetales	Corynebacteriaceae	Corynebacterium	unclassified
34464	12846	13241	12895	11919	13530	13545	13516	13993	Bacteria	Actinobacteria	Actinobacteria	Actinomycetales	Corynebacteriaceae	Corynebacterium	unclassified
34708	12165	12920	11517	11465	14499	14412	14623	14568	Bacteria	Actinobacteria	Actinobacteria	Actinomycetales	Corynebacteriaceae	Corynebacterium	unclassified
34741	11617	11942	12138	10760	12347	12471	12312	12656	Bacteria	Actinobacteria	Actinobacteria	Actinomycetales	Corynebacteriaceae	Corynebacterium	unclassified
34803	8584	6937	6219	7413	12443	12941	13149	13813	Bacteria	Actinobacteria	Actinobacteria	Actinomycetales	Corynebacteriaceae	Corynebacterium	unclassified
35009	12382	12781	11656	11667	14461	14219	14250	14655	Bacteria	Actinobacteria	Actinobacteria	Actinomycetales	Corynebacteriaceae	Corynebacterium	unclassified
35030	12595	13334	11862	11812	14183	14120	14158	14453	Bacteria	Actinobacteria	Actinobacteria	Actinomycetales	Corynebacteriaceae	Corynebacterium	unclassified
35066	11695	12390	11683	10728	13792	13892	13649	13975	Bacteria	Actinobacteria	Actinobacteria	Actinomycetales	Corynebacteriaceae	Corynebacterium	unclassified
35165	12689	13478	12568	12571	14654	14331	14406	14675	Bacteria	Actinobacteria	Actinobacteria	Actinomycetales	Corynebacteriaceae	Corynebacterium	unclassified
35183	8734	6675	6257	6892	12466	12557	12666	13995	Bacteria	Actinobacteria	Actinobacteria	Actinomycetales	Corynebacteriaceae	Corynebacterium	unclassified
35227	9409	10954	7051	7631	11448	12622	12162	12539	Bacteria	Actinobacteria	Actinobacteria	Actinomycetales	Corynebacteriaceae	Corynebacterium	unclassified
35235	13192	13848	13269	12185	14590	14490	14478	14777	Bacteria	Actinobacteria	Actinobacteria	Actinomycetales	Corynebacteriaceae	Corynebacterium	unclassified
35240	10976	12368	8816	9572	14429	14107	14334	14634	Bacteria	Actinobacteria	Actinobacteria	Actinomycetales	Corynebacteriaceae	Corynebacterium	unclassified
35254	12603	12667	12305	11592	14182	14011	13992	14311	Bacteria	Actinobacteria	Actinobacteria	Actinomycetales	Corynebacteriaceae	Corynebacterium	unclassified
35512	11294	11867	10516	10079	14155	14037	14037	14377	Bacteria	Actinobacteria	Actinobacteria	Actinomycetales	Corynebacteriaceae	Corynebacterium	unclassified
35586	8917	8721	6578	7186	12940	12847	12706	13729	Bacteria	Actinobacteria	Actinobacteria	Actinomycetales	Corynebacteriaceae	Corynebacterium	unclassified
35629	9431	8574	6459	7789	13246	12890	13197	14189	Bacteria	Actinobacteria	Actinobacteria	Actinomycetales	Corynebacteriaceae	Corynebacterium	unclassified
35716	9211	9444	8022	7953	12173	12399	12249	13095	Bacteria	Actinobacteria	Actinobacteria	Actinomycetales	Corynebacteriaceae	Corynebacterium	unclassified
35819	12146	13134	11335	11260	14471	14322	14637	14531	Bacteria	Actinobacteria	Actinobacteria	Actinomycetales	Corynebacteriaceae	Corynebacterium	unclassified
35860	11628	11402	5472	10412	14621	14194	14457	14512	Bacteria	Actinobacteria	Actinobacteria	Actinomycetales	Corynebacteriaceae	Corynebacterium	unclassified
36035	11334	11878	11682	9930	13715	13703	13622	13993	Bacteria	Actinobacteria	Actinobacteria	Actinomycetales	Corynebacteriaceae	Corynebacterium	unclassified
36089	8588	7786	6279	7288	12834	13075	13245	13965	Bacteria	Actinobacteria	Actinobacteria	Actinomycetales	Corynebacteriaceae	Corynebacterium	unclassified
36091	12031	12653	11840	10842	14181	14147	14094	14411	Bacteria	Actinobacteria	Actinobacteria	Actinomycetales	Corynebacteriaceae	Corynebacterium	unclassified
36137	12609	13103	12047	11995	14213	14211	14138	14492	Bacteria	Actinobacteria	Actinobacteria	Actinomycetales	Corynebacteriaceae	Corynebacterium	unclassified
36142	10472	11118	10542	9533	12990	13059	12886	13725	Bacteria	Actinobacteria	Actinobacteria	Actinomycetales	Corynebacteriaceae	Corynebacterium	unclassified
36231	8448	8320	6935	7095	12599	13009	12989	13535	Bacteria	Actinobacteria	Actinobacteria	Actinomycetales	Corynebacteriaceae	Corynebacterium	unclassified
36381	9577	8803	7288	6965	13476	13260	13495	13919	Bacteria	Actinobacteria	Actinobacteria	Actinomycetales	Corynebacteriaceae	Corynebacterium	unclassified
36510	10981	12104	10924	10874	14234	14324	14314	14444	Bacteria	Actinobacteria	Actinobacteria	Actinomycetales	Corynebacteriaceae	Corynebacterium	unclassified
36542	12432	12836	12729	12610	13107	12948	12974	13663	Bacteria	Actinobacteria	Actinobacteria	Actinomycetales	Corynebacteriaceae	Corynebacterium	unclassified
36693	11379	11661	11202	10423	13747	13694	13376	14034	Bacteria	Actinobacteria	Actinobacteria	Actinomycetales	Corynebacteriaceae	Corynebacterium	unclassified
36754	9378	10005	9507	8968	12112	12705	12459	12844	Bacteria	Actinobacteria	Actinobacteria	Actinomycetales	Corynebacteriaceae	Corynebacterium	unclassified
36759	9151	9456	8134	7584	13278	13071	13186	13896	Bacteria	Actinobacteria	Actinobacteria	Actinomycetales	Corynebacteriaceae	Corynebacterium	unclassified
36762	10358	9298	6308	7603	13306	13296	13526	14138	Bacteria	Actinobacteria	Actinobacteria	Actinomycetales	Corynebacteriaceae	Corynebacterium	unclassified
36763	10281	10447	7898	7704	12940	13265	13176	13539	Bacteria	Actinobacteria	Actinobacteria	Actinomycetales	Corynebacteriaceae	Corynebacterium	unclassified
36770	12582	12727	12186	11616	13477	13426	13188	13549	Bacteria	Actinobacteria	Actinobacteria	Actinomycetales	Corynebacteriaceae	Corynebacterium	unclassified
36772	9730	8856	7665	8005	13028	12917	13104	13768	Bacteria	Actinobacteria	Actinobacteria	Actinomycetales	Corynebacteriaceae	Corynebacterium	unclassified
36773	8609	8148	6892	7575	11974	12623	12416	12801	Bacteria	Actinobacteria	Actinobacteria	Actinomycetales	Corynebacteriaceae	Corynebacterium	unclassified
36785	9898	8652	6548	7367	12933	13243	13192	13892	Bacteria	Actinobacteria	Actinobacteria	Actinomycetales	Corynebacteriaceae	Corynebacterium	unclassified
36787	9059	9096	7526	7964	11166	12068	11678	12524	Bacteria	Actinobacteria	Actinobacteria	Actinomycetales	Corynebacteriaceae	Corynebacterium	unclassified
36803	11207	10882	10646	9134	13686	13635	13699	14195	Bacteria	Actinobacteria	Actinobacteria	Actinomycetales	Corynebacteriaceae	Corynebacterium	unclassified
36804	9113	7873	7267	7252	12700	12620	12640	13711	Bacteria	Actinobacteria	Actinobacteria	Actinomycetales	Corynebacteriaceae	Corynebacterium	unclassified
36823	9602	9446	7819	8237	11439	12201	11773	12223	Bacteria	Actinobacteria	Actinobacteria	Actinomycetales	Corynebacteriaceae	Corynebacterium	unclassified
36834	8720	8449	6599	7995	12526	12529	12612	13788	Bacteria	Actinobacteria	Actinobacteria	Actinomycetales	Corynebacteriaceae	Corynebacterium	unclassified
36837	9591	10417	9704	8956	12423	12727	12458	13007	Bacteria	Actinobacteria	Actinobacteria	Actinomycetales	Corynebacteriaceae	Corynebacterium	unclassified
36867	10227	10695	10255	9472	12814	12967	12947	13843	Bacteria	Actinobacteria	Actinobacteria	Actinomycetales	Corynebacteriaceae	Corynebacterium	unclassified
36869	8852	9315	6964	7755	12274	12740	12469	13044	Bacteria	Actinobacteria	Actinobacteria	Actinomycetales	Corynebacteriaceae	Corynebacterium	unclassified
36880	8991	8627	9109	7668	11632	11568	11469	12178	Bacteria	Actinobacteria	Actinobacteria	Actinomycetales	Corynebacteriaceae	Corynebacterium	unclassified
36895	9122	10277	6249	7533	12697	12759	12843	13758	Bacteria	Actinobacteria	Actinobacteria	Actinomycetales	Corynebacteriaceae	Corynebacterium	unclassified
36900	10022	9829	6957	8145	13211	13326	13361	13744	Bacteria	Actinobacteria	Actinobacteria	Actinomycetales	Corynebacteriaceae	Corynebacterium	unclassified
36906	9142	8508	7298	7976	12529	12638	12971	13474	Bacteria	Actinobacteria	Actinobacteria	Actinomycetales	Corynebacteriaceae	Corynebacterium	unclassified
36908	11270	11923	11041	8681	12410	12619	12799	13104	Bacteria	Actinobacteria	Actinobacteria	Actinomycetales	Corynebacteriaceae	Corynebacterium	unclassified
36911	9502	8673	6926	7156	13028	13247	13236	13791	Bacteria	Actinobacteria	Actinobacteria	Actinomycetales	Corynebacteriaceae	Corynebacterium	unclassified
36930	8229	6351	6095	6521	12531	12302	12488	14169	Bacteria	Actinobacteria	Actinobacteria	Actinomycetales	Corynebacteriaceae	Corynebacterium	unclassified
36931	9226	8890	7307	7806	11127	11937	11489	12166	Bacteria	Actinobacteria	Actinobacteria	Actinomycetales	Corynebacteriaceae	Corynebacterium	unclassified
36933	9783	9439	6348	7388	13109	13277	13215	13808	Bacteria	Actinobacteria	Actinobacteria	Actinomycetales	Corynebacteriaceae	Corynebacterium	unclassified
36937	10136	9253	6652	7715	13066	13155	13317	14042	Bacteria	Actinobacteria	Actinobacteria	Actinomycetales	Corynebacteriaceae	Corynebacterium	unclassified
36940	9505	9983	7678	7980	11756	12518	12001	12317	Bacteria	Actinobacteria	Actinobacteria	Actinomycetales	Corynebacteriaceae	Corynebacterium	unclassified
36943	10146	9273	7025	7722	13146	13112	13278	13956	Bacteria	Actinobacteria	Actinobacteria	Actinomycetales	Corynebacteriaceae	Corynebacterium	unclassified
36949	9532	10346	7711	8473	12169	12922	12389	12791	Bacteria	Actinobacteria	Actinobacteria	Actinomycetales	Corynebacteriaceae	Corynebacterium	unclassified
36955	10575	11218	10647	10804	12907	12941	12840	13365	Bacteria	Actinobacteria	Actinobacteria	Actinomycetales	Corynebacteriaceae	Corynebacterium	unclassified
36957	9149	8077	6969	7429	12805	12577	12861	13537	Bacteria	Actinobacteria	Actinobacteria	Actinomycetales	Corynebacteriaceae	Corynebacterium	unclassified
36960	9217	9292	6473	7233	13610	13181	13304	14445	Bacteria	Actinobacteria	Actinobacteria	Actinomycetales	Corynebacteriaceae	Corynebacterium	unclassified

Table S6 (part C): HybScores of OTUs that were called present in at least one of the samples. Taxonomic classifications are based on PhyloChip taxonomy (derived from GreenGenes). Please note, that data presented here was rank normalized before performing statisical analyses presented in the manuscript.

	Sample ID (PMA)				Sample ID				taxonomic classification of representative rOTU cluster						
OTU	UR_PMA	CO_PMA	CR8_PMA	CR5_PMA	UR	CO	CR8	CR5	kingdom	phylum	class	order	family	genus	species
36980	11038	11840	10630	9993	12980	13378	13163	13797	Bacteria	Actinobacteria	Actinobacteria	Actinomycetales	Corynebacteriaceae	Corynebacterium	unclassified
36983	9012	8392	7021	8014	11258	12006	11671	12191	Bacteria	Actinobacteria	Actinobacteria	Actinomycetales	Corynebacteriaceae	Corynebacterium	unclassified
36992	9917	10596	8822	8990	13864	13578	13529	13844	Bacteria	Actinobacteria	Actinobacteria	Actinomycetales	Corynebacteriaceae	Corynebacterium	unclassified
37002	9980	9615	6823	7457	13462	13481	13424	13879	Bacteria	Actinobacteria	Actinobacteria	Actinomycetales	Corynebacteriaceae	Corynebacterium	unclassified
37004	8635	9012	6394	7853	12554	12756	12528	13481	Bacteria	Actinobacteria	Actinobacteria	Actinomycetales	Corynebacteriaceae	Corynebacterium	unclassified
37007	8976	8328	6433	7060	12364	12430	12293	13113	Bacteria	Actinobacteria	Actinobacteria	Actinomycetales	Corynebacteriaceae	Corynebacterium	unclassified
37025	8329	7521	6914	7137	10594	12507	11331	11626	Bacteria	Actinobacteria	Actinobacteria	Actinomycetales	Corynebacteriaceae	Corynebacterium	unclassified
37029	11490	11582	10977	7832	13640	13682	13631	14079	Bacteria	Actinobacteria	Actinobacteria	Actinomycetales	Corynebacteriaceae	Corynebacterium	unclassified
37036	9677	9487	7271	7914	11462	12285	11667	12244	Bacteria	Actinobacteria	Actinobacteria	Actinomycetales	Corynebacteriaceae	Corynebacterium	unclassified
37046	9860	9379	9118	9565	12585	12653	12654	13568	Bacteria	Actinobacteria	Actinobacteria	Actinomycetales	Corynebacteriaceae	Corynebacterium	unclassified
37061	8851	7492	6405	6803	12868	12684	12659	14156	Bacteria	Actinobacteria	Actinobacteria	Actinomycetales	Corynebacteriaceae	Corynebacterium	unclassified
37071	9754	8759	6440	7532	12761	13007	13181	13709	Bacteria	Actinobacteria	Actinobacteria	Actinomycetales	Corynebacteriaceae	Corynebacterium	unclassified
37076	11521	12146	11997	11503	13821	13815	13657	13920	Bacteria	Actinobacteria	Actinobacteria	Actinomycetales	Corynebacteriaceae	Corynebacterium	unclassified
37086	7956	6428	6118	7031	11065	11757	11069	12254	Bacteria	Actinobacteria	Actinobacteria	Actinomycetales	Corynebacteriaceae	Corynebacterium	unclassified
37089	10317	9572	6623	8042	13332	13166	13393	14041	Bacteria	Actinobacteria	Actinobacteria	Actinomycetales	Corynebacteriaceae	Corynebacterium	unclassified
37097	11066	11769	9333	8517	14240	14058	14195	14695	Bacteria	Actinobacteria	Actinobacteria	Actinomycetales	Corynebacteriaceae	Corynebacterium	unclassified
37107	9829	9237	7005	7874	12883	13147	13190	13370	Bacteria	Actinobacteria	Actinobacteria	Actinomycetales	Corynebacteriaceae	Corynebacterium	unclassified
37124	10243	10054	9695	8207	12484	12637	12411	13113	Bacteria	Actinobacteria	Actinobacteria	Actinomycetales	Corynebacteriaceae	Corynebacterium	unclassified
37125	9746	9459	7201	7622	13031	13217	13196	13557	Bacteria	Actinobacteria	Actinobacteria	Actinomycetales	Corynebacteriaceae	Corynebacterium	unclassified
37129	10296	9556	7198	8125	13456	13302	13518	14166	Bacteria	Actinobacteria	Actinobacteria	Actinomycetales	Corynebacteriaceae	Corynebacterium	unclassified
37151	10156	9577	7348	7875	13251	13229	13452	14019	Bacteria	Actinobacteria	Actinobacteria	Actinomycetales	Corynebacteriaceae	Corynebacterium	unclassified
37161	8974	8228	6824	7330	12726	12825	12947	13747	Bacteria	Actinobacteria	Actinobacteria	Actinomycetales	Corynebacteriaceae	Corynebacterium	unclassified
37166	8125	7711	6732	7218	11907	11848	11678	13077	Bacteria	Actinobacteria	Actinobacteria	Actinomycetales	Corynebacteriaceae	Corynebacterium	unclassified
37170	9453	10135	7183	7745	12424	13001	12578	12791	Bacteria	Actinobacteria	Actinobacteria	Actinomycetales	Corynebacteriaceae	Corynebacterium	unclassified
37172	8286	8199	6921	7580	11844	12150	12224	12043	Bacteria	Actinobacteria	Actinobacteria	Actinomycetales	Corynebacteriaceae	Corynebacterium	unclassified
37178	10144	9922	6921	7620	12537	13148	12877	12966	Bacteria	Actinobacteria	Actinobacteria	Actinomycetales	Corynebacteriaceae	Corynebacterium	unclassified
37180	9158	8782	7043	7892	12866	12713	12147	13117	Bacteria	Actinobacteria	Actinobacteria	Actinomycetales	Corynebacteriaceae	Corynebacterium	unclassified
37183	10977	11454	9760	10160	12500	12620	12667	12931	Bacteria	Actinobacteria	Actinobacteria	Actinomycetales	Corynebacteriaceae	Corynebacterium	unclassified
37193	9800	10002	6949	7616	12884	13201	13088	13641	Bacteria	Actinobacteria	Actinobacteria	Actinomycetales	Corynebacteriaceae	Corynebacterium	unclassified
37198	11045	10510	10020	9906	13083	13212	13283	13585	Bacteria	Actinobacteria	Actinobacteria	Actinomycetales	Corynebacteriaceae	Corynebacterium	unclassified
37199	8988	8837	7148	7553	12118	12175	12078	13051	Bacteria	Actinobacteria	Actinobacteria	Actinomycetales	Corynebacteriaceae	Corynebacterium	unclassified
37201	10039	9174	5866	6263	12339	12628	12505	13802	Bacteria	Actinobacteria	Actinobacteria	Actinomycetales	Corynebacteriaceae	Corynebacterium	unclassified
37226	9315	8626	5638	7035	12098	12388	12323	12884	Bacteria	Actinobacteria	Actinobacteria	Actinomycetales	Corynebacteriaceae	Corynebacterium	unclassified
37232	10078	9557	7009	7598	13084	13288	13298	13739	Bacteria	Actinobacteria	Actinobacteria	Actinomycetales	Corynebacteriaceae	Corynebacterium	unclassified
37253	11355	11950	11400	11307	13420	13714	13542	13737	Bacteria	Actinobacteria	Actinobacteria	Actinomycetales	Corynebacteriaceae	Corynebacterium	unclassified
37255	9608	8594	7817	7653	12903	13093	13203	13613	Bacteria	Actinobacteria	Actinobacteria	Actinomycetales	Corynebacteriaceae	Corynebacterium	unclassified
37259	10173	10754	8872	8052	12809	13163	12811	13739	Bacteria	Actinobacteria	Actinobacteria	Actinomycetales	Corynebacteriaceae	Corynebacterium	unclassified
37264	9249	9641	7453	7653	11102	11971	11452	11960	Bacteria	Actinobacteria	Actinobacteria	Actinomycetales	Corynebacteriaceae	Corynebacterium	unclassified
37268	9330	9481	7481	8080	11337	12095	11636	12108	Bacteria	Actinobacteria	Actinobacteria	Actinomycetales	Corynebacteriaceae	Corynebacterium	unclassified
37293	9380	8972	8939	8019	12037	12361	12128	12607	Bacteria	Actinobacteria	Actinobacteria	Actinomycetales	Corynebacteriaceae	Corynebacterium	unclassified
37300	10608	10689	9967	8787	13657	13544	13734	14299	Bacteria	Actinobacteria	Actinobacteria	Actinomycetales	Corynebacteriaceae	Corynebacterium	unclassified
37315	9461	9038	6173	6944	13024	13290	13320	13958	Bacteria	Actinobacteria	Actinobacteria	Actinomycetales	Corynebacteriaceae	Corynebacterium	unclassified
37316	9147	8576	6898	7944	12420	12552	12585	13239	Bacteria	Actinobacteria	Actinobacteria	Actinomycetales	Corynebacteriaceae	Corynebacterium	unclassified
37325	9759	9201	7083	7364	12766	13116	13078	13521	Bacteria	Actinobacteria	Actinobacteria	Actinomycetales	Corynebacteriaceae	Corynebacterium	unclassified
37329	8951	8633	7396	7341	12241	12561	12353	13165	Bacteria	Actinobacteria	Actinobacteria	Actinomycetales	Corynebacteriaceae	Corynebacterium	unclassified
37358	10234	8682	6445	7588	13204	13358	13459	14085	Bacteria	Actinobacteria	Actinobacteria	Actinomycetales	Corynebacteriaceae	Corynebacterium	unclassified
37364	9143	8097	7282	8081	11507	11986	11905	12524	Bacteria	Actinobacteria	Actinobacteria	Actinomycetales	Corynebacteriaceae	Corynebacterium	unclassified
37365	11132	11329	10839	9325	13756	13704	13709	14230	Bacteria	Actinobacteria	Actinobacteria	Actinomycetales	Corynebacteriaceae	Corynebacterium	unclassified
37394	11178	11531	10751	9793	12400	12674	12023	12630	Bacteria	Actinobacteria	Actinobacteria	Actinomycetales	Corynebacteriaceae	unclassified	unclassified
37429	11201	11510	10935	10100	12624	12905	12575	13296	Bacteria	Actinobacteria	Actinobacteria	Actinomycetales	Corynebacteriaceae	unclassified	unclassified
37494	11116	11328	10481	9635	12126	12959	12394	13110	Bacteria	Actinobacteria	Actinobacteria	Actinomycetales	Corynebacteriaceae	unclassified	unclassified
37576	11458	11533	10720	9804	12764	13109	12758	13205	Bacteria	Actinobacteria	Actinobacteria	Actinomycetales	Corynebacteriaceae	unclassified	unclassified
37579	11130	11515	10549	9654	12816	13014	12741	13213	Bacteria	Actinobacteria	Actinobacteria	Actinomycetales	Corynebacteriaceae	unclassified	unclassified
37609	11619	12097	10993	10158	11468	12465	11658	12315	Bacteria	Actinobacteria	Actinobacteria	Actinomycetales	Corynebacteriaceae	unclassified	unclassified
37735	11003	11418	10551	9651	12046	12525	12191	12476	Bacteria	Actinobacteria	Actinobacteria	Actinomycetales	Corynebacteriaceae	unclassified	unclassified
37783	11098	10965	10917	10130	13030	13074	12682	13396	Bacteria	Actinobacteria	Actinobacteria	Actinomycetales	Corynebacteriaceae	unclassified	unclassified
37813	11499	11824	11107	10240	12341	12917	12416	13058	Bacteria	Actinobacteria	Actinobacteria	Actinomycetales	Corynebacteriaceae	unclassified	unclassified
37820	9193	9339	7901	7934	12095	12910	12283	12856	Bacteria	Actinobacteria	Actinobacteria	Actinomycetales	Corynebacteriaceae	unclassified	unclassified
37833	11140	11297	10721	9679	10927	11947	11258	11831	Bacteria	Actinobacteria	Actinobacteria	Actinomycetales	Corynebacteriaceae	unclassified	unclassified
37860	11022	11313	10518	9912	11392	12154	11634	12184	Bacteria	Actinobacteria	Actinobacteria	Actinomycetales	Corynebacteriaceae	unclassified	unclassified
37877	11809	12259	11554	10472	13309	13600	13386	13816	Bacteria	Actinobacteria	Actinobacteria	Actinomycetales	Corynebacteriaceae	unclassified	unclassified
37902	11148	11678	10951	9840	11858	12973	12364	12383	Bacteria	Actinobacteria	Actinobacteria	Actinomycetales	Corynebacteriaceae	unclassified	unclassified
38020	9596	9651	9492	8500	13140	13258	13243	13720	Bacteria	Actinobacteria	Actinobacteria	Actinomycetales	Corynebacteriaceae	unclassified	unclassified
38164	9740	9454	8419	8804	13265	13477	13272	13769	Bacteria	Actinobacteria	Actinobacteria	Actinomycetales	Corynebacteriaceae	unclassified	unclassified
38210	11153	11400	10868	9824	12042	12716	12172	12774	Bacteria	Actinobacteria	Actinobacteria	Actinomycetales	Corynebacteriaceae	unclassified	unclassified
38281	11955	11568	10512	9767	12574	13092	12718	13243	Bacteria	Actinobacteria	Actinobacteria	Actinomycetales	Corynebacteriaceae	unclassified	unclassified
38393	11279	11461	10644	9883	12319	12895	12431	13134	Bacteria	Actinobacteria	Actinobacteria	Actinomycetales	Corynebacteriaceae	unclassified	unclassified
38432	11118	11321	10751	10337	12948	12969	13021	13562	Bacteria	Actinobacteria	Actinobacteria	Actinomycetales	Corynebacteriaceae	unclassified	unclassified
38433	11499	11824	11107	10240	12341	12917	12416	13058	Bacteria	Actinobacteria	Actinobacteria	Actinomycetales	Corynebacteriaceae	unclassified	unclassified
40156	8376	8606	7155	6888	11838	12382	12037	12898	Bacteria	Actinobacteria	Actinobacteria	Actinomycetales	Dermabacteraceae	Dermabacter	Dermabacter hominis
40170	9106	9151	6930	7059	12949	12978	13029	13964	Bacteria	Actinobacteria	Actinobacteria	Actinomycetales	Dermabacteraceae	Dermabacter	Dermabacter hominis
40220	8810	8600	7724	8239	12074	12036	12138	13517	Bacteria	Actinobacteria	Actinobacteria	Actinomycetales	Dermabacteraceae	Dermabacter	Dermabacter hominis
40378	9484	8811	7932	8316	12724	12793	12596	13500	Bacteria	Actinobacteria	Actinobacteria	Actinomycetales	Dermabacteraceae	Dermabacter	Dermabacter hominis
40893	9693	9752	6938	7231	13083	13150	13179	13890	Bacteria	Actinobacteria	Actinobacteria	Actinomycetales	Dermabacteraceae	Dermabacter	Dermabacter hominis
40389	8709	10572	7228	7252	12616	12717	12523	13564	Bacteria	Actinobacteria	Actinobacteria	Actinomycetales	Dermabacteraceae	Dermabacter	unclassified
40391	9309	10053	7066	8232	12807	13029	12788	13505	Bacteria	Actinobacteria	Actinobacteria	Actinomycetales	Dermabacteraceae	Dermabacter	unclassified
40402	9679	9046	6570	7345	12846	12895	12855	13394	Bacteria	Actinobacteria	Actinobacteria	Actinomycetales	Dermabacteraceae	Dermabacter	unclassified
40529	9253	10436	6980	7252	12649	12754	12630	13716	Bacteria	Actinobacteria	Actinobacteria	Actinomycetales	Dermabacteraceae	Dermabacter	unclassified
40531	9407	10265	6426	7158	12713	12790	12716	13710	Bacteria	Actinobacteria	Actinobacteria	Actinomycetales	Dermabacteraceae	Dermabacter	unclassified
40899	10556	11689	10473	10404	12675	12897	12611	13655	Bacteria	Actinobacteria	Actinobacteria	Actinomycetales	Dermabacteraceae	Dermabacter	unclassified
40908	9415	10649	6955	7239	12718	12850	12724	13753	Bacteria	Actinobacteria	Actinobacteria	Actinomycetales	Dermabacteraceae	Dermabacter	unclassified
40996	9416	10523	7397	7664	12702	12815	12694	13785	Bacteria	Actinobacteria	Actinobacteria	Actinomycetales	Dermabacteraceae	Dermabacter	unclassified
41018	9615	10721	6964	8005	12929	13083	12939	13583	Bacteria	Actinobacteria	Actinobacteria	Actinomycetales	Dermabacteraceae	Dermabacter	unclassified
40426	8728	8317	6911	7655	12332	11882	12666	12662	Bacteria	Actinobacteria	Actinobacteria	Actinomycetales	Dermacoccaceae	Kytococcus	Kytococcus schroeteri
40278	7849	7411	6086	6329	12254	11781	12616	12611	Bacteria	Actinobacteria	Actinobacteria	Actinomycetales	Dermacoccaceae	Kytococcus	unclassified
40408	9308	8921	7595	8478	12564	12026	12537	12974	Bacteria	Actinobacteria	Actinobacteria	Actinomycetales	Dermacoccaceae	Kytococcus	unclassified
41016	9780	9729	7907	9077	12508	12275	12891	12760	Bacteria	Actinobacteria	Actinobacteria	Actinomycetales	Dermacoccaceae	Kytococcus	unclassified
37598	9513	10050	8937	8296	11910	13184	12232	12399	Bacteria	Actinobacteria	Actinobacteria	Actinomycetales	Frankiaceae	Frankia	unclassified
37814	8802	8219	6988	7341	11474	12756	11795	12241	Bacteria	Actinobacteria	Actinobacteria	Actinomycetales	Frankiaceae	Frankia	unclassified
38072	9904	9175	9016	8827	11699	12562	11575	12214	Bacteria	Actinobacteria	Actinobacteria	Actinomycetales	Frankiaceae	Frankia	unclassified
38101	9573	10640	8194	9679	12579	13302	12373	12772	Bacteria	Actinobacteria	Actinobacteria	Actinomycetales	Frankiaceae	Frankia	unclassified
38266	9469	10612	8340	8677	11928	13304	12135	12510	Bacteria	Actinobacteria	Actinobacteria	Actinomycetales	Frankiaceae	Frankia	unclassified
37549	8510	8223	6525	7618	10603	12052	10595	11215	Bacteria	Actinobacteria	Actinobacteria	Actinomycetales	Frankiaceae	unclassified	unclassified
39060	8269	9186	7012	7215	11208	12628	11871	12069	Bacteria	Actinobacteria	Actinobacteria	Actinomycetales	Geodermatophilaceae	Blastococcus	Blastococcus aggregatus
39124	7964	9234	6363	7397	11796	13012	12350	12676	Bacteria	Actinobacteria	Actinobacteria	Actinomycetales	Geodermatophilaceae	Blastococcus	Blastococcus aggregatus
39484	7868	9213	6007	6793	11762	12949	12317	12664	Bacteria	Actinobacteria	Actinobacteria	Actinomycetales	Geodermatophilaceae	Blastococcus	Blastococcus aggregatus
38463	9001	9520	7249	7791	12218	13532	12659	13074	Bacteria	Actinobacteria	Actinobacteria	Actinomycetales	Geodermatophilaceae	Geodermatophilus	Blastococcus saxobsidens
38726	9683	10717	7984	8560	12835	13572	12880	13190	Bacteria	Actinobacteria	Actinobacteria	Actinomycetales	Geodermatophilaceae	Geodermatophilus	unclassified
39054	8829	9423	7530	8039	11510	12875	12043	11943	Bacteria	Actinobacteria	Actinobacteria	Actinomycetales	Geodermatophilaceae	Geodermatophilus	unclassified
39208	10084	9333	7047	7664	12299	13171	12660	12921	Bacteria	Actinobacteria	Actinobacteria	Actinomycetales	Geodermatophilaceae	Geodermatophilus	unclassified

Table S6 (part D): HybScores of OTUs that were called present in at least one of the samples. Taxonomic classifications are based on PhyloChip taxonomy (derived from GreenGenes). Please note, that data presented here was rank normalized before performing statisical analyses presented in the manuscript.

	Sample ID (PMA)				Sample ID				taxonomic classification of representative rOTU cluster						
OTU	UR_PMA	CO_PMA	CR8_PMA	CR5_PMA	UR	CO	CR8	CR5	kingdom	phylum	class	order	family	genus	species
39926	8677	9470	6668	7907	11920	13359	12353	12904	Bacteria	Actinobacteria	Actinobacteria	Actinomycetales	Geodermatophilaceae	Geodermatophilus	unclassified
40055	9147	9107	6733	8076	11400	12365	12149	11890	Bacteria	Actinobacteria	Actinobacteria	Actinomycetales	Geodermatophilaceae	Geodermatophilus	unclassified
40076	8548	9240	6355	7726	12011	13283	12416	13114	Bacteria	Actinobacteria	Actinobacteria	Actinomycetales	Geodermatophilaceae	Geodermatophilus	unclassified
38640	8545	9373	6839	7607	11985	13368	12574	12811	Bacteria	Actinobacteria	Actinobacteria	Actinomycetales	Geodermatophilaceae	unclassified	unclassified
39558	8981	9545	6635	7828	11778	12953	12064	12143	Bacteria	Actinobacteria	Actinobacteria	Actinomycetales	Geodermatophilaceae	unclassified	unclassified
39644	10098	9565	8330	8380	11862	13038	12232	12934	Bacteria	Actinobacteria	Actinobacteria	Actinomycetales	Geodermatophilaceae	unclassified	unclassified
39661	9178	9799	8527	8966	11689	13087	11765	12334	Bacteria	Actinobacteria	Actinobacteria	Actinomycetales	Geodermatophilaceae	unclassified	unclassified
39857	9325	9657	7212	8004	11139	12714	11667	12063	Bacteria	Actinobacteria	Actinobacteria	Actinomycetales	Geodermatophilaceae	unclassified	unclassified
39910	8901	9943	6252	7683	12474	13699	12849	13254	Bacteria	Actinobacteria	Actinobacteria	Actinomycetales	Geodermatophilaceae	unclassified	unclassified
40039	8788	9882	6593	7390	11204	12818	11264	11997	Bacteria	Actinobacteria	Actinobacteria	Actinomycetales	Geodermatophilaceae	unclassified	unclassified
38103	8430	7158	5985	6638	11522	12355	11568	12303	Bacteria	Actinobacteria	Actinobacteria	Actinomycetales	Gordoniaceae	Gordonia	Gordonia amarae
37449	8684	6898	6211	6704	11934	12427	11701	12985	Bacteria	Actinobacteria	Actinobacteria	Actinomycetales	Gordoniaceae	Gordonia	unclassified
37622	8578	8788	6535	7111	12097	12740	12236	13291	Bacteria	Actinobacteria	Actinobacteria	Actinomycetales	Gordoniaceae	Gordonia	unclassified
37653	9556	9778	6482	7245	12508	12890	12720	13346	Bacteria	Actinobacteria	Actinobacteria	Actinomycetales	Gordoniaceae	Gordonia	unclassified
38270	8692	7385	5960	6674	11709	12556	11731	12408	Bacteria	Actinobacteria	Actinobacteria	Actinomycetales	Gordoniaceae	Gordonia	unclassified
40856	10315	10414	7345	8071	12192	12911	12312	12478	Bacteria	Actinobacteria	Actinobacteria	Actinomycetales	Intrasporangiaceae	Janibacter	unclassified
40578	8961	9450	7068	7207	13092	13703	12989	13781	Bacteria	Actinobacteria	Actinobacteria	Actinomycetales	Intrasporangiaceae	Knoellia	Knoellia subterranea
40705	8799	10308	6710	6698	12410	13429	12417	12571	Bacteria	Actinobacteria	Actinobacteria	Actinomycetales	Intrasporangiaceae	Knoellia	Knoellia subterranea
40505	11777	12149	10898	10019	12724	13513	12574	12998	Bacteria	Actinobacteria	Actinobacteria	Actinomycetales	Intrasporangiaceae	unclassified	unclassified
40592	8505	9166	6354	6776	11799	12719	11969	12685	Bacteria	Actinobacteria	Actinobacteria	Actinomycetales	Intrasporangiaceae	unclassified	unclassified
40629	9580	10761	9164	7848	11855	13281	11934	12349	Bacteria	Actinobacteria	Actinobacteria	Actinomycetales	Intrasporangiaceae	unclassified	unclassified
40716	10211	9823	7438	6904	13215	13315	13088	13168	Bacteria	Actinobacteria	Actinobacteria	Actinomycetales	Intrasporangiaceae	unclassified	unclassified
40749	10451	11627	8217	8053	12839	13506	12739	13192	Bacteria	Actinobacteria	Actinobacteria	Actinomycetales	Intrasporangiaceae	unclassified	unclassified
40976	10596	11138	9864	9452	13052	13653	12916	13355	Bacteria	Actinobacteria	Actinobacteria	Actinomycetales	Intrasporangiaceae	unclassified	unclassified
40984	9381	8288	6327	6810	12019	12650	11746	12263	Bacteria	Actinobacteria	Actinobacteria	Actinomycetales	Intrasporangiaceae	unclassified	unclassified
41001	9985	9513	8600	9210	12103	12814	11913	12151	Bacteria	Actinobacteria	Actinobacteria	Actinomycetales	Intrasporangiaceae	unclassified	unclassified
41060	8973	9979	6386	6746	12260	13048	12029	12377	Bacteria	Actinobacteria	Actinobacteria	Actinomycetales	Intrasporangiaceae	unclassified	unclassified
41078	9003	10024	7025	7807	11403	12782	11811	11846	Bacteria	Actinobacteria	Actinobacteria	Actinomycetales	Intrasporangiaceae	unclassified	unclassified
37917	8363	8707	6225	6674	10942	12018	11601	11897	Bacteria	Actinobacteria	Actinobacteria	Actinomycetales	Kineosporiaceae	Kineococcus	Kineococcus radiotolerans
37763	8425	8342	6163	6380	12031	12835	12655	12610	Bacteria	Actinobacteria	Actinobacteria	Actinomycetales	Kineosporiaceae	Kineococcus	unclassified
37881	8911	10936	6506	6950	12552	13319	12797	13071	Bacteria	Actinobacteria	Actinobacteria	Actinomycetales	Kineosporiaceae	Kineococcus	unclassified
37566	10388	12054	8459	8453	13224	13933	13287	13657	Bacteria	Actinobacteria	Actinobacteria	Actinomycetales	Kineosporiaceae	unclassified	unclassified
37920	8948	10213	6484	7663	11824	12998	11868	12410	Bacteria	Actinobacteria	Actinobacteria	Actinomycetales	Kineosporiaceae	unclassified	unclassified
38083	10556	10658	10656	8842	10948	12068	11124	11602	Bacteria	Actinobacteria	Actinobacteria	Actinomycetales	Kineosporiaceae	unclassified	unclassified
41978	11147	11454	11037	10367	12586	13243	12935	12764	Bacteria	Actinobacteria	Actinobacteria	Actinomycetales	Microbacteriaceae	Agreia	unclassified
42208	8979	9061	7040	7484	11580	12964	11811	11820	Bacteria	Actinobacteria	Actinobacteria	Actinomycetales	Microbacteriaceae	Agreia	unclassified
41369	8667	8586	6478	7878	11339	12796	11423	11329	Bacteria	Actinobacteria	Actinobacteria	Actinomycetales	Microbacteriaceae	Agrococcus	Agrococcus jenensis
38524	10551	11242	10241	8977	12347	13162	12749	12606	Bacteria	Actinobacteria	Actinobacteria	Actinomycetales	Microbacteriaceae	Agromyces	unclassified
38721	9073	10058	8002	7541	11395	12670	11983	12204	Bacteria	Actinobacteria	Actinobacteria	Actinomycetales	Microbacteriaceae	Agromyces	unclassified
38848	9779	10226	7193	9395	11846	12958	12300	12312	Bacteria	Actinobacteria	Actinobacteria	Actinomycetales	Microbacteriaceae	Agromyces	unclassified
39094	8989	8077	6268	7720	10024	11718	11432	10768	Bacteria	Actinobacteria	Actinobacteria	Actinomycetales	Microbacteriaceae	Agromyces	unclassified
39225	10300	9575	8450	8610	12083	12891	12125	12445	Bacteria	Actinobacteria	Actinobacteria	Actinomycetales	Microbacteriaceae	Agromyces	unclassified
39242	8354	9821	6501	7633	10949	12308	11683	11565	Bacteria	Actinobacteria	Actinobacteria	Actinomycetales	Microbacteriaceae	Agromyces	unclassified
39259	8384	9764	6873	7405	11877	12830	12417	12308	Bacteria	Actinobacteria	Actinobacteria	Actinomycetales	Microbacteriaceae	Agromyces	unclassified
39733	11474	12133	11274	9747	13069	13728	13228	13556	Bacteria	Actinobacteria	Actinobacteria	Actinomycetales	Microbacteriaceae	Agromyces	unclassified
40014	8107	7690	6417	7340	10969	12335	11752	11657	Bacteria	Actinobacteria	Actinobacteria	Actinomycetales	Microbacteriaceae	Agromyces	unclassified
40067	9216	9229	6992	7356	11937	12822	12229	12487	Bacteria	Actinobacteria	Actinobacteria	Actinomycetales	Microbacteriaceae	Agromyces	unclassified
41399	9273	9832	5881	7600	11831	13271	11974	12322	Bacteria	Actinobacteria	Actinobacteria	Actinomycetales	Microbacteriaceae	Leucobacter	unclassified
41760	8572	10112	7458	7458	11572	13196	11746	12469	Bacteria	Actinobacteria	Actinobacteria	Actinomycetales	Microbacteriaceae	Leucobacter	unclassified
41938	9230	9072	7399	7321	11737	12899	11906	12119	Bacteria	Actinobacteria	Actinobacteria	Actinomycetales	Microbacteriaceae	Leucobacter	unclassified
41964	9384	10408	9250	8121	11688	12933	12112	11931	Bacteria	Actinobacteria	Actinobacteria	Actinomycetales	Microbacteriaceae	Microbacterium	Microbacterium aurum
42310	9507	11462	10061	8752	11820	13184	12394	12286	Bacteria	Actinobacteria	Actinobacteria	Actinomycetales	Microbacteriaceae	Microbacterium	Microbacterium aurum
41088	8766	7539	6413	7846	11887	12693	12149	12480	Bacteria	Actinobacteria	Actinobacteria	Actinomycetales	Microbacteriaceae	Microbacterium	unclassified
41101	9239	9091	6764	8765	12384	12812	12521	12563	Bacteria	Actinobacteria	Actinobacteria	Actinomycetales	Microbacteriaceae	Microbacterium	unclassified
41123	9232	10016	5957	8796	12709	13359	12742	13272	Bacteria	Actinobacteria	Actinobacteria	Actinomycetales	Microbacteriaceae	Microbacterium	unclassified
41163	8752	8817	7115	7917	10653	11808	11306	11022	Bacteria	Actinobacteria	Actinobacteria	Actinomycetales	Microbacteriaceae	Microbacterium	unclassified
41171	9150	9222	6439	8770	12356	13097	12264	12454	Bacteria	Actinobacteria	Actinobacteria	Actinomycetales	Microbacteriaceae	Microbacterium	unclassified
41275	9006	8394	6478	7703	12339	13107	12462	12654	Bacteria	Actinobacteria	Actinobacteria	Actinomycetales	Microbacteriaceae	Microbacterium	unclassified
41284	8737	7473	6311	7456	12041	12914	12197	12408	Bacteria	Actinobacteria	Actinobacteria	Actinomycetales	Microbacteriaceae	Microbacterium	unclassified
41293	9911	10006	6768	9887	12939	13671	12766	13291	Bacteria	Actinobacteria	Actinobacteria	Actinomycetales	Microbacteriaceae	Microbacterium	unclassified
41373	9703	8456	7615	8810	12008	12499	12181	12255	Bacteria	Actinobacteria	Actinobacteria	Actinomycetales	Microbacteriaceae	Microbacterium	unclassified
41456	10819	11380	9673	8791	12858	13431	13227	12873	Bacteria	Actinobacteria	Actinobacteria	Actinomycetales	Microbacteriaceae	Microbacterium	unclassified
41460	8844	8435	6398	8440	12011	12938	11969	12261	Bacteria	Actinobacteria	Actinobacteria	Actinomycetales	Microbacteriaceae	Microbacterium	unclassified
41462	9339	8538	7273	8126	12245	12884	12209	12615	Bacteria	Actinobacteria	Actinobacteria	Actinomycetales	Microbacteriaceae	Microbacterium	unclassified
41474	9086	8147	6746	7729	11920	12859	12045	12364	Bacteria	Actinobacteria	Actinobacteria	Actinomycetales	Microbacteriaceae	Microbacterium	unclassified
41491	9159	7907	6153	8181	12018	13095	12175	12087	Bacteria	Actinobacteria	Actinobacteria	Actinomycetales	Microbacteriaceae	Microbacterium	unclassified
41493	9459	6839	6752	7514	11961	12639	11861	11925	Bacteria	Actinobacteria	Actinobacteria	Actinomycetales	Microbacteriaceae	Microbacterium	unclassified
41499	8907	7442	6635	7747	11886	12584	11922	12045	Bacteria	Actinobacteria	Actinobacteria	Actinomycetales	Microbacteriaceae	Microbacterium	unclassified
41517	9716	9580	8364	9254	12395	12818	12081	12678	Bacteria	Actinobacteria	Actinobacteria	Actinomycetales	Microbacteriaceae	Microbacterium	unclassified
41535	9012	8633	6657	7984	12324	13018	12194	12584	Bacteria	Actinobacteria	Actinobacteria	Actinomycetales	Microbacteriaceae	Microbacterium	unclassified
41546	11042	12075	7005	8655	13612	14116	13720	13948	Bacteria	Actinobacteria	Actinobacteria	Actinomycetales	Microbacteriaceae	Microbacterium	unclassified
41578	9204	8495	6629	8165	11183	12204	11489	11365	Bacteria	Actinobacteria	Actinobacteria	Actinomycetales	Microbacteriaceae	Microbacterium	unclassified
41601	9020	8774	6118	7508	11879	12309	11778	11892	Bacteria	Actinobacteria	Actinobacteria	Actinomycetales	Microbacteriaceae	Microbacterium	unclassified
41637	8883	7355	6778	7620	11077	12381	11243	11409	Bacteria	Actinobacteria	Actinobacteria	Actinomycetales	Microbacteriaceae	Microbacterium	unclassified
41657	9576	8071	7465	7848	12548	13326	12548	12799	Bacteria	Actinobacteria	Actinobacteria	Actinomycetales	Microbacteriaceae	Microbacterium	unclassified
41712	8902	8295	6654	7235	12386	13154	12416	12852	Bacteria	Actinobacteria	Actinobacteria	Actinomycetales	Microbacteriaceae	Microbacterium	unclassified
41721	9823	9720	7373	9073	12643	13267	12676	12864	Bacteria	Actinobacteria	Actinobacteria	Actinomycetales	Microbacteriaceae	Microbacterium	unclassified
41749	8726	7145	6418	7727	11599	12387	11686	11768	Bacteria	Actinobacteria	Actinobacteria	Actinomycetales	Microbacteriaceae	Microbacterium	unclassified
41753	10363	11308	8069	9071	12684	13328	12886	12956	Bacteria	Actinobacteria	Actinobacteria	Actinomycetales	Microbacteriaceae	Microbacterium	unclassified
41840	9433	8297	7355	8430	12179	12732	12293	12559	Bacteria	Actinobacteria	Actinobacteria	Actinomycetales	Microbacteriaceae	Microbacterium	unclassified
41853	8689	7501	7598	8238	11711	12242	11612	12011	Bacteria	Actinobacteria	Actinobacteria	Actinomycetales	Microbacteriaceae	Microbacterium	unclassified
41854	9067	7664	7111	8083	11712	12592	11835	11918	Bacteria	Actinobacteria	Actinobacteria	Actinomycetales	Microbacteriaceae	Microbacterium	unclassified
41881	8824	7618	6650	7317	12197	12917	12287	12818	Bacteria	Actinobacteria	Actinobacteria	Actinomycetales	Microbacteriaceae	Microbacterium	unclassified
41904	9506	9213	6847	7333	12906	13676	12976	13376	Bacteria	Actinobacteria	Actinobacteria	Actinomycetales	Microbacteriaceae	Microbacterium	unclassified
41908	11088	11658	9793	8893	12447	13243	12781	12603	Bacteria	Actinobacteria	Actinobacteria	Actinomycetales	Microbacteriaceae	Microbacterium	unclassified
41946	9300	9376	6497	8731	12362	13070	12337	12568	Bacteria	Actinobacteria	Actinobacteria	Actinomycetales	Microbacteriaceae	Microbacterium	unclassified
42038	9712	8549	7198	8473	12849	13103	12866	13551	Bacteria	Actinobacteria	Actinobacteria	Actinomycetales	Microbacteriaceae	Microbacterium	unclassified
42065	9281	9005	6058	7236	11988	12681	11805	12214	Bacteria	Actinobacteria	Actinobacteria	Actinomycetales	Microbacteriaceae	Microbacterium	unclassified
42104	8824	9741	6175	7590	11806	13062	11931	12091	Bacteria	Actinobacteria	Actinobacteria	Actinomycetales	Microbacteriaceae	Microbacterium	unclassified
42149	10192	11003	7086	9991	12890	13535	12965	13014	Bacteria	Actinobacteria	Actinobacteria	Actinomycetales	Microbacteriaceae	Microbacterium	unclassified
42173	9888	10368	6968	8277	12911	13368	13075	13527	Bacteria	Actinobacteria	Actinobacteria	Actinomycetales	Microbacteriaceae	Microbacterium	unclassified
42236	10048	8154	6842	9464	12081	12870	12303	12409	Bacteria	Actinobacteria	Actinobacteria	Actinomycetales	Microbacteriaceae	Microbacterium	unclassified
42295	9496	9186	6903	9008	12660	13206	12584	12879	Bacteria	Actinobacteria	Actinobacteria	Actinomycetales	Microbacteriaceae	Microbacterium	unclassified
42417	9311	8093	6188	8777	12226	12996	12245	12473	Bacteria	Actinobacteria	Actinobacteria	Actinomycetales	Microbacteriaceae	Microbacterium	unclassified
42432	9652	9966	7093	7599	11548	12851	11712	11769	Bacteria	Actinobacteria	Actinobacteria	Actinomycetales	Microbacteriaceae	Microbacterium	unclassified
42091	8742	7389	6173	6658	11740	13690	12197	11803	Bacteria	Actinobacteria	Actinobacteria	Actinomycetales	Microbacteriaceae	Mycetocola	Mycetocola reblochoni
42337	8735	9556	7595	8541	12102	12957	12478	12576	Bacteria	Actinobacteria	Actinobacteria	Actinomycetales	Microbacteriaceae	Rathayibacter	Rathayibacter caricis
41209	8221	7857	6118	7197	11598	12613	12186	11961	Bacteria	Actinobacteria	Actinobacteria	Actinomycetales	Microbacteriaceae	unclassified	unclassified
41250	9594	10265	8529	7782	12196	13077	12466	12659	Bacteria	Actinobacteria	Actinobacteria	Actinomycetales	Microbacteriaceae	unclassified	unclassified
41288	8276	8295	6324	6870	10766	12791	11459	11172	Bacteria	Actinobacteria	Actinobacteria	Actinomycetales	Microbacteriaceae	unclassified	unclassified

Table S6 (part E): HybScores of OTUs that were called present in at least one of the samples. Taxonomic classifications are based on PhyloChip taxonomy (derived from GreenGenes). Please note, that data presented here was rank normalized before performing statisical analyses presented in the manuscript.

	Sample ID (PMA)				Sample ID				taxonomic classification of representative rOTU cluster						
OTU	UR_PMA	CO_PMA	CR8_PMA	CR5_PMA	UR	CO	CR8	CR5	kingdom	phylum	class	order	family	genus	species
41444	8522	9025	6893	7671	11110	11725	11325	11303	Bacteria	Actinobacteria	Actinobacteria	Actinomycetales	Microbacteriaceae	unclassified	unclassified
41480	9200	9070	7343	8057	11209	12687	11590	11955	Bacteria	Actinobacteria	Actinobacteria	Actinomycetales	Microbacteriaceae	unclassified	unclassified
41730	8591	10335	6117	7304	11087	12500	11327	11791	Bacteria	Actinobacteria	Actinobacteria	Actinomycetales	Microbacteriaceae	unclassified	unclassified
41779	9049	9198	6777	7067	10445	12176	10693	11016	Bacteria	Actinobacteria	Actinobacteria	Actinomycetales	Microbacteriaceae	unclassified	unclassified
41868	11588	12025	12185	11007	12493	12853	12551	12981	Bacteria	Actinobacteria	Actinobacteria	Actinomycetales	Microbacteriaceae	unclassified	unclassified
41925	8606	9455	6400	7235	11196	12951	11878	12129	Bacteria	Actinobacteria	Actinobacteria	Actinomycetales	Microbacteriaceae	unclassified	unclassified
41931	7770	8337	6202	6568	10221	13177	10714	10617	Bacteria	Actinobacteria	Actinobacteria	Actinomycetales	Microbacteriaceae	unclassified	unclassified
42358	8942	8759	7556	7977	11300	12512	11757	11849	Bacteria	Actinobacteria	Actinobacteria	Actinomycetales	Microbacteriaceae	unclassified	unclassified
40920	9154	7045	6152	7086	11493	12285	11923	12006	Bacteria	Actinobacteria	Actinobacteria	Actinomycetales	Micrococcaceae	Arthrobacter	Arthrobacter agilis
40152	10253	9786	6218	8973	11922	12855	12150	12749	Bacteria	Actinobacteria	Actinobacteria	Actinomycetales	Micrococcaceae	Arthrobacter	unclassified
40172	10311	9804	6421	8842	12318	12583	12588	12651	Bacteria	Actinobacteria	Actinobacteria	Actinomycetales	Micrococcaceae	Arthrobacter	unclassified
40175	8770	9218	6608	7603	11922	13012	12534	12334	Bacteria	Actinobacteria	Actinobacteria	Actinomycetales	Micrococcaceae	Arthrobacter	unclassified
40191	9698	10700	7030	9185	12525	13438	12768	12843	Bacteria	Actinobacteria	Actinobacteria	Actinomycetales	Micrococcaceae	Arthrobacter	unclassified
40199	9779	10699	6601	7898	12083	12662	12464	12352	Bacteria	Actinobacteria	Actinobacteria	Actinomycetales	Micrococcaceae	Arthrobacter	unclassified
40206	8530	7937	6264	7114	12154	13049	11579	12170	Bacteria	Actinobacteria	Actinobacteria	Actinomycetales	Micrococcaceae	Arthrobacter	unclassified
40209	11555	11628	11219	10530	12993	13397	12860	13331	Bacteria	Actinobacteria	Actinobacteria	Actinomycetales	Micrococcaceae	Arthrobacter	unclassified
40216	10894	11266	6864	8333	13714	13979	13904	14071	Bacteria	Actinobacteria	Actinobacteria	Actinomycetales	Micrococcaceae	Arthrobacter	unclassified
40224	10332	8454	6517	7587	12319	13167	12303	13064	Bacteria	Actinobacteria	Actinobacteria	Actinomycetales	Micrococcaceae	Arthrobacter	unclassified
40234	7801	10224	6663	7469	10946	12052	11182	11540	Bacteria	Actinobacteria	Actinobacteria	Actinomycetales	Micrococcaceae	Arthrobacter	unclassified
40245	10155	9668	7113	8483	13259	13658	13239	13293	Bacteria	Actinobacteria	Actinobacteria	Actinomycetales	Micrococcaceae	Arthrobacter	unclassified
40262	10158	9653	6866	7853	12478	12919	12869	12837	Bacteria	Actinobacteria	Actinobacteria	Actinomycetales	Micrococcaceae	Arthrobacter	unclassified
40303	9762	9809	6965	7371	13128	13136	13204	13255	Bacteria	Actinobacteria	Actinobacteria	Actinomycetales	Micrococcaceae	Arthrobacter	unclassified
40304	9518	8906	6968	7580	12020	12386	12237	13035	Bacteria	Actinobacteria	Actinobacteria	Actinomycetales	Micrococcaceae	Arthrobacter	unclassified
40337	8651	10030	6344	7526	12119	12800	12232	12401	Bacteria	Actinobacteria	Actinobacteria	Actinomycetales	Micrococcaceae	Arthrobacter	unclassified
40340	9108	9666	6868	7167	12335	13154	12436	13119	Bacteria	Actinobacteria	Actinobacteria	Actinomycetales	Micrococcaceae	Arthrobacter	unclassified
40358	9819	11132	7732	8129	12640	13445	12899	12859	Bacteria	Actinobacteria	Actinobacteria	Actinomycetales	Micrococcaceae	Arthrobacter	unclassified
40382	8822	9164	7550	8362	12194	12976	11558	12136	Bacteria	Actinobacteria	Actinobacteria	Actinomycetales	Micrococcaceae	Arthrobacter	unclassified
40394	11991	12618	6093	8908	14052	14234	14248	14376	Bacteria	Actinobacteria	Actinobacteria	Actinomycetales	Micrococcaceae	Arthrobacter	unclassified
40413	9231	7824	6361	7007	11215	12119	12134	11565	Bacteria	Actinobacteria	Actinobacteria	Actinomycetales	Micrococcaceae	Arthrobacter	unclassified
40415	9449	9669	6751	7770	11982	12577	12120	12432	Bacteria	Actinobacteria	Actinobacteria	Actinomycetales	Micrococcaceae	Arthrobacter	unclassified
40416	8465	9048	7276	7465	11532	11994	11786	12230	Bacteria	Actinobacteria	Actinobacteria	Actinomycetales	Micrococcaceae	Arthrobacter	unclassified
40425	8733	8754	6698	7430	12380	12900	12259	12119	Bacteria	Actinobacteria	Actinobacteria	Actinomycetales	Micrococcaceae	Arthrobacter	unclassified
40427	9300	8367	7679	8022	11882	12109	11713	12620	Bacteria	Actinobacteria	Actinobacteria	Actinomycetales	Micrococcaceae	Arthrobacter	unclassified
40455	8852	9393	6198	7848	11923	13034	11922	12708	Bacteria	Actinobacteria	Actinobacteria	Actinomycetales	Micrococcaceae	Arthrobacter	unclassified
40474	9982	9965	7069	7237	13436	13264	13382	13426	Bacteria	Actinobacteria	Actinobacteria	Actinomycetales	Micrococcaceae	Arthrobacter	unclassified
40486	9874	9278	6137	8768	11219	12465	11654	11466	Bacteria	Actinobacteria	Actinobacteria	Actinomycetales	Micrococcaceae	Arthrobacter	unclassified
40488	9985	10443	8602	9456	12620	13223	12326	12444	Bacteria	Actinobacteria	Actinobacteria	Actinomycetales	Micrococcaceae	Arthrobacter	unclassified
40495	8812	9929	6045	7012	12278	13011	12214	12244	Bacteria	Actinobacteria	Actinobacteria	Actinomycetales	Micrococcaceae	Arthrobacter	unclassified
40503	9264	8989	7248	7615	11475	12523	11724	12034	Bacteria	Actinobacteria	Actinobacteria	Actinomycetales	Micrococcaceae	Arthrobacter	unclassified
40512	9969	9619	7415	8496	12956	13181	12992	13124	Bacteria	Actinobacteria	Actinobacteria	Actinomycetales	Micrococcaceae	Arthrobacter	unclassified
40522	9749	8819	6463	8709	12044	12893	11989	12516	Bacteria	Actinobacteria	Actinobacteria	Actinomycetales	Micrococcaceae	Arthrobacter	unclassified
40526	9463	9615	8133	8521	12059	12538	12074	12725	Bacteria	Actinobacteria	Actinobacteria	Actinomycetales	Micrococcaceae	Arthrobacter	unclassified
40535	8504	8212	6517	7651	11083	12662	11271	12383	Bacteria	Actinobacteria	Actinobacteria	Actinomycetales	Micrococcaceae	Arthrobacter	unclassified
40558	8530	7693	5698	7008	12318	12871	11826	11822	Bacteria	Actinobacteria	Actinobacteria	Actinomycetales	Micrococcaceae	Arthrobacter	unclassified
40568	9176	8515	6575	7383	12485	12966	12341	12192	Bacteria	Actinobacteria	Actinobacteria	Actinomycetales	Micrococcaceae	Arthrobacter	unclassified
40577	10230	11403	7815	8595	12467	13149	12514	12978	Bacteria	Actinobacteria	Actinobacteria	Actinomycetales	Micrococcaceae	Arthrobacter	unclassified
40583	8651	10030	6355	7281	12119	12800	12232	12401	Bacteria	Actinobacteria	Actinobacteria	Actinomycetales	Micrococcaceae	Arthrobacter	unclassified
40586	10604	10766	7567	9709	12866	13512	13209	13249	Bacteria	Actinobacteria	Actinobacteria	Actinomycetales	Micrococcaceae	Arthrobacter	unclassified
40587	9702	9564	8151	8730	11758	12477	12379	12452	Bacteria	Actinobacteria	Actinobacteria	Actinomycetales	Micrococcaceae	Arthrobacter	unclassified
40610	9498	9674	6474	7681	12786	13336	12889	13139	Bacteria	Actinobacteria	Actinobacteria	Actinomycetales	Micrococcaceae	Arthrobacter	unclassified
40620	10045	9661	6885	7775	12292	12776	12702	12684	Bacteria	Actinobacteria	Actinobacteria	Actinomycetales	Micrococcaceae	Arthrobacter	unclassified
40639	9175	9750	7963	8147	12493	12950	12466	12828	Bacteria	Actinobacteria	Actinobacteria	Actinomycetales	Micrococcaceae	Arthrobacter	unclassified
40666	10960	10608	10665	10637	12367	12936	12407	12574	Bacteria	Actinobacteria	Actinobacteria	Actinomycetales	Micrococcaceae	Arthrobacter	unclassified
40676	8841	9591	7176	7675	12198	12936	12688	13104	Bacteria	Actinobacteria	Actinobacteria	Actinomycetales	Micrococcaceae	Arthrobacter	unclassified
40678	9295	9781	7720	9136	12338	13037	11981	12536	Bacteria	Actinobacteria	Actinobacteria	Actinomycetales	Micrococcaceae	Arthrobacter	unclassified
40723	10742	12402	9889	10283	13314	13811	13209	13523	Bacteria	Actinobacteria	Actinobacteria	Actinomycetales	Micrococcaceae	Arthrobacter	unclassified
40741	9105	8794	6569	7267	12699	13149	12561	12420	Bacteria	Actinobacteria	Actinobacteria	Actinomycetales	Micrococcaceae	Arthrobacter	unclassified
40778	9978	10064	6642	7206	13000	13421	12967	13085	Bacteria	Actinobacteria	Actinobacteria	Actinomycetales	Micrococcaceae	Arthrobacter	unclassified
40797	8436	9551	6463	7325	11561	12552	11801	11732	Bacteria	Actinobacteria	Actinobacteria	Actinomycetales	Micrococcaceae	Arthrobacter	unclassified
40805	9897	9403	6628	7664	12189	12721	12442	12586	Bacteria	Actinobacteria	Actinobacteria	Actinomycetales	Micrococcaceae	Arthrobacter	unclassified
40827	9226	7621	5966	6935	11299	12095	11832	11876	Bacteria	Actinobacteria	Actinobacteria	Actinomycetales	Micrococcaceae	Arthrobacter	unclassified
40867	9061	9828	6368	7472	12472	13085	12527	12832	Bacteria	Actinobacteria	Actinobacteria	Actinomycetales	Micrococcaceae	Arthrobacter	unclassified
40875	9113	9421	7453	8767	12479	13121	11935	12361	Bacteria	Actinobacteria	Actinobacteria	Actinomycetales	Micrococcaceae	Arthrobacter	unclassified
40876	8136	10966	6355	8303	12844	13269	12794	13194	Bacteria	Actinobacteria	Actinobacteria	Actinomycetales	Micrococcaceae	Arthrobacter	unclassified
40898	11361	12148	11295	8835	13027	13401	13189	13254	Bacteria	Actinobacteria	Actinobacteria	Actinomycetales	Micrococcaceae	Arthrobacter	unclassified
40919	10307	10017	9773	9690	12176	12679	12271	12571	Bacteria	Actinobacteria	Actinobacteria	Actinomycetales	Micrococcaceae	Arthrobacter	unclassified
40930	9382	9088	6495	7165	11866	12502	11834	12036	Bacteria	Actinobacteria	Actinobacteria	Actinomycetales	Micrococcaceae	Arthrobacter	unclassified
40945	9603	10264	7190	8216	11910	12750	12183	12822	Bacteria	Actinobacteria	Actinobacteria	Actinomycetales	Micrococcaceae	Arthrobacter	unclassified
40962	9688	8797	6319	7184	12775	13163	12578	12558	Bacteria	Actinobacteria	Actinobacteria	Actinomycetales	Micrococcaceae	Arthrobacter	unclassified
40968	9354	9470	6712	7402	12614	13106	12604	12546	Bacteria	Actinobacteria	Actinobacteria	Actinomycetales	Micrococcaceae	Arthrobacter	unclassified
40979	8965	8976	7112	7525	11560	12305	11922	12814	Bacteria	Actinobacteria	Actinobacteria	Actinomycetales	Micrococcaceae	Arthrobacter	unclassified
41008	9722	9709	7665	8632	12509	13095	12271	12225	Bacteria	Actinobacteria	Actinobacteria	Actinomycetales	Micrococcaceae	Arthrobacter	unclassified
41013	9292	8171	6540	7365	11476	12517	11787	11966	Bacteria	Actinobacteria	Actinobacteria	Actinomycetales	Micrococcaceae	Arthrobacter	unclassified
41022	8078	7971	6513	7651	10456	12304	10800	11748	Bacteria	Actinobacteria	Actinobacteria	Actinomycetales	Micrococcaceae	Arthrobacter	unclassified
41035	9359	9770	7708	9257	11926	13380	11530	12591	Bacteria	Actinobacteria	Actinobacteria	Actinomycetales	Micrococcaceae	Arthrobacter	unclassified
41048	9231	8499	6276	7394	12669	13162	12431	12227	Bacteria	Actinobacteria	Actinobacteria	Actinomycetales	Micrococcaceae	Arthrobacter	unclassified
41049	9093	9736	7749	8204	12333	12817	12542	12942	Bacteria	Actinobacteria	Actinobacteria	Actinomycetales	Micrococcaceae	Arthrobacter	unclassified
41050	9520	9200	6501	7275	11963	12547	12390	12340	Bacteria	Actinobacteria	Actinobacteria	Actinomycetales	Micrococcaceae	Arthrobacter	unclassified
41058	9796	9129	6533	7286	12790	13221	12665	12629	Bacteria	Actinobacteria	Actinobacteria	Actinomycetales	Micrococcaceae	Arthrobacter	unclassified
41079	9729	9224	6862	7732	11985	12569	12473	12380	Bacteria	Actinobacteria	Actinobacteria	Actinomycetales	Micrococcaceae	Arthrobacter	unclassified
41080	11056	11613	10222	9810	12957	13477	13117	13145	Bacteria	Actinobacteria	Actinobacteria	Actinomycetales	Micrococcaceae	Arthrobacter	unclassified
41923	10857	10856	6265	8084	13331	13474	13380	13423	Bacteria	Actinobacteria	Actinobacteria	Actinomycetales	Micrococcaceae	Arthrobacter	unclassified
40145	9458	10432	7995	7341	12383	12837	12761	13128	Bacteria	Actinobacteria	Actinobacteria	Actinomycetales	Micrococcaceae	Kocuria	Kocuria marina
40235	10627	11112	10848	8802	13165	13375	13267	13743	Bacteria	Actinobacteria	Actinobacteria	Actinomycetales	Micrococcaceae	Kocuria	Kocuria marina
40727	9430	9523	7249	6937	12301	13070	13078	13146	Bacteria	Actinobacteria	Actinobacteria	Actinomycetales	Micrococcaceae	Kocuria	Kocuria marina
40846	9726	9724	7821	7807	12651	12870	12669	13231	Bacteria	Actinobacteria	Actinobacteria	Actinomycetales	Micrococcaceae	Kocuria	Kocuria marina
40141	9911	9187	6884	6859	12787	12935	12870	13241	Bacteria	Actinobacteria	Actinobacteria	Actinomycetales	Micrococcaceae	Kocuria	unclassified
39946	9122	10765	8933	8649	11795	13078	11938	11879	Bacteria	Actinobacteria	Actinobacteria	Actinomycetales	Micrococcaceae	Micrococcus	Micrococcus luteus
40178	9668	8837	6222	7033	13115	13205	13677	13271	Bacteria	Actinobacteria	Actinobacteria	Actinomycetales	Micrococcaceae	Micrococcus	Micrococcus luteus
40202	9457	9585	7604	8788	12934	13331	13352	13052	Bacteria	Actinobacteria	Actinobacteria	Actinomycetales	Micrococcaceae	Micrococcus	Micrococcus luteus
40219	9823	9547	6934	7388	13186	13412	13701	13453	Bacteria	Actinobacteria	Actinobacteria	Actinomycetales	Micrococcaceae	Micrococcus	Micrococcus luteus
40222	8695	7328	6217	6872	12527	12642	13466	12695	Bacteria	Actinobacteria	Actinobacteria	Actinomycetales	Micrococcaceae	Micrococcus	Micrococcus luteus
40238	8532	6727	6056	6427	12471	12554	13459	12570	Bacteria	Actinobacteria	Actinobacteria	Actinomycetales	Micrococcaceae	Micrococcus	Micrococcus luteus
40289	9569	9575	7753	8802	12805	13205	13348	12970	Bacteria	Actinobacteria	Actinobacteria	Actinomycetales	Micrococcaceae	Micrococcus	Micrococcus luteus
40314	9608	10115	7965	8979	12962	13401	13415	13095	Bacteria	Actinobacteria	Actinobacteria	Actinomycetales	Micrococcaceae	Micrococcus	Micrococcus luteus
40322	8908	9577	7918	9137	11898	12519	12493	12077	Bacteria	Actinobacteria	Actinobacteria	Actinomycetales	Micrococcaceae	Micrococcus	Micrococcus luteus
40325	9183	9457	8042	9026	12256	12579	13229	12408	Bacteria	Actinobacteria	Actinobacteria	Actinomycetales	Micrococcaceae	Micrococcus	Micrococcus luteus
40347	9569	9312	6547	7141	13195	13413	13646	13311	Bacteria	Actinobacteria	Actinobacteria	Actinomycetales	Micrococcaceae	Micrococcus	Micrococcus luteus
40373	9553	9574	7613	8811	12899	13286	13446	13045	Bacteria	Actinobacteria	Actinobacteria	Actinomycetales	Micrococcaceae	Micrococcus	Micrococcus luteus
40399	9547	9242	7503	8628	12556	12816	13390	12685	Bacteria	Actinobacteria	Actinobacteria	Actinomycetales	Micrococcaceae	Micrococcus	Micrococcus luteus

Table S6 (part F): HybScores of OTUs that were called present in at least one of the samples. Taxonomic classifications are based on PhyloChip taxonomy (derived from GreenGenes). Please note, that data presented here was rank normalized before performing statisical analyses presented in the manuscript.

	Sample ID (PMA)				Sample ID				taxonomic classification of representative rOTU cluster						
OTU	UR_PMA	CO_PMA	CR8_PMA	CR5_PMA	UR	CO	CR8	CR5	kingdom	phylum	class	order	family	genus	species
40436	9781	9337	6308	7149	13243	13343	13747	13442	Bacteria	Actinobacteria	Actinobacteria	Actinomycetales	Micrococcaceae	Micrococcus	Micrococcus luteus
40476	9578	9811	8909	9227	12173	12786	12313	12622	Bacteria	Actinobacteria	Actinobacteria	Actinomycetales	Micrococcaceae	Micrococcus	Micrococcus luteus
40479	9755	9483	6490	7122	13164	13337	13822	13404	Bacteria	Actinobacteria	Actinobacteria	Actinomycetales	Micrococcaceae	Micrococcus	Micrococcus luteus
40484	9626	9895	7951	9070	12573	13006	13391	12738	Bacteria	Actinobacteria	Actinobacteria	Actinomycetales	Micrococcaceae	Micrococcus	Micrococcus luteus
40490	9429	10696	8019	9187	11625	12449	12489	12103	Bacteria	Actinobacteria	Actinobacteria	Actinomycetales	Micrococcaceae	Micrococcus	Micrococcus luteus
40510	9488	9637	7590	8681	12857	13234	13262	12884	Bacteria	Actinobacteria	Actinobacteria	Actinomycetales	Micrococcaceae	Micrococcus	Micrococcus luteus
40574	9213	8236	6469	7021	12647	12804	13312	12919	Bacteria	Actinobacteria	Actinobacteria	Actinomycetales	Micrococcaceae	Micrococcus	Micrococcus luteus
40575	9474	9322	6300	6927	13045	13213	13782	13270	Bacteria	Actinobacteria	Actinobacteria	Actinomycetales	Micrococcaceae	Micrococcus	Micrococcus luteus
40621	9530	8332	6121	6702	12777	12827	13596	13019	Bacteria	Actinobacteria	Actinobacteria	Actinomycetales	Micrococcaceae	Micrococcus	Micrococcus luteus
40640	8781	7382	6181	6710	12691	12805	13578	12827	Bacteria	Actinobacteria	Actinobacteria	Actinomycetales	Micrococcaceae	Micrococcus	Micrococcus luteus
40696	8766	8050	6273	6756	12558	12743	13520	12616	Bacteria	Actinobacteria	Actinobacteria	Actinomycetales	Micrococcaceae	Micrococcus	Micrococcus luteus
40697	8989	8841	6429	8169	12583	12803	13508	12676	Bacteria	Actinobacteria	Actinobacteria	Actinomycetales	Micrococcaceae	Micrococcus	Micrococcus luteus
40718	9746	9650	6415	7286	13336	13498	13778	13471	Bacteria	Actinobacteria	Actinobacteria	Actinomycetales	Micrococcaceae	Micrococcus	Micrococcus luteus
40740	9738	10099	8086	8977	12924	13356	13269	13063	Bacteria	Actinobacteria	Actinobacteria	Actinomycetales	Micrococcaceae	Micrococcus	Micrococcus luteus
40753	8926	10574	7083	7360	12129	12860	12678	12458	Bacteria	Actinobacteria	Actinobacteria	Actinomycetales	Micrococcaceae	Micrococcus	Micrococcus luteus
40757	9826	9432	6410	7183	12913	13000	13678	13147	Bacteria	Actinobacteria	Actinobacteria	Actinomycetales	Micrococcaceae	Micrococcus	Micrococcus luteus
40834	9139	9393	8072	9070	12200	12603	13167	12325	Bacteria	Actinobacteria	Actinobacteria	Actinomycetales	Micrococcaceae	Micrococcus	Micrococcus luteus
40861	8939	8798	6526	8247	12562	12831	13457	12621	Bacteria	Actinobacteria	Actinobacteria	Actinomycetales	Micrococcaceae	Micrococcus	Micrococcus luteus
40884	8839	7960	6096	6666	12720	12838	13634	12905	Bacteria	Actinobacteria	Actinobacteria	Actinomycetales	Micrococcaceae	Micrococcus	Micrococcus luteus
40911	9152	9700	8079	8979	12695	13182	13171	12746	Bacteria	Actinobacteria	Actinobacteria	Actinomycetales	Micrococcaceae	Micrococcus	Micrococcus luteus
40960	9534	9206	7401	8762	12459	12685	13391	12642	Bacteria	Actinobacteria	Actinobacteria	Actinomycetales	Micrococcaceae	Micrococcus	Micrococcus luteus
40992	9218	8994	6555	7008	12782	13047	13576	12926	Bacteria	Actinobacteria	Actinobacteria	Actinomycetales	Micrococcaceae	Micrococcus	Micrococcus luteus
41054	9766	9632	6468	7153	13266	13427	13829	13486	Bacteria	Actinobacteria	Actinobacteria	Actinomycetales	Micrococcaceae	Micrococcus	Micrococcus luteus
41061	9572	9359	6623	7194	13316	13512	13666	13432	Bacteria	Actinobacteria	Actinobacteria	Actinomycetales	Micrococcaceae	Micrococcus	Micrococcus luteus
40263	8160	9030	6422	7080	11933	12427	12674	12014	Bacteria	Actinobacteria	Actinobacteria	Actinomycetales	Micrococcaceae	Micrococcus	unclassified
40376	8948	8642	6507	8221	12403	12750	13013	12696	Bacteria	Actinobacteria	Actinobacteria	Actinomycetales	Micrococcaceae	Micrococcus	unclassified
40473	9712	11039	9181	8113	13405	13617	13594	13453	Bacteria	Actinobacteria	Actinobacteria	Actinomycetales	Micrococcaceae	Micrococcus	unclassified
40679	9476	9823	7663	8725	12962	13376	13398	12975	Bacteria	Actinobacteria	Actinobacteria	Actinomycetales	Micrococcaceae	Micrococcus	unclassified
40819	9125	9696	8121	9185	12356	12770	13297	12454	Bacteria	Actinobacteria	Actinobacteria	Actinomycetales	Micrococcaceae	Micrococcus	unclassified
40889	8764	8080	6214	6661	12775	12951	13682	12903	Bacteria	Actinobacteria	Actinobacteria	Actinomycetales	Micrococcaceae	Micrococcus	unclassified
40943	9709	10532	6290	8223	13117	13546	13404	13091	Bacteria	Actinobacteria	Actinobacteria	Actinomycetales	Micrococcaceae	Micrococcus	unclassified
41105	8388	8113	6585	7402	11519	12675	11846	12145	Bacteria	Actinobacteria	Actinobacteria	Actinomycetales	Micromonosporaceae	Actinoplanes	unclassified
41110	9007	9763	6988	7846	11099	12431	11589	11882	Bacteria	Actinobacteria	Actinobacteria	Actinomycetales	Micromonosporaceae	Actinoplanes	unclassified
41199	9359	8414	6572	7481	11263	12681	11582	12446	Bacteria	Actinobacteria	Actinobacteria	Actinomycetales	Micromonosporaceae	Actinoplanes	unclassified
41237	8800	8365	7345	8174	10581	11799	11008	11907	Bacteria	Actinobacteria	Actinobacteria	Actinomycetales	Micromonosporaceae	Actinoplanes	unclassified
41371	8719	9867	6772	8061	11066	12494	11656	12099	Bacteria	Actinobacteria	Actinobacteria	Actinomycetales	Micromonosporaceae	Actinoplanes	unclassified
41489	9331	6857	6762	6806	11303	12248	11918	11768	Bacteria	Actinobacteria	Actinobacteria	Actinomycetales	Micromonosporaceae	Actinoplanes	unclassified
41636	8337	7819	6601	7674	11127	12480	11677	11839	Bacteria	Actinobacteria	Actinobacteria	Actinomycetales	Micromonosporaceae	Actinoplanes	unclassified
41502	9263	9387	6943	7647	12528	13195	12580	12737	Bacteria	Actinobacteria	Actinobacteria	Actinomycetales	Micromonosporaceae	Dactylosporangium	unclassified
42075	9533	9410	7656	8740	11728	12617	11782	12780	Bacteria	Actinobacteria	Actinobacteria	Actinomycetales	Micromonosporaceae	Dactylosporangium	unclassified
42425	8899	9454	7447	7847	10677	12052	10936	11491	Bacteria	Actinobacteria	Actinobacteria	Actinomycetales	Micromonosporaceae	Dactylosporangium	unclassified
41204	10162	9729	8895	8868	12886	13822	12768	13300	Bacteria	Actinobacteria	Actinobacteria	Actinomycetales	Micromonosporaceae	unclassified	unclassified
41253	10250	10380	7394	8830	11418	12749	11699	12238	Bacteria	Actinobacteria	Actinobacteria	Actinomycetales	Micromonosporaceae	unclassified	unclassified
41272	9675	9207	8022	8650	11520	12983	11677	12436	Bacteria	Actinobacteria	Actinobacteria	Actinomycetales	Micromonosporaceae	unclassified	unclassified
41368	9833	8642	6543	7344	11868	13035	12130	12732	Bacteria	Actinobacteria	Actinobacteria	Actinomycetales	Micromonosporaceae	unclassified	unclassified
41770	9860	10264	7436	7478	12514	13138	12539	12958	Bacteria	Actinobacteria	Actinobacteria	Actinomycetales	Micromonosporaceae	unclassified	unclassified
41900	9789	10047	6854	8169	11509	12876	12000	12147	Bacteria	Actinobacteria	Actinobacteria	Actinomycetales	Micromonosporaceae	unclassified	unclassified
42004	10152	10929	8876	9623	12828	13717	12684	13007	Bacteria	Actinobacteria	Actinobacteria	Actinomycetales	Micromonosporaceae	unclassified	unclassified
42357	9079	9175	6066	7708	12112	13057	12394	12667	Bacteria	Actinobacteria	Actinobacteria	Actinomycetales	Micromonosporaceae	unclassified	unclassified
37403	9502	9361	7599	8423	11339	12184	12059	12092	Bacteria	Actinobacteria	Actinobacteria	Actinomycetales	Mycobacteriaceae	Mycobacterium	unclassified
37630	9429	9781	8555	8664	11270	12109	11603	11721	Bacteria	Actinobacteria	Actinobacteria	Actinomycetales	Mycobacteriaceae	Mycobacterium	unclassified
37688	9058	8232	7250	7560	12364	12943	12548	13196	Bacteria	Actinobacteria	Actinobacteria	Actinomycetales	Mycobacteriaceae	Mycobacterium	unclassified
37726	10537	11407	9363	8985	13759	14209	13741	14145	Bacteria	Actinobacteria	Actinobacteria	Actinomycetales	Mycobacteriaceae	Mycobacterium	unclassified
37737	9681	11300	8224	9086	12057	13233	12473	12680	Bacteria	Actinobacteria	Actinobacteria	Actinomycetales	Mycobacteriaceae	Mycobacterium	unclassified
37907	8318	7851	7145	7567	11218	12241	12035	12044	Bacteria	Actinobacteria	Actinobacteria	Actinomycetales	Mycobacteriaceae	Mycobacterium	unclassified
37943	9692	10781	9144	9224	11828	12881	12518	12373	Bacteria	Actinobacteria	Actinobacteria	Actinomycetales	Mycobacteriaceae	Mycobacterium	unclassified
37955	8851	7886	7245	7877	10345	11882	11592	11367	Bacteria	Actinobacteria	Actinobacteria	Actinomycetales	Mycobacteriaceae	Mycobacterium	unclassified
37984	8856	8790	6544	7929	11045	12725	11849	11860	Bacteria	Actinobacteria	Actinobacteria	Actinomycetales	Mycobacteriaceae	Mycobacterium	unclassified
37996	8886	8211	6804	7409	12376	12980	12604	12979	Bacteria	Actinobacteria	Actinobacteria	Actinomycetales	Mycobacteriaceae	Mycobacterium	unclassified
38001	10679	11219	8835	10552	12698	12709	13109	13150	Bacteria	Actinobacteria	Actinobacteria	Actinomycetales	Mycobacteriaceae	Mycobacterium	unclassified
38115	8629	7906	6556	7144	12344	12451	12462	13266	Bacteria	Actinobacteria	Actinobacteria	Actinomycetales	Mycobacteriaceae	Mycobacterium	unclassified
38168	9206	9733	6790	8050	12346	12878	12587	13445	Bacteria	Actinobacteria	Actinobacteria	Actinomycetales	Mycobacteriaceae	Mycobacterium	unclassified
38238	8625	8194	7830	7788	10549	11728	11723	11612	Bacteria	Actinobacteria	Actinobacteria	Actinomycetales	Mycobacteriaceae	Mycobacterium	unclassified
38248	9164	10642	8137	8811	11271	12888	11472	11539	Bacteria	Actinobacteria	Actinobacteria	Actinomycetales	Mycobacteriaceae	Mycobacterium	unclassified
38371	9377	9910	7953	7601	11776	12267	12120	12203	Bacteria	Actinobacteria	Actinobacteria	Actinomycetales	Mycobacteriaceae	Mycobacterium	unclassified
38427	9034	9151	7177	8085	11502	12989	11951	12063	Bacteria	Actinobacteria	Actinobacteria	Actinomycetales	Mycobacteriaceae	Mycobacterium	unclassified
38438	8638	8502	7120	7315	11473	12416	12208	12016	Bacteria	Actinobacteria	Actinobacteria	Actinomycetales	Mycobacteriaceae	Mycobacterium	unclassified
38444	8451	8034	8048	7975	10222	11712	11565	11325	Bacteria	Actinobacteria	Actinobacteria	Actinomycetales	Mycobacteriaceae	Mycobacterium	unclassified
37536	10400	7185	6434	6822	11359	12604	11838	12357	Bacteria	Actinobacteria	Actinobacteria	Actinomycetales	Nakamurellaceae	unclassified	unclassified
37835	10144	10919	6835	7670	11960	13181	12237	12522	Bacteria	Actinobacteria	Actinobacteria	Actinomycetales	Nakamurellaceae	unclassified	unclassified
37963	8913	10431	6777	6895	10392	12125	10824	11353	Bacteria	Actinobacteria	Actinobacteria	Actinomycetales	Nakamurellaceae	unclassified	unclassified
38331	8990	9135	7223	7230	12700	12968	12290	13622	Bacteria	Actinobacteria	Actinobacteria	Actinomycetales	Nocardiaceae	Nocardia	unclassified
37390	8974	9419	7455	7200	10895	11803	11030	11127	Bacteria	Actinobacteria	Actinobacteria	Actinomycetales	Nocardiaceae	Rhodococcus	unclassified
37398	9325	9433	7354	7345	11721	12794	12227	12567	Bacteria	Actinobacteria	Actinobacteria	Actinomycetales	Nocardiaceae	Rhodococcus	unclassified
37506	9431	9527	7381	7236	12064	13089	12638	12749	Bacteria	Actinobacteria	Actinobacteria	Actinomycetales	Nocardiaceae	Rhodococcus	unclassified
37562	8333	8127	6896	7579	10577	11810	12139	11258	Bacteria	Actinobacteria	Actinobacteria	Actinomycetales	Nocardiaceae	Rhodococcus	unclassified
37671	9340	9267	7223	7252	11685	12795	12268	12580	Bacteria	Actinobacteria	Actinobacteria	Actinomycetales	Nocardiaceae	Rhodococcus	unclassified
37679	8763	8521	6912	7402	11432	12538	11827	12116	Bacteria	Actinobacteria	Actinobacteria	Actinomycetales	Nocardiaceae	Rhodococcus	unclassified
37691	8350	8116	6641	7337	10295	11639	12018	11211	Bacteria	Actinobacteria	Actinobacteria	Actinomycetales	Nocardiaceae	Rhodococcus	unclassified
37838	9359	9542	7411	7350	12062	13036	12602	12801	Bacteria	Actinobacteria	Actinobacteria	Actinomycetales	Nocardiaceae	Rhodococcus	unclassified
37868	8404	8314	6645	7425	10999	12011	12409	11600	Bacteria	Actinobacteria	Actinobacteria	Actinomycetales	Nocardiaceae	Rhodococcus	unclassified
37921	8892	8533	6769	7258	11813	12868	12362	12530	Bacteria	Actinobacteria	Actinobacteria	Actinomycetales	Nocardiaceae	Rhodococcus	unclassified
37948	9108	9588	7357	7239	12100	13049	12697	12689	Bacteria	Actinobacteria	Actinobacteria	Actinomycetales	Nocardiaceae	Rhodococcus	unclassified
38099	8806	8374	6655	7292	11498	12716	11963	12238	Bacteria	Actinobacteria	Actinobacteria	Actinomycetales	Nocardiaceae	Rhodococcus	unclassified
38272	8257	8035	6590	7382	10365	11596	12169	11055	Bacteria	Actinobacteria	Actinobacteria	Actinomycetales	Nocardiaceae	Rhodococcus	unclassified
38394	10919	11444	10999	10074	11925	12689	11985	12461	Bacteria	Actinobacteria	Actinobacteria	Actinomycetales	Nocardiaceae	Rhodococcus	unclassified
38449	8649	8768	7814	7252	11771	12505	12037	12355	Bacteria	Actinobacteria	Actinobacteria	Actinomycetales	Nocardiaceae	Rhodococcus	unclassified
39044	9261	10008	6978	7198	12499	12924	12167	12693	Bacteria	Actinobacteria	Actinobacteria	Actinomycetales	Nocardioidaceae	Actinopolymorpha	unclassified
39676	8654	8281	6289	7370	11957	12669	12183	12253	Bacteria	Actinobacteria	Actinobacteria	Actinomycetales	Nocardioidaceae	Aeromicrobium	unclassified
39684	9909	9428	6788	7821	12217	13192	12551	12461	Bacteria	Actinobacteria	Actinobacteria	Actinomycetales	Nocardioidaceae	Aeromicrobium	unclassified
39809	8526	9304	6539	7115	12091	13023	12259	12544	Bacteria	Actinobacteria	Actinobacteria	Actinomycetales	Nocardioidaceae	Aeromicrobium	unclassified
39869	9067	8887	8378	8636	11625	12884	11855	11709	Bacteria	Actinobacteria	Actinobacteria	Actinomycetales	Nocardioidaceae	Aeromicrobium	unclassified
38907	9904	9204	6632	7799	12733	13685	13050	13296	Bacteria	Actinobacteria	Actinobacteria	Actinomycetales	Nocardioidaceae	Friedmanniella	unclassified
39719	9679	9940	7853	8269	12161	13211	12484	12786	Bacteria	Actinobacteria	Actinobacteria	Actinomycetales	Nocardioidaceae	Friedmanniella	unclassified
39774	10077	9919	7721	8639	12985	13800	13088	13430	Bacteria	Actinobacteria	Actinobacteria	Actinomycetales	Nocardioidaceae	Friedmanniella	unclassified
39989	9855	9472	6287	7790	11850	12829	12181	12414	Bacteria	Actinobacteria	Actinobacteria	Actinomycetales	Nocardioidaceae	Friedmanniella	unclassified
39092	9611	9704	6339	6516	11636	12732	11844	12347	Bacteria	Actinobacteria	Actinobacteria	Actinomycetales	Nocardioidaceae	Marmoricola	unclassified
39654	10224	10298	6048	7338	12507	13181	12609	12855	Bacteria	Actinobacteria	Actinobacteria	Actinomycetales	Nocardioidaceae	Marmoricola	unclassified
39473	9966	11105	6651	7542	12361	13302	12641	12919	Bacteria	Actinobacteria	Actinobacteria	Actinomycetales	Nocardioidaceae	Nocardioides	Nocardioides lentus
38813	10077	10059	7245	8049	13115	13627	13161	13284	Bacteria	Actinobacteria	Actinobacteria	Actinomycetales	Nocardioidaceae	Nocardioides	Nocardioides oleivorans

Table S6 (part G): HybScores of OTUs that were called present in at least one of the samples. Taxonomic classifications are based on PhyloChip taxonomy (derived from GreenGenes). Please note, that data presented here was rank normalized before performing statisical analyses presented in the manuscript.

	Sample ID (PMA)				Sample ID				taxonomic classification of representative rOTU cluster						
OTU	UR_PMA	CO_PMA	CR8_PMA	CR5_PMA	UR	CO	CR8	CR5	kingdom	phylum	class	order	family	genus	species
38628	9913	11619	6750	8026	12968	13477	13015	13179	Bacteria	Actinobacteria	Actinobacteria	Actinomycetales	Nocardioidaceae	Nocardioides	unclassified
38646	10161	9318	9762	9513	12145	13082	12174	12389	Bacteria	Actinobacteria	Actinobacteria	Actinomycetales	Nocardioidaceae	Nocardioides	unclassified
38679	9725	9059	7123	7192	13172	13485	13148	13169	Bacteria	Actinobacteria	Actinobacteria	Actinomycetales	Nocardioidaceae	Nocardioides	unclassified
38746	9444	9180	5978	7293	12364	13184	12455	12279	Bacteria	Actinobacteria	Actinobacteria	Actinomycetales	Nocardioidaceae	Nocardioides	unclassified
38920	9414	8673	6593	7931	12255	12946	12392	12216	Bacteria	Actinobacteria	Actinobacteria	Actinomycetales	Nocardioidaceae	Nocardioides	unclassified
38958	9520	9342	6748	8126	12435	13215	12835	13267	Bacteria	Actinobacteria	Actinobacteria	Actinomycetales	Nocardioidaceae	Nocardioides	unclassified
38996	10174	9909	7434	7712	12854	13656	13148	13100	Bacteria	Actinobacteria	Actinobacteria	Actinomycetales	Nocardioidaceae	Nocardioides	unclassified
39027	10392	9655	7203	7155	13233	13770	13280	13571	Bacteria	Actinobacteria	Actinobacteria	Actinomycetales	Nocardioidaceae	Nocardioides	unclassified
39081	9745	10141	8025	7879	13175	13791	13196	13511	Bacteria	Actinobacteria	Actinobacteria	Actinomycetales	Nocardioidaceae	Nocardioides	unclassified
39446	10754	10822	7906	9571	12607	13143	12880	12907	Bacteria	Actinobacteria	Actinobacteria	Actinomycetales	Nocardioidaceae	Nocardioides	unclassified
39559	10071	9199	6938	7399	12312	13245	12556	13023	Bacteria	Actinobacteria	Actinobacteria	Actinomycetales	Nocardioidaceae	Nocardioides	unclassified
39646	8449	7893	6023	7074	9936	11672	11005	11103	Bacteria	Actinobacteria	Actinobacteria	Actinomycetales	Nocardioidaceae	Nocardioides	unclassified
40042	10167	9402	6591	7530	13076	13654	13118	13083	Bacteria	Actinobacteria	Actinobacteria	Actinomycetales	Nocardioidaceae	Nocardioides	unclassified
39968	8480	11309	6915	7438	10426	12773	10686	10490	Bacteria	Actinobacteria	Actinobacteria	Actinomycetales	Nocardioidaceae	Propionicimonas	unclassified
38499	9681	10739	6721	7990	12765	13213	13031	13084	Bacteria	Actinobacteria	Actinobacteria	Actinomycetales	Nocardioidaceae	unclassified	unclassified
38519	10184	10773	9349	9822	12476	12947	12676	12605	Bacteria	Actinobacteria	Actinobacteria	Actinomycetales	Nocardioidaceae	unclassified	unclassified
39106	9631	9105	6793	8152	12415	13180	12436	12496	Bacteria	Actinobacteria	Actinobacteria	Actinomycetales	Nocardioidaceae	unclassified	unclassified
39343	9927	9994	6907	8012	12574	13355	12932	13344	Bacteria	Actinobacteria	Actinobacteria	Actinomycetales	Nocardioidaceae	unclassified	unclassified
39833	9570	10118	7628	8367	12496	13542	12873	13070	Bacteria	Actinobacteria	Actinobacteria	Actinomycetales	Nocardioidaceae	unclassified	unclassified
39936	9068	8410	6559	7687	11742	12185	11834	12071	Bacteria	Actinobacteria	Actinobacteria	Actinomycetales	Nocardioidaceae	unclassified	unclassified
40101	9413	10004	7171	7346	12081	12980	12285	12776	Bacteria	Actinobacteria	Actinobacteria	Actinomycetales	Nocardioidaceae	unclassified	unclassified
41103	8573	9039	6406	6784	11618	12139	11989	11809	Bacteria	Actinobacteria	Actinobacteria	Actinomycetales	Nocardiopsaceae	Nocardiopsis	unclassified
41490	10049	10808	8606	9279	12714	13425	13091	12895	Bacteria	Actinobacteria	Actinobacteria	Actinomycetales	Nocardiopsaceae	Nocardiopsis	unclassified
41531	9087	9552	6686	7285	12012	12790	13008	12190	Bacteria	Actinobacteria	Actinobacteria	Actinomycetales	Nocardiopsaceae	Nocardiopsis	unclassified
41652	9256	9255	5486	6458	12053	12371	12391	12615	Bacteria	Actinobacteria	Actinobacteria	Actinomycetales	Nocardiopsaceae	Nocardiopsis	unclassified
41733	9284	9641	8439	9295	11522	12087	11918	11412	Bacteria	Actinobacteria	Actinobacteria	Actinomycetales	Nocardiopsaceae	Nocardiopsis	unclassified
42081	8906	8762	5994	6698	12117	12552	12304	12759	Bacteria	Actinobacteria	Actinobacteria	Actinomycetales	Nocardiopsaceae	Nocardiopsis	unclassified
42218	8941	9367	6366	8001	11953	12532	12273	12134	Bacteria	Actinobacteria	Actinobacteria	Actinomycetales	Nocardiopsaceae	Nocardiopsis	unclassified
39321	9988	10070	6346	8305	11812	13029	11897	12017	Bacteria	Actinobacteria	Actinobacteria	Actinomycetales	Propionibacteriaceae	Microlunatus	Microlunatus phosphovorus
38770	10809	9420	6839	7938	13104	13190	13076	13183	Bacteria	Actinobacteria	Actinobacteria	Actinomycetales	Propionibacteriaceae	Microlunatus	unclassified
40158	12788	13425	12266	11847	14303	14163	14307	14650	Bacteria	Actinobacteria	Actinobacteria	Actinomycetales	Propionibacteriaceae	Propionibacterium	Propionibacterium acnes
40159	12404	12610	12120	11340	13438	13493	13542	13918	Bacteria	Actinobacteria	Actinobacteria	Actinomycetales	Propionibacteriaceae	Propionibacterium	Propionibacterium acnes
40162	12064	12063	10715	11004	14326	14036	14303	14413	Bacteria	Actinobacteria	Actinobacteria	Actinomycetales	Propionibacteriaceae	Propionibacterium	Propionibacterium acnes
40182	12025	11464	11030	11706	14241	13921	14202	14301	Bacteria	Actinobacteria	Actinobacteria	Actinomycetales	Propionibacteriaceae	Propionibacterium	Propionibacterium acnes
40264	12899	13159	12310	12070	14181	14048	14135	14550	Bacteria	Actinobacteria	Actinobacteria	Actinomycetales	Propionibacteriaceae	Propionibacterium	Propionibacterium acnes
40356	10219	9573	7085	9480	13612	13478	13672	13734	Bacteria	Actinobacteria	Actinobacteria	Actinomycetales	Propionibacteriaceae	Propionibacterium	Propionibacterium acnes
40550	12003	11425	7825	10865	14147	13914	14142	14218	Bacteria	Actinobacteria	Actinobacteria	Actinomycetales	Propionibacteriaceae	Propionibacterium	Propionibacterium acnes
40686	11577	9576	7628	10449	13627	13526	13691	13744	Bacteria	Actinobacteria	Actinobacteria	Actinomycetales	Propionibacteriaceae	Propionibacterium	Propionibacterium acnes
40932	11045	10797	8088	10179	13939	13690	13959	14061	Bacteria	Actinobacteria	Actinobacteria	Actinomycetales	Propionibacteriaceae	Propionibacterium	Propionibacterium acnes
41006	10270	9046	6369	9478	13327	13236	13525	13460	Bacteria	Actinobacteria	Actinobacteria	Actinomycetales	Propionibacteriaceae	Propionibacterium	Propionibacterium acnes
41012	11996	12734	11791	11698	13899	13873	13877	14222	Bacteria	Actinobacteria	Actinobacteria	Actinomycetales	Propionibacteriaceae	Propionibacterium	Propionibacterium acnes
40241	9232	9389	6766	8275	12436	12617	12710	13214	Bacteria	Actinobacteria	Actinobacteria	Actinomycetales	Propionibacteriaceae	Propionibacterium	Propionibacterium granulosum
40171	10224	9693	6275	8429	13663	13555	13868	13931	Bacteria	Actinobacteria	Actinobacteria	Actinomycetales	Propionibacteriaceae	Propionibacterium	unclassified
40188	9317	8651	7016	7660	12145	12255	12579	12411	Bacteria	Actinobacteria	Actinobacteria	Actinomycetales	Propionibacteriaceae	Propionibacterium	unclassified
40193	10102	9545	6912	8966	13008	12993	13369	13193	Bacteria	Actinobacteria	Actinobacteria	Actinomycetales	Propionibacteriaceae	Propionibacterium	unclassified
40210	11440	10035	6311	10249	13751	13485	13723	13829	Bacteria	Actinobacteria	Actinobacteria	Actinomycetales	Propionibacteriaceae	Propionibacterium	unclassified
40333	12804	13044	12795	12192	14104	14028	14141	13988	Bacteria	Actinobacteria	Actinobacteria	Actinomycetales	Propionibacteriaceae	Propionibacterium	unclassified
40518	11135	11682	8463	9576	14128	13783	14183	13980	Bacteria	Actinobacteria	Actinobacteria	Actinomycetales	Propionibacteriaceae	Propionibacterium	unclassified
40597	11068	10906	10002	10093	13106	12877	13332	13330	Bacteria	Actinobacteria	Actinobacteria	Actinomycetales	Propionibacteriaceae	Propionibacterium	unclassified
40685	13048	13214	12720	12283	14352	14162	14331	14296	Bacteria	Actinobacteria	Actinobacteria	Actinomycetales	Propionibacteriaceae	Propionibacterium	unclassified
40743	9249	8733	7998	8314	12102	12303	12485	12373	Bacteria	Actinobacteria	Actinobacteria	Actinomycetales	Propionibacteriaceae	Propionibacterium	unclassified
40766	10685	9940	8343	9292	13733	13554	13886	13966	Bacteria	Actinobacteria	Actinobacteria	Actinomycetales	Propionibacteriaceae	Propionibacterium	unclassified
40853	10524	10321	10208	10135	13197	12974	13538	13445	Bacteria	Actinobacteria	Actinobacteria	Actinomycetales	Propionibacteriaceae	Propionibacterium	unclassified
40912	10033	9231	5749	8550	12960	12639	13173	12952	Bacteria	Actinobacteria	Actinobacteria	Actinomycetales	Propionibacteriaceae	Propionibacterium	unclassified
41068	10422	10165	6116	9055	13199	13210	13543	13589	Bacteria	Actinobacteria	Actinobacteria	Actinomycetales	Propionibacteriaceae	Propionibacterium	unclassified
41085	12617	12721	12625	12161	13730	13843	13844	13814	Bacteria	Actinobacteria	Actinobacteria	Actinomycetales	Propionibacteriaceae	Propionibacterium	unclassified
40352	9349	10997	6524	7736	10088	13425	9943	10055	Bacteria	Actinobacteria	Actinobacteria	Actinomycetales	Propionibacteriaceae	Tessaracoccus	unclassified
37611	9421	11171	6595	7612	11883	12898	12146	12418	Bacteria	Actinobacteria	Actinobacteria	Actinomycetales	Pseudonocardiaceae	Amycolatopsis	unclassified
37768	10122	12025	8382	8795	12531	13453	12698	12908	Bacteria	Actinobacteria	Actinobacteria	Actinomycetales	Pseudonocardiaceae	Amycolatopsis	unclassified
37841	9047	8721	7392	7642	11872	12235	11991	12737	Bacteria	Actinobacteria	Actinobacteria	Actinomycetales	Pseudonocardiaceae	Amycolatopsis	unclassified
38094	9668	9643	7626	7171	12517	12824	12286	12438	Bacteria	Actinobacteria	Actinobacteria	Actinomycetales	Pseudonocardiaceae	Amycolatopsis	unclassified
37746	8988	8707	7083	7508	11890	12551	12059	12487	Bacteria	Actinobacteria	Actinobacteria	Actinomycetales	Pseudonocardiaceae	Pseudonocardia	unclassified
38137	8853	9155	7819	7501	12193	12845	12146	12701	Bacteria	Actinobacteria	Actinobacteria	Actinomycetales	Pseudonocardiaceae	Pseudonocardia	unclassified
38337	9153	9989	6728	6708	12936	13472	13174	13512	Bacteria	Actinobacteria	Actinobacteria	Actinomycetales	Pseudonocardiaceae	Pseudonocardia	unclassified
38349	10747	10895	10678	10389	12470	13099	12678	13065	Bacteria	Actinobacteria	Actinobacteria	Actinomycetales	Pseudonocardiaceae	Pseudonocardia	unclassified
38108	8970	9057	7918	7985	11351	12283	11677	12313	Bacteria	Actinobacteria	Actinobacteria	Actinomycetales	Pseudonocardiaceae	Saccharopolyspora	unclassified
38506	11070	11451	11090	9990	13447	13483	13262	13675	Bacteria	Actinobacteria	Actinobacteria	Actinomycetales	Streptomycetaceae	Streptomyces	unclassified
38691	9463	10253	8875	9357	10994	12262	11081	11297	Bacteria	Actinobacteria	Actinobacteria	Actinomycetales	Streptomycetaceae	Streptomyces	unclassified
38747	9466	10032	8784	9500	10931	12222	10904	11191	Bacteria	Actinobacteria	Actinobacteria	Actinomycetales	Streptomycetaceae	Streptomyces	unclassified
38755	8909	7938	6429	7259	11686	12545	11797	12058	Bacteria	Actinobacteria	Actinobacteria	Actinomycetales	Streptomycetaceae	Streptomyces	unclassified
38808	8971	9819	6630	7131	11670	12882	12074	11749	Bacteria	Actinobacteria	Actinobacteria	Actinomycetales	Streptomycetaceae	Streptomyces	unclassified
38823	8599	9928	7530	8429	10335	12702	10567	10729	Bacteria	Actinobacteria	Actinobacteria	Actinomycetales	Streptomycetaceae	Streptomyces	unclassified
38959	8796	9264	6783	7042	11606	12618	11853	12007	Bacteria	Actinobacteria	Actinobacteria	Actinomycetales	Streptomycetaceae	Streptomyces	unclassified
39084	8892	9558	7719	7721	11376	11970	11619	11850	Bacteria	Actinobacteria	Actinobacteria	Actinomycetales	Streptomycetaceae	Streptomyces	unclassified
39109	9133	9330	6795	7805	11723	12597	11814	12307	Bacteria	Actinobacteria	Actinobacteria	Actinomycetales	Streptomycetaceae	Streptomyces	unclassified
39112	9268	9333	7152	7440	11914	13027	12098	12141	Bacteria	Actinobacteria	Actinobacteria	Actinomycetales	Streptomycetaceae	Streptomyces	unclassified
39248	8474	9476	7225	7260	12274	12798	12340	12365	Bacteria	Actinobacteria	Actinobacteria	Actinomycetales	Streptomycetaceae	Streptomyces	unclassified
39401	10907	11270	10823	10537	12280	12675	12158	12215	Bacteria	Actinobacteria	Actinobacteria	Actinomycetales	Streptomycetaceae	Streptomyces	unclassified
39424	8657	10016	7659	8485	10148	12729	10348	10432	Bacteria	Actinobacteria	Actinobacteria	Actinomycetales	Streptomycetaceae	Streptomyces	unclassified
39435	8898	9481	7388	7495	11274	12599	11590	12048	Bacteria	Actinobacteria	Actinobacteria	Actinomycetales	Streptomycetaceae	Streptomyces	unclassified
39541	9160	10273	8243	8419	11626	12805	11632	11951	Bacteria	Actinobacteria	Actinobacteria	Actinomycetales	Streptomycetaceae	Streptomyces	unclassified
39549	9897	10462	9437	8653	13806	14139	13548	14089	Bacteria	Actinobacteria	Actinobacteria	Actinomycetales	Streptomycetaceae	Streptomyces	unclassified
39606	8736	8596	6558	6405	12119	12688	12298	12897	Bacteria	Actinobacteria	Actinobacteria	Actinomycetales	Streptomycetaceae	Streptomyces	unclassified
39650	9451	10737	7994	8112	12926	13530	13166	13622	Bacteria	Actinobacteria	Actinobacteria	Actinomycetales	Streptomycetaceae	Streptomyces	unclassified
39746	9505	10547	6845	7673	11619	12532	11899	12492	Bacteria	Actinobacteria	Actinobacteria	Actinomycetales	Streptomycetaceae	Streptomyces	unclassified
39802	9140	8043	5798	6942	11982	12692	12071	12190	Bacteria	Actinobacteria	Actinobacteria	Actinomycetales	Streptomycetaceae	Streptomyces	unclassified
39819	8480	9147	7040	6975	10516	11771	10900	11212	Bacteria	Actinobacteria	Actinobacteria	Actinomycetales	Streptomycetaceae	Streptomyces	unclassified
39909	9195	9918	6575	7346	11994	13216	12302	12412	Bacteria	Actinobacteria	Actinobacteria	Actinomycetales	Streptomycetaceae	Streptomyces	unclassified
40049	8813	8556	7234	7534	11176	11972	11387	11458	Bacteria	Actinobacteria	Actinobacteria	Actinomycetales	Streptomycetaceae	Streptomyces	unclassified
40126	8991	8026	6710	6526	12413	12639	12587	12889	Bacteria	Actinobacteria	Actinobacteria	Actinomycetales	Streptomycetaceae	Streptomyces	unclassified
42400	9535	8771	6148	6510	11182	12024	11161	11645	Bacteria	Actinobacteria	Actinobacteria	Actinomycetales	Streptosporangiaceae	Nonomuraea	unclassified
41542	9170	8667	6859	7835	11896	12437	12039	12361	Bacteria	Actinobacteria	Actinobacteria	Actinomycetales	Thermomonosporaceae	Actinoallomurus	Actinoallomurus spadix
42285	9364	8704	6865	8048	12062	12530	12216	12483	Bacteria	Actinobacteria	Actinobacteria	Actinomycetales	Thermomonosporaceae	Actinoallomurus	Actinoallomurus spadix
41552	10213	10124	9844	8378	10813	12088	10974	11485	Bacteria	Actinobacteria	Actinobacteria	Actinomycetales	Thermomonosporaceae	Actinocorallia	unclassified
42247	8973	8531	7260	7946	11362	12505	11352	11548	Bacteria	Actinobacteria	Actinobacteria	Actinomycetales	Thermomonosporaceae	Actinocorallia	unclassified
41194	10281	9892	8747	8431	12667	13211	12718	13010	Bacteria	Actinobacteria	Actinobacteria	Actinomycetales	Thermomonosporaceae	Actinomadura	unclassified
41332	9458	9537	8703	8585	11947	12912	12124	12456	Bacteria	Actinobacteria	Actinobacteria	Actinomycetales	Thermomonosporaceae	Actinomadura	unclassified
41428	10028	9517	6846	7105	11631	12281	11843	11893	Bacteria	Actinobacteria	Actinobacteria	Actinomycetales	Thermomonosporaceae	Actinomadura	unclassified
41589	9863	9698	8342	7860	12010	12591	12194	12336	Bacteria	Actinobacteria	Actinobacteria	Actinomycetales	Thermomonosporaceae	Actinomadura	unclassified
42129	10081	9949	9502	8511	12397	12435	12437	12523	Bacteria	Actinobacteria	Actinobacteria	Actinomycetales	Thermomonosporaceae	Actinomadura	unclassified

Table S6 (part H): HybScores of OTUs that were called present in at least one of the samples. Taxonomic classifications are based on PhyloChip taxonomy (derived from GreenGenes). Please note, that data presented here was rank normalized before performing statisical analyses presented in the manuscript.

	Sample ID (PMA)				Sample ID				taxonomic classification of representative rOTU cluster						
OTU	UR_PMA	CO_PMA	CR8_PMA	CR5_PMA	UR	CO	CR8	CR5	kingdom	phylum	class	order	family	genus	species
38400	7766	6401	5801	6083	12315	12299	12237	13052	Bacteria	Actinobacteria	Actinobacteria	Actinomycetales	Tsukamurellaceae	Tsukamurella	Tsukamurella paurometabola
50864	9408	10146	7557	7816	13325	13505	13107	13552	Bacteria	Actinobacteria	Actinobacteria	Coriobacteriales	Coriobacteriaceae	Adlercreutzia	unclassified
50184	10741	9507	8853	8259	11907	12215	11705	12641	Bacteria	Actinobacteria	Actinobacteria	Coriobacteriales	Coriobacteriaceae	Collinsella	Collinsella aerofaciens
50477	9766	8874	8069	7304	11147	12156	11523	12420	Bacteria	Actinobacteria	Actinobacteria	Coriobacteriales	Coriobacteriaceae	Collinsella	Collinsella aerofaciens
50847	9007	9229	8445	8007	10960	11787	11136	12097	Bacteria	Actinobacteria	Actinobacteria	Coriobacteriales	Coriobacteriaceae	Collinsella	Collinsella aerofaciens
41425	10920	11487	10788	9829	12029	12543	12061	12106	Bacteria	Actinobacteria	Actinobacteria	koll13	unclassified	sfB	unclassified
41992	10112	10442	10099	9682	11260	12070	11420	11628	Bacteria	Actinobacteria	Actinobacteria	koll13	unclassified	sfB	unclassified
41886	9240	9267	7841	7768	12247	12640	11908	12388	Bacteria	Actinobacteria	Actinobacteria	koll13	unclassified	sfD	unclassified
42189	9305	9847	8006	8240	11854	12147	11602	11797	Bacteria	Actinobacteria	Actinobacteria	koll13	unclassified	sfD	unclassified
41114	8838	9233	7029	7293	11993	12886	12241	12443	Bacteria	Actinobacteria	Actinobacteria	MC47	unclassified	sfA	unclassified
41227	8970	9991	7456	7529	12439	13035	12478	12928	Bacteria	Actinobacteria	Actinobacteria	MC47	unclassified	sfA	unclassified
41235	10257	10936	10552	10127	12862	13329	12745	13021	Bacteria	Actinobacteria	Actinobacteria	MC47	unclassified	sfA	unclassified
41285	9952	10621	9574	9030	12778	13280	13065	13001	Bacteria	Actinobacteria	Actinobacteria	MC47	unclassified	sfA	unclassified
41301	9151	9149	7807	6861	11913	12540	12058	12254	Bacteria	Actinobacteria	Actinobacteria	MC47	unclassified	sfA	unclassified
41639	11191	10037	8131	8274	11747	12642	11933	12484	Bacteria	Actinobacteria	Actinobacteria	MC47	unclassified	sfA	unclassified
41755	8391	8684	6583	7107	11725	12192	11753	12094	Bacteria	Actinobacteria	Actinobacteria	MC47	unclassified	sfA	unclassified
41500	10503	10947	9864	9025	13085	13374	13258	13438	Bacteria	Actinobacteria	Actinobacteria	Solirubrobacterales	Conexibacteraceae	unclassified	unclassified
41631	9050	9417	7208	7474	12554	13200	12658	12681	Bacteria	Actinobacteria	Actinobacteria	Solirubrobacterales	Conexibacteraceae	unclassified	unclassified
42131	9550	9250	7716	6875	12162	13007	12691	12597	Bacteria	Actinobacteria	Actinobacteria	Solirubrobacterales	Conexibacteraceae	unclassified	unclassified
42436	8427	9181	7916	7048	11236	12483	11423	12066	Bacteria	Actinobacteria	Actinobacteria	Solirubrobacterales	Conexibacteraceae	unclassified	unclassified
42253	8670	7006	6738	7023	11368	12121	11567	11394	Bacteria	Actinobacteria	Actinobacteria	Solirubrobacterales	Solirubrobacteraceae	Solirubrobacter	unclassified
42426	8969	8158	7708	7342	11432	12329	11742	11525	Bacteria	Actinobacteria	Actinobacteria	Solirubrobacterales	Solirubrobacteraceae	Solirubrobacter	unclassified
42095	10781	11172	10943	10539	12139	12648	12180	12783	Bacteria	Actinobacteria	Actinobacteria	Solirubrobacterales	Solirubrobacteraceae	unclassified	unclassified
42150	9583	6819	6700	7101	11082	11713	10916	10973	Bacteria	Actinobacteria	Actinobacteria	Solirubrobacterales	Solirubrobacteraceae	unclassified	unclassified
58484	9523	9718	8442	7641	11669	12479	11910	12626	Bacteria	Actinobacteria	Actinobacteria	unclassified	unclassified	sfF	unclassified
55518	8817	9548	7947	7132	12320	13217	12213	12754	Bacteria	Aquificae	Aquificae	Aquificales	Desulfurobacteriaceae	unclassified	unclassified
55774	8406	8735	7790	6977	11443	12582	11842	12174	Bacteria	Aquificae	Aquificae	Aquificales	Desulfurobacteriaceae	unclassified	unclassified
69519	8137	6688	6368	6276	9659	12266	9541	9579	Bacteria	Bacteroidetes	Bacteroidia	Bacteroidales	Marinilabiaceae	unclassified	unclassified
68573	9760	9051	6986	7344	12330	12873	12186	12789	Bacteria	Bacteroidetes	Bacteroidia	Bacteroidales	Porphyromonadaceae	unclassified	unclassified
67028	8363	7835	7169	7333	11109	11844	11625	11775	Bacteria	Bacteroidetes	Bacteroidia	Bacteroidales	Prevotellaceae	Prevotella	unclassified
67542	8594	7666	6780	7213	10897	11814	11311	11698	Bacteria	Bacteroidetes	Bacteroidia	Bacteroidales	Prevotellaceae	Prevotella	unclassified
67664	8686	7350	6704	7144	10011	11067	11728	11240	Bacteria	Bacteroidetes	Bacteroidia	Bacteroidales	Prevotellaceae	Prevotella	unclassified
68290	9160	8724	7642	8464	11414	12193	11782	11664	Bacteria	Bacteroidetes	Bacteroidia	Bacteroidales	RikenellaceaeII	unclassified	unclassified
69046	8037	8486	6569	7059	10427	12758	11362	11594	Bacteria	Bacteroidetes	Bacteroidia	Bacteroidales	RikenellaceaeII	unclassified	unclassified
69770	11334	11986	10420	10583	13781	13752	13624	13959	Bacteria	Bacteroidetes	Bacteroidia	Bacteroidales	RikenellaceaeII	unclassified	unclassified
69931	11247	11670	11519	10982	12974	13123	12608	12920	Bacteria	Bacteroidetes	Bacteroidia	Bacteroidales	RikenellaceaeII	unclassified	unclassified
65875	9249	11340	7785	6951	12748	13601	12795	12717	Bacteria	Bacteroidetes	Flavobacteria	Flavobacteriales	Corymorphaceae	unclassified	unclassified
63161	8658	8581	7045	7394	11766	11677	11512	11921	Bacteria	Bacteroidetes	Flavobacteria	Flavobacteriales	Flavobacteriaceae	Chryseobacterium	unclassified
63187	8077	6063	5886	6339	11609	11933	11630	11946	Bacteria	Bacteroidetes	Flavobacteria	Flavobacteriales	Flavobacteriaceae	Chryseobacterium	unclassified
63286	9778	7876	5980	6382	13012	12480	12329	12706	Bacteria	Bacteroidetes	Flavobacteria	Flavobacteriales	Flavobacteriaceae	Chryseobacterium	unclassified
63400	7821	6863	6111	6708	11404	11508	11502	11835	Bacteria	Bacteroidetes	Flavobacteria	Flavobacteriales	Flavobacteriaceae	Chryseobacterium	unclassified
63573	10501	10241	9158	8813	12970	13008	13048	13245	Bacteria	Bacteroidetes	Flavobacteria	Flavobacteriales	Flavobacteriaceae	Chryseobacterium	unclassified
63711	8255	7823	6152	6806	12149	11573	11057	11558	Bacteria	Bacteroidetes	Flavobacteria	Flavobacteriales	Flavobacteriaceae	Chryseobacterium	unclassified
63864	8887	7058	6219	6901	11954	11979	12260	12770	Bacteria	Bacteroidetes	Flavobacteria	Flavobacteriales	Flavobacteriaceae	Chryseobacterium	unclassified
64220	9843	8905	8566	8268	12420	12309	12368	12776	Bacteria	Bacteroidetes	Flavobacteria	Flavobacteriales	Flavobacteriaceae	Chryseobacterium	unclassified
64623	8575	8969	7755	7078	10394	11306	11497	11875	Bacteria	Bacteroidetes	Flavobacteria	Flavobacteriales	Flavobacteriaceae	Chryseobacterium	unclassified
64654	10834	8258	7005	6643	11174	11751	10990	10916	Bacteria	Bacteroidetes	Flavobacteria	Flavobacteriales	Flavobacteriaceae	Chryseobacterium	unclassified
64734	8230	7491	6699	7553	11625	11882	12009	12371	Bacteria	Bacteroidetes	Flavobacteria	Flavobacteriales	Flavobacteriaceae	Chryseobacterium	unclassified
64986	8026	6577	6077	6434	11343	11549	12015	12444	Bacteria	Bacteroidetes	Flavobacteria	Flavobacteriales	Flavobacteriaceae	Chryseobacterium	unclassified
65050	8187	7295	6682	7146	11891	11794	11923	12399	Bacteria	Bacteroidetes	Flavobacteria	Flavobacteriales	Flavobacteriaceae	Chryseobacterium	unclassified
65435	9499	9442	8814	8414	12823	12610	12410	12837	Bacteria	Bacteroidetes	Flavobacteria	Flavobacteriales	Flavobacteriaceae	Chryseobacterium	unclassified
65635	9145	9315	7137	7611	12680	12707	12926	13055	Bacteria	Bacteroidetes	Flavobacteria	Flavobacteriales	Flavobacteriaceae	Chryseobacterium	unclassified
65807	10338	10258	9348	8556	13035	12942	12724	12978	Bacteria	Bacteroidetes	Flavobacteria	Flavobacteriales	Flavobacteriaceae	Chryseobacterium	unclassified
66605	8617	9444	8094	8661	10701	12308	11756	11492	Bacteria	Bacteroidetes	Flavobacteria	Flavobacteriales	Flavobacteriaceae	Flavobacterium	Flavobacterium columnare
65912	7576	8605	6623	7368	9548	11894	10106	10639	Bacteria	Bacteroidetes	Flavobacteria	Flavobacteriales	Flavobacteriaceae	Flavobacterium	unclassified
66715	7764	10341	6223	6866	11119	12539	12159	12265	Bacteria	Bacteroidetes	Flavobacteria	Flavobacteriales	Flavobacteriaceae	Flavobacterium	unclassified
63093	10296	10786	9876	9217	13640	13493	13161	13445	Bacteria	Bacteroidetes	Flavobacteria	Flavobacteriales	Flavobacteriaceae	unclassified	unclassified
63182	8314	7488	6946	6747	12518	12357	11811	11779	Bacteria	Bacteroidetes	Flavobacteria	Flavobacteriales	Flavobacteriaceae	unclassified	unclassified
63336	10942	11590	11053	9296	12574	12816	12522	12926	Bacteria	Bacteroidetes	Flavobacteria	Flavobacteriales	Flavobacteriaceae	unclassified	unclassified
64568	9164	9094	7791	7501	13121	12633	12279	12616	Bacteria	Bacteroidetes	Flavobacteria	Flavobacteriales	Flavobacteriaceae	unclassified	unclassified
66457	9528	6674	6077	6450	12322	10814	10210	10684	Bacteria	Bacteroidetes	Flavobacteria	Flavobacteriales	Flavobacteriaceae	Wautersiella	unclassified
66513	9514	7770	6177	7003	12685	12086	11320	11366	Bacteria	Bacteroidetes	Flavobacteria	Flavobacteriales	Flavobacteriaceae	Wautersiella	unclassified
61277	8978	9678	7006	7314	12057	13249	11944	13067	Bacteria	Bacteroidetes	Sphingobacteria	Sphingobacteriales	Chitinophagaceae	unclassified	unclassified
74655	7996	9886	8331	6995	9950	11981	10778	10421	Bacteria	Bacteroidetes	Sphingobacteria	Sphingobacteriales	Chitinophagaceae	unclassified	unclassified
74948	8760	8493	9105	7162	11286	12550	12115	11875	Bacteria	Bacteroidetes	Sphingobacteria	Sphingobacteriales	Chitinophagaceae	unclassified	unclassified
75676	8714	9654	8576	8179	10999	12509	11654	11887	Bacteria	Bacteroidetes	Sphingobacteria	Sphingobacteriales	Chitinophagaceae	unclassified	unclassified
73923	8219	8512	7499	6916	12090	12801	12187	12412	Bacteria	Bacteroidetes	Sphingobacteria	Sphingobacteriales	Flammeovirgaceae	Fulvivirga	unclassified
73689	10302	7874	8400	7109	12415	13421	12866	13388	Bacteria	Bacteroidetes	Sphingobacteria	Sphingobacteriales	Flexibacteraceae	Cytophaga	unclassified
73168	8349	7169	6245	7712	11350	11846	11748	12009	Bacteria	Bacteroidetes	Sphingobacteria	Sphingobacteriales	Flexibacteraceae	Dyadobacter	unclassified
73514	8484	8987	7721	8839	12998	13782	13322	13876	Bacteria	Bacteroidetes	Sphingobacteria	Sphingobacteriales	Flexibacteraceae	Hymenobacter	unclassified
74031	8548	8043	7215	7844	11585	12838	12097	12802	Bacteria	Bacteroidetes	Sphingobacteria	Sphingobacteriales	Flexibacteraceae	Hymenobacter	unclassified
74065	9728	10231	9473	9105	11440	12462	12078	12255	Bacteria	Bacteroidetes	Sphingobacteria	Sphingobacteriales	Flexibacteraceae	Hymenobacter	unclassified
74285	9041	7944	7880	9197	11343	12986	11100	12571	Bacteria	Bacteroidetes	Sphingobacteria	Sphingobacteriales	Flexibacteraceae	Hymenobacter	unclassified
73491	9683	10277	8384	8389	11632	12634	12033	12616	Bacteria	Bacteroidetes	Sphingobacteria	Sphingobacteriales	Flexibacteraceae	Runella	unclassified
73871	8023	8355	7079	7853	12573	13146	12733	13129	Bacteria	Bacteroidetes	Sphingobacteria	Sphingobacteriales	Flexibacteraceae	unclassified	unclassified
56623	8686	8829	8250	7607	10791	12435	11402	11491	Bacteria	Bacteroidetes	Sphingobacteria	Sphingobacteriales	Rhodothermaceae	unclassified	unclassified
56900	12751	12992	12487	12488	14283	14092	14107	14354	Bacteria	Bacteroidetes	Sphingobacteria	Sphingobacteriales	Rhodothermaceae	unclassified	unclassified
57707	8478	8390	7141	7676	11378	12209	11393	11695	Bacteria	Bacteroidetes	Sphingobacteria	Sphingobacteriales	Rhodothermaceae	unclassified	unclassified
75605	8505	9003	7467	7465	11723	12253	10988	11022	Bacteria	Bacteroidetes	Sphingobacteria	Sphingobacteriales	Saprospiraceae	unclassified	unclassified
66896	8144	7628	7077	7571	12297	11053	10561	10244	Bacteria	Bacteroidetes	Sphingobacteria	Sphingobacteriales	Sphingobacteriaceae	Sphingobacterium	unclassified
53690	12099	12732	12657	11973	13364	13553	13385	13796	Bacteria	Chloroflexi	Anaerolineae	Anaerolineales	Anaerolinaceae	A4b	unclassified
52305	9666	10660	10003	9917	11998	12308	11855	12126	Bacteria	Chloroflexi	Chloroflexi	Chloroflexales	Chloroflexaceae	Roseiflexus	unclassified
52201	11061	11820	11463	10071	13213	13572	13386	13514	Bacteria	Chloroflexi	Thermomicrobia	Sphaerobacterales	Sphaerobacterineae	HN1-15	unclassified
52805	8540	8349	6408	7517	11000	12486	11648	11813	Bacteria	Chloroflexi	Thermomicrobia	Sphaerobacterales	Sphaerobacterineae	HN1-15	unclassified
56168	9528	10282	9770	8361	11948	12366	11337	11683	Bacteria	Chloroflexi	unclassified	unclassified	unclassified	sfB	unclassified
75099	9300	9849	7982	8407	11480	12674	11858	12184	Bacteria	Cyanobacteria	Chloroplast	Euglenozoa	unclassified	sfA	unclassified
75661	9455	10001	7053	7519	12102	12539	12101	12087	Bacteria	Cyanobacteria	Chloroplast	Euglenozoa	unclassified	sfJ	unclassified
75644	7690	8531	7140	6815	11567	12807	11848	12611	Bacteria	Cyanobacteria	Chloroplast	Streptophyta	unclassified	sfA	unclassified
75878	9678	10200	9267	8736	11630	13002	12394	12495	Bacteria	Cyanobacteria	Chloroplast	Streptophyta	unclassified	sfA	unclassified
74689	8695	9611	8186	6931	11144	12095	11459	11997	Bacteria	Cyanobacteria	Chloroplast	unclassified	unclassified	sfJ	unclassified
53292	8724	9410	7746	7469	11073	12412	11142	11966	Bacteria	Cyanobacteria	Nostocophycideae	Nostocales	Nostocaceae	Anabaena	unclassified
53583	9113	8437	6772	6803	10373	12029	10757	11310	Bacteria	Cyanobacteria	Nostocophycideae	Nostocales	Nostocaceae	Aphanizomenon	Aphanizomenon issatschenkoi
53704	7898	9154	7234	7162	10619	12016	10512	11583	Bacteria	Cyanobacteria	Nostocophycideae	Nostocales	Nostocaceae	unclassified	unclassified
53368	8605	7424	7468	6923	10606	12108	11895	12178	Bacteria	Cyanobacteria	Nostocophycideae	Stigonematales	Fischerellaceae	Fischerella	Fischerella muscicola
52935	9809	11005	7686	8758	12533	13409	12863	13210	Bacteria	Cyanobacteria	Oscillatoriophycideae	Chroococcales	Xenococcaceae	Chroococcidiopsis	unclassified
53678	9431	9623	8774	8111	12761	13406	12826	13049	Bacteria	Cyanobacteria	Oscillatoriophycideae	Oscillatoriales	Phormidiaceae	Phormidium	Phormidium animale
53818	9827	9469	8463	6473	10246	11986	10853	11300	Bacteria	Cyanobacteria	Oscillatoriophycideae	Oscillatoriales	Phormidiaceae	Symploca	unclassified
53967	8898	10342	7963	7500	12070	12984	12560	12775	Bacteria	Cyanobacteria	Oscillatoriophycideae	unclassified	unclassified	sfB	unclassified
53955	11440	11884	12222	10669	13016	13200	12873	13246	Bacteria	Cyanobacteria	S15B-MN24	unclassified	unclassified	sfB	unclassified
53672	8808	10502	7510	7554	11306	12785	11868	12055	Bacteria	Cyanobacteria	Synechococcophycideae	Pseudanabaenales	Pseudanabaenaceae	Halomicronema	unclassified

Table S6 (part I): HybScores of OTUs that were called present in at least one of the samples. Taxonomic classifications are based on PhyloChip taxonomy (derived from GreenGenes). Please note, that data presented here was rank normalized before performing statisical analyses presented in the manuscript.

	Sample ID (PMA)				Sample ID				taxonomic classification of representative rOTU cluster						
OTU	UR_PMA	CO_PMA	CR8_PMA	CR5_PMA	UR	CO	CR8	CR5	kingdom	phylum	class	order	family	genus	species
49516	8906	9900	8022	7842	11109	12019	12712	11806	Bacteria	Firmicutes	Bacilli	Bacillales	Bacillaceae	Bacillus	Bacillus acidicola
49665	8733	10178	8433	7751	10909	11969	11806	11423	Bacteria	Firmicutes	Bacilli	Bacillales	Bacillaceae	Bacillus	Bacillus acidicola
43709	8004	9457	7867	7551	11820	10738	9946	10335	Bacteria	Firmicutes	Bacilli	Bacillales	Bacillaceae	Bacillus	Bacillus anthracis
46520	10241	11797	10197	9351	12617	12700	12077	11854	Bacteria	Firmicutes	Bacilli	Bacillales	Bacillaceae	Bacillus	Bacillus cytotoxicus
45955	11345	12035	10981	10457	13083	12854	12856	12891	Bacteria	Firmicutes	Bacilli	Bacillales	Bacillaceae	Bacillus	Bacillus flexus
42514	7897	9445	7830	7579	11721	10723	10132	10356	Bacteria	Firmicutes	Bacilli	Bacillales	Bacillaceae	Bacillus	unclassified
42609	11096	11685	10906	10643	12178	12416	12324	12291	Bacteria	Firmicutes	Bacilli	Bacillales	Bacillaceae	Bacillus	unclassified
42672	7605	9280	6705	7390	11761	10930	9781	10345	Bacteria	Firmicutes	Bacilli	Bacillales	Bacillaceae	Bacillus	unclassified
42684	7740	9320	7774	7249	11540	10772	9887	10114	Bacteria	Firmicutes	Bacilli	Bacillales	Bacillaceae	Bacillus	unclassified
42748	7988	9161	7706	7384	11914	11000	10210	10411	Bacteria	Firmicutes	Bacilli	Bacillales	Bacillaceae	Bacillus	unclassified
42933	8850	10131	8600	7168	12585	12054	11909	12005	Bacteria	Firmicutes	Bacilli	Bacillales	Bacillaceae	Bacillus	unclassified
43361	7702	9176	7683	7281	11661	10904	9984	10355	Bacteria	Firmicutes	Bacilli	Bacillales	Bacillaceae	Bacillus	unclassified
43461	8011	9251	7746	7542	11885	11010	10217	10395	Bacteria	Firmicutes	Bacilli	Bacillales	Bacillaceae	Bacillus	unclassified
43496	8169	10527	6671	7358	12012	11502	9843	10605	Bacteria	Firmicutes	Bacilli	Bacillales	Bacillaceae	Bacillus	unclassified
43571	8873	9871	8094	7491	12476	11512	11433	11453	Bacteria	Firmicutes	Bacilli	Bacillales	Bacillaceae	Bacillus	unclassified
43705	8374	11019	7771	7068	10835	11875	11685	10991	Bacteria	Firmicutes	Bacilli	Bacillales	Bacillaceae	Bacillus	unclassified
43740	7533	9156	6484	7379	11721	10772	9406	9941	Bacteria	Firmicutes	Bacilli	Bacillales	Bacillaceae	Bacillus	unclassified
43755	9013	10012	7548	7623	11959	11489	11333	11338	Bacteria	Firmicutes	Bacilli	Bacillales	Bacillaceae	Bacillus	unclassified
44069	7456	9126	6506	7285	11658	10748	9047	9849	Bacteria	Firmicutes	Bacilli	Bacillales	Bacillaceae	Bacillus	unclassified
47078	8908	10235	7911	8060	10486	11539	12007	11979	Bacteria	Firmicutes	Bacilli	Bacillales	Bacillaceae	Bacillus	unclassified
48213	9111	13413	9514	9086	10563	13320	11001	10894	Bacteria	Firmicutes	Bacilli	Bacillales	Bacillaceae	Bacillus	unclassified
48215	11383	13204	11249	11311	12355	13776	12545	12613	Bacteria	Firmicutes	Bacilli	Bacillales	Bacillaceae	Bacillus	unclassified
48279	8162	13433	8987	8519	9293	13411	8950	9527	Bacteria	Firmicutes	Bacilli	Bacillales	Bacillaceae	Bacillus	unclassified
48318	8646	12927	7910	7878	10370	12665	10531	10954	Bacteria	Firmicutes	Bacilli	Bacillales	Bacillaceae	Bacillus	unclassified
48364	8196	13171	7228	6958	8562	13425	8081	9089	Bacteria	Firmicutes	Bacilli	Bacillales	Bacillaceae	Bacillus	unclassified
48388	8705	13471	8564	8741	9942	13605	9795	10468	Bacteria	Firmicutes	Bacilli	Bacillales	Bacillaceae	Bacillus	unclassified
48423	8258	13208	8507	7952	10536	13454	10830	10925	Bacteria	Firmicutes	Bacilli	Bacillales	Bacillaceae	Bacillus	unclassified
48440	9063	13247	8309	8969	10191	13463	10186	10610	Bacteria	Firmicutes	Bacilli	Bacillales	Bacillaceae	Bacillus	unclassified
48497	8383	13191	8913	8049	10525	13147	10881	10726	Bacteria	Firmicutes	Bacilli	Bacillales	Bacillaceae	Bacillus	unclassified
48536	8947	13611	9258	8949	10368	13435	10792	10764	Bacteria	Firmicutes	Bacilli	Bacillales	Bacillaceae	Bacillus	unclassified
48581	8445	13364	8416	8192	9780	13218	10011	10298	Bacteria	Firmicutes	Bacilli	Bacillales	Bacillaceae	Bacillus	unclassified
48645	8286	12513	8202	7066	10707	12807	11047	10994	Bacteria	Firmicutes	Bacilli	Bacillales	Bacillaceae	Bacillus	unclassified
48660	8791	12788	7998	8164	11359	13395	11249	11437	Bacteria	Firmicutes	Bacilli	Bacillales	Bacillaceae	Bacillus	unclassified
48662	8715	13373	8828	8941	9870	13409	9262	10237	Bacteria	Firmicutes	Bacilli	Bacillales	Bacillaceae	Bacillus	unclassified
48675	10329	13193	10192	9862	12258	13573	12175	12148	Bacteria	Firmicutes	Bacilli	Bacillales	Bacillaceae	Bacillus	unclassified
48690	7445	11855	7456	7245	10802	12416	10976	10960	Bacteria	Firmicutes	Bacilli	Bacillales	Bacillaceae	Bacillus	unclassified
48691	8805	13274	8574	8108	10905	13051	10990	11171	Bacteria	Firmicutes	Bacilli	Bacillales	Bacillaceae	Bacillus	unclassified
48710	8806	12931	6960	7614	10383	12836	10413	10612	Bacteria	Firmicutes	Bacilli	Bacillales	Bacillaceae	Bacillus	unclassified
48776	12521	13023	12698	12542	13659	13401	13796	13661	Bacteria	Firmicutes	Bacilli	Bacillales	Bacillaceae	Bacillus	unclassified
48784	8345	12851	7149	7492	10726	12797	10979	11219	Bacteria	Firmicutes	Bacilli	Bacillales	Bacillaceae	Bacillus	unclassified
48805	8398	13152	9096	8165	11150	13088	11107	11357	Bacteria	Firmicutes	Bacilli	Bacillales	Bacillaceae	Bacillus	unclassified
48819	8773	12987	7740	7557	11299	12711	11764	12036	Bacteria	Firmicutes	Bacilli	Bacillales	Bacillaceae	Bacillus	unclassified
48821	8799	13435	8916	8655	10843	13514	11205	11264	Bacteria	Firmicutes	Bacilli	Bacillales	Bacillaceae	Bacillus	unclassified
48825	10089	11817	9967	9268	13069	13186	12908	12704	Bacteria	Firmicutes	Bacilli	Bacillales	Bacillaceae	Bacillus	unclassified
48851	9174	13156	8924	8951	10452	13155	10365	10737	Bacteria	Firmicutes	Bacilli	Bacillales	Bacillaceae	Bacillus	unclassified
48861	8712	13016	8644	8549	10045	13056	9657	10318	Bacteria	Firmicutes	Bacilli	Bacillales	Bacillaceae	Bacillus	unclassified
48936	7281	12691	7088	6913	10361	12800	9869	10013	Bacteria	Firmicutes	Bacilli	Bacillales	Bacillaceae	Bacillus	unclassified
48975	9095	12973	8722	8704	10258	12969	10161	10628	Bacteria	Firmicutes	Bacilli	Bacillales	Bacillaceae	Bacillus	unclassified
48981	8354	12819	8437	7681	10811	12681	10872	11131	Bacteria	Firmicutes	Bacilli	Bacillales	Bacillaceae	Bacillus	unclassified
49022	8950	13029	8924	8301	11080	12851	11148	11358	Bacteria	Firmicutes	Bacilli	Bacillales	Bacillaceae	Bacillus	unclassified
49050	10091	12530	10416	9935	12871	13772	12768	13054	Bacteria	Firmicutes	Bacilli	Bacillales	Bacillaceae	Bacillus	unclassified
49052	8326	13310	8713	7984	10361	13283	10708	10629	Bacteria	Firmicutes	Bacilli	Bacillales	Bacillaceae	Bacillus	unclassified
49107	8354	12819	8437	7681	10811	12681	10872	11131	Bacteria	Firmicutes	Bacilli	Bacillales	Bacillaceae	Bacillus	unclassified
49129	8384	13591	7360	7938	9612	13691	9601	10356	Bacteria	Firmicutes	Bacilli	Bacillales	Bacillaceae	Bacillus	unclassified
49160	9202	13405	9423	9374	10395	13458	10334	10719	Bacteria	Firmicutes	Bacilli	Bacillales	Bacillaceae	Bacillus	unclassified
49167	8077	13427	8415	7297	10188	13319	10435	10477	Bacteria	Firmicutes	Bacilli	Bacillales	Bacillaceae	Bacillus	unclassified
49168	8604	13148	8205	7986	10740	12938	10932	11228	Bacteria	Firmicutes	Bacilli	Bacillales	Bacillaceae	Bacillus	unclassified
49172	8904	13538	9037	8754	10032	13546	9585	10384	Bacteria	Firmicutes	Bacilli	Bacillales	Bacillaceae	Bacillus	unclassified
49178	9224	13760	9514	9287	10598	13574	11042	10984	Bacteria	Firmicutes	Bacilli	Bacillales	Bacillaceae	Bacillus	unclassified
49187	9145	13443	8990	8813	10522	13388	11049	11175	Bacteria	Firmicutes	Bacilli	Bacillales	Bacillaceae	Bacillus	unclassified
49214	8360	13583	8792	8263	9768	13443	9540	9917	Bacteria	Firmicutes	Bacilli	Bacillales	Bacillaceae	Bacillus	unclassified
49265	9765	13419	9296	8883	11563	13723	11754	11951	Bacteria	Firmicutes	Bacilli	Bacillales	Bacillaceae	Bacillus	unclassified
49268	10423	12990	10516	10793	12073	13450	12009	12153	Bacteria	Firmicutes	Bacilli	Bacillales	Bacillaceae	Bacillus	unclassified
49305	9345	12680	8842	8062	12339	12874	12485	12432	Bacteria	Firmicutes	Bacilli	Bacillales	Bacillaceae	Bacillus	unclassified
49312	7713	9936	6969	7211	10657	11787	10233	10160	Bacteria	Firmicutes	Bacilli	Bacillales	Bacillaceae	Bacillus	unclassified
49322	11839	12720	12183	11389	13662	13207	13446	12924	Bacteria	Firmicutes	Bacilli	Bacillales	Bacillaceae	Bacillus	unclassified
49325	8766	13436	8684	8532	10298	13183	10708	10846	Bacteria	Firmicutes	Bacilli	Bacillales	Bacillaceae	Bacillus	unclassified
49357	8715	13373	8828	8941	9870	13409	9262	10237	Bacteria	Firmicutes	Bacilli	Bacillales	Bacillaceae	Bacillus	unclassified
49365	8742	13631	8789	8843	10361	13695	10291	10632	Bacteria	Firmicutes	Bacilli	Bacillales	Bacillaceae	Bacillus	unclassified
49366	8451	13371	8086	7900	10004	12973	9518	10085	Bacteria	Firmicutes	Bacilli	Bacillales	Bacillaceae	Bacillus	unclassified
49395	8358	13310	8604	8090	10187	13255	10435	10424	Bacteria	Firmicutes	Bacilli	Bacillales	Bacillaceae	Bacillus	unclassified
49446	8947	13611	9258	8949	10368	13435	10792	10764	Bacteria	Firmicutes	Bacilli	Bacillales	Bacillaceae	Bacillus	unclassified
49493	9032	13547	9440	9090	9980	13309	10310	10330	Bacteria	Firmicutes	Bacilli	Bacillales	Bacillaceae	Bacillus	unclassified
49495	9088	13413	9204	9091	10244	13474	10160	10558	Bacteria	Firmicutes	Bacilli	Bacillales	Bacillaceae	Bacillus	unclassified
49532	8587	12717	8217	7919	10529	12695	10296	10903	Bacteria	Firmicutes	Bacilli	Bacillales	Bacillaceae	Bacillus	unclassified
49591	8684	13709	8408	8738	10149	13767	9685	10532	Bacteria	Firmicutes	Bacilli	Bacillales	Bacillaceae	Bacillus	unclassified
49623	8471	13216	8510	7883	10341	12926	10309	10588	Bacteria	Firmicutes	Bacilli	Bacillales	Bacillaceae	Bacillus	unclassified
49637	8924	13692	9441	9104	10062	13541	10329	10398	Bacteria	Firmicutes	Bacilli	Bacillales	Bacillaceae	Bacillus	unclassified
49669	8437	13062	7178	7491	9377	13316	8775	9970	Bacteria	Firmicutes	Bacilli	Bacillales	Bacillaceae	Bacillus	unclassified
49688	8581	12845	8084	7923	10530	12475	10694	10984	Bacteria	Firmicutes	Bacilli	Bacillales	Bacillaceae	Bacillus	unclassified
49766	8823	12818	6954	7545	12023	13421	11263	11484	Bacteria	Firmicutes	Bacilli	Bacillales	Bacillaceae	Bacillus	unclassified
49864	8423	12941	7522	8280	12086	13614	11948	11815	Bacteria	Firmicutes	Bacilli	Bacillales	Bacillaceae	Bacillus	unclassified
49874	11551	12003	11296	10467	13598	13977	13835	13961	Bacteria	Firmicutes	Bacilli	Bacillales	Bacillaceae	Bacillus	unclassified
49912	10213	13176	9883	9464	12213	13583	12238	11908	Bacteria	Firmicutes	Bacilli	Bacillales	Bacillaceae	Bacillus	unclassified
49937	8982	13563	9026	8992	10113	13535	10036	10486	Bacteria	Firmicutes	Bacilli	Bacillales	Bacillaceae	Bacillus	unclassified
46868	8187	9045	6975	7235	11121	11845	11463	11966	Bacteria	Firmicutes	Bacilli	Bacillales	Bacillaceae	Geobacillus	unclassified
47434	9090	9769	8696	8432	11164	12346	11657	11585	Bacteria	Firmicutes	Bacilli	Bacillales	Bacillaceae	Geobacillus	unclassified
47628	10692	11226	10585	10114	11861	12487	12296	12229	Bacteria	Firmicutes	Bacilli	Bacillales	Bacillaceae	Geobacillus	unclassified
47450	8432	9238	6870	6831	11183	11415	11544	11352	Bacteria	Firmicutes	Bacilli	Bacillales	Bacillaceae	Pontibacillus	unclassified
46079	11588	12613	12046	11897	13576	12863	13186	13547	Bacteria	Firmicutes	Bacilli	Bacillales	Listeriaceae	Brochothrix	Brochothrix thermosphacta
47960	8875	9956	8749	8419	10932	12075	12506	12502	Bacteria	Firmicutes	Bacilli	Bacillales	Listeriaceae	Brochothrix	Brochothrix thermosphacta
46095	8368	8636	7494	7642	10577	11579	12366	11715	Bacteria	Firmicutes	Bacilli	Bacillales	Listeriaceae	Brochothrix	unclassified
50031	8633	10165	10832	7554	11288	11664	12688	12286	Bacteria	Firmicutes	Bacilli	Bacillales	Paenibacillaceae	Paenibacillus	unclassified
50100	8214	10902	9239	7562	10494	11444	11643	11545	Bacteria	Firmicutes	Bacilli	Bacillales	Paenibacillaceae	Paenibacillus	unclassified
50102	8248	9286	8178	6434	10893	11970	12250	11762	Bacteria	Firmicutes	Bacilli	Bacillales	Paenibacillaceae	Paenibacillus	unclassified
50179	8784	9624	10015	6054	11069	11604	13058	12043	Bacteria	Firmicutes	Bacilli	Bacillales	Paenibacillaceae	Paenibacillus	unclassified
50382	9206	10651	8258	6884	11787	12581	12298	11946	Bacteria	Firmicutes	Bacilli	Bacillales	Paenibacillaceae	Paenibacillus	unclassified
50392	8699	11026	11160	7473	11425	11466	12794	12419	Bacteria	Firmicutes	Bacilli	Bacillales	Paenibacillaceae	Paenibacillus	unclassified
50648	9058	9927	10315	7209	11020	11322	12225	11862	Bacteria	Firmicutes	Bacilli	Bacillales	Paenibacillaceae	Paenibacillus	unclassified
50779	8161	10342	11394	6934	10810	11219	11744	11468	Bacteria	Firmicutes	Bacilli	Bacillales	Paenibacillaceae	Paenibacillus	unclassified

Table S6 (part J): HybScores of OTUs that were called present in at least one of the samples. Taxonomic classifications are based on PhyloChip taxonomy (derived from GreenGenes). Please note, that data presented here was rank normalized before performing statisical analyses presented in the manuscript.

	Sample ID (PMA)				Sample ID				taxonomic classification of representative rOTU cluster						
OTU	UR_PMA	CO_PMA	CR8_PMA	CR5_PMA	UR	CO	CR8	CR5	kingdom	phylum	class	order	family	genus	species
50857	8088	9874	10503	7425	11118	11768	12428	11669	Bacteria	Firmicutes	Bacilli	Bacillales	Paenibacillaceae	Paenibacillus	unclassified
50911	8437	10183	10084	6677	11728	12723	13621	12707	Bacteria	Firmicutes	Bacilli	Bacillales	Paenibacillaceae	Paenibacillus	unclassified
42472	8480	7571	6431	6580	11052	11935	11877	12221	Bacteria	Firmicutes	Bacilli	Bacillales	Planococcaceae	Bacillus	Bacillus psychrodurans
42470	9166	9281	8026	7400	11504	11148	12082	11677	Bacteria	Firmicutes	Bacilli	Bacillales	Planococcaceae	Solibacillus	unclassified
43752	10097	11541	10399	10128	12269	12234	12040	12456	Bacteria	Firmicutes	Bacilli	Bacillales	Planococcaceae	Solibacillus	unclassified
43416	9026	9562	8229	7723	11912	12752	12168	12642	Bacteria	Firmicutes	Bacilli	Bacillales	Planococcaceae	unclassified	unclassified
43095	8793	9014	8620	6723	12562	13056	13271	12949	Bacteria	Firmicutes	Bacilli	Bacillales	Staphylococcaceae	Jeotgalicoccus	unclassified
43749	9145	10099	8631	7780	13161	13379	13269	13210	Bacteria	Firmicutes	Bacilli	Bacillales	Staphylococcaceae	Jeotgalicoccus	unclassified
44033	8871	9844	7973	7376	12504	13050	13139	13127	Bacteria	Firmicutes	Bacilli	Bacillales	Staphylococcaceae	Jeotgalicoccus	unclassified
48021	9263	8382	7486	7545	11566	10929	12230	12225	Bacteria	Firmicutes	Bacilli	Bacillales	Staphylococcaceae	Staphylococcus	Staphylococcus arlettae
45914	10672	10362	7275	7298	13396	12871	13861	13629	Bacteria	Firmicutes	Bacilli	Bacillales	Staphylococcaceae	Staphylococcus	Staphylococcus epidermidis
45951	10347	10758	7480	6929	13320	12756	13727	13456	Bacteria	Firmicutes	Bacilli	Bacillales	Staphylococcaceae	Staphylococcus	Staphylococcus epidermidis
45953	11404	11148	10270	10423	12878	12510	13416	13274	Bacteria	Firmicutes	Bacilli	Bacillales	Staphylococcaceae	Staphylococcus	Staphylococcus epidermidis
46025	10840	10310	6609	7066	13447	12674	13711	13419	Bacteria	Firmicutes	Bacilli	Bacillales	Staphylococcaceae	Staphylococcus	Staphylococcus epidermidis
46067	10588	11031	8649	8908	13020	13288	13472	13530	Bacteria	Firmicutes	Bacilli	Bacillales	Staphylococcaceae	Staphylococcus	Staphylococcus epidermidis
46138	10646	10196	6991	7443	13575	13038	13998	13831	Bacteria	Firmicutes	Bacilli	Bacillales	Staphylococcaceae	Staphylococcus	Staphylococcus epidermidis
46162	10663	10822	7546	7370	13463	12962	13899	13683	Bacteria	Firmicutes	Bacilli	Bacillales	Staphylococcaceae	Staphylococcus	Staphylococcus epidermidis
46163	11261	10748	6955	7719	13732	13096	13920	13807	Bacteria	Firmicutes	Bacilli	Bacillales	Staphylococcaceae	Staphylococcus	Staphylococcus epidermidis
46189	11554	12280	10562	10004	13922	13545	14014	14186	Bacteria	Firmicutes	Bacilli	Bacillales	Staphylococcaceae	Staphylococcus	Staphylococcus epidermidis
46215	10511	10366	9158	8941	12734	12904	12889	12949	Bacteria	Firmicutes	Bacilli	Bacillales	Staphylococcaceae	Staphylococcus	Staphylococcus epidermidis
46217	11089	10323	7257	7784	13637	12981	13848	13672	Bacteria	Firmicutes	Bacilli	Bacillales	Staphylococcaceae	Staphylococcus	Staphylococcus epidermidis
46226	9957	11170	7987	7641	13401	12638	13303	13093	Bacteria	Firmicutes	Bacilli	Bacillales	Staphylococcaceae	Staphylococcus	Staphylococcus epidermidis
46243	10668	11342	9082	8963	13253	13011	13372	13265	Bacteria	Firmicutes	Bacilli	Bacillales	Staphylococcaceae	Staphylococcus	Staphylococcus epidermidis
46244	10023	11713	9255	7914	13477	13260	13717	13843	Bacteria	Firmicutes	Bacilli	Bacillales	Staphylococcaceae	Staphylococcus	Staphylococcus epidermidis
46260	11251	11120	7839	8839	13847	13434	13941	13814	Bacteria	Firmicutes	Bacilli	Bacillales	Staphylococcaceae	Staphylococcus	Staphylococcus epidermidis
46293	10981	10582	7091	7969	13402	12891	13740	13553	Bacteria	Firmicutes	Bacilli	Bacillales	Staphylococcaceae	Staphylococcus	Staphylococcus epidermidis
46372	9694	10511	9506	8424	12224	12559	12685	12106	Bacteria	Firmicutes	Bacilli	Bacillales	Staphylococcaceae	Staphylococcus	Staphylococcus epidermidis
46405	10211	10918	11137	8054	12618	12345	13493	13231	Bacteria	Firmicutes	Bacilli	Bacillales	Staphylococcaceae	Staphylococcus	Staphylococcus epidermidis
46417	11061	10486	7196	7804	13546	13023	13876	13720	Bacteria	Firmicutes	Bacilli	Bacillales	Staphylococcaceae	Staphylococcus	Staphylococcus epidermidis
46450	10691	10904	7573	7415	13368	12769	13714	13488	Bacteria	Firmicutes	Bacilli	Bacillales	Staphylococcaceae	Staphylococcus	Staphylococcus epidermidis
46451	10277	9807	7500	7530	13341	12890	13618	13274	Bacteria	Firmicutes	Bacilli	Bacillales	Staphylococcaceae	Staphylococcus	Staphylococcus epidermidis
46516	10874	10701	7016	7469	13520	12973	13890	13710	Bacteria	Firmicutes	Bacilli	Bacillales	Staphylococcaceae	Staphylococcus	Staphylococcus epidermidis
46562	10725	10985	6005	7258	13898	13416	14206	14152	Bacteria	Firmicutes	Bacilli	Bacillales	Staphylococcaceae	Staphylococcus	Staphylococcus epidermidis
46568	10252	10231	6683	7212	12819	12272	13478	13147	Bacteria	Firmicutes	Bacilli	Bacillales	Staphylococcaceae	Staphylococcus	Staphylococcus epidermidis
46571	11253	10444	7118	7781	13576	12904	13819	13603	Bacteria	Firmicutes	Bacilli	Bacillales	Staphylococcaceae	Staphylococcus	Staphylococcus epidermidis
46607	10526	10214	6758	7258	13407	12667	13656	13384	Bacteria	Firmicutes	Bacilli	Bacillales	Staphylococcaceae	Staphylococcus	Staphylococcus epidermidis
46611	11061	10401	6820	7636	13644	12911	13825	13609	Bacteria	Firmicutes	Bacilli	Bacillales	Staphylococcaceae	Staphylococcus	Staphylococcus epidermidis
46612	10685	9964	7737	8800	13605	13407	13576	13125	Bacteria	Firmicutes	Bacilli	Bacillales	Staphylococcaceae	Staphylococcus	Staphylococcus epidermidis
46667	11560	11320	7930	8640	13886	13378	13926	13838	Bacteria	Firmicutes	Bacilli	Bacillales	Staphylococcaceae	Staphylococcus	Staphylococcus epidermidis
46682	10754	10322	6737	7372	13450	12682	13710	13416	Bacteria	Firmicutes	Bacilli	Bacillales	Staphylococcaceae	Staphylococcus	Staphylococcus epidermidis
46684	10674	10270	6974	7508	13496	12853	13821	13653	Bacteria	Firmicutes	Bacilli	Bacillales	Staphylococcaceae	Staphylococcus	Staphylococcus epidermidis
46712	10722	10817	7666	7412	13623	13011	13941	13731	Bacteria	Firmicutes	Bacilli	Bacillales	Staphylococcaceae	Staphylococcus	Staphylococcus epidermidis
46718	10865	10301	7173	7816	13025	12472	13675	13291	Bacteria	Firmicutes	Bacilli	Bacillales	Staphylococcaceae	Staphylococcus	Staphylococcus epidermidis
46752	10854	10211	7197	7645	13487	12852	13792	13587	Bacteria	Firmicutes	Bacilli	Bacillales	Staphylococcaceae	Staphylococcus	Staphylococcus epidermidis
46779	10131	9877	6943	8080	12450	12462	13046	12746	Bacteria	Firmicutes	Bacilli	Bacillales	Staphylococcaceae	Staphylococcus	Staphylococcus epidermidis
46794	10028	10465	8330	8138	12556	12311	12816	12585	Bacteria	Firmicutes	Bacilli	Bacillales	Staphylococcaceae	Staphylococcus	Staphylococcus epidermidis
46819	10304	10341	8860	7922	12851	12795	13308	13245	Bacteria	Firmicutes	Bacilli	Bacillales	Staphylococcaceae	Staphylococcus	Staphylococcus epidermidis
46826	10592	10498	7670	7444	13571	12976	13913	13669	Bacteria	Firmicutes	Bacilli	Bacillales	Staphylococcaceae	Staphylococcus	Staphylococcus epidermidis
46886	9676	11576	8843	7289	13329	13082	13629	13635	Bacteria	Firmicutes	Bacilli	Bacillales	Staphylococcaceae	Staphylococcus	Staphylococcus epidermidis
46913	9017	10331	8103	7410	12760	12310	13032	12994	Bacteria	Firmicutes	Bacilli	Bacillales	Staphylococcaceae	Staphylococcus	Staphylococcus epidermidis
46998	9590	10704	7906	7274	13277	12445	13338	13093	Bacteria	Firmicutes	Bacilli	Bacillales	Staphylococcaceae	Staphylococcus	Staphylococcus epidermidis
47034	10167	10819	8154	7772	13402	12934	13803	13650	Bacteria	Firmicutes	Bacilli	Bacillales	Staphylococcaceae	Staphylococcus	Staphylococcus epidermidis
47109	10532	10793	7666	7422	13411	12830	13778	13559	Bacteria	Firmicutes	Bacilli	Bacillales	Staphylococcaceae	Staphylococcus	Staphylococcus epidermidis
47128	10671	11107	8198	7634	13802	13213	13974	13875	Bacteria	Firmicutes	Bacilli	Bacillales	Staphylococcaceae	Staphylococcus	Staphylococcus epidermidis
47130	10784	10666	7079	7525	13482	12852	13776	13599	Bacteria	Firmicutes	Bacilli	Bacillales	Staphylococcaceae	Staphylococcus	Staphylococcus epidermidis
47137	10378	11019	8730	8799	12960	12761	13114	13126	Bacteria	Firmicutes	Bacilli	Bacillales	Staphylococcaceae	Staphylococcus	Staphylococcus epidermidis
47173	8551	8607	7663	6925	11893	12479	12958	12758	Bacteria	Firmicutes	Bacilli	Bacillales	Staphylococcaceae	Staphylococcus	Staphylococcus epidermidis
47200	10461	10448	7672	7499	13311	12745	13714	13442	Bacteria	Firmicutes	Bacilli	Bacillales	Staphylococcaceae	Staphylococcus	Staphylococcus epidermidis
47276	10990	11145	10253	9146	12842	12842	13098	13058	Bacteria	Firmicutes	Bacilli	Bacillales	Staphylococcaceae	Staphylococcus	Staphylococcus epidermidis
47306	10734	10446	6959	7795	12659	12223	13338	13000	Bacteria	Firmicutes	Bacilli	Bacillales	Staphylococcaceae	Staphylococcus	Staphylococcus epidermidis
47320	10602	10431	6737	7608	13246	12874	13648	13493	Bacteria	Firmicutes	Bacilli	Bacillales	Staphylococcaceae	Staphylococcus	Staphylococcus epidermidis
47333	11035	11424	9452	9188	13930	13671	13856	13892	Bacteria	Firmicutes	Bacilli	Bacillales	Staphylococcaceae	Staphylococcus	Staphylococcus epidermidis
47341	11235	11260	10345	10016	13932	13557	13891	13872	Bacteria	Firmicutes	Bacilli	Bacillales	Staphylococcaceae	Staphylococcus	Staphylococcus epidermidis
47345	10865	10301	7173	7816	13025	12472	13675	13291	Bacteria	Firmicutes	Bacilli	Bacillales	Staphylococcaceae	Staphylococcus	Staphylococcus epidermidis
47361	10745	10444	6932	7584	13435	12795	13723	13506	Bacteria	Firmicutes	Bacilli	Bacillales	Staphylococcaceae	Staphylococcus	Staphylococcus epidermidis
47419	10213	11576	9523	7313	12036	12346	12676	12395	Bacteria	Firmicutes	Bacilli	Bacillales	Staphylococcaceae	Staphylococcus	Staphylococcus epidermidis
47425	10668	10444	6866	7927	13171	12711	13514	13407	Bacteria	Firmicutes	Bacilli	Bacillales	Staphylococcaceae	Staphylococcus	Staphylococcus epidermidis
47445	10252	10350	7639	7404	13311	12723	13698	13431	Bacteria	Firmicutes	Bacilli	Bacillales	Staphylococcaceae	Staphylococcus	Staphylococcus epidermidis
47462	10585	10047	6969	7543	13168	12525	13599	13353	Bacteria	Firmicutes	Bacilli	Bacillales	Staphylococcaceae	Staphylococcus	Staphylococcus epidermidis
47551	10791	10928	6945	7991	13480	12961	13795	13681	Bacteria	Firmicutes	Bacilli	Bacillales	Staphylococcaceae	Staphylococcus	Staphylococcus epidermidis
47552	10791	10842	7608	7447	13760	13163	14030	13860	Bacteria	Firmicutes	Bacilli	Bacillales	Staphylococcaceae	Staphylococcus	Staphylococcus epidermidis
47564	11022	10563	6852	7639	13536	12758	13731	13472	Bacteria	Firmicutes	Bacilli	Bacillales	Staphylococcaceae	Staphylococcus	Staphylococcus epidermidis
47616	9484	11452	8432	6799	12624	12618	13182	13128	Bacteria	Firmicutes	Bacilli	Bacillales	Staphylococcaceae	Staphylococcus	Staphylococcus epidermidis
47666	10571	10133	7260	7748	13121	12519	13674	13414	Bacteria	Firmicutes	Bacilli	Bacillales	Staphylococcaceae	Staphylococcus	Staphylococcus epidermidis
47672	11099	11160	8158	8476	13463	13157	13739	13645	Bacteria	Firmicutes	Bacilli	Bacillales	Staphylococcaceae	Staphylococcus	Staphylococcus epidermidis
47680	11021	10323	7174	7728	13487	12936	13812	13629	Bacteria	Firmicutes	Bacilli	Bacillales	Staphylococcaceae	Staphylococcus	Staphylococcus epidermidis
47715	10669	10825	7609	7503	13535	12962	13848	13664	Bacteria	Firmicutes	Bacilli	Bacillales	Staphylococcaceae	Staphylococcus	Staphylococcus epidermidis
47724	10107	11895	8765	6979	13641	13431	13960	13823	Bacteria	Firmicutes	Bacilli	Bacillales	Staphylococcaceae	Staphylococcus	Staphylococcus epidermidis
47756	10103	10769	8888	8550	13308	13155	13629	13463	Bacteria	Firmicutes	Bacilli	Bacillales	Staphylococcaceae	Staphylococcus	Staphylococcus epidermidis
47761	10474	9981	7642	8221	13261	12800	13691	13499	Bacteria	Firmicutes	Bacilli	Bacillales	Staphylococcaceae	Staphylococcus	Staphylococcus epidermidis
47792	9315	9165	6346	7029	12582	12280	13185	12853	Bacteria	Firmicutes	Bacilli	Bacillales	Staphylococcaceae	Staphylococcus	Staphylococcus epidermidis
47806	10274	12167	9059	7508	13380	13319	13635	13596	Bacteria	Firmicutes	Bacilli	Bacillales	Staphylococcaceae	Staphylococcus	Staphylococcus epidermidis
47819	11346	11462	10935	9905	13551	13307	13552	13829	Bacteria	Firmicutes	Bacilli	Bacillales	Staphylococcaceae	Staphylococcus	Staphylococcus epidermidis
47832	10704	10610	7507	7439	13507	12895	13879	13596	Bacteria	Firmicutes	Bacilli	Bacillales	Staphylococcaceae	Staphylococcus	Staphylococcus epidermidis
47869	10739	10717	7590	7351	13697	13073	13965	13766	Bacteria	Firmicutes	Bacilli	Bacillales	Staphylococcaceae	Staphylococcus	Staphylococcus epidermidis
47878	12246	12312	11653	10672	13511	13236	13718	13680	Bacteria	Firmicutes	Bacilli	Bacillales	Staphylococcaceae	Staphylococcus	Staphylococcus epidermidis
47993	10923	11528	10092	9752	13351	13555	13665	13752	Bacteria	Firmicutes	Bacilli	Bacillales	Staphylococcaceae	Staphylococcus	Staphylococcus epidermidis
48027	11112	10602	6849	7573	13762	12973	13915	13681	Bacteria	Firmicutes	Bacilli	Bacillales	Staphylococcaceae	Staphylococcus	Staphylococcus epidermidis
48075	10360	11389	10456	8942	12501	12919	13235	12811	Bacteria	Firmicutes	Bacilli	Bacillales	Staphylococcaceae	Staphylococcus	Staphylococcus epidermidis
48081	10966	10559	6816	7313	13438	12878	13841	13618	Bacteria	Firmicutes	Bacilli	Bacillales	Staphylococcaceae	Staphylococcus	Staphylococcus epidermidis
48083	10392	9901	7285	7586	13193	12618	13627	13385	Bacteria	Firmicutes	Bacilli	Bacillales	Staphylococcaceae	Staphylococcus	Staphylococcus epidermidis
48099	10025	10404	7953	7696	12586	12029	13229	12833	Bacteria	Firmicutes	Bacilli	Bacillales	Staphylococcaceae	Staphylococcus	Staphylococcus epidermidis
48122	10838	10411	6714	7238	13627	12888	13846	13596	Bacteria	Firmicutes	Bacilli	Bacillales	Staphylococcaceae	Staphylococcus	Staphylococcus epidermidis
48159	9735	9442	6309	6989	12761	12678	13465	13254	Bacteria	Firmicutes	Bacilli	Bacillales	Staphylococcaceae	Staphylococcus	Staphylococcus epidermidis
48186	10458	9827	6653	7139	13025	12265	13309	12921	Bacteria	Firmicutes	Bacilli	Bacillales	Staphylococcaceae	Staphylococcus	Staphylococcus epidermidis
45988	10325	9398	6713	8052	12645	12266	13369	13215	Bacteria	Firmicutes	Bacilli	Bacillales	Staphylococcaceae	Staphylococcus	Staphylococcus haemolyticus
46398	9690	9464	7828	8010	12820	12435	13126	12696	Bacteria	Firmicutes	Bacilli	Bacillales	Staphylococcaceae	Staphylococcus	Staphylococcus haemolyticus
46706	9476	9071	7095	7491	12526	12021	13157	12987	Bacteria	Firmicutes	Bacilli	Bacillales	Staphylococcaceae	Staphylococcus	Staphylococcus haemolyticus
47638	10316	10377	6972	7605	12773	12328	13318	13295	Bacteria	Firmicutes	Bacilli	Bacillales	Staphylococcaceae	Staphylococcus	Staphylococcus haemolyticus
47963	10436	10367	6905	7530	12981	12490	13439	13456	Bacteria	Firmicutes	Bacilli	Bacillales	Staphylococcaceae	Staphylococcus	Staphylococcus haemolyticus

Table S6 (part K): HybScores of OTUs that were called present in at least one of the samples. Taxonomic classifications are based on PhyloChip taxonomy (derived from GreenGenes). Please note, that data presented here was rank normalized before performing statisical analyses presented in the manuscript.

	Sample ID (PMA)				Sample ID				taxonomic classification of representative rOTU cluster						
OTU	UR_PMA	CO_PMA	CR8_PMA	CR5_PMA	UR	CO	CR8	CR5	kingdom	phylum	class	order	family	genus	species
48089	10324	9310	6625	7794	12640	12252	13370	13220	Bacteria	Firmicutes	Bacilli	Bacillales	Staphylococcaceae	Staphylococcus	Staphylococcus haemolyticus
46119	9215	8571	6068	5906	12656	11585	13485	13290	Bacteria	Firmicutes	Bacilli	Bacillales	Staphylococcaceae	Staphylococcus	Staphylococcus pettenkoferi
47000	10274	12028	9288	8388	11337	11548	12216	11644	Bacteria	Firmicutes	Bacilli	Bacillales	Staphylococcaceae	Staphylococcus	Staphylococcus sciuri
47738	9190	10589	7818	7773	11146	10982	11825	10872	Bacteria	Firmicutes	Bacilli	Bacillales	Staphylococcaceae	Staphylococcus	Staphylococcus sciuri
45900	10197	9962	7430	6962	13236	12783	13739	13495	Bacteria	Firmicutes	Bacilli	Bacillales	Staphylococcaceae	Staphylococcus	unclassified
45974	9351	10308	8256	7419	12575	12204	13143	12633	Bacteria	Firmicutes	Bacilli	Bacillales	Staphylococcaceae	Staphylococcus	unclassified
46013	8257	10195	6902	6489	13165	12741	13805	12929	Bacteria	Firmicutes	Bacilli	Bacillales	Staphylococcaceae	Staphylococcus	unclassified
46074	10422	9565	6705	7930	12935	12421	13404	13241	Bacteria	Firmicutes	Bacilli	Bacillales	Staphylococcaceae	Staphylococcus	unclassified
46081	10507	10445	7081	7441	13216	12469	13588	13329	Bacteria	Firmicutes	Bacilli	Bacillales	Staphylococcaceae	Staphylococcus	unclassified
46157	10259	11786	7584	8219	12838	12726	13334	13170	Bacteria	Firmicutes	Bacilli	Bacillales	Staphylococcaceae	Staphylococcus	unclassified
46167	10665	10315	6736	7209	13495	12835	13814	13627	Bacteria	Firmicutes	Bacilli	Bacillales	Staphylococcaceae	Staphylococcus	unclassified
46176	10449	10076	6886	7482	13280	12628	13662	13475	Bacteria	Firmicutes	Bacilli	Bacillales	Staphylococcaceae	Staphylococcus	unclassified
46190	10417	10785	6249	7083	13595	13167	13994	13862	Bacteria	Firmicutes	Bacilli	Bacillales	Staphylococcaceae	Staphylococcus	unclassified
46228	10293	10277	6908	7432	13022	12491	13553	13128	Bacteria	Firmicutes	Bacilli	Bacillales	Staphylococcaceae	Staphylococcus	unclassified
46259	9331	11253	7804	8179	11980	11886	12416	12316	Bacteria	Firmicutes	Bacilli	Bacillales	Staphylococcaceae	Staphylococcus	unclassified
46278	10312	10389	8717	8075	12841	12831	13353	13220	Bacteria	Firmicutes	Bacilli	Bacillales	Staphylococcaceae	Staphylococcus	unclassified
46329	11482	12001	11820	10894	13420	13382	13387	13443	Bacteria	Firmicutes	Bacilli	Bacillales	Staphylococcaceae	Staphylococcus	unclassified
46342	9955	9796	6675	6589	12767	12407	13341	12996	Bacteria	Firmicutes	Bacilli	Bacillales	Staphylococcaceae	Staphylococcus	unclassified
46360	10411	9499	6505	7584	12802	12352	13372	13221	Bacteria	Firmicutes	Bacilli	Bacillales	Staphylococcaceae	Staphylococcus	unclassified
46362	10051	8838	6676	7083	12717	12306	13104	12862	Bacteria	Firmicutes	Bacilli	Bacillales	Staphylococcaceae	Staphylococcus	unclassified
46367	10552	10282	7369	7347	13335	12719	13727	13491	Bacteria	Firmicutes	Bacilli	Bacillales	Staphylococcaceae	Staphylococcus	unclassified
46420	12231	12849	11812	11313	14355	14203	14283	14534	Bacteria	Firmicutes	Bacilli	Bacillales	Staphylococcaceae	Staphylococcus	unclassified
46463	10901	10591	7916	7831	13401	13109	13740	13579	Bacteria	Firmicutes	Bacilli	Bacillales	Staphylococcaceae	Staphylococcus	unclassified
46464	10749	10729	7141	8438	13480	12885	13817	13691	Bacteria	Firmicutes	Bacilli	Bacillales	Staphylococcaceae	Staphylococcus	unclassified
46484	9181	8771	8381	7774	11716	11975	12156	11957	Bacteria	Firmicutes	Bacilli	Bacillales	Staphylococcaceae	Staphylococcus	unclassified
46527	10807	11499	6656	7476	13787	13055	14153	14053	Bacteria	Firmicutes	Bacilli	Bacillales	Staphylococcaceae	Staphylococcus	unclassified
46532	10366	10110	6923	7179	13451	12895	13927	13722	Bacteria	Firmicutes	Bacilli	Bacillales	Staphylococcaceae	Staphylococcus	unclassified
46539	9955	9501	6936	6626	12653	12026	13134	12580	Bacteria	Firmicutes	Bacilli	Bacillales	Staphylococcaceae	Staphylococcus	unclassified
46548	8434	8051	6450	6795	11291	10608	11994	11770	Bacteria	Firmicutes	Bacilli	Bacillales	Staphylococcaceae	Staphylococcus	unclassified
46608	10898	10885	7150	7389	13531	12798	13833	13660	Bacteria	Firmicutes	Bacilli	Bacillales	Staphylococcaceae	Staphylococcus	unclassified
46621	10857	10382	7064	7567	13398	12807	13711	13518	Bacteria	Firmicutes	Bacilli	Bacillales	Staphylococcaceae	Staphylococcus	unclassified
46645	9616	10454	7293	6768	12884	12245	13176	12908	Bacteria	Firmicutes	Bacilli	Bacillales	Staphylococcaceae	Staphylococcus	unclassified
46661	9552	9850	8662	8254	12485	12371	13044	12840	Bacteria	Firmicutes	Bacilli	Bacillales	Staphylococcaceae	Staphylococcus	unclassified
46665	8612	8444	6794	7256	11462	11107	12175	11949	Bacteria	Firmicutes	Bacilli	Bacillales	Staphylococcaceae	Staphylococcus	unclassified
46668	8546	12323	6327	6753	11719	12503	12132	11896	Bacteria	Firmicutes	Bacilli	Bacillales	Staphylococcaceae	Staphylococcus	unclassified
46720	10113	9451	7044	7510	13418	12455	13713	13629	Bacteria	Firmicutes	Bacilli	Bacillales	Staphylococcaceae	Staphylococcus	unclassified
46733	10233	9766	8731	7620	13673	13405	14091	13839	Bacteria	Firmicutes	Bacilli	Bacillales	Staphylococcaceae	Staphylococcus	unclassified
46738	9210	8471	6446	7290	11959	11160	12751	12586	Bacteria	Firmicutes	Bacilli	Bacillales	Staphylococcaceae	Staphylococcus	unclassified
46766	10445	10683	6905	6983	13235	12562	13567	13542	Bacteria	Firmicutes	Bacilli	Bacillales	Staphylococcaceae	Staphylococcus	unclassified
46793	12167	12676	12314	11671	14313	13759	14154	14225	Bacteria	Firmicutes	Bacilli	Bacillales	Staphylococcaceae	Staphylococcus	unclassified
46809	10427	10435	8810	8846	13532	12884	13550	13230	Bacteria	Firmicutes	Bacilli	Bacillales	Staphylococcaceae	Staphylococcus	unclassified
46828	9405	9390	6311	7095	12327	11479	13033	12515	Bacteria	Firmicutes	Bacilli	Bacillales	Staphylococcaceae	Staphylococcus	unclassified
46891	10659	10519	6790	7211	13555	13252	13944	13753	Bacteria	Firmicutes	Bacilli	Bacillales	Staphylococcaceae	Staphylococcus	unclassified
46894	9362	9407	6626	6671	12426	11427	12714	12381	Bacteria	Firmicutes	Bacilli	Bacillales	Staphylococcaceae	Staphylococcus	unclassified
46905	10438	10832	7474	7397	13370	12601	13607	13522	Bacteria	Firmicutes	Bacilli	Bacillales	Staphylococcaceae	Staphylococcus	unclassified
46929	9378	9360	7108	7296	12394	11885	13040	12677	Bacteria	Firmicutes	Bacilli	Bacillales	Staphylococcaceae	Staphylococcus	unclassified
46947	10582	10190	6553	7316	13398	12746	13760	13560	Bacteria	Firmicutes	Bacilli	Bacillales	Staphylococcaceae	Staphylococcus	unclassified
46963	10519	10105	7203	7394	13238	12608	13597	13400	Bacteria	Firmicutes	Bacilli	Bacillales	Staphylococcaceae	Staphylococcus	unclassified
46981	10141	11418	8204	8295	13324	13048	13452	13494	Bacteria	Firmicutes	Bacilli	Bacillales	Staphylococcaceae	Staphylococcus	unclassified
47019	10353	10722	9685	9611	13452	12393	13380	12796	Bacteria	Firmicutes	Bacilli	Bacillales	Staphylococcaceae	Staphylococcus	unclassified
47101	8386	9627	5737	6655	11527	10446	12571	11467	Bacteria	Firmicutes	Bacilli	Bacillales	Staphylococcaceae	Staphylococcus	unclassified
47114	11301	11774	10342	9899	13074	12962	13416	13306	Bacteria	Firmicutes	Bacilli	Bacillales	Staphylococcaceae	Staphylococcus	unclassified
47129	10378	10182	6900	7201	13355	12689	13741	13529	Bacteria	Firmicutes	Bacilli	Bacillales	Staphylococcaceae	Staphylococcus	unclassified
47135	10036	10052	6817	7076	12717	11996	13419	13068	Bacteria	Firmicutes	Bacilli	Bacillales	Staphylococcaceae	Staphylococcus	unclassified
47152	10353	10392	6968	7089	13351	12680	13632	13477	Bacteria	Firmicutes	Bacilli	Bacillales	Staphylococcaceae	Staphylococcus	unclassified
47175	10552	10471	6525	7057	13373	12975	13812	13644	Bacteria	Firmicutes	Bacilli	Bacillales	Staphylococcaceae	Staphylococcus	unclassified
47185	10790	10350	7273	7556	13264	12604	13636	13431	Bacteria	Firmicutes	Bacilli	Bacillales	Staphylococcaceae	Staphylococcus	unclassified
47187	9893	9994	6979	7274	12371	12040	12921	12841	Bacteria	Firmicutes	Bacilli	Bacillales	Staphylococcaceae	Staphylococcus	unclassified
47188	11963	12288	10326	11186	12454	12306	13172	13025	Bacteria	Firmicutes	Bacilli	Bacillales	Staphylococcaceae	Staphylococcus	unclassified
47243	10456	11165	8177	7639	13603	13096	13936	13811	Bacteria	Firmicutes	Bacilli	Bacillales	Staphylococcaceae	Staphylococcus	unclassified
47246	9114	9712	6488	6955	12329	11907	12887	12945	Bacteria	Firmicutes	Bacilli	Bacillales	Staphylococcaceae	Staphylococcus	unclassified
47248	11253	10827	8624	8823	13567	13299	13839	13843	Bacteria	Firmicutes	Bacilli	Bacillales	Staphylococcaceae	Staphylococcus	unclassified
47296	10685	10311	7177	7594	13399	12781	13781	13575	Bacteria	Firmicutes	Bacilli	Bacillales	Staphylococcaceae	Staphylococcus	unclassified
47327	10112	10262	6577	6924	13036	12257	13494	13187	Bacteria	Firmicutes	Bacilli	Bacillales	Staphylococcaceae	Staphylococcus	unclassified
47338	10612	10689	7591	7403	13461	12858	13774	13555	Bacteria	Firmicutes	Bacilli	Bacillales	Staphylococcaceae	Staphylococcus	unclassified
47357	10475	9546	6751	7957	12865	12447	13439	13307	Bacteria	Firmicutes	Bacilli	Bacillales	Staphylococcaceae	Staphylococcus	unclassified
47476	9317	9411	7384	7441	12692	12405	13087	13066	Bacteria	Firmicutes	Bacilli	Bacillales	Staphylococcaceae	Staphylococcus	unclassified
47479	10507	10557	8656	7737	12798	12389	13483	13199	Bacteria	Firmicutes	Bacilli	Bacillales	Staphylococcaceae	Staphylococcus	unclassified
47519	11286	11820	10513	10336	13227	13275	13617	13736	Bacteria	Firmicutes	Bacilli	Bacillales	Staphylococcaceae	Staphylococcus	unclassified
47528	10464	11973	8974	8315	13267	12855	13627	13681	Bacteria	Firmicutes	Bacilli	Bacillales	Staphylococcaceae	Staphylococcus	unclassified
47544	10536	10793	6403	7046	13415	12647	13728	13584	Bacteria	Firmicutes	Bacilli	Bacillales	Staphylococcaceae	Staphylococcus	unclassified
47626	9432	8990	6235	6863	12291	11416	13252	12778	Bacteria	Firmicutes	Bacilli	Bacillales	Staphylococcaceae	Staphylococcus	unclassified
47663	10585	10343	7493	7473	13164	12535	13561	13328	Bacteria	Firmicutes	Bacilli	Bacillales	Staphylococcaceae	Staphylococcus	unclassified
47676	10494	9493	6632	7584	12964	12418	13422	13270	Bacteria	Firmicutes	Bacilli	Bacillales	Staphylococcaceae	Staphylococcus	unclassified
47728	10734	10295	7269	7412	13460	12835	13763	13586	Bacteria	Firmicutes	Bacilli	Bacillales	Staphylococcaceae	Staphylococcus	unclassified
47766	9859	10238	8023	7677	12860	12459	13143	12820	Bacteria	Firmicutes	Bacilli	Bacillales	Staphylococcaceae	Staphylococcus	unclassified
47821	10248	10469	7708	8276	13097	13097	13439	13666	Bacteria	Firmicutes	Bacilli	Bacillales	Staphylococcaceae	Staphylococcus	unclassified
47841	10072	9155	6601	7778	12421	12035	13178	13039	Bacteria	Firmicutes	Bacilli	Bacillales	Staphylococcaceae	Staphylococcus	unclassified
47862	10779	10672	7027	7356	13365	12596	13671	13480	Bacteria	Firmicutes	Bacilli	Bacillales	Staphylococcaceae	Staphylococcus	unclassified
47947	10598	10230	6994	7478	13342	12675	13725	13513	Bacteria	Firmicutes	Bacilli	Bacillales	Staphylococcaceae	Staphylococcus	unclassified
48039	10263	10136	7029	6843	12751	12003	13063	12720	Bacteria	Firmicutes	Bacilli	Bacillales	Staphylococcaceae	Staphylococcus	unclassified
48048	10776	10281	7368	7370	13429	12920	13806	13579	Bacteria	Firmicutes	Bacilli	Bacillales	Staphylococcaceae	Staphylococcus	unclassified
48138	9762	10416	8311	8110	12633	12210	13155	13138	Bacteria	Firmicutes	Bacilli	Bacillales	Staphylococcaceae	Staphylococcus	unclassified
48156	9706	9886	8542	7968	12878	12778	13239	13287	Bacteria	Firmicutes	Bacilli	Bacillales	Staphylococcaceae	Staphylococcus	unclassified
48200	10474	9433	6662	7875	12879	12424	13448	13347	Bacteria	Firmicutes	Bacilli	Bacillales	Staphylococcaceae	Staphylococcus	unclassified
43780	10846	11477	9669	9550	12732	12773	12992	13168	Bacteria	Firmicutes	Bacilli	Gemellales	Gemellaceae	Gemella	unclassified
42694	8092	7877	6842	6808	11100	11435	11196	13307	Bacteria	Firmicutes	Bacilli	Lactobacillales	Aerococcaceae	unclassified	unclassified
72531	11574	12364	11369	11402	12955	13050	12811	12997	Bacteria	Firmicutes	Bacilli	Lactobacillales	Carnobacteriaceae	Carnobacterium	Carnobacterium divergens
42706	9464	10002	8611	7749	11683	11561	11955	12522	Bacteria	Firmicutes	Bacilli	Lactobacillales	Carnobacteriaceae	unclassified	unclassified
42511	13757	14389	13968	12745	14498	14234	14209	14537	Bacteria	Firmicutes	Bacilli	Lactobacillales	Enterococcaceae	Enterococcus	unclassified
42616	9186	10226	7351	6607	12325	12553	12179	12046	Bacteria	Firmicutes	Bacilli	Lactobacillales	Enterococcaceae	Enterococcus	unclassified
42889	8583	10695	6810	7369	11841	12107	11260	11277	Bacteria	Firmicutes	Bacilli	Lactobacillales	Enterococcaceae	Enterococcus	unclassified
43507	8437	9553	7642	7656	11565	11668	11498	11208	Bacteria	Firmicutes	Bacilli	Lactobacillales	Enterococcaceae	Enterococcus	unclassified
43975	8858	9572	7228	7258	12305	12216	11781	11975	Bacteria	Firmicutes	Bacilli	Lactobacillales	Enterococcaceae	Enterococcus	unclassified
43982	8543	9460	6940	6748	12136	12315	11530	11624	Bacteria	Firmicutes	Bacilli	Lactobacillales	Enterococcaceae	Enterococcus	unclassified
44057	9447	9794	8877	8636	11681	12075	11286	11842	Bacteria	Firmicutes	Bacilli	Lactobacillales	Enterococcaceae	Enterococcus	unclassified
42743	8887	9243	8259	8116	11559	12203	11220	11435	Bacteria	Firmicutes	Bacilli	Lactobacillales	Enterococcaceae	Tetragenococcus	unclassified
71820	8279	10803	7553	7604	13212	12895	12587	12977	Bacteria	Firmicutes	Bacilli	Lactobacillales	Lactobacillaceae	Lactobacillus	Lactobacillus crispatus
72859	8289	7950	6985	6602	12055	11135	10511	10898	Bacteria	Firmicutes	Bacilli	Lactobacillales	Lactobacillaceae	Lactobacillus	Lactobacillus crispatus
72019	9267	7111	6238	7328	12021	10859	10570	10935	Bacteria	Firmicutes	Bacilli	Lactobacillales	Lactobacillaceae	Lactobacillus	Lactobacillus panis

Table S6 (part L): HybScores of OTUs that were called present in at least one of the samples. Taxonomic classifications are based on PhyloChip taxonomy (derived from GreenGenes). Please note, that data presented here was rank normalized before performing statisical analyses presented in the manuscript.

	Sample ID (PMA)				Sample ID				taxonomic classification of representative rOTU cluster						
OTU	UR_PMA	CO_PMA	CR8_PMA	CR5_PMA	UR	CO	CR8	CR5	kingdom	phylum	class	order	family	genus	species
71936	10710	10770	10561	7050	11780	10008	10273	10227	Bacteria	Firmicutes	Bacilli	Lactobacillales	Lactobacillaceae	Lactobacillus	unclassified
71954	8930	8539	7930	7793	12580	11047	10447	11179	Bacteria	Firmicutes	Bacilli	Lactobacillales	Lactobacillaceae	Lactobacillus	unclassified
71968	9146	8140	7826	7756	11613	10212	10049	10304	Bacteria	Firmicutes	Bacilli	Lactobacillales	Lactobacillaceae	Lactobacillus	unclassified
72006	9184	9332	7938	8937	11881	11571	11486	11728	Bacteria	Firmicutes	Bacilli	Lactobacillales	Lactobacillaceae	Lactobacillus	unclassified
72042	9314	7329	7131	7287	12984	11645	11385	11511	Bacteria	Firmicutes	Bacilli	Lactobacillales	Lactobacillaceae	Lactobacillus	unclassified
72132	10368	10721	9978	9146	12935	13153	12818	13585	Bacteria	Firmicutes	Bacilli	Lactobacillales	Lactobacillaceae	Lactobacillus	unclassified
72160	9512	7373	7236	7323	13004	11624	11460	11544	Bacteria	Firmicutes	Bacilli	Lactobacillales	Lactobacillaceae	Lactobacillus	unclassified
72197	8922	8337	7898	7324	12339	12256	12367	12573	Bacteria	Firmicutes	Bacilli	Lactobacillales	Lactobacillaceae	Lactobacillus	unclassified
72201	9615	9272	9062	8030	12611	12664	12494	13024	Bacteria	Firmicutes	Bacilli	Lactobacillales	Lactobacillaceae	Lactobacillus	unclassified
72285	8761	10763	8251	7673	12642	12878	12939	12961	Bacteria	Firmicutes	Bacilli	Lactobacillales	Lactobacillaceae	Lactobacillus	unclassified
72563	9653	10156	9218	8533	11786	11835	12545	12172	Bacteria	Firmicutes	Bacilli	Lactobacillales	Lactobacillaceae	Lactobacillus	unclassified
72597	9344	7413	7038	7324	13251	11999	11770	11794	Bacteria	Firmicutes	Bacilli	Lactobacillales	Lactobacillaceae	Lactobacillus	unclassified
72637	8233	7529	6131	6948	11513	11875	11639	11491	Bacteria	Firmicutes	Bacilli	Lactobacillales	Lactobacillaceae	Lactobacillus	unclassified
72729	8329	8910	7202	6934	10944	11188	12371	11224	Bacteria	Firmicutes	Bacilli	Lactobacillales	Lactobacillaceae	Lactobacillus	unclassified
72761	11480	11815	11311	10443	12568	12711	13031	12964	Bacteria	Firmicutes	Bacilli	Lactobacillales	Lactobacillaceae	Lactobacillus	unclassified
72839	11840	12270	11724	10483	13360	13416	13233	13939	Bacteria	Firmicutes	Bacilli	Lactobacillales	Lactobacillaceae	Lactobacillus	unclassified
72855	8919	6966	6520	7092	12698	11262	11050	11030	Bacteria	Firmicutes	Bacilli	Lactobacillales	Lactobacillaceae	Lactobacillus	unclassified
72884	8927	10092	7697	7720	11837	12140	12692	12476	Bacteria	Firmicutes	Bacilli	Lactobacillales	Lactobacillaceae	Lactobacillus	unclassified
72916	8351	8654	7291	6834	10922	11120	12320	11164	Bacteria	Firmicutes	Bacilli	Lactobacillales	Lactobacillaceae	Lactobacillus	unclassified
71863	9114	7973	7467	6832	12444	12449	12258	12199	Bacteria	Firmicutes	Bacilli	Lactobacillales	Leuconostocaceae	Leuconostoc	Leuconostoc citreum
71916	9223	8327	7191	6805	12431	12255	12106	11974	Bacteria	Firmicutes	Bacilli	Lactobacillales	Leuconostocaceae	Leuconostoc	Leuconostoc citreum
72147	9292	8486	7549	6959	12700	12578	12368	12410	Bacteria	Firmicutes	Bacilli	Lactobacillales	Leuconostocaceae	Leuconostoc	Leuconostoc citreum
72200	10092	10819	9761	9573	12634	12860	12776	12794	Bacteria	Firmicutes	Bacilli	Lactobacillales	Leuconostocaceae	Leuconostoc	Leuconostoc citreum
72453	8878	7763	7563	7001	12326	12197	12038	11943	Bacteria	Firmicutes	Bacilli	Lactobacillales	Leuconostocaceae	Leuconostoc	Leuconostoc citreum
72610	8877	8016	7551	6930	12361	12289	12113	12047	Bacteria	Firmicutes	Bacilli	Lactobacillales	Leuconostocaceae	Leuconostoc	Leuconostoc citreum
71907	8930	7984	7642	6939	12369	12173	12044	11996	Bacteria	Firmicutes	Bacilli	Lactobacillales	Leuconostocaceae	Leuconostoc	unclassified
72629	9406	8826	7369	7862	12524	12018	12301	12021	Bacteria	Firmicutes	Bacilli	Lactobacillales	Leuconostocaceae	Leuconostoc	unclassified
72646	8706	8096	7660	7044	12141	12289	12113	11903	Bacteria	Firmicutes	Bacilli	Lactobacillales	Leuconostocaceae	Leuconostoc	unclassified
72927	9613	8178	8165	8540	12020	12227	12177	12009	Bacteria	Firmicutes	Bacilli	Lactobacillales	Leuconostocaceae	Leuconostoc	unclassified
72987	9194	8399	7275	7645	12202	11637	11823	11616	Bacteria	Firmicutes	Bacilli	Lactobacillales	Leuconostocaceae	Leuconostoc	unclassified
42814	8280	7386	7045	7305	10980	12379	11050	11031	Bacteria	Firmicutes	Bacilli	Lactobacillales	Streptococcaceae	Lactococcus	Lactococcus lactis
42908	9194	7961	7570	7553	11323	12520	11595	11500	Bacteria	Firmicutes	Bacilli	Lactobacillales	Streptococcaceae	Lactococcus	Lactococcus lactis
43697	10887	10832	10732	10126	10745	12713	11680	11613	Bacteria	Firmicutes	Bacilli	Lactobacillales	Streptococcaceae	Lactococcus	Lactococcus lactis
45762	8067	7212	6735	7388	10579	11726	11657	11079	Bacteria	Firmicutes	Bacilli	Lactobacillales	Streptococcaceae	Streptococcus	Streptococcus cristatus
45888	8095	6878	6652	7446	12144	13013	12884	12561	Bacteria	Firmicutes	Bacilli	Lactobacillales	Streptococcaceae	Streptococcus	Streptococcus cristatus
44666	7502	6640	6248	6762	11443	12330	12240	11943	Bacteria	Firmicutes	Bacilli	Lactobacillales	Streptococcaceae	Streptococcus	Streptococcus infantis
44884	7981	6925	5446	6510	11544	12155	12207	12309	Bacteria	Firmicutes	Bacilli	Lactobacillales	Streptococcaceae	Streptococcus	Streptococcus infantis
44432	7751	6832	6121	6601	11455	12182	11952	11974	Bacteria	Firmicutes	Bacilli	Lactobacillales	Streptococcaceae	Streptococcus	Streptococcus oralis
44534	7779	7058	6187	6834	11682	12433	12180	12123	Bacteria	Firmicutes	Bacilli	Lactobacillales	Streptococcaceae	Streptococcus	Streptococcus oralis
44562	8218	7386	6655	6935	11806	12470	12361	12216	Bacteria	Firmicutes	Bacilli	Lactobacillales	Streptococcaceae	Streptococcus	Streptococcus oralis
44745	8358	7840	7771	6870	11919	12663	12845	12425	Bacteria	Firmicutes	Bacilli	Lactobacillales	Streptococcaceae	Streptococcus	Streptococcus oralis
44153	8332	7345	7013	7307	11986	12567	12406	12380	Bacteria	Firmicutes	Bacilli	Lactobacillales	Streptococcaceae	Streptococcus	Streptococcus pseudopneumoniae
44224	8434	7835	7481	7399	12112	12760	12629	12530	Bacteria	Firmicutes	Bacilli	Lactobacillales	Streptococcaceae	Streptococcus	Streptococcus pseudopneumoniae
44243	8219	7091	6976	7492	12115	12453	12258	12259	Bacteria	Firmicutes	Bacilli	Lactobacillales	Streptococcaceae	Streptococcus	Streptococcus pseudopneumoniae
44383	7701	6617	6499	7138	11586	12225	12038	11815	Bacteria	Firmicutes	Bacilli	Lactobacillales	Streptococcaceae	Streptococcus	Streptococcus pseudopneumoniae
44422	8071	6695	6469	6734	11631	12217	11993	11893	Bacteria	Firmicutes	Bacilli	Lactobacillales	Streptococcaceae	Streptococcus	Streptococcus pseudopneumoniae
44482	7912	6456	6609	6949	11611	12374	12255	11976	Bacteria	Firmicutes	Bacilli	Lactobacillales	Streptococcaceae	Streptococcus	Streptococcus pseudopneumoniae
44677	7748	7354	5822	6602	11600	12331	12102	12093	Bacteria	Firmicutes	Bacilli	Lactobacillales	Streptococcaceae	Streptococcus	Streptococcus pseudopneumoniae
44743	8093	7100	6985	6896	11805	12437	12271	12156	Bacteria	Firmicutes	Bacilli	Lactobacillales	Streptococcaceae	Streptococcus	Streptococcus pseudopneumoniae
44789	8009	7077	6977	6991	11996	12610	12401	12256	Bacteria	Firmicutes	Bacilli	Lactobacillales	Streptococcaceae	Streptococcus	Streptococcus pseudopneumoniae
44928	7897	6974	6812	7052	11491	12274	12100	11829	Bacteria	Firmicutes	Bacilli	Lactobacillales	Streptococcaceae	Streptococcus	Streptococcus pseudopneumoniae
44997	7932	6730	6644	7083	11690	12458	12316	12041	Bacteria	Firmicutes	Bacilli	Lactobacillales	Streptococcaceae	Streptococcus	Streptococcus pseudopneumoniae
45008	8192	7245	6877	7210	11922	12481	12265	12221	Bacteria	Firmicutes	Bacilli	Lactobacillales	Streptococcaceae	Streptococcus	Streptococcus pseudopneumoniae
45052	8310	7810	7405	7384	12272	12902	12751	12699	Bacteria	Firmicutes	Bacilli	Lactobacillales	Streptococcaceae	Streptococcus	Streptococcus pseudopneumoniae
45151	8657	8744	8259	7560	11828	12470	12210	12229	Bacteria	Firmicutes	Bacilli	Lactobacillales	Streptococcaceae	Streptococcus	Streptococcus pseudopneumoniae
45269	8360	7821	7439	7302	11985	12645	12516	12456	Bacteria	Firmicutes	Bacilli	Lactobacillales	Streptococcaceae	Streptococcus	Streptococcus pseudopneumoniae
45286	8059	7043	7005	6992	11957	12572	12435	12311	Bacteria	Firmicutes	Bacilli	Lactobacillales	Streptococcaceae	Streptococcus	Streptococcus pseudopneumoniae
45299	8450	7872	7469	7174	11838	12570	12420	12308	Bacteria	Firmicutes	Bacilli	Lactobacillales	Streptococcaceae	Streptococcus	Streptococcus pseudopneumoniae
45304	8214	6898	6936	7499	12107	12425	12246	12263	Bacteria	Firmicutes	Bacilli	Lactobacillales	Streptococcaceae	Streptococcus	Streptococcus pseudopneumoniae
45321	8220	6845	6536	7070	11836	12448	12472	12146	Bacteria	Firmicutes	Bacilli	Lactobacillales	Streptococcaceae	Streptococcus	Streptococcus pseudopneumoniae
45343	8421	7623	7206	7380	11932	12492	12320	12267	Bacteria	Firmicutes	Bacilli	Lactobacillales	Streptococcaceae	Streptococcus	Streptococcus pseudopneumoniae
45440	8095	7240	7007	6898	11634	12290	12116	12031	Bacteria	Firmicutes	Bacilli	Lactobacillales	Streptococcaceae	Streptococcus	Streptococcus pseudopneumoniae
45507	8883	9077	7665	7172	11458	12387	12351	12271	Bacteria	Firmicutes	Bacilli	Lactobacillales	Streptococcaceae	Streptococcus	Streptococcus pseudopneumoniae
45508	8409	7817	7465	7198	12047	12706	12569	12470	Bacteria	Firmicutes	Bacilli	Lactobacillales	Streptococcaceae	Streptococcus	Streptococcus pseudopneumoniae
45543	7660	6530	6534	6436	11835	12490	12371	12276	Bacteria	Firmicutes	Bacilli	Lactobacillales	Streptococcaceae	Streptococcus	Streptococcus pseudopneumoniae
45564	8129	7086	6968	7069	11071	11767	11404	11435	Bacteria	Firmicutes	Bacilli	Lactobacillales	Streptococcaceae	Streptococcus	Streptococcus pseudopneumoniae
45592	8382	7322	6998	7150	11891	12470	12318	12294	Bacteria	Firmicutes	Bacilli	Lactobacillales	Streptococcaceae	Streptococcus	Streptococcus pseudopneumoniae
45636	8054	6720	6691	6885	11657	12174	11958	11916	Bacteria	Firmicutes	Bacilli	Lactobacillales	Streptococcaceae	Streptococcus	Streptococcus pseudopneumoniae
45786	8179	7124	6846	7178	12370	12925	12827	12767	Bacteria	Firmicutes	Bacilli	Lactobacillales	Streptococcaceae	Streptococcus	Streptococcus pseudopneumoniae
45803	7727	6596	6439	7197	11846	12496	12343	12095	Bacteria	Firmicutes	Bacilli	Lactobacillales	Streptococcaceae	Streptococcus	Streptococcus pseudopneumoniae
45887	8203	6789	6685	7285	12059	12563	12402	12365	Bacteria	Firmicutes	Bacilli	Lactobacillales	Streptococcaceae	Streptococcus	Streptococcus pseudopneumoniae
44371	8640	8074	7251	6870	12325	12605	12596	12471	Bacteria	Firmicutes	Bacilli	Lactobacillales	Streptococcaceae	Streptococcus	Streptococcus sanguinis
45542	12747	13178	12716	11933	13447	13549	13482	13683	Bacteria	Firmicutes	Bacilli	Lactobacillales	Streptococcaceae	Streptococcus	Streptococcus sanguinis
45595	10490	10775	10411	9473	13056	13032	12824	13145	Bacteria	Firmicutes	Bacilli	Lactobacillales	Streptococcaceae	Streptococcus	Streptococcus sanguinis
44288	8410	7688	6392	6936	11505	12332	12547	12095	Bacteria	Firmicutes	Bacilli	Lactobacillales	Streptococcaceae	Streptococcus	Streptococcus thermophilus
44460	9090	10340	8570	8615	12561	12600	12592	12478	Bacteria	Firmicutes	Bacilli	Lactobacillales	Streptococcaceae	Streptococcus	Streptococcus thermophilus
44602	8383	7676	6344	6791	11239	12109	12314	11899	Bacteria	Firmicutes	Bacilli	Lactobacillales	Streptococcaceae	Streptococcus	Streptococcus thermophilus
44612	8196	6448	5569	6563	10836	11090	12918	11951	Bacteria	Firmicutes	Bacilli	Lactobacillales	Streptococcaceae	Streptococcus	Streptococcus thermophilus
44655	8483	7630	6704	6864	11276	12173	12436	11964	Bacteria	Firmicutes	Bacilli	Lactobacillales	Streptococcaceae	Streptococcus	Streptococcus thermophilus
45070	8448	7637	6361	6740	11187	12118	12307	11829	Bacteria	Firmicutes	Bacilli	Lactobacillales	Streptococcaceae	Streptococcus	Streptococcus thermophilus
45084	8437	7763	6343	6792	11491	12304	12529	12072	Bacteria	Firmicutes	Bacilli	Lactobacillales	Streptococcaceae	Streptococcus	Streptococcus thermophilus
45101	8502	7823	6654	7238	11197	12087	12310	11790	Bacteria	Firmicutes	Bacilli	Lactobacillales	Streptococcaceae	Streptococcus	Streptococcus thermophilus
45225	8482	7684	6435	6859	11359	12181	12400	11928	Bacteria	Firmicutes	Bacilli	Lactobacillales	Streptococcaceae	Streptococcus	Streptococcus thermophilus
45307	8466	7683	6332	6729	11260	12043	12262	11813	Bacteria	Firmicutes	Bacilli	Lactobacillales	Streptococcaceae	Streptococcus	Streptococcus thermophilus
45382	8512	7619	6395	6848	11275	12089	12320	11875	Bacteria	Firmicutes	Bacilli	Lactobacillales	Streptococcaceae	Streptococcus	Streptococcus thermophilus
45593	8968	9233	8626	7441	11975	12258	12493	12060	Bacteria	Firmicutes	Bacilli	Lactobacillales	Streptococcaceae	Streptococcus	Streptococcus thermophilus
44160	7745	6611	6225	6997	11885	12502	12348	12269	Bacteria	Firmicutes	Bacilli	Lactobacillales	Streptococcaceae	Streptococcus	unclassified
44162	8073	9332	6894	7108	12131	12731	12676	12580	Bacteria	Firmicutes	Bacilli	Lactobacillales	Streptococcaceae	Streptococcus	unclassified
44165	8330	7168	6328	6754	11532	12366	12272	12013	Bacteria	Firmicutes	Bacilli	Lactobacillales	Streptococcaceae	Streptococcus	unclassified
44167	7972	7961	6586	6830	11249	12047	11789	11881	Bacteria	Firmicutes	Bacilli	Lactobacillales	Streptococcaceae	Streptococcus	unclassified
44182	8251	7553	6424	6983	11246	12198	12104	11798	Bacteria	Firmicutes	Bacilli	Lactobacillales	Streptococcaceae	Streptococcus	unclassified
44212	8301	7349	6913	7352	12457	12939	12828	12770	Bacteria	Firmicutes	Bacilli	Lactobacillales	Streptococcaceae	Streptococcus	unclassified
44221	8655	9784	8446	7743	11762	12599	12463	12409	Bacteria	Firmicutes	Bacilli	Lactobacillales	Streptococcaceae	Streptococcus	unclassified
44257	8006	7123	6328	6460	11423	12301	12174	12212	Bacteria	Firmicutes	Bacilli	Lactobacillales	Streptococcaceae	Streptococcus	unclassified
44277	7823	7984	6063	6596	12110	12482	12535	12368	Bacteria	Firmicutes	Bacilli	Lactobacillales	Streptococcaceae	Streptococcus	unclassified
44280	8502	7922	7117	6940	11077	12173	11961	11651	Bacteria	Firmicutes	Bacilli	Lactobacillales	Streptococcaceae	Streptococcus	unclassified
44299	7940	7378	6192	6894	12226	12763	12579	12630	Bacteria	Firmicutes	Bacilli	Lactobacillales	Streptococcaceae	Streptococcus	unclassified
44308	8020	7091	6457	6456	11305	12099	11970	12041	Bacteria	Firmicutes	Bacilli	Lactobacillales	Streptococcaceae	Streptococcus	unclassified
44316	8196	7229	6591	6808	11256	12298	12235	11911	Bacteria	Firmicutes	Bacilli	Lactobacillales	Streptococcaceae	Streptococcus	unclassified
44346	8166	7644	6204	6509	11660	12628	12443	12334	Bacteria	Firmicutes	Bacilli	Lactobacillales	Streptococcaceae	Streptococcus	unclassified

Table S6 (part M): HybScores of OTUs that were called present in at least one of the samples. Taxonomic classifications are based on PhyloChip taxonomy (derived from GreenGenes). Please note, that data presented here was rank normalized before performing statisical analyses presented in the manuscript.

	Sample ID (PMA)				Sample ID				taxonomic classification of representative rOTU cluster						
OTU	UR_PMA	CO_PMA	CR8_PMA	CR5_PMA	UR	CO	CR8	CR5	kingdom	phylum	class	order	family	genus	species
44363	8279	7645	6482	6722	11281	12314	12158	12153	Bacteria	Firmicutes	Bacilli	Lactobacillales	Streptococcaceae	Streptococcus	unclassified
44380	8386	8674	7032	6692	12471	12882	12783	12640	Bacteria	Firmicutes	Bacilli	Lactobacillales	Streptococcaceae	Streptococcus	unclassified
44396	8286	7841	6573	7163	10648	11892	11768	11422	Bacteria	Firmicutes	Bacilli	Lactobacillales	Streptococcaceae	Streptococcus	unclassified
44452	7997	6523	6343	7012	11678	12258	12218	11930	Bacteria	Firmicutes	Bacilli	Lactobacillales	Streptococcaceae	Streptococcus	unclassified
44466	7746	6540	6222	6418	11204	11773	12233	11804	Bacteria	Firmicutes	Bacilli	Lactobacillales	Streptococcaceae	Streptococcus	unclassified
44501	8005	6793	6762	7007	11406	12226	12107	11829	Bacteria	Firmicutes	Bacilli	Lactobacillales	Streptococcaceae	Streptococcus	unclassified
44511	8728	8603	8164	7292	11437	12517	12391	12157	Bacteria	Firmicutes	Bacilli	Lactobacillales	Streptococcaceae	Streptococcus	unclassified
44516	8261	6851	6479	6729	11147	12020	11937	11667	Bacteria	Firmicutes	Bacilli	Lactobacillales	Streptococcaceae	Streptococcus	unclassified
44533	8445	7477	7032	6854	10985	12046	11947	11585	Bacteria	Firmicutes	Bacilli	Lactobacillales	Streptococcaceae	Streptococcus	unclassified
44552	8447	7658	6805	6873	11255	12284	12151	11850	Bacteria	Firmicutes	Bacilli	Lactobacillales	Streptococcaceae	Streptococcus	unclassified
44553	8515	7603	6803	7261	11081	11846	11833	11463	Bacteria	Firmicutes	Bacilli	Lactobacillales	Streptococcaceae	Streptococcus	unclassified
44554	8013	7118	6991	7129	11268	12245	12083	11852	Bacteria	Firmicutes	Bacilli	Lactobacillales	Streptococcaceae	Streptococcus	unclassified
44565	8402	7787	7427	7280	11923	12629	12494	12397	Bacteria	Firmicutes	Bacilli	Lactobacillales	Streptococcaceae	Streptococcus	unclassified
44574	8036	6807	6833	6938	11281	12023	11738	11729	Bacteria	Firmicutes	Bacilli	Lactobacillales	Streptococcaceae	Streptococcus	unclassified
44590	8527	8184	7092	7270	11342	12363	12153	11930	Bacteria	Firmicutes	Bacilli	Lactobacillales	Streptococcaceae	Streptococcus	unclassified
44604	8000	6934	5666	6530	11567	12248	12367	12138	Bacteria	Firmicutes	Bacilli	Lactobacillales	Streptococcaceae	Streptococcus	unclassified
44608	8326	7423	6886	6821	11125	12141	11998	11752	Bacteria	Firmicutes	Bacilli	Lactobacillales	Streptococcaceae	Streptococcus	unclassified
44623	7804	6979	5866	6677	11560	12191	11846	11899	Bacteria	Firmicutes	Bacilli	Lactobacillales	Streptococcaceae	Streptococcus	unclassified
44633	8176	7142	6840	6712	11040	12070	12093	11745	Bacteria	Firmicutes	Bacilli	Lactobacillales	Streptococcaceae	Streptococcus	unclassified
44644	9314	8546	7153	7559	11399	11997	11475	11682	Bacteria	Firmicutes	Bacilli	Lactobacillales	Streptococcaceae	Streptococcus	unclassified
44647	8262	7697	7057	7242	11693	12387	12313	12130	Bacteria	Firmicutes	Bacilli	Lactobacillales	Streptococcaceae	Streptococcus	unclassified
44651	7891	7148	6480	6722	10830	11832	11605	11660	Bacteria	Firmicutes	Bacilli	Lactobacillales	Streptococcaceae	Streptococcus	unclassified
44673	8056	6794	6819	7019	11794	12244	12043	11954	Bacteria	Firmicutes	Bacilli	Lactobacillales	Streptococcaceae	Streptococcus	unclassified
44680	9699	9416	9522	9557	11694	12332	12296	12228	Bacteria	Firmicutes	Bacilli	Lactobacillales	Streptococcaceae	Streptococcus	unclassified
44681	7745	6536	5892	6619	11747	12256	12195	12301	Bacteria	Firmicutes	Bacilli	Lactobacillales	Streptococcaceae	Streptococcus	unclassified
44688	9566	9068	8426	7896	11652	11996	11709	11901	Bacteria	Firmicutes	Bacilli	Lactobacillales	Streptococcaceae	Streptococcus	unclassified
44703	8325	8483	7235	7263	11947	12516	12399	12216	Bacteria	Firmicutes	Bacilli	Lactobacillales	Streptococcaceae	Streptococcus	unclassified
44721	9961	10361	8819	8438	12643	12890	12804	13272	Bacteria	Firmicutes	Bacilli	Lactobacillales	Streptococcaceae	Streptococcus	unclassified
44751	8413	7925	7345	7374	11748	12451	12329	12203	Bacteria	Firmicutes	Bacilli	Lactobacillales	Streptococcaceae	Streptococcus	unclassified
44757	8258	7795	6542	6504	11397	12376	11759	11487	Bacteria	Firmicutes	Bacilli	Lactobacillales	Streptococcaceae	Streptococcus	unclassified
44767	8421	7559	6829	6989	11071	12145	12022	11666	Bacteria	Firmicutes	Bacilli	Lactobacillales	Streptococcaceae	Streptococcus	unclassified
44770	8007	7207	6949	6776	11545	12280	12078	11953	Bacteria	Firmicutes	Bacilli	Lactobacillales	Streptococcaceae	Streptococcus	unclassified
44778	7709	6698	6045	6619	11491	12161	12017	12083	Bacteria	Firmicutes	Bacilli	Lactobacillales	Streptococcaceae	Streptococcus	unclassified
44792	9277	7943	8792	7918	11875	12652	12560	12450	Bacteria	Firmicutes	Bacilli	Lactobacillales	Streptococcaceae	Streptococcus	unclassified
44813	8112	8836	7086	6941	11865	11649	12123	12376	Bacteria	Firmicutes	Bacilli	Lactobacillales	Streptococcaceae	Streptococcus	unclassified
44816	7855	6470	6526	6669	12025	12506	12384	12337	Bacteria	Firmicutes	Bacilli	Lactobacillales	Streptococcaceae	Streptococcus	unclassified
44820	7770	6926	5912	6678	11669	12182	11825	11912	Bacteria	Firmicutes	Bacilli	Lactobacillales	Streptococcaceae	Streptococcus	unclassified
44823	8485	7605	7215	7539	11937	12546	12384	12273	Bacteria	Firmicutes	Bacilli	Lactobacillales	Streptococcaceae	Streptococcus	unclassified
44826	8093	7212	6232	6519	11157	12119	11965	11970	Bacteria	Firmicutes	Bacilli	Lactobacillales	Streptococcaceae	Streptococcus	unclassified
44829	7960	7291	6647	6812	10968	11921	11715	11745	Bacteria	Firmicutes	Bacilli	Lactobacillales	Streptococcaceae	Streptococcus	unclassified
44837	8340	7333	6633	6921	11186	12213	12122	11798	Bacteria	Firmicutes	Bacilli	Lactobacillales	Streptococcaceae	Streptococcus	unclassified
44852	8681	9195	7251	6967	11532	12207	12245	12480	Bacteria	Firmicutes	Bacilli	Lactobacillales	Streptococcaceae	Streptococcus	unclassified
44860	8408	7832	7474	7402	11965	12672	12470	12392	Bacteria	Firmicutes	Bacilli	Lactobacillales	Streptococcaceae	Streptococcus	unclassified
44896	7803	6841	6374	7234	12163	12817	12672	12636	Bacteria	Firmicutes	Bacilli	Lactobacillales	Streptococcaceae	Streptococcus	unclassified
44904	8488	9541	7089	7002	11490	11838	11976	11728	Bacteria	Firmicutes	Bacilli	Lactobacillales	Streptococcaceae	Streptococcus	unclassified
44910	8020	6952	6226	6437	11504	12287	12208	12302	Bacteria	Firmicutes	Bacilli	Lactobacillales	Streptococcaceae	Streptococcus	unclassified
44911	8198	7630	7053	7119	11733	12319	12263	12080	Bacteria	Firmicutes	Bacilli	Lactobacillales	Streptococcaceae	Streptococcus	unclassified
44912	8514	7940	7294	7095	10880	12006	11889	11515	Bacteria	Firmicutes	Bacilli	Lactobacillales	Streptococcaceae	Streptococcus	unclassified
44957	7999	6747	6692	6817	11554	12331	12348	12102	Bacteria	Firmicutes	Bacilli	Lactobacillales	Streptococcaceae	Streptococcus	unclassified
44958	8011	6664	6752	7105	11407	12234	12043	11786	Bacteria	Firmicutes	Bacilli	Lactobacillales	Streptococcaceae	Streptococcus	unclassified
44969	8607	8179	7124	7338	11506	12489	12301	12022	Bacteria	Firmicutes	Bacilli	Lactobacillales	Streptococcaceae	Streptococcus	unclassified
44976	9167	8233	7452	7848	12207	11429	11288	11614	Bacteria	Firmicutes	Bacilli	Lactobacillales	Streptococcaceae	Streptococcus	unclassified
45005	8105	7341	7245	7038	11501	12482	12457	12244	Bacteria	Firmicutes	Bacilli	Lactobacillales	Streptococcaceae	Streptococcus	unclassified
45015	8427	7778	7225	7212	11153	11970	11842	11825	Bacteria	Firmicutes	Bacilli	Lactobacillales	Streptococcaceae	Streptococcus	unclassified
45016	7945	6671	6618	6760	11571	12162	11916	11837	Bacteria	Firmicutes	Bacilli	Lactobacillales	Streptococcaceae	Streptococcus	unclassified
45053	7637	6519	6534	7143	11486	12157	11993	11740	Bacteria	Firmicutes	Bacilli	Lactobacillales	Streptococcaceae	Streptococcus	unclassified
45097	8207	7464	6642	6639	11029	11227	11617	11306	Bacteria	Firmicutes	Bacilli	Lactobacillales	Streptococcaceae	Streptococcus	unclassified
45105	8151	7243	6564	6704	11196	12111	12037	11727	Bacteria	Firmicutes	Bacilli	Lactobacillales	Streptococcaceae	Streptococcus	unclassified
45111	7813	7068	6490	6882	11208	12108	11880	11740	Bacteria	Firmicutes	Bacilli	Lactobacillales	Streptococcaceae	Streptococcus	unclassified
45118	7947	7119	6415	6787	11502	12389	12232	12280	Bacteria	Firmicutes	Bacilli	Lactobacillales	Streptococcaceae	Streptococcus	unclassified
45130	7860	6689	6519	6488	11941	12622	12484	12468	Bacteria	Firmicutes	Bacilli	Lactobacillales	Streptococcaceae	Streptococcus	unclassified
45131	7950	6756	6606	7028	11663	12324	12214	12061	Bacteria	Firmicutes	Bacilli	Lactobacillales	Streptococcaceae	Streptococcus	unclassified
45158	8401	7917	6500	6629	11499	12605	12423	12366	Bacteria	Firmicutes	Bacilli	Lactobacillales	Streptococcaceae	Streptococcus	unclassified
45161	7624	6229	5939	6710	12375	13211	13102	12849	Bacteria	Firmicutes	Bacilli	Lactobacillales	Streptococcaceae	Streptococcus	unclassified
45167	8257	7127	6683	7078	11395	12318	12254	11975	Bacteria	Firmicutes	Bacilli	Lactobacillales	Streptococcaceae	Streptococcus	unclassified
45179	7928	6298	6369	6469	11165	12054	12247	11722	Bacteria	Firmicutes	Bacilli	Lactobacillales	Streptococcaceae	Streptococcus	unclassified
45188	8518	8602	7637	7502	12268	12734	12691	12749	Bacteria	Firmicutes	Bacilli	Lactobacillales	Streptococcaceae	Streptococcus	unclassified
45210	7693	6681	5998	6781	12053	12518	12447	12514	Bacteria	Firmicutes	Bacilli	Lactobacillales	Streptococcaceae	Streptococcus	unclassified
45221	7833	6787	5718	6535	11408	12086	11687	11790	Bacteria	Firmicutes	Bacilli	Lactobacillales	Streptococcaceae	Streptococcus	unclassified
45228	8222	7572	6851	6856	11184	12223	12089	11798	Bacteria	Firmicutes	Bacilli	Lactobacillales	Streptococcaceae	Streptococcus	unclassified
45230	7811	6968	6210	6832	11800	12363	12198	12290	Bacteria	Firmicutes	Bacilli	Lactobacillales	Streptococcaceae	Streptococcus	unclassified
45238	7779	6702	5997	6245	11406	12054	11927	12015	Bacteria	Firmicutes	Bacilli	Lactobacillales	Streptococcaceae	Streptococcus	unclassified
45246	8802	8822	8582	7315	10748	11810	11641	11437	Bacteria	Firmicutes	Bacilli	Lactobacillales	Streptococcaceae	Streptococcus	unclassified
45297	8889	8787	8709	7340	12096	12126	12765	12070	Bacteria	Firmicutes	Bacilli	Lactobacillales	Streptococcaceae	Streptococcus	unclassified
45300	8162	7533	6267	6567	11498	12440	12195	12198	Bacteria	Firmicutes	Bacilli	Lactobacillales	Streptococcaceae	Streptococcus	unclassified
45308	8216	7582	6424	6952	11280	12213	12144	11754	Bacteria	Firmicutes	Bacilli	Lactobacillales	Streptococcaceae	Streptococcus	unclassified
45311	8078	6702	6371	7325	11846	12826	12596	12256	Bacteria	Firmicutes	Bacilli	Lactobacillales	Streptococcaceae	Streptococcus	unclassified
45320	8373	7806	7282	7342	12106	12771	12654	12542	Bacteria	Firmicutes	Bacilli	Lactobacillales	Streptococcaceae	Streptococcus	unclassified
45324	7711	6679	6082	6754	12193	12648	12474	12561	Bacteria	Firmicutes	Bacilli	Lactobacillales	Streptococcaceae	Streptococcus	unclassified
45326	8005	7091	6939	6944	11360	12284	12178	11931	Bacteria	Firmicutes	Bacilli	Lactobacillales	Streptococcaceae	Streptococcus	unclassified
45350	8275	7197	6956	7166	11331	12091	11775	11699	Bacteria	Firmicutes	Bacilli	Lactobacillales	Streptococcaceae	Streptococcus	unclassified
45352	7906	7056	6480	6882	11399	12200	12079	12018	Bacteria	Firmicutes	Bacilli	Lactobacillales	Streptococcaceae	Streptococcus	unclassified
45357	9562	9847	9201	8319	12036	12776	12614	12550	Bacteria	Firmicutes	Bacilli	Lactobacillales	Streptococcaceae	Streptococcus	unclassified
45385	9532	10901	7566	6904	12125	12428	12004	12376	Bacteria	Firmicutes	Bacilli	Lactobacillales	Streptococcaceae	Streptococcus	unclassified
45389	8156	7366	7229	7156	11354	12138	11967	11870	Bacteria	Firmicutes	Bacilli	Lactobacillales	Streptococcaceae	Streptococcus	unclassified
45402	8209	7165	6303	6512	11586	11891	11974	11865	Bacteria	Firmicutes	Bacilli	Lactobacillales	Streptococcaceae	Streptococcus	unclassified
45404	8143	7144	6944	7131	11399	12068	11771	11762	Bacteria	Firmicutes	Bacilli	Lactobacillales	Streptococcaceae	Streptococcus	unclassified
45405	7998	7204	7219	6963	11595	12229	11996	12060	Bacteria	Firmicutes	Bacilli	Lactobacillales	Streptococcaceae	Streptococcus	unclassified
45427	8386	7994	6963	7224	11401	12385	12257	11966	Bacteria	Firmicutes	Bacilli	Lactobacillales	Streptococcaceae	Streptococcus	unclassified
45438	8377	7801	7323	7403	12473	13086	12978	12892	Bacteria	Firmicutes	Bacilli	Lactobacillales	Streptococcaceae	Streptococcus	unclassified
45474	9660	10148	9613	7349	12947	13273	13111	13090	Bacteria	Firmicutes	Bacilli	Lactobacillales	Streptococcaceae	Streptococcus	unclassified
45481	7926	6607	6608	6691	11817	12407	12205	12110	Bacteria	Firmicutes	Bacilli	Lactobacillales	Streptococcaceae	Streptococcus	unclassified
45483	8065	7139	6516	6837	11081	11885	11488	11624	Bacteria	Firmicutes	Bacilli	Lactobacillales	Streptococcaceae	Streptococcus	unclassified
45484	11151	11446	11014	7425	12987	13303	13316	13495	Bacteria	Firmicutes	Bacilli	Lactobacillales	Streptococcaceae	Streptococcus	unclassified
45493	7885	6426	6711	7016	11579	12348	12197	11962	Bacteria	Firmicutes	Bacilli	Lactobacillales	Streptococcaceae	Streptococcus	unclassified
45500	8467	7976	7001	7215	10917	12119	11895	11594	Bacteria	Firmicutes	Bacilli	Lactobacillales	Streptococcaceae	Streptococcus	unclassified
45513	10971	10753	10692	9795	12557	13096	12657	12814	Bacteria	Firmicutes	Bacilli	Lactobacillales	Streptococcaceae	Streptococcus	unclassified
45523	8879	9090	8449	7953	12350	12855	12747	12683	Bacteria	Firmicutes	Bacilli	Lactobacillales	Streptococcaceae	Streptococcus	unclassified
45529	7936	7677	6493	6775	11594	12392	12110	12221	Bacteria	Firmicutes	Bacilli	Lactobacillales	Streptococcaceae	Streptococcus	unclassified
45531	8544	7797	6595	7720	11897	12753	12395	12549	Bacteria	Firmicutes	Bacilli	Lactobacillales	Streptococcaceae	Streptococcus	unclassified

Table S6 (part N): HybScores of OTUs that were called present in at least one of the samples. Taxonomic classifications are based on PhyloChip taxonomy (derived from GreenGenes). Please note, that data presented here was rank normalized before performing statisical analyses presented in the manuscript.

	Sample ID (PMA)				Sample ID				taxonomic classification of representative rOTU cluster						
OTU	UR_PMA	CO_PMA	CR8_PMA	CR5_PMA	UR	CO	CR8	CR5	kingdom	phylum	class	order	family	genus	species
45536	7847	6910	6690	6901	11543	12074	11915	11840	Bacteria	Firmicutes	Bacilli	Lactobacillales	Streptococcaceae	Streptococcus	unclassified
45549	8629	8109	7560	7021	11516	12360	12299	12006	Bacteria	Firmicutes	Bacilli	Lactobacillales	Streptococcaceae	Streptococcus	unclassified
45556	8156	7366	7229	7156	11354	12138	11967	11870	Bacteria	Firmicutes	Bacilli	Lactobacillales	Streptococcaceae	Streptococcus	unclassified
45563	7775	7007	6342	6934	11647	12413	12296	12258	Bacteria	Firmicutes	Bacilli	Lactobacillales	Streptococcaceae	Streptococcus	unclassified
45578	8939	8856	7971	7786	11748	12347	12306	12081	Bacteria	Firmicutes	Bacilli	Lactobacillales	Streptococcaceae	Streptococcus	unclassified
45587	8374	7751	7434	7373	12054	12739	12619	12528	Bacteria	Firmicutes	Bacilli	Lactobacillales	Streptococcaceae	Streptococcus	unclassified
45601	8622	8586	8101	7160	11394	12312	12198	11966	Bacteria	Firmicutes	Bacilli	Lactobacillales	Streptococcaceae	Streptococcus	unclassified
45626	8060	7283	6748	7149	11758	12348	12238	12067	Bacteria	Firmicutes	Bacilli	Lactobacillales	Streptococcaceae	Streptococcus	unclassified
45628	8079	7431	7018	7182	11428	12270	12133	12060	Bacteria	Firmicutes	Bacilli	Lactobacillales	Streptococcaceae	Streptococcus	unclassified
45679	8111	7223	6832	6842	11605	12330	12147	12008	Bacteria	Firmicutes	Bacilli	Lactobacillales	Streptococcaceae	Streptococcus	unclassified
45682	8124	7451	6452	7005	11912	12592	12399	12376	Bacteria	Firmicutes	Bacilli	Lactobacillales	Streptococcaceae	Streptococcus	unclassified
45694	8226	7891	6848	7484	11831	12331	12188	12153	Bacteria	Firmicutes	Bacilli	Lactobacillales	Streptococcaceae	Streptococcus	unclassified
45695	8145	7189	6991	6866	11509	12271	12086	11951	Bacteria	Firmicutes	Bacilli	Lactobacillales	Streptococcaceae	Streptococcus	unclassified
45720	8110	7262	6969	7005	11888	12515	12340	12217	Bacteria	Firmicutes	Bacilli	Lactobacillales	Streptococcaceae	Streptococcus	unclassified
45725	8164	7365	7240	6960	11469	12137	12147	12020	Bacteria	Firmicutes	Bacilli	Lactobacillales	Streptococcaceae	Streptococcus	unclassified
45744	8101	7172	7049	7033	11845	12485	12243	12149	Bacteria	Firmicutes	Bacilli	Lactobacillales	Streptococcaceae	Streptococcus	unclassified
45828	8152	7071	6762	6781	11100	12101	12076	11738	Bacteria	Firmicutes	Bacilli	Lactobacillales	Streptococcaceae	Streptococcus	unclassified
45837	8613	8268	7228	7182	11084	12155	12048	11714	Bacteria	Firmicutes	Bacilli	Lactobacillales	Streptococcaceae	Streptococcus	unclassified
45844	7937	6963	5888	6706	11814	12500	12295	12235	Bacteria	Firmicutes	Bacilli	Lactobacillales	Streptococcaceae	Streptococcus	unclassified
45855	8439	8379	6858	7025	11481	12408	12436	12077	Bacteria	Firmicutes	Bacilli	Lactobacillales	Streptococcaceae	Streptococcus	unclassified
45870	7701	6523	5870	6641	11486	12151	11744	11856	Bacteria	Firmicutes	Bacilli	Lactobacillales	Streptococcaceae	Streptococcus	unclassified
45878	7774	6749	6497	6708	12008	12671	12517	12446	Bacteria	Firmicutes	Bacilli	Lactobacillales	Streptococcaceae	Streptococcus	unclassified
45879	8817	8842	7484	7534	11963	12304	11955	11974	Bacteria	Firmicutes	Bacilli	Lactobacillales	Streptococcaceae	Streptococcus	unclassified
45896	8495	9163	7321	6638	12025	12335	12596	12244	Bacteria	Firmicutes	Bacilli	Lactobacillales	Streptococcaceae	Streptococcus	unclassified
49397	9551	10215	8774	7633	11958	12511	12240	12999	Bacteria	Firmicutes	Bacilli	Thermicanales	Thermicanaceae	Thermicanus	unclassified
72587	10532	11225	10700	9786	11885	12305	12311	11600	Bacteria	Firmicutes	Clostridia	Clostridiales	Clostridiaceae	Clostridium	Clostridium perfringens
27490	8763	9496	8070	7390	12154	12837	12561	12498	Bacteria	Firmicutes	Clostridia	Clostridiales	Clostridiaceae	Clostridium	unclassified
44235	10278	10754	9495	7920	11926	12152	11405	11834	Bacteria	Firmicutes	Clostridia	Clostridiales	Clostridiaceae	Clostridium	unclassified
44702	9610	10525	6881	7022	11542	11721	10693	11657	Bacteria	Firmicutes	Clostridia	Clostridiales	Clostridiaceae	Clostridium	unclassified
44744	10121	10568	9358	8626	11569	11926	10811	11634	Bacteria	Firmicutes	Clostridia	Clostridiales	Clostridiaceae	Clostridium	unclassified
45487	11653	11794	11889	11732	12578	12464	12227	12676	Bacteria	Firmicutes	Clostridia	Clostridiales	Clostridiaceae	Clostridium	unclassified
45735	10329	10974	9411	8247	11837	12240	11064	11900	Bacteria	Firmicutes	Clostridia	Clostridiales	Clostridiaceae	Clostridium	unclassified
45861	8547	9300	6362	6814	11800	12172	11043	11657	Bacteria	Firmicutes	Clostridia	Clostridiales	Clostridiaceae	Clostridium	unclassified
72582	9497	9208	7040	7717	12221	12828	12221	12616	Bacteria	Firmicutes	Clostridia	Clostridiales	Clostridiaceae	unclassified	unclassified
72795	9211	9705	8802	8268	11863	12271	11772	11793	Bacteria	Firmicutes	Clostridia	Clostridiales	Clostridiaceae	unclassified	unclassified
71089	7858	6744	6596	6901	11164	11303	11855	11620	Bacteria	Firmicutes	Clostridia	Clostridiales	Clostridiales Family XI. Incertae Sedis	Anaerococcus	Anaerococcus hydrogenalis
71224	9835	10063	9555	9130	11573	12355	11898	12424	Bacteria	Firmicutes	Clostridia	Clostridiales	Clostridiales Family XI. Incertae Sedis	Anaerococcus	Anaerococcus prevotii
70669	10952	11247	10307	10266	11918	12620	12386	12634	Bacteria	Firmicutes	Clostridia	Clostridiales	Clostridiales Family XI. Incertae Sedis	Anaerococcus	unclassified
70706	9153	9177	8659	7756	12053	12326	12182	12582	Bacteria	Firmicutes	Clostridia	Clostridiales	Clostridiales Family XI. Incertae Sedis	Anaerococcus	unclassified
70712	8468	9475	8222	7593	11885	12163	12520	13083	Bacteria	Firmicutes	Clostridia	Clostridiales	Clostridiales Family XI. Incertae Sedis	Anaerococcus	unclassified
70850	9770	11313	11102	10100	12474	12807	12876	13243	Bacteria	Firmicutes	Clostridia	Clostridiales	Clostridiales Family XI. Incertae Sedis	Anaerococcus	unclassified
70959	8966	9881	9204	8023	12937	13152	12786	13196	Bacteria	Firmicutes	Clostridia	Clostridiales	Clostridiales Family XI. Incertae Sedis	Anaerococcus	unclassified
71020	9774	9500	8422	7612	12840	13045	12863	13282	Bacteria	Firmicutes	Clostridia	Clostridiales	Clostridiales Family XI. Incertae Sedis	Anaerococcus	unclassified
71075	9907	10162	9401	9252	12916	12884	12969	13319	Bacteria	Firmicutes	Clostridia	Clostridiales	Clostridiales Family XI. Incertae Sedis	Anaerococcus	unclassified
71091	9527	8481	6686	8804	12361	12306	12535	12998	Bacteria	Firmicutes	Clostridia	Clostridiales	Clostridiales Family XI. Incertae Sedis	Anaerococcus	unclassified
71113	9162	8870	8465	8160	11420	11669	11714	12183	Bacteria	Firmicutes	Clostridia	Clostridiales	Clostridiales Family XI. Incertae Sedis	Anaerococcus	unclassified
71191	9211	9656	8919	7825	12616	12373	12668	13004	Bacteria	Firmicutes	Clostridia	Clostridiales	Clostridiales Family XI. Incertae Sedis	Anaerococcus	unclassified
71202	10046	10237	7957	9002	13283	13205	13255	13981	Bacteria	Firmicutes	Clostridia	Clostridiales	Clostridiales Family XI. Incertae Sedis	Anaerococcus	unclassified
71226	11828	12122	11634	11164	12829	13064	13106	13677	Bacteria	Firmicutes	Clostridia	Clostridiales	Clostridiales Family XI. Incertae Sedis	Anaerococcus	unclassified
70051	9122	7812	6241	6814	11778	11899	12750	13293	Bacteria	Firmicutes	Clostridia	Clostridiales	Clostridiales Family XI. Incertae Sedis	Finegoldia	Finegoldia magna
69992	9133	7817	6984	7290	11648	11924	12487	13047	Bacteria	Firmicutes	Clostridia	Clostridiales	Clostridiales Family XI. Incertae Sedis	Finegoldia	unclassified
70627	8846	7934	8283	7169	12074	12357	12484	12557	Bacteria	Firmicutes	Clostridia	Clostridiales	Clostridiales Family XI. Incertae Sedis	Helcococcus	unclassified
70180	9022	8553	8619	7035	11786	12293	12140	12690	Bacteria	Firmicutes	Clostridia	Clostridiales	Clostridiales Family XI. Incertae Sedis	Peptoniphilus	unclassified
70289	9090	7911	6501	8604	12035	12934	12296	12952	Bacteria	Firmicutes	Clostridia	Clostridiales	Clostridiales Family XI. Incertae Sedis	Peptoniphilus	unclassified
69286	8679	9090	6371	7156	11005	11902	11223	11760	Bacteria	Firmicutes	Clostridia	Clostridiales	Clostridiales Family XI. Incertae Sedis	unclassified	unclassified
69417	9294	8739	8815	8543	10855	12463	11056	11020	Bacteria	Firmicutes	Clostridia	Clostridiales	Clostridiales Family XI. Incertae Sedis	unclassified	unclassified
69681	8609	8185	7649	7147	10759	11884	11197	11821	Bacteria	Firmicutes	Clostridia	Clostridiales	Clostridiales Family XI. Incertae Sedis	unclassified	unclassified
70082	10097	10615	9593	9279	12987	13367	13145	13626	Bacteria	Firmicutes	Clostridia	Clostridiales	Clostridiales Family XIII. Incertae Sedis	Eubacterium	unclassified
70118	10980	11763	11432	9914	11733	12482	11952	12031	Bacteria	Firmicutes	Clostridia	Clostridiales	Clostridiales Family XIII. Incertae Sedis	Eubacterium	unclassified
69633	8344	8608	7117	7174	10675	11732	10999	11727	Bacteria	Firmicutes	Clostridia	Clostridiales	Eubacteriaceae	unclassified	unclassified
20652	10007	8939	8075	7786	11900	12515	12065	12783	Bacteria	Firmicutes	Clostridia	Clostridiales	Lachnospiraceae	Blautia	unclassified
21155	9285	9136	8479	8926	11160	11997	11607	11985	Bacteria	Firmicutes	Clostridia	Clostridiales	Lachnospiraceae	Blautia	unclassified
23140	8716	7947	7233	7179	10785	11862	11094	11609	Bacteria	Firmicutes	Clostridia	Clostridiales	Lachnospiraceae	Butyrivibrio	unclassified
23182	10024	10035	8060	7932	11611	12434	11722	12108	Bacteria	Firmicutes	Clostridia	Clostridiales	Lachnospiraceae	Butyrivibrio	unclassified
20192	9866	10136	9320	8788	11268	12194	11393	12118	Bacteria	Firmicutes	Clostridia	Clostridiales	Lachnospiraceae	Coprococcus	unclassified
22993	8675	8995	7167	7111	11766	12525	12214	12733	Bacteria	Firmicutes	Clostridia	Clostridiales	Lachnospiraceae	Coprococcus	unclassified
24363	9637	10287	7767	8204	12142	13675	12731	13099	Bacteria	Firmicutes	Clostridia	Clostridiales	Lachnospiraceae	Coprococcus	unclassified
19752	8284	7478	6372	6644	10762	11891	11490	11529	Bacteria	Firmicutes	Clostridia	Clostridiales	Lachnospiraceae	Lachnospira	unclassified
17427	7672	6754	6701	7223	10657	10582	10704	11815	Bacteria	Firmicutes	Clostridia	Clostridiales	Lachnospiraceae	Roseburia	Eubacterium rectale
17447	8286	7356	7001	8010	11203	10548	11086	12614	Bacteria	Firmicutes	Clostridia	Clostridiales	Lachnospiraceae	Roseburia	Eubacterium rectale
17519	8166	7828	6849	7067	11737	11693	11988	12656	Bacteria	Firmicutes	Clostridia	Clostridiales	Lachnospiraceae	Roseburia	Eubacterium rectale
17661	7812	7644	6677	6943	11012	10822	10647	11996	Bacteria	Firmicutes	Clostridia	Clostridiales	Lachnospiraceae	Roseburia	Eubacterium rectale
17677	8350	7506	6757	7356	11444	10823	11327	12227	Bacteria	Firmicutes	Clostridia	Clostridiales	Lachnospiraceae	Roseburia	Eubacterium rectale
17726	8497	8959	7189	7380	11138	10822	11055	12282	Bacteria	Firmicutes	Clostridia	Clostridiales	Lachnospiraceae	Roseburia	Eubacterium rectale
17744	8376	7307	7805	7460	10712	9994	10417	12028	Bacteria	Firmicutes	Clostridia	Clostridiales	Lachnospiraceae	Roseburia	Eubacterium rectale
17765	9572	10229	7780	8390	12940	12918	13155	13759	Bacteria	Firmicutes	Clostridia	Clostridiales	Lachnospiraceae	Roseburia	Eubacterium rectale
17792	9789	10012	8934	8689	10448	10976	10489	11699	Bacteria	Firmicutes	Clostridia	Clostridiales	Lachnospiraceae	Roseburia	Eubacterium rectale
17828	8256	7465	6520	6913	11275	11050	11265	12203	Bacteria	Firmicutes	Clostridia	Clostridiales	Lachnospiraceae	Roseburia	Eubacterium rectale
17893	8346	6742	6785	7751	11982	11343	11792	12779	Bacteria	Firmicutes	Clostridia	Clostridiales	Lachnospiraceae	Roseburia	Eubacterium rectale
17943	8565	8732	8396	8641	10539	10881	10080	11766	Bacteria	Firmicutes	Clostridia	Clostridiales	Lachnospiraceae	Roseburia	Eubacterium rectale
17964	7912	7154	6953	7723	10983	10406	10786	12249	Bacteria	Firmicutes	Clostridia	Clostridiales	Lachnospiraceae	Roseburia	Eubacterium rectale
18113	8429	7551	6849	7259	10947	10700	11009	11847	Bacteria	Firmicutes	Clostridia	Clostridiales	Lachnospiraceae	Roseburia	Eubacterium rectale
18137	8293	7291	6753	7319	11649	11158	11744	12759	Bacteria	Firmicutes	Clostridia	Clostridiales	Lachnospiraceae	Roseburia	Eubacterium rectale
18175	8220	7730	6854	7979	11190	11007	11302	12365	Bacteria	Firmicutes	Clostridia	Clostridiales	Lachnospiraceae	Roseburia	Eubacterium rectale
18222	7658	7575	6501	6677	11288	11802	11345	12241	Bacteria	Firmicutes	Clostridia	Clostridiales	Lachnospiraceae	Roseburia	Eubacterium rectale
18252	8646	8058	7107	6955	11380	11146	11376	12119	Bacteria	Firmicutes	Clostridia	Clostridiales	Lachnospiraceae	Roseburia	Eubacterium rectale
18254	8456	7659	6974	7463	11390	11017	11392	12380	Bacteria	Firmicutes	Clostridia	Clostridiales	Lachnospiraceae	Roseburia	Eubacterium rectale
18281	9243	9347	7424	8430	10567	10575	10386	11633	Bacteria	Firmicutes	Clostridia	Clostridiales	Lachnospiraceae	Roseburia	Eubacterium rectale
17852	8628	6788	6807	6834	11063	11701	11557	12307	Bacteria	Firmicutes	Clostridia	Clostridiales	Lachnospiraceae	Roseburia	unclassified
18761	9721	9816	9356	8821	11652	12189	11860	12081	Bacteria	Firmicutes	Clostridia	Clostridiales	Lachnospiraceae	Ruminococcus	unclassified
19470	11182	11997	11485	10329	12235	12459	12693	12785	Bacteria	Firmicutes	Clostridia	Clostridiales	Lachnospiraceae	unclassified	unclassified
20782	9272	9142	8314	7192	11789	12315	12016	12298	Bacteria	Firmicutes	Clostridia	Clostridiales	Lachnospiraceae	unclassified	unclassified
23349	8857	7970	6401	7144	11855	12089	11375	11811	Bacteria	Firmicutes	Clostridia	Clostridiales	Lachnospiraceae	unclassified	unclassified
23897	8328	8246	7171	7618	10956	12552	11676	12087	Bacteria	Firmicutes	Clostridia	Clostridiales	Lachnospiraceae	unclassified	unclassified
24119	9281	9692	9462	8630	11281	12096	11132	11450	Bacteria	Firmicutes	Clostridia	Clostridiales	Lachnospiraceae	unclassified	unclassified
69319	10092	10720	8500	8181	12175	12649	12295	12674	Bacteria	Firmicutes	Clostridia	Clostridiales	Lachnospiraceae	unclassified	unclassified
70043	9032	8339	7465	7469	11596	12243	12228	12593	Bacteria	Firmicutes	Clostridia	Clostridiales	Lachnospiraceae	unclassified	unclassified
70179	9828	9318	7032	10141	12628	13023	12582	13197	Bacteria	Firmicutes	Clostridia	Clostridiales	Lachnospiraceae	unclassified	unclassified
70417	8267	8661	7653	6825	10601	11672	10907	12026	Bacteria	Firmicutes	Clostridia	Clostridiales	Lachnospiraceae	unclassified	unclassified
70425	8467	7844	7434	8046	9282	12662	9391	9915	Bacteria	Firmicutes	Clostridia	Clostridiales	Lachnospiraceae	unclassified	unclassified

Table S6 (part O): HybScores of OTUs that were called present in at least one of the samples. Taxonomic classifications are based on PhyloChip taxonomy (derived from GreenGenes). Please note, that data presented here was rank normalized before performing statisical analyses presented in the manuscript.

	Sample ID (PMA)				Sample ID				taxonomic classification of representative rOTU cluster						
OTU	UR_PMA	CO_PMA	CR8_PMA	CR5_PMA	UR	CO	CR8	CR5	kingdom	phylum	class	order	family	genus	species
70485	9145	9873	6958	6785	11833	12711	11678	12610	Bacteria	Firmicutes	Clostridia	Clostridiales	Lachnospiraceae	unclassified	unclassified
70579	8937	9039	8095	7984	12169	12663	11550	12086	Bacteria	Firmicutes	Clostridia	Clostridiales	Lachnospiraceae	unclassified	unclassified
70606	11199	11538	10735	10434	11826	12701	11967	12865	Bacteria	Firmicutes	Clostridia	Clostridiales	Lachnospiraceae	unclassified	unclassified
70639	10477	11255	10555	10136	12365	12613	12033	12050	Bacteria	Firmicutes	Clostridia	Clostridiales	Lachnospiraceae	unclassified	unclassified
70729	9548	10493	9205	8920	11722	12321	11956	11944	Bacteria	Firmicutes	Clostridia	Clostridiales	Lachnospiraceae	unclassified	unclassified
70775	8747	8046	7874	7708	11500	12138	11768	11965	Bacteria	Firmicutes	Clostridia	Clostridiales	Lachnospiraceae	unclassified	unclassified
70838	8780	8576	7074	6961	11158	12020	11105	11807	Bacteria	Firmicutes	Clostridia	Clostridiales	Lachnospiraceae	unclassified	unclassified
70892	9388	7485	6735	8735	11289	12258	11450	11704	Bacteria	Firmicutes	Clostridia	Clostridiales	Lachnospiraceae	unclassified	unclassified
70902	9594	9782	9449	9056	11073	11777	11184	11410	Bacteria	Firmicutes	Clostridia	Clostridiales	Lachnospiraceae	unclassified	unclassified
70911	9939	11747	8284	9044	13063	13374	12774	13164	Bacteria	Firmicutes	Clostridia	Clostridiales	Lachnospiraceae	unclassified	unclassified
70956	8508	7242	7293	7870	10981	12007	11262	11785	Bacteria	Firmicutes	Clostridia	Clostridiales	Lachnospiraceae	unclassified	unclassified
70970	11214	12142	11261	8902	12724	12710	12314	12401	Bacteria	Firmicutes	Clostridia	Clostridiales	Lachnospiraceae	unclassified	unclassified
71056	9455	10230	9270	8742	12072	12838	12457	12726	Bacteria	Firmicutes	Clostridia	Clostridiales	Lachnospiraceae	unclassified	unclassified
71105	8526	7050	6906	7891	10663	12160	10985	11639	Bacteria	Firmicutes	Clostridia	Clostridiales	Lachnospiraceae	unclassified	unclassified
71159	8590	7060	7279	8080	10796	11871	11016	11435	Bacteria	Firmicutes	Clostridia	Clostridiales	Lachnospiraceae	unclassified	unclassified
71178	8539	7141	6538	7608	10864	11819	11007	11372	Bacteria	Firmicutes	Clostridia	Clostridiales	Lachnospiraceae	unclassified	unclassified
71190	10589	10611	10097	9459	12831	13010	12718	13015	Bacteria	Firmicutes	Clostridia	Clostridiales	Lachnospiraceae	unclassified	unclassified
71261	10020	10952	10257	9108	12144	12419	12054	12585	Bacteria	Firmicutes	Clostridia	Clostridiales	Lachnospiraceae	unclassified	unclassified
71274	9492	9668	9291	7969	11920	13307	12198	12528	Bacteria	Firmicutes	Clostridia	Clostridiales	Lachnospiraceae	unclassified	unclassified
48912	8620	8008	7629	7268	11300	12388	11580	11896	Bacteria	Firmicutes	Clostridia	Clostridiales	Peptococcaceae	Desulfotomaculum	unclassified
49213	8797	8841	8429	7544	10437	11725	10657	11342	Bacteria	Firmicutes	Clostridia	Clostridiales	Peptococcaceae	Desulfotomaculum	unclassified
48830	10208	11420	10703	10811	13235	13175	13253	13302	Bacteria	Firmicutes	Clostridia	Clostridiales	Peptococcaceae	unclassified	unclassified
24602	8977	9805	8715	7890	11545	12434	12142	12251	Bacteria	Firmicutes	Clostridia	Clostridiales	Ruminococcaceae	Faecalibacterium	Faecalibacterium prausnitzii
24700	8589	8267	6993	7278	11597	11938	11632	12035	Bacteria	Firmicutes	Clostridia	Clostridiales	Ruminococcaceae	Faecalibacterium	Faecalibacterium prausnitzii
24841	7987	7582	6916	6971	10841	12027	11456	11784	Bacteria	Firmicutes	Clostridia	Clostridiales	Ruminococcaceae	Faecalibacterium	Faecalibacterium prausnitzii
25077	8338	7697	7067	6848	10831	11883	11126	11714	Bacteria	Firmicutes	Clostridia	Clostridiales	Ruminococcaceae	Faecalibacterium	Faecalibacterium prausnitzii
25078	7719	7215	6027	6569	11057	12295	11726	11918	Bacteria	Firmicutes	Clostridia	Clostridiales	Ruminococcaceae	Faecalibacterium	Faecalibacterium prausnitzii
25139	8693	8940	7564	6641	11930	12505	11880	12653	Bacteria	Firmicutes	Clostridia	Clostridiales	Ruminococcaceae	Faecalibacterium	Faecalibacterium prausnitzii
25273	8170	7465	6963	7333	10632	11677	11039	10950	Bacteria	Firmicutes	Clostridia	Clostridiales	Ruminococcaceae	Faecalibacterium	Faecalibacterium prausnitzii
25288	8660	9036	7738	7333	11732	12453	11805	12359	Bacteria	Firmicutes	Clostridia	Clostridiales	Ruminococcaceae	Faecalibacterium	Faecalibacterium prausnitzii
25319	8243	7402	6653	7011	10522	11767	11246	11122	Bacteria	Firmicutes	Clostridia	Clostridiales	Ruminococcaceae	Faecalibacterium	Faecalibacterium prausnitzii
25325	8455	7312	6496	5742	11137	11759	11524	11955	Bacteria	Firmicutes	Clostridia	Clostridiales	Ruminococcaceae	Faecalibacterium	Faecalibacterium prausnitzii
25425	10706	11428	11031	9624	11993	12559	12205	12483	Bacteria	Firmicutes	Clostridia	Clostridiales	Ruminococcaceae	Faecalibacterium	Faecalibacterium prausnitzii
25549	8306	7436	6680	6837	10383	11785	10605	11252	Bacteria	Firmicutes	Clostridia	Clostridiales	Ruminococcaceae	Faecalibacterium	Faecalibacterium prausnitzii
25759	8635	6934	6139	6684	10200	11729	10846	10598	Bacteria	Firmicutes	Clostridia	Clostridiales	Ruminococcaceae	Faecalibacterium	Faecalibacterium prausnitzii
25804	8300	7672	7050	6977	11047	11964	11449	10963	Bacteria	Firmicutes	Clostridia	Clostridiales	Ruminococcaceae	Faecalibacterium	Faecalibacterium prausnitzii
25901	10958	10892	10702	10685	13193	13219	12988	13295	Bacteria	Firmicutes	Clostridia	Clostridiales	Ruminococcaceae	Faecalibacterium	Faecalibacterium prausnitzii
25914	8496	8832	7625	7252	11219	11971	11363	12021	Bacteria	Firmicutes	Clostridia	Clostridiales	Ruminococcaceae	Faecalibacterium	Faecalibacterium prausnitzii
25950	8272	7544	6757	7025	11218	12251	11704	11295	Bacteria	Firmicutes	Clostridia	Clostridiales	Ruminococcaceae	Faecalibacterium	Faecalibacterium prausnitzii
26109	7950	7359	6689	6960	10725	12075	11381	11678	Bacteria	Firmicutes	Clostridia	Clostridiales	Ruminococcaceae	Faecalibacterium	Faecalibacterium prausnitzii
26219	8053	7091	6489	6929	10797	12034	10984	11459	Bacteria	Firmicutes	Clostridia	Clostridiales	Ruminococcaceae	Faecalibacterium	Faecalibacterium prausnitzii
26288	9713	9829	7668	7452	12945	13200	12988	13720	Bacteria	Firmicutes	Clostridia	Clostridiales	Ruminococcaceae	Faecalibacterium	Faecalibacterium prausnitzii
26371	9675	10019	9087	9022	11689	12602	11913	12470	Bacteria	Firmicutes	Clostridia	Clostridiales	Ruminococcaceae	Faecalibacterium	Faecalibacterium prausnitzii
26393	8347	7692	6814	6519	11142	11781	11615	11824	Bacteria	Firmicutes	Clostridia	Clostridiales	Ruminococcaceae	Faecalibacterium	Faecalibacterium prausnitzii
26430	7844	6527	6092	6514	10320	11954	11133	10752	Bacteria	Firmicutes	Clostridia	Clostridiales	Ruminococcaceae	Faecalibacterium	Faecalibacterium prausnitzii
26462	10672	10572	10054	10113	12114	12772	12681	13193	Bacteria	Firmicutes	Clostridia	Clostridiales	Ruminococcaceae	Faecalibacterium	Faecalibacterium prausnitzii
26514	8304	7561	6944	7092	11184	12269	11555	11172	Bacteria	Firmicutes	Clostridia	Clostridiales	Ruminococcaceae	Faecalibacterium	Faecalibacterium prausnitzii
26613	7948	6730	6255	6788	10542	11866	10684	11138	Bacteria	Firmicutes	Clostridia	Clostridiales	Ruminococcaceae	Faecalibacterium	Faecalibacterium prausnitzii
24427	10613	11381	10744	8701	11960	12318	12242	12205	Bacteria	Firmicutes	Clostridia	Clostridiales	Ruminococcaceae	Faecalibacterium	unclassified
24447	9422	9485	6712	6968	12856	13098	12982	13647	Bacteria	Firmicutes	Clostridia	Clostridiales	Ruminococcaceae	Faecalibacterium	unclassified
24455	8004	7496	6708	7015	10323	11751	10992	11348	Bacteria	Firmicutes	Clostridia	Clostridiales	Ruminococcaceae	Faecalibacterium	unclassified
24509	9127	8993	6986	6922	12475	12634	12508	13296	Bacteria	Firmicutes	Clostridia	Clostridiales	Ruminococcaceae	Faecalibacterium	unclassified
24544	11014	11009	10613	9451	12425	12887	12347	12851	Bacteria	Firmicutes	Clostridia	Clostridiales	Ruminococcaceae	Faecalibacterium	unclassified
24600	8446	7700	7075	7759	10251	11860	10942	10998	Bacteria	Firmicutes	Clostridia	Clostridiales	Ruminococcaceae	Faecalibacterium	unclassified
24608	7873	6797	6519	7307	10300	11753	10949	11060	Bacteria	Firmicutes	Clostridia	Clostridiales	Ruminococcaceae	Faecalibacterium	unclassified
24646	7802	7149	6628	7395	10258	11747	10902	10963	Bacteria	Firmicutes	Clostridia	Clostridiales	Ruminococcaceae	Faecalibacterium	unclassified
24857	8498	8809	7616	7221	11335	12123	11502	12148	Bacteria	Firmicutes	Clostridia	Clostridiales	Ruminococcaceae	Faecalibacterium	unclassified
24978	9202	8535	8016	7301	11555	12300	12319	12593	Bacteria	Firmicutes	Clostridia	Clostridiales	Ruminococcaceae	Faecalibacterium	unclassified
24986	12402	12720	12683	12369	11654	12560	12312	12148	Bacteria	Firmicutes	Clostridia	Clostridiales	Ruminococcaceae	Faecalibacterium	unclassified
24996	8555	7912	7312	7658	11116	12177	11749	11845	Bacteria	Firmicutes	Clostridia	Clostridiales	Ruminococcaceae	Faecalibacterium	unclassified
25126	7643	7283	6729	7251	10489	11802	11136	11276	Bacteria	Firmicutes	Clostridia	Clostridiales	Ruminococcaceae	Faecalibacterium	unclassified
25221	8635	9014	7043	6888	12082	12594	12482	12735	Bacteria	Firmicutes	Clostridia	Clostridiales	Ruminococcaceae	Faecalibacterium	unclassified
25291	7991	7507	6782	7090	10449	11832	11158	11482	Bacteria	Firmicutes	Clostridia	Clostridiales	Ruminococcaceae	Faecalibacterium	unclassified
25341	10252	10804	9265	9038	12753	13178	12852	13128	Bacteria	Firmicutes	Clostridia	Clostridiales	Ruminococcaceae	Faecalibacterium	unclassified
25380	9453	9405	6731	7010	12772	12952	12832	13556	Bacteria	Firmicutes	Clostridia	Clostridiales	Ruminococcaceae	Faecalibacterium	unclassified
25471	9433	9690	6739	7027	13159	13133	13017	13706	Bacteria	Firmicutes	Clostridia	Clostridiales	Ruminococcaceae	Faecalibacterium	unclassified
25499	8315	7608	7404	7782	10938	12043	11353	10672	Bacteria	Firmicutes	Clostridia	Clostridiales	Ruminococcaceae	Faecalibacterium	unclassified
25558	7318	7120	6268	7498	10677	12000	11114	11302	Bacteria	Firmicutes	Clostridia	Clostridiales	Ruminococcaceae	Faecalibacterium	unclassified
25604	10114	10323	9373	7620	10751	11867	11238	11554	Bacteria	Firmicutes	Clostridia	Clostridiales	Ruminococcaceae	Faecalibacterium	unclassified
25637	10227	10309	9952	8693	11781	12215	11676	12284	Bacteria	Firmicutes	Clostridia	Clostridiales	Ruminococcaceae	Faecalibacterium	unclassified
25654	9532	11133	10877	9675	11626	12263	11727	12151	Bacteria	Firmicutes	Clostridia	Clostridiales	Ruminococcaceae	Faecalibacterium	unclassified
25719	8297	7987	7146	7003	10940	11818	11305	11834	Bacteria	Firmicutes	Clostridia	Clostridiales	Ruminococcaceae	Faecalibacterium	unclassified
25994	10102	10398	10042	9250	12319	12652	12238	12575	Bacteria	Firmicutes	Clostridia	Clostridiales	Ruminococcaceae	Faecalibacterium	unclassified
26041	9701	10144	8059	8233	11028	12013	11554	12067	Bacteria	Firmicutes	Clostridia	Clostridiales	Ruminococcaceae	Faecalibacterium	unclassified
26129	8392	7129	6945	7172	12048	11990	11544	11352	Bacteria	Firmicutes	Clostridia	Clostridiales	Ruminococcaceae	Faecalibacterium	unclassified
26227	8498	8688	6685	7461	12068	12453	12339	12765	Bacteria	Firmicutes	Clostridia	Clostridiales	Ruminococcaceae	Faecalibacterium	unclassified
26356	11389	8024	5896	6960	11267	11745	11461	11665	Bacteria	Firmicutes	Clostridia	Clostridiales	Ruminococcaceae	Faecalibacterium	unclassified
26599	8408	8753	7626	7149	11257	12082	11470	12146	Bacteria	Firmicutes	Clostridia	Clostridiales	Ruminococcaceae	Faecalibacterium	unclassified
26611	11005	7920	6045	7054	11227	11793	11465	11761	Bacteria	Firmicutes	Clostridia	Clostridiales	Ruminococcaceae	Faecalibacterium	unclassified
26746	9626	9817	9660	8626	11628	12132	11370	12326	Bacteria	Firmicutes	Clostridia	Clostridiales	Ruminococcaceae	Ruminococcus	Ruminococcus bromii
26910	8594	9108	6904	7714	10956	11990	11366	11415	Bacteria	Firmicutes	Clostridia	Clostridiales	Ruminococcaceae	Ruminococcus	Ruminococcus bromii
26768	9479	8984	8579	7446	11800	12505	11815	11934	Bacteria	Firmicutes	Clostridia	Clostridiales	Ruminococcaceae	Ruminococcus	unclassified
27392	10113	8308	7403	8107	11325	12091	11852	12183	Bacteria	Firmicutes	Clostridia	Clostridiales	Ruminococcaceae	Ruminococcus	unclassified
27536	8831	9316	7960	7980	11555	12515	11839	12066	Bacteria	Firmicutes	Clostridia	Clostridiales	Ruminococcaceae	Ruminococcus	unclassified
28159	9744	8647	7848	7371	11514	12514	12199	12607	Bacteria	Firmicutes	Clostridia	Clostridiales	Ruminococcaceae	Ruminococcus	unclassified
22140	9691	10106	9585	8322	11789	12297	12225	12449	Bacteria	Firmicutes	Clostridia	Clostridiales	Ruminococcaceae	unclassified	unclassified
22190	8223	8540	6738	7073	10741	12305	11516	11894	Bacteria	Firmicutes	Clostridia	Clostridiales	Ruminococcaceae	unclassified	unclassified
24612	8042	7087	6537	7014	10019	11763	10480	11397	Bacteria	Firmicutes	Clostridia	Clostridiales	Ruminococcaceae	unclassified	unclassified
26269	8750	7798	7733	7560	10724	11488	10665	11708	Bacteria	Firmicutes	Clostridia	Clostridiales	Ruminococcaceae	unclassified	unclassified
26744	7873	8776	6453	7507	11190	11733	10701	11211	Bacteria	Firmicutes	Clostridia	Clostridiales	Ruminococcaceae	unclassified	unclassified
26984	8804	8424	7963	6745	11114	11803	11541	12000	Bacteria	Firmicutes	Clostridia	Clostridiales	Ruminococcaceae	unclassified	unclassified
28064	8744	9159	7282	6848	12041	12753	12490	12953	Bacteria	Firmicutes	Clostridia	Clostridiales	Ruminococcaceae	unclassified	unclassified
28113	9574	8613	7610	7492	12289	12974	12571	12924	Bacteria	Firmicutes	Clostridia	Clostridiales	Ruminococcaceae	unclassified	unclassified
49101	9126	8652	7587	7476	11954	12163	11950	11724	Bacteria	Firmicutes	Clostridia	Clostridiales	Thermoanaerobacterales Family III. Incertae Sedis	Thermoanaerobacterium	Thermoanaerobacterium saccharolyticum
50564	9327	11297	9410	8175	12439	12894	12557	12871	Bacteria	Firmicutes	Clostridia	Clostridiales	Veillonellaceae	unclassified	unclassified
49990	12130	12032	12133	9877	11316	12485	12256	11644	Bacteria	Firmicutes	Clostridia	Clostridiales	Veillonellaceae	Veillonella	unclassified
50043	8457	7500	6929	8033	10104	11723	11454	10073	Bacteria	Firmicutes	Clostridia	Clostridiales	Veillonellaceae	Veillonella	unclassified
50073	8293	6465	6308	7137	9967	11880	11142	10383	Bacteria	Firmicutes	Clostridia	Clostridiales	Veillonellaceae	Veillonella	unclassified
50464	9168	9264	8406	8005	11411	12720	12198	11857	Bacteria	Firmicutes	Clostridia	Clostridiales	Veillonellaceae	Veillonella	unclassified
50569	8880	7810	8071	7936	10707	12351	11920	11075	Bacteria	Firmicutes	Clostridia	Clostridiales	Veillonellaceae	Veillonella	unclassified

Table S6 (part P): HybScores of OTUs that were called present in at least one of the samples. Taxonomic classifications are based on PhyloChip taxonomy (derived from GreenGenes). Please note, that data presented here was rank normalized before performing statisical analyses presented in the manuscript.

	Sample ID (PMA)				Sample ID				taxonomic classification of representative rOTU cluster						
OTU	UR_PMA	CO_PMA	CR8_PMA	CR5_PMA	UR	CO	CR8	CR5	kingdom	phylum	class	order	family	genus	species
50707	9140	9239	8398	7998	11354	12704	12117	11792	Bacteria	Firmicutes	Clostridia	Clostridiales	Veillonellaceae	Veillonella	unclassified
50725	8459	7560	6884	7996	10195	11744	11536	10206	Bacteria	Firmicutes	Clostridia	Clostridiales	Veillonellaceae	Veillonella	unclassified
50574	8712	7580	6985	7313	10784	11930	11060	11456	Bacteria	Firmicutes	Clostridia	Clostridiales	Veillonellaceae	Veillonella	Veillonella dispar
58422	9261	9480	8146	8293	10684	12126	10875	11029	Bacteria	Firmicutes	Clostridia	MBA08	unclassified	sfA	unclassified
71919	9342	9511	7336	7451	11391	12018	11819	12123	Bacteria	Fusobacteria	Fusobacteria	Fusobacteriales	Fusobacteriaceae	Leptotrichia	unclassified
57878	8927	8887	6790	7308	11349	12335	11493	12297	Bacteria	Gemmatimonadetes	Gemmatimonadetes	Gemmatimonadales	Gemmatimonadaceae	Gemmatimonas	unclassified
58123	10338	10665	10078	8872	12447	12929	12302	13022	Bacteria	Gemmatimonadetes	Gemmatimonadetes	Gemmatimonadales	Gemmatimonadaceae	unclassified	unclassified
58535	8188	8945	6341	6902	11010	12502	11235	11861	Bacteria	Gemmatimonadetes	Gemmatimonadetes	Gemmatimonadales	Gemmatimonadaceae	unclassified	unclassified
58567	10225	10813	10177	9243	12248	13345	12373	13021	Bacteria	Gemmatimonadetes	Gemmatimonadetes	Gemmatimonadales	Gemmatimonadaceae	unclassified	unclassified
58619	9321	9762	9371	8557	11703	13227	11976	12703	Bacteria	Gemmatimonadetes	Gemmatimonadetes	Gemmatimonadales	Gemmatimonadaceae	unclassified	unclassified
58625	8469	10034	8170	7388	11769	12922	11736	12429	Bacteria	Gemmatimonadetes	Gemmatimonadetes	Gemmatimonadales	Gemmatimonadaceae	unclassified	unclassified
58728	10689	11191	10626	9540	11438	12265	11693	11944	Bacteria	Gemmatimonadetes	Gemmatimonadetes	unclassified	unclassified	sfF	unclassified
58534	8909	9710	8814	7987	11230	12322	11786	11703	Bacteria	Gemmatimonadetes	Gemmatimonadetes	unclassified	unclassified	sfG	unclassified
57982	11889	12964	11887	11044	13856	13833	13674	14035	Bacteria	Gemmatimonadetes	Gemmatimonadetes	unclassified	unclassified	sfM	unclassified
58818	9602	10253	9132	7791	12350	12694	12163	12184	Bacteria	GN04	Sbav_B12	unclassified	unclassified	sfA	unclassified
58316	8464	9580	8703	7955	12347	12603	12105	12342	Bacteria	MD2896-B26	unclassified	unclassified	unclassified	sfA	unclassified
52294	8919	9510	7491	7641	11097	11848	11877	11579	Bacteria	Planctomycetes	C6	unclassified	unclassified	sfC	unclassified
57308	9985	10694	9985	8854	12140	12730	12503	12393	Bacteria	Planctomycetes	Planctomycea	Gemmatales	Isosphaeraceae	Isosphaera	unclassified
57019	7878	8174	6718	7092	10049	11877	11481	11116	Bacteria	Planctomycetes	Planctomycea	Gemmatales	Isosphaeraceae	unclassified	unclassified
57583	8235	10225	6943	6971	11255	12210	12249	11718	Bacteria	Planctomycetes	Planctomycea	Gemmatales	Isosphaeraceae	unclassified	unclassified
57616	9428	11368	8815	8878	11478	12717	11367	11269	Bacteria	Planctomycetes	Planctomycea	Gemmatales	Isosphaeraceae	unclassified	unclassified
57357	9500	10283	8720	8292	12712	13622	13211	13077	Bacteria	Planctomycetes	Planctomycea	Planctomycetales	Planctomycetaceae	Planctomyces	unclassified
58971	11668	11859	11840	11595	12954	12959	12540	12813	Bacteria	Proteobacteria	Alphaproteobacteria	Caulobacterales	Caulobacteraceae	Brevundimonas	Brevundimonas diminuta
58987	10277	11099	9365	9432	12954	12942	12745	12840	Bacteria	Proteobacteria	Alphaproteobacteria	Caulobacterales	Caulobacteraceae	Brevundimonas	Brevundimonas diminuta
59071	8117	7716	8383	7313	11775	11912	11851	11139	Bacteria	Proteobacteria	Alphaproteobacteria	Caulobacterales	Caulobacteraceae	Brevundimonas	Brevundimonas diminuta
59158	10168	10584	10144	8628	12167	12159	12009	11514	Bacteria	Proteobacteria	Alphaproteobacteria	Caulobacterales	Caulobacteraceae	Brevundimonas	Brevundimonas diminuta
59195	10144	9089	10095	10247	12244	12148	12094	11779	Bacteria	Proteobacteria	Alphaproteobacteria	Caulobacterales	Caulobacteraceae	Brevundimonas	Brevundimonas diminuta
59196	11684	12097	11851	10665	13061	12787	12827	12603	Bacteria	Proteobacteria	Alphaproteobacteria	Caulobacterales	Caulobacteraceae	Brevundimonas	Brevundimonas diminuta
59284	10881	11342	10273	10638	11822	12274	11821	12044	Bacteria	Proteobacteria	Alphaproteobacteria	Caulobacterales	Caulobacteraceae	Brevundimonas	Brevundimonas diminuta
59317	9913	10604	9781	9234	11609	12156	11705	11695	Bacteria	Proteobacteria	Alphaproteobacteria	Caulobacterales	Caulobacteraceae	Brevundimonas	Brevundimonas diminuta
59463	11029	9844	9684	9112	12801	13044	11789	11736	Bacteria	Proteobacteria	Alphaproteobacteria	Caulobacterales	Caulobacteraceae	Brevundimonas	Brevundimonas diminuta
59960	10665	11365	10790	9867	12504	12494	12501	12454	Bacteria	Proteobacteria	Alphaproteobacteria	Caulobacterales	Caulobacteraceae	Brevundimonas	Brevundimonas diminuta
60065	8201	9020	9600	7908	12185	12116	12081	10844	Bacteria	Proteobacteria	Alphaproteobacteria	Caulobacterales	Caulobacteraceae	Brevundimonas	Brevundimonas diminuta
58982	8261	9594	8019	7761	12026	11777	11585	11526	Bacteria	Proteobacteria	Alphaproteobacteria	Caulobacterales	Caulobacteraceae	Brevundimonas	unclassified
59062	10724	11200	10439	10462	13275	12996	12758	12645	Bacteria	Proteobacteria	Alphaproteobacteria	Caulobacterales	Caulobacteraceae	Brevundimonas	unclassified
59178	8500	10517	9592	8464	13263	12649	12344	11844	Bacteria	Proteobacteria	Alphaproteobacteria	Caulobacterales	Caulobacteraceae	Brevundimonas	unclassified
59740	8490	9684	8097	7448	12042	11984	11666	11683	Bacteria	Proteobacteria	Alphaproteobacteria	Caulobacterales	Caulobacteraceae	Brevundimonas	unclassified
59875	9624	10495	9330	8873	12982	12761	12695	12693	Bacteria	Proteobacteria	Alphaproteobacteria	Caulobacterales	Caulobacteraceae	Brevundimonas	unclassified
60072	7809	9334	7291	6516	12352	12304	11656	11805	Bacteria	Proteobacteria	Alphaproteobacteria	Caulobacterales	Caulobacteraceae	Brevundimonas	unclassified
60100	9676	10347	10018	8394	11964	12029	11731	11617	Bacteria	Proteobacteria	Alphaproteobacteria	Caulobacterales	Caulobacteraceae	Brevundimonas	unclassified
60119	8506	9735	8077	8387	12441	12429	12119	12403	Bacteria	Proteobacteria	Alphaproteobacteria	Caulobacterales	Caulobacteraceae	Brevundimonas	unclassified
59321	10189	10966	10242	7536	10545	11750	10839	11192	Bacteria	Proteobacteria	Alphaproteobacteria	Caulobacterales	Caulobacteraceae	Caulobacter	unclassified
59959	10064	10863	9966	9744	12239	12717	12218	12307	Bacteria	Proteobacteria	Alphaproteobacteria	Caulobacterales	Caulobacteraceae	Caulobacter	unclassified
58959	8304	7477	8721	6594	11561	11930	11758	11195	Bacteria	Proteobacteria	Alphaproteobacteria	Caulobacterales	Caulobacteraceae	Mycoplana	unclassified
59318	8132	7662	8087	7260	12254	12216	12493	11684	Bacteria	Proteobacteria	Alphaproteobacteria	Caulobacterales	Caulobacteraceae	Mycoplana	unclassified
59299	10105	9378	9339	8926	12166	12394	12020	11981	Bacteria	Proteobacteria	Alphaproteobacteria	Caulobacterales	Caulobacteraceae	unclassified	unclassified
59421	10734	11750	10673	10286	12404	13018	12645	12764	Bacteria	Proteobacteria	Alphaproteobacteria	Caulobacterales	Caulobacteraceae	unclassified	unclassified
59755	8778	8072	9793	8226	12056	11975	11993	10856	Bacteria	Proteobacteria	Alphaproteobacteria	Caulobacterales	Caulobacteraceae	unclassified	unclassified
59760	8893	7549	9224	7399	12261	12537	12389	11868	Bacteria	Proteobacteria	Alphaproteobacteria	Caulobacterales	Caulobacteraceae	unclassified	unclassified
60050	9039	10034	8555	7604	11879	12359	12092	12237	Bacteria	Proteobacteria	Alphaproteobacteria	Caulobacterales	Caulobacteraceae	unclassified	unclassified
60071	9698	10177	10052	9114	12213	13263	12510	13019	Bacteria	Proteobacteria	Alphaproteobacteria	Caulobacterales	Caulobacteraceae	unclassified	unclassified
59989	14000	14218	14173	13535	12252	12633	13243	12931	Bacteria	Proteobacteria	Alphaproteobacteria	Rhizobiales	Bradyrhizobiaceae	Afipia	Afipia broomeae
60042	13722	14106	14026	13221	12143	12455	12953	12598	Bacteria	Proteobacteria	Alphaproteobacteria	Rhizobiales	Bradyrhizobiaceae	Afipia	unclassified
58889	10598	10812	10315	10047	12363	12644	12244	12489	Bacteria	Proteobacteria	Alphaproteobacteria	Rhizobiales	Bradyrhizobiaceae	Bosea	unclassified
59322	9026	9729	7858	7894	12206	12420	12164	12182	Bacteria	Proteobacteria	Alphaproteobacteria	Rhizobiales	Bradyrhizobiaceae	Bosea	unclassified
59935	8973	9657	7868	7843	12228	12419	12171	12176	Bacteria	Proteobacteria	Alphaproteobacteria	Rhizobiales	Bradyrhizobiaceae	Bosea	unclassified
58976	12694	12618	12545	11751	12407	12656	12983	12899	Bacteria	Proteobacteria	Alphaproteobacteria	Rhizobiales	Bradyrhizobiaceae	Bradyrhizobium	Bradyrhizobium canariense
58993	13521	14057	13708	13402	13283	13345	13384	13423	Bacteria	Proteobacteria	Alphaproteobacteria	Rhizobiales	Bradyrhizobiaceae	Bradyrhizobium	Bradyrhizobium elkanii
59143	12444	12821	12659	11905	12403	12803	12778	12832	Bacteria	Proteobacteria	Alphaproteobacteria	Rhizobiales	Bradyrhizobiaceae	Bradyrhizobium	unclassified
59486	12811	13198	13043	12508	11396	11956	12380	12159	Bacteria	Proteobacteria	Alphaproteobacteria	Rhizobiales	Bradyrhizobiaceae	Bradyrhizobium	unclassified
59636	13250	13638	13566	12932	11917	12410	12711	12505	Bacteria	Proteobacteria	Alphaproteobacteria	Rhizobiales	Bradyrhizobiaceae	Bradyrhizobium	unclassified
59983	13095	13506	13409	12814	11155	12022	12346	12019	Bacteria	Proteobacteria	Alphaproteobacteria	Rhizobiales	Bradyrhizobiaceae	Bradyrhizobium	unclassified
59027	13128	13499	13456	12817	11825	12273	12541	12354	Bacteria	Proteobacteria	Alphaproteobacteria	Rhizobiales	Bradyrhizobiaceae	unclassified	unclassified
59127	10149	10165	10144	10323	12467	13272	12548	11975	Bacteria	Proteobacteria	Alphaproteobacteria	Rhizobiales	Bradyrhizobiaceae	unclassified	unclassified
59454	13737	13991	13820	13311	12951	13467	13639	13556	Bacteria	Proteobacteria	Alphaproteobacteria	Rhizobiales	Bradyrhizobiaceae	unclassified	unclassified
59524	12293	12715	13190	12026	11189	11810	11824	11868	Bacteria	Proteobacteria	Alphaproteobacteria	Rhizobiales	Bradyrhizobiaceae	unclassified	unclassified
60328	11859	12215	12080	11319	11592	12679	12051	12017	Bacteria	Proteobacteria	Alphaproteobacteria	Rhizobiales	Hyphomicrobiaceae	Devosia	unclassified
60472	12329	12860	12556	11979	12319	13209	12846	12345	Bacteria	Proteobacteria	Alphaproteobacteria	Rhizobiales	Hyphomicrobiaceae	Devosia	unclassified
61285	9918	10022	9686	8478	11741	12469	12006	12066	Bacteria	Proteobacteria	Alphaproteobacteria	Rhizobiales	Hyphomicrobiaceae	Devosia	unclassified
59047	11406	12094	11576	11317	11450	12392	12199	12124	Bacteria	Proteobacteria	Alphaproteobacteria	Rhizobiales	Hyphomicrobiaceae	Rhodoplanes	unclassified
59224	10699	11550	10129	10750	12312	13241	12436	12589	Bacteria	Proteobacteria	Alphaproteobacteria	Rhizobiales	Hyphomicrobiaceae	Rhodoplanes	unclassified
60211	11109	11809	11314	9669	10886	11945	11643	11793	Bacteria	Proteobacteria	Alphaproteobacteria	Rhizobiales	Hyphomicrobiaceae	Rhodoplanes	unclassified
59087	8654	8646	6927	8550	10782	11977	11892	11685	Bacteria	Proteobacteria	Alphaproteobacteria	Rhizobiales	Methylobacteriaceae	Methylobacterium	Methylobacterium adhaesivum
59786	10160	10710	10191	8035	11794	12577	12181	12335	Bacteria	Proteobacteria	Alphaproteobacteria	Rhizobiales	Methylobacteriaceae	Methylobacterium	Methylobacterium fujisawaense
59890	9626	7608	6877	7153	10422	11804	11321	11530	Bacteria	Proteobacteria	Alphaproteobacteria	Rhizobiales	Methylobacteriaceae	Methylobacterium	Methylobacterium fujisawaense
59259	9656	9993	9248	9257	11167	12141	11894	11767	Bacteria	Proteobacteria	Alphaproteobacteria	Rhizobiales	Methylobacteriaceae	Methylobacterium	Methylobacterium komagatae
59420	11440	11614	11691	10690	11606	12488	12328	12427	Bacteria	Proteobacteria	Alphaproteobacteria	Rhizobiales	Methylobacteriaceae	Methylobacterium	Methylobacterium komagatae
59903	11460	11569	11526	10483	11652	12504	12318	12513	Bacteria	Proteobacteria	Alphaproteobacteria	Rhizobiales	Methylobacteriaceae	Methylobacterium	Methylobacterium komagatae
59796	9451	8032	6045	7718	10443	11909	11393	11722	Bacteria	Proteobacteria	Alphaproteobacteria	Rhizobiales	Methylobacteriaceae	Methylobacterium	Methylobacterium radiotolerans
59212	10569	11778	10645	9825	11339	11672	11944	11749	Bacteria	Proteobacteria	Alphaproteobacteria	Rhizobiales	Methylobacteriaceae	Methylobacterium	Methylobacterium zatmanii
59718	10630	11731	10655	9777	11574	12001	12213	12020	Bacteria	Proteobacteria	Alphaproteobacteria	Rhizobiales	Methylobacteriaceae	Methylobacterium	Methylobacterium zatmanii
58886	9812	9592	9035	9000	11016	12494	12161	11792	Bacteria	Proteobacteria	Alphaproteobacteria	Rhizobiales	Methylobacteriaceae	Methylobacterium	unclassified
59247	9855	9271	8565	7637	10905	11972	11589	11771	Bacteria	Proteobacteria	Alphaproteobacteria	Rhizobiales	Methylobacteriaceae	Methylobacterium	unclassified
59404	9147	10715	8410	7631	11252	11687	11804	11613	Bacteria	Proteobacteria	Alphaproteobacteria	Rhizobiales	Methylobacteriaceae	Methylobacterium	unclassified
59410	9440	10774	8694	8365	10957	11666	11848	11605	Bacteria	Proteobacteria	Alphaproteobacteria	Rhizobiales	Methylobacteriaceae	Methylobacterium	unclassified
59417	9384	10789	8636	8279	10726	11449	11655	11379	Bacteria	Proteobacteria	Alphaproteobacteria	Rhizobiales	Methylobacteriaceae	Methylobacterium	unclassified
59547	9815	10156	9417	9222	12439	12921	12511	12773	Bacteria	Proteobacteria	Alphaproteobacteria	Rhizobiales	Methylobacteriaceae	Methylobacterium	unclassified
59549	8634	10686	7673	7414	10816	11322	11726	11331	Bacteria	Proteobacteria	Alphaproteobacteria	Rhizobiales	Methylobacteriaceae	Methylobacterium	unclassified
59551	9889	10704	8442	8120	11849	12679	12493	12588	Bacteria	Proteobacteria	Alphaproteobacteria	Rhizobiales	Methylobacteriaceae	Methylobacterium	unclassified
59560	11479	11753	11624	10567	12238	12935	12724	12718	Bacteria	Proteobacteria	Alphaproteobacteria	Rhizobiales	Methylobacteriaceae	Methylobacterium	unclassified
59571	9673	11072	8540	8364	11411	12051	11977	11790	Bacteria	Proteobacteria	Alphaproteobacteria	Rhizobiales	Methylobacteriaceae	Methylobacterium	unclassified
59710	9636	7073	7539	7236	10435	11816	11334	11459	Bacteria	Proteobacteria	Alphaproteobacteria	Rhizobiales	Methylobacteriaceae	Methylobacterium	unclassified
59725	9875	9372	8759	7688	10938	12736	12651	12867	Bacteria	Proteobacteria	Alphaproteobacteria	Rhizobiales	Methylobacteriaceae	Methylobacterium	unclassified
59754	9539	9287	8679	8532	10394	11767	11121	10972	Bacteria	Proteobacteria	Alphaproteobacteria	Rhizobiales	Methylobacteriaceae	Methylobacterium	unclassified
59791	9876	8075	8464	7702	11244	12253	11805	11962	Bacteria	Proteobacteria	Alphaproteobacteria	Rhizobiales	Methylobacteriaceae	Methylobacterium	unclassified
59904	11399	11422	11181	10963	12008	12822	12251	12364	Bacteria	Proteobacteria	Alphaproteobacteria	Rhizobiales	Methylobacteriaceae	Methylobacterium	unclassified
59915	11504	12362	11757	11150	13012	13304	13297	13172	Bacteria	Proteobacteria	Alphaproteobacteria	Rhizobiales	Methylobacteriaceae	Methylobacterium	unclassified
59826	9963	10631	9404	8948	10674	11904	11768	11183	Bacteria	Proteobacteria	Alphaproteobacteria	Rhizobiales	Methylocystaceae	Methylosinus	Methylosinus sporium
61557	10644	11339	10885	9488	10661	11866	11290	10686	Bacteria	Proteobacteria	Alphaproteobacteria	Rhizobiales	Nordellaceae	Nordella	unclassified
61105	11057	11306	10995	10815	12021	12622	12121	12206	Bacteria	Proteobacteria	Alphaproteobacteria	Rhizobiales	Phyllobacteriaceae	unclassified	unclassified

Table S6 (part Q): HybScores of OTUs that were called present in at least one of the samples. Taxonomic classifications are based on PhyloChip taxonomy (derived from GreenGenes). Please note, that data presented here was rank normalized before performing statisical analyses presented in the manuscript.

	Sample ID (PMA)				Sample ID				taxonomic classification of representative rOTU cluster						
OTU	UR_PMA	CO_PMA	CR8_PMA	CR5_PMA	UR	CO	CR8	CR5	kingdom	phylum	class	order	family	genus	species
61966	11241	11741	11307	11056	12798	13120	11346	12214	Bacteria	Proteobacteria	Alphaproteobacteria	Rhizobiales	Phyllobacteriaceae	unclassified	unclassified
62194	11698	12427	12028	10905	7276	8699	10680	8911	Bacteria	Proteobacteria	Alphaproteobacteria	Rhizobiales	Phyllobacteriaceae	unclassified	unclassified
62538	9765	10359	9642	9049	11565	12500	11292	10967	Bacteria	Proteobacteria	Alphaproteobacteria	Rhizobiales	Phyllobacteriaceae	unclassified	unclassified
62819	9124	9149	8951	7794	10928	12130	11145	10621	Bacteria	Proteobacteria	Alphaproteobacteria	Rhizobiales	Phyllobacteriaceae	unclassified	unclassified
61693	9072	9484	8015	7985	11508	12427	11492	12088	Bacteria	Proteobacteria	Alphaproteobacteria	Rhizobiales	Rhizobiaceae	Agrobacterium	Agrobacterium rubi
62512	9233	9599	8512	8009	11197	12153	11230	11998	Bacteria	Proteobacteria	Alphaproteobacteria	Rhizobiales	Rhizobiaceae	Agrobacterium	Agrobacterium rubi
61785	10018	8362	7624	7363	12201	11747	10922	11186	Bacteria	Proteobacteria	Alphaproteobacteria	Rhizobiales	Rhizobiaceae	Agrobacterium	unclassified
62086	10374	10941	10377	9103	11609	12527	11861	11989	Bacteria	Proteobacteria	Alphaproteobacteria	Rhizobiales	Rhizobiaceae	Candidatus Liberibacter	Candidatus Liberibacter africanus
61385	9866	10574	9769	9684	11798	12087	11050	11384	Bacteria	Proteobacteria	Alphaproteobacteria	Rhizobiales	Rhizobiaceae	Rhizobium	unclassified
61764	9415	8896	8474	8380	11282	12832	11333	11820	Bacteria	Proteobacteria	Alphaproteobacteria	Rhizobiales	Rhizobiaceae	Rhizobium	unclassified
61774	9413	10331	9450	8258	11697	12049	11005	11315	Bacteria	Proteobacteria	Alphaproteobacteria	Rhizobiales	Rhizobiaceae	Rhizobium	unclassified
61810	9682	9597	8885	8363	12392	12886	12059	12518	Bacteria	Proteobacteria	Alphaproteobacteria	Rhizobiales	Rhizobiaceae	Rhizobium	unclassified
61913	10296	11044	10539	9648	11714	12070	11035	11342	Bacteria	Proteobacteria	Alphaproteobacteria	Rhizobiales	Rhizobiaceae	Rhizobium	unclassified
62320	9866	10574	9769	9684	11798	12087	11050	11384	Bacteria	Proteobacteria	Alphaproteobacteria	Rhizobiales	Rhizobiaceae	Rhizobium	unclassified
62419	9141	9378	8652	8345	11639	11975	10887	11190	Bacteria	Proteobacteria	Alphaproteobacteria	Rhizobiales	Rhizobiaceae	Rhizobium	unclassified
62424	7892	9742	6485	7226	8696	12691	9165	9042	Bacteria	Proteobacteria	Alphaproteobacteria	Rhizobiales	Rhizobiaceae	Rhizobium	unclassified
62439	9793	9797	9122	8447	12255	12604	11511	11662	Bacteria	Proteobacteria	Alphaproteobacteria	Rhizobiales	Rhizobiaceae	Rhizobium	unclassified
62450	8052	10011	6447	6780	8851	13108	8533	8968	Bacteria	Proteobacteria	Alphaproteobacteria	Rhizobiales	Rhizobiaceae	Rhizobium	unclassified
62669	10134	10933	10331	9418	11762	12090	11277	11476	Bacteria	Proteobacteria	Alphaproteobacteria	Rhizobiales	Rhizobiaceae	Rhizobium	unclassified
62760	10038	10687	9942	9971	11979	12302	11403	11642	Bacteria	Proteobacteria	Alphaproteobacteria	Rhizobiales	Rhizobiaceae	Rhizobium	unclassified
62812	9098	9497	8654	8321	11641	11918	10850	11190	Bacteria	Proteobacteria	Alphaproteobacteria	Rhizobiales	Rhizobiaceae	Rhizobium	unclassified
61935	9215	8723	8253	8048	11484	12839	11622	11717	Bacteria	Proteobacteria	Alphaproteobacteria	Rhizobiales	Rhizobiaceae	Shinella	unclassified
59070	12274	12617	12730	11691	11908	12526	12588	12435	Bacteria	Proteobacteria	Alphaproteobacteria	Rhizobiales	unclassified	sfA	unclassified
59396	9222	9211	8851	8533	10741	11675	11279	10830	Bacteria	Proteobacteria	Alphaproteobacteria	Rhizobiales	unclassified	sfA	unclassified
61538	9091	8824	7530	7380	11176	11845	10370	10458	Bacteria	Proteobacteria	Alphaproteobacteria	Rhizobiales	unclassified	sfA	unclassified
62664	11267	10622	9254	9067	13138	13797	12985	13249	Bacteria	Proteobacteria	Alphaproteobacteria	Rhizobiales	unclassified	sfA	unclassified
59053	12658	12885	12593	11746	12833	12848	12959	13102	Bacteria	Proteobacteria	Alphaproteobacteria	Rhizobiales	Xanthobacteraceae	unclassified	unclassified
60560	9606	10109	9370	8901	11327	12119	11608	11375	Bacteria	Proteobacteria	Alphaproteobacteria	Rhodobacterales	Hyphomonadaceae	Oceanicaulis	unclassified
62391	9760	8784	8132	7902	11881	12725	11329	11974	Bacteria	Proteobacteria	Alphaproteobacteria	Rhodobacterales	Rhodobacteraceae	Nautella	Nautella italica
61909	9014	10083	6956	7455	12505	13285	13123	12986	Bacteria	Proteobacteria	Alphaproteobacteria	Rhodobacterales	Rhodobacteraceae	Paracoccus	Paracoccus aminovorans
62521	8311	8572	6890	7057	11233	12439	11849	11773	Bacteria	Proteobacteria	Alphaproteobacteria	Rhodobacterales	Rhodobacteraceae	Paracoccus	Paracoccus aminovorans
62769	10175	8673	8527	8391	12448	12804	12911	12723	Bacteria	Proteobacteria	Alphaproteobacteria	Rhodobacterales	Rhodobacteraceae	Paracoccus	Paracoccus aminovorans
62585	9632	9455	6016	6620	11960	12166	13139	12430	Bacteria	Proteobacteria	Alphaproteobacteria	Rhodobacterales	Rhodobacteraceae	Paracoccus	Paracoccus yeei
61965	10617	11843	9685	8555	13407	13793	13674	13689	Bacteria	Proteobacteria	Alphaproteobacteria	Rhodobacterales	Rhodobacteraceae	Paracoccus	unclassified
61984	8077	9312	6430	6440	10087	11875	11708	10446	Bacteria	Proteobacteria	Alphaproteobacteria	Rhodobacterales	Rhodobacteraceae	Paracoccus	unclassified
62220	9935	11663	9464	8276	12173	13365	12434	12455	Bacteria	Proteobacteria	Alphaproteobacteria	Rhodobacterales	Rhodobacteraceae	Paracoccus	unclassified
62280	10536	10962	9212	8164	12595	13206	12895	12986	Bacteria	Proteobacteria	Alphaproteobacteria	Rhodobacterales	Rhodobacteraceae	Paracoccus	unclassified
62348	10237	10553	9035	8738	12556	13252	13056	13096	Bacteria	Proteobacteria	Alphaproteobacteria	Rhodobacterales	Rhodobacteraceae	Paracoccus	unclassified
62371	11489	12227	10266	9743	13630	14032	13967	13909	Bacteria	Proteobacteria	Alphaproteobacteria	Rhodobacterales	Rhodobacteraceae	Paracoccus	unclassified
62476	9826	11466	7954	8198	12793	13629	13429	13240	Bacteria	Proteobacteria	Alphaproteobacteria	Rhodobacterales	Rhodobacteraceae	Paracoccus	unclassified
62719	8822	10018	8369	7308	11020	12383	12276	11769	Bacteria	Proteobacteria	Alphaproteobacteria	Rhodobacterales	Rhodobacteraceae	Paracoccus	unclassified
62734	8081	10406	7372	6825	11983	12990	13415	12829	Bacteria	Proteobacteria	Alphaproteobacteria	Rhodobacterales	Rhodobacteraceae	Paracoccus	unclassified
62890	9180	9178	7048	7726	11826	12707	12316	12228	Bacteria	Proteobacteria	Alphaproteobacteria	Rhodobacterales	Rhodobacteraceae	Paracoccus	unclassified
61473	8969	10318	6987	7551	12035	13260	12473	12978	Bacteria	Proteobacteria	Alphaproteobacteria	Rhodobacterales	Rhodobacteraceae	Rubellimicrobium	unclassified
61677	9858	10901	8984	8742	12936	13649	13251	13466	Bacteria	Proteobacteria	Alphaproteobacteria	Rhodobacterales	Rhodobacteraceae	Rubellimicrobium	unclassified
61835	10023	10875	10321	9813	12538	13447	12700	13260	Bacteria	Proteobacteria	Alphaproteobacteria	Rhodobacterales	Rhodobacteraceae	Rubellimicrobium	unclassified
62825	9421	9804	9009	8476	11590	12560	11989	12110	Bacteria	Proteobacteria	Alphaproteobacteria	Rhodobacterales	Rhodobacteraceae	Rubellimicrobium	unclassified
61317	10323	10842	9857	9233	12861	13378	12627	13039	Bacteria	Proteobacteria	Alphaproteobacteria	Rhodobacterales	Rhodobacteraceae	unclassified	unclassified
61612	9861	9951	8979	8499	11886	13008	11993	11814	Bacteria	Proteobacteria	Alphaproteobacteria	Rhodobacterales	Rhodobacteraceae	unclassified	unclassified
61699	8980	9958	8032	7970	11395	13320	11965	11784	Bacteria	Proteobacteria	Alphaproteobacteria	Rhodobacterales	Rhodobacteraceae	unclassified	unclassified
62019	9434	10259	9744	9503	10594	12797	11014	11541	Bacteria	Proteobacteria	Alphaproteobacteria	Rhodobacterales	Rhodobacteraceae	unclassified	unclassified
62417	9555	9809	9048	8089	11594	12631	11963	11802	Bacteria	Proteobacteria	Alphaproteobacteria	Rhodobacterales	Rhodobacteraceae	unclassified	unclassified
62496	11467	11756	11064	10270	12912	13498	13053	12996	Bacteria	Proteobacteria	Alphaproteobacteria	Rhodobacterales	Rhodobacteraceae	unclassified	unclassified
62578	11007	11694	10747	9311	13855	14397	13924	14119	Bacteria	Proteobacteria	Alphaproteobacteria	Rhodobacterales	Rhodobacteraceae	unclassified	unclassified
62724	9749	9251	8236	8575	10268	11676	10760	10775	Bacteria	Proteobacteria	Alphaproteobacteria	Rhodobacterales	Rhodobacteraceae	unclassified	unclassified
62790	9937	10327	9567	8672	11350	12321	11787	11956	Bacteria	Proteobacteria	Alphaproteobacteria	Rhodobacterales	Rhodobacteraceae	unclassified	unclassified
62853	10260	11131	9107	8661	12183	13384	12806	12607	Bacteria	Proteobacteria	Alphaproteobacteria	Rhodobacterales	Rhodobacteraceae	unclassified	unclassified
62927	10516	10230	8701	8790	11944	12379	11664	12181	Bacteria	Proteobacteria	Alphaproteobacteria	Rhodobacterales	Rhodobacteraceae	unclassified	unclassified
61082	8555	8361	6668	6917	10582	12415	11405	11731	Bacteria	Proteobacteria	Alphaproteobacteria	Rhodospirillales	Acetobacteraceae	Acidisphaera	unclassified
60231	7226	6209	6154	6338	10563	12048	11830	12125	Bacteria	Proteobacteria	Alphaproteobacteria	Rhodospirillales	Acetobacteraceae	Roseomonas	unclassified
60467	11200	11545	11200	10458	12874	13588	13189	13529	Bacteria	Proteobacteria	Alphaproteobacteria	Rhodospirillales	Acetobacteraceae	Roseomonas	unclassified
60475	10398	10239	9136	9642	12774	13166	12393	12906	Bacteria	Proteobacteria	Alphaproteobacteria	Rhodospirillales	Acetobacteraceae	Roseomonas	unclassified
60851	8302	8124	6846	6861	10858	11892	11141	11716	Bacteria	Proteobacteria	Alphaproteobacteria	Rhodospirillales	Acetobacteraceae	Roseomonas	unclassified
60378	9907	10826	7110	7854	12255	13265	12348	12990	Bacteria	Proteobacteria	Alphaproteobacteria	Rhodospirillales	Acetobacteraceae	unclassified	unclassified
60523	10205	11302	9155	10072	12746	13594	12990	13498	Bacteria	Proteobacteria	Alphaproteobacteria	Rhodospirillales	Acetobacteraceae	unclassified	unclassified
60926	10411	10001	8971	9865	11927	12743	12171	12435	Bacteria	Proteobacteria	Alphaproteobacteria	Rhodospirillales	Acetobacteraceae	unclassified	unclassified
59837	9150	9500	8416	8255	10893	12457	12843	12133	Bacteria	Proteobacteria	Alphaproteobacteria	Rhodospirillales	Rhodospirillaceae	Skermanella	unclassified
60105	8420	8341	8307	7942	10256	11626	11583	10125	Bacteria	Proteobacteria	Alphaproteobacteria	Rhodospirillales	Rhodospirillaceae	Skermanella	unclassified
59105	7892	6329	6183	6815	10686	11768	10662	10965	Bacteria	Proteobacteria	Alphaproteobacteria	Rhodospirillales	Rhodospirillaceae	unclassified	unclassified
59198	10478	11008	9927	9804	12406	13108	12520	12719	Bacteria	Proteobacteria	Alphaproteobacteria	Rhodospirillales	Rhodospirillaceae	unclassified	unclassified
59279	9816	9934	8796	8610	12408	13197	12770	12913	Bacteria	Proteobacteria	Alphaproteobacteria	Rhodospirillales	Rhodospirillaceae	unclassified	unclassified
59373	11808	12175	11997	11922	11979	12877	12802	12833	Bacteria	Proteobacteria	Alphaproteobacteria	Rhodospirillales	Rhodospirillaceae	unclassified	unclassified
59566	11775	12251	11815	11139	12867	13225	13006	13050	Bacteria	Proteobacteria	Alphaproteobacteria	Rhodospirillales	Rhodospirillaceae	unclassified	unclassified
59578	10346	10888	10142	8547	11155	11871	11535	11388	Bacteria	Proteobacteria	Alphaproteobacteria	Rhodospirillales	Rhodospirillaceae	unclassified	unclassified
59792	11793	12367	11865	11974	12448	13028	12642	12939	Bacteria	Proteobacteria	Alphaproteobacteria	Rhodospirillales	Rhodospirillaceae	unclassified	unclassified
60010	10493	10934	10160	9561	12948	12883	12283	12496	Bacteria	Proteobacteria	Alphaproteobacteria	Rhodospirillales	Rhodospirillaceae	unclassified	unclassified
60031	9179	9427	8682	7892	11342	12257	12180	12096	Bacteria	Proteobacteria	Alphaproteobacteria	Rhodospirillales	Rhodospirillaceae	unclassified	unclassified
60910	8976	9045	8965	8319	11334	12369	11415	11489	Bacteria	Proteobacteria	Alphaproteobacteria	Rhodospirillales	Rhodospirillaceae	unclassified	unclassified
61333	11425	11811	11297	10551	12957	13114	12567	12797	Bacteria	Proteobacteria	Alphaproteobacteria	Rhodospirillales	Rhodospirillaceae	unclassified	unclassified
73726	10435	10840	10147	9277	11766	12576	11764	11284	Bacteria	Proteobacteria	Alphaproteobacteria	Rickettsiales	Odyssellaceae	Candidatus Odyssella	unclassified
72592	9770	10181	9203	8813	11582	12526	12542	13179	Bacteria	Proteobacteria	Alphaproteobacteria	Rickettsiales	Rickettsiaceae	Rickettsia	Rickettsia akari
72956	9704	10393	8785	8305	11707	12508	12414	12587	Bacteria	Proteobacteria	Alphaproteobacteria	Rickettsiales	Rickettsiaceae	Rickettsia	Rickettsia akari
72061	9144	10855	8152	7782	12277	12726	12276	12242	Bacteria	Proteobacteria	Alphaproteobacteria	Rickettsiales	Rickettsiaceae	Rickettsia	Rickettsia australis
72329	8700	8677	7232	7061	9635	12322	11263	11990	Bacteria	Proteobacteria	Alphaproteobacteria	Rickettsiales	Rickettsiaceae	Rickettsia	unclassified
60565	8644	8708	7950	7998	11528	10662	9727	10268	Bacteria	Proteobacteria	Alphaproteobacteria	Sphingomonadales	Erythrobacteraceae	Erythrobacter	unclassified
60405	10694	11385	9089	10056	12019	12440	11187	11861	Bacteria	Proteobacteria	Alphaproteobacteria	Sphingomonadales	Erythrobacteraceae	unclassified	unclassified
61325	13566	13891	13641	13298	12411	13265	12702	12813	Bacteria	Proteobacteria	Alphaproteobacteria	Sphingomonadales	Erythrobacteraceae	unclassified	unclassified
60916	10975	11347	11103	9687	13024	12827	12609	12654	Bacteria	Proteobacteria	Alphaproteobacteria	Sphingomonadales	Sphingomonadaceae	Kaistobacter	unclassified
61102	10480	11774	10507	9847	12086	12149	11997	11951	Bacteria	Proteobacteria	Alphaproteobacteria	Sphingomonadales	Sphingomonadaceae	Novosphingobium	Novosphingobium resinovorum
60246	12224	12596	13023	11600	12231	12744	12352	12613	Bacteria	Proteobacteria	Alphaproteobacteria	Sphingomonadales	Sphingomonadaceae	Novosphingobium	unclassified
60346	10429	10924	10853	9944	11422	12340	11607	11814	Bacteria	Proteobacteria	Alphaproteobacteria	Sphingomonadales	Sphingomonadaceae	Novosphingobium	unclassified
60447	10757	10738	10414	11039	12411	12849	11765	12101	Bacteria	Proteobacteria	Alphaproteobacteria	Sphingomonadales	Sphingomonadaceae	Novosphingobium	unclassified
60608	11070	11087	10812	10613	12404	13025	12403	12870	Bacteria	Proteobacteria	Alphaproteobacteria	Sphingomonadales	Sphingomonadaceae	Novosphingobium	unclassified
60638	11608	12386	12199	11156	12794	13274	12556	12673	Bacteria	Proteobacteria	Alphaproteobacteria	Sphingomonadales	Sphingomonadaceae	Novosphingobium	unclassified
60769	8697	9244	8116	7359	11193	11805	10708	10810	Bacteria	Proteobacteria	Alphaproteobacteria	Sphingomonadales	Sphingomonadaceae	Novosphingobium	unclassified
60808	10853	11324	10518	8323	11919	12968	12505	13029	Bacteria	Proteobacteria	Alphaproteobacteria	Sphingomonadales	Sphingomonadaceae	Novosphingobium	unclassified
60837	10683	11080	11168	9721	10994	12344	11552	12397	Bacteria	Proteobacteria	Alphaproteobacteria	Sphingomonadales	Sphingomonadaceae	Novosphingobium	unclassified
60961	9275	12499	8185	10278	11593	12280	12312	12057	Bacteria	Proteobacteria	Alphaproteobacteria	Sphingomonadales	Sphingomonadaceae	Novosphingobium	unclassified
61046	12151	12473	12689	11575	12149	12653	12048	12312	Bacteria	Proteobacteria	Alphaproteobacteria	Sphingomonadales	Sphingomonadaceae	Novosphingobium	unclassified
61133	11151	12215	10984	10961	11011	12171	11702	11858	Bacteria	Proteobacteria	Alphaproteobacteria	Sphingomonadales	Sphingomonadaceae	Novosphingobium	unclassified

Table S6 (part R): HybScores of OTUs that were called present in at least one of the samples. Taxonomic classifications are based on PhyloChip taxonomy (derived from GreenGenes). Please note, that data presented here was rank normalized before performing statisical analyses presented in the manuscript.

	Sample ID (PMA)				Sample ID				taxonomic classification of representative rOTU cluster						
OTU	UR_PMA	CO_PMA	CR8_PMA	CR5_PMA	UR	CO	CR8	CR5	kingdom	phylum	class	order	family	genus	species
61355	12877	13259	13356	12117	12479	13031	12706	12932	Bacteria	Proteobacteria	Alphaproteobacteria	Sphingomonadales	Sphingomonadaceae	Novosphingobium	unclassified
60374	13062	13355	13056	12069	12373	12970	12635	12750	Bacteria	Proteobacteria	Alphaproteobacteria	Sphingomonadales	Sphingomonadaceae	Sphingobium	Sphingobium herbicidovorans
60291	12880	13118	12720	11911	11840	12364	12132	12316	Bacteria	Proteobacteria	Alphaproteobacteria	Sphingomonadales	Sphingomonadaceae	Sphingobium	unclassified
60301	10176	11383	9696	7758	11177	11764	11028	10942	Bacteria	Proteobacteria	Alphaproteobacteria	Sphingomonadales	Sphingomonadaceae	Sphingobium	unclassified
60332	11266	12147	10747	6519	11075	12106	11292	11284	Bacteria	Proteobacteria	Alphaproteobacteria	Sphingomonadales	Sphingomonadaceae	Sphingobium	unclassified
60395	12499	13261	12529	11341	12369	13047	12515	12175	Bacteria	Proteobacteria	Alphaproteobacteria	Sphingomonadales	Sphingomonadaceae	Sphingobium	unclassified
60421	11874	12517	12227	11362	12537	12312	12063	12049	Bacteria	Proteobacteria	Alphaproteobacteria	Sphingomonadales	Sphingomonadaceae	Sphingobium	unclassified
60538	9071	9885	8654	8002	12421	10261	9936	9818	Bacteria	Proteobacteria	Alphaproteobacteria	Sphingomonadales	Sphingomonadaceae	Sphingobium	unclassified
60557	9071	9885	8654	8002	12421	10261	9936	9818	Bacteria	Proteobacteria	Alphaproteobacteria	Sphingomonadales	Sphingomonadaceae	Sphingobium	unclassified
60579	11429	12306	11466	10119	9935	11327	11057	10472	Bacteria	Proteobacteria	Alphaproteobacteria	Sphingomonadales	Sphingomonadaceae	Sphingobium	unclassified
60732	12598	13065	12416	10570	11890	12581	12350	12601	Bacteria	Proteobacteria	Alphaproteobacteria	Sphingomonadales	Sphingomonadaceae	Sphingobium	unclassified
60822	9145	9935	8803	8090	12438	10610	10158	10191	Bacteria	Proteobacteria	Alphaproteobacteria	Sphingomonadales	Sphingomonadaceae	Sphingobium	unclassified
60902	11073	11444	11094	11033	12344	12367	12351	12367	Bacteria	Proteobacteria	Alphaproteobacteria	Sphingomonadales	Sphingomonadaceae	Sphingobium	unclassified
60970	10698	11765	10525	8540	12003	11854	11091	11002	Bacteria	Proteobacteria	Alphaproteobacteria	Sphingomonadales	Sphingomonadaceae	Sphingobium	unclassified
61013	11325	12153	10728	10325	13346	12640	10225	12707	Bacteria	Proteobacteria	Alphaproteobacteria	Sphingomonadales	Sphingomonadaceae	Sphingobium	unclassified
61101	10192	10759	10269	8609	12289	10940	10638	10555	Bacteria	Proteobacteria	Alphaproteobacteria	Sphingomonadales	Sphingomonadaceae	Sphingobium	unclassified
61127	11847	12292	11947	11199	9133	9903	10518	9613	Bacteria	Proteobacteria	Alphaproteobacteria	Sphingomonadales	Sphingomonadaceae	Sphingobium	unclassified
61290	9807	10526	9412	9495	12123	11746	10942	11108	Bacteria	Proteobacteria	Alphaproteobacteria	Sphingomonadales	Sphingomonadaceae	Sphingobium	unclassified
61302	11966	12837	11782	7916	11257	12170	11634	11570	Bacteria	Proteobacteria	Alphaproteobacteria	Sphingomonadales	Sphingomonadaceae	Sphingobium	unclassified
61327	11539	12426	11458	10134	10036	11424	11138	10531	Bacteria	Proteobacteria	Alphaproteobacteria	Sphingomonadales	Sphingomonadaceae	Sphingobium	unclassified
61060	12812	13070	12704	11866	9760	10822	11443	11087	Bacteria	Proteobacteria	Alphaproteobacteria	Sphingomonadales	Sphingomonadaceae	Sphingomonas	Caulobacter leidyia
60307	10028	10164	9841	8771	10874	11794	11201	11854	Bacteria	Proteobacteria	Alphaproteobacteria	Sphingomonadales	Sphingomonadaceae	Sphingomonas	Sphingomonas asaccharolytica
60205	10679	11166	10683	9743	11123	12649	12198	12112	Bacteria	Proteobacteria	Alphaproteobacteria	Sphingomonadales	Sphingomonadaceae	Sphingomonas	Sphingomonas wittichii
60278	10008	10199	9623	8429	10653	12120	11118	11193	Bacteria	Proteobacteria	Alphaproteobacteria	Sphingomonadales	Sphingomonadaceae	Sphingomonas	Sphingomonas wittichii
60430	10504	11757	10361	7583	11607	12346	11969	12287	Bacteria	Proteobacteria	Alphaproteobacteria	Sphingomonadales	Sphingomonadaceae	Sphingomonas	Sphingomonas wittichii
61106	9311	9785	8974	8432	11026	10491	12469	10135	Bacteria	Proteobacteria	Alphaproteobacteria	Sphingomonadales	Sphingomonadaceae	Sphingomonas	Sphingomonas yabuuchiae
60281	13838	13989	13995	12906	12800	12735	12911	12924	Bacteria	Proteobacteria	Alphaproteobacteria	Sphingomonadales	Sphingomonadaceae	Sphingomonas	unclassified
60315	12579	12921	12605	11656	11856	12300	12144	12253	Bacteria	Proteobacteria	Alphaproteobacteria	Sphingomonadales	Sphingomonadaceae	Sphingomonas	unclassified
60348	13051	13629	13170	11671	12467	13017	13125	13233	Bacteria	Proteobacteria	Alphaproteobacteria	Sphingomonadales	Sphingomonadaceae	Sphingomonas	unclassified
60351	10264	10803	10357	10110	11318	11741	11574	11829	Bacteria	Proteobacteria	Alphaproteobacteria	Sphingomonadales	Sphingomonadaceae	Sphingomonas	unclassified
60398	11714	12292	11936	11095	10559	11614	11334	11420	Bacteria	Proteobacteria	Alphaproteobacteria	Sphingomonadales	Sphingomonadaceae	Sphingomonas	unclassified
60449	11982	11914	11623	10543	9945	11943	10407	10690	Bacteria	Proteobacteria	Alphaproteobacteria	Sphingomonadales	Sphingomonadaceae	Sphingomonas	unclassified
60564	11341	11512	10574	9471	10826	11847	11188	11733	Bacteria	Proteobacteria	Alphaproteobacteria	Sphingomonadales	Sphingomonadaceae	Sphingomonas	unclassified
60568	12045	12338	11518	10185	11970	12659	12237	12471	Bacteria	Proteobacteria	Alphaproteobacteria	Sphingomonadales	Sphingomonadaceae	Sphingomonas	unclassified
60672	11883	12297	11818	11280	11688	12229	12029	12165	Bacteria	Proteobacteria	Alphaproteobacteria	Sphingomonadales	Sphingomonadaceae	Sphingomonas	unclassified
60758	12325	12538	12940	11644	11551	12211	12008	11962	Bacteria	Proteobacteria	Alphaproteobacteria	Sphingomonadales	Sphingomonadaceae	Sphingomonas	unclassified
60770	10773	11769	10673	9876	11206	12233	12131	12015	Bacteria	Proteobacteria	Alphaproteobacteria	Sphingomonadales	Sphingomonadaceae	Sphingomonas	unclassified
60826	11458	12131	11317	10606	12947	13037	12763	13019	Bacteria	Proteobacteria	Alphaproteobacteria	Sphingomonadales	Sphingomonadaceae	Sphingomonas	unclassified
60827	12841	13239	12876	11783	12012	12461	12350	12428	Bacteria	Proteobacteria	Alphaproteobacteria	Sphingomonadales	Sphingomonadaceae	Sphingomonas	unclassified
60858	12276	12665	12592	11902	10733	11849	11598	11623	Bacteria	Proteobacteria	Alphaproteobacteria	Sphingomonadales	Sphingomonadaceae	Sphingomonas	unclassified
60931	12067	12597	12252	11043	11272	12154	11918	12061	Bacteria	Proteobacteria	Alphaproteobacteria	Sphingomonadales	Sphingomonadaceae	Sphingomonas	unclassified
60966	11106	12025	11485	10370	11414	11670	12317	11815	Bacteria	Proteobacteria	Alphaproteobacteria	Sphingomonadales	Sphingomonadaceae	Sphingomonas	unclassified
61062	10792	11178	10170	9525	11677	12162	11843	11931	Bacteria	Proteobacteria	Alphaproteobacteria	Sphingomonadales	Sphingomonadaceae	Sphingomonas	unclassified
61087	11093	11916	10704	10070	13258	13145	12274	12907	Bacteria	Proteobacteria	Alphaproteobacteria	Sphingomonadales	Sphingomonadaceae	Sphingomonas	unclassified
61158	12838	13249	12898	11890	11513	12150	12066	12055	Bacteria	Proteobacteria	Alphaproteobacteria	Sphingomonadales	Sphingomonadaceae	Sphingomonas	unclassified
61191	12766	13072	12817	12264	11092	12449	12270	12206	Bacteria	Proteobacteria	Alphaproteobacteria	Sphingomonadales	Sphingomonadaceae	Sphingomonas	unclassified
61336	11224	10992	11202	9068	10964	12017	11367	11618	Bacteria	Proteobacteria	Alphaproteobacteria	Sphingomonadales	Sphingomonadaceae	Sphingomonas	unclassified
60697	10766	11163	10690	10178	11763	12064	12024	11699	Bacteria	Proteobacteria	Alphaproteobacteria	Sphingomonadales	Sphingomonadaceae	Sphingopyxis	unclassified
60279	10546	12072	10454	10856	12071	12675	12329	12783	Bacteria	Proteobacteria	Alphaproteobacteria	Sphingomonadales	Sphingomonadaceae	unclassified	unclassified
60492	10267	10427	9973	8931	11079	11714	10908	11362	Bacteria	Proteobacteria	Alphaproteobacteria	Sphingomonadales	Sphingomonadaceae	unclassified	unclassified
60840	10256	11084	9456	8329	10752	11828	10996	11304	Bacteria	Proteobacteria	Alphaproteobacteria	Sphingomonadales	Sphingomonadaceae	unclassified	unclassified
61094	14131	14427	14182	13481	13068	13558	13489	13517	Bacteria	Proteobacteria	Alphaproteobacteria	Sphingomonadales	Sphingomonadaceae	unclassified	unclassified
60741	8862	9615	8142	6934	11656	12505	12041	11932	Bacteria	Proteobacteria	Alphaproteobacteria	unclassified	unclassified	sfZ	unclassified
15566	10050	9352	8579	8127	12585	13134	12049	12282	Bacteria	Proteobacteria	Betaproteobacteria	Burkholderiales	Alcaligenaceae	unclassified	unclassified
10596	9909	10542	10045	8805	10166	12310	10976	10113	Bacteria	Proteobacteria	Betaproteobacteria	Burkholderiales	Aquabacteriaceae	Aquabacterium	unclassified
10657	11276	11833	11331	10177	12635	12765	12620	12406	Bacteria	Proteobacteria	Betaproteobacteria	Burkholderiales	Aquabacteriaceae	Aquabacterium	unclassified
10744	10204	6223	8039	6218	14178	12389	13124	12433	Bacteria	Proteobacteria	Betaproteobacteria	Burkholderiales	Aquabacteriaceae	Aquabacterium	unclassified
10760	9870	10499	10283	8250	12342	12733	11845	11533	Bacteria	Proteobacteria	Betaproteobacteria	Burkholderiales	Aquabacteriaceae	Aquabacterium	unclassified
10804	11521	12146	11997	11503	13821	13815	13657	13920	Bacteria	Proteobacteria	Betaproteobacteria	Burkholderiales	Aquabacteriaceae	Aquabacterium	unclassified
10851	10934	11392	11782	9402	11958	12397	12033	11714	Bacteria	Proteobacteria	Betaproteobacteria	Burkholderiales	Aquabacteriaceae	Aquabacterium	unclassified
10870	10044	9511	9467	8739	13439	12572	12642	12339	Bacteria	Proteobacteria	Betaproteobacteria	Burkholderiales	Aquabacteriaceae	Aquabacterium	unclassified
10995	10997	11227	11180	9833	11770	12305	11467	11281	Bacteria	Proteobacteria	Betaproteobacteria	Burkholderiales	Aquabacteriaceae	Aquabacterium	unclassified
11025	9773	10000	10210	8720	12890	12693	12282	12210	Bacteria	Proteobacteria	Betaproteobacteria	Burkholderiales	Aquabacteriaceae	Aquabacterium	unclassified
11137	11843	12670	12472	11252	13745	13779	13635	13471	Bacteria	Proteobacteria	Betaproteobacteria	Burkholderiales	Aquabacteriaceae	Aquabacterium	unclassified
11150	10555	11756	11506	10355	12918	13235	12923	13311	Bacteria	Proteobacteria	Betaproteobacteria	Burkholderiales	Aquabacteriaceae	Aquabacterium	unclassified
11204	11478	12193	11165	9342	14278	13581	13854	13757	Bacteria	Proteobacteria	Betaproteobacteria	Burkholderiales	Aquabacteriaceae	Aquabacterium	unclassified
11208	10602	11262	10883	8686	11589	12047	11094	11716	Bacteria	Proteobacteria	Betaproteobacteria	Burkholderiales	Aquabacteriaceae	Aquabacterium	unclassified
11318	11257	11495	11157	10439	12939	13008	12488	12753	Bacteria	Proteobacteria	Betaproteobacteria	Burkholderiales	Aquabacteriaceae	Aquabacterium	unclassified
11490	11349	11844	11645	11283	12960	12950	12969	13012	Bacteria	Proteobacteria	Betaproteobacteria	Burkholderiales	Aquabacteriaceae	Aquabacterium	unclassified
11543	11029	10891	10607	9190	12319	12699	12126	12695	Bacteria	Proteobacteria	Betaproteobacteria	Burkholderiales	Aquabacteriaceae	Aquabacterium	unclassified
11612	12224	12528	12828	10853	12056	12886	12635	12558	Bacteria	Proteobacteria	Betaproteobacteria	Burkholderiales	Aquabacteriaceae	Aquabacterium	unclassified
11636	10950	10664	10847	10541	12522	13283	12534	12890	Bacteria	Proteobacteria	Betaproteobacteria	Burkholderiales	Aquabacteriaceae	Aquabacterium	unclassified
11657	11515	11894	11410	9988	12724	13021	12706	13230	Bacteria	Proteobacteria	Betaproteobacteria	Burkholderiales	Aquabacteriaceae	Aquabacterium	unclassified
11724	11651	12194	12006	11064	12103	12904	12931	12715	Bacteria	Proteobacteria	Betaproteobacteria	Burkholderiales	Aquabacteriaceae	Aquabacterium	unclassified
11745	12200	12336	12263	11723	13585	13410	13034	13066	Bacteria	Proteobacteria	Betaproteobacteria	Burkholderiales	Aquabacteriaceae	Aquabacterium	unclassified
11757	13217	13437	13233	12057	14352	13739	13988	13910	Bacteria	Proteobacteria	Betaproteobacteria	Burkholderiales	Aquabacteriaceae	Aquabacterium	unclassified
11853	12849	13466	13156	12309	14280	14241	14404	14533	Bacteria	Proteobacteria	Betaproteobacteria	Burkholderiales	Aquabacteriaceae	Aquabacterium	unclassified
11897	9684	10340	10355	8069	12530	11821	11565	11312	Bacteria	Proteobacteria	Betaproteobacteria	Burkholderiales	Aquabacteriaceae	Aquabacterium	unclassified
11929	9808	9988	9526	8801	11556	11737	11195	11290	Bacteria	Proteobacteria	Betaproteobacteria	Burkholderiales	Aquabacteriaceae	Aquabacterium	unclassified
11964	13147	13289	12854	12545	14172	13739	13913	13901	Bacteria	Proteobacteria	Betaproteobacteria	Burkholderiales	Aquabacteriaceae	Aquabacterium	unclassified
11980	10248	10152	9593	9255	11607	12000	11221	11350	Bacteria	Proteobacteria	Betaproteobacteria	Burkholderiales	Aquabacteriaceae	Aquabacterium	unclassified
12094	12422	13115	13015	11449	12620	13336	12822	12890	Bacteria	Proteobacteria	Betaproteobacteria	Burkholderiales	Aquabacteriaceae	Aquabacterium	unclassified
12146	9690	11107	11075	9451	12210	12602	12123	12026	Bacteria	Proteobacteria	Betaproteobacteria	Burkholderiales	Aquabacteriaceae	Aquabacterium	unclassified
12175	9593	10346	9967	8683	12039	12449	11581	11892	Bacteria	Proteobacteria	Betaproteobacteria	Burkholderiales	Aquabacteriaceae	Aquabacterium	unclassified
12187	9362	9929	9184	8845	12209	12139	11584	11514	Bacteria	Proteobacteria	Betaproteobacteria	Burkholderiales	Aquabacteriaceae	Aquabacterium	unclassified
12211	11643	11457	11005	9648	11720	11876	11226	11668	Bacteria	Proteobacteria	Betaproteobacteria	Burkholderiales	Aquabacteriaceae	Aquabacterium	unclassified
12217	12341	12570	12329	11403	13629	13714	13125	13228	Bacteria	Proteobacteria	Betaproteobacteria	Burkholderiales	Aquabacteriaceae	Aquabacterium	unclassified
12333	9591	9448	10018	7594	11483	11991	11446	11334	Bacteria	Proteobacteria	Betaproteobacteria	Burkholderiales	Aquabacteriaceae	Aquabacterium	unclassified
12392	10999	11809	11799	10321	12649	13042	12634	12741	Bacteria	Proteobacteria	Betaproteobacteria	Burkholderiales	Aquabacteriaceae	Aquabacterium	unclassified
12476	10589	10346	10345	8908	13753	12837	13037	12909	Bacteria	Proteobacteria	Betaproteobacteria	Burkholderiales	Aquabacteriaceae	Aquabacterium	unclassified
12530	10110	10807	10684	9294	12781	13300	12429	12488	Bacteria	Proteobacteria	Betaproteobacteria	Burkholderiales	Aquabacteriaceae	Aquabacterium	unclassified
12654	10208	11309	11047	9838	12136	12380	12277	11928	Bacteria	Proteobacteria	Betaproteobacteria	Burkholderiales	Aquabacteriaceae	Aquabacterium	unclassified
12745	11225	11814	11701	8380	11903	12717	11821	12085	Bacteria	Proteobacteria	Betaproteobacteria	Burkholderiales	Aquabacteriaceae	Aquabacterium	unclassified
12809	9969	10572	8958	8551	12838	12774	12727	12990	Bacteria	Proteobacteria	Betaproteobacteria	Burkholderiales	Aquabacteriaceae	Aquabacterium	unclassified
12870	8370	7804	6954	7301	11643	12851	12117	12504	Bacteria	Proteobacteria	Betaproteobacteria	Burkholderiales	Aquabacteriaceae	Aquabacterium	unclassified
12882	10065	11289	11084	9920	11506	12049	11093	10917	Bacteria	Proteobacteria	Betaproteobacteria	Burkholderiales	Aquabacteriaceae	Aquabacterium	unclassified
12912	10469	11252	11299	9627	12325	12554	11870	11779	Bacteria	Proteobacteria	Betaproteobacteria	Burkholderiales	Aquabacteriaceae	Aquabacterium	unclassified
12928	10774	10588	10531	9577	11358	11758	11051	11057	Bacteria	Proteobacteria	Betaproteobacteria	Burkholderiales	Aquabacteriaceae	Aquabacterium	unclassified
13088	10950	11709	11542	9005	12759	12589	12289	12157	Bacteria	Proteobacteria	Betaproteobacteria	Burkholderiales	Aquabacteriaceae	Aquabacterium	unclassified
13092	9222	9691	9234	8673	11598	12017	11267	11223	Bacteria	Proteobacteria	Betaproteobacteria	Burkholderiales	Aquabacteriaceae	Aquabacterium	unclassified

Table S6 (part S): HybScores of OTUs that were called present in at least one of the samples. Taxonomic classifications are based on PhyloChip taxonomy (derived from GreenGenes). Please note, that data presented here was rank normalized before performing statisical analyses presented in the manuscript.

	Sample ID (PMA)				Sample ID				taxonomic classification of representative rOTU cluster						
OTU	UR_PMA	CO_PMA	CR8_PMA	CR5_PMA	UR	CO	CR8	CR5	kingdom	phylum	class	order	family	genus	species
13093	10679	11132	11390	10172	14038	13572	13237	12923	Bacteria	Proteobacteria	Betaproteobacteria	Burkholderiales	Aquabacteriaceae	Aquabacterium	unclassified
13106	9780	10416	10286	8545	11712	12186	11517	11405	Bacteria	Proteobacteria	Betaproteobacteria	Burkholderiales	Aquabacteriaceae	Aquabacterium	unclassified
13162	11290	11677	11061	10061	13392	13521	13191	13540	Bacteria	Proteobacteria	Betaproteobacteria	Burkholderiales	Aquabacteriaceae	Aquabacterium	unclassified
13274	11147	10822	10875	9811	12284	12808	12256	12451	Bacteria	Proteobacteria	Betaproteobacteria	Burkholderiales	Aquabacteriaceae	Aquabacterium	unclassified
13362	9118	8838	8339	7324	10866	11753	10913	10906	Bacteria	Proteobacteria	Betaproteobacteria	Burkholderiales	Aquabacteriaceae	Aquabacterium	unclassified
13400	10861	11243	10654	9443	12645	13070	12774	13059	Bacteria	Proteobacteria	Betaproteobacteria	Burkholderiales	Aquabacteriaceae	Aquabacterium	unclassified
13402	11375	12552	12465	10823	13353	13360	13124	13007	Bacteria	Proteobacteria	Betaproteobacteria	Burkholderiales	Aquabacteriaceae	Aquabacterium	unclassified
13681	8524	8606	8330	7115	11119	12507	11532	12200	Bacteria	Proteobacteria	Betaproteobacteria	Burkholderiales	Aquabacteriaceae	Aquabacterium	unclassified
13763	11764	11895	12397	10917	11995	12541	12079	12221	Bacteria	Proteobacteria	Betaproteobacteria	Burkholderiales	Aquabacteriaceae	Aquabacterium	unclassified
13769	10851	11523	11294	10578	13434	12862	12881	12846	Bacteria	Proteobacteria	Betaproteobacteria	Burkholderiales	Aquabacteriaceae	Aquabacterium	unclassified
13882	11091	11659	11663	10412	12030	12174	11616	11566	Bacteria	Proteobacteria	Betaproteobacteria	Burkholderiales	Aquabacteriaceae	Aquabacterium	unclassified
13991	11895	12197	12247	11283	12202	12696	12708	12519	Bacteria	Proteobacteria	Betaproteobacteria	Burkholderiales	Aquabacteriaceae	Aquabacterium	unclassified
14055	11078	11532	11504	10426	13557	13037	13029	12804	Bacteria	Proteobacteria	Betaproteobacteria	Burkholderiales	Aquabacteriaceae	Aquabacterium	unclassified
14121	12871	13193	13597	12260	12032	12480	12523	12499	Bacteria	Proteobacteria	Betaproteobacteria	Burkholderiales	Aquabacteriaceae	Aquabacterium	unclassified
14137	10766	11378	11444	9305	12442	12926	12447	11810	Bacteria	Proteobacteria	Betaproteobacteria	Burkholderiales	Aquabacteriaceae	Aquabacterium	unclassified
14183	11037	11292	11260	11093	13096	13172	12913	13327	Bacteria	Proteobacteria	Betaproteobacteria	Burkholderiales	Aquabacteriaceae	Aquabacterium	unclassified
14194	11527	12055	12314	9935	12406	12894	12634	12807	Bacteria	Proteobacteria	Betaproteobacteria	Burkholderiales	Aquabacteriaceae	Aquabacterium	unclassified
14199	11341	11424	10370	10105	12650	12840	12391	13024	Bacteria	Proteobacteria	Betaproteobacteria	Burkholderiales	Aquabacteriaceae	Aquabacterium	unclassified
14292	11684	12095	12232	10404	11671	12474	11570	11588	Bacteria	Proteobacteria	Betaproteobacteria	Burkholderiales	Aquabacteriaceae	Aquabacterium	unclassified
15292	10770	11130	10414	8500	13212	13505	12519	12694	Bacteria	Proteobacteria	Betaproteobacteria	Burkholderiales	Burkholderiaceae	Burkholderia	Burkholderia mallei
14622	9142	10655	8648	8442	11122	12390	11135	11014	Bacteria	Proteobacteria	Betaproteobacteria	Burkholderiales	Burkholderiaceae	Burkholderia	unclassified
14477	10028	10235	9978	9382	12871	12741	12274	12257	Bacteria	Proteobacteria	Betaproteobacteria	Burkholderiales	Burkholderiaceae	Pandoraea	unclassified
14545	10855	11529	11418	9665	12233	12844	12124	12214	Bacteria	Proteobacteria	Betaproteobacteria	Burkholderiales	Burkholderiaceae	Pandoraea	unclassified
15621	8032	10390	6781	7092	9798	12073	9350	9362	Bacteria	Proteobacteria	Betaproteobacteria	Burkholderiales	Burkholderiaceae	unclassified	unclassified
17051	11852	12311	12222	11420	12867	12398	12166	11939	Bacteria	Proteobacteria	Betaproteobacteria	Burkholderiales	Comamonadaceae	Comamonas	unclassified
8808	9887	10520	9724	8763	11905	11890	11601	11471	Bacteria	Proteobacteria	Betaproteobacteria	Burkholderiales	Comamonadaceae	Delftia	unclassified
10173	11339	11943	11315	10324	13000	13212	12437	12574	Bacteria	Proteobacteria	Betaproteobacteria	Burkholderiales	Comamonadaceae	Diaphorobacter	unclassified
10400	10526	11498	10719	10249	12916	12920	12726	12561	Bacteria	Proteobacteria	Betaproteobacteria	Burkholderiales	Comamonadaceae	Diaphorobacter	unclassified
10409	10975	11508	11273	10598	12169	12949	12246	12145	Bacteria	Proteobacteria	Betaproteobacteria	Burkholderiales	Comamonadaceae	Diaphorobacter	unclassified
7690	11331	11916	11898	10510	11753	12630	12225	12221	Bacteria	Proteobacteria	Betaproteobacteria	Burkholderiales	Comamonadaceae	Diaphorobacter	unclassified
7871	10334	11191	10906	9859	12775	12625	12339	12549	Bacteria	Proteobacteria	Betaproteobacteria	Burkholderiales	Comamonadaceae	Diaphorobacter	unclassified
8038	10831	11871	10164	9275	13426	13324	12896	13008	Bacteria	Proteobacteria	Betaproteobacteria	Burkholderiales	Comamonadaceae	Diaphorobacter	unclassified
8070	11914	12447	11526	11088	13475	13356	12653	13052	Bacteria	Proteobacteria	Betaproteobacteria	Burkholderiales	Comamonadaceae	Diaphorobacter	unclassified
8071	12228	12950	12364	11883	13875	13457	13323	13711	Bacteria	Proteobacteria	Betaproteobacteria	Burkholderiales	Comamonadaceae	Diaphorobacter	unclassified
8086	13681	13833	13699	13068	14399	14027	14018	14270	Bacteria	Proteobacteria	Betaproteobacteria	Burkholderiales	Comamonadaceae	Diaphorobacter	unclassified
8217	12207	12738	12490	11536	12879	13162	12928	12893	Bacteria	Proteobacteria	Betaproteobacteria	Burkholderiales	Comamonadaceae	Diaphorobacter	unclassified
8247	12449	12675	12590	11802	13419	13345	13453	13471	Bacteria	Proteobacteria	Betaproteobacteria	Burkholderiales	Comamonadaceae	Diaphorobacter	unclassified
8249	8510	7363	7023	7032	12712	9990	8752	9499	Bacteria	Proteobacteria	Betaproteobacteria	Burkholderiales	Comamonadaceae	Diaphorobacter	unclassified
8300	11457	12225	11806	10870	12581	13253	13178	13013	Bacteria	Proteobacteria	Betaproteobacteria	Burkholderiales	Comamonadaceae	Diaphorobacter	unclassified
8316	10352	11131	10070	8809	13341	12966	12351	12507	Bacteria	Proteobacteria	Betaproteobacteria	Burkholderiales	Comamonadaceae	Diaphorobacter	unclassified
8385	12422	13005	12424	11926	13743	13501	12717	13131	Bacteria	Proteobacteria	Betaproteobacteria	Burkholderiales	Comamonadaceae	Diaphorobacter	unclassified
8437	10560	11184	9536	8067	13169	12923	12023	12497	Bacteria	Proteobacteria	Betaproteobacteria	Burkholderiales	Comamonadaceae	Diaphorobacter	unclassified
8529	9611	10559	9819	7727	13273	12630	12536	12452	Bacteria	Proteobacteria	Betaproteobacteria	Burkholderiales	Comamonadaceae	Diaphorobacter	unclassified
8546	11206	11761	11361	10521	12946	12935	12733	12521	Bacteria	Proteobacteria	Betaproteobacteria	Burkholderiales	Comamonadaceae	Diaphorobacter	unclassified
8593	10669	10952	9599	9659	12006	12074	11565	11893	Bacteria	Proteobacteria	Betaproteobacteria	Burkholderiales	Comamonadaceae	Diaphorobacter	unclassified
8663	9837	9323	9204	8709	12505	12180	11573	11692	Bacteria	Proteobacteria	Betaproteobacteria	Burkholderiales	Comamonadaceae	Diaphorobacter	unclassified
8842	11890	11991	12167	12307	12725	12439	11984	12060	Bacteria	Proteobacteria	Betaproteobacteria	Burkholderiales	Comamonadaceae	Diaphorobacter	unclassified
8845	11710	11981	10921	10872	13940	13528	12411	13055	Bacteria	Proteobacteria	Betaproteobacteria	Burkholderiales	Comamonadaceae	Diaphorobacter	unclassified
8983	9989	10863	9102	8309	13070	12672	11840	12344	Bacteria	Proteobacteria	Betaproteobacteria	Burkholderiales	Comamonadaceae	Diaphorobacter	unclassified
9182	10678	11636	9407	8922	13753	13358	12656	12961	Bacteria	Proteobacteria	Betaproteobacteria	Burkholderiales	Comamonadaceae	Diaphorobacter	unclassified
9253	10819	11734	11742	9943	12017	12240	11752	11765	Bacteria	Proteobacteria	Betaproteobacteria	Burkholderiales	Comamonadaceae	Diaphorobacter	unclassified
9380	9937	10699	8792	7966	13321	12967	12110	12526	Bacteria	Proteobacteria	Betaproteobacteria	Burkholderiales	Comamonadaceae	Diaphorobacter	unclassified
9418	10628	10814	10510	9038	12868	13016	12686	12755	Bacteria	Proteobacteria	Betaproteobacteria	Burkholderiales	Comamonadaceae	Diaphorobacter	unclassified
9474	11516	11932	11626	10289	13517	13475	13365	13810	Bacteria	Proteobacteria	Betaproteobacteria	Burkholderiales	Comamonadaceae	Diaphorobacter	unclassified
9571	10035	10223	9677	7696	12990	13190	12984	12972	Bacteria	Proteobacteria	Betaproteobacteria	Burkholderiales	Comamonadaceae	Diaphorobacter	unclassified
9579	10626	11467	11013	10387	11603	12439	11964	12060	Bacteria	Proteobacteria	Betaproteobacteria	Burkholderiales	Comamonadaceae	Diaphorobacter	unclassified
9615	12440	12675	12633	11555	12851	12979	12942	12884	Bacteria	Proteobacteria	Betaproteobacteria	Burkholderiales	Comamonadaceae	Diaphorobacter	unclassified
9623	9984	10725	9562	8446	12891	12772	11899	12160	Bacteria	Proteobacteria	Betaproteobacteria	Burkholderiales	Comamonadaceae	Diaphorobacter	unclassified
9742	10133	11020	9549	8347	13245	12943	12152	12549	Bacteria	Proteobacteria	Betaproteobacteria	Burkholderiales	Comamonadaceae	Diaphorobacter	unclassified
9872	12391	12948	12718	12350	12987	12633	12613	13193	Bacteria	Proteobacteria	Betaproteobacteria	Burkholderiales	Comamonadaceae	Diaphorobacter	unclassified
9931	10306	10595	10938	9184	12580	12237	11630	12785	Bacteria	Proteobacteria	Betaproteobacteria	Burkholderiales	Comamonadaceae	Diaphorobacter	unclassified
9938	14026	14223	14082	13524	14039	13589	12942	13817	Bacteria	Proteobacteria	Betaproteobacteria	Burkholderiales	Comamonadaceae	Diaphorobacter	unclassified
9917	10139	10524	10470	9238	11007	11453	11769	11257	Bacteria	Proteobacteria	Betaproteobacteria	Burkholderiales	Comamonadaceae	Polaromonas	unclassified
9103	12200	11818	11400	9662	12764	12759	11857	11944	Bacteria	Proteobacteria	Betaproteobacteria	Burkholderiales	Comamonadaceae	Rhodoferax	unclassified
9232	12040	12659	12248	11325	11832	12743	12212	12206	Bacteria	Proteobacteria	Betaproteobacteria	Burkholderiales	Comamonadaceae	Rhodoferax	unclassified
9921	9711	9828	10101	9412	12107	12156	11458	12480	Bacteria	Proteobacteria	Betaproteobacteria	Burkholderiales	Comamonadaceae	Rhodoferax	unclassified
9359	9080	10002	8590	9720	9141	12169	8810	8992	Bacteria	Proteobacteria	Betaproteobacteria	Burkholderiales	Comamonadaceae	Simplicispira	unclassified
10116	11354	12297	12059	11232	10860	12142	11716	11279	Bacteria	Proteobacteria	Betaproteobacteria	Burkholderiales	Comamonadaceae	unclassified	unclassified
10362	11703	12197	12653	11001	11364	11437	11480	11240	Bacteria	Proteobacteria	Betaproteobacteria	Burkholderiales	Comamonadaceae	unclassified	unclassified
15978	9069	8621	7974	7776	9958	11716	10334	10137	Bacteria	Proteobacteria	Betaproteobacteria	Burkholderiales	Comamonadaceae	unclassified	unclassified
16384	11943	12412	12169	11581	12600	12538	12601	12506	Bacteria	Proteobacteria	Betaproteobacteria	Burkholderiales	Comamonadaceae	unclassified	unclassified
7636	13350	13592	13613	12253	11988	12382	12962	12816	Bacteria	Proteobacteria	Betaproteobacteria	Burkholderiales	Comamonadaceae	unclassified	unclassified
7682	12641	13023	12870	12384	13179	13311	13399	13393	Bacteria	Proteobacteria	Betaproteobacteria	Burkholderiales	Comamonadaceae	unclassified	unclassified
7917	9651	9847	9864	8768	10998	12216	10812	11236	Bacteria	Proteobacteria	Betaproteobacteria	Burkholderiales	Comamonadaceae	unclassified	unclassified
8368	12129	12449	12427	11144	12049	12493	12196	12088	Bacteria	Proteobacteria	Betaproteobacteria	Burkholderiales	Comamonadaceae	unclassified	unclassified
9071	11791	12533	12133	10822	11871	13015	12387	12057	Bacteria	Proteobacteria	Betaproteobacteria	Burkholderiales	Comamonadaceae	unclassified	unclassified
9082	12344	12746	12341	11589	13089	13024	13039	13019	Bacteria	Proteobacteria	Betaproteobacteria	Burkholderiales	Comamonadaceae	unclassified	unclassified
9140	10791	11070	11571	9791	11273	12197	11502	11754	Bacteria	Proteobacteria	Betaproteobacteria	Burkholderiales	Comamonadaceae	unclassified	unclassified
9425	11946	12336	12368	10792	11118	11925	11743	11364	Bacteria	Proteobacteria	Betaproteobacteria	Burkholderiales	Comamonadaceae	unclassified	unclassified
9681	9224	9203	8970	8299	10653	12275	11203	10193	Bacteria	Proteobacteria	Betaproteobacteria	Burkholderiales	Comamonadaceae	unclassified	unclassified
10344	13820	14123	14180	12923	12130	12514	13227	13130	Bacteria	Proteobacteria	Betaproteobacteria	Burkholderiales	Comamonadaceae	Variovorax	Variovorax paradoxus
7833	11904	12440	12465	10505	11909	12190	12497	12329	Bacteria	Proteobacteria	Betaproteobacteria	Burkholderiales	Comamonadaceae	Variovorax	Variovorax paradoxus
9427	11709	12251	12207	11475	12755	12234	11970	12082	Bacteria	Proteobacteria	Betaproteobacteria	Burkholderiales	Comamonadaceae	Verminephrobacter	Verminephrobacter eiseniae
9619	11253	11578	11637	9865	10204	11077	10429	10384	Bacteria	Proteobacteria	Betaproteobacteria	Burkholderiales	Comamonadaceae	Verminephrobacter	Verminephrobacter eiseniae
10737	9098	8264	7608	7687	11787	11758	10649	11060	Bacteria	Proteobacteria	Betaproteobacteria	Burkholderiales	Methylibiaceae	Methylibium	unclassified
12934	9256	9140	8021	8146	11477	11996	11043	11096	Bacteria	Proteobacteria	Betaproteobacteria	Burkholderiales	Methylibiaceae	Methylibium	unclassified
13091	8650	9887	7996	7580	11831	12582	10257	10992	Bacteria	Proteobacteria	Betaproteobacteria	Burkholderiales	Methylibiaceae	Methylibium	unclassified
13122	8383	8502	7473	7607	11105	12581	10176	10521	Bacteria	Proteobacteria	Betaproteobacteria	Burkholderiales	Methylibiaceae	Methylibium	unclassified
14597	10544	8346	8989	8508	13941	12687	13266	12807	Bacteria	Proteobacteria	Betaproteobacteria	Burkholderiales	Oxalobacteraceae	Massilia	Massilia timonae
14599	10583	6049	8114	6220	13755	11505	11073	10550	Bacteria	Proteobacteria	Betaproteobacteria	Burkholderiales	Oxalobacteraceae	Massilia	Massilia timonae
14641	11461	11859	11719	10982	12799	12564	12678	12722	Bacteria	Proteobacteria	Betaproteobacteria	Burkholderiales	Oxalobacteraceae	Massilia	Massilia timonae
14754	9604	7077	7765	6683	13408	12135	12870	12214	Bacteria	Proteobacteria	Betaproteobacteria	Burkholderiales	Oxalobacteraceae	Massilia	Massilia timonae
14779	11152	9701	10123	7441	14088	12798	13275	12890	Bacteria	Proteobacteria	Betaproteobacteria	Burkholderiales	Oxalobacteraceae	Massilia	Massilia timonae
15035	9832	8378	9583	7307	13022	12179	12532	12309	Bacteria	Proteobacteria	Betaproteobacteria	Burkholderiales	Oxalobacteraceae	Massilia	Massilia timonae
15249	11090	9401	9297	8404	13637	11474	11714	10906	Bacteria	Proteobacteria	Betaproteobacteria	Burkholderiales	Oxalobacteraceae	Massilia	Massilia timonae
15342	10799	9661	9197	8381	13951	11933	12373	11410	Bacteria	Proteobacteria	Betaproteobacteria	Burkholderiales	Oxalobacteraceae	Massilia	Massilia timonae
15570	8696	6847	8610	6582	12168	11688	11656	11919	Bacteria	Proteobacteria	Betaproteobacteria	Burkholderiales	Oxalobacteraceae	Massilia	Massilia timonae
15647	10454	10164	9523	8553	13004	11913	11763	12275	Bacteria	Proteobacteria	Betaproteobacteria	Burkholderiales	Oxalobacteraceae	Massilia	Massilia timonae
15685	11866	11069	11174	9987	14628	13553	13938	13705	Bacteria	Proteobacteria	Betaproteobacteria	Burkholderiales	Oxalobacteraceae	Massilia	Massilia timonae
15719	9300	9652	8934	8439	11828	12076	11175	11455	Bacteria	Proteobacteria	Betaproteobacteria	Burkholderiales	Oxalobacteraceae	Massilia	Massilia timonae

Table S6 (part T): HybScores of OTUs that were called present in at least one of the samples. Taxonomic classifications are based on PhyloChip taxonomy (derived from GreenGenes). Please note, that data presented here was rank normalized before performing statisical analyses presented in the manuscript.

	Sample ID (PMA)				Sample ID				taxonomic classification of representative rOTU cluster						
OTU	UR_PMA	CO_PMA	CR8_PMA	CR5_PMA	UR	CO	CR8	CR5	kingdom	phylum	class	order	family	genus	species
14633	8541	8899	7248	7275	11705	11791	11401	11605	Bacteria	Proteobacteria	Betaproteobacteria	Burkholderiales	Oxalobacteraceae	Oxalobacter	unclassified
15016	11475	11603	11358	10198	12172	12224	11841	11651	Bacteria	Proteobacteria	Betaproteobacteria	Burkholderiales	Oxalobacteraceae	unclassified	unclassified
15459	8774	7866	7668	7335	11313	12157	13207	11626	Bacteria	Proteobacteria	Betaproteobacteria	Burkholderiales	Oxalobacteraceae	unclassified	unclassified
15508	9008	8546	8287	7944	11102	11372	12327	11068	Bacteria	Proteobacteria	Betaproteobacteria	Burkholderiales	Oxalobacteraceae	unclassified	unclassified
15533	9150	7426	7632	7081	11403	11909	12038	11504	Bacteria	Proteobacteria	Betaproteobacteria	Burkholderiales	Oxalobacteraceae	unclassified	unclassified
15581	9391	7951	7846	7571	12010	12883	12914	12476	Bacteria	Proteobacteria	Betaproteobacteria	Burkholderiales	Oxalobacteraceae	unclassified	unclassified
15605	11371	11215	11314	9888	13061	11659	12317	11535	Bacteria	Proteobacteria	Betaproteobacteria	Burkholderiales	Oxalobacteraceae	unclassified	unclassified
15679	8870	8261	6106	8040	10354	11703	11491	10215	Bacteria	Proteobacteria	Betaproteobacteria	Burkholderiales	Oxalobacteraceae	unclassified	unclassified
15731	9917	7135	8138	6667	12029	12129	12091	12079	Bacteria	Proteobacteria	Betaproteobacteria	Burkholderiales	Oxalobacteraceae	unclassified	unclassified
15831	7489	6038	6135	7218	10379	12183	11642	11588	Bacteria	Proteobacteria	Betaproteobacteria	Burkholderiales	Oxalobacteraceae	unclassified	unclassified
14831	8965	9063	8361	8850	11916	13172	11869	12293	Bacteria	Proteobacteria	Betaproteobacteria	Nitrosomonadales	Nitrosomonadaceae	unclassified	unclassified
15127	8540	7449	6230	6825	9077	12926	8743	8896	Bacteria	Proteobacteria	Betaproteobacteria	Nitrosomonadales	Nitrosomonadaceae	unclassified	unclassified
15495	12796	13230	13001	11209	13313	13666	13191	13734	Bacteria	Proteobacteria	Betaproteobacteria	Nitrosomonadales	Nitrosomonadaceae	unclassified	unclassified
16687	7902	6668	6211	6716	8866	13050	9008	8772	Bacteria	Proteobacteria	Betaproteobacteria	Rhodocyclales	Rhodocyclaceae	Thauera	unclassified
16937	7950	7728	6647	7038	8853	13983	8960	9335	Bacteria	Proteobacteria	Betaproteobacteria	Rhodocyclales	Rhodocyclaceae	unclassified	unclassified
14678	9899	9845	9482	8047	11741	12544	12037	11943	Bacteria	Proteobacteria	Betaproteobacteria	unclassified	unclassified	sfA	unclassified
58369	8701	8861	8125	7767	11209	12294	11494	11801	Bacteria	Proteobacteria	Deltaproteobacteria	Desulfobacterales	Nitrospinaceae	unclassified	unclassified
57341	9055	9368	8508	7652	12283	12615	12354	12581	Bacteria	Proteobacteria	Deltaproteobacteria	Desulfovibrionales	Desulfovibrionaceae	Desulfovibrio	Desulfovibrio putealis
58435	13199	13602	13509	12926	14042	13854	13621	13673	Bacteria	Proteobacteria	Deltaproteobacteria	Desulfuromonadales	Desulfuromonadaceae	unclassified	unclassified
58114	8143	8984	6726	6653	10210	11983	11511	11448	Bacteria	Proteobacteria	Deltaproteobacteria	Myxococcales	Cystobacteraceae	Archangium	Cystobacter minus
58446	8281	9135	6746	6742	10407	12192	11730	11733	Bacteria	Proteobacteria	Deltaproteobacteria	Myxococcales	Cystobacteraceae	Archangium	Cystobacter minus
58691	7623	7718	6347	6621	9778	11751	11362	11133	Bacteria	Proteobacteria	Deltaproteobacteria	Myxococcales	Cystobacteraceae	Archangium	unclassified
51929	8437	8829	7255	7081	10518	11941	11052	11093	Bacteria	Proteobacteria	Deltaproteobacteria	Myxococcales	Haliangiaceae	unclassified	unclassified
51982	12664	13225	12606	12043	13431	13554	13397	13192	Bacteria	Proteobacteria	Deltaproteobacteria	Myxococcales	Haliangiaceae	unclassified	unclassified
52142	9016	10110	8834	7640	11555	12700	12072	11951	Bacteria	Proteobacteria	Deltaproteobacteria	Myxococcales	Haliangiaceae	unclassified	unclassified
52401	10121	10638	11409	10501	11186	12497	12393	11655	Bacteria	Proteobacteria	Deltaproteobacteria	Myxococcales	Haliangiaceae	unclassified	unclassified
58556	9565	9354	9177	8542	10140	11866	11168	11175	Bacteria	Proteobacteria	Deltaproteobacteria	Myxococcales	Myxococcaceae	Anaeromyxobacter	Anaeromyxobacter sp. Fw109-5
58706	8730	8604	6799	7420	11182	12298	11611	11610	Bacteria	Proteobacteria	Deltaproteobacteria	Myxococcales	Myxococcaceae	Myxococcus	unclassified
52843	9344	9466	7603	7837	12346	13056	12482	12565	Bacteria	Proteobacteria	Deltaproteobacteria	Myxococcales	unclassified	sfD	unclassified
52737	8041	6790	6354	6646	10913	11812	10368	11379	Bacteria	Proteobacteria	Deltaproteobacteria	Myxococcales	unclassified	sfE	unclassified
52703	8358	7775	6935	7456	11707	12250	11523	11872	Bacteria	Proteobacteria	Deltaproteobacteria	Syntrophobacterales	Desulfobacteraceae	Desulfobacterium	unclassified
52380	8102	7716	7621	6826	10664	11136	11769	12185	Bacteria	Proteobacteria	Deltaproteobacteria	Syntrophobacterales	Syntrophaceae	Syntrophus	unclassified
51890	9817	9755	8728	7805	14006	13577	13778	13770	Bacteria	Proteobacteria	Deltaproteobacteria	Syntrophobacterales	Syntrophaceae	unclassified	unclassified
52341	9227	8782	7713	7956	12002	12638	12251	12321	Bacteria	Proteobacteria	Deltaproteobacteria	unclassified	unclassified	sfAT	unclassified
52457	8567	7777	6784	7273	10899	12155	11167	11587	Bacteria	Proteobacteria	Deltaproteobacteria	unclassified	unclassified	sfC	unclassified
51836	11297	11857	11439	10348	12713	13386	12934	13333	Bacteria	Proteobacteria	Deltaproteobacteria	unclassified	unclassified	sfF	unclassified
16065	9035	8923	7965	7124	11730	12179	11420	11792	Bacteria	Proteobacteria	Gammaproteobacteria	Aeromonadales	Succinivibrionaceae	Succinivibrio	unclassified
2022	11435	10948	10577	9049	12233	12773	11733	11948	Bacteria	Proteobacteria	Gammaproteobacteria	Chromatiales	Chromatiaceae	unclassified	unclassified
6203	10972	11124	10863	10592	11430	11668	11273	11896	Bacteria	Proteobacteria	Gammaproteobacteria	Enterobacteriales	Enterobacteriaceae	Citrobacter	unclassified
4998	9168	9038	8757	7179	11221	11560	12091	11749	Bacteria	Proteobacteria	Gammaproteobacteria	Enterobacteriales	Enterobacteriaceae	Klebsiella	unclassified
5687	8897	8944	8480	7819	10813	11697	11624	11968	Bacteria	Proteobacteria	Gammaproteobacteria	Enterobacteriales	Enterobacteriaceae	Pantoea	unclassified
6094	8988	9013	7545	7854	10423	11487	11888	12149	Bacteria	Proteobacteria	Gammaproteobacteria	Enterobacteriales	Enterobacteriaceae	Pantoea	unclassified
6129	9913	10498	9496	9194	11790	12160	11963	12462	Bacteria	Proteobacteria	Gammaproteobacteria	Enterobacteriales	Enterobacteriaceae	Pantoea	unclassified
4273	9674	9527	7833	8627	12884	12331	12637	12449	Bacteria	Proteobacteria	Gammaproteobacteria	Enterobacteriales	Enterobacteriaceae	unclassified	unclassified
4421	8378	8052	6579	7591	10213	11071	11319	11876	Bacteria	Proteobacteria	Gammaproteobacteria	Enterobacteriales	Enterobacteriaceae	unclassified	unclassified
4467	8389	8053	7536	7501	10762	11201	11526	11825	Bacteria	Proteobacteria	Gammaproteobacteria	Enterobacteriales	Enterobacteriaceae	unclassified	unclassified
4497	9980	11807	8366	8762	11418	12861	11327	11830	Bacteria	Proteobacteria	Gammaproteobacteria	Enterobacteriales	Enterobacteriaceae	unclassified	unclassified
5272	11034	10992	10639	10019	11970	12449	11874	12323	Bacteria	Proteobacteria	Gammaproteobacteria	Enterobacteriales	Enterobacteriaceae	unclassified	unclassified
5538	10425	11286	10777	8523	12798	13273	13290	13377	Bacteria	Proteobacteria	Gammaproteobacteria	Enterobacteriales	Enterobacteriaceae	unclassified	unclassified
6211	11304	12099	11462	10872	12478	12857	12488	12825	Bacteria	Proteobacteria	Gammaproteobacteria	Enterobacteriales	Enterobacteriaceae	unclassified	unclassified
4011	9827	8223	7349	7560	11573	12083	11314	11886	Bacteria	Proteobacteria	Gammaproteobacteria	Oceanospirillales	Alcanivoracaceae	Kangiella	unclassified
3351	9643	9634	9109	8356	13676	11122	10579	10942	Bacteria	Proteobacteria	Gammaproteobacteria	Pseudomonadales	Moraxellaceae	Acinetobacter	Acinetobacter johnsonii
3360	9144	8056	7042	7051	13234	11214	11036	11456	Bacteria	Proteobacteria	Gammaproteobacteria	Pseudomonadales	Moraxellaceae	Acinetobacter	Acinetobacter johnsonii
3464	9836	9456	8959	7945	13020	10883	10450	10561	Bacteria	Proteobacteria	Gammaproteobacteria	Pseudomonadales	Moraxellaceae	Acinetobacter	Acinetobacter johnsonii
3482	9425	8777	7903	7678	13627	10396	10560	10738	Bacteria	Proteobacteria	Gammaproteobacteria	Pseudomonadales	Moraxellaceae	Acinetobacter	Acinetobacter johnsonii
3571	9573	9013	8544	7842	13256	10208	10286	10368	Bacteria	Proteobacteria	Gammaproteobacteria	Pseudomonadales	Moraxellaceae	Acinetobacter	Acinetobacter johnsonii
3624	9713	9225	8124	7894	13699	11891	11553	12124	Bacteria	Proteobacteria	Gammaproteobacteria	Pseudomonadales	Moraxellaceae	Acinetobacter	Acinetobacter johnsonii
3672	9671	8999	8326	7663	13279	10273	9860	10482	Bacteria	Proteobacteria	Gammaproteobacteria	Pseudomonadales	Moraxellaceae	Acinetobacter	Acinetobacter johnsonii
3746	9490	9861	8625	8239	13481	10943	10516	10705	Bacteria	Proteobacteria	Gammaproteobacteria	Pseudomonadales	Moraxellaceae	Acinetobacter	Acinetobacter johnsonii
3774	9783	8815	7568	7532	12805	12126	11433	11859	Bacteria	Proteobacteria	Gammaproteobacteria	Pseudomonadales	Moraxellaceae	Acinetobacter	Acinetobacter johnsonii
3871	10182	9273	9007	7850	13515	11235	10868	11240	Bacteria	Proteobacteria	Gammaproteobacteria	Pseudomonadales	Moraxellaceae	Acinetobacter	Acinetobacter johnsonii
4039	9681	7967	7915	7137	13741	11058	10777	11290	Bacteria	Proteobacteria	Gammaproteobacteria	Pseudomonadales	Moraxellaceae	Acinetobacter	Acinetobacter johnsonii
4055	9392	9422	8459	7975	13401	10305	10119	10556	Bacteria	Proteobacteria	Gammaproteobacteria	Pseudomonadales	Moraxellaceae	Acinetobacter	Acinetobacter johnsonii
4078	10046	8217	7692	7218	12716	11151	10272	10522	Bacteria	Proteobacteria	Gammaproteobacteria	Pseudomonadales	Moraxellaceae	Acinetobacter	Acinetobacter johnsonii
4093	10567	9713	9070	8193	13049	12057	11475	11784	Bacteria	Proteobacteria	Gammaproteobacteria	Pseudomonadales	Moraxellaceae	Acinetobacter	Acinetobacter johnsonii
4102	9580	8718	8234	7685	13526	10261	10427	10381	Bacteria	Proteobacteria	Gammaproteobacteria	Pseudomonadales	Moraxellaceae	Acinetobacter	Acinetobacter johnsonii
3369	13300	13303	13780	12535	13705	13780	13323	13326	Bacteria	Proteobacteria	Gammaproteobacteria	Pseudomonadales	Moraxellaceae	Acinetobacter	Acinetobacter lwoffii
3494	11149	11263	9849	10543	12910	12266	11781	12242	Bacteria	Proteobacteria	Gammaproteobacteria	Pseudomonadales	Moraxellaceae	Acinetobacter	Acinetobacter lwoffii
3647	8816	7135	6434	7122	11952	9888	9677	10430	Bacteria	Proteobacteria	Gammaproteobacteria	Pseudomonadales	Moraxellaceae	Acinetobacter	Acinetobacter schindleri
3796	10252	10491	9811	9513	12346	10521	9975	10605	Bacteria	Proteobacteria	Gammaproteobacteria	Pseudomonadales	Moraxellaceae	Acinetobacter	Acinetobacter schindleri
3752	8657	7508	7518	6894	12865	9380	9380	9760	Bacteria	Proteobacteria	Gammaproteobacteria	Pseudomonadales	Moraxellaceae	Acinetobacter	unclassified
3771	9891	8059	8073	7511	12595	11318	10549	11247	Bacteria	Proteobacteria	Gammaproteobacteria	Pseudomonadales	Moraxellaceae	Acinetobacter	unclassified
3925	10569	12553	11914	10081	12331	12320	12473	12673	Bacteria	Proteobacteria	Gammaproteobacteria	Pseudomonadales	Pseudomonadaceae	Pseudomonas	Pseudomonas mendocina
2281	9233	7973	8304	7677	11223	11027	11957	12341	Bacteria	Proteobacteria	Gammaproteobacteria	Pseudomonadales	Pseudomonadaceae	Pseudomonas	Pseudomonas stutzeri
1007	12871	13132	12696	12619	12433	12262	12220	12066	Bacteria	Proteobacteria	Gammaproteobacteria	Pseudomonadales	Pseudomonadaceae	Pseudomonas	unclassified
1095	10910	11248	11135	10732	12584	12846	12643	12607	Bacteria	Proteobacteria	Gammaproteobacteria	Pseudomonadales	Pseudomonadaceae	Pseudomonas	unclassified
1193	8159	7214	6345	6573	11177	9822	11581	11637	Bacteria	Proteobacteria	Gammaproteobacteria	Pseudomonadales	Pseudomonadaceae	Pseudomonas	unclassified
1257	10142	9548	8920	7994	12019	12462	11740	12347	Bacteria	Proteobacteria	Gammaproteobacteria	Pseudomonadales	Pseudomonadaceae	Pseudomonas	unclassified
1277	11630	11215	10772	10450	12610	12770	12278	12138	Bacteria	Proteobacteria	Gammaproteobacteria	Pseudomonadales	Pseudomonadaceae	Pseudomonas	unclassified
1302	10117	10316	9285	9227	12267	12329	12212	12103	Bacteria	Proteobacteria	Gammaproteobacteria	Pseudomonadales	Pseudomonadaceae	Pseudomonas	unclassified
1331	8374	7602	7109	6918	11775	10463	11820	11998	Bacteria	Proteobacteria	Gammaproteobacteria	Pseudomonadales	Pseudomonadaceae	Pseudomonas	unclassified
134	10266	10575	10440	8986	11552	11681	11756	11373	Bacteria	Proteobacteria	Gammaproteobacteria	Pseudomonadales	Pseudomonadaceae	Pseudomonas	unclassified
1440	8388	7763	7114	7029	11851	10560	11852	12117	Bacteria	Proteobacteria	Gammaproteobacteria	Pseudomonadales	Pseudomonadaceae	Pseudomonas	unclassified
1444	10204	11117	9825	8932	11546	11799	11758	11739	Bacteria	Proteobacteria	Gammaproteobacteria	Pseudomonadales	Pseudomonadaceae	Pseudomonas	unclassified
1481	9718	10405	9483	8682	11778	12449	11749	12092	Bacteria	Proteobacteria	Gammaproteobacteria	Pseudomonadales	Pseudomonadaceae	Pseudomonas	unclassified
1566	9778	10707	10066	8091	11458	11494	11094	11224	Bacteria	Proteobacteria	Gammaproteobacteria	Pseudomonadales	Pseudomonadaceae	Pseudomonas	unclassified
1567	11742	12430	11186	10974	13023	13125	12909	12977	Bacteria	Proteobacteria	Gammaproteobacteria	Pseudomonadales	Pseudomonadaceae	Pseudomonas	unclassified
158	12875	13131	12695	12617	12630	12581	12369	12464	Bacteria	Proteobacteria	Gammaproteobacteria	Pseudomonadales	Pseudomonadaceae	Pseudomonas	unclassified
160	9361	8911	8085	8571	11780	11438	11937	11889	Bacteria	Proteobacteria	Gammaproteobacteria	Pseudomonadales	Pseudomonadaceae	Pseudomonas	unclassified
1638	9953	10959	9945	9479	11615	11703	11662	11837	Bacteria	Proteobacteria	Gammaproteobacteria	Pseudomonadales	Pseudomonadaceae	Pseudomonas	unclassified
1650	10451	11937	10448	8981	12284	12600	12102	11955	Bacteria	Proteobacteria	Gammaproteobacteria	Pseudomonadales	Pseudomonadaceae	Pseudomonas	unclassified
1738	9238	7867	6826	6797	11132	11100	11618	11452	Bacteria	Proteobacteria	Gammaproteobacteria	Pseudomonadales	Pseudomonadaceae	Pseudomonas	unclassified
1796	8070	6345	6560	6910	11784	11291	11889	11595	Bacteria	Proteobacteria	Gammaproteobacteria	Pseudomonadales	Pseudomonadaceae	Pseudomonas	unclassified
182	8526	8314	6951	7284	11094	11517	11718	11565	Bacteria	Proteobacteria	Gammaproteobacteria	Pseudomonadales	Pseudomonadaceae	Pseudomonas	unclassified
1844	12635	13204	12525	12464	12873	12412	12972	12943	Bacteria	Proteobacteria	Gammaproteobacteria	Pseudomonadales	Pseudomonadaceae	Pseudomonas	unclassified
1884	11516	11267	11269	10556	12766	12510	12643	12895	Bacteria	Proteobacteria	Gammaproteobacteria	Pseudomonadales	Pseudomonadaceae	Pseudomonas	unclassified
1891	9228	9291	7926	7561	12115	12029	12019	11868	Bacteria	Proteobacteria	Gammaproteobacteria	Pseudomonadales	Pseudomonadaceae	Pseudomonas	unclassified
2058	8190	7283	6668	7609	11072	10086	11570	11686	Bacteria	Proteobacteria	Gammaproteobacteria	Pseudomonadales	Pseudomonadaceae	Pseudomonas	unclassified
339	10031	9874	9229	8516	11707	11917	11477	11825	Bacteria	Proteobacteria	Gammaproteobacteria	Pseudomonadales	Pseudomonadaceae	Pseudomonas	unclassified
3719	8412	7630	6839	7159	10502	9420	11756	11769	Bacteria	Proteobacteria	Gammaproteobacteria	Pseudomonadales	Pseudomonadaceae	Pseudomonas	unclassified

Table S6 (part U): HybScores of OTUs that were called present in at least one of the samples. Taxonomic classifications are based on PhyloChip taxonomy (derived from GreenGenes). Please note, that data presented here was rank normalized before performing statisical analyses presented in the manuscript.

	Sample ID (PMA)				Sample ID				taxonomic classification of representative rOTU cluster						
OTU	UR_PMA	CO_PMA	CR8_PMA	CR5_PMA	UR	CO	CR8	CR5	kingdom	phylum	class	order	family	genus	species
3736	8177	6446	6249	6863	9897	8367	11591	11651	Bacteria	Proteobacteria	Gammaproteobacteria	Pseudomonadales	Pseudomonadaceae	Pseudomonas	unclassified
421	8625	7213	6303	6956	11633	10870	11533	11945	Bacteria	Proteobacteria	Gammaproteobacteria	Pseudomonadales	Pseudomonadaceae	Pseudomonas	unclassified
507	9804	10810	10056	10030	11551	12269	11848	11881	Bacteria	Proteobacteria	Gammaproteobacteria	Pseudomonadales	Pseudomonadaceae	Pseudomonas	unclassified
508	8397	7930	7315	7692	11755	12432	11912	12264	Bacteria	Proteobacteria	Gammaproteobacteria	Pseudomonadales	Pseudomonadaceae	Pseudomonas	unclassified
841	10540	11070	10859	10050	11491	11860	11549	11586	Bacteria	Proteobacteria	Gammaproteobacteria	Pseudomonadales	Pseudomonadaceae	Pseudomonas	unclassified
17392	7648	10442	7464	6524	9849	13006	9630	9488	Bacteria	Proteobacteria	Gammaproteobacteria	Xanthomonadales	Xanthomonadaceae	Dokdonella	unclassified
17015	9650	10205	9190	10461	11625	13136	12326	11826	Bacteria	Proteobacteria	Gammaproteobacteria	Xanthomonadales	Xanthomonadaceae	Dyella	unclassified
16349	10693	11025	11325	10885	12076	12525	11829	12418	Bacteria	Proteobacteria	Gammaproteobacteria	Xanthomonadales	Xanthomonadaceae	Luteimonas	Luteimonas mephitis
16030	8890	11006	9190	7456	11629	12267	10962	11899	Bacteria	Proteobacteria	Gammaproteobacteria	Xanthomonadales	Xanthomonadaceae	Lysobacter	unclassified
16221	8674	8071	6980	6521	11106	12675	11075	12651	Bacteria	Proteobacteria	Gammaproteobacteria	Xanthomonadales	Xanthomonadaceae	Lysobacter	unclassified
16495	8084	6534	6139	6560	9728	12702	10986	11828	Bacteria	Proteobacteria	Gammaproteobacteria	Xanthomonadales	Xanthomonadaceae	Lysobacter	unclassified
16734	9420	10124	7876	7422	11811	12643	10859	10886	Bacteria	Proteobacteria	Gammaproteobacteria	Xanthomonadales	Xanthomonadaceae	Lysobacter	unclassified
16819	8498	11280	9031	6965	11852	12434	11601	11761	Bacteria	Proteobacteria	Gammaproteobacteria	Xanthomonadales	Xanthomonadaceae	Lysobacter	unclassified
16847	7979	8139	6248	6916	11844	12169	11069	11120	Bacteria	Proteobacteria	Gammaproteobacteria	Xanthomonadales	Xanthomonadaceae	Lysobacter	unclassified
15908	9023	8327	7446	7022	11656	10922	10353	10572	Bacteria	Proteobacteria	Gammaproteobacteria	Xanthomonadales	Xanthomonadaceae	Pseudoxanthomonas	unclassified
15920	9721	10881	9926	8606	10405	12350	10356	10890	Bacteria	Proteobacteria	Gammaproteobacteria	Xanthomonadales	Xanthomonadaceae	Rhodanobacter	unclassified
16340	7903	9600	7348	6386	7982	12229	8734	8804	Bacteria	Proteobacteria	Gammaproteobacteria	Xanthomonadales	Xanthomonadaceae	Rhodanobacter	unclassified
16376	9165	9893	9140	8475	10715	12836	11798	11197	Bacteria	Proteobacteria	Gammaproteobacteria	Xanthomonadales	Xanthomonadaceae	Rhodanobacter	unclassified
17357	9078	7829	6791	6892	10471	12146	10387	10438	Bacteria	Proteobacteria	Gammaproteobacteria	Xanthomonadales	Xanthomonadaceae	Rhodanobacter	unclassified
16520	8643	7353	7025	7360	12313	10179	9476	9734	Bacteria	Proteobacteria	Gammaproteobacteria	Xanthomonadales	Xanthomonadaceae	Stenotrophomonas	Pseudomonas geniculata
16522	7988	6786	6188	6948	12324	9966	9488	9829	Bacteria	Proteobacteria	Gammaproteobacteria	Xanthomonadales	Xanthomonadaceae	Stenotrophomonas	Pseudomonas geniculata
16590	8595	7435	6607	7503	12709	10976	10225	10344	Bacteria	Proteobacteria	Gammaproteobacteria	Xanthomonadales	Xanthomonadaceae	Stenotrophomonas	Pseudomonas geniculata
17322	8758	7749	6979	7557	12943	11484	10841	10926	Bacteria	Proteobacteria	Gammaproteobacteria	Xanthomonadales	Xanthomonadaceae	Stenotrophomonas	Pseudomonas geniculata
15941	9950	8875	6848	7352	12165	10910	9525	10376	Bacteria	Proteobacteria	Gammaproteobacteria	Xanthomonadales	Xanthomonadaceae	Stenotrophomonas	unclassified
16250	8975	8547	7457	7588	12327	11286	10611	10549	Bacteria	Proteobacteria	Gammaproteobacteria	Xanthomonadales	Xanthomonadaceae	Stenotrophomonas	unclassified
16971	9227	7776	7300	7556	12652	11036	11306	10826	Bacteria	Proteobacteria	Gammaproteobacteria	Xanthomonadales	Xanthomonadaceae	Stenotrophomonas	unclassified
17229	10464	10353	9555	9142	13395	12951	12531	12324	Bacteria	Proteobacteria	Gammaproteobacteria	Xanthomonadales	Xanthomonadaceae	Stenotrophomonas	unclassified
15917	8274	10320	7969	7137	11074	12449	10118	10341	Bacteria	Proteobacteria	Gammaproteobacteria	Xanthomonadales	Xanthomonadaceae	Thermomonas	unclassified
15987	9476	10661	7156	8170	11145	12425	10636	11888	Bacteria	Proteobacteria	Gammaproteobacteria	Xanthomonadales	Xanthomonadaceae	Thermomonas	unclassified
16000	11443	12265	11958	10414	13021	12983	12480	12314	Bacteria	Proteobacteria	Gammaproteobacteria	Xanthomonadales	Xanthomonadaceae	unclassified	unclassified
16096	8145	8279	7049	6547	12437	11641	11204	11123	Bacteria	Proteobacteria	Gammaproteobacteria	Xanthomonadales	Xanthomonadaceae	unclassified	unclassified
16717	8467	10029	7737	6835	12149	12491	11539	11382	Bacteria	Proteobacteria	Gammaproteobacteria	Xanthomonadales	Xanthomonadaceae	unclassified	unclassified
16161	9457	9254	6953	7785	12369	12379	10799	11558	Bacteria	Proteobacteria	Gammaproteobacteria	Xanthomonadales	Xanthomonadaceae	Xanthomonas	Xanthomonas axonopodis
16425	8691	9090	6903	7770	12209	12382	10755	11467	Bacteria	Proteobacteria	Gammaproteobacteria	Xanthomonadales	Xanthomonadaceae	Xanthomonas	Xanthomonas axonopodis
16916	9059	9219	7052	7622	12393	12113	10948	11536	Bacteria	Proteobacteria	Gammaproteobacteria	Xanthomonadales	Xanthomonadaceae	Xanthomonas	Xanthomonas axonopodis
17348	9049	8512	6733	7690	12006	11646	10185	11097	Bacteria	Proteobacteria	Gammaproteobacteria	Xanthomonadales	Xanthomonadaceae	Xanthomonas	Xanthomonas axonopodis
54344	11499	12186	10845	10794	13610	13598	13474	13808	Bacteria	SAR406	AB16	Arctic96B-7	A714017	SHAG537	unclassified
54147	11069	11088	10452	9790	11704	12604	11931	12150	Bacteria	SAR406	AB16	SSW63Au	SHAS460	unclassified	unclassified
54684	11009	11219	11324	10167	12602	13027	12515	13169	Bacteria	SAR406	AB16	SSW63Au	SHAS460	unclassified	unclassified
54850	8702	8891	8303	7376	11731	12133	11717	11944	Bacteria	SAR406	AB16	ZA3648c	AEGEAN_185	unclassified	unclassified
51571	7757	7134	6761	6984	10232	12088	11025	11416	Bacteria	SC4	unclassified	unclassified	unclassified	sfA	unclassified
76572	9757	10833	9553	9413	12297	12997	12358	12395	Bacteria	Spirochaetes	Spirochaetes	Spirochaetales	Spirochaetaceae	Spirochaeta	unclassified
71526	8607	8171	6523	7314	11217	11881	11337	11606	Bacteria	Spirochaetes	WWE1	PBS-18	SHA-4	Zplanct13	unclassified
54955	7996	8801	7279	7258	11213	12330	11850	11972	Bacteria	Synergistetes	Synergistia	Synergistales	Dethiosulfovibrionaceae	HA73	unclassified
71623	8314	8759	6438	6704	10270	11738	10787	11050	Bacteria	Tenericutes	Erysipelotrichi	Erysipelotrichales	Erysipelotrichaceae	PSB-M-3	unclassified
70828	11476	11793	11435	11032	13439	13297	13413	14144	Bacteria	Tenericutes	Mollicutes	Entomoplasmatales	Spiroplasmataceae	Spiroplasma	unclassified
71411	9425	9766	8698	8180	12644	12607	12388	12799	Bacteria	Tenericutes	Mollicutes	RF39	unclassified	sfA	unclassified
71601	8636	9563	8344	7874	11522	12271	11769	12122	Bacteria	Tenericutes	Mollicutes	RF39	unclassified	sfA	unclassified
53679	8522	7750	7076	7152	10822	12325	11072	12312	Bacteria	Thermi	Deinococci	Deinococcales	Deinococcaceae	Deinococcus	unclassified
53750	8571	8721	8002	6772	12423	13202	12521	12720	Bacteria	Thermi	Deinococci	Deinococcales	Deinococcaceae	Deinococcus	unclassified
53827	9134	9619	8253	7898	12534	13152	12074	12907	Bacteria	Thermi	Deinococci	Deinococcales	Deinococcaceae	Deinococcus	unclassified
55906	7646	6323	5699	6560	10597	10400	11537	11066	Bacteria	Verrucomicrobia	Opitutae	Opitutales	Opitutaceae	unclassified	unclassified
55241	9444	9801	8891	8642	11239	11804	11704	11664	Bacteria	Verrucomicrobia	Opitutae	Puniceicoccales	Puniceicoccaceae	unclassified	unclassified
55347	10478	11331	10079	9583	12601	13077	12349	12407	Bacteria	Verrucomicrobia	Verrucomicrobiae	Verrucomicrobiales	Verrucomicrobiaceae	unclassified	unclassified
56113	8531	8942	7399	7421	12043	13119	12114	12331	Bacteria	Verrucomicrobia	Verrucomicrobiae	Verrucomicrobiales	Verrucomicrobiaceae	unclassified	unclassified
58667	8144	8199	6778	6966	10631	11886	10754	11626	Bacteria	WS3	PRR-12	CV106	unclassified	sfA	unclassified
58579	11592	12208	9787	10836	11982	12807	11886	12286	Bacteria	WS3	PRR-12	GN03	unclassified	sfC	unclassified
58597	8744	8672	6467	6909	11644	12406	11369	11605	Bacteria	WS3	PRR-12	GN03	unclassified	sfC	unclassified
58646	8382	9601	6883	6833	11821	12482	11856	11901	Bacteria	WS3	PRR-12	GN03	unclassified	sfD	unclassified

Table S7: Archaeal 454 pyrotag sequencing reads

	Sample ID and no. of reads				taxonomic classification of archaeal 16S rRNA gene amplicons				
OTU	UR	CO	CR8	CR5	phylum	class	order	family	genus
0	0	3	0	0	Euryarchaeota	Methanomicrobia	Methanocellales	Methanocellaceae	Methanocella
2	34	0	0	0	Euryarchaeota	Halobacteria	Halobacteriales	Halobacteriaceae	unclassified
3	0	0	6	0	Euryarchaeota	Halobacteria	Halobacteriales	Halobacteriaceae	Halococcus
12	0	0	0	12	Euryarchaeota	Halobacteria	Halobacteriales	Halobacteriaceae	Halococcus
16	0	15	1	14	Euryarchaeota	Halobacteria	Halobacteriales	Halobacteriaceae	Halalkalicoccus
17	0	1	14	0	Euryarchaeota	Halobacteria	Halobacteriales	Halobacteriaceae	Halococcus
1	0	1	0	0	Thaumarchaeota	Thaumarchaeota	Nitrososphaerales	Nitrososphaeraceae	Candidatus Nitrososphaera (SCA1145)
4	1	0	0	0	Thaumarchaeota	Thaumarchaeota	Nitrososphaerales	Nitrososphaeraceae	Candidatus Nitrososphaera (SCA1170)
5	79	120	3	40	Thaumarchaeota	Thaumarchaeota	Nitrososphaerales	Nitrososphaeraceae	Candidatus Nitrososphaera (SCA1145)
6	0	1	0	0	Thaumarchaeota	Thaumarchaeota	Nitrososphaerales	Nitrososphaeraceae	Candidatus Nitrososphaera
7	3	2	1	0	Thaumarchaeota	Thaumarchaeota	Nitrososphaerales	Nitrososphaeraceae	Candidatus Nitrososphaera (SCA1170)
8	0	3	0	0	Thaumarchaeota	Thaumarchaeota	Nitrososphaerales	Nitrososphaeraceae	Candidatus Nitrososphaera (SCA1170)
9	1	72	21	16	Thaumarchaeota	Thaumarchaeota	Nitrososphaerales	Nitrososphaeraceae	Candidatus Nitrososphaera
10	7	6	0	1	Thaumarchaeota	Thaumarchaeota	Nitrososphaerales	Nitrososphaeraceae	Candidatus Nitrososphaera (SCA1145)
11	0	1	0	0	Thaumarchaeota	Thaumarchaeota	Nitrososphaerales	Nitrososphaeraceae	Candidatus Nitrososphaera (SCA1145)
13	0	2	1	0	Thaumarchaeota	Thaumarchaeota	Nitrososphaerales	Nitrososphaeraceae	Candidatus Nitrososphaera (SCA1145)
14	0	1	0	0	Thaumarchaeota	Thaumarchaeota	Nitrososphaerales	Nitrososphaeraceae	Candidatus Nitrososphaera
15	0	1	0	0	Thaumarchaeota	Thaumarchaeota	Nitrososphaerales	Nitrososphaeraceae	Candidatus Nitrososphaera (N. gargensis)

Supplementary Fig. to Table S7: Assignment of picked archaeal OTUs to their phylogenetic groups. Proportions are shown in relative abundance [%]. Beside the family of Halobacteriaceae all other taxonomic ranks are resolved up to genus level. Colored boxes (from left to right) show their presence throughout sampled rooms (UR, CO, CR8 and CR5).

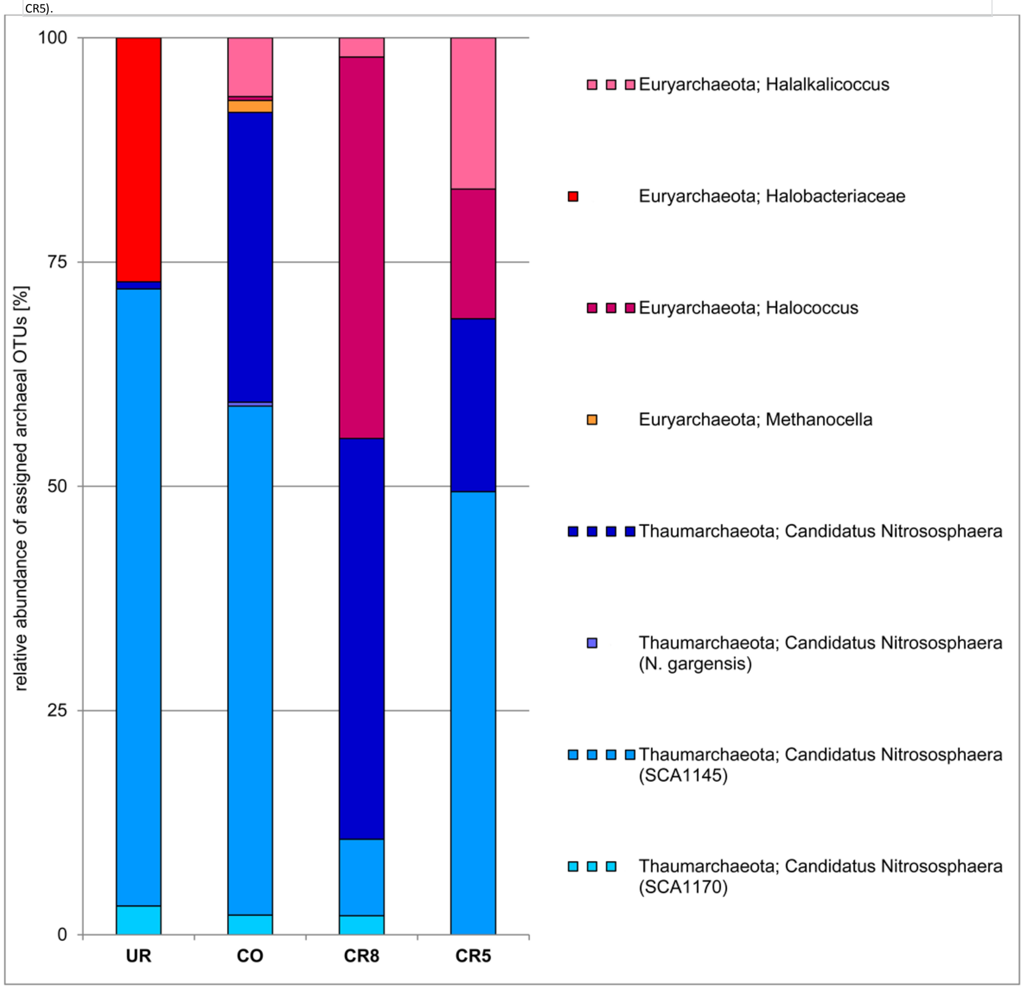

Table S8: Detected bacterial diversity in the extraction blank sample by 16S rRNA gene cloning (cOTUs), PhyloChip (rOTUs) and 454 pyrotag sequencing (pOTUs).

	**cOTUs**				
	Acc. No./ OTU No.	class	order	family	genus
16S rRNA gene cloning	JQ855568	Actinobacteria	Actinomycetales	Brevibacteriaceae	Brevibacterium
	JQ855515	Bacilli	Lactobacillales	Carnobacteriaceae	Trichococcus
	JQ855519	Alphaproteobacteria	Rhizobiales	Bradyrhizobiaceae	Afipia
	JQ855602	Alphaproteobacteria	Rhizobiales	Phyllobacteriaceae	unclassified
	JQ855523	Alphaproteobacteria	Rhizobiales	Xynthobacteraceae	unclassified
	JQ855574	Alphaproteobacteria	Sphingomonadales	Sphingomonadaceae	Sphingomonas
	JQ855516	Alphaproteobacteria	Sphingomonadales	Sphingomonadaceae	Sphingomonas
	JQ855557	Alphaproteobacteria	Rhodobacterales	Rhodobacteraceae	Rubellimicrobium
	JQ855508	Betaproteobacteria	Burkholderiales	Comamonadaceae	Variovorax
	JQ855524	Betaproteobacteria	Burkholderiales	Comamonadaceae	Variovorax
	**rOTUs**				
PhyloChip		Actinobacteria	Actinomycetales	Corynebacteriaceae	Corynebacterium
		Actinobacteria	Actinomycetales	Corynebacteriaceae	Corynebacterium
		Actinobacteria	Actinomycetales	Corynebacteriaceae	Corynebacterium
		Actinobacteria	Actinomycetales	Corynebacteriaceae	Corynebacterium
		Actinobacteria	Actinomycetales	Corynebacteriaceae	Corynebacterium
		Actinobacteria	Actinomycetales	Corynebacteriaceae	Corynebacterium
		Actinobacteria	Actinomycetales	Corynebacteriaceae	Corynebacterium
		Actinobacteria	Actinomycetales	Corynebacteriaceae	Corynebacterium
		Actinobacteria	Actinomycetales	Corynebacteriaceae	Corynebacterium
		Actinobacteria	Actinomycetales	Corynebacteriaceae	Corynebacterium
		Alphaproteobacteria	Rhizobiales	Rhizobiaceae	Ochrobactrum
		Alphaproteobacteria	Sphingomonadales	Sphingomonadaceae	Sphingomonas
		Betaproteobacteria	Burkholderiales	Aquabacteriaceae	Aquabacterium
		Betaproteobacteria	Burkholderiales	Aquabacteriaceae	Aquabacterium
		Betaproteobacteria	Burkholderiales	Aquabacteriaceae	Aquabacterium
		Betaproteobacteria	Burkholderiales	Comamonadaceae	Diaphorobacter
		Betaproteobacteria	Burkholderiales	Comamonadaceae	Diaphorobacter
		Betaproteobacteria	Burkholderiales	Comamonadaceae	Giesbergeria
		Betaproteobacteria	Burkholderiales	Comamonadaceae	Simplicispira
		Betaproteobacteria	Burkholderiales	Comamonadaceae	unclassified
		Deltaproteobacteria	Myxococcales	Haliangiaceae	unclassified
	**pOTUs**				
bacterial 16S rRNA amplicons by 454 pyrotag sequencing		Actinobacteria	Actinomycetales	Micrococcaceae	Micrococcus
		Actinobacteria	Actinomycetales	Micrococcaceae	Arthrobacter
		Actinobacteria	Actinomycetales	Nocardiaceae	Rhodococcus
		Actinobacteria	Actinomycetales	Propionibacteriaceae	Propionibacterium
		Actinobacteria	Actinomycetales	unclassified	unclassified
		Actinobacteria	Solirubrobacterales	unclassified	unclassified
		Alphaproteobacteria	Caulobacterales	Caulobacteraceae	unclassified
		Alphaproteobacteria	Rhizobiales	Bradyrhizobiaceae	Afipia
		Alphaproteobacteria	Rhizobiales	Methylobacteriaceae	Methylobacterium
		Alphaproteobacteria	Rhodobacterales	Rhodobacteraceae	Paracoccus
		Alphaproteobacteria	Sphingomonadales	Sphingomonadaceae	Novosphingobium
		Alphaproteobacteria	Sphingomonadales	Sphingomonadaceae	Sphingobium
		Alphaproteobacteria	Sphingomonadales	Sphingomonadaceae	Sphingomonas
		Anaerolineae	Anaerolineales	Anaerolinaceae	DRC31
		Bacilli	Bacillales	Paenibacillaceae	unclassified
		Bacilli	Bacillales	Staphylococcaceae	Staphylococcus
		Bacteroidia	Bacteroidales	Prevotellaceae	Prevotella
		Betaproteobacteria	Burkholderiales	Aquabacteriaceae	Aquabacterium
		Betaproteobacteria	Burkholderiales	Burkholderiaceae	Burkholderia
		Betaproteobacteria	Burkholderiales	Burkholderiaceae	Pandoraea
		Betaproteobacteria	Burkholderiales	Comamonadaceae	Variovorax
		Betaproteobacteria	Burkholderiales	Oxalobacteraceae	Janthinobacterium
		Betaproteobacteria	Burkholderiales	Oxalobacteraceae	Massilia
		Betaproteobacteria	Burkholderiales	Burkholderiaceae	Pandoraea
		Betaproteobacteria	Burkholderiales	Burkholderiaceae	Chitinimonas
		Betaproteobacteria	Methylophilales	Methylophilaceae	Methylotenera
		Betaproteobacteria	Neisseriales	Neisseriaceae	Chromobacterium
		Chloracidobacteria	unclassified	unclassified	unclassified
		Chloroplast	Streptophyta	unclassified	unclassified
		Flavobacteria	Flavobacteriales	Flavobacteriaceae	Flavobacterium
		Gammaproteobacteria	Chromatiales	Sinobacteraceae	Steroidobacter
		Gammaproteobacteria	Enterobacteriales	Enterobacteriaceae	Yersinia
		Gammaproteobacteria	Oceanospirillales	Halomonadaceae	Halomonas
		Gammaproteobacteria	Pseudomonadales	Moraxellaceae	Acinetobacter
		Gammaproteobacteria	Pseudomonadales	Pseudomonadaceae	Pseudomonas
		Gammaproteobacteria	Xanthomonadales	Xanthomonadaceae	Pseudoxanthomonas
		Gammaproteobacteria	unclassified	unclassified	unclassified
		Sphingobacteria	Sphingobacteriales	Rhodothermaceae	unclassified
		Synechococcophycide	Pseudanabaenales	Pseudanabaenaceae	unclassified
		Thermomicrobia	unclassified	unclassified	unclassified

Table S9.1. real node table for pOTU network (based on pyrotagsequencing data) and visualization in Cytoscape

node_name	degree	weighted_degree	shared
denovo0	1	3	CR8
denovo1	1	2	CR8
denovo10	1	1	CO
denovo100	1	14	CO
denovo1000	2	4	CR8,CR5
denovo1001	2	2	CO,CR5
denovo1002	1	1	CR8
denovo1003	1	8	CO
denovo1004	1	1	CO
denovo1005	1	1	CR5
denovo1006	1	2	CO
denovo1007	1	1	CO
denovo1008	1	3	CO
denovo1009	2	2	CO,CR8
denovo101	1	1	CR8
denovo1010	1	1	CO
denovo1011	1	1	CR8
denovo1012	1	1	CO
denovo1013	2	8	CO,CR5
denovo1014	4	25	CO,UR,CR8,CR5
denovo1015	2	7	UR,CR5
denovo1016	2	2	CO,CR8
denovo1017	1	3	CO
denovo1018	3	25	CO,CR8,CR5
denovo1019	1	1	CR5
denovo102	1	1	CO
denovo1020	1	1	CR5
denovo1021	1	1	CO
denovo1023	1	7	CO
denovo1024	1	6	CO
denovo1025	1	10	UR
denovo1026	1	4	CR8
denovo1027	1	13	CO
denovo1028	1	1	CR8
denovo1029	1	1	CR5
denovo103	1	1	CR5
denovo1030	1	1	CO
denovo1031	1	1	CR5
denovo1032	1	1	CO
denovo1033	1	1	CR8
denovo1034	1	1	CR5
denovo1035	2	4	CO,CR5
denovo1036	1	1	UR
denovo1037	4	11	CO,UR,CR8,CR5
denovo1038	1	1	UR
denovo104	1	1	CO
denovo1040	2	4	CO,CR5
denovo1041	1	1	UR
denovo1042	1	2	CO
denovo1043	2	12	UR,CR8
denovo1044	3	8	UR,CR8,CR5
denovo1045	3	9	UR,CR8,CR5
denovo1046	1	14	CO
denovo1047	1	1	CR5
denovo1048	1	1	CO
denovo1049	1	1	CR5
denovo105	1	4	CR5
denovo1051	1	2	CR8
denovo1052	1	1	CR5
denovo1053	1	1	CR5
denovo1054	2	11	CO,CR8
denovo1055	1	1	CO
denovo1056	1	3	CO

denovo1057	1	2	CO
denovo1058	1	1	CO
denovo1059	4	25	CO,UR,CR8,CR5
denovo106	2	7	CO,CR5
denovo1060	1	3	CR5
denovo1062	1	1	UR
denovo1063	1	3	CO
denovo1064	2	5	CR8,CR5
denovo1065	1	1	CO
denovo1066	1	1	CO
denovo1067	2	3	CO,CR5
denovo1068	1	2	CR5
denovo1069	1	1	CR5
denovo107	3	4	CO,CR8,CR5
denovo1070	1	1	CR8
denovo1071	1	5	CR8
denovo1072	1	1	CO
denovo1073	3	3	CO,CR8,CR5
denovo1074	1	1	CR8
denovo1075	1	1	CO
denovo1076	1	2	CR8
denovo1077	2	9	CO,CR8
denovo1078	1	2	CO
denovo1079	1	8	CR5
denovo108	1	1	CR5
denovo1080	1	5	CO
denovo1081	1	1	CO
denovo1082	1	2	CR8
denovo1083	1	4	CO
denovo1084	3	11	CO,UR,CR5
denovo1085	1	1	CR8
denovo1086	4	15	CO,UR,CR8,CR5
denovo1088	2	4	CO,CR5
denovo1089	1	1	CR8
denovo109	3	11	CO,CR8,CR5
denovo1090	2	7	CR8,CR5
denovo1091	1	1	CO
denovo1093	3	5	CO,UR,CR5
denovo1094	4	63	CO,UR,CR8,CR5
denovo1096	1	1	CR5
denovo1097	1	2	UR
denovo1098	1	1	CO
denovo1099	1	2	CR5
denovo11	1	1	CO
denovo110	1	1	UR
denovo1100	2	4	CO,UR
denovo1101	2	2	CO,CR8
denovo1102	1	1	CO
denovo1103	2	5	CO,CR5
denovo1104	1	1	CR5
denovo1105	1	1	CR5
denovo1107	1	1	CR5
denovo1108	2	3	CR8,CR5
denovo1109	1	83	CO
denovo111	1	1	CO
denovo1110	1	1	CR8
denovo1111	1	1	CR5
denovo1112	1	2	CO
denovo1113	1	1	CO
denovo1114	2	2	CO,CR8
denovo1115	1	2	CR5
denovo1116	1	1	CR8
denovo1117	1	1	CR5
denovo1118	1	1	CO
denovo1119	1	1	CO
denovo112	1	1	CR5
denovo1120	1	1	CR8

denovo1121	3	6	CO,UR,CR5
denovo1122	3	167	CO,UR,CR5
denovo1123	1	2	CR8
denovo1124	1	107	CO
denovo1125	1	1	CR8
denovo1127	1	1	CR5
denovo1128	1	1	CR5
denovo1129	1	1	CR5
denovo113	1	1	UR
denovo1130	1	1	CR8
denovo1131	2	3	CO,CR8
denovo1132	3	4	CO,UR,CR8
denovo1133	1	1	CO
denovo1134	1	2	CO
denovo1135	1	1	CO
denovo1136	1	1	CO
denovo1137	1	2	CR8
denovo1139	1	1	CR8
denovo114	3	8	CO,CR8,CR5
denovo1140	1	1	CO
denovo1141	1	1	CR8
denovo1142	1	2	UR
denovo1143	1	1	CO
denovo1144	1	1	CO
denovo1145	1	4	CR8
denovo1146	1	1	CR8
denovo1147	1	1	CR8
denovo1148	1	3	CR8
denovo1149	2	11	CO,CR5
denovo115	2	5	CO,CR5
denovo1150	3	9	CO,CR8,CR5
denovo1151	3	6	CO,UR,CR5
denovo1152	1	3	CR5
denovo1153	2	6	CO,CR8
denovo1154	2	3	CO,CR5
denovo1155	1	1	CR8
denovo1156	1	1	CR5
denovo1157	2	2	CO,CR5
denovo1158	1	1	UR
denovo1159	1	2	CR5
denovo116	2	3	CR8,CR5
denovo1160	1	1	CR8
denovo1161	3	7	UR,CR8,CR5
denovo1162	1	1	CO
denovo1164	1	2	CO
denovo1165	4	15	CO,UR,CR8,CR5
denovo1166	1	2	CO
denovo1167	1	2	CR5
denovo1168	1	2	CO
denovo1169	1	1	CO
denovo117	1	1	UR
denovo1170	2	2	CO,CR8
denovo1171	1	2	CR8
denovo1172	1	1	CR8
denovo1173	4	48	CO,UR,CR8,CR5
denovo1174	1	1	CO
denovo1175	1	2	CO
denovo1177	1	1	CR5
denovo1178	2	7	CO,CR5
denovo1179	1	4	CO
denovo118	1	1	CO
denovo1181	1	2	CO
denovo1182	1	1	CO
denovo1183	1	2	CO
denovo1184	3	11	CO,UR,CR8
denovo1185	1	1	CR5
denovo1186	1	1	CR5

denovo1187	1	3	CO
denovo1188	1	1	CR5
denovo119	1	1	CR8
denovo1190	1	1	UR
denovo1191	1	1	CR8
denovo1192	1	1	CR5
denovo1193	2	2	CO,UR
denovo1194	1	2	CO
denovo1195	2	2	CO,CR5
denovo1196	1	1	CR5
denovo1197	1	5	CO
denovo1198	1	1	CO
denovo1199	2	2	CO,CR8
denovo12	1	1	CO
denovo1200	1	1	CR8
denovo1201	2	4	CO,CR8
denovo1202	4	52	CO,UR,CR8,CR5
denovo1203	2	3	CO,UR
denovo1205	1	1	UR
denovo1206	1	1	CR8
denovo1207	1	1	CR8
denovo1208	1	1	CR5
denovo1209	1	1	CO
denovo1210	1	2	CR8
denovo1211	1	3	CO
denovo1212	1	1	UR
denovo1215	1	1	UR
denovo1216	1	1	CR8
denovo1217	3	6	CO,CR8,CR5
denovo1218	1	2	UR
denovo1219	1	1	CO
denovo122	1	1	CR5
denovo1220	1	1	UR
denovo1221	1	1	CO
denovo1222	1	2	CO
denovo1223	1	1	CR8
denovo1224	1	1	CO
denovo1225	1	1	CR8
denovo1226	1	4	CO
denovo1227	3	6	CO,CR8,CR5
denovo1228	1	3	UR
denovo1229	1	1	CO
denovo123	1	2	CR5
denovo1230	3	5	CO,UR,CR8
denovo1231	3	12	CO,CR8,CR5
denovo1232	1	1	CO
denovo1233	1	1	CR8
denovo1234	1	2	UR
denovo1235	3	3	CO,CR8,CR5
denovo1236	3	5	CO,CR8,CR5
denovo1237	1	1	CR8
denovo1238	1	2	CR8
denovo1239	1	2	CR5
denovo124	1	1	CR8
denovo1241	1	2	UR
denovo1242	1	13	CO
denovo1243	1	11	CO
denovo1244	1	3	CR5
denovo1245	1	1	CR5
denovo1246	2	2	CO,CR5
denovo1247	1	1	CR5
denovo1248	2	5	UR,CR8
denovo125	1	1	CO
denovo1250	1	3	UR
denovo1251	1	1	CO
denovo1252	1	2	CR5
denovo1253	4	34	CO,UR,CR8,CR5

denovo1254	1	2	UR
denovo1255	2	2	CR8,CR5
denovo1256	1	1	CO
denovo1257	4	44	CO,UR,CR8,CR5
denovo1258	1	2	CR5
denovo1259	1	4	CO
denovo126	2	4	CO,CR8
denovo1260	1	1	CR8
denovo1261	1	2	CR5
denovo1262	1	1	CO
denovo1263	1	3	CO
denovo1264	1	2	CO
denovo1265	1	2	CR8
denovo1266	1	1	CO
denovo1267	1	3	CR8
denovo1268	1	3	CO
denovo1269	1	1	CR8
denovo127	2	2	CO,CR8
denovo1270	1	1	CR8
denovo1271	1	3	CR8
denovo1272	1	1	UR
denovo1273	1	1	CR5
denovo1274	1	1	CO
denovo1276	1	1	CR8
denovo1277	3	8	CO,CR8,CR5
denovo1278	2	2	CO,CR5
denovo1279	3	7	CO,CR8,CR5
denovo128	1	2	CO
denovo1280	1	1	CR8
denovo1281	1	2	CO
denovo1282	1	1	CR5
denovo1283	1	2	CO
denovo1284	1	2	UR
denovo1285	1	2	CO
denovo1287	2	6	CR8,CR5
denovo1288	1	3	CR8
denovo1289	1	1	CR5
denovo129	1	1	CO
denovo1290	4	33	CO,UR,CR8,CR5
denovo1291	1	2	CR5
denovo1292	2	4	CO,CR8
denovo1293	2	3	CO,CR8
denovo1294	1	15	CO
denovo1295	1	1	CR5
denovo1297	1	1	CO
denovo1298	1	1	UR
denovo1299	1	1	CO
denovo13	1	4	CR8
denovo130	1	1	CR8
denovo1300	4	68	CO,UR,CR8,CR5
denovo1301	3	6	UR,CR8,CR5
denovo1302	1	2	UR
denovo1303	3	3	CO,CR8,CR5
denovo1304	1	1	CO
denovo1305	1	1	CO
denovo1306	1	1	CR5
denovo1307	1	8	CO
denovo1308	4	34	CO,UR,CR8,CR5
denovo1309	2	3	CO,CR8
denovo131	1	2	CR5
denovo1310	1	3	CO
denovo1311	3	15	CO,UR,CR5
denovo1312	1	1	CR8
denovo1313	2	2	CO,CR5
denovo1315	1	1	CR5
denovo1317	1	2	CR8
denovo1318	1	3	CR8

denovo1319	2	4	CO,CR8
denovo132	2	7	CO,CR5
denovo1320	1	1	CO
denovo1321	1	1	CR5
denovo1322	1	1	CR8
denovo1323	2	8	CO,CR8
denovo1324	3	6	CO,CR8,CR5
denovo1325	1	1	CO
denovo1326	1	2	CO
denovo1327	1	1	CO
denovo1328	1	3	CR8
denovo1329	2	2	CR8,CR5
denovo133	1	3	CR8
denovo1330	1	1	CR5
denovo1331	1	1	CO
denovo1332	1	1	CO
denovo1334	4	176	CO,UR,CR8,CR5
denovo1335	1	3	CR8
denovo1336	2	2	CO,CR8
denovo1337	1	1	CO
denovo1339	1	1	CR5
denovo134	4	77	CO,UR,CR8,CR5
denovo1341	4	24	CO,UR,CR8,CR5
denovo1342	2	2	CO,CR8
denovo1343	2	5	CO,CR5
denovo1344	1	1	CO
denovo1345	3	8	CO,CR8,CR5
denovo1346	4	62	CO,UR,CR8,CR5
denovo1347	3	5	UR,CR8,CR5
denovo1349	2	10	CO,CR8
denovo135	1	1	CR8
denovo1350	2	40	UR,CR5
denovo1351	2	5	CO,CR5
denovo1352	1	32	CO
denovo1353	1	2	CO
denovo1354	1	13	CO
denovo1355	1	1	CO
denovo1356	1	8	CO
denovo1357	2	3	UR,CR8
denovo1358	1	1	CO
denovo1359	1	1	CO
denovo136	1	1	CR5
denovo1360	1	1	CO
denovo1361	1	12	CO
denovo137	2	5	CO,CR5
denovo138	4	18	CO,UR,CR8,CR5
denovo14	1	1	CO
denovo140	1	1	CR8
denovo141	1	1	CO
denovo142	1	3	CO
denovo143	1	1	CO
denovo144	1	1	CO
denovo145	1	1	CR5
denovo147	1	3	CO
denovo148	2	2	CO,CR8
denovo149	3	4	CO,UR,CR8
denovo15	3	6	CO,CR8,CR5
denovo150	1	2	CO
denovo151	1	2	CO
denovo152	3	3	CO,CR8,CR5
denovo153	2	3	CO,CR8
denovo154	1	1	CO
denovo155	3	7	CO,CR8,CR5
denovo156	2	2	CO,CR5
denovo157	1	1	CR8
denovo158	2	4	CO,CR5
denovo159	2	11	CO,UR

denovo160	1	1	CR8
denovo161	1	2	CR8
denovo162	1	2	CR8
denovo163	1	1	CO
denovo164	1	1	CR8
denovo165	2	2	CO,CR5
denovo166	1	2	UR
denovo167	1	1	CO
denovo168	1	2	CR8
denovo169	2	2	CR8,CR5
denovo17	1	1	CR8
denovo170	2	3	CR8,CR5
denovo172	1	1	CO
denovo173	1	4	CO
denovo174	2	2	UR,CR5
denovo175	1	1	CR5
denovo176	1	2	CR8
denovo177	1	31	CO
denovo178	4	10	CO,UR,CR8,CR5
denovo179	1	1	CR8
denovo18	3	10	UR,CR8,CR5
denovo180	2	2	CR8,CR5
denovo181	1	1	CR5
denovo182	1	1	CR8
denovo183	4	332	CO,UR,CR8,CR5
denovo184	4	43	CO,UR,CR8,CR5
denovo185	4	84	CO,UR,CR8,CR5
denovo186	2	4	CR8,CR5
denovo187	4	16	CO,UR,CR8,CR5
denovo189	1	1	CR8
denovo19	1	16	CO
denovo190	1	1	CR5
denovo191	1	2	CR5
denovo192	1	1	CR8
denovo193	4	9	CO,UR,CR8,CR5
denovo194	1	1	CR5
denovo195	1	2	CR8
denovo196	1	1	CO
denovo197	1	2	CO
denovo198	1	1	CO
denovo199	1	1	UR
denovo2	2	2	CO,CR8
denovo20	1	5	CO
denovo200	1	42	CO
denovo201	2	3	UR,CR8
denovo202	2	4	CO,CR5
denovo203	1	2	CO
denovo204	1	1	CR5
denovo205	1	1	CO
denovo206	1	1	CR5
denovo207	4	13	CO,UR,CR8,CR5
denovo208	1	1	CR8
denovo209	1	1	CO
denovo21	1	1	CR5
denovo210	4	13	CO,UR,CR8,CR5
denovo211	1	1	CO
denovo212	1	1	CO
denovo213	4	10	CO,UR,CR8,CR5
denovo214	1	1	CR8
denovo215	1	10	CO
denovo216	1	2	CO
denovo217	1	3	CO
denovo218	3	11	CO,CR8,CR5
denovo219	3	5	CO,UR,CR5
denovo22	1	1	CO
denovo221	1	3	CR8
denovo222	1	1	CO

denovo223	1	2	CO
denovo224	1	1	UR
denovo225	1	3	CR8
denovo226	1	1	CO
denovo228	4	9	CO,UR,CR8,CR5
denovo229	2	8	CO,CR8
denovo23	2	2	CO,CR5
denovo231	1	1	CR8
denovo232	3	65	UR,CR8,CR5
denovo233	1	1	CO
denovo234	1	2	CR8
denovo235	1	5	CO
denovo236	3	7	CO,CR8,CR5
denovo237	1	115	CO
denovo239	1	3	CO
denovo24	1	1	CR8
denovo240	1	1	CO
denovo241	2	2	CO,CR5
denovo242	1	1	CO
denovo244	1	1	UR
denovo245	1	1	UR
denovo246	1	3	CR5
denovo247	1	1	CR5
denovo248	1	2	CR8
denovo249	1	1	CR5
denovo25	1	2	CR5
denovo250	1	3	CO
denovo251	1	2	CO
denovo252	2	8	UR,CR5
denovo253	1	2	UR
denovo254	1	2	CR8
denovo255	1	3	CR8
denovo256	1	1	CO
denovo258	1	1	CO
denovo259	1	1	CO
denovo26	1	1	CO
denovo261	1	1	CR5
denovo262	1	2	CO
denovo263	1	1	CO
denovo264	1	1	CR8
denovo265	1	3	CO
denovo266	2	6	CO,UR
denovo267	1	1	UR
denovo268	1	1	CO
denovo269	1	1	CR5
denovo27	1	1	CR8
denovo270	1	1	CR8
denovo271	1	1	UR
denovo272	3	18	CO,CR8,CR5
denovo273	1	2	CR8
denovo274	1	3	CR5
denovo275	1	1	CR8
denovo276	1	1	CO
denovo277	1	1	CR8
denovo279	1	1	CO
denovo28	1	1	CO
denovo280	1	2	CO
denovo281	1	3	CR5
denovo282	2	2	UR,CR5
denovo283	1	1	CO
denovo284	1	1	CR5
denovo285	1	1	CR8
denovo286	2	3	CO,CR8
denovo287	1	5	CO
denovo288	4	16	CO,UR,CR8,CR5
denovo289	1	3	UR
denovo29	1	1	CR5

denovo290	2	4	CO,CR8
denovo291	3	4	CO,UR,CR8
denovo292	1	1	CR8
denovo293	2	3	CO,UR
denovo294	3	3	CO,UR,CR5
denovo295	1	2	CR8
denovo296	3	15	CO,CR8,CR5
denovo297	1	1	CR5
denovo298	1	1	CR8
denovo299	1	1	CR8
denovo3	1	1	CO
denovo30	2	5	CO,UR
denovo301	1	2	CO
denovo302	1	1	CO
denovo303	1	1	CR8
denovo305	1	1	CR8
denovo306	1	3	UR
denovo308	1	1	CO
denovo309	1	1	CO
denovo31	1	1	CR8
denovo310	2	6	CO,CR5
denovo311	1	1	CO
denovo312	1	1	CO
denovo313	1	2	CR8
denovo314	2	5	CO,CR8
denovo315	2	13	CO,CR8
denovo316	1	1	CR5
denovo317	1	2	CO
denovo318	2	3	CO,UR
denovo319	2	4	CO,CR8
denovo32	1	1	CO
denovo320	1	3	CO
denovo321	1	2	CR5
denovo322	1	1	UR
denovo323	3	4	CO,UR,CR8
denovo324	2	8	CO,CR8
denovo325	2	5	CR8,CR5
denovo326	1	2	CO
denovo327	3	4	CO,CR8,CR5
denovo329	2	3	CO,CR5
denovo33	1	1	CO
denovo330	1	1	CR5
denovo331	1	2	CR8
denovo332	1	1	CR8
denovo333	1	2	CR8
denovo334	1	1	CR8
denovo335	3	7	CO,CR8,CR5
denovo336	1	1	CO
denovo337	1	4	CR8
denovo338	1	16	CO
denovo34	2	27	CO,CR8
denovo340	2	8	CR8,CR5
denovo343	1	3	CO
denovo344	1	2	CO
denovo345	1	1	CR5
denovo346	1	1	CR5
denovo347	2	2	CO,CR5
denovo348	1	1	CR8
denovo349	2	4	CR8,CR5
denovo35	1	12	CR8
denovo350	1	1	CR5
denovo352	1	1	CO
denovo353	4	71	CO,UR,CR8,CR5
denovo354	1	1	CO
denovo355	1	1	CR5
denovo356	1	1	CO
denovo357	2	7	CR8,CR5

denovo358	1	1	CO
denovo359	1	5	CR8
denovo36	1	1	CO
denovo360	1	3	CR8
denovo361	3	23	CO,UR,CR8
denovo362	1	1	CR8
denovo364	1	2	CO
denovo366	1	17	CO
denovo367	1	1	CR8
denovo368	1	1	CO
denovo369	1	2	CO
denovo37	1	1	CR8
denovo370	1	1	CR5
denovo371	2	2	CO,CR5
denovo372	1	2	CO
denovo373	1	1	CO
denovo374	2	7	CO,UR
denovo375	1	1	CO
denovo376	1	1	CR5
denovo377	3	5	CO,CR8,CR5
denovo378	1	2	CR8
denovo379	1	1	CR8
denovo38	1	9	CR8
denovo380	1	1	CR8
denovo381	1	1	CR5
denovo382	1	1	CO
denovo383	1	1	CR5
denovo384	2	7	CO,CR8
denovo385	1	3	CR5
denovo386	1	1	CO
denovo387	2	2	CR8,CR5
denovo388	1	3	UR
denovo389	2	3	CO,CR8
denovo390	3	9	CO,CR8,CR5
denovo391	1	1	UR
denovo392	1	1	CO
denovo393	4	47	CO,UR,CR8,CR5
denovo394	1	1	CO
denovo395	1	4	CO
denovo396	1	3	CO
denovo397	1	1	CO
denovo398	2	2	CO,CR8
denovo399	1	1	CO
denovo4	1	1	CO
denovo40	1	1	UR
denovo400	2	2	CO,CR8
denovo401	1	1	CO
denovo402	1	1	CR5
denovo403	1	12	CR5
denovo404	4	22	CO,UR,CR8,CR5
denovo405	1	1	CO
denovo406	1	1	CO
denovo407	1	4	CR8
denovo408	1	1	CR5
denovo409	2	4	CO,CR5
denovo41	1	1	CO
denovo410	1	4	CR8
denovo411	1	1	CO
denovo413	1	1	CO
denovo414	3	3	CO,CR8,CR5
denovo415	2	10	CO,CR5
denovo416	2	3	CO,CR8
denovo417	1	1	CO
denovo418	1	1	CO
denovo419	1	1	CO
denovo42	1	1	CR8
denovo420	1	1	CR8

denovo421	1	5	CR8
denovo423	1	1	CO
denovo424	1	2	CO
denovo425	1	28	CO
denovo426	1	1	CO
denovo427	1	4	CR5
denovo428	1	1	CO
denovo429	2	2	CO,UR
denovo43	1	1	UR
denovo430	1	4	CO
denovo431	3	13	CO,CR8,CR5
denovo432	2	6	CO,CR5
denovo433	1	1	CR8
denovo434	2	2	CO,CR8
denovo435	1	1	CO
denovo436	1	1	CR8
denovo437	2	8	CR8,CR5
denovo438	1	1	CR8
denovo439	1	2	UR
denovo44	1	1	CO
denovo440	1	1	CR5
denovo441	2	2	CO,CR5
denovo442	1	1	CO
denovo443	3	4	CO,UR,CR8
denovo444	1	3	CO
denovo445	1	2	CR8
denovo446	2	4	CR8,CR5
denovo447	1	2	CO
denovo448	1	4	CR5
denovo449	1	1	CR8
denovo45	3	5	CO,CR8,CR5
denovo450	1	5	CR8
denovo451	1	13	CO
denovo452	1	1	CO
denovo454	3	5	CO,UR,CR8
denovo455	1	1	CO
denovo456	1	4	CO
denovo457	2	5	CO,CR8
denovo458	3	3	CO,CR8,CR5
denovo459	1	3	CO
denovo46	1	1	CO
denovo460	1	1	CO
denovo461	1	1	CO
denovo462	1	1	CO
denovo463	1	2	CR8
denovo464	1	3	UR
denovo466	1	1	CO
denovo468	1	2	UR
denovo469	1	1	CO
denovo470	2	2	UR,CR5
denovo471	3	6	CO,CR8,CR5
denovo472	1	1	CR5
denovo473	1	2	CO
denovo474	1	1	CO
denovo475	1	1	CR8
denovo476	1	1	CR5
denovo477	1	1	CO
denovo478	1	1	CR5
denovo479	1	1	CO
denovo48	1	1	CR8
denovo480	1	1	CR5
denovo481	1	1	CR5
denovo482	1	1	UR
denovo483	1	1	CR8
denovo484	1	1	CR5
denovo485	3	3	UR,CR8,CR5
denovo486	1	1	CR5

denovo487	1	63	CR5
denovo488	1	7	CR8
denovo489	3	3	UR,CR8,CR5
denovo49	1	2	CO
denovo490	1	1	CR8
denovo491	1	4	CO
denovo492	4	176	CO,UR,CR8,CR5
denovo493	1	1	CR8
denovo494	2	2	CR8,CR5
denovo495	1	1	CR5
denovo496	1	1	CO
denovo497	1	1	CO
denovo498	3	10	CO,CR8,CR5
denovo499	1	1	UR
denovo5	2	6	CO,CR5
denovo50	1	1	CO
denovo500	1	7	CR5
denovo501	2	2	CO,CR8
denovo502	4	7	CO,UR,CR8,CR5
denovo504	2	2	CO,CR5
denovo505	1	1	UR
denovo506	1	1	UR
denovo508	2	2	CO,CR8
denovo509	1	1	CR5
denovo51	1	1	CR5
denovo510	1	1	UR
denovo511	2	11	CR8,CR5
denovo512	1	1	CO
denovo513	1	5	CO
denovo514	1	2	UR
denovo515	3	6	CO,CR8,CR5
denovo516	1	4	CO
denovo517	1	2	CR5
denovo518	1	1	CR8
denovo519	1	1	CR8
denovo52	1	1	CO
denovo520	3	4	CO,UR,CR5
denovo521	2	2	CR8,CR5
denovo522	1	1	CO
denovo524	2	3	CO,UR
denovo525	1	1	CO
denovo526	1	1	CO
denovo527	1	50	CO
denovo528	2	9	CR8,CR5
denovo529	1	1	CO
denovo53	1	1	CO
denovo530	4	22	CO,UR,CR8,CR5
denovo531	1	1	CO
denovo532	1	2	CR8
denovo533	1	1	CO
denovo534	1	2	CR8
denovo535	1	1	CO
denovo537	1	3	CO
denovo538	1	2	CR8
denovo54	1	1	CR5
denovo540	3	128	CO,CR8,CR5
denovo541	1	1	CR8
denovo542	4	208	CO,UR,CR8,CR5
denovo543	1	1	CO
denovo544	1	1	CR8
denovo545	1	1	CR8
denovo546	1	1	CO
denovo547	1	1	CO
denovo548	3	3	CO,UR,CR8
denovo550	1	1	CR5
denovo551	1	16	CO
denovo553	1	1	CR5

denovo554	1	1	CO
denovo555	2	3	CR8,CR5
denovo557	1	1	CR8
denovo559	1	1	CR5
denovo56	4	7	CO,UR,CR8,CR5
denovo560	1	1	CO
denovo561	1	3	CR5
denovo562	1	1	CO
denovo563	1	1	CO
denovo564	1	2	CR8
denovo565	1	1	CR5
denovo566	1	3	CR5
denovo567	1	1	CR5
denovo568	2	7	UR,CR8
denovo569	1	1	CR5
denovo57	3	4	CO,UR,CR5
denovo570	1	1	CR5
denovo571	1	1	CO
denovo572	1	1	CR5
denovo573	1	1	CO
denovo574	2	2	CO,CR5
denovo575	2	3	CO,CR8
denovo576	1	3	CO
denovo577	1	1	CR8
denovo578	1	1	CR8
denovo579	1	1	CO
denovo58	2	4	CO,UR
denovo580	1	1	CO
denovo581	1	1	CO
denovo582	1	2	CO
denovo583	1	24	CO
denovo584	2	4	CO,CR8
denovo585	1	1	CO
denovo586	2	3	CO,CR5
denovo587	2	2	CO,CR5
denovo588	1	1	CR8
denovo589	1	1	CO
denovo59	1	2	CR5
denovo591	1	1	CR8
denovo592	4	28	CO,UR,CR8,CR5
denovo593	2	6	CO,CR8
denovo594	2	2	CR8,CR5
denovo595	3	3	UR,CR8,CR5
denovo596	2	3	CO,UR
denovo597	1	1	CO
denovo598	1	6	CO
denovo599	1	1	CR8
denovo6	1	7	CO
denovo60	2	7	CO,CR8
denovo600	4	55	CO,UR,CR8,CR5
denovo601	2	2	CR8,CR5
denovo602	1	2	CR8
denovo603	1	1	CR5
denovo604	1	1	CR5
denovo605	4	22	CO,UR,CR8,CR5
denovo606	1	1	CR8
denovo607	2	4	CO,CR5
denovo608	1	1	CR5
denovo609	1	36	CO
denovo61	2	3	UR,CR5
denovo610	1	3	CR5
denovo611	1	1	CO
denovo612	1	1	CR8
denovo613	1	1	CR5
denovo614	1	1	CO
denovo615	1	1	CO
denovo616	2	2	CR8,CR5

denovo617	1	2	CO
denovo618	2	5	CR8,CR5
denovo619	1	5	CO
denovo62	1	7	CO
denovo620	1	1	CR5
denovo621	2	4	CO,CR5
denovo622	1	2	CR8
denovo624	4	15	CO,UR,CR8,CR5
denovo625	1	1	CO
denovo626	2	5	CO,UR
denovo627	1	5	CO
denovo628	3	9	CO,UR,CR8
denovo629	1	2	CO
denovo63	4	78	CO,UR,CR8,CR5
denovo630	2	2	UR,CR8
denovo631	2	7	UR,CR5
denovo632	1	1	UR
denovo633	1	4	UR
denovo634	1	1	CR8
denovo635	1	1	UR
denovo636	1	2	CR8
denovo637	1	2	CO
denovo638	1	2	CR8
denovo639	4	7	CO,UR,CR8,CR5
denovo64	3	5	CO,UR,CR5
denovo640	2	2	UR,CR5
denovo641	2	3	CO,CR8
denovo642	1	1	UR
denovo643	1	1	CO
denovo644	1	1	CR8
denovo645	1	1	CR8
denovo646	1	1	CR8
denovo647	1	1	CO
denovo648	2	3	CR8,CR5
denovo649	1	1	CO
denovo65	3	11	CO,UR,CR8
denovo650	1	1	CR8
denovo651	2	2	CR8,CR5
denovo652	1	1	CR8
denovo653	1	1	CO
denovo654	2	7	CO,CR5
denovo655	4	107	CO,UR,CR8,CR5
denovo656	1	1	CR5
denovo657	3	12	CO,CR8,CR5
denovo658	2	3	CO,CR5
denovo66	1	6	CR8
denovo660	1	2	CR8
denovo661	4	14	CO,UR,CR8,CR5
denovo662	4	10	CO,UR,CR8,CR5
denovo663	2	6	CO,CR8
denovo664	1	1	CR5
denovo665	3	12	CO,UR,CR8
denovo666	3	6	UR,CR8,CR5
denovo667	4	6	CO,UR,CR8,CR5
denovo668	3	4	CO,UR,CR5
denovo669	1	1	CR5
denovo67	1	1	CR5
denovo672	1	1	UR
denovo673	1	1	CR5
denovo674	1	3	CO
denovo675	2	2	CO,CR5
denovo676	1	1	CO
denovo677	1	3	CO
denovo678	1	1	CR5
denovo679	3	5	CO,CR8,CR5
denovo68	1	1	CO
denovo680	1	1	CO

denovo681	1	8	CO
denovo683	1	2	CO
denovo684	1	1	CO
denovo685	1	1	CO
denovo686	1	1	CO
denovo687	1	2	CR8
denovo688	1	1	CR5
denovo689	1	4	CR8
denovo69	1	1	CO
denovo690	1	1	CO
denovo691	2	4	CO,CR8
denovo692	1	1	CR8
denovo693	2	3	CR8,CR5
denovo694	2	2	CR8,CR5
denovo695	1	2	CR8
denovo696	3	8	CO,CR8,CR5
denovo697	1	1	CO
denovo698	1	3	CR5
denovo699	4	55	CO,UR,CR8,CR5
denovo7	1	1	CO
denovo70	3	20	CO,CR8,CR5
denovo700	2	3	UR,CR8
denovo701	2	3	CO,CR5
denovo702	1	1	CO
denovo703	3	6	CO,CR8,CR5
denovo704	1	2	CO
denovo705	1	1	CO
denovo706	1	1	CR5
denovo707	1	1	CO
denovo708	1	17	CO
denovo709	4	39	CO,UR,CR8,CR5
denovo71	3	12	CO,CR8,CR5
denovo710	1	2	UR
denovo713	2	3	UR,CR5
denovo714	1	1	CR5
denovo715	1	2	CR5
denovo716	2	2	CO,CR5
denovo717	1	2	CO
denovo718	1	2	CO
denovo719	2	3	CO,CR5
denovo72	1	1	CO
denovo720	1	39	CO
denovo721	1	3	CO
denovo722	1	1	CR8
denovo723	1	1	CR5
denovo724	1	1	CR5
denovo725	2	5	CR8,CR5
denovo728	2	7	CO,CR5
denovo729	3	11	CO,UR,CR8
denovo73	2	2	CO,CR5
denovo730	1	1	CO
denovo731	1	1	CR8
denovo732	1	1	CR5
denovo733	2	6	CO,UR
denovo734	4	5	CO,UR,CR8,CR5
denovo735	1	2	CO
denovo736	1	1	CR5
denovo737	1	1	CO
denovo738	1	1	CO
denovo739	1	1	CR5
denovo74	3	13	CO,CR8,CR5
denovo740	2	4	CO,CR5
denovo742	2	2	CR8,CR5
denovo743	1	1	CO
denovo744	1	1	CO
denovo745	1	1	CO
denovo746	1	19	CR5

denovo747	1	1	CR5
denovo748	4	23	CO,UR,CR8,CR5
denovo749	1	1	CR5
denovo75	3	11	CO,UR,CR8
denovo750	1	1	CR5
denovo752	1	1	CR5
denovo753	1	2	CO
denovo754	1	1	CR8
denovo755	2	3	CO,CR8
denovo756	1	2	CR8
denovo758	2	3	CO,CR8
denovo759	2	3	CO,CR8
denovo76	2	3	CO,CR5
denovo760	2	2	UR,CR8
denovo761	2	3	CO,CR8
denovo762	1	1	CO
denovo763	3	10	CO,UR,CR5
denovo765	1	1	CO
denovo766	3	7	CO,CR8,CR5
denovo767	2	3	CR8,CR5
denovo768	1	1	CO
denovo769	1	1	CR5
denovo77	1	1	CR8
denovo770	4	235	CO,UR,CR8,CR5
denovo771	3	5	CO,CR8,CR5
denovo772	1	1	CO
denovo773	1	1	CR8
denovo774	2	2	CO,CR8
denovo775	1	1	CO
denovo776	1	4	CR5
denovo777	3	12	CO,UR,CR5
denovo778	1	1	UR
denovo779	1	2	CO
denovo78	1	1	CO
denovo780	1	2	CO
denovo781	1	1	CO
denovo782	1	1	CO
denovo783	3	6	CO,CR8,CR5
denovo784	1	3	CR8
denovo785	1	30	CO
denovo786	3	20	CO,CR8,CR5
denovo787	2	3	CO,CR8
denovo788	1	1	CO
denovo789	1	2	CR8
denovo79	4	62	CO,UR,CR8,CR5
denovo790	3	13	CO,CR8,CR5
denovo791	1	28	CO
denovo792	1	7	CO
denovo794	1	1	CO
denovo795	2	17	CO,CR8
denovo796	3	9	CO,CR8,CR5
denovo797	1	1	CR8
denovo798	1	1	UR
denovo799	3	6	CO,CR8,CR5
denovo80	1	1	CO
denovo800	1	1	CO
denovo801	2	6	CO,CR5
denovo802	1	1	CO
denovo803	4	24	CO,UR,CR8,CR5
denovo804	1	1	CO
denovo805	1	1	UR
denovo806	3	15	CO,CR8,CR5
denovo807	4	18	CO,UR,CR8,CR5
denovo808	1	1	UR
denovo809	1	2	CR5
denovo81	2	4	CR8,CR5
denovo810	1	1	CO

denovo811	1	2	CR8
denovo812	1	65	UR
denovo813	1	1	UR
denovo815	1	1	CO
denovo816	2	4	CO,CR5
denovo817	1	2	CO
denovo818	2	5	CR8,CR5
denovo819	1	1	CR8
denovo82	1	2	CO
denovo820	1	1	CR5
denovo821	3	10	CO,CR8,CR5
denovo822	1	2	CR5
denovo823	1	1	CR5
denovo824	1	14	CR8
denovo825	1	1	CR5
denovo826	1	1	CR5
denovo827	1	1	CO
denovo828	1	2	UR
denovo829	4	41	CO,UR,CR8,CR5
denovo83	2	4	UR,CR8
denovo830	1	3	CO
denovo831	3	3	CO,UR,CR8
denovo832	1	2	CR8
denovo833	1	2	CO
denovo834	3	18	CO,CR8,CR5
denovo836	4	31	CO,UR,CR8,CR5
denovo837	1	1	CO
denovo838	2	2	CO,UR
denovo839	1	1	CR5
denovo84	1	2	CR5
denovo840	1	1	UR
denovo841	3	12	CO,UR,CR5
denovo842	1	1	UR
denovo843	1	1	CR5
denovo844	4	10	CO,UR,CR8,CR5
denovo845	1	13	UR
denovo847	1	12	CO
denovo848	1	3	CR5
denovo849	1	1	CO
denovo85	1	1	CO
denovo850	2	4	CO,CR5
denovo851	1	8	CO
denovo852	1	2	CR8
denovo853	3	40	CO,CR8,CR5
denovo854	1	1	CR5
denovo855	1	1	CO
denovo856	1	1	CR5
denovo857	1	1	CR5
denovo858	2	45	CO,UR
denovo859	1	4	CO
denovo86	1	3	CO
denovo860	1	1	CO
denovo861	1	1	CR8
denovo862	2	7	CO,CR5
denovo863	1	1	CO
denovo864	1	1	CO
denovo865	1	2	CR8
denovo866	2	4	CO,CR8
denovo867	1	1	CR5
denovo868	4	14	CO,UR,CR8,CR5
denovo869	1	1	CR8
denovo87	2	3	CO,UR
denovo870	1	2	CR5
denovo871	1	1	CR8
denovo872	2	2	CO,CR5
denovo873	2	3	CO,CR5
denovo874	3	5	CO,UR,CR5

denovo875	1	2	CR8
denovo876	2	3	CO,CR8
denovo877	1	4	CO
denovo878	4	41	CO,UR,CR8,CR5
denovo879	1	1	CR8
denovo88	1	2	CR5
denovo880	4	32	CO,UR,CR8,CR5
denovo881	1	1	UR
denovo882	3	7	CO,UR,CR5
denovo883	1	4	CR8
denovo885	4	37	CO,UR,CR8,CR5
denovo886	3	12	UR,CR8,CR5
denovo887	2	5	CO,CR8
denovo888	1	2	UR
denovo889	1	5	CR5
denovo89	1	1	CR5
denovo890	1	1	CR5
denovo891	1	1	CR8
denovo892	1	1	CR8
denovo893	1	5	UR
denovo894	1	1	UR
denovo895	1	1	UR
denovo896	3	11	UR,CR8,CR5
denovo897	3	26	CO,CR8,CR5
denovo899	1	1	CO
denovo9	1	1	UR
denovo901	1	2	CO
denovo902	3	28	CO,CR8,CR5
denovo903	1	1	CO
denovo904	1	1	CR8
denovo905	3	3	CO,CR8,CR5
denovo906	1	1	CO
denovo907	2	3	CO,CR8
denovo908	1	1	CR8
denovo909	1	2	CR8
denovo91	3	6	CO,UR,CR5
denovo910	1	1	CO
denovo911	3	4	CO,UR,CR8
denovo912	1	2	CO
denovo913	1	1	CO
denovo915	1	3	CO
denovo916	1	1	CO
denovo917	1	1	CO
denovo918	3	10	CO,CR8,CR5
denovo919	1	3	CR8
denovo92	1	9	UR
denovo920	1	2	CO
denovo921	1	2	CR8
denovo922	3	3	CO,CR8,CR5
denovo923	2	2	CR8,CR5
denovo924	1	1	UR
denovo925	1	1	CR5
denovo926	1	1	CR5
denovo927	1	2	CR8
denovo928	3	28	CO,CR8,CR5
denovo929	2	2	CO,CR8
denovo93	1	11	CR8
denovo930	3	4	CO,CR8,CR5
denovo931	1	1	CO
denovo933	1	1	CR5
denovo934	2	2	CO,CR5
denovo935	4	22	CO,UR,CR8,CR5
denovo936	1	4	CO
denovo937	1	2	CR5
denovo938	2	8	CR8,CR5
denovo939	1	58	CO
denovo940	1	2	CO

denovo941	1	1	CO
denovo942	1	1	UR
denovo943	1	1	CR5
denovo944	1	48	CO
denovo945	1	2	CO
denovo946	2	8	CO,CR8
denovo947	3	11	CO,CR8,CR5
denovo948	1	3	CO
denovo949	2	6	CO,CR8
denovo95	4	8	CO,UR,CR8,CR5
denovo950	1	1	CR5
denovo951	1	1	CO
denovo952	1	1	CR8
denovo953	4	22	CO,UR,CR8,CR5
denovo954	1	1	CO
denovo955	4	6	CO,UR,CR8,CR5
denovo956	1	2	CR8
denovo957	1	6	CR8
denovo958	1	1	CO
denovo96	4	32	CO,UR,CR8,CR5
denovo960	2	2	CO,CR8
denovo961	1	1	CO
denovo963	3	10	CO,CR8,CR5
denovo964	4	14	CO,UR,CR8,CR5
denovo965	2	4	UR,CR5
denovo966	1	1	CO
denovo967	2	3	CO,CR5
denovo968	1	18	CO
denovo969	1	1	CO
denovo97	2	2	CO,CR5
denovo970	1	1	CO
denovo971	1	1	UR
denovo972	1	1	CO
denovo973	3	24	CO,CR8,CR5
denovo974	1	1	CR8
denovo975	1	1	CO
denovo977	3	9	CO,UR,CR8
denovo978	1	1	CR8
denovo979	2	7	CO,CR8
denovo98	1	3	CR8
denovo980	3	6	CO,UR,CR8
denovo981	3	19	CO,CR8,CR5
denovo982	1	2	CO
denovo983	1	1	CR5
denovo984	1	10	CO
denovo985	1	1	CO
denovo987	1	1	CO
denovo988	1	6	CO
denovo989	1	1	CO
denovo99	2	2	CO,CR8
denovo990	1	1	CO
denovo991	1	1	CR5
denovo992	1	2	CR8
denovo993	1	1	CO
denovo994	1	1	CO
denovo995	1	2	CO
denovo996	1	7	CO
denovo997	3	7	CO,UR,CR5
denovo998	1	1	CO
denovo999	2	3	UR,CR8
MID1	725	3619	
MID2	234	834	
MID3	486	1659	
MID4	455	1837	
MID5	0	0	

Table S9.2. real edge table for pOTU network (based on pyrotagsequencing data) and visualization in Cytoscape

from	to	eweight	consensus_lin
MID1	denovo10	1	k__Bacteria:p__Planctomycetes:c__Planctomycetia:o__Pirellulales:f__Pirellulaceae:g__
MID1	denovo100	14	k__Bacteria:p__Actinobacteria:c__Actinobacteria:o__Actinomycetales:f__Microbacteriaceae:g__Leucobacter
MID1	denovo1001	1	k__Bacteria:p__Proteobacteria:c__Alphaproteobacteria:o__Rhodobacterales:f__Rhodobacteraceae:g__Rubellimicrobium
MID1	denovo1003	8	k__Bacteria:p__Chloroflexi:c__Thermomicrobia:o__JG30-KF-CM45:f__:g__
MID1	denovo1004	1	k__Bacteria:p__Actinobacteria:c__Actinobacteria:o__Actinomycetales:f__Nocardioidaceae:g__Aeromicrobium
MID1	denovo1006	2	k__Bacteria:p__Bacteroidetes:c__Flavobacteriia:o__Flavobacteriales:f__[Weeksellaceae]:g__Chryseobacterium
MID1	denovo1007	1	k__Bacteria:p__Armatimonadetes:c__Armatimonadia:o__Armatimonadales:f__Armatimonadaceae:g__
MID1	denovo1008	3	k__Bacteria:p__Actinobacteria:c__Actinobacteria:o__Actinomycetales:f__Micromonosporaceae:g__
MID1	denovo1009	1	k__Bacteria:p__Acidobacteria:c__[Chloracidobacteria]:o__RB41:f__Ellin6075:g__
MID1	denovo1010	1	k__Bacteria:p__Firmicutes:c__Bacilli:o__Bacillales:f__Bacillaceae:g__Bacillus
MID1	denovo1012	1	k__Bacteria:p__Proteobacteria:c__Alphaproteobacteria:o__Rhizobiales:f__Bradyrhizobiaceae:g__
MID1	denovo1013	3	k__Bacteria:p__Cyanobacteria:c__Nostocophycideae:o__Nostocales:f__Nostocaceae
MID1	denovo1014	9	k__Bacteria:p__Proteobacteria:c__Alphaproteobacteria:o__Rhizobiales:f__Bradyrhizobiaceae:g__Balneimonas
MID1	denovo1016	1	k__Bacteria:p__Firmicutes:c__Clostridia:o__Clostridiales:f__Lachnospiraceae:g__[Ruminococcus]
MID1	denovo1017	3	k__Bacteria:p__Cyanobacteria:c__Synechococcophycideae:o__Pseudanabaenales:f__Pseudanabaenaceae:g__Leptolyngbya
MID1	denovo1018	3	k__Bacteria:p__Firmicutes:c__Clostridia:o__Clostridiales:f__[Tissierellaceae]:g__Peptoniphilus
MID1	denovo102	1	k__Bacteria:p__Proteobacteria:c__Betaproteobacteria:o__Burkholderiales:f__Oxalobacteraceae:g__
MID1	denovo1021	1	k__Bacteria:p__Acidobacteria:c__Solibacteres:o__Solibacterales:f__Solibacteraceae:g__
MID1	denovo1023	7	k__Bacteria:p__Planctomycetes:c__Planctomycetia:o__Pirellulales:f__Pirellulaceae:g__
MID1	denovo1024	6	k__Bacteria:p__Proteobacteria:c__Betaproteobacteria:o__Burkholderiales:f__Comamonadaceae:g__
MID1	denovo1027	13	k__Bacteria:p__Cyanobacteria:c__Nostocophycideae:o__Nostocales:f__Nostocaceae:g__Nostoc
MID1	denovo1030	1	k__Bacteria:p__Actinobacteria:c__Acidimicrobiia:o__Acidimicrobiales:f__:g__
MID1	denovo1032	1	k__Bacteria:p__Firmicutes:c__Bacilli:o__Lactobacillales:f__:g__
MID1	denovo1035	1	k__Bacteria:p__Actinobacteria:c__Actinobacteria:o__Actinomycetales:f__Micrococcaceae:g__
MID1	denovo1037	2	k__Bacteria:p__Actinobacteria:c__Actinobacteria:o__Actinomycetales:f__Corynebacteriaceae:g__Corynebacterium
MID1	denovo104	1	k__Bacteria:p__Proteobacteria:c__Gammaproteobacteria:o__Alteromonadales:f__Alteromonadaceae:g__Cellvibrio
MID1	denovo1040	1	k__Bacteria:p__Proteobacteria:c__Alphaproteobacteria:o__Rickettsiales:f__Rickettsiaceae:g__Rickettsia
MID1	denovo1042	2	k__Bacteria:p__Firmicutes:c__Clostridia:o__Clostridiales:f__Ruminococcaceae:g__
MID1	denovo1046	14	k__Bacteria:p__Proteobacteria:c__Betaproteobacteria:o__Burkholderiales:f__Comamonadaceae:g__
MID1	denovo1048	1	k__Bacteria:p__Firmicutes:c__Clostridia:o__Clostridiales:f__Lachnospiraceae:g__
MID1	denovo1054	3	k__Bacteria:p__Firmicutes:c__Bacilli:o__Bacillales:f__Bacillaceae:g__Bacillus
MID1	denovo1055	1	k__Bacteria:p__Proteobacteria:c__Alphaproteobacteria:o__Sphingomonadales:f__Sphingomonadaceae
MID1	denovo1056	3	k__Bacteria:p__Actinobacteria:c__Actinobacteria:o__Actinomycetales:f__Nocardioidaceae:g__Propionicimonas
MID1	denovo1057	2	k__Bacteria:p__Actinobacteria:c__Acidimicrobiia:o__Acidimicrobiales:f__Iamiaceae:g__Iamia
MID1	denovo1058	1	k__Bacteria:p__Proteobacteria:c__Alphaproteobacteria:o__Rhodospirillales:f__Acetobacteraceae:g__
MID1	denovo1059	10	k__Bacteria:p__Actinobacteria:c__Actinobacteria:o__Actinomycetales:f__Microbacteriaceae:g__Microbacterium
MID1	denovo106	4	k__Bacteria:p__Firmicutes:c__Clostridia:o__Clostridiales:f__Lachnospiraceae
MID1	denovo1063	3	k__Bacteria:p__Planctomycetes:c__Phycisphaerae:o__WD2101:f__:g__
MID1	denovo1065	1	k__Bacteria:p__Gemmatimonadetes:c__Gemmatimonadetes:o__Gemmatimonadales:f__:g__
MID1	denovo1066	1	k__Bacteria:p__Gemmatimonadetes:c__Gemmatimonadetes:o__:f__:g__
MID1	denovo1067	2	k__Bacteria:p__Proteobacteria:c__Alphaproteobacteria:o__Rhizobiales:f__Beijerinckiaceae:g__
MID1	denovo107	2	k__Bacteria:p__Proteobacteria:c__Alphaproteobacteria:o__Rhodobacterales:f__Rhodobacteraceae:g__Rubellimicrobium
MID1	denovo1072	1	k__Bacteria:p__Firmicutes:c__Clostridia:o__Clostridiales:f__Lachnospiraceae:g__
MID1	denovo1073	1	k__Bacteria:p__Proteobacteria:c__Gammaproteobacteria:o__Xanthomonadales:f__Xanthomonadaceae:g__Stenotrophomonas
MID1	denovo1075	1	k__Bacteria:p__Acidobacteria:c__[Chloracidobacteria]:o__DS-100:f__:g__
MID1	denovo1077	8	k__Bacteria:p__Proteobacteria:c__Alphaproteobacteria:o__Rhizobiales:f__Hyphomicrobiaceae:g__Devosia
MID1	denovo1078	2	k__Bacteria:p__Acidobacteria:c__Acidobacteria-6:o__iii1-15:f__:g__
MID1	denovo1080	5	k__Bacteria:p__Gemmatimonadetes:c__Gemmatimonadetes:o__Gemmatimonadales:f__:g__
MID1	denovo1081	1	k__Bacteria:p__Cyanobacteria:c__4C0d-2:o__SM1D11:f__:g__
MID1	denovo1083	4	k__Bacteria:p__Chloroflexi:c__Thermomicrobia:o__JG30-KF-CM45:f__:g__
MID1	denovo1084	6	k__Bacteria:p__Proteobacteria:c__Alphaproteobacteria:o__Caulobacterales:f__Caulobacteraceae:g__
MID1	denovo1086	3	k__Bacteria:p__Actinobacteria:c__Actinobacteria:o__Actinomycetales:f__Nocardioidaceae:g__
MID1	denovo1088	1	k__Bacteria:p__Actinobacteria:c__Actinobacteria:o__Actinomycetales:f__Kineosporiaceae:g__
MID1	denovo109	1	k__Bacteria:p__Proteobacteria:c__Gammaproteobacteria:o__Enterobacteriales:f__Enterobacteriaceae
MID1	denovo1091	1	k__Bacteria:p__Proteobacteria:c__Betaproteobacteria:o__Burkholderiales:f__Comamonadaceae:g__
MID1	denovo1093	3	k__Bacteria:p__Firmicutes:c__Bacilli:o__Lactobacillales:f__Lactobacillaceae:g__Lactobacillus
MID1	denovo1094	7	k__Bacteria:p__Firmicutes:c__Bacilli:o__Lactobacillales:f__Aerococcaceae:g__Facklamia
MID1	denovo1098	1	k__Bacteria:p__Actinobacteria:c__Actinobacteria:o__Actinomycetales:f__Nocardioidaceae:g__
MID1	denovo11	1	k__Bacteria:p__Acidobacteria:c__Solibacteres:o__Solibacterales:f__:g__
MID1	denovo1100	2	k__Bacteria:p__Firmicutes:c__Clostridia:o__Clostridiales:f__Lachnospiraceae:g__
MID1	denovo1101	1	k__Bacteria:p__Proteobacteria:c__Alphaproteobacteria:o__Rhodobacterales:f__Rhodobacteraceae:g__Rubellimicrobium
MID1	denovo1102	1	k__Bacteria:p__Proteobacteria:c__Alphaproteobacteria:o__Rhodospirillales:f__Rhodospirillaceae:g__
MID1	denovo1103	4	k__Bacteria:p__Gemmatimonadetes:c__Gemmatimonadetes:o__Gemmatimonadales:f__:g__

MID1	denovo1109	83	k__Bacteria:p__Proteobacteria:c__Alphaproteobacteria:o__Rhodobacterales:f__Rhodobacteraceae
MID1	denovo111	1	k__Bacteria:p__Proteobacteria:c__Alphaproteobacteria:o__Rhizobiales:f__Phyllobacteriaceae
MID1	denovo1112	2	k__Bacteria:p__Proteobacteria:c__Alphaproteobacteria:o__Rhizobiales:f__Brucellaceae
MID1	denovo1113	1	k__Bacteria:p__Proteobacteria:c__Gammaproteobacteria:o__Xanthomonadales:f__Xanthomonadaceae:g__
MID1	denovo1114	1	k__Bacteria:p__Proteobacteria:c__Alphaproteobacteria:o__Rhizobiales:f__Bradyrhizobiaceae:g__
MID1	denovo1118	1	k__Bacteria:p__Firmicutes:c__Bacilli:o__Bacillales:f__Paenibacillaceae:g__
MID1	denovo1119	1	k__Bacteria:p__Proteobacteria:c__Alphaproteobacteria:o__Rhodobacterales:f__Rhodobacteraceae:g__Amaricoccus
MID1	denovo1121	3	k__Bacteria:p__Bacteroidetes:c__Flavobacteriia:o__Flavobacteriales:f__[Weeksellaceae]:g__Chryseobacterium
MID1	denovo1122	99	k__Bacteria:p__Proteobacteria:c__Gammaproteobacteria:o__Legionellales:f__Coxiellaceae:g__Rickettsiella
MID1	denovo1124	107	k__Bacteria:p__Proteobacteria:c__Betaproteobacteria:o__Rhodocyclales:f__Rhodocyclaceae:g__Thauera
MID1	denovo1131	1	k__Bacteria:p__Actinobacteria:c__Actinobacteria:o__Actinomycetales:f__Sanguibacteraceae:g__Sanguibacter
MID1	denovo1132	1	k__Bacteria:p__Firmicutes:c__Bacilli:o__Lactobacillales:f__Enterococcaceae:g__Vagococcus
MID1	denovo1133	1	k__Bacteria:p__Firmicutes:c__Bacilli:o__Bacillales:f__Planococcaceae:g__Sporosarcina
MID1	denovo1134	2	k__Bacteria:p__Proteobacteria:c__Alphaproteobacteria:o__Sphingomonadales:f__Sphingomonadaceae:g__
MID1	denovo1135	1	k__Bacteria:p__Actinobacteria:c__Acidimicrobiia:o__Acidimicrobiales:f__:g__
MID1	denovo1136	1	k__Bacteria:p__Firmicutes:c__Clostridia:o__Clostridiales:f__[Tissierellaceae]:g__Parvimonas
MID1	denovo114	3	k__Bacteria:p__Cyanobacteria:c__Chloroplast:o__Chlorophyta:f__:g__
MID1	denovo1140	1	k__Bacteria:p__[Thermi]:c__Deinococci:o__Deinococcales:f__Deinococcaceae:g__Deinococcus
MID1	denovo1143	1	k__Bacteria:p__Proteobacteria:c__Alphaproteobacteria:o__Rhizobiales:f__Brucellaceae
MID1	denovo1144	1	k__Bacteria:p__Gemmatimonadetes:c__Gemmatimonadetes
MID1	denovo1149	6	k__Bacteria:p__Proteobacteria:c__Alphaproteobacteria:o__Rhizobiales:f__Methylobacteriaceae:g__Methylobacterium
MID1	denovo115	3	k__Bacteria:p__Gemmatimonadetes:c__Gemmatimonadetes:o__Gemmatimonadales:f__:g__
MID1	denovo1150	3	k__Bacteria:p__Firmicutes:c__Clostridia:o__Clostridiales:f__Lachnospiraceae:g__Roseburia
MID1	denovo1151	1	k__Bacteria:p__Firmicutes:c__Bacilli:o__Bacillales:f__Planococcaceae:g__Solibacillus
MID1	denovo1153	3	k__Bacteria:p__Cyanobacteria:c__Nostocophycideae:o__Stigonematales:f__Rivulariaceae:g__Calothrix
MID1	denovo1154	2	k__Bacteria:p__Proteobacteria:c__Betaproteobacteria:o__Burkholderiales:f__Alcaligenaceae:g__Pigmentiphaga
MID1	denovo1157	1	k__Bacteria:p__Proteobacteria:c__Alphaproteobacteria:o__Rhodobacterales:f__Rhodobacteraceae:g__Amaricoccus
MID1	denovo1162	1	k__Bacteria:p__Proteobacteria:c__Alphaproteobacteria:o__Sphingomonadales:f__Sphingomonadaceae:g__
MID1	denovo1164	2	k__Bacteria:p__Bacteroidetes:c__Cytophagia:o__Cytophagales:f__Cytophagaceae:g__
MID1	denovo1165	2	k__Bacteria:p__Firmicutes:c__Clostridia:o__Clostridiales:f__[Tissierellaceae]:g__Anaerococcus
MID1	denovo1166	2	k__Bacteria:p__Proteobacteria:c__Gammaproteobacteria:o__Xanthomonadales:f__Sinobacteraceae:g__
MID1	denovo1168	2	k__Bacteria:p__Proteobacteria:c__Deltaproteobacteria:o__Myxococcales:f__:g__
MID1	denovo1169	1	k__Bacteria:p__Cyanobacteria:c__Synechococcophycideae:o__Pseudanabaenales:f__Pseudanabaenaceae:g__Leptolyngbya
MID1	denovo1170	1	k__Bacteria:p__Actinobacteria:c__Actinobacteria:o__Actinomycetales:f__Dermacoccaceae:g__Dermacoccus
MID1	denovo1173	39	k__Bacteria:p__Proteobacteria:c__Alphaproteobacteria:o__Rhodobacterales:f__Rhodobacteraceae:g__Paracoccus
MID1	denovo1174	1	Unassigned
MID1	denovo1175	2	k__Bacteria:p__Proteobacteria:c__Alphaproteobacteria:o__Rhizobiales:f__Hyphomicrobiaceae:g__Hyphomicrobium
MID1	denovo1178	4	k__Bacteria:p__Firmicutes:c__Clostridia:o__Clostridiales:f__Lachnospiraceae:g__
MID1	denovo1179	4	k__Bacteria:p__Cyanobacteria:c__Synechococcophycideae:o__Pseudanabaenales:f__Pseudanabaenaceae:g__Leptolyngbya
MID1	denovo118	1	k__Bacteria:p__Acidobacteria:c__Solibacteres:o__Solibacterales:f__:g__
MID1	denovo1181	2	k__Bacteria:p__Firmicutes:c__Bacilli:o__Bacillales:f__:g__
MID1	denovo1182	1	k__Bacteria:p__Proteobacteria:c__Alphaproteobacteria:o__Rhodospirillales:f__Acetobacteraceae:g__
MID1	denovo1183	2	k__Bacteria:p__Actinobacteria:c__Actinobacteria:o__Actinomycetales:f__:g__
MID1	denovo1184	3	k__Bacteria:p__Proteobacteria:c__Alphaproteobacteria:o__Rhizobiales:f__Phyllobacteriaceae:g__
MID1	denovo1187	3	k__Bacteria:p__Proteobacteria:c__Alphaproteobacteria:o__Rhodospirillales:f__Acetobacteraceae:g__
MID1	denovo1193	1	k__Bacteria:p__Proteobacteria:c__Alphaproteobacteria:o__Rhodobacterales:f__Rhodobacteraceae:g__Rubellimicrobium
MID1	denovo1194	2	k__Bacteria:p__Actinobacteria:c__Acidimicrobiia:o__Acidimicrobiales:f__C111:g__
MID1	denovo1195	1	k__Bacteria:p__Proteobacteria:c__Alphaproteobacteria:o__Rhizobiales:f__Phyllobacteriaceae:g__Aminobacter
MID1	denovo1197	5	k__Bacteria:p__Proteobacteria:c__Betaproteobacteria:o__Burkholderiales:f__Burkholderiaceae:g__
MID1	denovo1198	1	k__Bacteria:p__Cyanobacteria:c__Synechococcophycideae:o__Pseudanabaenales:f__Pseudanabaenaceae:g__Leptolyngbya
MID1	denovo1199	1	k__Bacteria:p__Actinobacteria:c__Acidimicrobiia:o__Acidimicrobiales:f__C111:g__
MID1	denovo12	1	k__Bacteria:p__Bacteroidetes:c__Sphingobacteriia:o__Sphingobacteriales:f__:g__
MID1	denovo1201	2	k__Bacteria:p__Proteobacteria:c__Alphaproteobacteria:o__Rickettsiales:f__Rickettsiaceae:g__Rickettsia
MID1	denovo1202	14	k__Bacteria:p__Proteobacteria:c__Alphaproteobacteria:o__Rhizobiales:f__Methylobacteriaceae:g__Methylobacterium
MID1	denovo1203	2	k__Bacteria:p__Proteobacteria:c__Alphaproteobacteria:o__Rhizobiales:f__Methylobacteriaceae:g__Methylobacterium
MID1	denovo1209	1	k__Bacteria:p__Firmicutes:c__Bacilli:o__Bacillales:f__Paenibacillaceae:g__
MID1	denovo1211	3	k__Bacteria:p__Proteobacteria:c__Gammaproteobacteria:o__Xanthomonadales:f__Xanthomonadaceae:g__Dokdonella
MID1	denovo1217	2	k__Bacteria:p__Cyanobacteria:c__Chloroplast:o__Chlorophyta:f__:g__
MID1	denovo1219	1	k__Bacteria:p__Firmicutes:c__Clostridia:o__Clostridiales:f__Lachnospiraceae:g__
MID1	denovo1221	1	k__Bacteria:p__Firmicutes:c__Clostridia:o__Clostridiales:f__Ruminococcaceae:g__Ruminococcus
MID1	denovo1222	2	k__Bacteria:p__Proteobacteria:c__Betaproteobacteria:o__Burkholderiales:f__Alcaligenaceae:g__
MID1	denovo1224	1	k__Bacteria:p__Actinobacteria:c__Actinobacteria:o__Actinomycetales:f__Micromonosporaceae:g__Virgisporangium
MID1	denovo1226	4	k__Bacteria:p__Actinobacteria:c__Actinobacteria:o__Actinomycetales:f__Nocardioidaceae:g__Friedmanniella
MID1	denovo1227	3	k__Bacteria:p__Proteobacteria:c__Alphaproteobacteria:o__Rhizobiales:f__Rhizobiaceae
MID1	denovo1229	1	k__Bacteria:p__Bacteroidetes:c__Cytophagia:o__Cytophagales:f__Flammeovirgaceae
MID1	denovo1230	1	k__Bacteria:p__Proteobacteria:c__Betaproteobacteria:o__Burkholderiales:f__Comamonadaceae:g__Comamonas
MID1	denovo1231	5	k__Bacteria:p__Proteobacteria:c__Alphaproteobacteria:o__Rhodobacterales:f__Rhodobacteraceae:g__Rubellimicrobium

MID1	denovo1232	1	k__Bacteria:p__Bacteroidetes:c__Flavobacteriia:o__Flavobacteriales:f__Flavobacteriaceae:g__Flavobacterium
MID1	denovo1235	1	k__Bacteria:p__Firmicutes:c__Clostridia:o__Clostridiales:f__Clostridiaceae:g__Clostridium
MID1	denovo1236	1	k__Bacteria:p__Bacteroidetes:c__Cytophagia:o__Cytophagales:f__Cytophagaceae:g__Rhodocytophaga
MID1	denovo1242	13	k__Bacteria:p__Proteobacteria:c__Gammaproteobacteria:o__Xanthomonadales:f__Xanthomonadaceae:g__Lysobacter
MID1	denovo1243	11	k__Bacteria:p__Proteobacteria:c__Alphaproteobacteria:o__Rhizobiales:f__Phyllobacteriaceae
MID1	denovo1246	1	k__Bacteria:p__Acidobacteria:c__Acidobacteria-6:o__iii1-15:f__mb2424:g__
MID1	denovo125	1	k__Bacteria:p__Acidobacteria:c__[Chloracidobacteria]:o__RB41:f__:g__
MID1	denovo1251	1	k__Bacteria:p__Actinobacteria:c__Thermoleophilia:o__Solirubrobacterales:f__:g__
MID1	denovo1253	11	k__Bacteria:p__Proteobacteria:c__Alphaproteobacteria:o__Rhizobiales:f__Rhizobiaceae:g__Agrobacterium
MID1	denovo1256	1	k__Bacteria:p__Bacteroidetes:c__Cytophagia:o__Cytophagales:f__Cytophagaceae:g__
MID1	denovo1257	13	k__Bacteria:p__Proteobacteria:c__Alphaproteobacteria:o__Sphingomonadales:f__Sphingomonadaceae
MID1	denovo1259	4	k__Bacteria:p__Proteobacteria:c__Alphaproteobacteria:o__Sphingomonadales:f__Sphingomonadaceae:g__Sphingobium
MID1	denovo126	1	k__Bacteria:p__Proteobacteria:c__Alphaproteobacteria:o__Rhizobiales:f__Hyphomicrobiaceae:g__Rhodoplanes
MID1	denovo1262	1	k__Bacteria:p__Proteobacteria:c__Deltaproteobacteria:o__Myxococcales:f__:g__
MID1	denovo1263	3	k__Bacteria:p__Firmicutes:c__Clostridia:o__Clostridiales:f__[Tissierellaceae]:g__
MID1	denovo1264	2	k__Bacteria:p__Proteobacteria:c__Betaproteobacteria:o__Burkholderiales:f__Comamonadaceae
MID1	denovo1266	1	k__Bacteria:p__Cyanobacteria:c__Oscillatoriophycideae:o__Chroococcales:f__Xenococcaceae:g__
MID1	denovo1268	3	k__Bacteria:p__Proteobacteria:c__Alphaproteobacteria:o__Sphingomonadales:f__Sphingomonadaceae:g__
MID1	denovo127	1	k__Bacteria:p__Proteobacteria:c__Gammaproteobacteria:o__Pseudomonadales:f__Pseudomonadaceae:g__
MID1	denovo1274	1	k__Bacteria:p__Bacteroidetes:c__Flavobacteriia:o__Flavobacteriales:f__[Weeksellaceae]:g__
MID1	denovo1277	1	k__Bacteria:p__Firmicutes:c__Bacilli:o__Bacillales:f__Paenibacillaceae:g__Paenibacillus
MID1	denovo1278	1	k__Bacteria:p__Proteobacteria:c__Alphaproteobacteria:o__Rhizobiales:f__Methylobacteriaceae:g__Methylobacterium
MID1	denovo1279	1	k__Bacteria:p__Firmicutes:c__Bacilli:o__Lactobacillales
MID1	denovo128	2	k__Bacteria:p__Armatimonadetes:c__Armatimonadia:o__Armatimonadales:f__Armatimonadaceae:g__
MID1	denovo1281	2	k__Bacteria:p__Actinobacteria:c__Acidimicrobiia:o__Acidimicrobiales:f__C111:g__
MID1	denovo1283	2	k__Bacteria:p__Chlorobi:c__OPB56:o__:f__:g__
MID1	denovo1285	2	k__Bacteria:p__Firmicutes:c__Clostridia:o__Clostridiales:f__Lachnospiraceae:g__Blautia
MID1	denovo129	1	k__Bacteria:p__Bacteroidetes:c__Flavobacteriia:o__Flavobacteriales:f__Flavobacteriaceae:g__Gelidibacter
MID1	denovo1290	17	k__Bacteria:p__Actinobacteria:c__Actinobacteria:o__Actinomycetales:f__Dermabacteraceae:g__Brachybacterium
MID1	denovo1292	1	k__Bacteria:p__Proteobacteria:c__Alphaproteobacteria:o__Rhodospirillales:f__Acetobacteraceae:g__
MID1	denovo1293	1	k__Bacteria:p__Firmicutes:c__Clostridia:o__Clostridiales:f__:g__
MID1	denovo1294	15	k__Bacteria:p__Proteobacteria:c__Betaproteobacteria:o__Burkholderiales:f__Alcaligenaceae:g__
MID1	denovo1297	1	k__Bacteria:p__Cyanobacteria:c__Chloroplast:o__Chlorophyta:f__Trebouxiophyceae
MID1	denovo1299	1	Unassigned
MID1	denovo1300	38	k__Bacteria:p__Actinobacteria:c__Actinobacteria:o__Actinomycetales:f__Nocardioidaceae:g__Friedmanniella
MID1	denovo1303	1	k__Bacteria:p__Actinobacteria:c__Actinobacteria:o__Actinomycetales:f__Propionibacteriaceae:g__
MID1	denovo1304	1	k__Bacteria:p__Chloroflexi:c__Chloroflexi:o__Chloroflexales:f__Chloroflexaceae
MID1	denovo1305	1	k__Bacteria:p__Gemmatimonadetes:c__Gemmatimonadetes:o__Gemmatimonadales:f__:g__
MID1	denovo1307	8	k__Bacteria:p__Cyanobacteria:c__Oscillatoriophycideae:o__Oscillatoriales:f__Phormidiaceae:g__Phormidium
MID1	denovo1308	9	k__Bacteria:p__Firmicutes:c__Bacilli:o__Lactobacillales:f__Streptococcaceae:g__Streptococcus
MID1	denovo1309	2	k__Bacteria:p__Proteobacteria:c__Alphaproteobacteria:o__Rhodospirillales:f__Rhodospirillaceae:g__
MID1	denovo1310	3	k__Bacteria:p__Proteobacteria:c__Betaproteobacteria:o__Burkholderiales:f__Comamonadaceae:g__
MID1	denovo1311	7	k__Bacteria:p__Firmicutes:c__Clostridia:o__Clostridiales:f__Clostridiaceae:g__
MID1	denovo1313	1	k__Bacteria:p__Acidobacteria:c__Solibacteres:o__Solibacterales:f__:g__
MID1	denovo1319	3	k__Bacteria:p__Proteobacteria:c__Alphaproteobacteria:o__Sphingomonadales:f__Sphingomonadaceae:g__Kaistobacter
MID1	denovo132	6	k__Bacteria:p__Proteobacteria:c__Alphaproteobacteria:o__Sphingomonadales:f__Erythrobacteraceae:g__
MID1	denovo1320	1	k__Bacteria:p__Acidobacteria:c__Acidobacteriia:o__Acidobacteriales:f__Acidobacteriaceae:g__
MID1	denovo1323	1	k__Bacteria:p__Proteobacteria:c__Alphaproteobacteria:o__Rhizobiales:f__Methylocystaceae:g__
MID1	denovo1324	1	k__Bacteria:p__Proteobacteria:c__Alphaproteobacteria:o__Rhodospirillales:f__Acetobacteraceae:g__
MID1	denovo1325	1	k__Bacteria:p__Proteobacteria:c__Alphaproteobacteria:o__Rhizobiales:f__Hyphomicrobiaceae:g__Devosia
MID1	denovo1326	2	k__Bacteria:p__Proteobacteria:c__Alphaproteobacteria:o__Rhizobiales:f__Aurantimonadaceae:g__
MID1	denovo1327	1	k__Bacteria:p__Firmicutes:c__Clostridia:o__Clostridiales:f__Peptococcaceae:g__
MID1	denovo1331	1	k__Bacteria:p__Proteobacteria:c__Alphaproteobacteria:o__Rhizobiales:f__Beijerinckiaceae:g__
MID1	denovo1332	1	k__Bacteria:p__Firmicutes:c__Clostridia:o__Clostridiales:f__Ruminococcaceae:g__Oscillospira
MID1	denovo1334	60	k__Bacteria:p__Firmicutes:c__Bacilli:o__Lactobacillales:f__Streptococcaceae:g__Streptococcus
MID1	denovo1336	1	k__Bacteria:p__Actinobacteria:c__Acidimicrobiia:o__Acidimicrobiales:f__C111:g__
MID1	denovo1337	1	k__Bacteria:p__Proteobacteria:c__Alphaproteobacteria:o__Sphingomonadales:f__Sphingomonadaceae:g__Sphingomonas
MID1	denovo134	9	k__Bacteria:p__Firmicutes:c__Bacilli:o__Lactobacillales:f__:g__
MID1	denovo1341	1	k__Bacteria:p__Firmicutes:c__Clostridia:o__Clostridiales:f__[Tissierellaceae]:g__Anaerococcus
MID1	denovo1342	1	k__Bacteria:p__Actinobacteria:c__Actinobacteria:o__Actinomycetales:f__Intrasporangiaceae
MID1	denovo1343	3	k__Bacteria:p__Proteobacteria:c__Alphaproteobacteria:o__Sphingomonadales:f__Sphingomonadaceae:g__Sphingomonas
MID1	denovo1344	1	k__Bacteria:p__Bacteroidetes:c__Cytophagia:o__Cytophagales:f__Cytophagaceae:g__Spirosoma
MID1	denovo1345	4	k__Bacteria:p__Firmicutes:c__Bacilli:o__Bacillales:f__Alicyclobacillaceae:g__Alicyclobacillus
MID1	denovo1346	40	k__Bacteria:p__Firmicutes:c__Bacilli:o__Lactobacillales:f__Streptococcaceae:g__Lactococcus
MID1	denovo1349	1	k__Bacteria:p__Proteobacteria:c__Deltaproteobacteria:o__Myxococcales:f__Nannocystaceae:g__Nannocystis
MID1	denovo1351	4	k__Bacteria:p__Proteobacteria:c__Alphaproteobacteria:o__Rhizobiales:f__Methylocystaceae:g__
MID1	denovo1352	32	k__Bacteria:p__Proteobacteria:c__Alphaproteobacteria:o__Rhodobacterales:f__Rhodobacteraceae

MID1	denovo1353	2	k__Bacteria:p__Firmicutes:c__Clostridia:o__Clostridiales:f__Caldicoprobacteraceae:g__Caldicoprobacter
MID1	denovo1354	13	k__Bacteria:p__Actinobacteria:c__Actinobacteria:o__Actinomycetales:f__Intrasporangiaceae:g__
MID1	denovo1355	1	k__Bacteria:p__Firmicutes:c__Clostridia:o__Clostridiales:f__Eubacteriaceae:g__Alkalibacter
MID1	denovo1356	8	k__Bacteria:p__Bacteroidetes:c__Flavobacteriia:o__Flavobacteriales:f__Flavobacteriaceae:g__Aequorivita
MID1	denovo1358	1	k__Bacteria:p__Bacteroidetes:c__Flavobacteriia:o__Flavobacteriales:f__Flavobacteriaceae:g__Aequorivita
MID1	denovo1359	1	k__Bacteria:p__Proteobacteria:c__Gammaproteobacteria:o__Xanthomonadales:f__Xanthomonadaceae:g__
MID1	denovo1360	1	k__Bacteria:p__Proteobacteria:c__Gammaproteobacteria:o__Pseudomonadales:f__Moraxellaceae:g__
MID1	denovo1361	12	k__Bacteria:p__Actinobacteria:c__Actinobacteria:o__Actinomycetales:f__Microbacteriaceae:g__Leucobacter
MID1	denovo137	2	k__Bacteria:p__Cyanobacteria:c__Chloroplast:o__Chlorophyta:f__:g__
MID1	denovo138	2	k__Bacteria:p__Proteobacteria:c__Alphaproteobacteria:o__Rhodospirillales:f__Rhodospirillaceae:g__Skermanella
MID1	denovo14	1	k__Bacteria:p__Firmicutes:c__Clostridia:o__Clostridiales:f__Clostridiaceae:g__Clostridium
MID1	denovo141	1	k__Bacteria:p__Proteobacteria:c__Betaproteobacteria:o__Neisseriales:f__Neisseriaceae:g__
MID1	denovo142	3	k__Bacteria:p__Proteobacteria:c__Alphaproteobacteria:o__Rhodobacterales:f__Rhodobacteraceae:g__
MID1	denovo143	1	k__Bacteria:p__Actinobacteria:c__Actinobacteria:o__Actinomycetales:f__Nocardioidaceae:g__
MID1	denovo144	1	k__Bacteria:p__Actinobacteria:c__Actinobacteria:o__Actinomycetales:f__Propionibacteriaceae:g__Luteococcus
MID1	denovo147	3	k__Bacteria:p__Firmicutes:c__Clostridia:o__Clostridiales:f__Lachnospiraceae:g__Dorea
MID1	denovo148	1	k__Bacteria:p__Cyanobacteria:c__Synechococcophycideae:o__Pseudanabaenales:f__Pseudanabaenaceae:g__Leptolyngbya
MID1	denovo149	2	k__Bacteria:p__Actinobacteria:c__Actinobacteria:o__Actinomycetales:f__Cellulomonadaceae:g__Cellulomonas
MID1	denovo15	3	k__Bacteria:p__[Thermi]:c__Deinococci:o__Deinococcales:f__Trueperaceae:g__Truepera
MID1	denovo150	2	k__Bacteria:p__Proteobacteria:c__Gammaproteobacteria:o__[Marinicellales]:f__[Marinicellaceae]:g__
MID1	denovo151	2	k__Bacteria:p__FBP:c__:o__:f__:g__
MID1	denovo152	1	k__Bacteria:p__Actinobacteria:c__Thermoleophilia:o__Solirubrobacterales:f__:g__
MID1	denovo153	2	k__Bacteria:p__Chlorobi:c__SJA-28:o__:f__:g__
MID1	denovo154	1	k__Bacteria:p__Gemmatimonadetes:c__Gemmatimonadetes:o__Gemmatimonadales:f__:g__
MID1	denovo155	2	k__Bacteria:p__Proteobacteria:c__Alphaproteobacteria:o__Rhodobacterales:f__Rhodobacteraceae:g__Rubellimicrobium
MID1	denovo156	1	k__Bacteria:p__Acidobacteria:c__[Chloracidobacteria]:o__RB41:f__:g__
MID1	denovo158	1	k__Bacteria:p__Proteobacteria:c__Alphaproteobacteria:o__Sphingomonadales:f__Sphingomonadaceae:g__
MID1	denovo159	10	k__Bacteria:p__Actinobacteria:c__Actinobacteria:o__Actinomycetales:f__Propionibacteriaceae:g__Microlunatus
MID1	denovo163	1	k__Bacteria:p__Proteobacteria:c__Alphaproteobacteria:o__Rhodobacterales:f__Rhodobacteraceae:g__Paracoccus
MID1	denovo165	1	k__Bacteria:p__Proteobacteria:c__Deltaproteobacteria:o__Spirobacillales:f__:g__
MID1	denovo167	1	k__Bacteria:p__Bacteroidetes:c__Bacteroidia:o__Bacteroidales:f__S24-7:g__
MID1	denovo172	1	k__Bacteria:p__Planctomycetes:c__Planctomycetia:o__Gemmatales:f__Gemmataceae:g__
MID1	denovo173	4	k__Bacteria:p__Proteobacteria:c__Alphaproteobacteria:o__Rhodospirillales:f__Rhodospirillaceae:g__
MID1	denovo177	31	k__Bacteria:p__Proteobacteria:c__Betaproteobacteria:o__Nitrosomonadales:f__Nitrosomonadaceae
MID1	denovo178	2	k__Bacteria:p__Bacteroidetes:c__[Rhodothermi]:o__[Rhodothermales]:f__Rhodothermaceae:g__Rubricoccus
MID1	denovo183	168	k__Bacteria:p__Cyanobacteria:c__Oscillatoriophycideae:o__Chroococcales:f__Xenococcaceae
MID1	denovo184	1	k__Bacteria:p__Firmicutes:c__Clostridia:o__Clostridiales:f__[Tissierellaceae]:g__Peptoniphilus
MID1	denovo185	2	k__Bacteria:p__Proteobacteria:c__Gammaproteobacteria:o__Xanthomonadales:f__Xanthomonadaceae:g__
MID1	denovo187	4	k__Bacteria:p__Firmicutes:c__Clostridia:o__Clostridiales:f__Clostridiaceae:g__
MID1	denovo19	16	k__Bacteria:p__Bacteroidetes:c__Bacteroidia:o__Bacteroidales:f__Porphyromonadaceae:g__
MID1	denovo193	3	k__Bacteria:p__Proteobacteria:c__Alphaproteobacteria:o__Caulobacterales:f__Caulobacteraceae:g__Phenylobacterium
MID1	denovo196	1	k__Bacteria:p__Proteobacteria:c__Alphaproteobacteria:o__Rhodobacterales:f__Rhodobacteraceae:g__
MID1	denovo197	2	k__Bacteria:p__Bacteroidetes:c__Flavobacteriia:o__Flavobacteriales:f__[Weeksellaceae]:g__Wautersiella
MID1	denovo198	1	k__Bacteria:p__Actinobacteria:c__Actinobacteria:o__Actinomycetales:f__Microbacteriaceae:g__Leucobacter
MID1	denovo2	1	k__Bacteria:p__Proteobacteria:c__Betaproteobacteria:o__Burkholderiales:f__Oxalobacteraceae:g__
MID1	denovo20	5	k__Bacteria:p__Proteobacteria:c__Alphaproteobacteria:o__Sphingomonadales:f__Sphingomonadaceae:g__
MID1	denovo200	42	k__Bacteria:p__Proteobacteria:c__Alphaproteobacteria:o__Rhodobacterales:f__Rhodobacteraceae:g__Rhodobacter
MID1	denovo202	1	k__Bacteria:p__Proteobacteria:c__Alphaproteobacteria:o__Rhizobiales:f__Hyphomicrobiaceae:g__Devosia
MID1	denovo203	2	k__Bacteria:p__Proteobacteria:c__Gammaproteobacteria:o__Xanthomonadales:f__Xanthomonadaceae:g__
MID1	denovo205	1	k__Bacteria:p__Bacteroidetes:c__Flavobacteriia:o__Flavobacteriales:f__[Weeksellaceae]:g__
MID1	denovo207	4	k__Bacteria:p__Actinobacteria:c__Acidimicrobiia:o__Acidimicrobiales:f__C111:g__
MID1	denovo209	1	k__Bacteria:p__Cyanobacteria:c__Oscillatoriophycideae:o__Chroococcales:f__Xenococcaceae:g__Chroococcidiopsis
MID1	denovo210	5	k__Bacteria:p__Proteobacteria:c__Gammaproteobacteria:o__Xanthomonadales:f__Xanthomonadaceae:g__
MID1	denovo211	1	k__Bacteria:p__Proteobacteria:c__Alphaproteobacteria:o__Rhizobiales:f__Phyllobacteriaceae:g__
MID1	denovo212	1	k__Bacteria:p__Cyanobacteria:c__Chloroplast:o__Streptophyta:f__:g__
MID1	denovo213	5	k__Bacteria:p__Proteobacteria:c__Gammaproteobacteria:o__Xanthomonadales:f__Xanthomonadaceae:g__Luteimonas
MID1	denovo215	10	k__Bacteria:p__Planctomycetes:c__Planctomycetia:o__Pirellulales:f__Pirellulaceae:g__
MID1	denovo216	2	k__Bacteria:p__Actinobacteria:c__Actinobacteria:o__Actinomycetales:f__Nocardioidaceae:g__Propionicimonas
MID1	denovo217	3	k__Bacteria:p__Actinobacteria:c__Actinobacteria:o__Actinomycetales:f__:g__
MID1	denovo218	5	k__Bacteria:p__Cyanobacteria:c__Oscillatoriophycideae:o__Oscillatoriales:f__Phormidiaceae:g__Phormidium
MID1	denovo219	1	k__Bacteria:p__Proteobacteria:c__Alphaproteobacteria:o__Rhodobacterales:f__Rhodobacteraceae:g__Rubellimicrobium
MID1	denovo22	1	k__Bacteria:p__Proteobacteria:c__Alphaproteobacteria:o__Rhodobacterales:f__Rhodobacteraceae
MID1	denovo222	1	k__Bacteria:p__Proteobacteria:c__Deltaproteobacteria:o__MIZ46:f__:g__
MID1	denovo223	2	k__Bacteria:p__FBP:c__:o__:f__:g__
MID1	denovo226	1	k__Bacteria:p__Proteobacteria:c__Gammaproteobacteria:o__Xanthomonadales:f__Xanthomonadaceae:g__
MID1	denovo228	5	k__Bacteria:p__Proteobacteria:c__Alphaproteobacteria:o__Sphingomonadales:f__Sphingomonadaceae:g__Sphingomonas
MID1	denovo229	2	k__Bacteria:p__Proteobacteria:c__Alphaproteobacteria:o__Rhodospirillales:f__Acetobacteraceae:g__

MID1	denovo23	1	k__Bacteria:p__Cyanobacteria:c__Oscillatoriophycideae:o__Chroococcales:f__Xenococcaceae:g__
MID1	denovo233	1	k__Bacteria:p__Bacteroidetes:c__[Saprospirae]:o__[Saprospirales]:f__Chitinophagaceae:g__
MID1	denovo235	5	k__Bacteria:p__Proteobacteria:c__Alphaproteobacteria:o__Sphingomonadales:f__Erythrobacteraceae:g__
MID1	denovo236	4	k__Bacteria:p__Actinobacteria:c__Actinobacteria:o__Actinomycetales:f__Geodermatophilaceae:g__
MID1	denovo237	115	k__Bacteria:p__Firmicutes:c__Clostridia:o__Clostridiales:f__[Tissierellaceae]:g__
MID1	denovo239	3	k__Bacteria:p__Gemmatimonadetes:c__Gemmatimonadetes:o__:f__:g__
MID1	denovo240	1	k__Bacteria:p__Acidobacteria:c__Solibacteres:o__Solibacterales:f__:g__
MID1	denovo241	1	k__Bacteria:p__Proteobacteria:c__Alphaproteobacteria:o__Rhodospirillales:f__:g__
MID1	denovo242	1	k__Bacteria:p__Actinobacteria:c__Acidimicrobiia:o__Acidimicrobiales:f__AKIW874:g__
MID1	denovo250	3	k__Bacteria:p__Proteobacteria:c__Alphaproteobacteria:o__Rhizobiales:f__Methylocystaceae
MID1	denovo251	2	k__Bacteria:p__Cyanobacteria:c__Synechococcophycideae:o__Pseudanabaenales:f__Pseudanabaenaceae:g__Leptolyngbya
MID1	denovo256	1	k__Bacteria:p__Actinobacteria:c__Actinobacteria:o__Actinomycetales:f__Propionibacteriaceae:g__
MID1	denovo258	1	k__Bacteria:p__Firmicutes:c__Clostridia:o__Clostridiales:f__Lachnospiraceae:g__Oribacterium
MID1	denovo259	1	k__Bacteria:p__Gemmatimonadetes:c__Gemmatimonadetes:o__Gemmatimonadales:f__:g__
MID1	denovo26	1	k__Bacteria:p__Proteobacteria:c__Alphaproteobacteria:o__Rhizobiales:f__Brucellaceae
MID1	denovo262	2	k__Bacteria:p__Proteobacteria:c__Alphaproteobacteria:o__Caulobacterales:f__Caulobacteraceae:g__Phenylobacterium
MID1	denovo263	1	k__Bacteria:p__Proteobacteria:c__Betaproteobacteria:o__Rhodocyclales:f__Rhodocyclaceae:g__Dechloromonas
MID1	denovo265	3	k__Bacteria:p__Cyanobacteria:c__Oscillatoriophycideae:o__Oscillatoriales:f__Phormidiaceae
MID1	denovo266	2	k__Bacteria:p__Proteobacteria:c__Alphaproteobacteria:o__Sphingomonadales:f__Erythrobacteraceae
MID1	denovo268	1	k__Bacteria:p__Actinobacteria:c__Actinobacteria:o__Actinomycetales:f__Microbacteriaceae:g__Agrococcus
MID1	denovo272	8	k__Bacteria:p__Proteobacteria:c__Alphaproteobacteria:o__Rhodobacterales:f__Rhodobacteraceae:g__Rubellimicrobium
MID1	denovo276	1	k__Bacteria:p__Actinobacteria:c__Actinobacteria:o__Actinomycetales:f__:g__
MID1	denovo279	1	k__Bacteria:p__Proteobacteria:c__Gammaproteobacteria:o__Xanthomonadales:f__Xanthomonadaceae:g__
MID1	denovo28	1	k__Bacteria:p__Proteobacteria:c__Alphaproteobacteria:o__Caulobacterales:f__Caulobacteraceae:g__
MID1	denovo280	2	k__Bacteria:p__Firmicutes:c__Clostridia:o__Clostridiales:f__Clostridiaceae:g__Alkaliphilus
MID1	denovo283	1	k__Bacteria:p__Firmicutes:c__Clostridia:o__Clostridiales:f__Peptostreptococcaceae:g__Tepidibacter
MID1	denovo286	1	k__Bacteria:p__Actinobacteria:c__Actinobacteria:o__Actinomycetales:f__Frankiaceae:g__
MID1	denovo287	5	k__Bacteria:p__Bacteroidetes:c__Flavobacteriia:o__Flavobacteriales:f__Flavobacteriaceae:g__Arenibacter
MID1	denovo288	4	k__Bacteria:p__Proteobacteria:c__Alphaproteobacteria:o__Caulobacterales:f__Caulobacteraceae:g__
MID1	denovo290	2	k__Bacteria:p__Proteobacteria:c__Alphaproteobacteria:o__Sphingomonadales:f__Sphingomonadaceae:g__
MID1	denovo291	2	k__Bacteria:p__Proteobacteria:c__Betaproteobacteria:o__Burkholderiales:f__Comamonadaceae:g__Delftia
MID1	denovo293	1	k__Bacteria:p__Acidobacteria:c__Solibacteres:o__Solibacterales:f__:g__
MID1	denovo294	1	k__Bacteria:p__Firmicutes:c__Clostridia:o__Clostridiales:f__[Tissierellaceae]:g__Peptoniphilus
MID1	denovo296	3	k__Bacteria:p__Firmicutes:c__Clostridia:o__Clostridiales:f__[Tissierellaceae]:g__WAL_1855D
MID1	denovo3	1	k__Bacteria:p__Armatimonadetes:c__Armatimonadia:o__Armatimonadales:f__Armatimonadaceae:g__
MID1	denovo30	4	k__Bacteria:p__Actinobacteria:c__Actinobacteria:o__Actinomycetales:f__Bogoriellaceae:g__Georgenia
MID1	denovo301	2	k__Bacteria:p__Bacteroidetes:c__Bacteroidia:o__Bacteroidales:f__Porphyromonadaceae:g__
MID1	denovo302	1	k__Bacteria:p__Proteobacteria:c__Alphaproteobacteria:o__Rhizobiales:f__Aurantimonadaceae:g__
MID1	denovo308	1	k__Bacteria:p__Bacteroidetes:c__Cytophagia:o__Cytophagales:f__Cyclobacteriaceae:g__
MID1	denovo309	1	k__Bacteria:p__Actinobacteria:c__Acidimicrobiia:o__Acidimicrobiales:f__C111:g__
MID1	denovo310	1	k__Bacteria:p__Cyanobacteria:c__Synechococcophycideae:o__Pseudanabaenales:f__Pseudanabaenaceae:g__Leptolyngbya
MID1	denovo311	1	k__Bacteria:p__Chloroflexi:c__Gitt-GS-136:o__:f__:g__
MID1	denovo312	1	k__Bacteria:p__Proteobacteria:c__Alphaproteobacteria:o__Rhodobacterales:f__Rhodobacteraceae:g__Rubellimicrobium
MID1	denovo314	1	k__Bacteria:p__Proteobacteria:c__Alphaproteobacteria:o__Rhizobiales:f__Beijerinckiaceae:g__
MID1	denovo315	6	k__Bacteria:p__Proteobacteria:c__Alphaproteobacteria:o__Rhizobiales:f__Rhizobiaceae:g__Rhizobium
MID1	denovo317	2	k__Bacteria:p__Actinobacteria:c__Acidimicrobiia:o__Acidimicrobiales:f__:g__
MID1	denovo318	2	k__Bacteria:p__Cyanobacteria:c__Oscillatoriophycideae:o__Chroococcales:f__Xenococcaceae:g__
MID1	denovo319	3	k__Bacteria:p__Bacteroidetes:c__Flavobacteriia:o__Flavobacteriales:f__[Weeksellaceae]:g__Chryseobacterium
MID1	denovo32	1	k__Bacteria:p__Gemmatimonadetes:c__Gemmatimonadetes:o__Gemmatimonadales:f__:g__
MID1	denovo320	3	k__Bacteria:p__Gemmatimonadetes:c__Gemmatimonadetes:o__Gemmatimonadales:f__:g__
MID1	denovo323	1	k__Bacteria:p__Proteobacteria:c__Gammaproteobacteria:o__Pseudomonadales:f__Pseudomonadaceae:g__Pseudomonas
MID1	denovo324	5	k__Bacteria:p__Proteobacteria:c__Alphaproteobacteria:o__Rhizobiales:f__Hyphomicrobiaceae:g__Devosia
MID1	denovo326	2	k__Bacteria:p__Bacteroidetes:c__Cytophagia:o__Cytophagales:f__Flammeovirgaceae:g__
MID1	denovo327	1	k__Bacteria:p__Actinobacteria:c__Actinobacteria:o__Actinomycetales:f__Pseudonocardiaceae:g__Pseudonocardia
MID1	denovo329	1	k__Bacteria:p__Acidobacteria:c__Acidobacteriia:o__Acidobacteriales:f__Acidobacteriaceae:g__
MID1	denovo33	1	k__Bacteria:p__Proteobacteria:c__Gammaproteobacteria:o__Xanthomonadales:f__Xanthomonadaceae:g__Lysobacter
MID1	denovo335	4	k__Bacteria:p__Actinobacteria:c__Actinobacteria:o__Actinomycetales:f__Micrococcaceae:g__Arthrobacter
MID1	denovo336	1	Unassigned
MID1	denovo338	16	k__Bacteria:p__Proteobacteria:c__Alphaproteobacteria:o__Rhodobacterales:f__Rhodobacteraceae:g__Paracoccus
MID1	denovo34	26	k__Bacteria:p__Proteobacteria:c__Gammaproteobacteria:o__Xanthomonadales:f__Xanthomonadaceae:g__Dokdonella
MID1	denovo343	3	k__Bacteria:p__Proteobacteria:c__Betaproteobacteria:o__Burkholderiales:f__Comamonadaceae:g__
MID1	denovo344	2	k__Bacteria:p__Proteobacteria:c__Gammaproteobacteria:o__Xanthomonadales:f__Xanthomonadaceae:g__
MID1	denovo347	1	k__Bacteria:p__Cyanobacteria:c__Oscillatoriophycideae:o__Chroococcales:f__Xenococcaceae:g__
MID1	denovo352	1	k__Bacteria:p__Cyanobacteria:c__Nostocophycideae:o__Nostocales:f__Nostocaceae:g__Nostoc
MID1	denovo353	40	k__Bacteria:p__Proteobacteria:c__Alphaproteobacteria:o__Rhodobacterales:f__Rhodobacteraceae:g__Rubellimicrobium
MID1	denovo354	1	k__Bacteria:p__[Thermi]:c__Deinococci:o__Deinococcales:f__Deinococcaceae:g__Deinococcus
MID1	denovo356	1	k__Bacteria:p__Proteobacteria:c__Gammaproteobacteria:o__Alteromonadales:f__:g__

MID1	denovo358	1	k__Bacteria:p__Proteobacteria:c__Alphaproteobacteria:o__Rhizobiales:f__Methylobacteriaceae:g__Methylobacterium
MID1	denovo36	1	k__Bacteria:p__Proteobacteria:c__Alphaproteobacteria:o__Rhizobiales:f__Hyphomicrobiaceae:g__
MID1	denovo361	2	k__Bacteria:p__Firmicutes:c__Bacilli:o__Lactobacillales:f__Lactobacillaceae:g__Lactobacillus
MID1	denovo364	2	k__Bacteria:p__Cyanobacteria:c__Chloroplast:o__Chlorophyta:f__:g__
MID1	denovo366	17	k__Bacteria:p__Proteobacteria:c__Alphaproteobacteria:o__Rhodobacterales:f__Rhodobacteraceae:g__
MID1	denovo368	1	k__Bacteria:p__Actinobacteria:c__Actinobacteria:o__Actinomycetales:f__:g__
MID1	denovo369	2	k__Bacteria:p__Proteobacteria:c__Deltaproteobacteria:o__Myxococcales:f__:g__
MID1	denovo371	1	k__Bacteria:p__Firmicutes:c__Clostridia:o__Clostridiales:f__:g__
MID1	denovo372	2	k__Bacteria:p__Proteobacteria:c__Alphaproteobacteria:o__Sphingomonadales:f__Sphingomonadaceae:g__Zymomonas
MID1	denovo373	1	k__Bacteria:p__Proteobacteria:c__Betaproteobacteria:o__Burkholderiales:f__Alcaligenaceae:g__
MID1	denovo374	6	k__Bacteria:p__Actinobacteria:c__Actinobacteria:o__Actinomycetales:f__Microbacteriaceae:g__Cryocola
MID1	denovo375	1	k__Bacteria:p__Acidobacteria:c__[Chloracidobacteria]:o__RB41:f__Ellin6075:g__
MID1	denovo377	1	k__Bacteria:p__Actinobacteria:c__Actinobacteria:o__Actinomycetales:f__Nocardioidaceae:g__Propionicimonas
MID1	denovo382	1	k__Bacteria:p__FBP:c__:o__:f__:g__
MID1	denovo384	6	k__Bacteria:p__Fusobacteria:c__Fusobacteriia:o__Fusobacteriales:f__Fusobacteriaceae:g__Fusobacterium
MID1	denovo386	1	k__Bacteria:p__Chloroflexi:c__Anaerolineae:o__Anaerolineales:f__Anaerolinaceae:g__Anaerolinea
MID1	denovo389	2	k__Bacteria:p__Proteobacteria:c__Alphaproteobacteria:o__Rhizobiales:f__Methylocystaceae:g__
MID1	denovo390	7	k__Bacteria:p__Actinobacteria:c__Actinobacteria:o__Actinomycetales:f__Microbacteriaceae:g__Frigoribacterium
MID1	denovo392	1	k__Bacteria:p__Proteobacteria:c__Alphaproteobacteria:o__Rhodospirillales:f__Acetobacteraceae:g__
MID1	denovo393	19	k__Bacteria:p__Proteobacteria:c__Gammaproteobacteria:o__Xanthomonadales:f__Xanthomonadaceae:g__Lysobacter
MID1	denovo394	1	k__Bacteria:p__Proteobacteria:c__Betaproteobacteria:o__Gallionellales:f__Gallionellaceae:g__Gallionella
MID1	denovo395	4	k__Bacteria:p__Proteobacteria:c__Alphaproteobacteria:o__Rhodospirillales:f__Acetobacteraceae:g__
MID1	denovo396	3	k__Bacteria:p__Proteobacteria:c__Deltaproteobacteria:o__Myxococcales:f__Polyangiaceae:g__
MID1	denovo397	1	k__Bacteria:p__Actinobacteria:c__Acidimicrobiia:o__Acidimicrobiales:f__EB1017:g__
MID1	denovo398	1	k__Bacteria:p__Bacteroidetes:c__Cytophagia:o__Cytophagales:f__Cytophagaceae:g__Rhodocytophaga
MID1	denovo399	1	k__Bacteria:p__Proteobacteria:c__Deltaproteobacteria:o__Myxococcales:f__:g__
MID1	denovo4	1	k__Bacteria:p__Armatimonadetes:c__Armatimonadia:o__Armatimonadales:f__Armatimonadaceae:g__
MID1	denovo400	1	k__Bacteria:p__Proteobacteria:c__Alphaproteobacteria:o__Rhizobiales:f__Methylobacteriaceae:g__Methylobacterium
MID1	denovo401	1	k__Bacteria:p__Proteobacteria:c__Deltaproteobacteria:o__Myxococcales:f__:g__
MID1	denovo404	9	k__Bacteria:p__Firmicutes:c__Bacilli:o__Lactobacillales:f__Enterococcaceae:g__Enterococcus
MID1	denovo405	1	k__Bacteria:p__Planctomycetes:c__Planctomycetia:o__Gemmatales:f__Gemmataceae:g__Gemmata
MID1	denovo406	1	k__Bacteria:p__Proteobacteria:c__Alphaproteobacteria:o__Sphingomonadales:f__Sphingomonadaceae:g__Sphingobium
MID1	denovo409	3	k__Bacteria:p__Actinobacteria:c__Actinobacteria:o__Actinomycetales:f__Nakamurellaceae:g__
MID1	denovo41	1	k__Bacteria:p__Proteobacteria:c__Alphaproteobacteria:o__Sphingomonadales:f__Sphingomonadaceae:g__
MID1	denovo411	1	k__Bacteria:p__Proteobacteria:c__Betaproteobacteria:o__Neisseriales:f__Neisseriaceae:g__
MID1	denovo413	1	k__Bacteria:p__Bacteroidetes:c__Cytophagia:o__Cytophagales:f__Cytophagaceae:g__
MID1	denovo414	1	k__Bacteria:p__Proteobacteria:c__Alphaproteobacteria:o__Caulobacterales:f__Caulobacteraceae:g__
MID1	denovo415	1	k__Bacteria:p__Cyanobacteria:c__Synechococcophycideae:o__Pseudanabaenales:f__Pseudanabaenaceae:g__Leptolyngbya
MID1	denovo416	1	k__Bacteria:p__Bacteroidetes:c__Bacteroidia:o__Bacteroidales:f__Porphyromonadaceae:g__Candidatus_Azobacteroides
MID1	denovo417	1	k__Bacteria:p__Fibrobacteres:c__Fibrobacteria:o__258ds10:f__:g__
MID1	denovo418	1	Unassigned
MID1	denovo419	1	k__Bacteria:p__Proteobacteria:c__Alphaproteobacteria:o__Rhodospirillales:f__Rhodospirillaceae:g__Skermanella
MID1	denovo423	1	k__Bacteria:p__Firmicutes:c__Clostridia:o__Clostridiales:f__:g__
MID1	denovo424	2	k__Bacteria:p__Bacteroidetes:c__Cytophagia:o__Cytophagales:f__Cytophagaceae:g__Spirosoma
MID1	denovo425	28	k__Bacteria:p__Proteobacteria:c__Gammaproteobacteria:o__Xanthomonadales:f__Xanthomonadaceae:g__
MID1	denovo426	1	k__Bacteria:p__Bacteroidetes:c__Flavobacteriia:o__Flavobacteriales:f__Flavobacteriaceae:g__Gillisia
MID1	denovo428	1	k__Bacteria:p__Proteobacteria:c__Betaproteobacteria:o__Neisseriales:f__Neisseriaceae:g__Kingella
MID1	denovo429	1	k__Bacteria:p__Firmicutes:c__Clostridia:o__Clostridiales:f__Clostridiaceae:g__Clostridium
MID1	denovo430	4	k__Bacteria:p__Actinobacteria:c__Acidimicrobiia:o__Acidimicrobiales:f__Microthrixaceae:g__
MID1	denovo431	3	k__Bacteria:p__Proteobacteria:c__Alphaproteobacteria:o__Rhizobiales:f__Methylobacteriaceae:g__Methylobacterium
MID1	denovo432	4	k__Bacteria:p__Cyanobacteria:c__Synechococcophycideae:o__Pseudanabaenales:f__Pseudanabaenaceae:g__Leptolyngbya
MID1	denovo434	1	k__Bacteria:p__Actinobacteria:c__Actinobacteria:o__Actinomycetales:f__Microbacteriaceae:g__Microbacterium
MID1	denovo435	1	k__Bacteria:p__Actinobacteria:c__Actinobacteria:o__Actinomycetales:f__Nocardiaceae:g__Rhodococcus
MID1	denovo44	1	k__Bacteria:p__Bacteroidetes:c__Cytophagia:o__Cytophagales:f__Cytophagaceae:g__Hymenobacter
MID1	denovo441	1	k__Bacteria:p__Proteobacteria:c__Alphaproteobacteria:o__Rhizobiales:f__Phyllobacteriaceae:g__Aminobacter
MID1	denovo442	1	k__Bacteria:p__Firmicutes:c__Clostridia:o__Clostridiales:f__:g__
MID1	denovo443	2	k__Bacteria:p__Proteobacteria:c__Alphaproteobacteria:o__Rhizobiales:f__Bradyrhizobiaceae:g__
MID1	denovo444	3	k__Bacteria:p__Bacteroidetes:c__Bacteroidia:o__Bacteroidales:f__Porphyromonadaceae:g__Porphyromonas
MID1	denovo447	2	k__Bacteria:p__Proteobacteria:c__Alphaproteobacteria:o__Rhizobiales:f__Hyphomicrobiaceae:g__Rhodoplanes
MID1	denovo45	2	k__Bacteria:p__Actinobacteria:c__Actinobacteria:o__Actinomycetales:f__Kineosporiaceae:g__
MID1	denovo451	13	k__Bacteria:p__[Thermi]:c__Deinococci:o__Deinococcales:f__Trueperaceae:g__B-42
MID1	denovo452	1	k__Bacteria:p__Proteobacteria:c__Alphaproteobacteria:o__Caulobacterales:f__Caulobacteraceae:g__
MID1	denovo454	3	k__Bacteria:p__Proteobacteria:c__Alphaproteobacteria:o__Sphingomonadales:f__Sphingomonadaceae:g__Kaistobacter
MID1	denovo455	1	k__Bacteria:p__Proteobacteria:c__Gammaproteobacteria:o__Pasteurellales:f__Pasteurellaceae:g__Haemophilus
MID1	denovo456	4	k__Bacteria:p__Firmicutes:c__Clostridia:o__Clostridiales:f__[Tissierellaceae]
MID1	denovo457	4	k__Bacteria:p__Actinobacteria:c__Thermoleophilia:o__Solirubrobacterales:f__:g__
MID1	denovo458	1	k__Bacteria:p__Actinobacteria:c__Actinobacteria:o__Actinomycetales:f__:g__

MID1	denovo459	3	k__Bacteria;p__Actinobacteria;c__Actinobacteria;o__Actinomycetales;f__Propionibacteriaceae;g__
MID1	denovo46	1	k__Bacteria;p__Bacteroidetes;c__Cytophagia;o__Cytophagales;f__Cytophagaceae;g__Spirosoma
MID1	denovo460	1	k__Bacteria;p__Actinobacteria;c__Actinobacteria;o__Actinomycetales;f__Propionibacteriaceae;g__
MID1	denovo461	1	k__Bacteria;p__Actinobacteria;c__Thermoleophilia;o__Solirubrobacterales;f__;g__
MID1	denovo462	1	k__Bacteria;p__Chloroflexi;c__Thermomicrobia;o__JG30-KF-CM45;f__;g__
MID1	denovo466	1	k__Bacteria;p__Proteobacteria;c__Alphaproteobacteria;o__Rhodospirillales
MID1	denovo469	1	k__Bacteria;p__Firmicutes;c__Clostridia;o__Clostridiales;f__Ruminococcaceae;g__
MID1	denovo471	2	k__Bacteria;p__Proteobacteria;c__Alphaproteobacteria;o__Rhizobiales;f__Hyphomicrobiaceae;g__Devosia
MID1	denovo473	2	k__Bacteria;p__Firmicutes;c__Clostridia;o__Clostridiales;f__Christensenellaceae;g__
MID1	denovo474	1	Unassigned
MID1	denovo477	1	k__Bacteria;p__Bacteroidetes;c__Bacteroidia;o__Bacteroidales;f__Prevotellaceae;g__Prevotella
MID1	denovo479	1	k__Bacteria;p__Proteobacteria;c__Alphaproteobacteria;o__Caulobacterales;f__Caulobacteraceae
MID1	denovo49	2	k__Bacteria;p__Planctomycetes;c__Planctomycetia;o__Pirellulales;f__Pirellulaceae;g__
MID1	denovo491	4	k__Bacteria;p__Proteobacteria;c__Deltaproteobacteria;o__Myxococcales;f__Haliangiaceae;g__
MID1	denovo492	65	k__Bacteria;p__Cyanobacteria;c__Oscillatoriophycideae;o__Chroococcales;f__Xenococcaceae;g__Chroococcidiopsis
MID1	denovo496	1	k__Bacteria;p__Chloroflexi;c__Anaerolineae;o__SBR1031;f__A4b;g__
MID1	denovo497	1	k__Bacteria;p__Proteobacteria;c__Alphaproteobacteria;o__Rhizobiales;f__Hyphomicrobiaceae;g__Devosia
MID1	denovo498	5	k__Bacteria;p__Proteobacteria;c__Betaproteobacteria;o__Burkholderiales;f__;g__
MID1	denovo5	5	k__Bacteria;p__Proteobacteria;c__Alphaproteobacteria;o__Rhodobacterales;f__Rhodobacteraceae;g__Rubellimicrobium
MID1	denovo50	1	Unassigned
MID1	denovo501	1	k__Bacteria;p__Actinobacteria;c__Actinobacteria;o__Actinomycetales;f__Nocardioidaceae;g__Propionicimonas
MID1	denovo502	2	k__Bacteria;p__Firmicutes;c__Bacilli;o__Lactobacillales;f__Leuconostocaceae;g__Leuconostoc
MID1	denovo504	1	k__Bacteria;p__Proteobacteria;c__Alphaproteobacteria;o__Rhodospirillales;f__Rhodospirillaceae;g__
MID1	denovo508	1	k__Bacteria;p__Bacteroidetes;c__Cytophagia;o__Cytophagales;f__Cytophagaceae;g__Spirosoma
MID1	denovo512	1	k__Bacteria;p__Proteobacteria;c__Gammaproteobacteria;o__Enterobacteriales;f__Enterobacteriaceae;g__Erwinia
MID1	denovo513	5	k__Bacteria;p__Proteobacteria;c__Alphaproteobacteria;o__Rhizobiales;f__Aurantimonadaceae;g__
MID1	denovo515	1	k__Bacteria;p__Actinobacteria;c__Thermoleophilia;o__Gaiellales;f__Gaiellaceae;g__
MID1	denovo516	4	k__Bacteria;p__Gemmatimonadetes;c__Gemm-3;o__;f__;g__
MID1	denovo52	1	k__Bacteria;p__Proteobacteria;c__Alphaproteobacteria;o__Rhizobiales;f__Bradyrhizobiaceae;g__
MID1	denovo520	1	k__Bacteria;p__Acidobacteria;c__[Chloracidobacteria];o__RB41;f__Ellin6075;g__
MID1	denovo522	1	k__Bacteria;p__Proteobacteria;c__Deltaproteobacteria;o__Syntrophobacterales;f__Syntrophobacteraceae;g__
MID1	denovo524	2	k__Bacteria;p__Acidobacteria;c__[Chloracidobacteria];o__RB41;f__Ellin6075;g__
MID1	denovo525	1	k__Bacteria;p__Actinobacteria;c__Actinobacteria;o__Actinomycetales;f__Geodermatophilaceae;g__Modestobacter
MID1	denovo526	1	k__Bacteria;p__Proteobacteria;c__Alphaproteobacteria;o__Rhizobiales;f__Beijerinckiaceae;g__
MID1	denovo527	50	k__Bacteria;p__Proteobacteria;c__Alphaproteobacteria;o__Rhodobacterales;f__Rhodobacteraceae;g__
MID1	denovo529	1	k__Bacteria;p__Proteobacteria;c__Alphaproteobacteria;o__Sphingomonadales;f__Sphingomonadaceae
MID1	denovo53	1	k__Bacteria;p__Proteobacteria;c__Alphaproteobacteria;o__Rhizobiales;f__Hyphomicrobiaceae;g__Devosia
MID1	denovo530	3	k__Bacteria;p__Firmicutes;c__Bacilli;o__Lactobacillales;f__Lactobacillaceae;g__Lactobacillus
MID1	denovo531	1	k__Bacteria;p__Proteobacteria;c__Gammaproteobacteria;o__Xanthomonadales;f__Xanthomonadaceae;g__
MID1	denovo533	1	k__Bacteria;p__Actinobacteria;c__Actinobacteria;o__Actinomycetales;f__Cellulomonadaceae;g__Demequina
MID1	denovo535	1	k__Bacteria;p__Proteobacteria;c__Alphaproteobacteria;o__Caulobacterales;f__Caulobacteraceae;g__
MID1	denovo537	3	k__Bacteria;p__Firmicutes;c__Clostridia;o__Clostridiales;f__Clostridiaceae;g__Proteiniclasticum
MID1	denovo540	123	k__Bacteria;p__Firmicutes;c__Bacilli;o__Bacillales;f__Bacillaceae;g__Bacillus
MID1	denovo542	9	k__Bacteria;p__Firmicutes;c__Clostridia;o__Clostridiales;f__[Tissierellaceae];g__Finegoldia
MID1	denovo543	1	k__Bacteria;p__Firmicutes;c__Bacilli;o__Bacillales;f__Planococcaceae
MID1	denovo546	1	k__Bacteria;p__Armatimonadetes;c__Armatimonadia;o__FW68;f__;g__
MID1	denovo547	1	k__Bacteria;p__Proteobacteria;c__Alphaproteobacteria;o__Rhizobiales;f__Phyllobacteriaceae;g__
MID1	denovo548	1	k__Bacteria;p__Firmicutes;c__Clostridia;o__Clostridiales;f__Lachnospiraceae;g__
MID1	denovo551	16	k__Bacteria;p__Proteobacteria;c__Gammaproteobacteria;o__Alteromonadales;f__Alteromonadaceae;g__Marinobacter
MID1	denovo554	1	k__Bacteria;p__Proteobacteria;c__Betaproteobacteria;o__Burkholderiales;f__Comamonadaceae;g__
MID1	denovo56	3	k__Bacteria;p__Proteobacteria;c__Alphaproteobacteria;o__Rhodobacterales;f__Rhodobacteraceae;g__Rubellimicrobium
MID1	denovo560	1	k__Bacteria;p__Actinobacteria;c__Acidimicrobiia;o__Acidimicrobiales;f__;g__
MID1	denovo562	1	k__Bacteria;p__Actinobacteria;c__Thermoleophilia;o__Solirubrobacterales;f__;g__
MID1	denovo563	1	k__Bacteria;p__Bacteroidetes;c__Flavobacteriia;o__Flavobacteriales;f__Cryomorphaceae;g__Cryomorpha
MID1	denovo57	2	k__Bacteria;p__Proteobacteria;c__Alphaproteobacteria;o__Sphingomonadales;f__Sphingomonadaceae
MID1	denovo571	1	k__Bacteria;p__Proteobacteria;c__Alphaproteobacteria;o__Rhizobiales;f__Hyphomicrobiaceae;g__
MID1	denovo573	1	k__Bacteria;p__Bacteroidetes;c__Cytophagia;o__Cytophagales;f__Cytophagaceae;g__
MID1	denovo574	1	k__Bacteria;p__Proteobacteria;c__Alphaproteobacteria;o__Rhizobiales;f__Methylocystaceae;g__
MID1	denovo575	2	k__Bacteria;p__Actinobacteria;c__Thermoleophilia;o__Gaiellales;f__;g__
MID1	denovo576	3	k__Bacteria;p__Firmicutes;c__Bacilli;o__Lactobacillales;f__Aerococcaceae;g__Facklamia
MID1	denovo579	1	k__Bacteria;p__Bacteroidetes;c__Cytophagia;o__Cytophagales;f__Cytophagaceae;g__Spirosoma
MID1	denovo58	1	k__Bacteria;p__Firmicutes;c__Bacilli;o__Lactobacillales;f__Lactobacillaceae;g__Lactobacillus
MID1	denovo580	1	k__Bacteria;p__Proteobacteria;c__Alphaproteobacteria;o__Sphingomonadales;f__Sphingomonadaceae
MID1	denovo581	1	k__Bacteria;p__Bacteroidetes;c__Flavobacteriia;o__Flavobacteriales;f__[Weeksellaceae];g__Chryseobacterium
MID1	denovo582	2	k__Bacteria;p__Proteobacteria;c__Alphaproteobacteria;o__Sphingomonadales;f__Sphingomonadaceae;g__Kaistobacter
MID1	denovo583	24	k__Bacteria;p__Proteobacteria;c__Betaproteobacteria;o__Burkholderiales;f__Comamonadaceae;g__
MID1	denovo584	3	k__Bacteria;p__Actinobacteria;c__Acidimicrobiia;o__Acidimicrobiales;f__Microthrixaceae;g__

MID1	denovo585	1	k__Bacteria:p__Proteobacteria:c__Alphaproteobacteria:o__Rhizobiales:f__Hyphomicrobiaceae:g__Hyphomicrobium
MID1	denovo586	1	k__Bacteria:p__Planctomycetes:c__Planctomycetia:o__Gemmatales:f__Isosphaeraceae:g__
MID1	denovo587	1	k__Bacteria:p__Cyanobacteria:c__Nostocophycideae
MID1	denovo589	1	k__Bacteria:p__Proteobacteria:c__Alphaproteobacteria:o__Rhodospirillales:f__Acetobacteraceae
MID1	denovo592	20	k__Bacteria:p__Firmicutes:c__Clostridia:o__Clostridiales:f__Clostridiaceae:g__
MID1	denovo593	5	k__Bacteria:p__Actinobacteria:c__Actinobacteria:o__Actinomycetales:f__:g__
MID1	denovo596	2	k__Bacteria:p__Firmicutes:c__Bacilli:o__Bacillales:f__Bacillaceae:g__Geobacillus
MID1	denovo597	1	k__Bacteria:p__Gemmatimonadetes:c__Gemmatimonadetes:o__Gemmatimonadales:f__:g__
MID1	denovo598	6	k__Bacteria:p__Proteobacteria:c__Betaproteobacteria:o__Burkholderiales:f__Comamonadaceae:g__
MID1	denovo6	7	k__Bacteria:p__Proteobacteria:c__Alphaproteobacteria:o__Rhodobacterales:f__Rhodobacteraceae:g__Rhodobacter
MID1	denovo60	2	k__Bacteria:p__Proteobacteria:c__Alphaproteobacteria:o__Sphingomonadales:f__Sphingomonadaceae:g__
MID1	denovo600	28	k__Bacteria:p__Cyanobacteria:c__Oscillatoriophycideae:o__Oscillatoriales:f__Phormidiaceae:g__Phormidium
MID1	denovo605	9	k__Bacteria:p__Firmicutes:c__Bacilli:o__Gemellales:f__Gemellaceae:g__Gemella
MID1	denovo607	3	Unassigned
MID1	denovo609	36	k__Bacteria:p__Actinobacteria:c__Actinobacteria:o__Actinomycetales:f__Intrasporangiaceae:g__
MID1	denovo611	1	k__Bacteria:p__Cyanobacteria:c__Synechococcophycideae:o__Pseudanabaenales:f__Pseudanabaenaceae:g__Leptolyngbya
MID1	denovo614	1	k__Bacteria:p__Acidobacteria:c__[Chloracidobacteria]:o__RB41:f__:g__
MID1	denovo615	1	k__Bacteria:p__Firmicutes:c__Clostridia:o__Clostridiales:f__Clostridiaceae:g__Clostridium
MID1	denovo617	2	k__Bacteria:p__Proteobacteria:c__Betaproteobacteria:o__Burkholderiales:f__Alcaligenaceae:g__
MID1	denovo619	5	k__Bacteria:p__Actinobacteria:c__Acidimicrobiia:o__Acidimicrobiales:f__:g__
MID1	denovo62	7	k__Bacteria:p__Proteobacteria:c__Alphaproteobacteria:o__Sphingomonadales:f__Sphingomonadaceae:g__Sphingomonas
MID1	denovo621	3	k__Bacteria:p__Proteobacteria:c__Alphaproteobacteria:o__Caulobacterales:f__Caulobacteraceae:g__
MID1	denovo624	11	k__Bacteria:p__Proteobacteria:c__Gammaproteobacteria:o__Xanthomonadales:f__Xanthomonadaceae:g__
MID1	denovo625	1	k__Bacteria:p__Bacteroidetes:c__Cytophagia:o__Cytophagales:f__Cytophagaceae:g__Dyadobacter
MID1	denovo626	4	k__Bacteria:p__Cyanobacteria:c__Oscillatoriophycideae:o__Chroococcales:f__:g__
MID1	denovo627	5	k__Bacteria:p__Actinobacteria:c__Actinobacteria:o__Actinomycetales:f__Propionibacteriaceae:g__
MID1	denovo628	1	k__Bacteria:p__Proteobacteria:c__Gammaproteobacteria:o__Xanthomonadales:f__Xanthomonadaceae:g__Arenimonas
MID1	denovo629	2	k__Bacteria:p__Proteobacteria:c__Alphaproteobacteria:o__Rhizobiales:f__Methylocystaceae:g__
MID1	denovo63	7	k__Bacteria:p__Firmicutes:c__Bacilli:o__Lactobacillales:f__Lactobacillaceae:g__Lactobacillus
MID1	denovo637	2	k__Bacteria:p__Cyanobacteria:c__Nostocophycideae:o__Nostocales:f__Nostocaceae
MID1	denovo639	2	k__Bacteria:p__Actinobacteria:c__Actinobacteria:o__Actinomycetales:f__Geodermatophilaceae:g__Modestobacter
MID1	denovo64	2	k__Bacteria:p__Proteobacteria:c__Betaproteobacteria:o__Burkholderiales:f__Comamonadaceae
MID1	denovo641	1	k__Bacteria:p__Acidobacteria:c__Solibacteres:o__Solibacterales:f__:g__
MID1	denovo643	1	k__Bacteria:p__Proteobacteria:c__Alphaproteobacteria:o__Sphingomonadales:f__Sphingomonadaceae:g__Kaistobacter
MID1	denovo647	1	k__Bacteria:p__Proteobacteria:c__Alphaproteobacteria:o__:f__:g__
MID1	denovo649	1	k__Bacteria:p__Cyanobacteria:c__Synechococcophycideae:o__Pseudanabaenales:f__Pseudanabaenaceae:g__Leptolyngbya
MID1	denovo65	3	k__Bacteria:p__Firmicutes:c__Bacilli:o__Bacillales:f__[Exiguobacteraceae]:g__Exiguobacterium
MID1	denovo653	1	k__Bacteria:p__Proteobacteria:c__Alphaproteobacteria:o__Rhizobiales:f__Bradyrhizobiaceae:g__
MID1	denovo654	5	k__Bacteria:p__Proteobacteria:c__Betaproteobacteria:o__Burkholderiales
MID1	denovo655	3	k__Bacteria:p__Firmicutes:c__Clostridia:o__Clostridiales:f__[Tissierellaceae]:g__Anaerococcus
MID1	denovo657	2	k__Bacteria:p__Proteobacteria:c__Alphaproteobacteria:o__Rhodospirillales:f__Acetobacteraceae:g__
MID1	denovo658	1	k__Bacteria:p__Actinobacteria:c__Actinobacteria:o__Actinomycetales:f__Pseudonocardiaceae:g__Pseudonocardia
MID1	denovo661	7	k__Bacteria:p__Actinobacteria:c__Actinobacteria:o__Actinomycetales:f__:g__
MID1	denovo662	1	k__Bacteria:p__Bacteroidetes:c__Bacteroidia:o__Bacteroidales:f__Prevotellaceae:g__Prevotella
MID1	denovo663	2	k__Bacteria:p__Proteobacteria:c__Alphaproteobacteria:o__Rhizobiales:f__Methylobacteriaceae:g__Methylobacterium
MID1	denovo665	4	k__Bacteria:p__Firmicutes:c__Bacilli:o__Lactobacillales:f__Lactobacillaceae:g__Lactobacillus
MID1	denovo667	2	k__Bacteria:p__Actinobacteria:c__Thermoleophilia:o__Solirubrobacterales:f__:g__
MID1	denovo668	1	k__Bacteria:p__Proteobacteria:c__Betaproteobacteria:o__Neisseriales:f__Neisseriaceae:g__Neisseria
MID1	denovo674	3	k__Bacteria:p__Proteobacteria:c__Alphaproteobacteria:o__Rhizobiales:f__Beijerinckiaceae:g__
MID1	denovo675	1	k__Bacteria:p__Bacteroidetes:c__Cytophagia:o__Cytophagales:f__Cytophagaceae:g__Spirosoma
MID1	denovo676	1	k__Bacteria:p__Actinobacteria:c__Actinobacteria:o__Actinomycetales:f__Cellulomonadaceae:g__Actinotalea
MID1	denovo677	3	k__Bacteria:p__Cyanobacteria:c__Nostocophycideae:o__Nostocales:f__Nostocaceae:g__Nostoc
MID1	denovo679	2	k__Bacteria:p__Firmicutes:c__Bacilli:o__Bacillales:f__Bacillaceae:g__Bacillus
MID1	denovo68	1	k__Bacteria:p__Proteobacteria:c__Alphaproteobacteria:o__Rhizobiales:f__Hyphomicrobiaceae:g__Rhodoplanes
MID1	denovo680	1	k__Bacteria:p__Bacteroidetes:c__Bacteroidia:o__Bacteroidales:f__:g__
MID1	denovo681	8	k__Bacteria:p__Bacteroidetes:c__Flavobacteriia:o__Flavobacteriales:f__Flavobacteriaceae:g__Aequorivita
MID1	denovo683	2	k__Bacteria:p__Firmicutes:c__Clostridia:o__Clostridiales:f__Ruminococcaceae:g__
MID1	denovo684	1	k__Bacteria:p__Acidobacteria:c__[Chloracidobacteria]:o__RB41:f__Ellin6075:g__
MID1	denovo685	1	k__Bacteria:p__Proteobacteria:c__TA18:o__CV90:f__:g__
MID1	denovo686	1	k__Bacteria:p__Proteobacteria:c__Deltaproteobacteria:o__Spirobacillales:f__:g__
MID1	denovo69	1	k__Bacteria:p__Proteobacteria:c__Alphaproteobacteria:o__Rhodobacterales:f__Rhodobacteraceae:g__
MID1	denovo690	1	k__Bacteria:p__Proteobacteria:c__Epsilonproteobacteria:o__Campylobacterales:f__Campylobacteraceae:g__Campylobacter
MID1	denovo691	2	k__Bacteria:p__Gemmatimonadetes:c__Gemmatimonadetes:o__Gemmatimonadales:f__Gemmatimonadaceae:g__Gemmatimonas
MID1	denovo696	1	k__Bacteria:p__Actinobacteria:c__Actinobacteria:o__Actinomycetales:f__Propionibacteriaceae:g__Propionibacterium
MID1	denovo697	1	k__Bacteria:p__Proteobacteria:c__Alphaproteobacteria:o__Rhodospirillales:f__Acetobacteraceae:g__
MID1	denovo699	36	k__Bacteria:p__Proteobacteria:c__Alphaproteobacteria:o__Rhodospirillales:f__Acetobacteraceae:g__
MID1	denovo7	1	k__Bacteria:p__Proteobacteria:c__Alphaproteobacteria:o__Sphingomonadales:f__Sphingomonadaceae:g__

MID1	denovo70	4	k__Bacteria:p__Cyanobacteria:c__Nostocophycideae:o__Nostocales:f__Nostocaceae:g__Cylindrospermopsis
MID1	denovo701	1	k__Bacteria:p__Proteobacteria:c__Alphaproteobacteria:o__Rhodospirillales:f__Acetobacteraceae:g__Roseomonas
MID1	denovo702	1	k__Bacteria:p__Firmicutes:c__Clostridia:o__Clostridiales:f__Lachnospiraceae:g__Coprococcus
MID1	denovo703	1	k__Bacteria:p__Firmicutes:c__Bacilli:o__Bacillales:f__Staphylococcaceae:g__Macrococcus
MID1	denovo704	2	k__Bacteria:p__Cyanobacteria:c__Nostocophycideae:o__Nostocales:f__Nostocaceae:g__
MID1	denovo705	1	k__Bacteria:p__Firmicutes:c__Bacilli:o__Bacillales:f__Planococcaceae:g__
MID1	denovo707	1	k__Bacteria:p__Bacteroidetes:c__Bacteroidia:o__Bacteroidales:f__Bacteroidaceae:g__Bacteroides
MID1	denovo708	17	k__Bacteria:p__Firmicutes:c__Clostridia:o__Clostridiales:f__[Tissierellaceae]:g__
MID1	denovo709	6	k__Bacteria:p__Proteobacteria:c__Gammaproteobacteria:o__Pseudomonadales:f__Moraxellaceae:g__Enhydrobacter
MID1	denovo71	4	k__Bacteria:p__Proteobacteria:c__Alphaproteobacteria:o__Rhizobiales:f__Hyphomicrobiaceae:g__Devosia
MID1	denovo716	1	k__Bacteria:p__Bacteroidetes:c__Cytophagia:o__Cytophagales:f__Cytophagaceae:g__Hymenobacter
MID1	denovo717	2	k__Bacteria:p__Proteobacteria:c__Alphaproteobacteria:o__Caulobacterales:f__Caulobacteraceae:g__
MID1	denovo718	2	k__Bacteria:p__Acidobacteria:c__[Chloracidobacteria]:o__RB41:f__Ellin6075:g__
MID1	denovo719	2	k__Bacteria:p__Firmicutes:c__Clostridia:o__Clostridiales:f__:g__
MID1	denovo72	1	k__Bacteria:p__Proteobacteria:c__Alphaproteobacteria:o__Rhodospirillales:f__:g__
MID1	denovo720	39	k__Bacteria:p__Firmicutes:c__Bacilli:o__Lactobacillales:f__Aerococcaceae:g__Facklamia
MID1	denovo721	3	k__Bacteria:p__Bacteroidetes:c__[Saprospirae]:o__[Saprospirales]:f__Chitinophagaceae:g__
MID1	denovo728	5	k__Bacteria:p__Planctomycetes:c__Planctomycetia:o__Gemmatales:f__Isosphaeraceae:g__
MID1	denovo729	1	k__Bacteria:p__Proteobacteria:c__Alphaproteobacteria:o__Rhizobiales:f__Phyllobacteriaceae
MID1	denovo73	1	k__Bacteria:p__FBP:c__:o__:f__:g__
MID1	denovo730	1	k__Bacteria:p__Proteobacteria:c__Alphaproteobacteria:o__BD7-3:f__:g__
MID1	denovo733	5	k__Bacteria:p__Proteobacteria:c__Gammaproteobacteria:o__Xanthomonadales:f__Xanthomonadaceae:g__
MID1	denovo734	1	k__Bacteria:p__Proteobacteria:c__Betaproteobacteria:o__Neisseriales:f__Neisseriaceae:g__Neisseria
MID1	denovo735	2	k__Bacteria:p__Firmicutes:c__Clostridia:o__Clostridiales:f__Gracilibacteraceae:g__
MID1	denovo737	1	k__Bacteria:p__Proteobacteria:c__Alphaproteobacteria:o__RF32:f__:g__
MID1	denovo738	1	k__Bacteria:p__Proteobacteria:c__Alphaproteobacteria:o__Sphingomonadales:f__Erythrobacteraceae:g__
MID1	denovo74	1	k__Bacteria:p__Actinobacteria:c__Actinobacteria:o__Actinomycetales:f__Corynebacteriaceae:g__Corynebacterium
MID1	denovo740	1	k__Bacteria:p__Firmicutes:c__Clostridia:o__Clostridiales:f__Lachnospiraceae:g__
MID1	denovo743	1	k__Bacteria:p__Proteobacteria:c__Gammaproteobacteria:o__Xanthomonadales:f__Xanthomonadaceae:g__
MID1	denovo744	1	k__Bacteria:p__Actinobacteria:c__Acidimicrobiia:o__Acidimicrobiales:f__C111:g__
MID1	denovo745	1	k__Bacteria:p__Proteobacteria:c__Alphaproteobacteria:o__Sphingomonadales:f__Sphingomonadaceae:g__Sphingobium
MID1	denovo748	9	k__Bacteria:p__Proteobacteria:c__Gammaproteobacteria:o__Xanthomonadales:f__Xanthomonadaceae:g__Lysobacter
MID1	denovo75	1	k__Bacteria:p__Proteobacteria:c__Betaproteobacteria:o__Burkholderiales:f__Comamonadaceae:g__Comamonas
MID1	denovo753	2	k__Bacteria:p__Proteobacteria:c__Alphaproteobacteria:o__Sphingomonadales:f__:g__
MID1	denovo755	1	k__Bacteria:p__Proteobacteria:c__Gammaproteobacteria:o__Xanthomonadales:f__Xanthomonadaceae:g__Dokdonella
MID1	denovo758	1	k__Bacteria:p__Actinobacteria:c__Actinobacteria:o__Actinomycetales:f__:g__
MID1	denovo759	2	k__Bacteria:p__Proteobacteria:c__Alphaproteobacteria:o__Sphingomonadales:f__Sphingomonadaceae:g__Sphingomonas
MID1	denovo76	2	k__Bacteria:p__Chloroflexi:c__Ellin6529:o__:f__:g__
MID1	denovo761	2	k__Bacteria:p__Proteobacteria:c__Alphaproteobacteria:o__Rhodobacterales:f__Rhodobacteraceae:g__Paracoccus
MID1	denovo762	1	k__Bacteria:p__Firmicutes:c__Clostridia:o__Clostridiales:f__Veillonellaceae:g__Selenomonas
MID1	denovo763	3	k__Bacteria:p__Actinobacteria:c__Thermoleophilia:o__Gaiellales:f__Gaiellaceae:g__
MID1	denovo765	1	k__Bacteria:p__Actinobacteria:c__Thermoleophilia:o__Solirubrobacterales:f__:g__
MID1	denovo766	3	k__Bacteria:p__Actinobacteria:c__Actinobacteria:o__Actinomycetales:f__Microbacteriaceae:g__Agrococcus
MID1	denovo768	1	k__Bacteria:p__Proteobacteria:c__Alphaproteobacteria:o__Rhizobiales:f__Hyphomicrobiaceae:g__Devosia
MID1	denovo770	122	k__Bacteria:p__Cyanobacteria:c__Oscillatoriophycideae:o__Chroococcales:f__Xenococcaceae:g__
MID1	denovo771	1	k__Bacteria:p__Armatimonadetes:c__Armatimonadia:o__Armatimonadales:f__Armatimonadaceae:g__
MID1	denovo772	1	k__Bacteria:p__Proteobacteria:c__Alphaproteobacteria:o__Rhodobacterales:f__Rhodobacteraceae:g__
MID1	denovo774	1	k__Bacteria:p__Proteobacteria:c__Gammaproteobacteria:o__Xanthomonadales:f__Sinobacteraceae:g__
MID1	denovo775	1	k__Bacteria:p__Proteobacteria:c__Betaproteobacteria:o__Rhodocyclales:f__Rhodocyclaceae:g__Azoarcus
MID1	denovo777	6	k__Bacteria:p__Firmicutes:c__Clostridia:o__Clostridiales:f__Ruminococcaceae:g__
MID1	denovo779	2	k__Bacteria:p__Firmicutes:c__Bacilli:o__Bacillales:f__Bacillaceae:g__Bacillus
MID1	denovo78	1	k__Bacteria:p__Actinobacteria:c__Thermoleophilia:o__Solirubrobacterales:f__Conexibacteraceae:g__
MID1	denovo780	2	k__Bacteria:p__Cyanobacteria:c__ML635J-21:o__:f__:g__
MID1	denovo781	1	k__Bacteria:p__Proteobacteria:c__Alphaproteobacteria:o__Rhizobiales:f__:g__
MID1	denovo782	1	k__Bacteria:p__Proteobacteria:c__Betaproteobacteria:o__Burkholderiales:f__Oxalobacteraceae:g__
MID1	denovo783	2	k__Bacteria:p__Cyanobacteria:c__Oscillatoriophycideae:o__Oscillatoriales:f__Phormidiaceae:g__Phormidium
MID1	denovo785	30	k__Bacteria:p__Firmicutes:c__Clostridia:o__Clostridiales:f__:g__
MID1	denovo786	10	k__Bacteria:p__Firmicutes:c__Clostridia:o__Clostridiales
MID1	denovo787	1	k__Bacteria:p__Firmicutes:c__Clostridia:o__Clostridiales:f__[Mogibacteriaceae]:g__Mogibacterium
MID1	denovo788	1	k__Bacteria:p__Proteobacteria:c__Betaproteobacteria:o__Nitrosomonadales:f__Nitrosomonadaceae:g__
MID1	denovo79	1	k__Bacteria:p__Firmicutes:c__Clostridia:o__Clostridiales:f__[Tissierellaceae]:g__Anaerococcus
MID1	denovo790	10	k__Bacteria:p__Cyanobacteria:c__Chloroplast:o__Streptophyta:f__:g__
MID1	denovo791	28	k__Bacteria:p__Actinobacteria:c__Acidimicrobiia:o__Acidimicrobiales:f__:g__
MID1	denovo792	7	k__Bacteria:p__Actinobacteria:c__Actinobacteria:o__Actinomycetales:f__:g__
MID1	denovo794	1	k__Bacteria:p__Gemmatimonadetes:c__Gemmatimonadetes:o__Gemmatimonadales:f__:g__
MID1	denovo795	16	k__Bacteria:p__Actinobacteria:c__Actinobacteria:o__Actinomycetales:f__:g__
MID1	denovo796	3	k__Bacteria:p__Proteobacteria:c__Alphaproteobacteria:o__Rhodobacterales:f__Rhodobacteraceae:g__Rubellimicrobium

MID1	denovo799	2	k__Bacteria:p__Firmicutes:c__Clostridia:o__Clostridiales:f__Lachnospiraceae:g__Coprococcus
MID1	denovo80	1	k__Bacteria:p__Cyanobacteria:c__Synechococcophycideae:o__Pseudanabaenales:f__Pseudanabaenaceae:g__Leptolyngbya
MID1	denovo800	1	k__Bacteria:p__Armatimonadetes:c__Armatimonadia:o__Armatimonadales:f__Armatimonadaceae:g__
MID1	denovo801	4	k__Bacteria:p__Proteobacteria:c__Alphaproteobacteria:o__Rhodobacterales:f__Rhodobacteraceae:g__Rubellimicrobium
MID1	denovo802	1	k__Bacteria:p__Proteobacteria:c__Alphaproteobacteria:o__Rhodospirillales:f__Acetobacteraceae:g__
MID1	denovo803	17	k__Bacteria:p__Actinobacteria:c__Actinobacteria:o__Actinomycetales:f__Sporichthyaceae:g__
MID1	denovo804	1	k__Bacteria:p__Proteobacteria:c__Alphaproteobacteria:o__Rickettsiales:f__:g__
MID1	denovo806	2	k__Bacteria:p__Firmicutes:c__Bacilli:o__Bacillales:f__Bacillaceae:g__Bacillus
MID1	denovo807	2	k__Bacteria:p__Firmicutes:c__Clostridia:o__Clostridiales:f__Lachnospiraceae:g__
MID1	denovo810	1	k__Bacteria:p__Actinobacteria:c__Thermoleophilia:o__Gaiellales:f__Gaiellaceae:g__
MID1	denovo815	1	k__Bacteria:p__Firmicutes:c__Clostridia:o__Clostridiales:f__Lachnospiraceae:g__
MID1	denovo816	2	k__Bacteria:p__Firmicutes:c__Bacilli:o__Lactobacillales:f__Carnobacteriaceae:g__Desemzia
MID1	denovo817	2	k__Bacteria:p__Actinobacteria:c__Actinobacteria:o__Actinomycetales:f__Actinomycetaceae:g__Actinomyces
MID1	denovo82	2	k__Bacteria:p__Gemmatimonadetes:c__Gemmatimonadetes:o__Gemmatimonadales:f__:g__
MID1	denovo821	3	k__Bacteria:p__Proteobacteria:c__Alphaproteobacteria:o__Rhizobiales:f__Methylocystaceae:g__
MID1	denovo827	1	k__Bacteria:p__Actinobacteria:c__Actinobacteria:o__Actinomycetales:f__Nocardioidaceae:g__Nocardioides
MID1	denovo829	18	k__Bacteria:p__Firmicutes:c__Bacilli:o__Lactobacillales:f__Carnobacteriaceae:g__Carnobacterium
MID1	denovo830	3	k__Bacteria:p__Proteobacteria:c__Alphaproteobacteria:o__Rhodobacterales:f__Rhodobacteraceae:g__Paracoccus
MID1	denovo831	1	k__Bacteria:p__Actinobacteria:c__Actinobacteria:o__Actinomycetales:f__Propionibacteriaceae:g__
MID1	denovo833	2	k__Bacteria:p__Firmicutes:c__Clostridia:o__Clostridiales:f__Ruminococcaceae:g__Faecalibacterium
MID1	denovo834	11	k__Bacteria:p__Proteobacteria:c__Alphaproteobacteria:o__Sphingomonadales:f__Sphingomonadaceae:g__Sphingomonas
MID1	denovo836	16	k__Bacteria:p__Cyanobacteria:c__Oscillatoriophycideae:o__Chroococcales:f__Xenococcaceae
MID1	denovo837	1	k__Bacteria:p__Firmicutes:c__Clostridia:o__Clostridiales:f__Clostridiaceae:g__Clostridium
MID1	denovo838	1	k__Bacteria:p__Actinobacteria:c__Acidimicrobiia:o__Acidimicrobiales:f__:g__
MID1	denovo841	7	k__Bacteria:p__Proteobacteria:c__Alphaproteobacteria:o__Rhizobiales:f__:g__
MID1	denovo844	4	k__Bacteria:p__Proteobacteria:c__Betaproteobacteria:o__Burkholderiales:f__Comamonadaceae:g__
MID1	denovo847	12	k__Bacteria:p__Actinobacteria:c__Acidimicrobiia:o__Acidimicrobiales:f__C111:g__
MID1	denovo849	1	k__Bacteria:p__Proteobacteria:c__Alphaproteobacteria:o__Ellin329:f__:g__
MID1	denovo85	1	k__Bacteria:p__Proteobacteria:c__Deltaproteobacteria:o__Myxococcales:f__Nannocystaceae:g__Plesiocystis
MID1	denovo850	3	k__Bacteria:p__Cyanobacteria:c__Oscillatoriophycideae:o__Oscillatoriales:f__Phormidiaceae:g__Phormidium
MID1	denovo851	8	k__Bacteria:p__Proteobacteria:c__Alphaproteobacteria:o__Rhizobiales:f__Hyphomicrobiaceae:g__Devosia
MID1	denovo853	15	k__Bacteria:p__Proteobacteria:c__Betaproteobacteria:o__Burkholderiales:f__:g__
MID1	denovo855	1	k__Bacteria:p__Proteobacteria:c__Alphaproteobacteria:o__Rhodobacterales:f__Rhodobacteraceae
MID1	denovo858	44	k__Bacteria:p__Actinobacteria:c__Actinobacteria:o__Actinomycetales:f__Nocardioidaceae:g__Propionicimonas
MID1	denovo859	4	k__Bacteria:p__Firmicutes:c__Clostridia:o__Clostridiales:f__Lachnospiraceae:g__Anaerostipes
MID1	denovo86	3	k__Bacteria:p__Proteobacteria:c__Alphaproteobacteria:o__Sphingomonadales:f__Erythrobacteraceae:g__
MID1	denovo860	1	k__Bacteria:p__Firmicutes:c__Clostridia:o__Clostridiales:f__:g__
MID1	denovo862	3	k__Bacteria:p__FBP:c__:o__:f__:g__
MID1	denovo863	1	k__Bacteria:p__Actinobacteria:c__Actinobacteria:o__Actinomycetales:f__Intrasporangiaceae:g__
MID1	denovo864	1	k__Bacteria:p__Proteobacteria:c__Gammaproteobacteria:o__Legionellales:f__:g__
MID1	denovo866	1	k__Bacteria:p__Proteobacteria:c__Deltaproteobacteria:o__Spirobacillales:f__:g__
MID1	denovo868	1	k__Bacteria:p__Firmicutes:c__Clostridia:o__Clostridiales:f__[Tissierellaceae]:g__Anaerococcus
MID1	denovo87	1	k__Bacteria:p__Proteobacteria:c__Alphaproteobacteria:o__Caulobacterales:f__Caulobacteraceae:g__Phenylobacterium
MID1	denovo872	1	k__Bacteria:p__Acidobacteria:c__iii1-8:o__32-20:f__:g__
MID1	denovo873	1	k__Bacteria:p__Cyanobacteria:c__Synechococcophycideae:o__Pseudanabaenales:f__Pseudanabaenaceae:g__
MID1	denovo874	2	k__Bacteria:p__Proteobacteria:c__Alphaproteobacteria:o__Rhodobacterales:f__Rhodobacteraceae:g__Amaricoccus
MID1	denovo876	1	k__Bacteria:p__Actinobacteria:c__Actinobacteria:o__Actinomycetales:f__Mycobacteriaceae:g__Mycobacterium
MID1	denovo877	4	k__Bacteria:p__Proteobacteria:c__Alphaproteobacteria:o__Rhodobacterales:f__Rhodobacteraceae:g__
MID1	denovo878	17	k__Bacteria:p__Firmicutes:c__Bacilli:o__Lactobacillales:f__Streptococcaceae:g__Streptococcus
MID1	denovo880	6	k__Bacteria:p__Firmicutes:c__Clostridia:o__Clostridiales:f__Ruminococcaceae:g__Ruminococcus
MID1	denovo882	4	k__Bacteria:p__Cyanobacteria:c__Synechococcophycideae:o__Pseudanabaenales:f__Pseudanabaenaceae:g__Leptolyngbya
MID1	denovo885	14	k__Bacteria:p__Firmicutes:c__Clostridia:o__Clostridiales:f__Ruminococcaceae:g__
MID1	denovo887	3	k__Bacteria:p__Proteobacteria:c__Alphaproteobacteria:o__Rhizobiales:f__Beijerinckiaceae:g__
MID1	denovo897	4	k__Bacteria:p__Actinobacteria:c__Actinobacteria:o__Actinomycetales:f__Corynebacteriaceae:g__Corynebacterium
MID1	denovo899	1	k__Bacteria:p__Proteobacteria:c__Alphaproteobacteria:o__Rhizobiales:f__Hyphomicrobiaceae:g__Devosia
MID1	denovo901	2	k__Bacteria:p__Bacteroidetes:c__Cytophagia:o__Cytophagales:f__Cytophagaceae:g__Spirosoma
MID1	denovo902	20	k__Bacteria:p__Cyanobacteria:c__Oscillatoriophycideae:o__Chroococcales:f__Xenococcaceae:g__
MID1	denovo903	1	k__Bacteria:p__Bacteroidetes:c__Bacteroidia:o__Bacteroidales:f__Prevotellaceae:g__Prevotella
MID1	denovo905	1	k__Bacteria:p__Acidobacteria:c__Acidobacteria-6:o__iii1-15:f__mb2424:g__
MID1	denovo906	1	k__Bacteria:p__Proteobacteria:c__Betaproteobacteria:o__Burkholderiales:f__Oxalobacteraceae:g__
MID1	denovo907	1	k__Bacteria:p__Proteobacteria:c__Gammaproteobacteria:o__Xanthomonadales:f__Sinobacteraceae:g__
MID1	denovo91	2	k__Bacteria:p__Proteobacteria:c__Alphaproteobacteria:o__Caulobacterales:f__Caulobacteraceae:g__Brevundimonas
MID1	denovo910	1	k__Bacteria:p__Proteobacteria:c__Alphaproteobacteria:o__Rhodobacterales:f__Rhodobacteraceae:g__Rubellimicrobium
MID1	denovo911	2	k__Bacteria:p__Acidobacteria:c__[Chloracidobacteria]:o__RB41:f__Ellin6075:g__
MID1	denovo912	2	k__Bacteria:p__Proteobacteria:c__Alphaproteobacteria:o__Rhodobacterales:f__Rhodobacteraceae:g__Anaerospora
MID1	denovo913	1	k__Bacteria:p__Acidobacteria:c__Solibacteres:o__Solibacterales:f__:g__
MID1	denovo915	3	k__Bacteria:p__Actinobacteria:c__Acidimicrobiia:o__Acidimicrobiales:f__:g__

MID1	denovo916	1	k__Bacteria:p__Firmicutes:c__Clostridia:o__Clostridiales:f__Ruminococcaceae:g__
MID1	denovo917	1	k__Bacteria:p__Proteobacteria:c__Alphaproteobacteria:o__Rhodobacterales:f__Rhodobacteraceae
MID1	denovo918	5	k__Bacteria:p__Actinobacteria:c__Actinobacteria:o__Actinomycetales:f__Microbacteriaceae:g__Rathayibacter
MID1	denovo920	2	k__Bacteria:p__Proteobacteria:c__Gammaproteobacteria:o__Xanthomonadales:f__Xanthomonadaceae:g__
MID1	denovo922	1	k__Bacteria:p__Proteobacteria:c__Alphaproteobacteria:o__Rhizobiales:f__Bradyrhizobiaceae
MID1	denovo928	8	k__Bacteria:p__Proteobacteria:c__Alphaproteobacteria:o__Rhodospirillales:f__Acetobacteraceae:g__
MID1	denovo929	1	k__Bacteria:p__Actinobacteria:c__Actinobacteria:o__Actinomycetales:f__Corynebacteriaceae:g__Corynebacterium
MID1	denovo930	1	k__Bacteria:p__Proteobacteria:c__Alphaproteobacteria:o__Rhodospirillales:f__Acetobacteraceae:g__
MID1	denovo931	1	k__Bacteria:p__Cyanobacteria:c__:o__:f__:g__
MID1	denovo934	1	k__Bacteria:p__Proteobacteria:c__Alphaproteobacteria:o__Rhodobacterales:f__Rhodobacteraceae:g__Rhodobacter
MID1	denovo935	18	k__Bacteria:p__Proteobacteria:c__Gammaproteobacteria:o__Xanthomonadales:f__Xanthomonadaceae:g__Thermomonas
MID1	denovo936	4	k__Bacteria:p__Cyanobacteria:c__Oscillatoriophycideae:o__Oscillatoriales:f__Phormidiaceae:g__Phormidium
MID1	denovo939	58	k__Bacteria:p__Firmicutes:c__Clostridia:o__Clostridiales:f__Clostridiaceae:g__
MID1	denovo940	2	k__Bacteria:p__Proteobacteria:c__Alphaproteobacteria:o__Rhodobacterales:f__Rhodobacteraceae:g__Paracoccus
MID1	denovo941	1	k__Bacteria:p__Bacteroidetes:c__Flavobacteriia:o__Flavobacteriales:f__Flavobacteriaceae:g__Aequorivita
MID1	denovo944	48	k__Bacteria:p__Actinobacteria:c__Actinobacteria:o__Actinomycetales:f__Propionibacteriaceae:g__
MID1	denovo945	2	k__Bacteria:p__Cyanobacteria:c__Synechococcophycideae:o__Pseudanabaenales:f__Pseudanabaenaceae:g__Leptolyngbya
MID1	denovo946	3	k__Bacteria:p__Proteobacteria:c__Gammaproteobacteria:o__Xanthomonadales:f__Xanthomonadaceae:g__Lysobacter
MID1	denovo947	7	k__Bacteria:p__Firmicutes:c__Clostridia:o__Clostridiales:f__Ruminococcaceae:g__
MID1	denovo948	3	k__Bacteria:p__Cyanobacteria:c__Oscillatoriophycideae:o__Oscillatoriales:f__Phormidiaceae:g__Phormidium
MID1	denovo949	1	k__Bacteria:p__Proteobacteria:c__Alphaproteobacteria:o__Caulobacterales:f__Caulobacteraceae:g__
MID1	denovo95	2	k__Bacteria:p__Bacteroidetes:c__Flavobacteriia:o__Flavobacteriales:f__[Weeksellaceae]:g__Chryseobacterium
MID1	denovo951	1	k__Bacteria:p__Acidobacteria:c__[Chloracidobacteria]:o__RB41:f__Ellin6075:g__
MID1	denovo953	9	k__Bacteria:p__Actinobacteria:c__Actinobacteria:o__Actinomycetales:f__Microbacteriaceae:g__Curtobacterium
MID1	denovo954	1	k__Bacteria:p__Proteobacteria:c__Betaproteobacteria:o__SC-I-84:f__:g__
MID1	denovo955	1	k__Bacteria:p__Proteobacteria:c__Alphaproteobacteria:o__Rhizobiales:f__Methylobacteriaceae:g__Methylobacterium
MID1	denovo958	1	k__Bacteria:p__Proteobacteria:c__Alphaproteobacteria:o__Rhizobiales:f__Phyllobacteriaceae:g__
MID1	denovo96	5	k__Bacteria:p__Firmicutes:c__Clostridia:o__Clostridiales:f__[Tissierellaceae]:g__Anaerococcus
MID1	denovo960	1	k__Bacteria:p__Proteobacteria:c__Alphaproteobacteria:o__Rhodospirillales:f__Rhodospirillaceae:g__
MID1	denovo961	1	k__Bacteria:p__Proteobacteria:c__Deltaproteobacteria:o__GMD14H09:f__:g__
MID1	denovo963	6	k__Bacteria:p__Proteobacteria:c__Alphaproteobacteria:o__Sphingomonadales:f__Sphingomonadaceae:g__
MID1	denovo964	3	k__Bacteria:p__Proteobacteria:c__Gammaproteobacteria:o__Pseudomonadales:f__Moraxellaceae:g__Acinetobacter
MID1	denovo966	1	k__Bacteria:p__Actinobacteria:c__Actinobacteria:o__Actinomycetales:f__Intrasporangiaceae:g__Arsenicicoccus
MID1	denovo967	2	k__Bacteria:p__Gemmatimonadetes:c__Gemmatimonadetes:o__Gemmatimonadales:f__:g__
MID1	denovo968	18	k__Bacteria:p__Bacteroidetes:c__Flavobacteriia:o__Flavobacteriales:f__Flavobacteriaceae:g__Gelidibacter
MID1	denovo969	1	k__Bacteria:p__Proteobacteria:c__Gammaproteobacteria:o__Xanthomonadales:f__Xanthomonadaceae:g__Lysobacter
MID1	denovo97	1	k__Bacteria:p__Gemmatimonadetes:c__Gemm-3:o__:f__:g__
MID1	denovo970	1	k__Bacteria:p__Proteobacteria:c__Betaproteobacteria:o__Burkholderiales:f__Comamonadaceae:g__Hylemonella
MID1	denovo972	1	k__Bacteria:p__Firmicutes:c__Bacilli:o__Lactobacillales:f__Aerococcaceae:g__
MID1	denovo973	10	k__Bacteria:p__Firmicutes:c__Bacilli:o__Bacillales:f__Staphylococcaceae:g__Jeotgalicoccus
MID1	denovo975	1	k__Bacteria:p__Proteobacteria:c__Alphaproteobacteria:o__Sphingomonadales:f__Sphingomonadaceae:g__Sphingomonas
MID1	denovo977	6	k__Bacteria:p__Cyanobacteria:c__Nostocophycideae:o__Stigonematales:f__Rivulariaceae:g__Calothrix
MID1	denovo979	3	k__Bacteria:p__Proteobacteria:c__Alphaproteobacteria:o__Sphingomonadales:f__Sphingomonadaceae:g__Sphingomonas
MID1	denovo980	3	k__Bacteria:p__Actinobacteria:c__Thermoleophilia:o__Solirubrobacterales:f__Solirubrobacteraceae:g__
MID1	denovo981	10	k__Bacteria:p__Firmicutes:c__Bacilli:o__Lactobacillales:f__Leuconostocaceae:g__
MID1	denovo982	2	k__Bacteria:p__Proteobacteria:c__Alphaproteobacteria:o__Rhizobiales:f__Phyllobacteriaceae:g__
MID1	denovo984	10	k__Bacteria:p__Firmicutes:c__Clostridia:o__Clostridiales:f__[Tissierellaceae]:g__Tissierella_Soehngenia
MID1	denovo985	1	k__Bacteria:p__Proteobacteria:c__Gammaproteobacteria:o__Pseudomonadales:f__Pseudomonadaceae:g__
MID1	denovo987	1	k__Bacteria:p__Proteobacteria:c__Betaproteobacteria:o__Burkholderiales:f__Burkholderiaceae:g__Burkholderia
MID1	denovo988	6	k__Bacteria:p__Firmicutes:c__Bacilli:o__Lactobacillales:f__Enterococcaceae:g__Enterococcus
MID1	denovo989	1	k__Bacteria:p__Actinobacteria:c__Acidimicrobiia:o__Acidimicrobiales:f__EB1017:g__
MID1	denovo99	1	k__Bacteria:p__WPS-2:c__:o__:f__:g__
MID1	denovo990	1	k__Bacteria:p__Actinobacteria:c__Actinobacteria:o__Actinomycetales:f__Microbacteriaceae:g__Yonghaparkia
MID1	denovo993	1	k__Bacteria:p__Gemmatimonadetes:c__Gemmatimonadetes:o__Gemmatimonadales:f__:g__
MID1	denovo994	1	k__Bacteria:p__Proteobacteria:c__Alphaproteobacteria:o__Rhodobacterales:f__Rhodobacteraceae
MID1	denovo995	2	k__Bacteria:p__Cyanobacteria:c__Oscillatoriophycideae:o__Oscillatoriales:f__Phormidiaceae:g__Phormidium
MID1	denovo996	7	k__Bacteria:p__Actinobacteria:c__Actinobacteria:o__Actinomycetales:f__Intrasporangiaceae:g__
MID1	denovo997	4	k__Bacteria:p__Proteobacteria:c__Alphaproteobacteria:o__Sphingomonadales:f__Erythrobacteraceae:g__
MID1	denovo998	1	k__Bacteria:p__Actinobacteria:c__Actinobacteria:o__Actinomycetales:f__Nocardioidaceae:g__
MID2	denovo1014	2	k__Bacteria:p__Proteobacteria:c__Alphaproteobacteria:o__Rhizobiales:f__Bradyrhizobiaceae:g__Balneimonas
MID2	denovo1015	1	k__Bacteria:p__Firmicutes:c__Clostridia:o__Clostridiales:f__[Tissierellaceae]:g__1-68
MID2	denovo1025	10	k__Bacteria:p__Cyanobacteria:c__Nostocophycideae:o__Nostocales:f__Scytonemataceae:g__
MID2	denovo1036	1	k__Bacteria:p__Firmicutes:c__Bacilli:o__Bacillales:f__Bacillaceae
MID2	denovo1037	3	k__Bacteria:p__Actinobacteria:c__Actinobacteria:o__Actinomycetales:f__Corynebacteriaceae:g__Corynebacterium
MID2	denovo1038	1	k__Bacteria:p__Proteobacteria:c__Betaproteobacteria:o__Burkholderiales
MID2	denovo1041	1	k__Bacteria:p__Nitrospirae:c__Nitrospira:o__Nitrospirales:f__Nitrospiraceae:g__
MID2	denovo1043	11	k__Bacteria:p__Firmicutes:c__Bacilli:o__Bacillales:f__Planococcaceae

MID2	denovo1044	1	k__Bacteria:p__Firmicutes:c__Clostridia:o__Clostridiales:f__Lachnospiraceae:g__Coprococcus
MID2	denovo1045	1	k__Bacteria:p__Actinobacteria:c__Actinobacteria:o__Actinomycetales:f__Micrococcaceae:g__Kocuria
MID2	denovo1059	3	k__Bacteria:p__Actinobacteria:c__Actinobacteria:o__Actinomycetales:f__Microbacteriaceae:g__Microbacterium
MID2	denovo1062	1	k__Bacteria:p__Proteobacteria:c__Gammaproteobacteria:o__Legionellales:f__Coxiellaceae:g__
MID2	denovo1084	1	k__Bacteria:p__Proteobacteria:c__Alphaproteobacteria:o__Caulobacterales:f__Caulobacteraceae:g__
MID2	denovo1086	2	k__Bacteria:p__Actinobacteria:c__Actinobacteria:o__Actinomycetales:f__Nocardioidaceae:g__
MID2	denovo1093	1	k__Bacteria:p__Firmicutes:c__Bacilli:o__Lactobacillales:f__Lactobacillaceae:g__Lactobacillus
MID2	denovo1094	2	k__Bacteria:p__Firmicutes:c__Bacilli:o__Lactobacillales:f__Aerococcaceae:g__Facklamia
MID2	denovo1097	2	k__Bacteria:p__Proteobacteria:c__Gammaproteobacteria:o__Xanthomonadales:f__Xanthomonadaceae:g__
MID2	denovo110	1	k__Bacteria:p__Proteobacteria:c__Betaproteobacteria:o__Procabacteriales:f__Procabacteriaceae:g__
MID2	denovo1100	2	k__Bacteria:p__Firmicutes:c__Clostridia:o__Clostridiales:f__Lachnospiraceae:g__
MID2	denovo1121	1	k__Bacteria:p__Bacteroidetes:c__Flavobacteriia:o__Flavobacteriales:f__[Weeksellaceae]:g__Chryseobacterium
MID2	denovo1122	66	k__Bacteria:p__Proteobacteria:c__Gammaproteobacteria:o__Legionellales:f__Coxiellaceae:g__Rickettsiella
MID2	denovo113	1	k__Bacteria:p__Proteobacteria:c__Alphaproteobacteria:o__Rhizobiales:f__Rhizobiaceae:g__
MID2	denovo1132	1	k__Bacteria:p__Firmicutes:c__Bacilli:o__Lactobacillales:f__Enterococcaceae:g__Vagococcus
MID2	denovo1142	2	k__Bacteria:p__Acidobacteria:c__[Chloracidobacteria]:o__RB41:f__Ellin6075:g__
MID2	denovo1151	2	k__Bacteria:p__Firmicutes:c__Bacilli:o__Bacillales:f__Planococcaceae:g__Solibacillus
MID2	denovo1158	1	k__Bacteria:p__Firmicutes:c__Clostridia:o__Clostridiales:f__Lachnospiraceae:g__Epulopiscium
MID2	denovo1161	3	k__Bacteria:p__Firmicutes:c__Bacilli:o__Bacillales:f__Staphylococcaceae:g__Salinicoccus
MID2	denovo1165	2	k__Bacteria:p__Firmicutes:c__Clostridia:o__Clostridiales:f__[Tissierellaceae]:g__Anaerococcus
MID2	denovo117	1	k__Bacteria:p__Proteobacteria:c__Alphaproteobacteria:o__Rhodobacterales:f__Rhodobacteraceae:g__Amaricoccus
MID2	denovo1173	1	k__Bacteria:p__Proteobacteria:c__Alphaproteobacteria:o__Rhodobacterales:f__Rhodobacteraceae:g__Paracoccus
MID2	denovo1184	1	k__Bacteria:p__Proteobacteria:c__Alphaproteobacteria:o__Rhizobiales:f__Phyllobacteriaceae:g__
MID2	denovo1190	1	k__Bacteria:p__Actinobacteria:c__Actinobacteria:o__Actinomycetales:f__Micrococcaceae:g__Kocuria
MID2	denovo1193	1	k__Bacteria:p__Proteobacteria:c__Alphaproteobacteria:o__Rhodobacterales:f__Rhodobacteraceae:g__Rubellimicrobium
MID2	denovo1202	2	k__Bacteria:p__Proteobacteria:c__Alphaproteobacteria:o__Rhizobiales:f__Methylobacteriaceae:g__Methylobacterium
MID2	denovo1203	1	k__Bacteria:p__Proteobacteria:c__Alphaproteobacteria:o__Rhizobiales:f__Methylobacteriaceae:g__Methylobacterium
MID2	denovo1205	1	k__Bacteria:p__Actinobacteria:c__Acidimicrobiia:o__Acidimicrobiales:f__:g__
MID2	denovo1212	1	k__Bacteria:p__Proteobacteria:c__Alphaproteobacteria:o__Rhodobacterales:f__Rhodobacteraceae:g__Rhodobacter
MID2	denovo1215	1	k__Bacteria:p__Proteobacteria:c__Deltaproteobacteria:o__Myxococcales:f__:g__
MID2	denovo1218	2	k__Bacteria:p__Proteobacteria:c__Alphaproteobacteria:o__Sphingomonadales:f__Sphingomonadaceae:g__Sphingobium
MID2	denovo1220	1	k__Bacteria:p__Bacteroidetes:c__[Saprospirae]:o__[Saprospirales]:f__Saprospiraceae:g__
MID2	denovo1228	3	k__Bacteria:p__Firmicutes:c__Bacilli:o__Lactobacillales:f__Lactobacillaceae:g__Lactobacillus
MID2	denovo1230	3	k__Bacteria:p__Proteobacteria:c__Betaproteobacteria:o__Burkholderiales:f__Comamonadaceae:g__Comamonas
MID2	denovo1234	2	k__Bacteria:p__Actinobacteria:c__Thermoleophilia:o__Solirubrobacterales:f__:g__
MID2	denovo1241	2	k__Bacteria:p__Actinobacteria:c__Thermoleophilia:o__Solirubrobacterales:f__Patulibacteraceae:g__
MID2	denovo1248	1	k__Bacteria:p__Proteobacteria:c__Alphaproteobacteria:o__Rhodospirillales:f__Rhodospirillaceae:g__
MID2	denovo1250	3	k__Bacteria:p__Proteobacteria:c__Alphaproteobacteria:o__Sphingomonadales:f__Sphingomonadaceae:g__Kaistobacter
MID2	denovo1253	12	k__Bacteria:p__Proteobacteria:c__Alphaproteobacteria:o__Rhizobiales:f__Rhizobiaceae:g__Agrobacterium
MID2	denovo1254	2	k__Bacteria:p__Proteobacteria:c__Alphaproteobacteria:o__Caulobacterales:f__Caulobacteraceae:g__Mycoplana
MID2	denovo1257	3	k__Bacteria:p__Proteobacteria:c__Alphaproteobacteria:o__Sphingomonadales:f__Sphingomonadaceae
MID2	denovo1272	1	k__Bacteria:p__Proteobacteria:c__Alphaproteobacteria:o__Caulobacterales:f__Caulobacteraceae:g__
MID2	denovo1284	2	k__Bacteria:p__Proteobacteria:c__Alphaproteobacteria:o__Sphingomonadales:f__Sphingomonadaceae:g__Kaistobacter
MID2	denovo1290	2	k__Bacteria:p__Actinobacteria:c__Actinobacteria:o__Actinomycetales:f__Dermabacteraceae:g__Brachybacterium
MID2	denovo1298	1	k__Bacteria:p__Proteobacteria:c__Alphaproteobacteria:o__Caulobacterales:f__Caulobacteraceae:g__Brevundimonas
MID2	denovo1300	3	k__Bacteria:p__Actinobacteria:c__Actinobacteria:o__Actinomycetales:f__Nocardioidaceae:g__Friedmanniella
MID2	denovo1301	2	k__Bacteria:p__Actinobacteria:c__Coriobacteriia:o__Coriobacteriales:f__Coriobacteriaceae:g__Collinsella
MID2	denovo1302	2	k__Bacteria:p__Spirochaetes:c__Spirochaetes:o__Spirochaetales:f__Spirochaetaceae:g__Treponema
MID2	denovo1308	3	k__Bacteria:p__Firmicutes:c__Bacilli:o__Lactobacillales:f__Streptococcaceae:g__Streptococcus
MID2	denovo1311	5	k__Bacteria:p__Firmicutes:c__Clostridia:o__Clostridiales:f__Clostridiaceae:g__
MID2	denovo1334	23	k__Bacteria:p__Firmicutes:c__Bacilli:o__Lactobacillales:f__Streptococcaceae:g__Streptococcus
MID2	denovo134	6	k__Bacteria:p__Firmicutes:c__Bacilli:o__Lactobacillales:f__:g__
MID2	denovo1341	2	k__Bacteria:p__Firmicutes:c__Clostridia:o__Clostridiales:f__[Tissierellaceae]:g__Anaerococcus
MID2	denovo1346	2	k__Bacteria:p__Firmicutes:c__Bacilli:o__Lactobacillales:f__Streptococcaceae:g__Lactococcus
MID2	denovo1347	2	k__Bacteria:p__Proteobacteria:c__Gammaproteobacteria:o__Xanthomonadales:f__Xanthomonadaceae:g__Luteibacter
MID2	denovo1350	3	k__Bacteria:p__Firmicutes:c__Clostridia:o__Clostridiales:f__[Tissierellaceae]:g__Anaerococcus
MID2	denovo1357	1	k__Bacteria:p__Proteobacteria:c__Alphaproteobacteria:o__Rhodospirillales:f__Rhodospirillaceae:g__
MID2	denovo138	2	k__Bacteria:p__Proteobacteria:c__Alphaproteobacteria:o__Rhodospirillales:f__Rhodospirillaceae:g__Skermanella
MID2	denovo149	1	k__Bacteria:p__Actinobacteria:c__Actinobacteria:o__Actinomycetales:f__Cellulomonadaceae:g__Cellulomonas
MID2	denovo159	1	k__Bacteria:p__Actinobacteria:c__Actinobacteria:o__Actinomycetales:f__Propionibacteriaceae:g__Microlunatus
MID2	denovo166	2	k__Bacteria:p__Proteobacteria:c__Gammaproteobacteria:o__Enterobacteriales:f__Enterobacteriaceae:g__
MID2	denovo174	1	k__Bacteria:p__Proteobacteria:c__Alphaproteobacteria:o__Sphingomonadales:f__Sphingomonadaceae:g__
MID2	denovo178	2	k__Bacteria:p__Bacteroidetes:c__[Rhodothermi]:o__[Rhodothermales]:f__Rhodothermaceae:g__Rubricoccus
MID2	denovo18	6	k__Bacteria:p__Proteobacteria:c__Gammaproteobacteria:o__Pseudomonadales:f__Pseudomonadaceae:g__Pseudomonas
MID2	denovo183	11	k__Bacteria:p__Cyanobacteria:c__Oscillatoriophycideae:o__Chroococcales:f__Xenococcaceae
MID2	denovo184	6	k__Bacteria:p__Firmicutes:c__Clostridia:o__Clostridiales:f__[Tissierellaceae]:g__Peptoniphilus
MID2	denovo185	63	k__Bacteria:p__Proteobacteria:c__Gammaproteobacteria:o__Xanthomonadales:f__Xanthomonadaceae:g__

MID2	denovo187	3	k__Bacteria:p__Firmicutes:c__Clostridia:o__Clostridiales:f__Clostridiaceae:g__
MID2	denovo193	1	k__Bacteria:p__Proteobacteria:c__Alphaproteobacteria:o__Caulobacterales:f__Caulobacteraceae:g__Phenylobacterium
MID2	denovo199	1	k__Bacteria:p__Proteobacteria:c__Betaproteobacteria:o__Rhodocyclales:f__Rhodocyclaceae
MID2	denovo201	1	k__Bacteria:p__Firmicutes:c__Clostridia:o__Clostridiales:f__Peptostreptococcaceae:g__Peptostreptococcus
MID2	denovo207	3	k__Bacteria:p__Actinobacteria:c__Acidimicrobiia:o__Acidimicrobiales:f__C111:g__
MID2	denovo210	1	k__Bacteria:p__Proteobacteria:c__Gammaproteobacteria:o__Xanthomonadales:f__Xanthomonadaceae:g__
MID2	denovo213	2	k__Bacteria:p__Proteobacteria:c__Gammaproteobacteria:o__Xanthomonadales:f__Xanthomonadaceae:g__Luteimonas
MID2	denovo219	1	k__Bacteria:p__Proteobacteria:c__Alphaproteobacteria:o__Rhodobacterales:f__Rhodobacteraceae:g__Rubellimicrobium
MID2	denovo224	1	k__Bacteria:p__Actinobacteria:c__Actinobacteria:o__Actinomycetales:f__Microbacteriaceae:g__Agromyces
MID2	denovo228	1	k__Bacteria:p__Proteobacteria:c__Alphaproteobacteria:o__Sphingomonadales:f__Sphingomonadaceae:g__Sphingomonas
MID2	denovo232	3	k__Bacteria:p__Firmicutes:c__Clostridia:o__Clostridiales:f__[Tissierellaceae]:g__Anaerococcus
MID2	denovo244	1	k__Bacteria:p__Proteobacteria:c__Alphaproteobacteria:o__Caulobacterales:f__Caulobacteraceae
MID2	denovo245	1	k__Bacteria:p__Proteobacteria:c__Alphaproteobacteria:o__Rhizobiales:f__Methylobacteriaceae:g__Methylobacterium
MID2	denovo252	3	k__Bacteria:p__Actinobacteria:c__Actinobacteria:o__Actinomycetales:f__Intrasporangiaceae:g__Kytococcus
MID2	denovo253	2	k__Bacteria:p__Gemmatimonadetes:c__Gemmatimonadetes:o__N1423WL:f__:g__
MID2	denovo266	4	k__Bacteria:p__Proteobacteria:c__Alphaproteobacteria:o__Sphingomonadales:f__Erythrobacteraceae
MID2	denovo267	1	k__Bacteria:p__Proteobacteria:c__Alphaproteobacteria:o__Sphingomonadales:f__Sphingomonadaceae:g__Sphingomonas
MID2	denovo271	1	k__Bacteria:p__Actinobacteria:c__Thermoleophilia:o__Solirubrobacterales:f__:g__
MID2	denovo282	1	k__Bacteria:p__Proteobacteria:c__Gammaproteobacteria:o__Xanthomonadales:f__Xanthomonadaceae:g__Stenotrophomonas
MID2	denovo288	5	k__Bacteria:p__Proteobacteria:c__Alphaproteobacteria:o__Caulobacterales:f__Caulobacteraceae:g__
MID2	denovo289	3	k__Bacteria:p__Cyanobacteria:c__Synechococcophycideae:o__Pseudanabaenales:f__Pseudanabaenaceae:g__Leptolyngbya
MID2	denovo291	1	k__Bacteria:p__Proteobacteria:c__Betaproteobacteria:o__Burkholderiales:f__Comamonadaceae:g__Delftia
MID2	denovo293	2	k__Bacteria:p__Acidobacteria:c__Solibacteres:o__Solibacterales:f__:g__
MID2	denovo294	1	k__Bacteria:p__Firmicutes:c__Clostridia:o__Clostridiales:f__[Tissierellaceae]:g__Peptoniphilus
MID2	denovo30	1	k__Bacteria:p__Actinobacteria:c__Actinobacteria:o__Actinomycetales:f__Bogoriellaceae:g__Georgenia
MID2	denovo306	3	k__Bacteria:p__Bacteroidetes:c__Flavobacteriia:o__Flavobacteriales:f__Flavobacteriaceae:g__Aequorivita
MID2	denovo318	1	k__Bacteria:p__Cyanobacteria:c__Oscillatoriophycideae:o__Chroococcales:f__Xenococcaceae:g__
MID2	denovo322	1	k__Bacteria:p__Proteobacteria:c__Alphaproteobacteria:o__Rhodobacterales:f__Rhodobacteraceae:g__
MID2	denovo323	1	k__Bacteria:p__Proteobacteria:c__Gammaproteobacteria:o__Pseudomonadales:f__Pseudomonadaceae:g__Pseudomonas
MID2	denovo353	8	k__Bacteria:p__Proteobacteria:c__Alphaproteobacteria:o__Rhodobacterales:f__Rhodobacteraceae:g__Rubellimicrobium
MID2	denovo361	20	k__Bacteria:p__Firmicutes:c__Bacilli:o__Lactobacillales:f__Lactobacillaceae:g__Lactobacillus
MID2	denovo374	1	k__Bacteria:p__Actinobacteria:c__Actinobacteria:o__Actinomycetales:f__Microbacteriaceae:g__Cryocola
MID2	denovo388	3	k__Bacteria:p__Proteobacteria:c__Gammaproteobacteria:o__Pseudomonadales:f__Pseudomonadaceae:g__Pseudomonas
MID2	denovo391	1	k__Bacteria:p__Bacteroidetes:c__Cytophagia:o__Cytophagales:f__Cytophagaceae:g__Rudanella
MID2	denovo393	5	k__Bacteria:p__Proteobacteria:c__Gammaproteobacteria:o__Xanthomonadales:f__Xanthomonadaceae:g__Lysobacter
MID2	denovo40	1	k__Bacteria:p__Proteobacteria:c__Alphaproteobacteria:o__Sphingomonadales:f__Erythrobacteraceae:g__
MID2	denovo404	4	k__Bacteria:p__Firmicutes:c__Bacilli:o__Lactobacillales:f__Enterococcaceae:g__Enterococcus
MID2	denovo429	1	k__Bacteria:p__Firmicutes:c__Clostridia:o__Clostridiales:f__Clostridiaceae:g__Clostridium
MID2	denovo43	1	k__Bacteria:p__Actinobacteria:c__Actinobacteria:o__Actinomycetales:f__:g__
MID2	denovo439	2	k__Bacteria:p__Firmicutes:c__Bacilli:o__Turicibacterales:f__Turicibacteraceae:g__Turicibacter
MID2	denovo443	1	k__Bacteria:p__Proteobacteria:c__Alphaproteobacteria:o__Rhizobiales:f__Bradyrhizobiaceae:g__
MID2	denovo454	1	k__Bacteria:p__Proteobacteria:c__Alphaproteobacteria:o__Sphingomonadales:f__Sphingomonadaceae:g__Kaistobacter
MID2	denovo464	3	k__Bacteria:p__Proteobacteria:c__Alphaproteobacteria:o__Rhodospirillales:f__:g__
MID2	denovo468	2	k__Bacteria:p__Firmicutes:c__Clostridia:o__Clostridiales:f__Ruminococcaceae:g__
MID2	denovo470	1	k__Bacteria:p__Actinobacteria:c__Actinobacteria:o__Actinomycetales:f__:g__
MID2	denovo482	1	k__Bacteria:p__Actinobacteria:c__Thermoleophilia:o__Solirubrobacterales:f__:g__
MID2	denovo485	1	k__Bacteria:p__Firmicutes:c__Clostridia:o__Clostridiales:f__Lachnospiraceae:g__Blautia
MID2	denovo489	1	k__Bacteria:p__Proteobacteria:c__Gammaproteobacteria:o__Pseudomonadales:f__Pseudomonadaceae:g__
MID2	denovo492	2	k__Bacteria:p__Cyanobacteria:c__Oscillatoriophycideae:o__Chroococcales:f__Xenococcaceae:g__Chroococcidiopsis
MID2	denovo499	1	k__Bacteria:p__Firmicutes:c__Clostridia:o__Clostridiales:f__Lachnospiraceae:g__Roseburia
MID2	denovo502	1	k__Bacteria:p__Firmicutes:c__Bacilli:o__Lactobacillales:f__Leuconostocaceae:g__Leuconostoc
MID2	denovo505	1	k__Bacteria:p__Acidobacteria:c__Sva0725:o__Sva0725:f__:g__
MID2	denovo506	1	k__Bacteria:p__Proteobacteria:c__Gammaproteobacteria:o__Enterobacteriales:f__Enterobacteriaceae
MID2	denovo510	1	k__Bacteria:p__Firmicutes:c__Clostridia:o__Clostridiales:f__[Mogibacteriaceae]:g__
MID2	denovo514	2	k__Bacteria:p__Cyanobacteria:c__Chloroplast:o__Stramenopiles:f__:g__
MID2	denovo520	1	k__Bacteria:p__Acidobacteria:c__[Chloracidobacteria]:o__RB41:f__Ellin6075:g__
MID2	denovo524	1	k__Bacteria:p__Acidobacteria:c__[Chloracidobacteria]:o__RB41:f__Ellin6075:g__
MID2	denovo530	3	k__Bacteria:p__Firmicutes:c__Bacilli:o__Lactobacillales:f__Lactobacillaceae:g__Lactobacillus
MID2	denovo542	25	k__Bacteria:p__Firmicutes:c__Clostridia:o__Clostridiales:f__[Tissierellaceae]:g__Finegoldia
MID2	denovo548	1	k__Bacteria:p__Firmicutes:c__Clostridia:o__Clostridiales:f__Lachnospiraceae:g__
MID2	denovo56	1	k__Bacteria:p__Proteobacteria:c__Alphaproteobacteria:o__Rhodobacterales:f__Rhodobacteraceae:g__Rubellimicrobium
MID2	denovo568	5	k__Bacteria:p__Proteobacteria:c__Alphaproteobacteria:o__Sphingomonadales:f__Sphingomonadaceae:g__Sphingobium
MID2	denovo57	1	k__Bacteria:p__Proteobacteria:c__Alphaproteobacteria:o__Sphingomonadales:f__Sphingomonadaceae
MID2	denovo58	3	k__Bacteria:p__Firmicutes:c__Bacilli:o__Lactobacillales:f__Lactobacillaceae:g__Lactobacillus
MID2	denovo592	4	k__Bacteria:p__Firmicutes:c__Clostridia:o__Clostridiales:f__Clostridiaceae:g__
MID2	denovo595	1	k__Bacteria:p__Proteobacteria:c__Alphaproteobacteria:o__Rhodobacterales:f__Rhodobacteraceae:g__Amaricoccus
MID2	denovo596	1	k__Bacteria:p__Firmicutes:c__Bacilli:o__Bacillales:f__Bacillaceae:g__Geobacillus

MID2	denovo600	3	k__Bacteria:p__Cyanobacteria:c__Oscillatoriophycideae:o__Oscillatoriales:f__Phormidiaceae:g__Phormidium
MID2	denovo605	3	k__Bacteria:p__Firmicutes:c__Bacilli:o__Gemellales:f__Gemellaceae:g__Gemella
MID2	denovo61	2	k__Bacteria:p__Actinobacteria:c__Actinobacteria:o__Actinomycetales:f__Actinomycetaceae:g__Actinomyces
MID2	denovo624	1	k__Bacteria:p__Proteobacteria:c__Gammaproteobacteria:o__Xanthomonadales:f__Xanthomonadaceae:g__
MID2	denovo626	1	k__Bacteria:p__Cyanobacteria:c__Oscillatoriophycideae:o__Chroococcales:f__:g__
MID2	denovo628	1	k__Bacteria:p__Proteobacteria:c__Gammaproteobacteria:o__Xanthomonadales:f__Xanthomonadaceae:g__Arenimonas
MID2	denovo63	53	k__Bacteria:p__Firmicutes:c__Bacilli:o__Lactobacillales:f__Lactobacillaceae:g__Lactobacillus
MID2	denovo630	1	k__Bacteria:p__Bacteroidetes:c__Flavobacteriia:o__Flavobacteriales:f__[Weeksellaceae]:g__Chryseobacterium
MID2	denovo631	3	k__Bacteria:p__Firmicutes:c__Clostridia:o__Clostridiales:f__:g__
MID2	denovo632	1	k__Bacteria:p__FBP:c__:o__:f__:g__
MID2	denovo633	4	k__Bacteria:p__Armatimonadetes:c__Armatimonadia:o__Armatimonadales:f__Armatimonadaceae:g__
MID2	denovo635	1	k__Bacteria:p__Proteobacteria:c__Betaproteobacteria:o__SC-I-84:f__:g__
MID2	denovo639	3	k__Bacteria:p__Actinobacteria:c__Actinobacteria:o__Actinomycetales:f__Geodermatophilaceae:g__Modestobacter
MID2	denovo64	1	k__Bacteria:p__Proteobacteria:c__Betaproteobacteria:o__Burkholderiales:f__Comamonadaceae
MID2	denovo640	1	k__Bacteria:p__Firmicutes:c__Clostridia:o__Clostridiales:f__Ruminococcaceae:g__
MID2	denovo642	1	k__Bacteria:p__Proteobacteria:c__Alphaproteobacteria:o__Sphingomonadales:f__Erythrobacteraceae:g__Lutibacterium
MID2	denovo65	3	k__Bacteria:p__Firmicutes:c__Bacilli:o__Bacillales:f__[Exiguobacteraceae]:g__Exiguobacterium
MID2	denovo655	2	k__Bacteria:p__Firmicutes:c__Clostridia:o__Clostridiales:f__[Tissierellaceae]:g__Anaerococcus
MID2	denovo661	1	k__Bacteria:p__Actinobacteria:c__Actinobacteria:o__Actinomycetales:f__:g__
MID2	denovo662	1	k__Bacteria:p__Bacteroidetes:c__Bacteroidia:o__Bacteroidales:f__Prevotellaceae:g__Prevotella
MID2	denovo665	5	k__Bacteria:p__Firmicutes:c__Bacilli:o__Lactobacillales:f__Lactobacillaceae:g__Lactobacillus
MID2	denovo666	1	k__Bacteria:p__Firmicutes:c__Bacilli:o__Lactobacillales:f__Aerococcaceae:g__Aerococcus
MID2	denovo667	1	k__Bacteria:p__Actinobacteria:c__Thermoleophilia:o__Solirubrobacterales:f__:g__
MID2	denovo668	1	k__Bacteria:p__Proteobacteria:c__Betaproteobacteria:o__Neisseriales:f__Neisseriaceae:g__Neisseria
MID2	denovo672	1	k__Bacteria:p__Firmicutes:c__Bacilli:o__Bacillales:f__:g__
MID2	denovo699	1	k__Bacteria:p__Proteobacteria:c__Alphaproteobacteria:o__Rhodospirillales:f__Acetobacteraceae:g__
MID2	denovo700	2	k__Bacteria:p__Proteobacteria:c__Gammaproteobacteria:o__Xanthomonadales:f__Xanthomonadaceae:g__Pseudoxanthomonas
MID2	denovo709	15	k__Bacteria:p__Proteobacteria:c__Gammaproteobacteria:o__Pseudomonadales:f__Moraxellaceae:g__Enhydrobacter
MID2	denovo710	2	k__Bacteria:p__Proteobacteria:c__Alphaproteobacteria:o__Sphingomonadales:f__:g__
MID2	denovo713	2	k__Bacteria:p__Bacteroidetes:c__Sphingobacteriia:o__Sphingobacteriales:f__Sphingobacteriaceae:g__Sphingobacterium
MID2	denovo729	3	k__Bacteria:p__Proteobacteria:c__Alphaproteobacteria:o__Rhizobiales:f__Phyllobacteriaceae
MID2	denovo733	1	k__Bacteria:p__Proteobacteria:c__Gammaproteobacteria:o__Xanthomonadales:f__Xanthomonadaceae:g__
MID2	denovo734	2	k__Bacteria:p__Proteobacteria:c__Betaproteobacteria:o__Neisseriales:f__Neisseriaceae:g__Neisseria
MID2	denovo748	9	k__Bacteria:p__Proteobacteria:c__Gammaproteobacteria:o__Xanthomonadales:f__Xanthomonadaceae:g__Lysobacter
MID2	denovo75	9	k__Bacteria:p__Proteobacteria:c__Betaproteobacteria:o__Burkholderiales:f__Comamonadaceae:g__Comamonas
MID2	denovo760	1	k__Bacteria:p__Firmicutes:c__Bacilli:o__Bacillales:f__Paenibacillaceae:g__Paenibacillus
MID2	denovo763	6	k__Bacteria:p__Actinobacteria:c__Thermoleophilia:o__Gaiellales:f__Gaiellaceae:g__
MID2	denovo770	11	k__Bacteria:p__Cyanobacteria:c__Oscillatoriophycideae:o__Chroococcales:f__Xenococcaceae:g__
MID2	denovo777	2	k__Bacteria:p__Firmicutes:c__Clostridia:o__Clostridiales:f__Ruminococcaceae:g__
MID2	denovo778	1	k__Bacteria:p__Fusobacteria:c__Fusobacteriia:o__Fusobacteriales:f__Fusobacteriaceae:g__Fusobacterium
MID2	denovo79	4	k__Bacteria:p__Firmicutes:c__Clostridia:o__Clostridiales:f__[Tissierellaceae]:g__Anaerococcus
MID2	denovo798	1	k__Bacteria:p__Proteobacteria:c__Deltaproteobacteria:o__Myxococcales:f__:g__
MID2	denovo803	3	k__Bacteria:p__Actinobacteria:c__Actinobacteria:o__Actinomycetales:f__Sporichthyaceae:g__
MID2	denovo805	1	k__Bacteria:p__Firmicutes:c__Clostridia:o__Clostridiales:f__[Mogibacteriaceae]:g__
MID2	denovo807	3	k__Bacteria:p__Firmicutes:c__Clostridia:o__Clostridiales:f__Lachnospiraceae:g__
MID2	denovo808	1	k__Bacteria:p__Proteobacteria:c__Alphaproteobacteria:o__Sphingomonadales:f__Sphingomonadaceae:g__Sphingomonas
MID2	denovo812	65	k__Bacteria:p__Bacteroidetes:c__Flavobacteriia:o__Flavobacteriales:f__[Weeksellaceae]:g__Wautersiella
MID2	denovo813	1	k__Bacteria:p__Proteobacteria:c__Gammaproteobacteria:o__Xanthomonadales:f__Xanthomonadaceae:g__Stenotrophomonas
MID2	denovo828	2	k__Bacteria:p__Firmicutes:c__Clostridia:o__Clostridiales:f__Clostridiaceae:g__Clostridium
MID2	denovo829	6	k__Bacteria:p__Firmicutes:c__Bacilli:o__Lactobacillales:f__Carnobacteriaceae:g__Carnobacterium
MID2	denovo83	1	k__Bacteria:p__Actinobacteria:c__Actinobacteria:o__Actinomycetales:f__Corynebacteriaceae:g__Corynebacterium
MID2	denovo831	1	k__Bacteria:p__Actinobacteria:c__Actinobacteria:o__Actinomycetales:f__Propionibacteriaceae:g__
MID2	denovo836	1	k__Bacteria:p__Cyanobacteria:c__Oscillatoriophycideae:o__Chroococcales:f__Xenococcaceae
MID2	denovo838	1	k__Bacteria:p__Actinobacteria:c__Acidimicrobiia:o__Acidimicrobiales:f__:g__
MID2	denovo840	1	k__Bacteria:p__Gemmatimonadetes:c__Gemm-1:o__:f__:g__
MID2	denovo841	4	k__Bacteria:p__Proteobacteria:c__Alphaproteobacteria:o__Rhizobiales:f__:g__
MID2	denovo842	1	k__Bacteria:p__Firmicutes:c__Bacilli:o__Lactobacillales:f__Lactobacillaceae:g__Lactobacillus
MID2	denovo844	1	k__Bacteria:p__Proteobacteria:c__Betaproteobacteria:o__Burkholderiales:f__Comamonadaceae:g__
MID2	denovo845	13	k__Bacteria:p__Proteobacteria:c__Gammaproteobacteria:o__Xanthomonadales:f__Xanthomonadaceae:g__Stenotrophomonas
MID2	denovo858	1	k__Bacteria:p__Actinobacteria:c__Actinobacteria:o__Actinomycetales:f__Nocardioidaceae:g__Propionicimonas
MID2	denovo868	1	k__Bacteria:p__Firmicutes:c__Clostridia:o__Clostridiales:f__[Tissierellaceae]:g__Anaerococcus
MID2	denovo87	2	k__Bacteria:p__Proteobacteria:c__Alphaproteobacteria:o__Caulobacterales:f__Caulobacteraceae:g__Phenylobacterium
MID2	denovo874	2	k__Bacteria:p__Proteobacteria:c__Alphaproteobacteria:o__Rhodobacterales:f__Rhodobacteraceae:g__Amaricoccus
MID2	denovo878	2	k__Bacteria:p__Firmicutes:c__Bacilli:o__Lactobacillales:f__Streptococcaceae:g__Streptococcus
MID2	denovo880	1	k__Bacteria:p__Firmicutes:c__Clostridia:o__Clostridiales:f__Ruminococcaceae:g__Ruminococcus
MID2	denovo881	1	k__Bacteria:p__Firmicutes:c__Clostridia:o__Clostridiales:f__[Tissierellaceae]:g__1-68
MID2	denovo882	1	k__Bacteria:p__Cyanobacteria:c__Synechococcophycideae:o__Pseudanabaenales:f__Pseudanabaenaceae:g__Leptolyngbya

MID2	denovo885	1	k__Bacteria:p__Firmicutes:c__Clostridia:o__Clostridiales:f__Ruminococcaceae:g__
MID2	denovo886	2	k__Bacteria:p__Firmicutes:c__Clostridia:o__Clostridiales:f__[Tissierellaceae]:g__Peptoniphilus
MID2	denovo888	2	k__Bacteria:p__Proteobacteria:c__Alphaproteobacteria:o__Rhodobacterales:f__Rhodobacteraceae:g__
MID2	denovo893	5	k__Bacteria:p__Firmicutes:c__Bacilli:o__Lactobacillales:f__Lactobacillaceae:g__Lactobacillus
MID2	denovo894	1	k__Bacteria:p__Firmicutes:c__Clostridia:o__MBA08:f__:g__
MID2	denovo895	1	k__Bacteria:p__Proteobacteria:c__Betaproteobacteria:o__Burkholderiales:f__Comamonadaceae:g__
MID2	denovo896	1	k__Bacteria:p__Bacteroidetes:c__Bacteroidia:o__Bacteroidales:f__Prevotellaceae:g__Prevotella
MID2	denovo9	1	k__Bacteria:p__Actinobacteria:c__Thermoleophilia:o__Gaiellales:f__Gaiellaceae:g__
MID2	denovo91	3	k__Bacteria:p__Proteobacteria:c__Alphaproteobacteria:o__Caulobacterales:f__Caulobacteraceae:g__Brevundimonas
MID2	denovo911	1	k__Bacteria:p__Acidobacteria:c__[Chloracidobacteria]:o__RB41:f__Ellin6075:g__
MID2	denovo92	9	k__Bacteria:p__Firmicutes:c__Bacilli:o__Lactobacillales:f__Lactobacillaceae:g__Lactobacillus
MID2	denovo924	1	k__Bacteria:p__Proteobacteria:c__Alphaproteobacteria:o__Sphingomonadales:f__Sphingomonadaceae:g__Sphingomonas
MID2	denovo935	1	k__Bacteria:p__Proteobacteria:c__Gammaproteobacteria:o__Xanthomonadales:f__Xanthomonadaceae:g__Thermomonas
MID2	denovo942	1	k__Bacteria:p__Proteobacteria:c__Gammaproteobacteria:o__Alteromonadales:f__[Chromatiaceae]:g__Rheinheimera
MID2	denovo95	3	k__Bacteria:p__Bacteroidetes:c__Flavobacteriia:o__Flavobacteriales:f__[Weeksellaceae]:g__Chryseobacterium
MID2	denovo953	3	k__Bacteria:p__Actinobacteria:c__Actinobacteria:o__Actinomycetales:f__Microbacteriaceae:g__Curtobacterium
MID2	denovo955	1	k__Bacteria:p__Proteobacteria:c__Alphaproteobacteria:o__Rhizobiales:f__Methylobacteriaceae:g__Methylobacterium
MID2	denovo96	4	k__Bacteria:p__Firmicutes:c__Clostridia:o__Clostridiales:f__[Tissierellaceae]:g__Anaerococcus
MID2	denovo964	8	k__Bacteria:p__Proteobacteria:c__Gammaproteobacteria:o__Pseudomonadales:f__Moraxellaceae:g__Acinetobacter
MID2	denovo965	2	k__Bacteria:p__Firmicutes:c__Bacilli:o__Lactobacillales:f__Aerococcaceae:g__Facklamia
MID2	denovo971	1	k__Bacteria:p__Firmicutes:c__Clostridia:o__Clostridiales:f__Lachnospiraceae:g__
MID2	denovo977	1	k__Bacteria:p__Cyanobacteria:c__Nostocophycideae:o__Stigonematales:f__Rivulariaceae:g__Calothrix
MID2	denovo980	2	k__Bacteria:p__Actinobacteria:c__Thermoleophilia:o__Solirubrobacterales:f__Solirubrobacteraceae:g__
MID2	denovo997	2	k__Bacteria:p__Proteobacteria:c__Alphaproteobacteria:o__Sphingomonadales:f__Erythrobacteraceae:g__
MID2	denovo999	1	k__Bacteria:p__Proteobacteria:c__Alphaproteobacteria:o__Rhizobiales:f__:g__
MID3	denovo0	3	k__Bacteria:p__Actinobacteria:c__Actinobacteria:o__Actinomycetales:f__Actinomycetaceae:g__Actinomyces
MID3	denovo1	2	k__Bacteria:p__Acidobacteria:c__Solibacteres:o__Solibacterales:f__:g__
MID3	denovo1000	3	k__Bacteria:p__Bacteroidetes:c__Flavobacteriia:o__Flavobacteriales:f__Flavobacteriaceae:g__Flavobacterium
MID3	denovo1002	1	k__Bacteria:p__Firmicutes:c__Clostridia:o__Clostridiales:f__[Tissierellaceae]:g__Anaerococcus
MID3	denovo1009	1	k__Bacteria:p__Acidobacteria:c__[Chloracidobacteria]:o__RB41:f__Ellin6075:g__
MID3	denovo101	1	k__Bacteria:p__Actinobacteria:c__Actinobacteria:o__Actinomycetales:f__Propionibacteriaceae:g__Propionibacterium
MID3	denovo1011	1	k__Bacteria:p__Planctomycetes:c__Planctomycetia:o__Pirellulales:f__Pirellulaceae:g__Pirellula
MID3	denovo1014	11	k__Bacteria:p__Proteobacteria:c__Alphaproteobacteria:o__Rhizobiales:f__Bradyrhizobiaceae:g__Balneimonas
MID3	denovo1016	1	k__Bacteria:p__Firmicutes:c__Clostridia:o__Clostridiales:f__Lachnospiraceae:g__[Ruminococcus]
MID3	denovo1018	10	k__Bacteria:p__Firmicutes:c__Clostridia:o__Clostridiales:f__[Tissierellaceae]:g__Peptoniphilus
MID3	denovo1026	4	k__Bacteria:p__Proteobacteria:c__Gammaproteobacteria:o__Xanthomonadales:f__Xanthomonadaceae:g__Lysobacter
MID3	denovo1028	1	k__Bacteria:p__Firmicutes:c__Bacilli:o__Bacillales:f__Staphylococcaceae:g__Staphylococcus
MID3	denovo1033	1	k__Bacteria:p__Firmicutes:c__Clostridia:o__Clostridiales:f__Ruminococcaceae:g__
MID3	denovo1037	1	k__Bacteria:p__Actinobacteria:c__Actinobacteria:o__Actinomycetales:f__Corynebacteriaceae:g__Corynebacterium
MID3	denovo1043	1	k__Bacteria:p__Firmicutes:c__Bacilli:o__Bacillales:f__Planococcaceae
MID3	denovo1044	6	k__Bacteria:p__Firmicutes:c__Clostridia:o__Clostridiales:f__Lachnospiraceae:g__Coprococcus
MID3	denovo1045	3	k__Bacteria:p__Actinobacteria:c__Actinobacteria:o__Actinomycetales:f__Micrococcaceae:g__Kocuria
MID3	denovo1051	2	k__Bacteria:p__Firmicutes:c__Clostridia:o__Clostridiales:f__:g__
MID3	denovo1054	8	k__Bacteria:p__Firmicutes:c__Bacilli:o__Bacillales:f__Bacillaceae:g__Bacillus
MID3	denovo1059	9	k__Bacteria:p__Actinobacteria:c__Actinobacteria:o__Actinomycetales:f__Microbacteriaceae:g__Microbacterium
MID3	denovo1064	4	k__Bacteria:p__Proteobacteria:c__Alphaproteobacteria:o__Rhizobiales:f__Hyphomicrobiaceae:g__
MID3	denovo107	1	k__Bacteria:p__Proteobacteria:c__Alphaproteobacteria:o__Rhodobacterales:f__Rhodobacteraceae:g__Rubellimicrobium
MID3	denovo1070	1	k__Bacteria:p__Firmicutes:c__Clostridia:o__Clostridiales:f__[Tissierellaceae]:g__Anaerococcus
MID3	denovo1071	5	k__Bacteria:p__Bacteroidetes:c__Cytophagia:o__Cytophagales:f__Cytophagaceae:g__
MID3	denovo1073	1	k__Bacteria:p__Proteobacteria:c__Gammaproteobacteria:o__Xanthomonadales:f__Xanthomonadaceae:g__Stenotrophomonas
MID3	denovo1074	1	k__Bacteria:p__Proteobacteria:c__Alphaproteobacteria:o__Rhodobacterales:f__Rhodobacteraceae:g__Rubellimicrobium
MID3	denovo1076	2	k__Bacteria:p__Bacteroidetes:c__Bacteroidia:o__Bacteroidales:f__[Paraprevotellaceae]:g__CF231
MID3	denovo1077	1	k__Bacteria:p__Proteobacteria:c__Alphaproteobacteria:o__Rhizobiales:f__Hyphomicrobiaceae:g__Devosia
MID3	denovo1082	2	k__Bacteria:p__Firmicutes:c__Clostridia:o__Clostridiales:f__Clostridiaceae:g__Clostridium
MID3	denovo1085	1	k__Bacteria:p__Actinobacteria:c__Acidimicrobiia:o__Acidimicrobiales:f__C111:g__
MID3	denovo1086	6	k__Bacteria:p__Actinobacteria:c__Actinobacteria:o__Actinomycetales:f__Nocardioidaceae:g__
MID3	denovo1089	1	k__Bacteria:p__Proteobacteria:c__Alphaproteobacteria:o__Rhodospirillales:f__Acetobacteraceae:g__
MID3	denovo109	6	k__Bacteria:p__Proteobacteria:c__Gammaproteobacteria:o__Enterobacteriales:f__Enterobacteriaceae
MID3	denovo1090	5	k__Bacteria:p__Proteobacteria:c__Alphaproteobacteria:o__Rhodobacterales:f__Rhodobacteraceae:g__Amaricoccus
MID3	denovo1094	3	k__Bacteria:p__Firmicutes:c__Bacilli:o__Lactobacillales:f__Aerococcaceae:g__Facklamia
MID3	denovo1101	1	k__Bacteria:p__Proteobacteria:c__Alphaproteobacteria:o__Rhodobacterales:f__Rhodobacteraceae:g__Rubellimicrobium
MID3	denovo1108	2	k__Bacteria:p__Proteobacteria:c__Alphaproteobacteria:o__Caulobacterales:f__Caulobacteraceae
MID3	denovo1110	1	k__Bacteria:p__Bacteroidetes:c__Flavobacteriia:o__Flavobacteriales:f__Flavobacteriaceae:g__Flavobacterium
MID3	denovo1114	1	k__Bacteria:p__Proteobacteria:c__Alphaproteobacteria:o__Rhizobiales:f__Bradyrhizobiaceae:g__
MID3	denovo1116	1	k__Bacteria:p__Proteobacteria:c__Alphaproteobacteria:o__Rhodospirillales:f__Acetobacteraceae:g__
MID3	denovo1120	1	k__Bacteria:p__Firmicutes:c__Clostridia:o__Clostridiales:f__[Tissierellaceae]:g__Helcococcus
MID3	denovo1123	2	k__Bacteria:p__Proteobacteria:c__Alphaproteobacteria:o__Sphingomonadales:f__Sphingomonadaceae:g__Sphingomonas

MID3	denovo1125	1	k__Bacteria:p__Acidobacteria:c__[Chloracidobacteria]:o__RB41:f__Ellin6075:g__
MID3	denovo1130	1	k__Bacteria:p__Proteobacteria:c__Alphaproteobacteria:o__Rhodospirillales:f__Rhodospirillaceae:g__
MID3	denovo1131	2	k__Bacteria:p__Actinobacteria:c__Actinobacteria:o__Actinomycetales:f__Sanguibacteraceae:g__Sanguibacter
MID3	denovo1132	2	k__Bacteria:p__Firmicutes:c__Bacilli:o__Lactobacillales:f__Enterococcaceae:g__Vagococcus
MID3	denovo1137	2	k__Bacteria:p__Proteobacteria:c__Alphaproteobacteria:o__BD7-3:f__:g__
MID3	denovo1139	1	k__Bacteria:p__Actinobacteria:c__Thermoleophilia:o__Solirubrobacterales:f__:g__
MID3	denovo114	4	k__Bacteria:p__Cyanobacteria:c__Chloroplast:o__Chlorophyta:f__:g__
MID3	denovo1141	1	k__Bacteria:p__Firmicutes:c__Bacilli:o__Lactobacillales
MID3	denovo1145	4	k__Bacteria:p__Proteobacteria:c__Alphaproteobacteria:o__Rhodospirillales:f__Rhodospirillaceae:g__
MID3	denovo1146	1	k__Bacteria:p__Proteobacteria:c__Gammaproteobacteria:o__Legionellales:f__Coxiellaceae:g__
MID3	denovo1147	1	k__Bacteria:p__Proteobacteria:c__Deltaproteobacteria:o__Myxococcales:f__:g__
MID3	denovo1148	3	k__Bacteria:p__Firmicutes:c__Clostridia:o__Clostridiales:f__Lachnospiraceae:g__
MID3	denovo1150	1	k__Bacteria:p__Firmicutes:c__Clostridia:o__Clostridiales:f__Lachnospiraceae:g__Roseburia
MID3	denovo1153	3	k__Bacteria:p__Cyanobacteria:c__Nostocophycideae:o__Stigonematales:f__Rivulariaceae:g__Calothrix
MID3	denovo1155	1	k__Bacteria:p__Firmicutes:c__Bacilli:o__Bacillales:f__Bacillaceae:g__Bacillus
MID3	denovo116	1	k__Bacteria:p__Firmicutes:c__Bacilli:o__Lactobacillales:f__Streptococcaceae:g__Streptococcus
MID3	denovo1160	1	k__Bacteria:p__Proteobacteria:c__Deltaproteobacteria:o__Spirobacillales:f__:g__
MID3	denovo1161	2	k__Bacteria:p__Firmicutes:c__Bacilli:o__Bacillales:f__Staphylococcaceae:g__Salinicoccus
MID3	denovo1165	7	k__Bacteria:p__Firmicutes:c__Clostridia:o__Clostridiales:f__[Tissierellaceae]:g__Anaerococcus
MID3	denovo1170	1	k__Bacteria:p__Actinobacteria:c__Actinobacteria:o__Actinomycetales:f__Dermacoccaceae:g__Dermacoccus
MID3	denovo1171	2	k__Bacteria:p__Proteobacteria:c__Gammaproteobacteria:o__Xanthomonadales:f__Xanthomonadaceae:g__Luteimonas
MID3	denovo1172	1	k__Bacteria:p__Firmicutes:c__Bacilli:o__Bacillales:f__Paenibacillaceae:g__Cohnella
MID3	denovo1173	6	k__Bacteria:p__Proteobacteria:c__Alphaproteobacteria:o__Rhodobacterales:f__Rhodobacteraceae:g__Paracoccus
MID3	denovo1184	7	k__Bacteria:p__Proteobacteria:c__Alphaproteobacteria:o__Rhizobiales:f__Phyllobacteriaceae:g__
MID3	denovo119	1	k__Bacteria:p__Proteobacteria:c__Alphaproteobacteria:o__Sphingomonadales:f__Sphingomonadaceae:g__Kaistobacter
MID3	denovo1191	1	k__Bacteria:p__Planctomycetes:c__Planctomycetia:o__Gemmatales:f__Isosphaeraceae:g__
MID3	denovo1199	1	k__Bacteria:p__Actinobacteria:c__Acidimicrobiia:o__Acidimicrobiales:f__C111:g__
MID3	denovo1200	1	k__Bacteria:p__Chloroflexi:c__Anaerolineae:o__WCHB1-50:f__:g__
MID3	denovo1201	2	k__Bacteria:p__Proteobacteria:c__Alphaproteobacteria:o__Rickettsiales:f__Rickettsiaceae:g__Rickettsia
MID3	denovo1202	29	k__Bacteria:p__Proteobacteria:c__Alphaproteobacteria:o__Rhizobiales:f__Methylobacteriaceae:g__Methylobacterium
MID3	denovo1206	1	k__Bacteria:p__Planctomycetes:c__Planctomycetia:o__Planctomycetales:f__Planctomycetaceae:g__Planctomyces
MID3	denovo1207	1	k__Bacteria:p__Proteobacteria:c__Betaproteobacteria:o__SBla14:f__:g__
MID3	denovo1210	2	k__Bacteria:p__Planctomycetes:c__vadinHA49:o__DH61:f__:g__
MID3	denovo1216	1	k__Bacteria:p__Actinobacteria:c__MB-A2-108:o__0319-7L14:f__:g__
MID3	denovo1217	1	k__Bacteria:p__Cyanobacteria:c__Chloroplast:o__Chlorophyta:f__:g__
MID3	denovo1223	1	k__Bacteria:p__Proteobacteria:c__Gammaproteobacteria:o__Enterobacteriales:f__Enterobacteriaceae:g__Dickeya
MID3	denovo1225	1	k__Bacteria:p__Proteobacteria:c__Alphaproteobacteria:o__Rhizobiales:f__Phyllobacteriaceae:g__Mesorhizobium
MID3	denovo1227	1	k__Bacteria:p__Proteobacteria:c__Alphaproteobacteria:o__Rhizobiales:f__Rhizobiaceae
MID3	denovo1230	1	k__Bacteria:p__Proteobacteria:c__Betaproteobacteria:o__Burkholderiales:f__Comamonadaceae:g__Comamonas
MID3	denovo1231	3	k__Bacteria:p__Proteobacteria:c__Alphaproteobacteria:o__Rhodobacterales:f__Rhodobacteraceae:g__Rubellimicrobium
MID3	denovo1233	1	k__Bacteria:p__Actinobacteria:c__Actinobacteria:o__Actinomycetales:f__Nocardioidaceae:g__Nocardioides
MID3	denovo1235	1	k__Bacteria:p__Firmicutes:c__Clostridia:o__Clostridiales:f__Clostridiaceae:g__Clostridium
MID3	denovo1236	3	k__Bacteria:p__Bacteroidetes:c__Cytophagia:o__Cytophagales:f__Cytophagaceae:g__Rhodocytophaga
MID3	denovo1237	1	k__Bacteria:p__Firmicutes:c__Clostridia:o__Clostridiales:f__Ruminococcaceae:g__
MID3	denovo1238	2	k__Bacteria:p__Proteobacteria:c__Gammaproteobacteria:o__Legionellales:f__Coxiellaceae:g__
MID3	denovo124	1	k__Bacteria:p__Firmicutes:c__Clostridia:o__Clostridiales:f__Christensenellaceae:g__
MID3	denovo1248	4	k__Bacteria:p__Proteobacteria:c__Alphaproteobacteria:o__Rhodospirillales:f__Rhodospirillaceae:g__
MID3	denovo1253	4	k__Bacteria:p__Proteobacteria:c__Alphaproteobacteria:o__Rhizobiales:f__Rhizobiaceae:g__Agrobacterium
MID3	denovo1255	1	k__Bacteria:p__Bacteroidetes:c__Bacteroidia:o__Bacteroidales:f__Prevotellaceae:g__Prevotella
MID3	denovo1257	7	k__Bacteria:p__Proteobacteria:c__Alphaproteobacteria:o__Sphingomonadales:f__Sphingomonadaceae
MID3	denovo126	3	k__Bacteria:p__Proteobacteria:c__Alphaproteobacteria:o__Rhizobiales:f__Hyphomicrobiaceae:g__Rhodoplanes
MID3	denovo1260	1	k__Bacteria:p__Planctomycetes:c__Planctomycetia:o__Gemmatales:f__Gemmataceae:g__Gemmata
MID3	denovo1265	2	k__Bacteria:p__Firmicutes:c__Bacilli:o__Lactobacillales:f__Lactobacillaceae:g__Lactobacillus
MID3	denovo1267	3	k__Bacteria:p__Proteobacteria:c__Alphaproteobacteria:o__Rickettsiales:f__:g__
MID3	denovo1269	1	k__Bacteria:p__Proteobacteria:c__Gammaproteobacteria:o__Thiotrichales:f__Piscirickettsiaceae:g__
MID3	denovo127	1	k__Bacteria:p__Proteobacteria:c__Gammaproteobacteria:o__Pseudomonadales:f__Pseudomonadaceae:g__
MID3	denovo1270	1	k__Bacteria:p__Proteobacteria:c__Alphaproteobacteria:o__Rhizobiales:f__Methylocystaceae:g__Methylopila
MID3	denovo1271	3	k__Bacteria:p__Cyanobacteria:c__4C0d-2:o__MLE1-12:f__:g__
MID3	denovo1276	1	k__Bacteria:p__Actinobacteria:c__Actinobacteria:o__Actinomycetales:f__Williamsiaceae:g__Williamsia
MID3	denovo1277	6	k__Bacteria:p__Firmicutes:c__Bacilli:o__Bacillales:f__Paenibacillaceae:g__Paenibacillus
MID3	denovo1279	4	k__Bacteria:p__Firmicutes:c__Bacilli:o__Lactobacillales
MID3	denovo1280	1	k__Bacteria:p__Proteobacteria:c__Gammaproteobacteria:o__Pseudomonadales:f__Pseudomonadaceae:g__Pseudomonas
MID3	denovo1287	2	k__Bacteria:p__Actinobacteria:c__Actinobacteria:o__Actinomycetales:f__Corynebacteriaceae:g__Corynebacterium
MID3	denovo1288	3	k__Bacteria:p__Firmicutes:c__Clostridia:o__Clostridiales:f__Lachnospiraceae:g__
MID3	denovo1290	4	k__Bacteria:p__Actinobacteria:c__Actinobacteria:o__Actinomycetales:f__Dermabacteraceae:g__Brachybacterium
MID3	denovo1292	3	k__Bacteria:p__Proteobacteria:c__Alphaproteobacteria:o__Rhodospirillales:f__Acetobacteraceae:g__
MID3	denovo1293	2	k__Bacteria:p__Firmicutes:c__Clostridia:o__Clostridiales:f__:g__

MID3	denovo13	4	k__Bacteria;p__Actinobacteria;c__Actinobacteria;o__Actinomycetales;f__Corynebacteriaceae;g__Corynebacterium
MID3	denovo130	1	k__Bacteria;p__Planctomycetes;c__Planctomycetia;o__Gemmatales;f__Gemmataceae;g__
MID3	denovo1300	13	k__Bacteria;p__Actinobacteria;c__Actinobacteria;o__Actinomycetales;f__Nocardioidaceae;g__Friedmanniella
MID3	denovo1301	2	k__Bacteria;p__Actinobacteria;c__Coriobacteriia;o__Coriobacteriales;f__Coriobacteriaceae;g__Collinsella
MID3	denovo1303	1	k__Bacteria;p__Actinobacteria;c__Actinobacteria;o__Actinomycetales;f__Propionibacteriaceae;g__
MID3	denovo1308	12	k__Bacteria;p__Firmicutes;c__Bacilli;o__Lactobacillales;f__Streptococcaceae;g__Streptococcus
MID3	denovo1309	1	k__Bacteria;p__Proteobacteria;c__Alphaproteobacteria;o__Rhodospirillales;f__Rhodospirillaceae;g__
MID3	denovo1312	1	k__Bacteria;p__Proteobacteria;c__Betaproteobacteria;o__Burkholderiales;f__Oxalobacteraceae;g__
MID3	denovo1317	2	k__Bacteria;p__Firmicutes;c__Bacilli;o__Lactobacillales;f__Aerococcaceae;g__Alloiococcus
MID3	denovo1318	3	k__Bacteria;p__Cyanobacteria;c__Chloroplast;o__Chlorophyta;f__Trebouxiophyceae;g__Coccomyxa
MID3	denovo1319	1	k__Bacteria;p__Proteobacteria;c__Alphaproteobacteria;o__Sphingomonadales;f__Sphingomonadaceae;g__Kaistobacter
MID3	denovo1322	1	k__Bacteria;p__Proteobacteria;c__Alphaproteobacteria;o__Rhodospirillales;f__Rhodospirillaceae;g__
MID3	denovo1323	7	k__Bacteria;p__Proteobacteria;c__Alphaproteobacteria;o__Rhizobiales;f__Methylocystaceae;g__
MID3	denovo1324	4	k__Bacteria;p__Proteobacteria;c__Alphaproteobacteria;o__Rhodospirillales;f__Acetobacteraceae;g__
MID3	denovo1328	3	k__Bacteria;p__Firmicutes;c__Clostridia;o__Clostridiales;f__Lachnospiraceae;g__Coprococcus
MID3	denovo1329	1	k__Bacteria;p__Acidobacteria;c__[Chloracidobacteria];o__RB41;f__Ellin6075;g__
MID3	denovo133	3	k__Bacteria;p__Bacteroidetes;c__Bacteroidia;o__Bacteroidales;f__Porphyromonadaceae;g__Candidatus_Azobacteroides
MID3	denovo1334	55	k__Bacteria;p__Firmicutes;c__Bacilli;o__Lactobacillales;f__Streptococcaceae;g__Streptococcus
MID3	denovo1335	3	k__Bacteria;p__Proteobacteria;c__Gammaproteobacteria;o__Legionellales;f__Legionellaceae;g__
MID3	denovo1336	1	k__Bacteria;p__Actinobacteria;c__Acidimicrobiia;o__Acidimicrobiales;f__C111;g__
MID3	denovo134	47	k__Bacteria;p__Firmicutes;c__Bacilli;o__Lactobacillales;f__;g__
MID3	denovo1341	13	k__Bacteria;p__Firmicutes;c__Clostridia;o__Clostridiales;f__[Tissierellaceae];g__Anaerococcus
MID3	denovo1342	1	k__Bacteria;p__Actinobacteria;c__Actinobacteria;o__Actinomycetales;f__Intrasporangiaceae
MID3	denovo1345	3	k__Bacteria;p__Firmicutes;c__Bacilli;o__Bacillales;f__Alicyclobacillaceae;g__Alicyclobacillus
MID3	denovo1346	11	k__Bacteria;p__Firmicutes;c__Bacilli;o__Lactobacillales;f__Streptococcaceae;g__Lactococcus
MID3	denovo1347	2	k__Bacteria;p__Proteobacteria;c__Gammaproteobacteria;o__Xanthomonadales;f__Xanthomonadaceae;g__Luteibacter
MID3	denovo1349	9	k__Bacteria;p__Proteobacteria;c__Deltaproteobacteria;o__Myxococcales;f__Nannocystaceae;g__Nannocystis
MID3	denovo135	1	k__Bacteria;p__Firmicutes;c__Clostridia;o__Clostridiales;f__Clostridiaceae;g__Clostridium
MID3	denovo1357	2	k__Bacteria;p__Proteobacteria;c__Alphaproteobacteria;o__Rhodospirillales;f__Rhodospirillaceae;g__
MID3	denovo138	13	k__Bacteria;p__Proteobacteria;c__Alphaproteobacteria;o__Rhodospirillales;f__Rhodospirillaceae;g__Skermanella
MID3	denovo140	1	k__Bacteria;p__Proteobacteria;c__Alphaproteobacteria;o__Caulobacterales;f__Caulobacteraceae;g__
MID3	denovo148	1	k__Bacteria;p__Cyanobacteria;c__Synechococcophycideae;o__Pseudanabaenales;f__Pseudanabaenaceae;g__Leptolyngbya
MID3	denovo149	1	k__Bacteria;p__Actinobacteria;c__Actinobacteria;o__Actinomycetales;f__Cellulomonadaceae;g__Cellulomonas
MID3	denovo15	2	k__Bacteria;p__[Thermi];c__Deinococci;o__Deinococcales;f__Trueperaceae;g__Truepera
MID3	denovo152	1	k__Bacteria;p__Actinobacteria;c__Thermoleophilia;o__Solirubrobacterales;f__;g__
MID3	denovo153	1	k__Bacteria;p__Chlorobi;c__SJA-28;o__;f__;g__
MID3	denovo155	1	k__Bacteria;p__Proteobacteria;c__Alphaproteobacteria;o__Rhodobacterales;f__Rhodobacteraceae;g__Rubellimicrobium
MID3	denovo157	1	k__Bacteria;p__Proteobacteria;c__Alphaproteobacteria;o__Rhodospirillales;f__;g__
MID3	denovo160	1	k__Bacteria;p__Gemmatimonadetes;c__Gemm-3;o__;f__;g__
MID3	denovo161	2	k__Bacteria;p__Bacteroidetes;c__Bacteroidia;o__Bacteroidales;f__Porphyromonadaceae;g__Candidatus_Azobacteroides
MID3	denovo162	2	k__Bacteria;p__Bacteroidetes;c__Bacteroidia;o__Bacteroidales;f__Bacteroidaceae;g__Bacteroides
MID3	denovo164	1	k__Bacteria;p__Proteobacteria;c__Gammaproteobacteria;o__Pseudomonadales;f__Moraxellaceae;g__Psychrobacter
MID3	denovo168	2	k__Bacteria;p__Firmicutes;c__Bacilli;o__Lactobacillales;f__Streptococcaceae;g__Streptococcus
MID3	denovo169	1	k__Bacteria;p__Actinobacteria;c__Acidimicrobiia;o__Acidimicrobiales;f__;g__
MID3	denovo17	1	k__Bacteria;p__Firmicutes;c__Clostridia;o__Clostridiales;f__Veillonellaceae
MID3	denovo170	2	k__Bacteria;p__Firmicutes;c__Clostridia;o__Clostridiales;f__[Tissierellaceae];g__Anaerococcus
MID3	denovo176	2	k__Bacteria;p__Proteobacteria;c__Alphaproteobacteria;o__Sphingomonadales;f__Sphingomonadaceae;g__
MID3	denovo178	4	k__Bacteria;p__Bacteroidetes;c__[Rhodothermi];o__[Rhodothermales];f__Rhodothermaceae;g__Rubricoccus
MID3	denovo179	1	k__Bacteria;p__Proteobacteria;c__Alphaproteobacteria;o__Sphingomonadales;f__Sphingomonadaceae;g__Sphingomonas
MID3	denovo18	1	k__Bacteria;p__Proteobacteria;c__Gammaproteobacteria;o__Pseudomonadales;f__Pseudomonadaceae;g__Pseudomonas
MID3	denovo180	1	k__Bacteria;p__Proteobacteria;c__Alphaproteobacteria;o__Caulobacterales;f__Caulobacteraceae;g__Phenylobacterium
MID3	denovo182	1	k__Bacteria;p__Proteobacteria;c__Alphaproteobacteria;o__Rhodospirillales;f__Acetobacteraceae;g__
MID3	denovo183	67	k__Bacteria;p__Cyanobacteria;c__Oscillatoriophycideae;o__Chroococcales;f__Xenococcaceae
MID3	denovo184	24	k__Bacteria;p__Firmicutes;c__Clostridia;o__Clostridiales;f__[Tissierellaceae];g__Peptoniphilus
MID3	denovo185	13	k__Bacteria;p__Proteobacteria;c__Gammaproteobacteria;o__Xanthomonadales;f__Xanthomonadaceae;g__
MID3	denovo186	2	k__Bacteria;p__Proteobacteria;c__Alphaproteobacteria;o__Rhizobiales;f__Bradyrhizobiaceae;g__Balneimonas
MID3	denovo187	1	k__Bacteria;p__Firmicutes;c__Clostridia;o__Clostridiales;f__Clostridiaceae;g__
MID3	denovo189	1	k__Bacteria;p__Actinobacteria;c__Actinobacteria;o__Actinomycetales;f__;g__
MID3	denovo192	1	k__Bacteria;p__Proteobacteria;c__Alphaproteobacteria;o__Rhodospirillales;f__Rhodospirillaceae;g__Azospirillum
MID3	denovo193	3	k__Bacteria;p__Proteobacteria;c__Alphaproteobacteria;o__Caulobacterales;f__Caulobacteraceae;g__Phenylobacterium
MID3	denovo195	2	k__Bacteria;p__Firmicutes;c__Bacilli;o__Bacillales;f__Paenibacillaceae;g__Saccharibacillus
MID3	denovo2	1	k__Bacteria;p__Proteobacteria;c__Betaproteobacteria;o__Burkholderiales;f__Oxalobacteraceae;g__
MID3	denovo201	2	k__Bacteria;p__Firmicutes;c__Clostridia;o__Clostridiales;f__Peptostreptococcaceae;g__Peptostreptococcus
MID3	denovo207	2	k__Bacteria;p__Actinobacteria;c__Acidimicrobiia;o__Acidimicrobiales;f__C111;g__
MID3	denovo208	1	Unassigned
MID3	denovo210	3	k__Bacteria;p__Proteobacteria;c__Gammaproteobacteria;o__Xanthomonadales;f__Xanthomonadaceae;g__
MID3	denovo213	2	k__Bacteria;p__Proteobacteria;c__Gammaproteobacteria;o__Xanthomonadales;f__Xanthomonadaceae;g__Luteimonas

MID3	denovo214	1	k__Bacteria:p__Proteobacteria:c__Alphaproteobacteria:o__Rhizobiales:f__Methylobacteriaceae:g__
MID3	denovo218	2	k__Bacteria:p__Cyanobacteria:c__Oscillatoriophycideae:o__Oscillatoriales:f__Phormidiaceae:g__Phormidium
MID3	denovo221	3	k__Bacteria:p__Proteobacteria:c__Alphaproteobacteria:o__:f__:g__
MID3	denovo225	3	k__Bacteria:p__Proteobacteria:c__Gammaproteobacteria:o__Alteromonadales:f__211ds20:g__
MID3	denovo228	2	k__Bacteria:p__Proteobacteria:c__Alphaproteobacteria:o__Sphingomonadales:f__Sphingomonadaceae:g__Sphingomonas
MID3	denovo229	6	k__Bacteria:p__Proteobacteria:c__Alphaproteobacteria:o__Rhodospirillales:f__Acetobacteraceae:g__
MID3	denovo231	1	k__Bacteria:p__Proteobacteria:c__Deltaproteobacteria:o__Myxococcales:f__:g__
MID3	denovo232	16	k__Bacteria:p__Firmicutes:c__Clostridia:o__Clostridiales:f__[Tissierellaceae]:g__Anaerococcus
MID3	denovo234	2	k__Bacteria:p__Proteobacteria:c__Alphaproteobacteria:o__Rhizobiales:f__Bradyrhizobiaceae:g__
MID3	denovo236	2	k__Bacteria:p__Actinobacteria:c__Actinobacteria:o__Actinomycetales:f__Geodermatophilaceae:g__
MID3	denovo24	1	k__Bacteria:p__Firmicutes:c__Clostridia:o__Clostridiales:f__[Tissierellaceae]:g__Anaerococcus
MID3	denovo248	2	k__Bacteria:p__Proteobacteria:c__Alphaproteobacteria:o__Rhodobacterales:f__Rhodobacteraceae:g__Rubellimicrobium
MID3	denovo254	2	k__Bacteria:p__Firmicutes:c__Clostridia:o__MBA08:f__:g__
MID3	denovo255	3	k__Bacteria:p__Proteobacteria:c__Alphaproteobacteria:o__Rhizobiales:f__Beijerinckiaceae:g__
MID3	denovo264	1	k__Bacteria:p__Proteobacteria:c__Alphaproteobacteria:o__Rhodospirillales:f__Acetobacteraceae:g__
MID3	denovo27	1	k__Bacteria:p__Proteobacteria:c__Deltaproteobacteria:o__Myxococcales:f__:g__
MID3	denovo270	1	k__Bacteria:p__Proteobacteria:c__Alphaproteobacteria:o__Rhizobiales:f__Methylobacteriaceae:g__
MID3	denovo272	2	k__Bacteria:p__Proteobacteria:c__Alphaproteobacteria:o__Rhodobacterales:f__Rhodobacteraceae:g__Rubellimicrobium
MID3	denovo273	2	k__Bacteria:p__Planctomycetes:c__Planctomycetia:o__Planctomycetales:f__Planctomycetaceae:g__Planctomyces
MID3	denovo275	1	k__Bacteria:p__Proteobacteria:c__Alphaproteobacteria:o__Rhizobiales:f__:g__
MID3	denovo277	1	Unassigned
MID3	denovo285	1	k__Bacteria:p__Actinobacteria:c__Actinobacteria:o__Actinomycetales:f__Nocardioidaceae:g__
MID3	denovo286	2	k__Bacteria:p__Actinobacteria:c__Actinobacteria:o__Actinomycetales:f__Frankiaceae:g__
MID3	denovo288	3	k__Bacteria:p__Proteobacteria:c__Alphaproteobacteria:o__Caulobacterales:f__Caulobacteraceae:g__
MID3	denovo290	2	k__Bacteria:p__Proteobacteria:c__Alphaproteobacteria:o__Sphingomonadales:f__Sphingomonadaceae:g__
MID3	denovo291	1	k__Bacteria:p__Proteobacteria:c__Betaproteobacteria:o__Burkholderiales:f__Comamonadaceae:g__Delftia
MID3	denovo292	1	k__Bacteria:p__Firmicutes:c__Clostridia:o__Clostridiales:f__Ruminococcaceae:g__
MID3	denovo295	2	k__Bacteria:p__Proteobacteria:c__Alphaproteobacteria:o__Rhizobiales:f__Rhizobiaceae:g__Kaistia
MID3	denovo296	3	k__Bacteria:p__Firmicutes:c__Clostridia:o__Clostridiales:f__[Tissierellaceae]:g__WAL_1855D
MID3	denovo298	1	k__Bacteria:p__Cyanobacteria:c__Oscillatoriophycideae:o__Oscillatoriales:f__Phormidiaceae:g__Phormidium
MID3	denovo299	1	k__Bacteria:p__Firmicutes:c__Clostridia:o__:f__:g__
MID3	denovo303	1	k__Bacteria:p__Bacteroidetes:c__Bacteroidia:o__Bacteroidales:f__[Paraprevotellaceae]:g__[Prevotella]
MID3	denovo305	1	k__Bacteria:p__Proteobacteria:c__Alphaproteobacteria:o__Rhizobiales:f__Methylocystaceae:g__Methylosinus
MID3	denovo31	1	k__Bacteria:p__Proteobacteria:c__Alphaproteobacteria:o__Rhodospirillales:f__Rhodospirillaceae:g__Magnetospirillum
MID3	denovo313	2	k__Bacteria:p__Proteobacteria:c__Gammaproteobacteria:o__Vibrionales:f__Pseudoalteromonadaceae:g__Pseudoalteromonas
MID3	denovo314	4	k__Bacteria:p__Proteobacteria:c__Alphaproteobacteria:o__Rhizobiales:f__Beijerinckiaceae:g__
MID3	denovo315	7	k__Bacteria:p__Proteobacteria:c__Alphaproteobacteria:o__Rhizobiales:f__Rhizobiaceae:g__Rhizobium
MID3	denovo319	1	k__Bacteria:p__Bacteroidetes:c__Flavobacteriia:o__Flavobacteriales:f__[Weeksellaceae]:g__Chryseobacterium
MID3	denovo323	2	k__Bacteria:p__Proteobacteria:c__Gammaproteobacteria:o__Pseudomonadales:f__Pseudomonadaceae:g__Pseudomonas
MID3	denovo324	3	k__Bacteria:p__Proteobacteria:c__Alphaproteobacteria:o__Rhizobiales:f__Hyphomicrobiaceae:g__Devosia
MID3	denovo325	1	k__Bacteria:p__Firmicutes:c__Clostridia:o__Clostridiales:f__[Tissierellaceae]:g__Anaerococcus
MID3	denovo327	1	k__Bacteria:p__Actinobacteria:c__Actinobacteria:o__Actinomycetales:f__Pseudonocardiaceae:g__Pseudonocardia
MID3	denovo331	2	k__Bacteria:p__Cyanobacteria:c__ML635J-21:o__:f__:g__
MID3	denovo332	1	k__Bacteria:p__Chloroflexi:c__Anaerolineae:o__SBR1031:f__A4b:g__
MID3	denovo333	2	k__Bacteria:p__Firmicutes:c__Bacilli:o__Bacillales:f__Bacillaceae:g__Bacillus
MID3	denovo334	1	k__Bacteria:p__Proteobacteria:c__Alphaproteobacteria:o__Rhodobacterales:f__Rhodobacteraceae
MID3	denovo335	2	k__Bacteria:p__Actinobacteria:c__Actinobacteria:o__Actinomycetales:f__Micrococcaceae:g__Arthrobacter
MID3	denovo337	4	k__Bacteria:p__Proteobacteria:c__Alphaproteobacteria:o__Rhizobiales:f__Aurantimonadaceae:g__
MID3	denovo34	1	k__Bacteria:p__Proteobacteria:c__Gammaproteobacteria:o__Xanthomonadales:f__Xanthomonadaceae:g__Dokdonella
MID3	denovo340	3	k__Bacteria:p__Cyanobacteria:c__Chloroplast:o__Chlorophyta:f__:g__
MID3	denovo348	1	k__Bacteria:p__Proteobacteria:c__Alphaproteobacteria:o__Rhodospirillales:f__Rhodospirillaceae:g__
MID3	denovo349	2	k__Bacteria:p__Proteobacteria:c__Alphaproteobacteria:o__Sphingomonadales:f__Erythrobacteraceae:g__
MID3	denovo35	12	k__Bacteria:p__Proteobacteria:c__Deltaproteobacteria:o__Myxococcales:f__Haliangiaceae:g__
MID3	denovo353	9	k__Bacteria:p__Proteobacteria:c__Alphaproteobacteria:o__Rhodobacterales:f__Rhodobacteraceae:g__Rubellimicrobium
MID3	denovo357	2	k__Bacteria:p__Actinobacteria:c__Actinobacteria:o__Actinomycetales:f__Corynebacteriaceae:g__Corynebacterium
MID3	denovo359	5	k__Bacteria:p__Bacteroidetes:c__Sphingobacteriia:o__Sphingobacteriales:f__:g__
MID3	denovo360	3	k__Bacteria:p__Bacteroidetes:c__Cytophagia:o__Cytophagales:f__Cytophagaceae:g__Emticicia
MID3	denovo361	1	k__Bacteria:p__Firmicutes:c__Bacilli:o__Lactobacillales:f__Lactobacillaceae:g__Lactobacillus
MID3	denovo362	1	k__Bacteria:p__Proteobacteria:c__Alphaproteobacteria:o__:f__:g__
MID3	denovo367	1	k__Bacteria:p__Firmicutes:c__Clostridia:o__Clostridiales:f__:g__
MID3	denovo37	1	k__Bacteria:p__Cyanobacteria:c__Nostocophycideae:o__Nostocales:f__Nostocaceae:g__
MID3	denovo377	3	k__Bacteria:p__Actinobacteria:c__Actinobacteria:o__Actinomycetales:f__Nocardioidaceae:g__Propionicimonas
MID3	denovo378	2	k__Bacteria:p__Proteobacteria:c__Gammaproteobacteria:o__Enterobacteriales:f__Enterobacteriaceae:g__Erwinia
MID3	denovo379	1	k__Bacteria:p__Proteobacteria:c__Alphaproteobacteria:o__Rickettsiales:f__Rickettsiaceae:g__Wolbachia
MID3	denovo38	9	k__Bacteria:p__Firmicutes:c__Clostridia:o__Clostridiales:f__Ruminococcaceae:g__
MID3	denovo380	1	k__Bacteria:p__Actinobacteria:c__Thermoleophilia:o__Gaiellales:f__Gaiellaceae:g__
MID3	denovo384	1	k__Bacteria:p__Fusobacteria:c__Fusobacteriia:o__Fusobacteriales:f__Fusobacteriaceae:g__Fusobacterium

MID3	denovo387	1	k__Bacteria:p__Proteobacteria:c__Gammaproteobacteria:o__Aeromonadales:f__Aeromonadaceae:g__
MID3	denovo389	1	k__Bacteria:p__Proteobacteria:c__Alphaproteobacteria:o__Rhizobiales:f__Methylocystaceae:g__
MID3	denovo390	1	k__Bacteria:p__Actinobacteria:c__Actinobacteria:o__Actinomycetales:f__Microbacteriaceae:g__Frigoribacterium
MID3	denovo393	5	k__Bacteria:p__Proteobacteria:c__Gammaproteobacteria:o__Xanthomonadales:f__Xanthomonadaceae:g__Lysobacter
MID3	denovo398	1	k__Bacteria:p__Bacteroidetes:c__Cytophagia:o__Cytophagales:f__Cytophagaceae:g__Rhodocytophaga
MID3	denovo400	1	k__Bacteria:p__Proteobacteria:c__Alphaproteobacteria:o__Rhizobiales:f__Methylobacteriaceae:g__Methylobacterium
MID3	denovo404	4	k__Bacteria:p__Firmicutes:c__Bacilli:o__Lactobacillales:f__Enterococcaceae:g__Enterococcus
MID3	denovo407	4	k__Bacteria:p__Proteobacteria:c__Betaproteobacteria:o__:f__:g__
MID3	denovo410	4	k__Bacteria:p__Bacteroidetes:c__Sphingobacteriia:o__Sphingobacteriales:f__:g__
MID3	denovo414	1	k__Bacteria:p__Proteobacteria:c__Alphaproteobacteria:o__Caulobacterales:f__Caulobacteraceae:g__
MID3	denovo416	2	k__Bacteria:p__Bacteroidetes:c__Bacteroidia:o__Bacteroidales:f__Porphyromonadaceae:g__Candidatus_Azobacteroides
MID3	denovo42	1	k__Bacteria:p__Planctomycetes:c__Planctomycetia:o__Gemmatales:f__Isosphaeraceae:g__
MID3	denovo420	1	k__Bacteria:p__Proteobacteria:c__Deltaproteobacteria:o__Myxococcales:f__:g__
MID3	denovo421	5	k__Bacteria:p__Bacteroidetes:c__Cytophagia:o__Cytophagales:f__Cytophagaceae:g__
MID3	denovo431	9	k__Bacteria:p__Proteobacteria:c__Alphaproteobacteria:o__Rhizobiales:f__Methylobacteriaceae:g__Methylobacterium
MID3	denovo433	1	k__Bacteria:p__Proteobacteria:c__Alphaproteobacteria:o__Sphingomonadales:f__Sphingomonadaceae:g__
MID3	denovo434	1	k__Bacteria:p__Actinobacteria:c__Actinobacteria:o__Actinomycetales:f__Microbacteriaceae:g__Microbacterium
MID3	denovo436	1	k__Bacteria:p__Firmicutes:c__Bacilli:o__Lactobacillales:f__Streptococcaceae:g__Streptococcus
MID3	denovo437	3	k__Bacteria:p__Bacteroidetes:c__Bacteroidia:o__Bacteroidales:f__Prevotellaceae:g__Prevotella
MID3	denovo438	1	k__Bacteria:p__Firmicutes:c__Clostridia:o__Clostridiales:f__Veillonellaceae:g__Veillonella
MID3	denovo443	1	k__Bacteria:p__Proteobacteria:c__Alphaproteobacteria:o__Rhizobiales:f__Bradyrhizobiaceae:g__
MID3	denovo445	2	k__Bacteria:p__Proteobacteria:c__Alphaproteobacteria:o__Rhizobiales:f__Bradyrhizobiaceae:g__
MID3	denovo446	3	k__Bacteria:p__Proteobacteria:c__Alphaproteobacteria:o__Rhodospirillales:f__Rhodospirillaceae:g__
MID3	denovo449	1	k__Bacteria:p__Proteobacteria:c__Alphaproteobacteria:o__Rhizobiales:f__Phyllobacteriaceae:g__Aminobacter
MID3	denovo45	1	k__Bacteria:p__Actinobacteria:c__Actinobacteria:o__Actinomycetales:f__Kineosporiaceae:g__
MID3	denovo450	5	k__Bacteria:p__Planctomycetes:c__Planctomycetia:o__Planctomycetales:f__Planctomycetaceae:g__Planctomyces
MID3	denovo454	1	k__Bacteria:p__Proteobacteria:c__Alphaproteobacteria:o__Sphingomonadales:f__Sphingomonadaceae:g__Kaistobacter
MID3	denovo457	1	k__Bacteria:p__Actinobacteria:c__Thermoleophilia:o__Solirubrobacterales:f__:g__
MID3	denovo458	1	k__Bacteria:p__Actinobacteria:c__Actinobacteria:o__Actinomycetales:f__:g__
MID3	denovo463	2	k__Bacteria:p__Proteobacteria:c__Alphaproteobacteria:o__Rhodospirillales:f__Acetobacteraceae:g__Roseococcus
MID3	denovo471	3	k__Bacteria:p__Proteobacteria:c__Alphaproteobacteria:o__Rhizobiales:f__Hyphomicrobiaceae:g__Devosia
MID3	denovo475	1	k__Bacteria:p__Firmicutes:c__Clostridia:o__:f__:g__
MID3	denovo48	1	k__Bacteria:p__Cyanobacteria:c__4C0d-2:o__SM1D11:f__:g__
MID3	denovo483	1	k__Bacteria:p__Proteobacteria:c__Alphaproteobacteria:o__Rhodospirillales:f__:g__
MID3	denovo485	1	k__Bacteria:p__Firmicutes:c__Clostridia:o__Clostridiales:f__Lachnospiraceae:g__Blautia
MID3	denovo488	7	k__Bacteria:p__Proteobacteria:c__Gammaproteobacteria:o__Pseudomonadales:f__Moraxellaceae:g__Moraxella
MID3	denovo489	1	k__Bacteria:p__Proteobacteria:c__Gammaproteobacteria:o__Pseudomonadales:f__Pseudomonadaceae:g__
MID3	denovo490	1	k__Bacteria:p__Proteobacteria:c__Alphaproteobacteria:o__Rhodospirillales:f__Rhodospirillaceae:g__
MID3	denovo492	40	k__Bacteria:p__Cyanobacteria:c__Oscillatoriophycideae:o__Chroococcales:f__Xenococcaceae:g__Chroococcidiopsis
MID3	denovo493	1	k__Bacteria:p__Firmicutes:c__Clostridia:o__Clostridiales:f__Clostridiaceae:g__Clostridium
MID3	denovo494	1	k__Bacteria:p__Firmicutes:c__Clostridia:o__Clostridiales:f__Lachnospiraceae:g__Coprococcus
MID3	denovo498	2	k__Bacteria:p__Proteobacteria:c__Betaproteobacteria:o__Burkholderiales:f__:g__
MID3	denovo501	1	k__Bacteria:p__Actinobacteria:c__Actinobacteria:o__Actinomycetales:f__Nocardioidaceae:g__Propionicimonas
MID3	denovo502	3	k__Bacteria:p__Firmicutes:c__Bacilli:o__Lactobacillales:f__Leuconostocaceae:g__Leuconostoc
MID3	denovo508	1	k__Bacteria:p__Bacteroidetes:c__Cytophagia:o__Cytophagales:f__Cytophagaceae:g__Spirosoma
MID3	denovo511	5	k__Bacteria:p__Bacteroidetes:c__Flavobacteriia:o__Flavobacteriales:f__[Weeksellaceae]:g__Chryseobacterium
MID3	denovo515	3	k__Bacteria:p__Actinobacteria:c__Thermoleophilia:o__Gaiellales:f__Gaiellaceae:g__
MID3	denovo518	1	k__Bacteria:p__Proteobacteria:c__Alphaproteobacteria:o__Rhizobiales:f__:g__
MID3	denovo519	1	k__Bacteria:p__Proteobacteria:c__Betaproteobacteria:o__Ellin6067:f__:g__
MID3	denovo521	1	k__Bacteria:p__Proteobacteria:c__Betaproteobacteria:o__:f__:g__
MID3	denovo528	8	k__Bacteria:p__Bacteroidetes:c__Cytophagia:o__Cytophagales:f__Cytophagaceae:g__
MID3	denovo530	15	k__Bacteria:p__Firmicutes:c__Bacilli:o__Lactobacillales:f__Lactobacillaceae:g__Lactobacillus
MID3	denovo532	2	k__Bacteria:p__Firmicutes:c__Clostridia:o__Clostridiales:f__Veillonellaceae:g__Sporomusa
MID3	denovo534	2	k__Bacteria:p__Proteobacteria:c__Betaproteobacteria:o__Burkholderiales:f__Oxalobacteraceae:g__Janthinobacterium
MID3	denovo538	2	k__Bacteria:p__Proteobacteria:c__Betaproteobacteria:o__SC-I-84:f__:g__
MID3	denovo540	3	k__Bacteria:p__Firmicutes:c__Bacilli:o__Bacillales:f__Bacillaceae:g__Bacillus
MID3	denovo541	1	k__Bacteria:p__Bacteroidetes:c__Bacteroidia:o__Bacteroidales:f__Bacteroidaceae:g__Bacteroides
MID3	denovo542	49	k__Bacteria:p__Firmicutes:c__Clostridia:o__Clostridiales:f__[Tissierellaceae]:g__Finegoldia
MID3	denovo544	1	k__Bacteria:p__Firmicutes:c__Clostridia:o__Clostridiales:f__Ruminococcaceae:g__
MID3	denovo545	1	k__Bacteria:p__Acidobacteria:c__Solibacteres:o__Solibacterales:f__:g__
MID3	denovo548	1	k__Bacteria:p__Firmicutes:c__Clostridia:o__Clostridiales:f__Lachnospiraceae:g__
MID3	denovo555	1	k__Bacteria:p__Cyanobacteria:c__Oscillatoriophycideae:o__Chroococcales:f__Xenococcaceae:g__
MID3	denovo557	1	k__Bacteria:p__Proteobacteria:c__Alphaproteobacteria:o__Rhizobiales:f__Hyphomicrobiaceae:g__
MID3	denovo56	1	k__Bacteria:p__Proteobacteria:c__Alphaproteobacteria:o__Rhodobacterales:f__Rhodobacteraceae:g__Rubellimicrobium
MID3	denovo564	2	k__Bacteria:p__Firmicutes:c__Clostridia:o__Clostridiales:f__Christensenellaceae:g__
MID3	denovo568	2	k__Bacteria:p__Proteobacteria:c__Alphaproteobacteria:o__Sphingomonadales:f__Sphingomonadaceae:g__Sphingobium
MID3	denovo575	1	k__Bacteria:p__Actinobacteria:c__Thermoleophilia:o__Gaiellales:f__:g__

MID3	denovo577	1	k__Bacteria:p__Firmicutes:c__Clostridia:o__Clostridiales:f__Veillonellaceae:g__Veillonella
MID3	denovo578	1	k__Bacteria:p__Actinobacteria:c__Actinobacteria:o__Actinomycetales:f__Microbacteriaceae:g__Mycetocola
MID3	denovo584	1	k__Bacteria:p__Actinobacteria:c__Acidimicrobiia:o__Acidimicrobiales:f__Microthrixaceae:g__
MID3	denovo588	1	k__Bacteria:p__Firmicutes:c__Clostridia:o__OPB54:f__:g__
MID3	denovo591	1	k__Bacteria:p__Actinobacteria:c__MB-A2-108:o__0319-7L14:f__:g__
MID3	denovo592	3	k__Bacteria:p__Firmicutes:c__Clostridia:o__Clostridiales:f__Clostridiaceae:g__
MID3	denovo593	1	k__Bacteria:p__Actinobacteria:c__Actinobacteria:o__Actinomycetales:f__:g__
MID3	denovo594	1	k__Bacteria:p__Nitrospirae:c__Nitrospira:o__Nitrospirales:f__Nitrospiraceae:g__Nitrospira
MID3	denovo595	1	k__Bacteria:p__Proteobacteria:c__Alphaproteobacteria:o__Rhodobacterales:f__Rhodobacteraceae:g__Amaricoccus
MID3	denovo599	1	k__Bacteria:p__Firmicutes:c__Clostridia:o__Clostridiales:f__Lachnospiraceae:g__Dorea
MID3	denovo60	5	k__Bacteria:p__Proteobacteria:c__Alphaproteobacteria:o__Sphingomonadales:f__Sphingomonadaceae:g__
MID3	denovo600	4	k__Bacteria:p__Cyanobacteria:c__Oscillatoriophycideae:o__Oscillatoriales:f__Phormidiaceae:g__Phormidium
MID3	denovo601	1	k__Bacteria:p__Actinobacteria:c__Actinobacteria:o__Actinomycetales:f__Geodermatophilaceae:g__Modestobacter
MID3	denovo602	2	k__Bacteria:p__Firmicutes:c__Clostridia:o__Clostridiales:f__Lachnospiraceae:g__Catonella
MID3	denovo605	6	k__Bacteria:p__Firmicutes:c__Bacilli:o__Gemellales:f__Gemellaceae:g__Gemella
MID3	denovo606	1	k__Bacteria:p__Planctomycetes:c__Planctomycetia:o__Gemmatales:f__Gemmataceae:g__
MID3	denovo612	1	k__Bacteria:p__Proteobacteria:c__Alphaproteobacteria:o__Rhizobiales
MID3	denovo616	1	k__Bacteria:p__Proteobacteria:c__Alphaproteobacteria:o__Rhizobiales:f__Phyllobacteriaceae
MID3	denovo618	1	k__Bacteria:p__Proteobacteria:c__Alphaproteobacteria:o__Rhizobiales:f__Hyphomicrobiaceae:g__Rhodoplanes
MID3	denovo622	2	k__Bacteria:p__Firmicutes:c__Clostridia:o__Clostridiales:f__Lachnospiraceae:g__Coprococcus
MID3	denovo624	2	k__Bacteria:p__Proteobacteria:c__Gammaproteobacteria:o__Xanthomonadales:f__Xanthomonadaceae:g__
MID3	denovo628	7	k__Bacteria:p__Proteobacteria:c__Gammaproteobacteria:o__Xanthomonadales:f__Xanthomonadaceae:g__Arenimonas
MID3	denovo63	8	k__Bacteria:p__Firmicutes:c__Bacilli:o__Lactobacillales:f__Lactobacillaceae:g__Lactobacillus
MID3	denovo630	1	k__Bacteria:p__Bacteroidetes:c__Flavobacteriia:o__Flavobacteriales:f__[Weeksellaceae]:g__Chryseobacterium
MID3	denovo634	1	k__Bacteria:p__Cyanobacteria:c__Chloroplast:o__Chlorophyta:f__Chlamydomonadaceae:g__
MID3	denovo636	2	k__Bacteria:p__Actinobacteria:c__Thermoleophilia:o__Solirubrobacterales:f__Patulibacteraceae:g__
MID3	denovo638	2	k__Bacteria:p__Cyanobacteria:c__Chloroplast:o__Stramenopiles:f__:g__
MID3	denovo639	1	k__Bacteria:p__Actinobacteria:c__Actinobacteria:o__Actinomycetales:f__Geodermatophilaceae:g__Modestobacter
MID3	denovo641	2	k__Bacteria:p__Acidobacteria:c__Solibacteres:o__Solibacterales:f__:g__
MID3	denovo644	1	k__Bacteria:p__Proteobacteria:c__Alphaproteobacteria:o__Rhizobiales:f__Methylocystaceae:g__
MID3	denovo645	1	k__Bacteria:p__Proteobacteria:c__Alphaproteobacteria:o__Rhodospirillales:f__Acetobacteraceae:g__
MID3	denovo646	1	k__Bacteria:p__Proteobacteria:c__Deltaproteobacteria:o__Myxococcales:f__:g__
MID3	denovo648	1	k__Bacteria:p__Proteobacteria:c__Alphaproteobacteria:o__Rhizobiales:f__Aurantimonadaceae:g__
MID3	denovo65	5	k__Bacteria:p__Firmicutes:c__Bacilli:o__Bacillales:f__[Exiguobacteraceae]:g__Exiguobacterium
MID3	denovo650	1	k__Bacteria:p__Proteobacteria:c__Gammaproteobacteria:o__Legionellales:f__Legionellaceae:g__Legionella
MID3	denovo651	1	k__Bacteria:p__Firmicutes:c__Bacilli:o__Lactobacillales:f__Aerococcaceae:g__Alloiococcus
MID3	denovo652	1	k__Bacteria:p__Proteobacteria:c__Gammaproteobacteria:o__Xanthomonadales:f__Sinobacteraceae:g__
MID3	denovo655	11	k__Bacteria:p__Firmicutes:c__Clostridia:o__Clostridiales:f__[Tissierellaceae]:g__Anaerococcus
MID3	denovo657	8	k__Bacteria:p__Proteobacteria:c__Alphaproteobacteria:o__Rhodospirillales:f__Acetobacteraceae:g__
MID3	denovo66	6	k__Bacteria:p__Proteobacteria:c__Alphaproteobacteria:o__Rhodospirillales:f__Rhodospirillaceae:g__
MID3	denovo660	2	k__Bacteria:p__Firmicutes:c__Erysipelotrichi:o__Erysipelotrichales:f__Erysipelotrichaceae:g__[Eubacterium]
MID3	denovo661	4	k__Bacteria:p__Actinobacteria:c__Actinobacteria:o__Actinomycetales:f__:g__
MID3	denovo662	1	k__Bacteria:p__Bacteroidetes:c__Bacteroidia:o__Bacteroidales:f__Prevotellaceae:g__Prevotella
MID3	denovo663	4	k__Bacteria:p__Proteobacteria:c__Alphaproteobacteria:o__Rhizobiales:f__Methylobacteriaceae:g__Methylobacterium
MID3	denovo665	3	k__Bacteria:p__Firmicutes:c__Bacilli:o__Lactobacillales:f__Lactobacillaceae:g__Lactobacillus
MID3	denovo666	2	k__Bacteria:p__Firmicutes:c__Bacilli:o__Lactobacillales:f__Aerococcaceae:g__Aerococcus
MID3	denovo667	2	k__Bacteria:p__Actinobacteria:c__Thermoleophilia:o__Solirubrobacterales:f__:g__
MID3	denovo679	1	k__Bacteria:p__Firmicutes:c__Bacilli:o__Bacillales:f__Bacillaceae:g__Bacillus
MID3	denovo687	2	k__Bacteria:p__Firmicutes:c__Clostridia:o__Clostridiales
MID3	denovo689	4	k__Bacteria:p__Firmicutes:c__Clostridia:o__Clostridiales:f__Clostridiaceae:g__Clostridium
MID3	denovo691	2	k__Bacteria:p__Gemmatimonadetes:c__Gemmatimonadetes:o__Gemmatimonadales:f__Gemmatimonadaceae:g__Gemmatimonas
MID3	denovo692	1	k__Bacteria:p__Proteobacteria:c__Alphaproteobacteria:o__Rickettsiales:f__:g__
MID3	denovo693	2	k__Bacteria:p__Proteobacteria:c__Alphaproteobacteria:o__Caulobacterales:f__Caulobacteraceae:g__
MID3	denovo694	1	k__Bacteria:p__Actinobacteria:c__Actinobacteria:o__Actinomycetales:f__Mycobacteriaceae:g__Mycobacterium
MID3	denovo695	2	k__Bacteria:p__Bacteroidetes:c__[Saprospirae]:o__[Saprospirales]:f__Chitinophagaceae:g__
MID3	denovo696	3	k__Bacteria:p__Actinobacteria:c__Actinobacteria:o__Actinomycetales:f__Propionibacteriaceae:g__Propionibacterium
MID3	denovo699	2	k__Bacteria:p__Proteobacteria:c__Alphaproteobacteria:o__Rhodospirillales:f__Acetobacteraceae:g__
MID3	denovo70	9	k__Bacteria:p__Cyanobacteria:c__Nostocophycideae:o__Nostocales:f__Nostocaceae:g__Cylindrospermopsis
MID3	denovo700	1	k__Bacteria:p__Proteobacteria:c__Gammaproteobacteria:o__Xanthomonadales:f__Xanthomonadaceae:g__Pseudoxanthomonas
MID3	denovo703	4	k__Bacteria:p__Firmicutes:c__Bacilli:o__Bacillales:f__Staphylococcaceae:g__Macrococcus
MID3	denovo709	11	k__Bacteria:p__Proteobacteria:c__Gammaproteobacteria:o__Pseudomonadales:f__Moraxellaceae:g__Enhydrobacter
MID3	denovo71	7	k__Bacteria:p__Proteobacteria:c__Alphaproteobacteria:o__Rhizobiales:f__Hyphomicrobiaceae:g__Devosia
MID3	denovo722	1	k__Bacteria:p__Firmicutes:c__Clostridia:o__Clostridiales:f__Clostridiaceae
MID3	denovo725	1	k__Bacteria:p__Proteobacteria:c__Gammaproteobacteria:o__Enterobacteriales:f__Enterobacteriaceae:g__Erwinia
MID3	denovo729	7	k__Bacteria:p__Proteobacteria:c__Alphaproteobacteria:o__Rhizobiales:f__Phyllobacteriaceae
MID3	denovo731	1	k__Bacteria:p__Firmicutes:c__Clostridia:o__Clostridiales:f__[Tissierellaceae]:g__Finegoldia
MID3	denovo734	1	k__Bacteria:p__Proteobacteria:c__Betaproteobacteria:o__Neisseriales:f__Neisseriaceae:g__Neisseria

MID3	denovo74	1	k__Bacteria:p__Actinobacteria:c__Actinobacteria:o__Actinomycetales:f__Corynebacteriaceae:g__Corynebacterium
MID3	denovo742	1	k__Bacteria:p__Actinobacteria:c__Actinobacteria:o__Actinomycetales:f__Frankiaceae:g__
MID3	denovo748	1	k__Bacteria:p__Proteobacteria:c__Gammaproteobacteria:o__Xanthomonadales:f__Xanthomonadaceae:g__Lysobacter
MID3	denovo75	1	k__Bacteria:p__Proteobacteria:c__Betaproteobacteria:o__Burkholderiales:f__Comamonadaceae:g__Comamonas
MID3	denovo754	1	k__Bacteria:p__Proteobacteria:c__Alphaproteobacteria:o__Sphingomonadales:f__Erythrobacteraceae:g__
MID3	denovo755	2	k__Bacteria:p__Proteobacteria:c__Gammaproteobacteria:o__Xanthomonadales:f__Xanthomonadaceae:g__Dokdonella
MID3	denovo756	2	k__Bacteria:p__Actinobacteria:c__Thermoleophilia:o__Solirubrobacterales:f__Solirubrobacteraceae:g__
MID3	denovo758	2	k__Bacteria:p__Actinobacteria:c__Actinobacteria:o__Actinomycetales:f__:g__
MID3	denovo759	1	k__Bacteria:p__Proteobacteria:c__Alphaproteobacteria:o__Sphingomonadales:f__Sphingomonadaceae:g__Sphingomonas
MID3	denovo760	1	k__Bacteria:p__Firmicutes:c__Bacilli:o__Bacillales:f__Paenibacillaceae:g__Paenibacillus
MID3	denovo761	1	k__Bacteria:p__Proteobacteria:c__Alphaproteobacteria:o__Rhodobacterales:f__Rhodobacteraceae:g__Paracoccus
MID3	denovo766	2	k__Bacteria:p__Actinobacteria:c__Actinobacteria:o__Actinomycetales:f__Microbacteriaceae:g__Agrococcus
MID3	denovo767	2	k__Bacteria:p__Proteobacteria:c__Betaproteobacteria:o__Methylophilales:f__Methylophilaceae:g__Methylotenera
MID3	denovo77	1	k__Bacteria:p__Firmicutes:c__Clostridia:o__Clostridiales:f__Ruminococcaceae:g__
MID3	denovo770	52	k__Bacteria:p__Cyanobacteria:c__Oscillatoriophycideae:o__Chroococcales:f__Xenococcaceae:g__
MID3	denovo771	3	k__Bacteria:p__Armatimonadetes:c__Armatimonadia:o__Armatimonadales:f__Armatimonadaceae:g__
MID3	denovo773	1	k__Bacteria:p__Acidobacteria:c__Solibacteres:o__Solibacterales:f__:g__
MID3	denovo774	1	k__Bacteria:p__Proteobacteria:c__Gammaproteobacteria:o__Xanthomonadales:f__Sinobacteraceae:g__
MID3	denovo783	3	k__Bacteria:p__Cyanobacteria:c__Oscillatoriophycideae:o__Oscillatoriales:f__Phormidiaceae:g__Phormidium
MID3	denovo784	3	k__Bacteria:p__Proteobacteria:c__Gammaproteobacteria:o__Xanthomonadales:f__Xanthomonadaceae:g__
MID3	denovo786	7	k__Bacteria:p__Firmicutes:c__Clostridia:o__Clostridiales
MID3	denovo787	2	k__Bacteria:p__Firmicutes:c__Clostridia:o__Clostridiales:f__[Mogibacteriaceae]:g__Mogibacterium
MID3	denovo789	2	k__Bacteria:p__Proteobacteria:c__Betaproteobacteria:o__Burkholderiales:f__Oxalobacteraceae:g__Janthinobacterium
MID3	denovo79	25	k__Bacteria:p__Firmicutes:c__Clostridia:o__Clostridiales:f__[Tissierellaceae]:g__Anaerococcus
MID3	denovo790	2	k__Bacteria:p__Cyanobacteria:c__Chloroplast:o__Streptophyta:f__:g__
MID3	denovo795	1	k__Bacteria:p__Actinobacteria:c__Actinobacteria:o__Actinomycetales:f__:g__
MID3	denovo796	4	k__Bacteria:p__Proteobacteria:c__Alphaproteobacteria:o__Rhodobacterales:f__Rhodobacteraceae:g__Rubellimicrobium
MID3	denovo797	1	k__Bacteria:p__Proteobacteria:c__Alphaproteobacteria:o__Rhizobiales:f__Phyllobacteriaceae:g__
MID3	denovo799	1	k__Bacteria:p__Firmicutes:c__Clostridia:o__Clostridiales:f__Lachnospiraceae:g__Coprococcus
MID3	denovo803	2	k__Bacteria:p__Actinobacteria:c__Actinobacteria:o__Actinomycetales:f__Sporichthyaceae:g__
MID3	denovo806	12	k__Bacteria:p__Firmicutes:c__Bacilli:o__Bacillales:f__Bacillaceae:g__Bacillus
MID3	denovo807	8	k__Bacteria:p__Firmicutes:c__Clostridia:o__Clostridiales:f__Lachnospiraceae:g__
MID3	denovo81	3	k__Bacteria:p__Acidobacteria:c__Acidobacteria-6:o__iii1-15:f__:g__
MID3	denovo811	2	k__Bacteria:p__Proteobacteria:c__Betaproteobacteria:o__Burkholderiales:f__Comamonadaceae
MID3	denovo818	3	k__Bacteria:p__Proteobacteria:c__Alphaproteobacteria:o__Rhodobacterales:f__Rhodobacteraceae:g__Rubellimicrobium
MID3	denovo819	1	k__Bacteria:p__Proteobacteria:c__Alphaproteobacteria:o__Rhizobiales:f__Hyphomicrobiaceae:g__Rhodoplanes
MID3	denovo821	1	k__Bacteria:p__Proteobacteria:c__Alphaproteobacteria:o__Rhizobiales:f__Methylocystaceae:g__
MID3	denovo824	14	k__Bacteria:p__Firmicutes:c__Bacilli:o__Bacillales:f__Paenibacillaceae:g__Paenibacillus
MID3	denovo829	7	k__Bacteria:p__Firmicutes:c__Bacilli:o__Lactobacillales:f__Carnobacteriaceae:g__Carnobacterium
MID3	denovo83	3	k__Bacteria:p__Actinobacteria:c__Actinobacteria:o__Actinomycetales:f__Corynebacteriaceae:g__Corynebacterium
MID3	denovo831	1	k__Bacteria:p__Actinobacteria:c__Actinobacteria:o__Actinomycetales:f__Propionibacteriaceae:g__
MID3	denovo832	2	k__Bacteria:p__Firmicutes:c__Clostridia:o__Clostridiales:f__Clostridiaceae:g__Clostridium
MID3	denovo834	4	k__Bacteria:p__Proteobacteria:c__Alphaproteobacteria:o__Sphingomonadales:f__Sphingomonadaceae:g__Sphingomonas
MID3	denovo836	8	k__Bacteria:p__Cyanobacteria:c__Oscillatoriophycideae:o__Chroococcales:f__Xenococcaceae
MID3	denovo844	4	k__Bacteria:p__Proteobacteria:c__Betaproteobacteria:o__Burkholderiales:f__Comamonadaceae:g__
MID3	denovo852	2	k__Bacteria:p__Firmicutes:c__Clostridia:o__Clostridiales:f__Lachnospiraceae:g__
MID3	denovo853	13	k__Bacteria:p__Proteobacteria:c__Betaproteobacteria:o__Burkholderiales:f__:g__
MID3	denovo861	1	k__Bacteria:p__Actinobacteria:c__Thermoleophilia:o__Solirubrobacterales:f__:g__
MID3	denovo865	2	k__Bacteria:p__Planctomycetes:c__Planctomycetia:o__Gemmatales:f__Isosphaeraceae:g__
MID3	denovo866	3	k__Bacteria:p__Proteobacteria:c__Deltaproteobacteria:o__Spirobacillales:f__:g__
MID3	denovo868	8	k__Bacteria:p__Firmicutes:c__Clostridia:o__Clostridiales:f__[Tissierellaceae]:g__Anaerococcus
MID3	denovo869	1	k__Bacteria:p__Proteobacteria:c__Alphaproteobacteria:o__Rhizobiales:f__Hyphomicrobiaceae:g__Pedomicrobium
MID3	denovo871	1	k__Bacteria:p__Firmicutes:c__Bacilli:o__Lactobacillales:f__Streptococcaceae:g__Lactococcus
MID3	denovo875	2	k__Bacteria:p__Firmicutes:c__Clostridia:o__Clostridiales:f__Clostridiaceae:g__
MID3	denovo876	2	k__Bacteria:p__Actinobacteria:c__Actinobacteria:o__Actinomycetales:f__Mycobacteriaceae:g__Mycobacterium
MID3	denovo878	18	k__Bacteria:p__Firmicutes:c__Bacilli:o__Lactobacillales:f__Streptococcaceae:g__Streptococcus
MID3	denovo879	1	k__Bacteria:p__Planctomycetes:c__Planctomycetia:o__Pirellulales:f__Pirellulaceae:g__
MID3	denovo880	3	k__Bacteria:p__Firmicutes:c__Clostridia:o__Clostridiales:f__Ruminococcaceae:g__Ruminococcus
MID3	denovo883	4	k__Bacteria:p__Proteobacteria:c__Alphaproteobacteria:o__Rhizobiales:f__Hyphomicrobiaceae:g__Rhodoplanes
MID3	denovo885	11	k__Bacteria:p__Firmicutes:c__Clostridia:o__Clostridiales:f__Ruminococcaceae:g__
MID3	denovo886	7	k__Bacteria:p__Firmicutes:c__Clostridia:o__Clostridiales:f__[Tissierellaceae]:g__Peptoniphilus
MID3	denovo887	2	k__Bacteria:p__Proteobacteria:c__Alphaproteobacteria:o__Rhizobiales:f__Beijerinckiaceae:g__
MID3	denovo891	1	k__Bacteria:p__Acidobacteria:c__Acidobacteria-6:o__iii1-15:f__:g__
MID3	denovo892	1	k__Bacteria:p__Firmicutes:c__Clostridia:o__Clostridiales:f__[Tissierellaceae]:g__Peptoniphilus
MID3	denovo896	1	k__Bacteria:p__Bacteroidetes:c__Bacteroidia:o__Bacteroidales:f__Prevotellaceae:g__Prevotella
MID3	denovo897	6	k__Bacteria:p__Actinobacteria:c__Actinobacteria:o__Actinomycetales:f__Corynebacteriaceae:g__Corynebacterium
MID3	denovo902	2	k__Bacteria:p__Cyanobacteria:c__Oscillatoriophycideae:o__Chroococcales:f__Xenococcaceae:g__

MID3	denovo904	1	k__Bacteria;p__Firmicutes;c__Bacilli;o__Bacillales;f__Bacillaceae;g__Bacillus
MID3	denovo905	1	k__Bacteria;p__Acidobacteria;c__Acidobacteria-6;o__iii1-15;f__mb2424;g__
MID3	denovo907	2	k__Bacteria;p__Proteobacteria;c__Gammaproteobacteria;o__Xanthomonadales;f__Sinobacteraceae;g__
MID3	denovo908	1	k__Bacteria;p__Firmicutes;c__Clostridia;o__Clostridiales;f__Ruminococcaceae;g__
MID3	denovo909	2	k__Bacteria;p__Actinobacteria;c__Actinobacteria;o__Actinomycetales;f__Propionibacteriaceae;g__
MID3	denovo911	1	k__Bacteria;p__Acidobacteria;c__[Chloracidobacteria];o__RB41;f__Ellin6075;g__
MID3	denovo918	2	k__Bacteria;p__Actinobacteria;c__Actinobacteria;o__Actinomycetales;f__Microbacteriaceae;g__Rathayibacter
MID3	denovo919	3	k__Bacteria;p__Bacteroidetes;c__Cytophagia;o__Cytophagales;f__Cytophagaceae;g__Hymenobacter
MID3	denovo921	2	k__Bacteria;p__Chloroflexi;c__TK10;o__;f__;g__
MID3	denovo922	1	k__Bacteria;p__Proteobacteria;c__Alphaproteobacteria;o__Rhizobiales;f__Bradyrhizobiaceae
MID3	denovo923	1	k__Bacteria;p__Firmicutes;c__Clostridia;o__Clostridiales;f__Ruminococcaceae;g__
MID3	denovo927	2	k__Bacteria;p__Planctomycetes;c__Planctomycetia;o__Gemmatales;f__Isosphaeraceae;g__
MID3	denovo928	9	k__Bacteria;p__Proteobacteria;c__Alphaproteobacteria;o__Rhodospirillales;f__Acetobacteraceae;g__
MID3	denovo929	1	k__Bacteria;p__Actinobacteria;c__Actinobacteria;o__Actinomycetales;f__Corynebacteriaceae;g__Corynebacterium
MID3	denovo93	11	k__Bacteria;p__Bacteroidetes;c__Bacteroidia;o__Bacteroidales;f__Prevotellaceae;g__Prevotella
MID3	denovo930	1	k__Bacteria;p__Proteobacteria;c__Alphaproteobacteria;o__Rhodospirillales;f__Acetobacteraceae;g__
MID3	denovo935	2	k__Bacteria;p__Proteobacteria;c__Gammaproteobacteria;o__Xanthomonadales;f__Xanthomonadaceae;g__Thermomonas
MID3	denovo938	3	k__Bacteria;p__Actinobacteria;c__Actinobacteria;o__Actinomycetales;f__Brevibacteriaceae;g__Brevibacterium
MID3	denovo946	5	k__Bacteria;p__Proteobacteria;c__Gammaproteobacteria;o__Xanthomonadales;f__Xanthomonadaceae;g__Lysobacter
MID3	denovo947	1	k__Bacteria;p__Firmicutes;c__Clostridia;o__Clostridiales;f__Ruminococcaceae;g__
MID3	denovo949	5	k__Bacteria;p__Proteobacteria;c__Alphaproteobacteria;o__Caulobacterales;f__Caulobacteraceae;g__
MID3	denovo95	1	k__Bacteria;p__Bacteroidetes;c__Flavobacteriia;o__Flavobacteriales;f__[Weeksellaceae];g__Chryseobacterium
MID3	denovo952	1	k__Bacteria;p__Chloroflexi;c__Ellin6529;o__;f__;g__
MID3	denovo953	2	k__Bacteria;p__Actinobacteria;c__Actinobacteria;o__Actinomycetales;f__Microbacteriaceae;g__Curtobacterium
MID3	denovo955	3	k__Bacteria;p__Proteobacteria;c__Alphaproteobacteria;o__Rhizobiales;f__Methylobacteriaceae;g__Methylobacterium
MID3	denovo956	2	k__Bacteria;p__Cyanobacteria;c__Chloroplast;o__Chlorophyta;f__;g__
MID3	denovo957	6	k__Bacteria;p__Actinobacteria;c__MB-A2-108;o__0319-7L14;f__;g__
MID3	denovo96	11	k__Bacteria;p__Firmicutes;c__Clostridia;o__Clostridiales;f__[Tissierellaceae];g__Anaerococcus
MID3	denovo960	1	k__Bacteria;p__Proteobacteria;c__Alphaproteobacteria;o__Rhodospirillales;f__Rhodospirillaceae;g__
MID3	denovo963	1	k__Bacteria;p__Proteobacteria;c__Alphaproteobacteria;o__Sphingomonadales;f__Sphingomonadaceae;g__
MID3	denovo964	1	k__Bacteria;p__Proteobacteria;c__Gammaproteobacteria;o__Pseudomonadales;f__Moraxellaceae;g__Acinetobacter
MID3	denovo973	7	k__Bacteria;p__Firmicutes;c__Bacilli;o__Bacillales;f__Staphylococcaceae;g__Jeotgalicoccus
MID3	denovo974	1	k__Bacteria;p__Bacteroidetes;c__Bacteroidia;o__Bacteroidales;f__Porphyromonadaceae;g__
MID3	denovo977	2	k__Bacteria;p__Cyanobacteria;c__Nostocophycideae;o__Stigonematales;f__Rivulariaceae;g__Calothrix
MID3	denovo978	1	k__Bacteria;p__Proteobacteria;c__Alphaproteobacteria;o__BD7-3;f__;g__
MID3	denovo979	4	k__Bacteria;p__Proteobacteria;c__Alphaproteobacteria;o__Sphingomonadales;f__Sphingomonadaceae;g__Sphingomonas
MID3	denovo98	3	k__Bacteria;p__Bacteroidetes;c__Cytophagia;o__Cytophagales;f__Cytophagaceae;g__
MID3	denovo980	1	k__Bacteria;p__Actinobacteria;c__Thermoleophilia;o__Solirubrobacterales;f__Solirubrobacteraceae;g__
MID3	denovo981	7	k__Bacteria;p__Firmicutes;c__Bacilli;o__Lactobacillales;f__Leuconostocaceae;g__
MID3	denovo99	1	k__Bacteria;p__WPS-2;c__;o__;f__;g__
MID3	denovo992	2	k__Bacteria;p__Proteobacteria;c__Betaproteobacteria;o__Neisseriales;f__Neisseriaceae;g__
MID3	denovo999	2	k__Bacteria;p__Proteobacteria;c__Alphaproteobacteria;o__Rhizobiales;f__;g__
MID4	denovo1000	1	k__Bacteria;p__Bacteroidetes;c__Flavobacteriia;o__Flavobacteriales;f__Flavobacteriaceae;g__Flavobacterium
MID4	denovo1001	1	k__Bacteria;p__Proteobacteria;c__Alphaproteobacteria;o__Rhodobacterales;f__Rhodobacteraceae;g__Rubellimicrobium
MID4	denovo1005	1	k__Bacteria;p__Proteobacteria;c__Alphaproteobacteria;o__Rhodobacterales;f__Rhodobacteraceae;g__Rubellimicrobium
MID4	denovo1013	5	k__Bacteria;p__Cyanobacteria;c__Nostocophycideae;o__Nostocales;f__Nostocaceae
MID4	denovo1014	3	k__Bacteria;p__Proteobacteria;c__Alphaproteobacteria;o__Rhizobiales;f__Bradyrhizobiaceae;g__Balneimonas
MID4	denovo1015	6	k__Bacteria;p__Firmicutes;c__Clostridia;o__Clostridiales;f__[Tissierellaceae];g__1-68
MID4	denovo1018	12	k__Bacteria;p__Firmicutes;c__Clostridia;o__Clostridiales;f__[Tissierellaceae];g__Peptoniphilus
MID4	denovo1019	1	k__Bacteria;p__Firmicutes;c__Bacilli;o__Bacillales;f__Bacillaceae;g__Bacillus
MID4	denovo1020	1	k__Bacteria;p__Cyanobacteria;c__Chloroplast;o__Streptophyta;f__;g__
MID4	denovo1029	1	k__Bacteria;p__Nitrospirae;c__Nitrospira;o__Nitrospirales;f__;g__
MID4	denovo103	1	k__Bacteria;p__Bacteroidetes;c__Bacteroidia;o__Bacteroidales;f__Prevotellaceae;g__Prevotella
MID4	denovo1031	1	k__Bacteria;p__Actinobacteria;c__Actinobacteria;o__Actinomycetales
MID4	denovo1034	1	k__Bacteria;p__Firmicutes;c__Clostridia;o__Clostridiales;f__Lachnospiraceae;g__Anaerostipes
MID4	denovo1035	3	k__Bacteria;p__Actinobacteria;c__Actinobacteria;o__Actinomycetales;f__Micrococcaceae;g__
MID4	denovo1037	5	k__Bacteria;p__Actinobacteria;c__Actinobacteria;o__Actinomycetales;f__Corynebacteriaceae;g__Corynebacterium
MID4	denovo1040	3	k__Bacteria;p__Proteobacteria;c__Alphaproteobacteria;o__Rickettsiales;f__Rickettsiaceae;g__Rickettsia
MID4	denovo1044	1	k__Bacteria;p__Firmicutes;c__Clostridia;o__Clostridiales;f__Lachnospiraceae;g__Coprococcus
MID4	denovo1045	5	k__Bacteria;p__Actinobacteria;c__Actinobacteria;o__Actinomycetales;f__Micrococcaceae;g__Kocuria
MID4	denovo1047	1	k__Bacteria;p__Bacteroidetes;c__Flavobacteriia;o__Flavobacteriales;f__Flavobacteriaceae;g__Flavobacterium
MID4	denovo1049	1	k__Bacteria;p__Proteobacteria;c__Gammaproteobacteria;o__Legionellales;f__;g__
MID4	denovo105	4	k__Bacteria;p__Actinobacteria;c__Actinobacteria;o__Actinomycetales;f__Brevibacteriaceae;g__Brevibacterium
MID4	denovo1052	1	k__Bacteria;p__Firmicutes;c__Clostridia;o__Clostridiales;f__Veillonellaceae;g__Phascolarctobacterium
MID4	denovo1053	1	k__Bacteria;p__Proteobacteria;c__Alphaproteobacteria;o__Rhizobiales;f__Beijerinckiaceae;g__
MID4	denovo1059	3	k__Bacteria;p__Actinobacteria;c__Actinobacteria;o__Actinomycetales;f__Microbacteriaceae;g__Microbacterium
MID4	denovo106	3	k__Bacteria;p__Firmicutes;c__Clostridia;o__Clostridiales;f__Lachnospiraceae

MID4	denovo1060	3	k__Bacteria:p__Proteobacteria:c__Alphaproteobacteria:o__Sphingomonadales:f__Sphingomonadaceae:g__
MID4	denovo1064	1	k__Bacteria:p__Proteobacteria:c__Alphaproteobacteria:o__Rhizobiales:f__Hyphomicrobiaceae:g__
MID4	denovo1067	1	k__Bacteria:p__Proteobacteria:c__Alphaproteobacteria:o__Rhizobiales:f__Beijerinckiaceae:g__
MID4	denovo1068	2	k__Bacteria:p__Proteobacteria:c__Gammaproteobacteria:o__Xanthomonadales:f__Xanthomonadaceae:g__Stenotrophomonas
MID4	denovo1069	1	k__Bacteria:p__Firmicutes:c__Clostridia:o__Clostridiales:f__Ruminococcaceae:g__Ruminococcus
MID4	denovo107	1	k__Bacteria:p__Proteobacteria:c__Alphaproteobacteria:o__Rhodobacterales:f__Rhodobacteraceae:g__Rubellimicrobium
MID4	denovo1073	1	k__Bacteria:p__Proteobacteria:c__Gammaproteobacteria:o__Xanthomonadales:f__Xanthomonadaceae:g__Stenotrophomonas
MID4	denovo1079	8	k__Bacteria:p__Firmicutes:c__Bacilli:o__Lactobacillales:f__Aerococcaceae:g__Facklamia
MID4	denovo108	1	k__Bacteria:p__Actinobacteria:c__Actinobacteria:o__Actinomycetales:f__Microbacteriaceae:g__Microbacterium
MID4	denovo1084	4	k__Bacteria:p__Proteobacteria:c__Alphaproteobacteria:o__Caulobacterales:f__Caulobacteraceae:g__
MID4	denovo1086	4	k__Bacteria:p__Actinobacteria:c__Actinobacteria:o__Actinomycetales:f__Nocardioidaceae:g__
MID4	denovo1088	3	k__Bacteria:p__Actinobacteria:c__Actinobacteria:o__Actinomycetales:f__Kineosporiaceae:g__
MID4	denovo109	4	k__Bacteria:p__Proteobacteria:c__Gammaproteobacteria:o__Enterobacteriales:f__Enterobacteriaceae
MID4	denovo1090	2	k__Bacteria:p__Proteobacteria:c__Alphaproteobacteria:o__Rhodobacterales:f__Rhodobacteraceae:g__Amaricoccus
MID4	denovo1093	1	k__Bacteria:p__Firmicutes:c__Bacilli:o__Lactobacillales:f__Lactobacillaceae:g__Lactobacillus
MID4	denovo1094	51	k__Bacteria:p__Firmicutes:c__Bacilli:o__Lactobacillales:f__Aerococcaceae:g__Facklamia
MID4	denovo1096	1	k__Bacteria:p__Proteobacteria:c__Gammaproteobacteria:o__Legionellales:f__:g__
MID4	denovo1099	2	k__Bacteria:p__Firmicutes:c__Clostridia:o__Clostridiales:f__Lachnospiraceae:g__
MID4	denovo1103	1	k__Bacteria:p__Gemmatimonadetes:c__Gemmatimonadetes:o__Gemmatimonadales:f__:g__
MID4	denovo1104	1	k__Bacteria:p__Bacteroidetes:c__Bacteroidia:o__Bacteroidales:f__Prevotellaceae:g__Prevotella
MID4	denovo1105	1	k__Bacteria:p__Proteobacteria:c__Alphaproteobacteria:o__Rhodobacterales:f__Rhodobacteraceae:g__Rhodobacter
MID4	denovo1107	1	k__Bacteria:p__Proteobacteria:c__Deltaproteobacteria:o__:f__:g__
MID4	denovo1108	1	k__Bacteria:p__Proteobacteria:c__Alphaproteobacteria:o__Caulobacterales:f__Caulobacteraceae
MID4	denovo1111	1	k__Bacteria:p__Bacteroidetes:c__Flavobacteriia:o__Flavobacteriales:f__Flavobacteriaceae:g__Flavobacterium
MID4	denovo1115	2	k__Bacteria:p__Cyanobacteria:c__Synechococcophycideae:o__Pseudanabaenales:f__Pseudanabaenaceae:g__
MID4	denovo1117	1	k__Bacteria:p__Proteobacteria:c__Alphaproteobacteria:o__Rhodospirillales:f__Acetobacteraceae:g__
MID4	denovo112	1	k__Bacteria:p__Firmicutes:c__Clostridia:o__Clostridiales:f__Lachnospiraceae:g__
MID4	denovo1121	2	k__Bacteria:p__Bacteroidetes:c__Flavobacteriia:o__Flavobacteriales:f__[Weeksellaceae]:g__Chryseobacterium
MID4	denovo1122	2	k__Bacteria:p__Proteobacteria:c__Gammaproteobacteria:o__Legionellales:f__Coxiellaceae:g__Rickettsiella
MID4	denovo1127	1	k__Bacteria:p__Actinobacteria:c__Actinobacteria:o__Actinomycetales:f__Nocardioidaceae
MID4	denovo1128	1	k__Bacteria:p__Acidobacteria:c__iii1-8:o__32-20:f__:g__
MID4	denovo1129	1	k__Bacteria:p__Planctomycetes:c__Planctomycetia:o__Gemmatales:f__Isosphaeraceae:g__
MID4	denovo114	1	k__Bacteria:p__Cyanobacteria:c__Chloroplast:o__Chlorophyta:f__:g__
MID4	denovo1149	5	k__Bacteria:p__Proteobacteria:c__Alphaproteobacteria:o__Rhizobiales:f__Methylobacteriaceae:g__Methylobacterium
MID4	denovo115	2	k__Bacteria:p__Gemmatimonadetes:c__Gemmatimonadetes:o__Gemmatimonadales:f__:g__
MID4	denovo1150	5	k__Bacteria:p__Firmicutes:c__Clostridia:o__Clostridiales:f__Lachnospiraceae:g__Roseburia
MID4	denovo1151	3	k__Bacteria:p__Firmicutes:c__Bacilli:o__Bacillales:f__Planococcaceae:g__Solibacillus
MID4	denovo1152	3	k__Bacteria:p__Cyanobacteria:c__Synechococcophycideae:o__Pseudanabaenales:f__Pseudanabaenaceae:g__Leptolyngbya
MID4	denovo1154	1	k__Bacteria:p__Proteobacteria:c__Betaproteobacteria:o__Burkholderiales:f__Alcaligenaceae:g__Pigmentiphaga
MID4	denovo1156	1	k__Bacteria:p__Proteobacteria:c__Gammaproteobacteria:o__Xanthomonadales:f__Xanthomonadaceae:g__Luteimonas
MID4	denovo1157	1	k__Bacteria:p__Proteobacteria:c__Alphaproteobacteria:o__Rhodobacterales:f__Rhodobacteraceae:g__Amaricoccus
MID4	denovo1159	2	k__Bacteria:p__Proteobacteria:c__Alphaproteobacteria:o__Rhizobiales:f__Bradyrhizobiaceae:g__
MID4	denovo116	2	k__Bacteria:p__Firmicutes:c__Bacilli:o__Lactobacillales:f__Streptococcaceae:g__Streptococcus
MID4	denovo1161	2	k__Bacteria:p__Firmicutes:c__Bacilli:o__Bacillales:f__Staphylococcaceae:g__Salinicoccus
MID4	denovo1165	4	k__Bacteria:p__Firmicutes:c__Clostridia:o__Clostridiales:f__[Tissierellaceae]:g__Anaerococcus
MID4	denovo1167	2	k__Bacteria:p__Firmicutes:c__Clostridia:o__Clostridiales:f__[Tissierellaceae]:g__Anaerococcus
MID4	denovo1173	2	k__Bacteria:p__Proteobacteria:c__Alphaproteobacteria:o__Rhodobacterales:f__Rhodobacteraceae:g__Paracoccus
MID4	denovo1177	1	k__Bacteria:p__Proteobacteria:c__Gammaproteobacteria:o__Enterobacteriales:f__Enterobacteriaceae
MID4	denovo1178	3	k__Bacteria:p__Firmicutes:c__Clostridia:o__Clostridiales:f__Lachnospiraceae:g__
MID4	denovo1185	1	k__Bacteria:p__Bacteroidetes:c__Cytophagia:o__Cytophagales:f__Cytophagaceae:g__Emticicia
MID4	denovo1186	1	k__Bacteria:p__Firmicutes:c__Clostridia:o__Clostridiales:f__:g__
MID4	denovo1188	1	k__Bacteria:p__Proteobacteria:c__Alphaproteobacteria:o__Rhizobiales:f__Rhodobiaceae:g__Afifella
MID4	denovo1192	1	k__Bacteria:p__Gemmatimonadetes:c__Gemmatimonadetes:o__Gemmatimonadales:f__:g__
MID4	denovo1195	1	k__Bacteria:p__Proteobacteria:c__Alphaproteobacteria:o__Rhizobiales:f__Phyllobacteriaceae:g__Aminobacter
MID4	denovo1196	1	k__Bacteria:p__Planctomycetes:c__Planctomycetia:o__Gemmatales:f__Isosphaeraceae:g__
MID4	denovo1202	7	k__Bacteria:p__Proteobacteria:c__Alphaproteobacteria:o__Rhizobiales:f__Methylobacteriaceae:g__Methylobacterium
MID4	denovo1208	1	k__Bacteria:p__Proteobacteria:c__Betaproteobacteria:o__Burkholderiales:f__Comamonadaceae:g__
MID4	denovo1217	3	k__Bacteria:p__Cyanobacteria:c__Chloroplast:o__Chlorophyta:f__:g__
MID4	denovo122	1	k__Bacteria:p__Firmicutes:c__Clostridia:o__Clostridiales:f__Ruminococcaceae:g__Ruminococcus
MID4	denovo1227	2	k__Bacteria:p__Proteobacteria:c__Alphaproteobacteria:o__Rhizobiales:f__Rhizobiaceae
MID4	denovo123	2	k__Bacteria:p__Fibrobacteres:c__Fibrobacteria:o__258ds10:f__:g__
MID4	denovo1231	4	k__Bacteria:p__Proteobacteria:c__Alphaproteobacteria:o__Rhodobacterales:f__Rhodobacteraceae:g__Rubellimicrobium
MID4	denovo1235	1	k__Bacteria:p__Firmicutes:c__Clostridia:o__Clostridiales:f__Clostridiaceae:g__Clostridium
MID4	denovo1236	1	k__Bacteria:p__Bacteroidetes:c__Cytophagia:o__Cytophagales:f__Cytophagaceae:g__Rhodocytophaga
MID4	denovo1239	2	k__Bacteria:p__Firmicutes:c__Bacilli:o__Lactobacillales:f__Aerococcaceae:g__Facklamia
MID4	denovo1244	3	k__Bacteria:p__Proteobacteria:c__Alphaproteobacteria:o__Caulobacterales:f__Caulobacteraceae:g__
MID4	denovo1245	1	k__Bacteria:p__Armatimonadetes:c__Armatimonadia:o__Armatimonadales:f__Armatimonadaceae:g__

MID4	denovo1246	1	k__Bacteria:p__Acidobacteria:c__Acidobacteria-6:o__iii1-15:f__mb2424:g__
MID4	denovo1247	1	k__Bacteria:p__Proteobacteria:c__Gammaproteobacteria:o__Xanthomonadales:f__Xanthomonadaceae
MID4	denovo1252	2	k__Bacteria:p__Proteobacteria:c__Alphaproteobacteria:o__Rhizobiales:f__Methylobacteriaceae:g__Methylobacterium
MID4	denovo1253	7	k__Bacteria:p__Proteobacteria:c__Alphaproteobacteria:o__Rhizobiales:f__Rhizobiaceae:g__Agrobacterium
MID4	denovo1255	1	k__Bacteria:p__Bacteroidetes:c__Bacteroidia:o__Bacteroidales:f__Prevotellaceae:g__Prevotella
MID4	denovo1257	21	k__Bacteria:p__Proteobacteria:c__Alphaproteobacteria:o__Sphingomonadales:f__Sphingomonadaceae
MID4	denovo1258	2	k__Bacteria:p__Proteobacteria:c__Alphaproteobacteria:o__Rhodospirillales:f__Acetobacteraceae:g__
MID4	denovo1261	2	k__Bacteria:p__Bacteroidetes:c__Cytophagia:o__Cytophagales:f__Cytophagaceae:g__Rudanella
MID4	denovo1273	1	k__Bacteria:p__Armatimonadetes:c__0319-6E2:o__:f__:g__
MID4	denovo1277	1	k__Bacteria:p__Firmicutes:c__Bacilli:o__Bacillales:f__Paenibacillaceae:g__Paenibacillus
MID4	denovo1278	1	k__Bacteria:p__Proteobacteria:c__Alphaproteobacteria:o__Rhizobiales:f__Methylobacteriaceae:g__Methylobacterium
MID4	denovo1279	2	k__Bacteria:p__Firmicutes:c__Bacilli:o__Lactobacillales
MID4	denovo1282	1	k__Bacteria:p__Planctomycetes:c__Planctomycetia:o__Gemmatales:f__Isosphaeraceae:g__
MID4	denovo1287	4	k__Bacteria:p__Actinobacteria:c__Actinobacteria:o__Actinomycetales:f__Corynebacteriaceae:g__Corynebacterium
MID4	denovo1289	1	k__Bacteria:p__Chloroflexi:c__Thermomicrobia:o__JG30-KF-CM45:f__:g__
MID4	denovo1290	10	k__Bacteria:p__Actinobacteria:c__Actinobacteria:o__Actinomycetales:f__Dermabacteraceae:g__Brachybacterium
MID4	denovo1291	2	k__Bacteria:p__Bacteroidetes:c__Flavobacteriia:o__Flavobacteriales:f__Flavobacteriaceae:g__Flavobacterium
MID4	denovo1295	1	k__Bacteria:p__Bacteroidetes:c__Bacteroidia:o__Bacteroidales:f__Porphyromonadaceae:g__Porphyromonas
MID4	denovo1300	14	k__Bacteria:p__Actinobacteria:c__Actinobacteria:o__Actinomycetales:f__Nocardioidaceae:g__Friedmanniella
MID4	denovo1301	2	k__Bacteria:p__Actinobacteria:c__Coriobacteriia:o__Coriobacteriales:f__Coriobacteriaceae:g__Collinsella
MID4	denovo1303	1	k__Bacteria:p__Actinobacteria:c__Actinobacteria:o__Actinomycetales:f__Propionibacteriaceae:g__
MID4	denovo1306	1	k__Bacteria:p__Proteobacteria:c__Alphaproteobacteria:o__Rhizobiales:f__:g__
MID4	denovo1308	10	k__Bacteria:p__Firmicutes:c__Bacilli:o__Lactobacillales:f__Streptococcaceae:g__Streptococcus
MID4	denovo131	2	k__Bacteria:p__Actinobacteria:c__Acidimicrobiia:o__Acidimicrobiales:f__:g__
MID4	denovo1311	3	k__Bacteria:p__Firmicutes:c__Clostridia:o__Clostridiales:f__Clostridiaceae:g__
MID4	denovo1313	1	k__Bacteria:p__Acidobacteria:c__Solibacteres:o__Solibacterales:f__:g__
MID4	denovo1315	1	k__Bacteria:p__Firmicutes:c__Clostridia:o__Clostridiales:f__:g__
MID4	denovo132	1	k__Bacteria:p__Proteobacteria:c__Alphaproteobacteria:o__Sphingomonadales:f__Erythrobacteraceae:g__
MID4	denovo1321	1	k__Bacteria:p__Actinobacteria:c__Actinobacteria:o__Actinomycetales:f__Pseudonocardiaceae:g__
MID4	denovo1324	1	k__Bacteria:p__Proteobacteria:c__Alphaproteobacteria:o__Rhodospirillales:f__Acetobacteraceae:g__
MID4	denovo1329	1	k__Bacteria:p__Acidobacteria:c__[Chloracidobacteria]:o__RB41:f__Ellin6075:g__
MID4	denovo1330	1	k__Bacteria:p__Bacteroidetes:c__Bacteroidia:o__Bacteroidales:f__Prevotellaceae:g__Prevotella
MID4	denovo1334	38	k__Bacteria:p__Firmicutes:c__Bacilli:o__Lactobacillales:f__Streptococcaceae:g__Streptococcus
MID4	denovo1339	1	k__Bacteria:p__Actinobacteria:c__Acidimicrobiia:o__Acidimicrobiales:f__C111:g__
MID4	denovo134	15	k__Bacteria:p__Firmicutes:c__Bacilli:o__Lactobacillales:f__:g__
MID4	denovo1341	8	k__Bacteria:p__Firmicutes:c__Clostridia:o__Clostridiales:f__[Tissierellaceae]:g__Anaerococcus
MID4	denovo1343	2	k__Bacteria:p__Proteobacteria:c__Alphaproteobacteria:o__Sphingomonadales:f__Sphingomonadaceae:g__Sphingomonas
MID4	denovo1345	1	k__Bacteria:p__Firmicutes:c__Bacilli:o__Bacillales:f__Alicyclobacillaceae:g__Alicyclobacillus
MID4	denovo1346	9	k__Bacteria:p__Firmicutes:c__Bacilli:o__Lactobacillales:f__Streptococcaceae:g__Lactococcus
MID4	denovo1347	1	k__Bacteria:p__Proteobacteria:c__Gammaproteobacteria:o__Xanthomonadales:f__Xanthomonadaceae:g__Luteibacter
MID4	denovo1350	37	k__Bacteria:p__Firmicutes:c__Clostridia:o__Clostridiales:f__[Tissierellaceae]:g__Anaerococcus
MID4	denovo1351	1	k__Bacteria:p__Proteobacteria:c__Alphaproteobacteria:o__Rhizobiales:f__Methylocystaceae:g__
MID4	denovo136	1	k__Bacteria:p__Chloroflexi:c__Chloroflexi:o__[Roseiflexales]:f__:g__
MID4	denovo137	3	k__Bacteria:p__Cyanobacteria:c__Chloroplast:o__Chlorophyta:f__:g__
MID4	denovo138	1	k__Bacteria:p__Proteobacteria:c__Alphaproteobacteria:o__Rhodospirillales:f__Rhodospirillaceae:g__Skermanella
MID4	denovo145	1	k__Bacteria:p__Bacteroidetes:c__Bacteroidia:o__Bacteroidales:f__Prevotellaceae:g__Prevotella
MID4	denovo15	1	k__Bacteria:p__[Thermi]:c__Deinococci:o__Deinococcales:f__Trueperaceae:g__Truepera
MID4	denovo152	1	k__Bacteria:p__Actinobacteria:c__Thermoleophilia:o__Solirubrobacterales:f__:g__
MID4	denovo155	4	k__Bacteria:p__Proteobacteria:c__Alphaproteobacteria:o__Rhodobacterales:f__Rhodobacteraceae:g__Rubellimicrobium
MID4	denovo156	1	k__Bacteria:p__Acidobacteria:c__[Chloracidobacteria]:o__RB41:f__:g__
MID4	denovo158	3	k__Bacteria:p__Proteobacteria:c__Alphaproteobacteria:o__Sphingomonadales:f__Sphingomonadaceae:g__
MID4	denovo165	1	k__Bacteria:p__Proteobacteria:c__Deltaproteobacteria:o__Spirobacillales:f__:g__
MID4	denovo169	1	k__Bacteria:p__Actinobacteria:c__Acidimicrobiia:o__Acidimicrobiales:f__:g__
MID4	denovo170	1	k__Bacteria:p__Firmicutes:c__Clostridia:o__Clostridiales:f__[Tissierellaceae]:g__Anaerococcus
MID4	denovo174	1	k__Bacteria:p__Proteobacteria:c__Alphaproteobacteria:o__Sphingomonadales:f__Sphingomonadaceae:g__
MID4	denovo175	1	k__Bacteria:p__Firmicutes:c__Clostridia:o__Clostridiales:f__:g__
MID4	denovo178	2	k__Bacteria:p__Bacteroidetes:c__[Rhodothermi]:o__[Rhodothermales]:f__Rhodothermaceae:g__Rubricoccus
MID4	denovo18	3	k__Bacteria:p__Proteobacteria:c__Gammaproteobacteria:o__Pseudomonadales:f__Pseudomonadaceae:g__Pseudomonas
MID4	denovo180	1	k__Bacteria:p__Proteobacteria:c__Alphaproteobacteria:o__Caulobacterales:f__Caulobacteraceae:g__Phenylobacterium
MID4	denovo181	1	k__Bacteria:p__Proteobacteria:c__Alphaproteobacteria:o__Rhodospirillales:f__Rhodospirillaceae:g__
MID4	denovo183	86	k__Bacteria:p__Cyanobacteria:c__Oscillatoriophycideae:o__Chroococcales:f__Xenococcaceae
MID4	denovo184	12	k__Bacteria:p__Firmicutes:c__Clostridia:o__Clostridiales:f__[Tissierellaceae]:g__Peptoniphilus
MID4	denovo185	6	k__Bacteria:p__Proteobacteria:c__Gammaproteobacteria:o__Xanthomonadales:f__Xanthomonadaceae:g__
MID4	denovo186	2	k__Bacteria:p__Proteobacteria:c__Alphaproteobacteria:o__Rhizobiales:f__Bradyrhizobiaceae:g__Balneimonas
MID4	denovo187	8	k__Bacteria:p__Firmicutes:c__Clostridia:o__Clostridiales:f__Clostridiaceae:g__
MID4	denovo190	1	k__Bacteria:p__Proteobacteria:c__Alphaproteobacteria:o__Sphingomonadales:f__Sphingomonadaceae:g__
MID4	denovo191	2	k__Bacteria:p__Firmicutes:c__Clostridia:o__Clostridiales:f__[Tissierellaceae]:g__

MID4	denovo193	2	k__Bacteria:p__Proteobacteria:c__Alphaproteobacteria:o__Caulobacterales:f__Caulobacteraceae:g__Phenylobacterium
MID4	denovo194	1	k__Bacteria:p__Firmicutes:c__Bacilli:o__Bacillales:f__Paenibacillaceae:g__
MID4	denovo202	3	k__Bacteria:p__Proteobacteria:c__Alphaproteobacteria:o__Rhizobiales:f__Hyphomicrobiaceae:g__Devosia
MID4	denovo204	1	k__Bacteria:p__Firmicutes:c__Clostridia:o__Clostridiales:f__:g__
MID4	denovo206	1	k__Bacteria:p__Acidobacteria:c__[Chloracidobacteria]:o__DS-100:f__:g__
MID4	denovo207	4	k__Bacteria:p__Actinobacteria:c__Acidimicrobiia:o__Acidimicrobiales:f__C111:g__
MID4	denovo21	1	k__Bacteria:p__Firmicutes:c__Bacilli:o__Lactobacillales:f__:g__
MID4	denovo210	4	k__Bacteria:p__Proteobacteria:c__Gammaproteobacteria:o__Xanthomonadales:f__Xanthomonadaceae:g__
MID4	denovo213	1	k__Bacteria:p__Proteobacteria:c__Gammaproteobacteria:o__Xanthomonadales:f__Xanthomonadaceae:g__Luteimonas
MID4	denovo218	4	k__Bacteria:p__Cyanobacteria:c__Oscillatoriophycideae:o__Oscillatoriales:f__Phormidiaceae:g__Phormidium
MID4	denovo219	3	k__Bacteria:p__Proteobacteria:c__Alphaproteobacteria:o__Rhodobacterales:f__Rhodobacteraceae:g__Rubellimicrobium
MID4	denovo228	1	k__Bacteria:p__Proteobacteria:c__Alphaproteobacteria:o__Sphingomonadales:f__Sphingomonadaceae:g__Sphingomonas
MID4	denovo23	1	k__Bacteria:p__Cyanobacteria:c__Oscillatoriophycideae:o__Chroococcales:f__Xenococcaceae:g__
MID4	denovo232	46	k__Bacteria:p__Firmicutes:c__Clostridia:o__Clostridiales:f__[Tissierellaceae]:g__Anaerococcus
MID4	denovo236	1	k__Bacteria:p__Actinobacteria:c__Actinobacteria:o__Actinomycetales:f__Geodermatophilaceae:g__
MID4	denovo241	1	k__Bacteria:p__Proteobacteria:c__Alphaproteobacteria:o__Rhodospirillales:f__:g__
MID4	denovo246	3	k__Bacteria:p__Acidobacteria:c__Solibacteres:o__Solibacterales:f__Solibacteraceae:g__
MID4	denovo247	1	k__Bacteria:p__Firmicutes:c__Clostridia:o__Clostridiales:f__:g__
MID4	denovo249	1	k__Bacteria:p__Proteobacteria:c__Deltaproteobacteria:o__Myxococcales:f__:g__
MID4	denovo25	2	k__Bacteria:p__Firmicutes:c__Clostridia:o__Clostridiales:f__Lachnospiraceae:g__
MID4	denovo252	5	k__Bacteria:p__Actinobacteria:c__Actinobacteria:o__Actinomycetales:f__Intrasporangiaceae:g__Kytococcus
MID4	denovo261	1	k__Bacteria:p__Actinobacteria:c__Thermoleophilia:o__Gaiellales:f__Gaiellaceae:g__
MID4	denovo269	1	k__Bacteria:p__Proteobacteria:c__Betaproteobacteria:o__:f__:g__
MID4	denovo272	8	k__Bacteria:p__Proteobacteria:c__Alphaproteobacteria:o__Rhodobacterales:f__Rhodobacteraceae:g__Rubellimicrobium
MID4	denovo274	3	k__Bacteria:p__Firmicutes:c__Clostridia:o__Clostridiales:f__Ruminococcaceae:g__Ruminococcus
MID4	denovo281	3	k__Bacteria:p__Cyanobacteria:c__Oscillatoriophycideae:o__Chroococcales:f__Xenococcaceae:g__
MID4	denovo282	1	k__Bacteria:p__Proteobacteria:c__Gammaproteobacteria:o__Xanthomonadales:f__Xanthomonadaceae:g__Stenotrophomonas
MID4	denovo284	1	k__Bacteria:p__Firmicutes:c__Bacilli:o__Lactobacillales:f__Streptococcaceae:g__Streptococcus
MID4	denovo288	4	k__Bacteria:p__Proteobacteria:c__Alphaproteobacteria:o__Caulobacterales:f__Caulobacteraceae:g__
MID4	denovo29	1	k__Bacteria:p__Firmicutes:c__Clostridia:o__Clostridiales:f__:g__
MID4	denovo294	1	k__Bacteria:p__Firmicutes:c__Clostridia:o__Clostridiales:f__[Tissierellaceae]:g__Peptoniphilus
MID4	denovo296	9	k__Bacteria:p__Firmicutes:c__Clostridia:o__Clostridiales:f__[Tissierellaceae]:g__WAL_1855D
MID4	denovo297	1	k__Bacteria:p__Proteobacteria:c__Alphaproteobacteria:o__Rhodospirillales:f__Acetobacteraceae:g__
MID4	denovo310	5	k__Bacteria:p__Cyanobacteria:c__Synechococcophycideae:o__Pseudanabaenales:f__Pseudanabaenaceae:g__Leptolyngbya
MID4	denovo316	1	k__Bacteria:p__Bacteroidetes:c__Flavobacteriia:o__Flavobacteriales:f__Flavobacteriaceae:g__Flavobacterium
MID4	denovo321	2	k__Bacteria:p__Actinobacteria:c__Actinobacteria:o__Actinomycetales:f__Micrococcaceae:g__Nesterenkonia
MID4	denovo325	4	k__Bacteria:p__Firmicutes:c__Clostridia:o__Clostridiales:f__[Tissierellaceae]:g__Anaerococcus
MID4	denovo327	2	k__Bacteria:p__Actinobacteria:c__Actinobacteria:o__Actinomycetales:f__Pseudonocardiaceae:g__Pseudonocardia
MID4	denovo329	2	k__Bacteria:p__Acidobacteria:c__Acidobacteriia:o__Acidobacteriales:f__Acidobacteriaceae:g__
MID4	denovo330	1	k__Bacteria:p__Gemmatimonadetes:c__Gemm-5:o__:f__:g__
MID4	denovo335	1	k__Bacteria:p__Actinobacteria:c__Actinobacteria:o__Actinomycetales:f__Micrococcaceae:g__Arthrobacter
MID4	denovo340	5	k__Bacteria:p__Cyanobacteria:c__Chloroplast:o__Chlorophyta:f__:g__
MID4	denovo345	1	k__Bacteria:p__Firmicutes:c__Clostridia:o__Clostridiales:f__Clostridiaceae:g__Clostridium
MID4	denovo346	1	k__Bacteria:p__Proteobacteria:c__Betaproteobacteria:o__Burkholderiales:f__Oxalobacteraceae:g__
MID4	denovo347	1	k__Bacteria:p__Cyanobacteria:c__Oscillatoriophycideae:o__Chroococcales:f__Xenococcaceae:g__
MID4	denovo349	2	k__Bacteria:p__Proteobacteria:c__Alphaproteobacteria:o__Sphingomonadales:f__Erythrobacteraceae:g__
MID4	denovo350	1	k__Bacteria:p__Firmicutes:c__Clostridia:o__Clostridiales:f__Clostridiaceae:g__Clostridium
MID4	denovo353	14	k__Bacteria:p__Proteobacteria:c__Alphaproteobacteria:o__Rhodobacterales:f__Rhodobacteraceae:g__Rubellimicrobium
MID4	denovo355	1	k__Bacteria:p__Bacteroidetes:c__Bacteroidia:o__Bacteroidales:f__Prevotellaceae:g__Prevotella
MID4	denovo357	5	k__Bacteria:p__Actinobacteria:c__Actinobacteria:o__Actinomycetales:f__Corynebacteriaceae:g__Corynebacterium
MID4	denovo370	1	k__Bacteria:p__Actinobacteria:c__Actinobacteria:o__Actinomycetales:f__Propionibacteriaceae:g__Propionibacterium
MID4	denovo371	1	k__Bacteria:p__Firmicutes:c__Clostridia:o__Clostridiales:f__:g__
MID4	denovo376	1	k__Bacteria:p__[Thermi]:c__Deinococci:o__Deinococcales:f__Deinococcaceae:g__Deinococcus
MID4	denovo377	1	k__Bacteria:p__Actinobacteria:c__Actinobacteria:o__Actinomycetales:f__Nocardioidaceae:g__Propionicimonas
MID4	denovo381	1	k__Bacteria:p__Firmicutes:c__Clostridia:o__Clostridiales:f__Clostridiaceae:g__Clostridium
MID4	denovo383	1	k__Bacteria:p__Actinobacteria:c__Thermoleophilia:o__Gaiellales:f__Gaiellaceae:g__
MID4	denovo385	3	k__Bacteria:p__Bacteroidetes:c__Bacteroidia:o__Bacteroidales:f__Bacteroidaceae:g__Bacteroides
MID4	denovo387	1	k__Bacteria:p__Proteobacteria:c__Gammaproteobacteria:o__Aeromonadales:f__Aeromonadaceae:g__
MID4	denovo390	1	k__Bacteria:p__Actinobacteria:c__Actinobacteria:o__Actinomycetales:f__Microbacteriaceae:g__Frigoribacterium
MID4	denovo393	18	k__Bacteria:p__Proteobacteria:c__Gammaproteobacteria:o__Xanthomonadales:f__Xanthomonadaceae:g__Lysobacter
MID4	denovo402	1	k__Bacteria:p__Bacteroidetes:c__Bacteroidia:o__Bacteroidales:f__S24-7:g__
MID4	denovo403	12	k__Bacteria:p__Proteobacteria:c__Gammaproteobacteria:o__Enterobacteriales:f__Enterobacteriaceae:g__Buchnera
MID4	denovo404	5	k__Bacteria:p__Firmicutes:c__Bacilli:o__Lactobacillales:f__Enterococcaceae:g__Enterococcus
MID4	denovo408	1	k__Bacteria:p__Proteobacteria:c__Gammaproteobacteria:o__Pseudomonadales:f__Moraxellaceae:g__
MID4	denovo409	1	k__Bacteria:p__Actinobacteria:c__Actinobacteria:o__Actinomycetales:f__Nakamurellaceae:g__
MID4	denovo414	1	k__Bacteria:p__Proteobacteria:c__Alphaproteobacteria:o__Caulobacterales:f__Caulobacteraceae:g__
MID4	denovo415	9	k__Bacteria:p__Cyanobacteria:c__Synechococcophycideae:o__Pseudanabaenales:f__Pseudanabaenaceae:g__Leptolyngbya

MID4	denovo427	4	k__Bacteria:p__Bacteroidetes:c__Bacteroidia:o__Bacteroidales:f__Prevotellaceae:g__Prevotella
MID4	denovo431	1	k__Bacteria:p__Proteobacteria:c__Alphaproteobacteria:o__Rhizobiales:f__Methylobacteriaceae:g__Methylobacterium
MID4	denovo432	2	k__Bacteria:p__Cyanobacteria:c__Synechococcophycideae:o__Pseudanabaenales:f__Pseudanabaenaceae:g__Leptolyngbya
MID4	denovo437	5	k__Bacteria:p__Bacteroidetes:c__Bacteroidia:o__Bacteroidales:f__Prevotellaceae:g__Prevotella
MID4	denovo440	1	k__Bacteria:p__Firmicutes:c__Clostridia:o__Clostridiales:f__Ruminococcaceae:g__Oscillospira
MID4	denovo441	1	k__Bacteria:p__Proteobacteria:c__Alphaproteobacteria:o__Rhizobiales:f__Phyllobacteriaceae:g__Aminobacter
MID4	denovo446	1	k__Bacteria:p__Proteobacteria:c__Alphaproteobacteria:o__Rhodospirillales:f__Rhodospirillaceae:g__
MID4	denovo448	4	k__Bacteria:p__Actinobacteria:c__Coriobacteriia:o__Coriobacteriales:f__Coriobacteriaceae:g__
MID4	denovo45	2	k__Bacteria:p__Actinobacteria:c__Actinobacteria:o__Actinomycetales:f__Kineosporiaceae:g__
MID4	denovo458	1	k__Bacteria:p__Actinobacteria:c__Actinobacteria:o__Actinomycetales:f__:g__
MID4	denovo470	1	k__Bacteria:p__Actinobacteria:c__Actinobacteria:o__Actinomycetales:f__:g__
MID4	denovo471	1	k__Bacteria:p__Proteobacteria:c__Alphaproteobacteria:o__Rhizobiales:f__Hyphomicrobiaceae:g__Devosia
MID4	denovo472	1	k__Bacteria:p__Bacteroidetes:c__[Rhodothermi]:o__[Rhodothermales]:f__Rhodothermaceae:g__Rubricoccus
MID4	denovo476	1	k__Bacteria:p__Firmicutes:c__Bacilli:o__Lactobacillales:f__Aerococcaceae:g__Facklamia
MID4	denovo478	1	k__Bacteria:p__Actinobacteria:c__Thermoleophilia:o__Solirubrobacterales:f__Conexibacteraceae:g__
MID4	denovo480	1	k__Bacteria:p__Firmicutes:c__Bacilli:o__Lactobacillales:f__Aerococcaceae:g__
MID4	denovo481	1	k__Bacteria:p__Bacteroidetes:c__Bacteroidia:o__Bacteroidales:f__[Paraprevotellaceae]:g__[Prevotella]
MID4	denovo484	1	k__Bacteria:p__Actinobacteria:c__Thermoleophilia:o__Solirubrobacterales:f__:g__
MID4	denovo485	1	k__Bacteria:p__Firmicutes:c__Clostridia:o__Clostridiales:f__Lachnospiraceae:g__Blautia
MID4	denovo486	1	k__Bacteria:p__Cyanobacteria:c__Chloroplast:o__Streptophyta:f__:g__
MID4	denovo487	63	k__Bacteria:p__Actinobacteria:c__Actinobacteria:o__Actinomycetales:f__Dermabacteraceae:g__Dermabacter
MID4	denovo489	1	k__Bacteria:p__Proteobacteria:c__Gammaproteobacteria:o__Pseudomonadales:f__Pseudomonadaceae:g__
MID4	denovo492	69	k__Bacteria:p__Cyanobacteria:c__Oscillatoriophycideae:o__Chroococcales:f__Xenococcaceae:g__Chroococcidiopsis
MID4	denovo494	1	k__Bacteria:p__Firmicutes:c__Clostridia:o__Clostridiales:f__Lachnospiraceae:g__Coprococcus
MID4	denovo495	1	k__Bacteria:p__Actinobacteria:c__Actinobacteria:o__Actinomycetales:f__Streptomycetaceae:g__Streptomyces
MID4	denovo498	3	k__Bacteria:p__Proteobacteria:c__Betaproteobacteria:o__Burkholderiales:f__:g__
MID4	denovo5	1	k__Bacteria:p__Proteobacteria:c__Alphaproteobacteria:o__Rhodobacterales:f__Rhodobacteraceae:g__Rubellimicrobium
MID4	denovo500	7	k__Bacteria:p__Cyanobacteria:c__Oscillatoriophycideae:o__Chroococcales:f__Xenococcaceae:g__
MID4	denovo502	1	k__Bacteria:p__Firmicutes:c__Bacilli:o__Lactobacillales:f__Leuconostocaceae:g__Leuconostoc
MID4	denovo504	1	k__Bacteria:p__Proteobacteria:c__Alphaproteobacteria:o__Rhodospirillales:f__Rhodospirillaceae:g__
MID4	denovo509	1	k__Bacteria:p__Proteobacteria:c__Alphaproteobacteria:o__Rickettsiales:f__:g__
MID4	denovo51	1	k__Bacteria:p__Nitrospirae:c__Nitrospira:o__Nitrospirales:f__Nitrospiraceae:g__Nitrospira
MID4	denovo511	6	k__Bacteria:p__Bacteroidetes:c__Flavobacteriia:o__Flavobacteriales:f__[Weeksellaceae]:g__Chryseobacterium
MID4	denovo515	2	k__Bacteria:p__Actinobacteria:c__Thermoleophilia:o__Gaiellales:f__Gaiellaceae:g__
MID4	denovo517	2	k__Bacteria:p__Proteobacteria:c__Alphaproteobacteria:o__Caulobacterales:f__Caulobacteraceae:g__
MID4	denovo520	2	k__Bacteria:p__Acidobacteria:c__[Chloracidobacteria]:o__RB41:f__Ellin6075:g__
MID4	denovo521	1	k__Bacteria:p__Proteobacteria:c__Betaproteobacteria:o__:f__:g__
MID4	denovo528	1	k__Bacteria:p__Bacteroidetes:c__Cytophagia:o__Cytophagales:f__Cytophagaceae:g__
MID4	denovo530	1	k__Bacteria:p__Firmicutes:c__Bacilli:o__Lactobacillales:f__Lactobacillaceae:g__Lactobacillus
MID4	denovo54	1	k__Bacteria:p__Planctomycetes:c__Planctomycetia:o__Planctomycetales:f__Planctomycetaceae:g__Planctomyces
MID4	denovo540	2	k__Bacteria:p__Firmicutes:c__Bacilli:o__Bacillales:f__Bacillaceae:g__Bacillus
MID4	denovo542	125	k__Bacteria:p__Firmicutes:c__Clostridia:o__Clostridiales:f__[Tissierellaceae]:g__Finegoldia
MID4	denovo550	1	k__Bacteria:p__Firmicutes:c__Clostridia:o__Clostridiales:f__:g__
MID4	denovo553	1	k__Bacteria:p__Chloroflexi:c__TK17:o__mle1-48:f__:g__
MID4	denovo555	2	k__Bacteria:p__Cyanobacteria:c__Oscillatoriophycideae:o__Chroococcales:f__Xenococcaceae:g__
MID4	denovo559	1	k__Bacteria:p__Bacteroidetes:c__Bacteroidia:o__Bacteroidales:f__p-2534-18B5:g__
MID4	denovo56	2	k__Bacteria:p__Proteobacteria:c__Alphaproteobacteria:o__Rhodobacterales:f__Rhodobacteraceae:g__Rubellimicrobium
MID4	denovo561	3	k__Bacteria:p__Firmicutes:c__Clostridia:o__Clostridiales:f__Lachnospiraceae:g__Coprococcus
MID4	denovo565	1	k__Bacteria:p__Acidobacteria:c__[Chloracidobacteria]:o__RB41:f__:g__
MID4	denovo566	3	k__Bacteria:p__Gemmatimonadetes:c__Gemmatimonadetes:o__Gemmatimonadales:f__:g__
MID4	denovo567	1	k__Bacteria:p__Proteobacteria:c__Gammaproteobacteria:o__Xanthomonadales:f__Xanthomonadaceae:g__
MID4	denovo569	1	k__Bacteria:p__[Thermi]:c__Deinococci:o__Deinococcales:f__Deinococcaceae:g__Deinococcus
MID4	denovo57	1	k__Bacteria:p__Proteobacteria:c__Alphaproteobacteria:o__Sphingomonadales:f__Sphingomonadaceae
MID4	denovo570	1	k__Bacteria:p__Proteobacteria:c__Gammaproteobacteria:o__Xanthomonadales:f__Xanthomonadaceae:g__Xanthomonas
MID4	denovo572	1	k__Bacteria:p__Bacteroidetes:c__Cytophagia:o__Cytophagales:f__Cytophagaceae:g__Hymenobacter
MID4	denovo574	1	k__Bacteria:p__Proteobacteria:c__Alphaproteobacteria:o__Rhizobiales:f__Methylocystaceae:g__
MID4	denovo586	2	k__Bacteria:p__Planctomycetes:c__Planctomycetia:o__Gemmatales:f__Isosphaeraceae:g__
MID4	denovo587	1	k__Bacteria:p__Cyanobacteria:c__Nostocophycideae
MID4	denovo59	2	k__Bacteria:p__Proteobacteria:c__Alphaproteobacteria:o__Rhodospirillales:f__Acetobacteraceae:g__
MID4	denovo592	1	k__Bacteria:p__Firmicutes:c__Clostridia:o__Clostridiales:f__Clostridiaceae:g__
MID4	denovo594	1	k__Bacteria:p__Nitrospirae:c__Nitrospira:o__Nitrospirales:f__Nitrospiraceae:g__Nitrospira
MID4	denovo595	1	k__Bacteria:p__Proteobacteria:c__Alphaproteobacteria:o__Rhodobacterales:f__Rhodobacteraceae:g__Amaricoccus
MID4	denovo600	20	k__Bacteria:p__Cyanobacteria:c__Oscillatoriophycideae:o__Oscillatoriales:f__Phormidiaceae:g__Phormidium
MID4	denovo601	1	k__Bacteria:p__Actinobacteria:c__Actinobacteria:o__Actinomycetales:f__Geodermatophilaceae:g__Modestobacter
MID4	denovo603	1	k__Bacteria:p__Actinobacteria:c__Thermoleophilia:o__Gaiellales:f__Gaiellaceae:g__
MID4	denovo604	1	k__Bacteria:p__Proteobacteria:c__Alphaproteobacteria:o__Rhizobiales:f__:g__
MID4	denovo605	4	k__Bacteria:p__Firmicutes:c__Bacilli:o__Gemellales:f__Gemellaceae:g__Gemella

MID4	denovo607	1	Unassigned
MID4	denovo608	1	k__Bacteria:p__Proteobacteria:c__Gammaproteobacteria:o__Xanthomonadales:f__Xanthomonadaceae:g__
MID4	denovo61	1	k__Bacteria:p__Actinobacteria:c__Actinobacteria:o__Actinomycetales:f__Actinomycetaceae:g__Actinomyces
MID4	denovo610	3	k__Bacteria:p__Proteobacteria:c__Alphaproteobacteria:o__Sphingomonadales:f__Sphingomonadaceae:g__Sphingomonas
MID4	denovo613	1	k__Bacteria:p__Proteobacteria:c__Alphaproteobacteria:o__Sphingomonadales:f__Sphingomonadaceae:g__Sphingomonas
MID4	denovo616	1	k__Bacteria:p__Proteobacteria:c__Alphaproteobacteria:o__Rhizobiales:f__Phyllobacteriaceae
MID4	denovo618	4	k__Bacteria:p__Proteobacteria:c__Alphaproteobacteria:o__Rhizobiales:f__Hyphomicrobiaceae:g__Rhodoplanes
MID4	denovo620	1	k__Bacteria:p__Actinobacteria:c__Actinobacteria:o__Actinomycetales:f__Dermabacteraceae:g__Dermabacter
MID4	denovo621	1	k__Bacteria:p__Proteobacteria:c__Alphaproteobacteria:o__Caulobacterales:f__Caulobacteraceae:g__
MID4	denovo624	1	k__Bacteria:p__Proteobacteria:c__Gammaproteobacteria:o__Xanthomonadales:f__Xanthomonadaceae:g__
MID4	denovo63	10	k__Bacteria:p__Firmicutes:c__Bacilli:o__Lactobacillales:f__Lactobacillaceae:g__Lactobacillus
MID4	denovo631	4	k__Bacteria:p__Firmicutes:c__Clostridia:o__Clostridiales:f__:g__
MID4	denovo639	1	k__Bacteria:p__Actinobacteria:c__Actinobacteria:o__Actinomycetales:f__Geodermatophilaceae:g__Modestobacter
MID4	denovo64	2	k__Bacteria:p__Proteobacteria:c__Betaproteobacteria:o__Burkholderiales:f__Comamonadaceae
MID4	denovo640	1	k__Bacteria:p__Firmicutes:c__Clostridia:o__Clostridiales:f__Ruminococcaceae:g__
MID4	denovo648	2	k__Bacteria:p__Proteobacteria:c__Alphaproteobacteria:o__Rhizobiales:f__Aurantimonadaceae:g__
MID4	denovo651	1	k__Bacteria:p__Firmicutes:c__Bacilli:o__Lactobacillales:f__Aerococcaceae:g__Alloiococcus
MID4	denovo654	2	k__Bacteria:p__Proteobacteria:c__Betaproteobacteria:o__Burkholderiales
MID4	denovo655	91	k__Bacteria:p__Firmicutes:c__Clostridia:o__Clostridiales:f__[Tissierellaceae]:g__Anaerococcus
MID4	denovo656	1	k__Bacteria:p__Chlorobi:c__Ignavibacteria:o__Ignavibacteriales:f__Ignavibacteriaceae:g__
MID4	denovo657	2	k__Bacteria:p__Proteobacteria:c__Alphaproteobacteria:o__Rhodospirillales:f__Acetobacteraceae:g__
MID4	denovo658	2	k__Bacteria:p__Actinobacteria:c__Actinobacteria:o__Actinomycetales:f__Pseudonocardiaceae:g__Pseudonocardia
MID4	denovo661	2	k__Bacteria:p__Actinobacteria:c__Actinobacteria:o__Actinomycetales:f__:g__
MID4	denovo662	7	k__Bacteria:p__Bacteroidetes:c__Bacteroidia:o__Bacteroidales:f__Prevotellaceae:g__Prevotella
MID4	denovo664	1	k__Bacteria:p__Cyanobacteria:c__Oscillatoriophycideae:o__Chroococcales:f__Xenococcaceae:g__
MID4	denovo666	3	k__Bacteria:p__Firmicutes:c__Bacilli:o__Lactobacillales:f__Aerococcaceae:g__Aerococcus
MID4	denovo667	1	k__Bacteria:p__Actinobacteria:c__Thermoleophilia:o__Solirubrobacterales:f__:g__
MID4	denovo668	2	k__Bacteria:p__Proteobacteria:c__Betaproteobacteria:o__Neisseriales:f__Neisseriaceae:g__Neisseria
MID4	denovo669	1	k__Bacteria:p__Firmicutes:c__Clostridia:o__Clostridiales:f__Ruminococcaceae:g__Ethanoligenens
MID4	denovo67	1	Unassigned
MID4	denovo673	1	k__Bacteria:p__WPS-2:c__:o__:f__:g__
MID4	denovo675	1	k__Bacteria:p__Bacteroidetes:c__Cytophagia:o__Cytophagales:f__Cytophagaceae:g__Spirosoma
MID4	denovo678	1	k__Bacteria:p__Firmicutes:c__Clostridia:o__Clostridiales:f__[Tissierellaceae]:g__Peptoniphilus
MID4	denovo679	2	k__Bacteria:p__Firmicutes:c__Bacilli:o__Bacillales:f__Bacillaceae:g__Bacillus
MID4	denovo688	1	k__Bacteria:p__Actinobacteria:c__Thermoleophilia:o__Gaiellales:f__Gaiellaceae:g__
MID4	denovo693	1	k__Bacteria:p__Proteobacteria:c__Alphaproteobacteria:o__Caulobacterales:f__Caulobacteraceae:g__
MID4	denovo694	1	k__Bacteria:p__Actinobacteria:c__Actinobacteria:o__Actinomycetales:f__Mycobacteriaceae:g__Mycobacterium
MID4	denovo696	4	k__Bacteria:p__Actinobacteria:c__Actinobacteria:o__Actinomycetales:f__Propionibacteriaceae:g__Propionibacterium
MID4	denovo698	3	k__Bacteria:p__Bacteroidetes:c__Bacteroidia:o__Bacteroidales:f__[Paraprevotellaceae]:g__CF231
MID4	denovo699	16	k__Bacteria:p__Proteobacteria:c__Alphaproteobacteria:o__Rhodospirillales:f__Acetobacteraceae:g__
MID4	denovo70	7	k__Bacteria:p__Cyanobacteria:c__Nostocophycideae:o__Nostocales:f__Nostocaceae:g__Cylindrospermopsis
MID4	denovo701	2	k__Bacteria:p__Proteobacteria:c__Alphaproteobacteria:o__Rhodospirillales:f__Acetobacteraceae:g__Roseomonas
MID4	denovo703	1	k__Bacteria:p__Firmicutes:c__Bacilli:o__Bacillales:f__Staphylococcaceae:g__Macrococcus
MID4	denovo706	1	k__Bacteria:p__Firmicutes:c__Clostridia:o__Clostridiales:f__Clostridiaceae:g__Clostridium
MID4	denovo709	7	k__Bacteria:p__Proteobacteria:c__Gammaproteobacteria:o__Pseudomonadales:f__Moraxellaceae:g__Enhydrobacter
MID4	denovo71	1	k__Bacteria:p__Proteobacteria:c__Alphaproteobacteria:o__Rhizobiales:f__Hyphomicrobiaceae:g__Devosia
MID4	denovo713	1	k__Bacteria:p__Bacteroidetes:c__Sphingobacteriia:o__Sphingobacteriales:f__Sphingobacteriaceae:g__Sphingobacterium
MID4	denovo714	1	k__Bacteria:p__Proteobacteria:c__Alphaproteobacteria:o__Sphingomonadales:f__Erythrobacteraceae:g__
MID4	denovo715	2	k__Bacteria:p__Firmicutes:c__Clostridia:o__Clostridiales:f__[Tissierellaceae]:g__Peptoniphilus
MID4	denovo716	1	k__Bacteria:p__Bacteroidetes:c__Cytophagia:o__Cytophagales:f__Cytophagaceae:g__Hymenobacter
MID4	denovo719	1	k__Bacteria:p__Firmicutes:c__Clostridia:o__Clostridiales:f__:g__
MID4	denovo723	1	k__Bacteria:p__Bacteroidetes:c__Cytophagia:o__Cytophagales:f__Cytophagaceae:g__Hymenobacter
MID4	denovo724	1	k__Bacteria:p__Acidobacteria:c__Solibacteres:o__Solibacterales:f__:g__
MID4	denovo725	4	k__Bacteria:p__Proteobacteria:c__Gammaproteobacteria:o__Enterobacteriales:f__Enterobacteriaceae:g__Erwinia
MID4	denovo728	2	k__Bacteria:p__Planctomycetes:c__Planctomycetia:o__Gemmatales:f__Isosphaeraceae:g__
MID4	denovo73	1	k__Bacteria:p__FBP:c__:o__:f__:g__
MID4	denovo732	1	k__Bacteria:p__Bacteroidetes:c__Cytophagia:o__Cytophagales:f__Cytophagaceae:g__
MID4	denovo734	1	k__Bacteria:p__Proteobacteria:c__Betaproteobacteria:o__Neisseriales:f__Neisseriaceae:g__Neisseria
MID4	denovo736	1	k__Bacteria:p__Firmicutes:c__Clostridia:o__Clostridiales:f__[Tissierellaceae]:g__Parvimonas
MID4	denovo739	1	k__Bacteria:p__Cyanobacteria:c__Synechococcophycideae:o__Pseudanabaenales:f__Pseudanabaenaceae:g__Leptolyngbya
MID4	denovo74	11	k__Bacteria:p__Actinobacteria:c__Actinobacteria:o__Actinomycetales:f__Corynebacteriaceae:g__Corynebacterium
MID4	denovo740	3	k__Bacteria:p__Firmicutes:c__Clostridia:o__Clostridiales:f__Lachnospiraceae:g__
MID4	denovo742	1	k__Bacteria:p__Actinobacteria:c__Actinobacteria:o__Actinomycetales:f__Frankiaceae:g__
MID4	denovo746	19	k__Bacteria:p__Proteobacteria:c__Gammaproteobacteria:o__Enterobacteriales:f__Enterobacteriaceae:g__Candidatus_Regiella
MID4	denovo747	1	k__Bacteria:p__Proteobacteria:c__Alphaproteobacteria:o__Sphingomonadales:f__Sphingomonadaceae:g__
MID4	denovo748	4	k__Bacteria:p__Proteobacteria:c__Gammaproteobacteria:o__Xanthomonadales:f__Xanthomonadaceae:g__Lysobacter
MID4	denovo749	1	k__Bacteria:p__Firmicutes:c__Clostridia:o__Clostridiales:f__:g__

MID4	denovo750	1	k__Bacteria:p__Bacteroidetes:c__Bacteroidia:o__Bacteroidales:f__Bacteroidaceae:g__Bacteroides
MID4	denovo752	1	k__Bacteria:p__Proteobacteria:c__Alphaproteobacteria:o__Sphingomonadales:f__Sphingomonadaceae:g__
MID4	denovo76	1	k__Bacteria:p__Chloroflexi:c__Ellin6529:o__:f__:g__
MID4	denovo763	1	k__Bacteria:p__Actinobacteria:c__Thermoleophilia:o__Gaiellales:f__Gaiellaceae:g__
MID4	denovo766	2	k__Bacteria:p__Actinobacteria:c__Actinobacteria:o__Actinomycetales:f__Microbacteriaceae:g__Agrococcus
MID4	denovo767	1	k__Bacteria:p__Proteobacteria:c__Betaproteobacteria:o__Methylophilales:f__Methylophilaceae:g__Methylotenera
MID4	denovo769	1	k__Bacteria:p__Acidobacteria:c__Acidobacteria-6:o__iii1-15:f__mb2424:g__
MID4	denovo770	50	k__Bacteria:p__Cyanobacteria:c__Oscillatoriophycideae:o__Chroococcales:f__Xenococcaceae:g__
MID4	denovo771	1	k__Bacteria:p__Armatimonadetes:c__Armatimonadia:o__Armatimonadales:f__Armatimonadaceae:g__
MID4	denovo776	4	k__Bacteria:p__Firmicutes:c__Clostridia:o__Clostridiales:f__Ruminococcaceae:g__
MID4	denovo777	4	k__Bacteria:p__Firmicutes:c__Clostridia:o__Clostridiales:f__Ruminococcaceae:g__
MID4	denovo783	1	k__Bacteria:p__Cyanobacteria:c__Oscillatoriophycideae:o__Oscillatoriales:f__Phormidiaceae:g__Phormidium
MID4	denovo786	3	k__Bacteria:p__Firmicutes:c__Clostridia:o__Clostridiales
MID4	denovo79	32	k__Bacteria:p__Firmicutes:c__Clostridia:o__Clostridiales:f__[Tissierellaceae]:g__Anaerococcus
MID4	denovo790	1	k__Bacteria:p__Cyanobacteria:c__Chloroplast:o__Streptophyta:f__:g__
MID4	denovo796	2	k__Bacteria:p__Proteobacteria:c__Alphaproteobacteria:o__Rhodobacterales:f__Rhodobacteraceae:g__Rubellimicrobium
MID4	denovo799	3	k__Bacteria:p__Firmicutes:c__Clostridia:o__Clostridiales:f__Lachnospiraceae:g__Coprococcus
MID4	denovo801	2	k__Bacteria:p__Proteobacteria:c__Alphaproteobacteria:o__Rhodobacterales:f__Rhodobacteraceae:g__Rubellimicrobium
MID4	denovo803	2	k__Bacteria:p__Actinobacteria:c__Actinobacteria:o__Actinomycetales:f__Sporichthyaceae:g__
MID4	denovo806	1	k__Bacteria:p__Firmicutes:c__Bacilli:o__Bacillales:f__Bacillaceae:g__Bacillus
MID4	denovo807	5	k__Bacteria:p__Firmicutes:c__Clostridia:o__Clostridiales:f__Lachnospiraceae:g__
MID4	denovo809	2	k__Bacteria:p__Planctomycetes:c__Planctomycetia:o__Planctomycetales:f__Planctomycetaceae:g__Planctomyces
MID4	denovo81	1	k__Bacteria:p__Acidobacteria:c__Acidobacteria-6:o__iii1-15:f__:g__
MID4	denovo816	2	k__Bacteria:p__Firmicutes:c__Bacilli:o__Lactobacillales:f__Carnobacteriaceae:g__Desemzia
MID4	denovo818	2	k__Bacteria:p__Proteobacteria:c__Alphaproteobacteria:o__Rhodobacterales:f__Rhodobacteraceae:g__Rubellimicrobium
MID4	denovo820	1	k__Bacteria:p__Proteobacteria:c__Gammaproteobacteria:o__Xanthomonadales:f__Xanthomonadaceae:g__
MID4	denovo821	6	k__Bacteria:p__Proteobacteria:c__Alphaproteobacteria:o__Rhizobiales:f__Methylocystaceae:g__
MID4	denovo822	2	k__Bacteria:p__Proteobacteria:c__Alphaproteobacteria:o__Rhodobacterales:f__Rhodobacteraceae:g__
MID4	denovo823	1	k__Bacteria:p__Firmicutes:c__Clostridia:o__Clostridiales:f__Clostridiaceae:g__Clostridium
MID4	denovo825	1	k__Bacteria:p__Planctomycetes:c__Planctomycetia:o__Pirellulales:f__Pirellulaceae:g__
MID4	denovo826	1	k__Bacteria:p__Proteobacteria:c__Gammaproteobacteria:o__Pseudomonadales:f__Pseudomonadaceae:g__Pseudomonas
MID4	denovo829	10	k__Bacteria:p__Firmicutes:c__Bacilli:o__Lactobacillales:f__Carnobacteriaceae:g__Carnobacterium
MID4	denovo834	3	k__Bacteria:p__Proteobacteria:c__Alphaproteobacteria:o__Sphingomonadales:f__Sphingomonadaceae:g__Sphingomonas
MID4	denovo836	6	k__Bacteria:p__Cyanobacteria:c__Oscillatoriophycideae:o__Chroococcales:f__Xenococcaceae
MID4	denovo839	1	k__Bacteria:p__Firmicutes:c__Clostridia:o__Clostridiales:f__[Tissierellaceae]:g__Peptoniphilus
MID4	denovo84	2	k__Bacteria:p__Firmicutes:c__Bacilli:o__Lactobacillales:f__Aerococcaceae:g__Alloiococcus
MID4	denovo841	1	k__Bacteria:p__Proteobacteria:c__Alphaproteobacteria:o__Rhizobiales:f__:g__
MID4	denovo843	1	k__Bacteria:p__Proteobacteria:c__Alphaproteobacteria:o__Rhizobiales:f__Xanthobacteraceae:g__Labrys
MID4	denovo844	1	k__Bacteria:p__Proteobacteria:c__Betaproteobacteria:o__Burkholderiales:f__Comamonadaceae:g__
MID4	denovo848	3	k__Bacteria:p__Cyanobacteria:c__4C0d-2:o__SM2F09:f__:g__
MID4	denovo850	1	k__Bacteria:p__Cyanobacteria:c__Oscillatoriophycideae:o__Oscillatoriales:f__Phormidiaceae:g__Phormidium
MID4	denovo853	12	k__Bacteria:p__Proteobacteria:c__Betaproteobacteria:o__Burkholderiales:f__:g__
MID4	denovo854	1	k__Bacteria:p__Cyanobacteria:c__Oscillatoriophycideae:o__Chroococcales:f__Xenococcaceae:g__
MID4	denovo856	1	k__Bacteria:p__Firmicutes:c__Clostridia:o__Clostridiales:f__[Tissierellaceae]:g__ph2
MID4	denovo857	1	k__Bacteria:p__Proteobacteria:c__Gammaproteobacteria:o__Xanthomonadales:f__Sinobacteraceae:g__
MID4	denovo862	4	k__Bacteria:p__FBP:c__:o__:f__:g__
MID4	denovo867	1	k__Bacteria:p__Proteobacteria:c__Alphaproteobacteria:o__Sphingomonadales:f__Sphingomonadaceae:g__
MID4	denovo868	4	k__Bacteria:p__Firmicutes:c__Clostridia:o__Clostridiales:f__[Tissierellaceae]:g__Anaerococcus
MID4	denovo870	2	k__Bacteria:p__Proteobacteria:c__Gammaproteobacteria:o__Pseudomonadales:f__Pseudomonadaceae:g__Pseudomonas
MID4	denovo872	1	k__Bacteria:p__Acidobacteria:c__iii1-8:o__32-20:f__:g__
MID4	denovo873	2	k__Bacteria:p__Cyanobacteria:c__Synechococcophycideae:o__Pseudanabaenales:f__Pseudanabaenaceae:g__
MID4	denovo874	1	k__Bacteria:p__Proteobacteria:c__Alphaproteobacteria:o__Rhodobacterales:f__Rhodobacteraceae:g__Amaricoccus
MID4	denovo878	4	k__Bacteria:p__Firmicutes:c__Bacilli:o__Lactobacillales:f__Streptococcaceae:g__Streptococcus
MID4	denovo88	2	k__Bacteria:p__Cyanobacteria:c__Synechococcophycideae:o__Pseudanabaenales:f__Pseudanabaenaceae:g__Leptolyngbya
MID4	denovo880	22	k__Bacteria:p__Firmicutes:c__Clostridia:o__Clostridiales:f__Ruminococcaceae:g__Ruminococcus
MID4	denovo882	2	k__Bacteria:p__Cyanobacteria:c__Synechococcophycideae:o__Pseudanabaenales:f__Pseudanabaenaceae:g__Leptolyngbya
MID4	denovo885	11	k__Bacteria:p__Firmicutes:c__Clostridia:o__Clostridiales:f__Ruminococcaceae:g__
MID4	denovo886	3	k__Bacteria:p__Firmicutes:c__Clostridia:o__Clostridiales:f__[Tissierellaceae]:g__Peptoniphilus
MID4	denovo889	5	k__Bacteria:p__Firmicutes:c__Clostridia:o__Clostridiales:f__Veillonellaceae:g__Megasphaera
MID4	denovo89	1	k__Bacteria:p__Firmicutes:c__Clostridia:o__Clostridiales:f__Lachnospiraceae:g__Coprococcus
MID4	denovo890	1	k__Bacteria:p__Bacteroidetes:c__[Rhodothermi]:o__[Rhodothermales]:f__Rhodothermaceae:g__
MID4	denovo896	9	k__Bacteria:p__Bacteroidetes:c__Bacteroidia:o__Bacteroidales:f__Prevotellaceae:g__Prevotella
MID4	denovo897	16	k__Bacteria:p__Actinobacteria:c__Actinobacteria:o__Actinomycetales:f__Corynebacteriaceae:g__Corynebacterium
MID4	denovo902	6	k__Bacteria:p__Cyanobacteria:c__Oscillatoriophycideae:o__Chroococcales:f__Xenococcaceae:g__
MID4	denovo905	1	k__Bacteria:p__Acidobacteria:c__Acidobacteria-6:o__iii1-15:f__mb2424:g__
MID4	denovo91	1	k__Bacteria:p__Proteobacteria:c__Alphaproteobacteria:o__Caulobacterales:f__Caulobacteraceae:g__Brevundimonas
MID4	denovo918	3	k__Bacteria:p__Actinobacteria:c__Actinobacteria:o__Actinomycetales:f__Microbacteriaceae:g__Rathayibacter

MID4	denovo922	1	k__Bacteria:p__Proteobacteria:c__Alphaproteobacteria:o__Rhizobiales:f__Bradyrhizobiaceae
MID4	denovo923	1	k__Bacteria:p__Firmicutes:c__Clostridia:o__Clostridiales:f__Ruminococcaceae:g__
MID4	denovo925	1	k__Bacteria:p__Firmicutes:c__Bacilli:o__Bacillales:f__Paenibacillaceae:g__Paenibacillus
MID4	denovo926	1	k__Bacteria:p__Armatimonadetes:c__Chthonomonadetes:o__Chthonomonadales:f__Chthonomonadaceae:g__
MID4	denovo928	11	k__Bacteria:p__Proteobacteria:c__Alphaproteobacteria:o__Rhodospirillales:f__Acetobacteraceae:g__
MID4	denovo930	2	k__Bacteria:p__Proteobacteria:c__Alphaproteobacteria:o__Rhodospirillales:f__Acetobacteraceae:g__
MID4	denovo933	1	k__Bacteria:p__Firmicutes:c__Bacilli:o__Bacillales:f__Paenibacillaceae:g__Paenibacillus
MID4	denovo934	1	k__Bacteria:p__Proteobacteria:c__Alphaproteobacteria:o__Rhodobacterales:f__Rhodobacteraceae:g__Rhodobacter
MID4	denovo935	1	k__Bacteria:p__Proteobacteria:c__Gammaproteobacteria:o__Xanthomonadales:f__Xanthomonadaceae:g__Thermomonas
MID4	denovo937	2	k__Bacteria:p__Actinobacteria:c__Actinobacteria:o__Actinomycetales:f__Brevibacteriaceae:g__Brevibacterium
MID4	denovo938	5	k__Bacteria:p__Actinobacteria:c__Actinobacteria:o__Actinomycetales:f__Brevibacteriaceae:g__Brevibacterium
MID4	denovo943	1	k__Bacteria:p__Proteobacteria:c__Alphaproteobacteria:o__Rhodospirillales:f__Rhodospirillaceae:g__
MID4	denovo947	3	k__Bacteria:p__Firmicutes:c__Clostridia:o__Clostridiales:f__Ruminococcaceae:g__
MID4	denovo95	2	k__Bacteria:p__Bacteroidetes:c__Flavobacteriia:o__Flavobacteriales:f__[Weeksellaceae]:g__Chryseobacterium
MID4	denovo950	1	k__Bacteria:p__Proteobacteria:c__Alphaproteobacteria:o__Rhodobacterales:f__Rhodobacteraceae:g__Rubellimicrobium
MID4	denovo953	8	k__Bacteria:p__Actinobacteria:c__Actinobacteria:o__Actinomycetales:f__Microbacteriaceae:g__Curtobacterium
MID4	denovo955	1	k__Bacteria:p__Proteobacteria:c__Alphaproteobacteria:o__Rhizobiales:f__Methylobacteriaceae:g__Methylobacterium
MID4	denovo96	12	k__Bacteria:p__Firmicutes:c__Clostridia:o__Clostridiales:f__[Tissierellaceae]:g__Anaerococcus
MID4	denovo963	3	k__Bacteria:p__Proteobacteria:c__Alphaproteobacteria:o__Sphingomonadales:f__Sphingomonadaceae:g__
MID4	denovo964	2	k__Bacteria:p__Proteobacteria:c__Gammaproteobacteria:o__Pseudomonadales:f__Moraxellaceae:g__Acinetobacter
MID4	denovo965	2	k__Bacteria:p__Firmicutes:c__Bacilli:o__Lactobacillales:f__Aerococcaceae:g__Facklamia
MID4	denovo967	1	k__Bacteria:p__Gemmatimonadetes:c__Gemmatimonadetes:o__Gemmatimonadales:f__:g__
MID4	denovo97	1	k__Bacteria:p__Gemmatimonadetes:c__Gemm-3:o__:f__:g__
MID4	denovo973	7	k__Bacteria:p__Firmicutes:c__Bacilli:o__Bacillales:f__Staphylococcaceae:g__Jeotgalicoccus
MID4	denovo981	2	k__Bacteria:p__Firmicutes:c__Bacilli:o__Lactobacillales:f__Leuconostocaceae:g__
MID4	denovo983	1	k__Bacteria:p__Proteobacteria:c__Betaproteobacteria:o__Burkholderiales:f__Burkholderiaceae:g__Burkholderia
MID4	denovo991	1	k__Bacteria:p__Proteobacteria:c__Alphaproteobacteria:o__Rhizobiales:f__Phyllobacteriaceae:g__Mesorhizobium
MID4	denovo997	1	k__Bacteria:p__Proteobacteria:c__Alphaproteobacteria:o__Sphingomonadales:f__Erythrobacteraceae:g__

Table S10.1. real node table for rOTU network (based on PhyloChip data) and visualization in Cytoscape

node_name	degree	weighted_degree	shared	genus&species
134	1	11756	CR8	Pseudomonas
158	1	12369	CR8	Pseudomonas
160	2	23826	CR8,CR5	Pseudomonas
182	1	11718	CR8	Pseudomonas
339	1	11707	UR	Pseudomonas
421	1	11633	UR	Pseudomonas
507	3	35998	CO,UR,CR5	Pseudomonas
508	1	11912	CR8	Pseudomonas
841	1	11549	CR8	Pseudomonas
1007	1	12220	CR8	Pseudomonas
1095	3	38096	CO,UR,CR5	Pseudomonas
1193	1	11637	CR5	Pseudomonas
1257	1	11740	CR8	Pseudomonas
1277	1	12278	CR8	Pseudomonas
1302	1	12212	CR8	Pseudomonas
1331	1	11775	UR	Pseudomonas
1440	2	23968	UR,CR5	Pseudomonas
1444	1	11758	CR8	Pseudomonas
1481	3	35976	CO,UR,CR8	Pseudomonas
1566	1	11458	UR	Pseudomonas
1567	1	12909	CR8	Pseudomonas
1638	3	35114	CO,UR,CR5	Pseudomonas
1650	1	12284	UR	Pseudomonas
1738	1	11618	CR8	Pseudomonas
1796	1	11889	CR8	Pseudomonas
1844	1	12972	CR8	Pseudomonas
1884	1	12643	CR8	Pseudomonas
1891	1	12019	CR8	Pseudomonas
2022	1	11733	CR8	
2058	1	11686	CR5	Pseudomonas
2281	2	24298	CR8,CR5	Pseudomonas stutzeri
3351	1	13676	UR	Acinetobacter johnsonii
3360	1	13234	UR	Acinetobacter johnsonii
3369	2	26649	CR8,CR5	Acinetobacter lwoffii
3464	1	13020	UR	Acinetobacter johnsonii
3482	1	13627	UR	Acinetobacter johnsonii
3494	1	12242	CR5	Acinetobacter lwoffii
3571	1	13256	UR	Acinetobacter johnsonii
3624	1	13699	UR	Acinetobacter johnsonii
3647	1	11952	UR	Acinetobacter schindleri
3672	1	13279	UR	Acinetobacter johnsonii
3719	2	23525	CR8,CR5	Pseudomonas
3736	2	23242	CR8,CR5	Pseudomonas
3746	1	13481	UR	Acinetobacter johnsonii
3752	1	12865	UR	Acinetobacter
3771	1	12595	UR	Acinetobacter

3774	1	11859	CR5	Acinetobacter johnsonii
3796	1	12346	UR	Acinetobacter schindleri
3871	1	13515	UR	Acinetobacter johnsonii
3925	3	37477	CO,UR,CR5	Pseudomonas mendocina
4011	1	11886	CR5	Kangiella
4039	1	13741	UR	Acinetobacter johnsonii
4055	1	13401	UR	Acinetobacter johnsonii
4078	1	12716	UR	Acinetobacter johnsonii
4093	2	24833	UR,CR5	Acinetobacter johnsonii
4102	1	13526	UR	Acinetobacter johnsonii
4273	1	12884	UR	
4421	1	11876	CR5	
4467	1	11825	CR5	
4497	1	11830	CR5	
4998	1	11749	CR5	Klebsiella
5272	2	24197	CR8,CR5	
5538	4	52738	CO,UR,CR8,CR5	
5687	1	11624	CR8	Pantoea
6094	1	11888	CR8	Pantoea
6129	1	12462	CR5	Pantoea
6203	1	11896	CR5	Citrobacter
6211	1	12825	CR5	
7636	1	12962	CR8	
7682	2	25893	CO_PMA,CR8_PMA	
7690	1	11898	CR8_PMA	Diaphorobacter
7833	1	12497	CR8	Variovorax paradoxus
7871	2	25114	UR,CR8	Diaphorobacter
7899	1	1	CO_PMA	
7917	1	12216	CO	
8038	1	12896	CR8	Diaphorobacter
8070	2	25100	CR8,CR5_PMA	Diaphorobacter
8071	2	27034	CR8,CR5	Diaphorobacter
8086	1	13833	CO_PMA	Diaphorobacter
8217	2	25228	CO_PMA,CR8_PMA	Diaphorobacter
8247	1	13453	CR8	Diaphorobacter
8249	1	12712	UR	Diaphorobacter
8300	3	39444	CO,UR,CR5	Diaphorobacter
8316	1	12351	CR8	Diaphorobacter
8368	1	12049	UR	
8385	1	13005	CO_PMA	Diaphorobacter
8437	1	12023	CR8	Diaphorobacter
8529	1	12536	CR8	Diaphorobacter
8546	2	24494	CR8,CR5_PMA	Diaphorobacter
8593	1	11565	CR8	Diaphorobacter
8663	2	23753	CO,CR8	Diaphorobacter
8808	2	23506	UR,CR8	Delftia
8842	1	11984	CR8	Diaphorobacter
8845	1	11981	CO_PMA	Diaphorobacter
8961	1	1	CR5_PMA	
8983	2	24910	UR,CR8	Diaphorobacter

9071	2	24666	CO_PMA,CR8_PMA	
9082	2	25087	CO_PMA,CR8_PMA	
9103	3	36434	CO,CO_PMA,CR8	Rhodoferax
9140	1	12197	CO	
9176	2	2	CO_PMA,CR5_PMA	
9182	1	12656	CR8	Diaphorobacter
9232	3	36787	CO,UR,CR8	Rhodoferax
9253	1	11752	CR8	Diaphorobacter
9359	1	12169	CO	Simplicispira
9380	1	12967	CO	Diaphorobacter
9418	1	13016	CO	Diaphorobacter
9425	1	12368	CR8_PMA	
9427	1	12207	CR8_PMA	Verminephrobacter eiseniae
9474	1	11626	CR8_PMA	Diaphorobacter
9571	1	12984	CR8	Diaphorobacter
9579	1	11467	CO_PMA	Diaphorobacter
9615	1	12633	CR8_PMA	Diaphorobacter
9619	2	23215	CO_PMA,CR8_PMA	Verminephrobacter eiseniae
9623	1	12772	CO	Diaphorobacter
9681	1	12275	CO	
9742	1	12943	CO	Diaphorobacter
9872	2	26180	UR,CR5	Diaphorobacter
9917	1	11769	CR8	Polaromonas
9921	1	12107	UR	Rhodoferax
9931	1	12580	UR	Diaphorobacter
9938	1	12942	CR8	Diaphorobacter
10116	1	12142	CO	
10173	2	25155	CO,CO_PMA	Diaphorobacter
10344	2	27350	CR8,CR5_PMA	Variovorax paradoxus
10362	1	12653	CR8_PMA	
10400	2	24224	CR8,CR5_PMA	Diaphorobacter
10409	1	12246	CR8	Diaphorobacter
10464	0	0	COURCR8CR5CO_PMAUR_PMACR8_PMACR5_PMA	
10596	1	12310	CO	Aquabacterium
10657	2	25385	CO,CR8	Aquabacterium
10672	4	4	CR8,CR5,CO_PMA,CR8_PMA	
10737	1	11758	CO	Methylibium
10744	4	52124	CO,UR,CR8,CR5	Aquabacterium
10760	3	36920	CO,UR,CR8	Aquabacterium
10804	2	24143	CO_PMA,CR8_PMA	Aquabacterium
10851	1	12033	CR8	Aquabacterium
10870	4	50992	CO,UR,CR8,CR5	Aquabacterium
10995	2	24075	CO,UR	Aquabacterium
11025	1	12890	UR	Aquabacterium
11137	2	25142	CO_PMA,CR8_PMA	Aquabacterium
11150	4	52387	CO,UR,CR8,CR5	Aquabacterium
11204	2	27435	CO,CR8	Aquabacterium
11208	1	12047	CO	Aquabacterium
11318	1	13008	CO	Aquabacterium
11490	1	12950	CO	Aquabacterium

11543	2	24445	UR,CR8	Aquabacterium
11612	1	12635	CR8	Aquabacterium
11636	2	25817	CO,CR8	Aquabacterium
11657	5	62261	CO,CR8,CR5,CO_PMA,CR8_PMA	Aquabacterium
11724	3	38550	CO,UR,CR5	Aquabacterium
11745	1	12263	CR8_PMA	Aquabacterium
11757	1	13437	CO_PMA	Aquabacterium
11853	2	25465	CR8_PMA,CR5_PMA	Aquabacterium
11897	3	35916	CO,UR,CR8	Aquabacterium
11929	1	11737	CO	Aquabacterium
11964	1	13913	CR8	Aquabacterium
11980	1	12000	CO	Aquabacterium
12094	4	52288	CO,CO_PMA,CR8_PMA,CR5_PMA	Aquabacterium
12146	1	12123	CR8	Aquabacterium
12175	2	24030	CO,CR8	Aquabacterium
12187	2	23723	CO,CR8	Aquabacterium
12211	2	23596	CO,UR	Aquabacterium
12217	3	39982	CO,UR,CR5	Aquabacterium
12326	6	6	UR,CR8,CR5,CO_PMA,CR8_PMA,CR5_PMA	
12333	1	11483	UR	Aquabacterium
12392	3	38325	CO,UR,CR8	Aquabacterium
12476	1	13037	CR8	Aquabacterium
12529	2	2	CO_PMA,CR5_PMA	
12530	1	12429	CR8	Aquabacterium
12654	1	12277	CR8	Aquabacterium
12745	2	24538	CO,CR8	Aquabacterium
12809	1	12727	CR8	Aquabacterium
12870	2	24968	CO,CR8	Aquabacterium
12882	1	12049	CO	Aquabacterium
12912	4	48528	CO,UR,CR8,CR5	Aquabacterium
12928	1	11758	CO	Aquabacterium
12934	1	11996	CO	Methylibium
13088	1	11709	CO_PMA	Aquabacterium
13091	2	24413	CO,UR	Methylibium
13092	2	23615	CO,UR	Aquabacterium
13093	1	11390	CR8_PMA	Aquabacterium
13106	2	23898	CO,UR	Aquabacterium
13122	1	12581	CO	Methylibium
13162	1	13191	CR8	Aquabacterium
13274	3	37348	CO,UR,CR8	Aquabacterium
13362	1	11753	CO	Aquabacterium
13400	1	12774	CR8	Aquabacterium
13402	1	13124	CR8	Aquabacterium
13681	1	11532	CR8	Aquabacterium
13763	1	12079	CR8	Aquabacterium
13769	2	22817	CO_PMA,CR8_PMA	Aquabacterium
13882	2	23790	CO,CR8	Aquabacterium
13991	1	12708	CR8	Aquabacterium
14055	2	23036	CO_PMA,CR8_PMA	Aquabacterium
14121	1	12523	CR8	Aquabacterium

14137	1	11444	CR8_PMA	Aquabacterium
14183	1	12913	CR8	Aquabacterium
14194	3	37003	CR8,CO_PMA,CR8_PMA	Aquabacterium
14199	3	37881	CO,UR,CR8	Aquabacterium
14292	1	12474	CO	Aquabacterium
14477	2	25145	UR,CR8	Pandoraea
14545	4	48100	UR,CR8,CR5,CO_PMA	Pandoraea
14597	1	13266	CR8	Massilia timonae
14599	1	13755	UR	Massilia timonae
14622	1	12390	CO	Burkholderia
14633	1	11705	UR	Oxalobacter
14641	1	12678	CR8	Massilia timonae
14678	1	11741	UR	sfA
14754	1	12870	CR8	Massilia timonae
14779	1	13275	CR8	Massilia timonae
14831	1	13172	CO	
15016	2	24065	CO,CR8	
15035	2	25554	UR,CR8	Massilia timonae
15127	1	12926	CO	
15249	1	13637	UR	Massilia timonae
15292	2	25731	UR,CR8	Burkholderia mallei
15342	2	26324	UR,CR8	Massilia timonae
15459	2	25364	CO,CR8	
15495	1	12796	UR_PMA	
15508	1	12327	CR8	
15533	1	12038	CR8	
15566	1	12049	CR8	
15570	1	11656	CR8	Massilia timonae
15581	3	38273	CO,UR,CR5	
15605	1	12317	CR8	
15621	1	12073	CO	
15647	1	11763	CR8	Massilia timonae
15679	1	11703	CO	
15685	2	27643	CR8,CR5	Massilia timonae
15719	1	12076	CO	Massilia timonae
15731	2	24220	CO,CR8	
15831	2	23825	CO,CR8	
15908	1	11656	UR	Pseudoxanthomonas
15917	1	12449	CO	Thermomonas
15920	1	12350	CO	Rhodanobacter
15941	1	12165	UR	Stenotrophomonas
15978	1	11716	CO	
15987	1	12425	CO	Thermomonas
16000	1	12480	CR8	
16030	1	11899	CR5	Lysobacter
16065	1	12179	CO	Succinivibrio
16096	1	12437	UR	
16161	2	24748	CO,UR	Xanthomonas axonopodis
16221	2	25326	CO,CR5	Lysobacter
16250	1	12327	UR	Stenotrophomonas

16340	1	12229	CO	Rhodanobacter
16347	0	0	COURCR8CR5CO_PMAUR_PMACR8_PMACR5_PMA	
16349	1	12525	CO	Luteimonas mephitis
16376	1	12836	CO	Rhodanobacter
16384	4	50057	CO,CO_PMA,CR8,CR5	
16425	2	24591	CO,UR	Xanthomonas axonopodis
16495	2	24530	CO,CR5	Lysobacter
16520	1	12313	UR	Pseudomonas geniculata
16522	1	12324	UR	Pseudomonas geniculata
16590	1	12709	UR	Pseudomonas geniculata
16687	1	13050	CO	Thauera
16717	1	11539	CR8	
16734	1	12643	CO	Lysobacter
16819	2	24195	CO,CR5	Lysobacter
16847	2	24013	CO,UR	Lysobacter
16916	2	24506	CO,UR	Xanthomonas axonopodis
16937	1	13983	CO	
16971	1	12652	UR	Stenotrophomonas
17015	1	12326	CR8	Dyella
17051	1	12867	UR	Comamonas
17229	1	12324	CR5	Stenotrophomonas
17322	1	12943	UR	Pseudomonas geniculata
17348	1	12006	UR	Xanthomonas axonopodis
17357	1	12146	CO	Rhodanobacter
17392	1	13006	CO	Dokdonella
17427	1	11815	CR5	Eubacterium rectale
17447	1	12614	CR5	Eubacterium rectale
17519	2	23725	UR,CR8	Eubacterium rectale
17661	1	11996	CR5	Eubacterium rectale
17677	1	12227	CR5	Eubacterium rectale
17726	1	12282	CR5	Eubacterium rectale
17744	1	12028	CR5	Eubacterium rectale
17765	2	26914	CR8,CR5	Eubacterium rectale
17792	1	11699	CR5	Eubacterium rectale
17828	1	12203	CR5	Eubacterium rectale
17852	2	23864	CR8,CR5	Roseburia
17893	1	12779	CR5	Eubacterium rectale
17943	1	11766	CR5	Eubacterium rectale
17964	1	12249	CR5	Eubacterium rectale
18113	1	11847	CR5	Eubacterium rectale
18137	1	11744	CR8	Eubacterium rectale
18175	1	12365	CR5	Eubacterium rectale
18222	1	12241	CR5	Eubacterium rectale
18252	1	12119	CR5	Eubacterium rectale
18254	1	12380	CR5	Eubacterium rectale
18281	1	11633	CR5	Eubacterium rectale
18761	1	11860	CR8	Ruminococcus
19470	1	12693	CR8	
19752	1	11891	CO	Lachnospira
20192	1	12194	CO	Coprococcus

20652	3	36748	CO,UR,CR5	Blautia
20782	2	23805	UR,CR8	
21155	2	23982	CO,CR5	Blautia
22140	1	12225	CR8	
22190	1	12305	CO	
22993	1	12214	CR8	Coprococcus
23140	1	11862	CO	Butyrivibrio
23182	1	12434	CO	Butyrivibrio
23349	1	12089	CO	
23897	1	12552	CO	
24119	1	12096	CO	
24363	1	12731	CR8	Coprococcus
24427	1	12205	CR5	Faecalibacterium
24447	1	13098	CO	Faecalibacterium
24455	1	11751	CO	Faecalibacterium
24509	3	38279	CO,UR,CR5	Faecalibacterium
24544	1	12347	CR8	Faecalibacterium
24600	1	11860	CO	Faecalibacterium
24602	2	24576	CO,CR8	Faecalibacterium prausnitzii
24608	1	11753	CO	Faecalibacterium
24612	1	11763	CO	
24646	1	11747	CO	Faecalibacterium
24700	3	35605	CO,UR,CR5	Faecalibacterium prausnitzii
24841	2	23811	CO,CR5	Faecalibacterium prausnitzii
24857	2	24271	CO,CR5	Faecalibacterium
24978	3	37212	CO,UR,CR5	Faecalibacterium
24986	1	12312	CR8	Faecalibacterium
24996	2	23926	CO,CR8	Faecalibacterium
25077	1	11714	CR5	Faecalibacterium prausnitzii
25078	2	24021	CO,CR8	Faecalibacterium prausnitzii
25126	1	11802	CO	Faecalibacterium
25139	3	37088	CO,UR,CR5	Faecalibacterium prausnitzii
25221	1	12482	CR8	Faecalibacterium
25273	1	11677	CO	Faecalibacterium prausnitzii
25288	1	12453	CO	Faecalibacterium prausnitzii
25291	1	11832	CO	Faecalibacterium
25319	1	11767	CO	Faecalibacterium prausnitzii
25325	1	11524	CR8	Faecalibacterium prausnitzii
25341	2	26030	CO,CR8	Faecalibacterium
25380	1	12952	CO	Faecalibacterium
25425	2	24198	UR,CR8	Faecalibacterium prausnitzii
25471	1	13159	UR	Faecalibacterium
25499	1	12043	CO	Faecalibacterium
25549	1	11785	CO	Faecalibacterium prausnitzii
25558	1	12000	CO	Faecalibacterium
25604	1	11867	CO	Faecalibacterium
25637	3	35741	CO,UR,CR5	Faecalibacterium
25654	1	11727	CR8	Faecalibacterium
25719	1	11818	CO	Faecalibacterium
25759	1	11729	CO	Faecalibacterium prausnitzii

25804	1	11964	CO	Faecalibacterium prausnitzii
25901	3	39502	CO,UR,CR5	Faecalibacterium prausnitzii
25914	1	12021	CR5	Faecalibacterium prausnitzii
25950	2	23955	CO,CR8	Faecalibacterium prausnitzii
25994	2	24890	CO,CR8	Faecalibacterium
26041	2	23567	CO,CR8	Faecalibacterium
26109	2	23753	CO,CR5	Faecalibacterium prausnitzii
26129	1	11990	CO	Faecalibacterium
26219	1	12034	CO	Faecalibacterium prausnitzii
26227	3	37286	CO,UR,CR5	Faecalibacterium
26269	1	11708	CR5	
26288	3	39653	CO,UR,CR5	Faecalibacterium prausnitzii
26356	1	11745	CO	Faecalibacterium
26371	3	36985	CO,UR,CR5	Faecalibacterium prausnitzii
26393	1	11615	CR8	Faecalibacterium prausnitzii
26430	1	11954	CO	Faecalibacterium prausnitzii
26462	2	25453	CO,CR8	Faecalibacterium prausnitzii
26514	1	11555	CR8	Faecalibacterium prausnitzii
26599	2	24228	CO,CR5	Faecalibacterium
26611	2	23554	CO,CR5	Faecalibacterium
26613	1	11866	CO	Faecalibacterium prausnitzii
26744	1	11733	CO	
26746	1	12132	CO	Ruminococcus bromii
26768	3	36254	CO,UR,CR5	Ruminococcus
26910	1	11990	CO	Ruminococcus bromii
26984	3	35344	CO,UR,CR5	
27392	2	24035	CR8,CR5	Ruminococcus
27490	1	12561	CR8	Clostridium
27536	1	12066	CR5	Ruminococcus
28064	1	12041	UR	
28113	2	25545	CO,CR8	
28159	1	12607	CR5	Ruminococcus
28504	3	38547	CO_PMA,CR8_PMA,CR5_PMA	Corynebacterium
28641	1	13073	CO_PMA	Corynebacterium
28655	1	13034	CR5	Corynebacterium
28664	2	24340	CO,CR5	Corynebacterium
28691	1	12293	CO_PMA	Corynebacterium
28754	3	40691	CO,CO_PMA,CR5	Corynebacterium
28768	1	12409	CR5	Corynebacterium
28819	1	13820	CR8	Corynebacterium
28952	1	13251	CR8	Corynebacterium
28961	2	22447	CO_PMA,CR5_PMA	Corynebacterium
28989	2	24320	CO,CR5	Corynebacterium
29039	0	0	COURCR8CR5CO_PMAUR_PMACR8_PMACR5_PMA	
29061	1	12804	UR	Corynebacterium
29382	2	2	CO_PMA,CR8_PMA	
29693	1	11892	CO_PMA	Corynebacterium
29743	1	12633	CO_PMA	Corynebacterium
29861	1	1	CO_PMA	
29901	3	37510	CO,UR,CR5	Corynebacterium

29909	2	26071	CO,CR5	Corynebacterium
30016	1	12156	CR5	Corynebacterium
30017	4	53743	CO,UR,CR8,CR5	Corynebacterium
30066	0	0	COURCR8CR5CO_PMAUR_PMACR8_PMACR5_PMA	
30142	1	11929	CO_PMA	Corynebacterium
30177	1	12916	CO_PMA	Corynebacterium
30421	1	13719	CR5	Corynebacterium
30430	2	25588	CO_PMA,CR8_PMA	Corynebacterium
30513	3	36418	CO_PMA,CR8_PMA,CR5_PMA	Corynebacterium
30645	1	12818	CR8	Corynebacterium
30652	1	13013	CO_PMA	Corynebacterium
30734	1	12555	CO_PMA	Corynebacterium
30787	6	6	UR,CR8,CR5,CO_PMA,CR8_PMA,CR5_PMA	
30849	2	25698	CO_PMA,CR5_PMA	Corynebacterium
30882	1	12450	UR	Corynebacterium
30922	2	25577	CO,CR5	Corynebacterium
30996	4	53367	CO,CO_PMA,CR8_PMA,CR5_PMA	Corynebacterium
31080	3	38006	CO,UR,CR5	Corynebacterium
31163	2	24939	CR8,CR5_PMA	Corynebacterium
31205	3	37317	CO,UR,CR5	Corynebacterium
31218	3	36560	CO_PMA,CR8_PMA,CR5_PMA	Corynebacterium
31307	1	12838	CR8_PMA	Corynebacterium
31464	4	4	CR8,CO_PMA,CR8_PMA,CR5_PMA	
31479	1	13557	CR8	Corynebacterium
31616	1	13126	UR	Corynebacterium
31655	1	13168	CR8	Corynebacterium
31702	1	12178	CO_PMA	Corynebacterium
31823	1	13325	CO_PMA	Corynebacterium
32200	5	62261	CO,CR8,CR5,CO_PMA,CR8_PMA	Corynebacterium
32344	1	13429	CO_PMA	Corynebacterium
32357	2	27220	UR,CR5	Corynebacterium
32411	1	12582	CO_PMA	Corynebacterium
32497	3	3	CO_PMA,CR8_PMA,CR5_PMA	
32607	2	26344	CR8,CR5	Corynebacterium
32857	1	13228	CR8	Corynebacterium
33058	4	53650	CO,UR,CR8,CR5	Corynebacterium
33189	1	11653	UR	Corynebacterium
33258	2	24025	CO_PMA,CR8_PMA	Corynebacterium
33300	1	12993	CR5	Corynebacterium
33488	1	14094	CR5	Corynebacterium
33570	1	11937	CR8	Corynebacterium
33576	1	13463	CR8	Corynebacterium
33646	1	12764	CR8	Corynebacterium
33688	1	13395	CR8	Corynebacterium
33700	1	13858	CO	Corynebacterium
33847	1	13074	CR8	Corynebacterium
33873	3	38827	CR8,CO_PMA,CR8_PMA	Corynebacterium
33892	1	13248	CR5	Corynebacterium
33895	1	10972	CR5_PMA	Corynebacterium
34124	1	13744	CR5	Corynebacterium

34161	3	3	CO_PMA,CR8_PMA,CR5_PMA	
34240	4	51595	CO,UR,CR8,CR5	Corynebacterium
34246	1	13293	CR8	Corynebacterium
34266	1	11950	CR8_PMA	Corynebacterium
34318	1	11700	CO_PMA	Corynebacterium
34389	1	12911	CO_PMA	Corynebacterium
34438	3	39709	CO,CO_PMA,CR5	Corynebacterium
34464	4	54584	CO,UR,CR8,CR5	Corynebacterium
34708	1	12920	CO_PMA	Corynebacterium
34741	4	49786	CO,UR,CR8,CR5	Corynebacterium
34803	1	13149	CR8	Corynebacterium
35009	1	12781	CO_PMA	Corynebacterium
35030	1	13334	CO_PMA	Corynebacterium
35042	3	3	CO_PMA,CR8_PMA,CR5_PMA	
35066	4	51697	CR8,CR5,CO_PMA,CR8_PMA	Corynebacterium
35165	1	13478	CO_PMA	Corynebacterium
35183	2	26661	CR8,CR5	Corynebacterium
35227	2	25161	CO,CR5	Corynebacterium
35235	2	27117	CO_PMA,CR8_PMA	Corynebacterium
35240	1	12368	CO_PMA	Corynebacterium
35254	2	24972	CO_PMA,CR8_PMA	Corynebacterium
35383	1	1	CO_PMA	
35512	1	11867	CO_PMA	Corynebacterium
35586	3	39516	CO,UR,CR5	Corynebacterium
35629	2	27435	UR,CR5	Corynebacterium
35716	2	25344	CR8,CR5	Corynebacterium
35819	1	13134	CO_PMA	Corynebacterium
35860	1	11402	CO_PMA	Corynebacterium
36035	2	23560	CO_PMA,CR8_PMA	Corynebacterium
36089	1	13245	CR8	Corynebacterium
36091	1	12653	CO_PMA	Corynebacterium
36137	1	13103	CO_PMA	Corynebacterium
36142	4	52660	CO,UR,CR8,CR5	Corynebacterium
36231	1	12989	CR8	Corynebacterium
36381	2	26971	UR,CR8	Corynebacterium
36510	1	12104	CO_PMA	Corynebacterium
36542	1	13663	CR5	Corynebacterium
36693	2	22863	CO_PMA,CR8_PMA	Corynebacterium
36754	1	12844	CR5	Corynebacterium
36759	1	13071	CO	Corynebacterium
36762	2	26832	UR,CR8	Corynebacterium
36763	2	26804	CO,CR5	Corynebacterium
36770	1	13549	CR5	Corynebacterium
36772	1	13104	CR8	Corynebacterium
36773	1	12416	CR8	Corynebacterium
36785	4	53260	CO,UR,CR8,CR5	Corynebacterium
36787	3	36270	CO,UR,CR5	Corynebacterium
36803	4	55215	CO,UR,CR8,CR5	Corynebacterium
36804	2	26351	CR8,CR5	Corynebacterium
36823	2	24424	CO,CR5	Corynebacterium

36834	1	12612	CR8	Corynebacterium
36837	3	38192	CO,UR,CR5	Corynebacterium
36867	3	39604	CO,UR,CR5	Corynebacterium
36869	1	12469	CR8	Corynebacterium
36880	1	12178	CR5	Corynebacterium
36895	2	26601	CR8,CR5	Corynebacterium
36899	1	12809	UR	Corynebacterium flavescens
36900	1	13361	CR8	Corynebacterium
36906	2	26445	CR8,CR5	Corynebacterium
36908	1	13104	CR5	Corynebacterium
36911	1	13247	CO	Corynebacterium
36930	2	24790	CO,CR8	Corynebacterium
36931	2	24103	CO,CR5	Corynebacterium
36933	2	27085	CO,CR5	Corynebacterium
36937	4	53580	CO,UR,CR8,CR5	Corynebacterium
36940	1	12317	CR5	Corynebacterium
36943	4	53492	CO,UR,CR8,CR5	Corynebacterium
36949	1	12791	CR5	Corynebacterium
36955	1	13365	CR5	Corynebacterium
36957	1	12861	CR8	Corynebacterium
36960	1	13304	CR8	Corynebacterium
36980	1	13797	CR5	Corynebacterium
36983	2	23677	CO,CR8	Corynebacterium
36992	1	13529	CR8	Corynebacterium
37002	1	13462	UR	Corynebacterium
37004	1	13481	CR5	Corynebacterium
37007	3	37907	CO,UR,CR5	Corynebacterium
37025	1	12507	CO	Corynebacterium
37029	1	14079	CR5	Corynebacterium
37036	1	12285	CO	Corynebacterium
37046	2	26222	CR8,CR5	Corynebacterium
37061	1	12684	CO	Corynebacterium
37071	3	39651	CO,UR,CR5	Corynebacterium
37076	2	24143	CO_PMA,CR8_PMA	Corynebacterium
37086	1	12254	CR5	Corynebacterium
37089	4	53932	CO,UR,CR8,CR5	Corynebacterium
37097	2	25964	CR8,CR5_PMA	Corynebacterium
37107	3	39220	CO,UR,CR8	Corynebacterium
37124	3	37532	CO,UR,CR8	Corynebacterium
37125	3	39444	CO,UR,CR8	Corynebacterium
37129	4	54442	CO,UR,CR8,CR5	Corynebacterium
37151	2	27471	CR8,CR5	Corynebacterium
37161	2	26572	CO,CR5	Corynebacterium
37166	3	35433	CO,UR,CR8	Corynebacterium
37170	2	25369	CR8,CR5	Corynebacterium
37172	2	24267	CR8,CR5	Corynebacterium
37178	4	51528	CO,UR,CR8,CR5	Corynebacterium
37180	1	13117	CR5	Corynebacterium
37183	2	25551	CO,CR5	Corynebacterium
37193	3	39173	CO,UR,CR8	Corynebacterium

37198	2	26366	UR,CR8	Corynebacterium
37199	2	25129	CR8,CR5	Corynebacterium
37201	1	12505	CR8	Corynebacterium
37226	4	49693	CO,UR,CR8,CR5	Corynebacterium
37232	1	13298	CR8	Corynebacterium
37247	2	25743	CR8,CR5	Corynebacterium jeikeium
37253	2	23350	CO_PMA,CR8_PMA	Corynebacterium
37255	1	13203	CR8	Corynebacterium
37259	1	12811	CR8	Corynebacterium
37264	1	11960	CR5	Corynebacterium
37268	3	35839	CO,UR,CR5	Corynebacterium
37280	2	27077	CO,CR8	Corynebacterium riegelii
37293	4	49133	CO,UR,CR8,CR5	Corynebacterium
37300	2	27391	UR,CR8	Corynebacterium
37312	2	24874	CO,UR	Corynebacterium variabile
37315	4	53592	CO,UR,CR8,CR5	Corynebacterium
37316	1	12420	UR	Corynebacterium
37325	1	13116	CO	Corynebacterium
37329	2	25406	UR,CR5	Corynebacterium
37358	1	14085	CR5	Corynebacterium
37364	1	11905	CR8	Corynebacterium
37365	2	27939	CR8,CR5	Corynebacterium
37390	1	11803	CO	Rhodococcus
37393	1	12410	CR8	Actinokineospora diospyrosa
37394	3	37327	CO,UR,CR5	
37398	2	25361	CO,CR5	Rhodococcus
37403	1	12059	CR8	Mycobacterium
37407	1	12491	CR8	Actinokineospora diospyrosa
37429	3	38776	CO,UR,CR5	
37449	1	12985	CR5	Gordonia
37494	1	13110	CR5	
37506	1	13089	CO	Rhodococcus
37536	3	36799	CO,UR,CR5	
37549	1	12052	CO	
37562	1	12139	CR8	Rhodococcus
37566	2	25278	UR,CO_PMA	
37576	3	39072	CO,UR,CR5	
37579	1	13213	CR5	
37598	1	13184	CO	Frankia
37609	1	11658	CR8	
37611	1	12146	CR8	Amycolatopsis
37619	1	13158	CR8	Actinokineospora diospyrosa
37622	1	13291	CR5	Gordonia
37630	2	23712	CO,CR8	Mycobacterium
37633	1	12562	CR5	
37653	1	12508	UR	Gordonia
37671	2	25063	CO,CR8	Rhodococcus
37679	3	36481	CO,UR,CR5	Rhodococcus
37688	3	38687	CO,UR,CR5	Mycobacterium
37691	1	12018	CR8	Rhodococcus

37726	3	41645	CO,UR,CR5	Mycobacterium
37735	3	36713	CO,UR,CR5	
37737	2	25153	CR8,CR5	Mycobacterium
37746	1	12487	CR5	Pseudonocardia
37763	1	12610	CR5	Kineococcus
37768	1	12698	CR8	Amycolatopsis
37779	3	36264	CO,UR,CR5	
37783	2	26470	CO,CR5	
37813	3	38391	CO,UR,CR5	
37814	2	24997	CO,CR5	Frankia
37820	2	25766	CO,CR5	
37833	1	11831	CR5	
37835	3	37940	CO,UR,CR5	
37838	2	25837	CO,CR5	Rhodococcus
37841	1	11991	CR8	Amycolatopsis
37860	2	23818	CR8,CR5	
37868	2	24420	CO,CR8	Rhodococcus
37877	4	54111	CO,UR,CR8,CR5	
37881	1	13071	CR5	Kineococcus
37902	1	12364	CR8	
37907	2	24276	CO,CR8	Mycobacterium
37917	1	12018	CO	Kineococcus radiotolerans
37920	2	24234	UR,CR5	
37921	3	37760	CO,UR,CR5	Rhodococcus
37924	1	11731	CR8	
37943	1	12518	CR8	Mycobacterium
37948	3	37838	CO,UR,CR5	Rhodococcus
37955	2	23474	CO,CR8	Mycobacterium
37963	1	12125	CO	
37984	2	24574	CO,CR8	Mycobacterium
37996	2	25583	CR8,CR5	Mycobacterium
38001	3	38516	CO,UR,CR8	Mycobacterium
38020	2	26963	CR8,CR5	
38072	1	12562	CO	Frankia
38083	1	12068	CO	
38094	1	12286	CR8	Amycolatopsis
38099	2	24954	CO,CR5	Rhodococcus
38101	2	26074	CO,CR5	Frankia
38103	1	12303	CR5	Gordonia amarae
38108	1	12313	CR5	Saccharopolyspora
38115	1	12462	CR8	Mycobacterium
38137	1	12146	CR8	Pseudonocardia
38164	1	13477	CO	
38165	1	11737	CR5	
38168	1	13445	CR5	Mycobacterium
38210	3	37662	CO,UR,CR5	
38238	1	11728	CO	Mycobacterium
38248	1	12888	CO	Mycobacterium
38266	1	13304	CO	Frankia
38270	1	12408	CR5	Gordonia

38272	1	12169	CR8	Rhodococcus
38281	1	12718	CR8	
38303	2	25371	UR,CR5	
38331	2	26322	UR,CR5	Nocardia
38337	1	13512	CR5	Pseudonocardia
38349	1	12678	CR8	Pseudonocardia
38371	1	12120	CR8	Mycobacterium
38393	3	38460	CO,UR,CR5	
38394	1	12461	CR5	Rhodococcus
38400	1	12315	UR	Tsukamurella paurometabola
38427	1	12989	CO	Mycobacterium
38432	3	38938	CO,UR,CR8	
38433	3	38391	CO,UR,CR5	
38438	1	12208	CR8	Mycobacterium
38444	2	23277	CO,CR8	Mycobacterium
38449	2	23808	UR,CR8	Rhodococcus
38463	1	12218	UR	Blastococcus saxobsidens
38499	1	13084	CR5	
38506	1	13262	CR8	Streptomyces
38519	1	12476	UR	
38524	2	25911	CO,CR8	Agromyces
38628	2	26194	CR8,CR5	Nocardioides
38640	2	25942	CO,CR8	
38646	3	37645	CO,UR,CR5	Nocardioides
38679	1	13169	CR5	Nocardioides
38691	1	12262	CO	Streptomyces
38721	1	12670	CO	Agromyces
38726	2	26070	CR8,CR5	Geodermatophilus
38746	1	12279	CR5	Nocardioides
38747	1	12222	CO	Streptomyces
38755	1	12545	CO	Streptomyces
38770	1	13104	UR	Microlunatus
38808	1	12074	CR8	Streptomyces
38813	1	13115	UR	Nocardioides oleivorans
38823	1	12702	CO	Streptomyces
38848	2	25258	CO,CR8	Agromyces
38907	2	26981	CO,CR5	Friedmanniella
38920	3	37554	CO,UR,CR5	Nocardioides
38958	3	39317	CO,UR,CR5	Nocardioides
38959	1	11853	CR8	Streptomyces
38996	3	39904	CO,UR,CR5	Nocardioides
39019	1	11609	CR8	
39027	2	26851	CR8,CR5	Nocardioides
39044	1	12499	UR	Actinopolymorpha
39054	1	11943	CR5	Geodermatophilus
39060	1	11871	CR8	Blastococcus aggregatus
39081	1	13791	CO	Nocardioides
39084	1	11970	CO	Streptomyces
39092	3	36923	CO,UR,CR5	Marmoricola
39094	1	11718	CO	Agromyces

39106	1	12415	UR	
39109	1	12597	CO	Streptomyces
39112	1	12098	CR8	Streptomyces
39124	4	49834	CO,UR,CR8,CR5	Blastococcus aggregatus
39208	2	25581	CR8,CR5	Geodermatophilus
39225	1	12125	CR8	Agromyces
39242	2	23991	CO,CR8	Agromyces
39248	1	12340	CR8	Streptomyces
39259	1	12830	CO	Agromyces
39321	1	11812	UR	Microlunatus phosphovorus
39343	1	13344	CR5	
39401	2	24373	CR8,CR5	Streptomyces
39415	1	12279	CR8	Actinomyces
39424	1	12729	CO	Streptomyces
39435	1	12599	CO	Streptomyces
39446	1	12880	CR8	Nocardioides
39473	1	12919	CR5	Nocardioides lentus
39484	4	49692	CO,UR,CR8,CR5	Blastococcus aggregatus
39541	1	12805	CO	Streptomyces
39549	2	27637	CR8,CR5	Streptomyces
39558	1	12953	CO	
39559	2	25579	CR8,CR5	Nocardioides
39588	2	26255	CR8,CR5	
39602	1	12378	CO	
39606	1	12298	CR8	Streptomyces
39607	1	12548	CR8	Actinomyces
39644	1	12232	CR8	
39646	1	11672	CO	Nocardioides
39650	3	40318	CO,UR,CR5	Streptomyces
39654	3	38645	CO,UR,CR5	Marmoricola
39661	2	24852	CO,CR8	
39676	1	11957	UR	Aeromicrobium
39684	1	12217	UR	Aeromicrobium
39719	2	25997	CO,CR5	Friedmanniella
39733	3	39089	CO,CO_PMA,CR8	Agromyces
39746	2	24431	CO,CR8	Streptomyces
39774	1	13430	CR5	Friedmanniella
39802	1	12692	CO	Streptomyces
39809	1	12091	UR	Aeromicrobium
39819	1	11771	CO	Streptomyces
39833	1	13070	CR5	
39857	1	12714	CO	
39869	1	11625	UR	Aeromicrobium
39909	1	12302	CR8	Streptomyces
39910	2	26548	CO,CR8	
39926	2	25257	CR8,CR5	Geodermatophilus
39936	1	11742	UR	
39946	1	13078	CO	Micrococcus luteus
39968	1	12773	CO	Propionicimonas
39989	1	12829	CO	Friedmanniella

40014	2	24087	CO,CR8	Agromyces
40039	1	12818	CO	
40042	3	39855	CO,UR,CR5	Nocardioides
40049	1	11972	CO	Streptomyces
40055	2	24039	CR8,CR5	Geodermatophilus
40067	1	12822	CO	Agromyces
40076	2	25530	CR8,CR5	Geodermatophilus
40101	1	12776	CR5	
40117	2	24606	CO,CR5	
40126	1	12587	CR8	Streptomyces
40141	1	12935	CO	Kocuria
40145	1	13128	CR5	Kocuria marina
40148	3	39164	CO,UR,CR5	
40152	2	25604	CO,CR5	Arthrobacter
40156	2	24935	CR8,CR5	Dermabacter hominis
40158	4	56685	UR,CR8,CR5,CO_PMA	Propionibacterium acnes
40159	3	40070	CO,CO_PMA,CR5	Propionibacterium acnes
40162	1	12063	CO_PMA	Propionibacterium acnes
40164	1	11679	CR8	Actinomyces oris
40170	2	26993	CR8,CR5	Dermabacter hominis
40171	2	27486	CO,CR5	Propionibacterium
40172	4	50140	CO,UR,CR8,CR5	Arthrobacter
40175	1	13012	CO	Arthrobacter
40178	2	26320	CO,UR	Micrococcus luteus
40182	2	23170	CO_PMA,CR5_PMA	Propionibacterium acnes
40188	1	12255	CO	Propionibacterium
40191	3	39049	CO,UR,CR5	Arthrobacter
40193	3	39555	CO,UR,CR5	Propionibacterium
40199	3	37478	CO,UR,CR5	Arthrobacter
40202	2	26265	CO,UR	Micrococcus luteus
40206	3	36798	CO,UR,CR5	Arthrobacter
40209	1	12860	CR8	Arthrobacter
40210	3	41037	CO,UR,CR5	Propionibacterium
40216	1	14071	CR5	Arthrobacter
40219	2	26598	CO,UR	Micrococcus luteus
40220	2	25655	CR8,CR5	Dermabacter hominis
40222	2	25169	CO,UR	Micrococcus luteus
40224	1	13064	CR5	Arthrobacter
40234	1	12052	CO	Arthrobacter
40235	2	27118	CO,CR5	Kocuria marina
40238	2	25025	CO,UR	Micrococcus luteus
40241	4	50977	CO,UR,CR8,CR5	Propionibacterium granulosum
40245	4	53449	CO,UR,CR8,CR5	Arthrobacter
40262	3	38625	CO,UR,CR5	Arthrobacter
40263	1	11933	UR	Micrococcus
40264	3	41475	UR,CO_PMA,CR8	Propionibacterium acnes
40278	2	24397	CO,CR8	Kytococcus
40286	2	24093	UR,CR8	Actinomyces oris
40289	2	26010	CO,UR	Micrococcus luteus
40303	3	39595	CO,UR,CR5	Arthrobacter

40304	1	13035	CR5	Arthrobacter
40314	2	26363	CO,UR	Micrococcus luteus
40322	1	11898	UR	Micrococcus luteus
40325	2	24835	CO,UR	Micrococcus luteus
40333	2	28129	CR8,CR5	Propionibacterium
40334	4	49849	CO,UR,CR8,CR5	Actinomyces oris
40335	2	25315	CO,CR8	
40337	1	12401	CR5	Arthrobacter
40340	2	26273	CO,CR5	Arthrobacter
40347	2	26608	CO,UR	Micrococcus luteus
40352	1	13425	CO	Tessaracoccus
40356	2	27406	CR8,CR5	Propionibacterium acnes
40358	1	12899	CR8	Arthrobacter
40373	2	26185	CO,UR	Micrococcus luteus
40375	1	12426	CO	Georgenia
40376	1	12403	UR	Micrococcus
40378	1	12596	CR8	Dermabacter hominis
40382	3	36670	CO,UR,CR5	Arthrobacter
40389	2	26180	UR,CR5	Dermabacter
40391	1	12807	UR	Dermabacter
40394	2	28624	CR8,CR5	Arthrobacter
40399	2	25372	CO,UR	Micrococcus luteus
40402	1	13394	CR5	Dermabacter
40407	2	23852	UR,CR8	Actinomyces
40408	3	37127	CO,UR,CR8	Kytococcus
40412	2	24332	CO,UR	
40413	1	12134	CR8	Arthrobacter
40415	1	12432	CR5	Arthrobacter
40416	1	12230	CR5	Arthrobacter
40419	1	12157	CR8	
40425	1	12900	CO	Arthrobacter
40426	3	36880	CO,UR,CR8	Kytococcus schroeteri
40427	1	12620	CR5	Arthrobacter
40436	2	26586	CO,UR	Micrococcus luteus
40442	1	12854	CO	Actinomyces
40443	2	23430	UR,CR8	Actinomyces
40455	2	25742	CO,CR5	Arthrobacter
40473	1	13405	UR	Micrococcus
40474	2	26690	CO,CR5	Arthrobacter
40476	1	12173	UR	Micrococcus luteus
40479	2	26501	CO,UR	Micrococcus luteus
40484	2	25579	CO,UR	Micrococcus luteus
40486	1	12465	CO	Arthrobacter
40488	3	37993	CO,UR,CR5	Arthrobacter
40490	1	12449	CO	Micrococcus luteus
40492	2	23758	UR,CR8	Actinomyces
40495	3	37469	CO,UR,CR5	Arthrobacter
40503	1	11724	CR8	Arthrobacter
40505	3	39085	CO,UR,CR5	
40510	2	26091	CO,UR	Micrococcus luteus

40512	3	39297	CO,UR,CR5	Arthrobacter
40518	2	28163	CR8,CR5	Propionibacterium
40522	3	37398	CO,UR,CR5	Arthrobacter
40526	1	12725	CR5	Arthrobacter
40529	1	13716	CR5	Dermabacter
40531	1	13710	CR5	Dermabacter
40535	2	25045	CO,CR5	Arthrobacter
40549	2	23789	CO,CR8	Actinomyces
40550	2	25572	UR,CO_PMA	Propionibacterium acnes
40558	2	24693	CO,CR5	Arthrobacter
40568	1	12966	CO	Arthrobacter
40574	1	12647	UR	Micrococcus luteus
40575	2	26258	CO,UR	Micrococcus luteus
40577	2	25445	UR,CR5	Arthrobacter
40578	1	13781	CR5	Knoellia subterranea
40583	1	12401	CR5	Arthrobacter
40586	2	26761	CO,CR5	Arthrobacter
40587	1	12452	CR5	Arthrobacter
40592	1	11969	CR8	
40597	2	26209	CO,CR8	Propionibacterium
40610	2	26475	CO,CR5	Arthrobacter
40611	1	13356	CO	Cellulomonas
40619	1	12366	CO	Cellulomonas
40620	1	12702	CR8	Arthrobacter
40621	2	25604	CO,UR	Micrococcus luteus
40629	1	13281	CO	
40639	1	12950	CO	Arthrobacter
40640	2	25496	CO,UR	Micrococcus luteus
40651	2	23457	UR,CR8	Actinomyces oris
40666	2	25510	CO,CR5	Arthrobacter
40676	2	25792	CR8,CR5	Arthrobacter
40678	2	25018	CO,CR8	Arthrobacter
40679	1	12962	UR	Micrococcus
40685	1	13214	CO_PMA	Propionibacterium
40686	4	54588	CO,UR,CR8,CR5	Propionibacterium acnes
40696	2	25301	CO,UR	Micrococcus luteus
40697	2	25386	CO,UR	Micrococcus luteus
40705	1	12571	CR5	Knoellia subterranea
40716	3	39471	CO,UR,CR5	
40718	2	26834	CO,UR	Micrococcus luteus
40719	1	13209	CR8	Brevibacterium
40723	1	13523	CR5	Arthrobacter
40727	2	26216	CO,CR5	Kocuria marina
40733	2	24355	UR,CR8	Actinomyces
40740	2	26280	CO,UR	Micrococcus luteus
40741	2	25710	CO,CR8	Arthrobacter
40743	1	12303	CO	Propionibacterium
40749	3	39437	CO,UR,CR5	
40753	2	24989	CO,UR	Micrococcus luteus
40757	1	13000	CO	Micrococcus luteus

40766	1	13966	CR5	Propionibacterium
40774	1	11831	CR8	Actinomyces
40778	2	26506	CO,CR5	Arthrobacter
40779	2	25447	UR,CR8	Actinomyces oris
40792	1	13220	CR8	Brevibacterium
40797	1	11801	CR8	Arthrobacter
40805	2	25163	CO,CR8	Arthrobacter
40819	1	12356	UR	Micrococcus
40827	3	35803	CO,UR,CR5	Arthrobacter
40834	1	12603	CO	Micrococcus luteus
40838	1	11831	CR8	Actinomyces
40846	1	12870	CO	Kocuria marina
40853	1	13538	CR8	Propionibacterium
40856	1	12192	UR	Janibacter
40861	2	25393	CO,UR	Micrococcus luteus
40867	1	12832	CR5	Arthrobacter
40875	3	37417	CO,UR,CR5	Arthrobacter
40876	1	13194	CR5	Arthrobacter
40882	2	24654	UR,CR8	Brevibacterium
40884	2	25558	CO,UR	Micrococcus luteus
40889	1	12775	UR	Micrococcus
40890	1	12212	CO	Georgenia
40892	2	24360	UR,CR8	Actinomyces
40893	2	27069	CR8,CR5	Dermabacter hominis
40898	3	39470	CO,UR,CR5	Arthrobacter
40899	1	12675	UR	Dermabacter
40908	1	13753	CR5	Dermabacter
40911	2	25877	CO,UR	Micrococcus luteus
40912	3	38764	CO,UR,CR5	Propionibacterium
40917	1	13240	CO	Cellulomonas
40919	2	24950	CO,CR8	Arthrobacter
40920	2	23929	CR8,CR5	Arthrobacter agilis
40921	1	12976	CO	
40925	2	25215	CO,CO_PMA	Cellulomonas
40929	1	12289	CR8	Actinomyces
40930	1	11866	UR	Arthrobacter
40932	4	55649	CO,UR,CR8,CR5	Propionibacterium acnes
40943	1	13117	UR	Micrococcus
40945	2	24732	UR,CR5	Arthrobacter
40958	2	23894	UR,CR8	Actinomyces
40960	2	25144	CO,UR	Micrococcus luteus
40962	1	13163	CO	Arthrobacter
40968	4	50870	CO,UR,CR8,CR5	Arthrobacter
40976	2	27008	CO,CR5	
40979	1	12814	CR5	Arthrobacter
40983	1	12702	CR8	Brevibacterium paucivorans
40984	3	36659	CO,UR,CR5	
40988	1	13545	CO	
40992	2	25829	CO,UR	Micrococcus luteus
40996	1	13785	CR5	Dermabacter

41001	4	48981	CO,UR,CR8,CR5	
41006	4	53548	CO,UR,CR8,CR5	Propionibacterium acnes
41008	4	50100	CO,UR,CR8,CR5	Arthrobacter
41012	2	26611	CR8,CR5_PMA	Propionibacterium acnes
41013	1	12517	CO	Arthrobacter
41015	1	11989	CO	
41016	3	37674	CO,UR,CR8	Kytococcus
41018	2	26512	UR,CR5	Dermabacter
41022	2	24052	CO,CR5	Arthrobacter
41035	3	37897	CO,UR,CR5	Arthrobacter
41048	2	25593	CO,CR8	Arthrobacter
41049	2	25759	CO,CR5	Arthrobacter
41050	2	24937	CO,CR8	Arthrobacter
41054	2	26693	CO,UR	Micrococcus luteus
41058	2	25886	CO,CR8	Arthrobacter
41060	1	13048	CO	
41061	2	26828	CO,UR	Micrococcus luteus
41068	4	53541	CO,UR,CR8,CR5	Propionibacterium
41078	1	12782	CO	
41079	2	25042	CO,CR8	Arthrobacter
41080	4	52696	CO,UR,CR8,CR5	Arthrobacter
41085	1	13844	CR8	Propionibacterium
41088	4	49209	CO,UR,CR8,CR5	Microbacterium
41101	2	25333	CO,CR8	Microbacterium
41103	1	11989	CR8	Nocardiopsis
41105	2	24820	CO,CR5	Actinoplanes
41110	1	12431	CO	Actinoplanes
41114	3	37570	CO,UR,CR5	sfA
41119	1	11719	CR8	sfA
41123	2	26068	CO,UR	Microbacterium
41163	1	11808	CO	Microbacterium
41171	1	12264	CR8	Microbacterium
41194	1	12667	UR	Actinomadura
41199	2	25127	CO,CR5	Actinoplanes
41204	1	13300	CR5	
41209	1	12613	CO	
41227	4	50880	CO,UR,CR8,CR5	sfA
41235	2	26350	CO,CR5	sfA
41237	1	11907	CR5	Actinoplanes
41250	1	13077	CO	
41253	1	12238	CR5	
41272	1	12436	CR5	
41275	2	25569	CO,CR8	Microbacterium
41284	4	49560	CO,UR,CR8,CR5	Microbacterium
41285	3	39346	CO,UR,CR5	sfA
41288	1	12791	CO	
41293	3	39728	CO,UR,CR5	Microbacterium
41301	1	12540	CO	sfA
41305	2	25430	CO,CR8	sfA
41332	1	11947	UR	Actinomadura

41368	1	12732	CR5	
41369	1	12796	CO	Agrococcus jenensis
41371	2	24593	CO,CR5	Actinoplanes
41373	4	48943	CO,UR,CR8,CR5	Microbacterium
41399	1	13271	CO	Leucobacter
41425	1	12061	CR8	sfB
41428	1	11631	UR	Actinomadura
41444	1	11725	CO	
41456	1	12858	UR	Microbacterium
41460	1	12261	CR5	Microbacterium
41462	2	24454	UR,CR8	Microbacterium
41474	2	24409	CR8,CR5	Microbacterium
41480	1	12687	CO	
41489	1	11768	CR5	Actinoplanes
41490	1	13091	CR8	Nocardiopsis
41491	3	36280	CO,UR,CR5	Microbacterium
41493	2	23786	CR8,CR5	Microbacterium
41499	2	23808	UR,CR8	Microbacterium
41500	1	13258	CR8	
41502	1	12580	CR8	Dactylosporangium
41517	1	12081	CR8	Microbacterium
41531	1	13008	CR8	Nocardiopsis
41535	2	25212	CO,CR8	Microbacterium
41542	1	11896	UR	Actinoallomurus spadix
41546	3	41784	CO,UR,CR5	Microbacterium
41552	1	12088	CO	Actinocorallia
41578	1	12204	CO	Microbacterium
41589	1	12010	UR	Actinomadura
41601	1	11778	CR8	Microbacterium
41631	4	51093	CO,UR,CR8,CR5	
41636	2	24319	CO,CR5	Actinoplanes
41637	1	12381	CO	Microbacterium
41639	3	36873	CO,UR,CR5	sfA
41652	1	12391	CR8	Nocardiopsis
41657	3	38673	CO,UR,CR5	Microbacterium
41712	1	12416	CR8	Microbacterium
41721	2	25540	CR8,CR5	Microbacterium
41730	1	12500	CO	
41733	1	11918	CR8	Nocardiopsis
41745	4	50943	CO,UR,CR8,CR5	
41749	3	35053	CO,UR,CR5	Microbacterium
41753	2	25842	CR8,CR5	Microbacterium
41755	2	23945	CO,CR8	sfA
41760	1	13196	CO	Leucobacter
41770	1	12958	CR5	
41779	1	12176	CO	
41840	4	49763	CO,UR,CR8,CR5	Microbacterium
41851	1	12798	CR8	
41853	1	11612	CR8	Microbacterium
41854	4	48057	CO,UR,CR8,CR5	Microbacterium

41868	1	12853	CO	
41881	4	50219	CO,UR,CR8,CR5	Microbacterium
41886	4	49183	CO,UR,CR8,CR5	sfD
41900	1	12147	CR5	
41904	4	52934	CO,UR,CR8,CR5	Microbacterium
41908	4	51074	CO,UR,CR8,CR5	Microbacterium
41923	3	40277	CO,UR,CR5	Arthrobacter
41925	1	12951	CO	
41931	1	13177	CO	
41938	3	36542	CO,UR,CR8	Leucobacter
41946	3	37975	CO,UR,CR5	Microbacterium
41964	1	11688	UR	Microbacterium aurum
41978	1	12935	CR8	Agreia
41992	1	12070	CO	sfB
42004	1	13007	CR5	
42038	2	26417	CR8,CR5	Microbacterium
42065	3	36474	CO,UR,CR8	Microbacterium
42075	3	37179	CO,UR,CR5	Dactylosporangium
42081	3	37615	CO,UR,CR5	Nocardiopsis
42091	1	13690	CO	Mycetocola reblochoni
42095	1	12648	CO	
42097	1	12149	CR8	
42104	4	48890	CO,UR,CR8,CR5	Microbacterium
42129	1	12397	UR	Actinomadura
42131	3	38295	CO,UR,CR5	
42149	3	39514	CO,UR,CR5	Microbacterium
42150	1	11713	CO	
42173	1	13075	CR8	Microbacterium
42189	2	23749	CO,CR8	sfD
42208	1	11811	CR8	Agreia
42218	1	12273	CR8	Nocardiopsis
42236	3	37582	CO,UR,CR5	Microbacterium
42247	1	12505	CO	Actinocorallia
42253	1	11567	CR8	Solirubrobacter
42285	1	12062	UR	Actinoallomurus spadix
42295	3	38123	CO,UR,CR5	Microbacterium
42310	1	11820	UR	Microbacterium aurum
42337	1	12576	CR5	Rathayibacter caricis
42357	1	12667	CR5	
42358	1	12512	CO	
42400	1	12024	CO	Nonomuraea
42417	1	12245	CR8	Microbacterium
42425	1	12052	CO	Dactylosporangium
42426	1	11742	CR8	Solirubrobacter
42432	2	24563	CO,CR8	Microbacterium
42436	1	12483	CO	
42470	1	12082	CR8	Solibacillus
42472	1	11877	CR8	Bacillus psychrodurans
42479	4	50708	CO,UR,CR8,CR5	
42497	1	11753	CR8	

42505	2	24313	CO,CR8	
42511	1	14498	UR	Enterococcus
42514	1	11721	UR	Bacillus
42582	1	11794	CR8	
42609	1	12178	UR	Bacillus
42616	1	12325	UR	Enterococcus
42622	1	12064	CR8	
42672	1	11761	UR	Bacillus
42676	1	13089	CR8	
42684	1	11540	UR	Bacillus
42694	1	13307	CR5	
42706	1	11683	UR	
42743	1	12203	CO	Tetragenococcus
42748	1	11914	UR	Bacillus
42814	1	12379	CO	Lactococcus lactis
42818	2	26445	CR8,CR5	
42889	1	11841	UR	Enterococcus
42908	1	11595	CR8	Lactococcus lactis
42933	1	12585	UR	Bacillus
42959	4	52499	CO,UR,CR8,CR5	
42997	1	11648	CR8	
43095	1	13271	CR8	Jeotgalicoccus
43227	2	25484	CO,CR8	
43361	1	11661	UR	Bacillus
43416	1	12642	CR5	
43461	1	11885	UR	Bacillus
43496	1	12012	UR	Bacillus
43507	1	11565	UR	Enterococcus
43571	1	12476	UR	Bacillus
43697	2	23293	CR8,CR5	Lactococcus lactis
43705	2	23560	CO,CR8	Bacillus
43709	1	11820	UR	Bacillus anthracis
43740	1	11721	UR	Bacillus
43749	1	13210	CR5	Jeotgalicoccus
43752	1	12456	CR5	Solibacillus
43755	1	11959	UR	Bacillus
43780	2	25765	CO,CR8	Gemella
43927	1	11774	CR5	
43975	1	12216	CO	Enterococcus
43982	1	12136	UR	Enterococcus
44033	1	13139	CR8	Jeotgalicoccus
44057	1	11681	UR	Enterococcus
44069	1	11658	UR	Bacillus
44151	2	24391	CR8,CR5	
44153	2	24553	CO,UR	Streptococcus pseudopneumoniae
44160	3	37119	CO,UR,CR5	Streptococcus
44162	1	12676	CR8	Streptococcus
44165	1	12272	CR8	Streptococcus
44167	2	23928	CO,CR5	Streptococcus
44182	2	24302	CO,CR8	Streptococcus

44212	4	50994	CO,UR,CR8,CR5	Streptococcus
44221	2	24872	CR8,CR5	Streptococcus
44224	2	24872	CO,UR	Streptococcus pseudopneumoniae
44235	1	12152	CO	Clostridium
44243	2	24568	CO,UR	Streptococcus pseudopneumoniae
44257	3	36687	CO,UR,CR5	Streptococcus
44277	3	37127	CO,UR,CR8	Streptococcus
44280	2	24134	CO,CR8	Streptococcus
44288	1	12547	CR8	Streptococcus thermophilus
44299	4	50198	CO,UR,CR8,CR5	Streptococcus
44308	2	24069	CO,CR8	Streptococcus
44316	2	24533	CO,CR8	Streptococcus
44346	4	49065	CO,UR,CR8,CR5	Streptococcus
44363	3	36625	CO,UR,CR5	Streptococcus
44371	1	12325	UR	Streptococcus sanguinis
44380	1	12471	UR	Streptococcus
44383	1	12225	CO	Streptococcus pseudopneumoniae
44396	1	11768	CR8	Streptococcus
44422	2	23848	CO,UR	Streptococcus pseudopneumoniae
44432	2	23637	CO,UR	Streptococcus oralis
44452	3	36406	CO,UR,CR5	Streptococcus
44460	1	12592	CR8	Streptococcus thermophilus
44466	1	12233	CR8	Streptococcus
44482	2	23985	CO,UR	Streptococcus pseudopneumoniae
44501	3	36162	CO,UR,CR5	Streptococcus
44511	2	24908	CO,CR8	Streptococcus
44516	2	23957	CO,CR8	Streptococcus
44533	2	23993	CO,CR8	Streptococcus
44534	2	24115	CO,UR	Streptococcus oralis
44552	2	24435	CO,CR8	Streptococcus
44553	1	11846	CO	Streptococcus
44554	2	24097	CO,CR5	Streptococcus
44562	2	24276	CO,UR	Streptococcus oralis
44565	4	49443	CO,UR,CR8,CR5	Streptococcus
44574	3	35490	CO,UR,CR5	Streptococcus
44590	2	24516	CO,CR8	Streptococcus
44602	1	12314	CR8	Streptococcus thermophilus
44604	2	24615	CO,CR8	Streptococcus
44608	2	24139	CO,CR8	Streptococcus
44612	1	12918	CR8	Streptococcus thermophilus
44623	2	23406	UR,CR8	Streptococcus
44633	3	35908	CO,UR,CR5	Streptococcus
44644	1	11997	CO	Streptococcus
44647	4	48523	CO,UR,CR8,CR5	Streptococcus
44651	2	23437	CO,CR8	Streptococcus
44655	1	12436	CR8	Streptococcus thermophilus
44666	1	11943	CR5	Streptococcus infantis
44673	4	48035	CO,UR,CR8,CR5	Streptococcus
44677	1	11600	UR	Streptococcus pseudopneumoniae
44680	2	23990	UR,CR8	Streptococcus

44681	3	36198	CO,UR,CR8	Streptococcus
44688	2	23361	UR,CR8	Streptococcus
44702	2	23263	CO,UR	Clostridium
44703	3	37131	CO,UR,CR5	Streptococcus
44721	1	13272	CR5	Streptococcus
44743	2	24242	CO,UR	Streptococcus pseudopneumoniae
44744	3	35129	CO,UR,CR5	Clostridium
44745	2	24582	CO,UR	Streptococcus oralis
44751	4	48731	CO,UR,CR8,CR5	Streptococcus
44757	1	12376	CO	Streptococcus
44767	2	24167	CO,CR8	Streptococcus
44770	4	47856	CO,UR,CR8,CR5	Streptococcus
44778	2	24178	CO,CR8	Streptococcus
44789	2	24606	CO,UR	Streptococcus pseudopneumoniae
44792	4	49537	CO,UR,CR8,CR5	Streptococcus
44813	1	12123	CR8	Streptococcus
44816	4	49252	CO,UR,CR8,CR5	Streptococcus
44820	2	23494	UR,CR8	Streptococcus
44823	4	49140	CO,UR,CR8,CR5	Streptococcus
44826	3	36054	CO,UR,CR5	Streptococcus
44829	3	35381	CO,UR,CR5	Streptococcus
44837	1	12122	CR8	Streptococcus
44852	1	12245	CR8	Streptococcus
44860	4	49499	CO,UR,CR8,CR5	Streptococcus
44884	1	12309	CR5	Streptococcus infantis
44896	4	50288	CO,UR,CR8,CR5	Streptococcus
44904	1	11728	CR5	Streptococcus
44910	4	48301	CO,UR,CR8,CR5	Streptococcus
44911	3	36662	CO,UR,CR5	Streptococcus
44912	2	23895	CO,CR8	Streptococcus
44928	1	12274	CO	Streptococcus pseudopneumoniae
44957	2	24450	CR8,CR5	Streptococcus
44958	3	36063	CO,UR,CR5	Streptococcus
44969	2	24790	CO,CR8	Streptococcus
44976	1	12207	UR	Streptococcus
44997	1	12458	CO	Streptococcus pseudopneumoniae
45005	3	36227	CO,UR,CR5	Streptococcus
45008	2	24403	CO,UR	Streptococcus pseudopneumoniae
45015	1	11970	CO	Streptococcus
45016	4	47486	CO,UR,CR8,CR5	Streptococcus
45052	2	25174	CO,UR	Streptococcus pseudopneumoniae
45053	2	24150	CO,CR8	Streptococcus
45070	1	12307	CR8	Streptococcus thermophilus
45084	1	12529	CR8	Streptococcus thermophilus
45097	1	11617	CR8	Streptococcus
45101	1	12310	CR8	Streptococcus thermophilus
45105	3	35875	CO,UR,CR5	Streptococcus
45111	2	23620	CR8,CR5	Streptococcus
45118	4	48403	CO,UR,CR8,CR5	Streptococcus
45130	3	37574	CO,UR,CR5	Streptococcus

45131	4	48262	CO,UR,CR8,CR5	Streptococcus
45151	1	11828	UR	Streptococcus pseudopneumoniae
45158	4	48893	CO,UR,CR8,CR5	Streptococcus
45161	4	51537	CO,UR,CR8,CR5	Streptococcus
45167	2	24572	CO,CR8	Streptococcus
45179	1	12247	CR8	Streptococcus
45188	2	25440	CR8,CR5	Streptococcus
45210	3	37018	CO,UR,CR8	Streptococcus
45221	1	11687	CR8	Streptococcus
45225	1	12400	CR8	Streptococcus thermophilus
45228	2	24312	CO,CR8	Streptococcus
45230	3	36361	CO,UR,CR8	Streptococcus
45238	2	23981	CO,CR8	Streptococcus
45246	1	11641	CR8	Streptococcus
45269	2	24630	CO,UR	Streptococcus pseudopneumoniae
45286	2	24529	CO,UR	Streptococcus pseudopneumoniae
45297	3	36931	CO,UR,CR5	Streptococcus
45299	2	24408	CO,UR	Streptococcus pseudopneumoniae
45300	4	48331	CO,UR,CR8,CR5	Streptococcus
45304	2	24532	CO,UR	Streptococcus pseudopneumoniae
45307	1	12262	CR8	Streptococcus thermophilus
45308	2	24357	CO,CR8	Streptococcus
45311	3	37678	CO,UR,CR5	Streptococcus
45320	4	50073	CO,UR,CR8,CR5	Streptococcus
45321	1	12448	CO	Streptococcus pseudopneumoniae
45324	3	37315	CO,UR,CR8	Streptococcus
45326	3	36393	CO,UR,CR5	Streptococcus
45343	2	24424	CO,UR	Streptococcus pseudopneumoniae
45350	3	35565	CO,UR,CR5	Streptococcus
45352	3	36297	CO,UR,CR5	Streptococcus
45357	2	25164	CR8,CR5	Streptococcus
45382	1	12320	CR8	Streptococcus thermophilus
45385	3	36808	CO,UR,CR5	Streptococcus
45389	1	11967	CR8	Streptococcus
45402	2	23560	UR,CR8	Streptococcus
45404	3	35601	CO,UR,CR5	Streptococcus
45405	1	11595	UR	Streptococcus
45427	3	36608	CO,UR,CR5	Streptococcus
45438	4	51429	CO,UR,CR8,CR5	Streptococcus
45440	2	23924	CO,UR	Streptococcus pseudopneumoniae
45474	1	13273	CO	Streptococcus
45481	4	48539	CO,UR,CR8,CR5	Streptococcus
45483	2	23509	CO,CR5	Streptococcus
45484	1	13316	CR8	Streptococcus
45487	1	12578	UR	Clostridium
45493	2	24310	CO,CR5	Streptococcus
45500	2	24014	CO,CR8	Streptococcus
45507	2	23845	CO,UR	Streptococcus pseudopneumoniae
45508	2	24753	CO,UR	Streptococcus pseudopneumoniae
45513	4	51124	CO,UR,CR8,CR5	Streptococcus

45523	4	50635	CO,UR,CR8,CR5	Streptococcus
45529	2	23704	UR,CR8	Streptococcus
45531	2	25302	CO,CR5	Streptococcus
45536	3	35829	CO,UR,CR5	Streptococcus
45542	4	54161	CO,UR,CR8,CR5	Streptococcus sanguinis
45543	1	12490	CO	Streptococcus pseudopneumoniae
45549	2	24659	CO,CR8	Streptococcus
45556	1	11967	CR8	Streptococcus
45563	4	48614	CO,UR,CR8,CR5	Streptococcus
45564	1	11767	CO	Streptococcus pseudopneumoniae
45578	1	12306	CR8	Streptococcus
45587	4	49940	CO,UR,CR8,CR5	Streptococcus
45592	1	11891	UR	Streptococcus pseudopneumoniae
45593	1	12493	CR8	Streptococcus thermophilus
45595	1	13056	UR	Streptococcus sanguinis
45601	2	24510	CO,CR8	Streptococcus
45626	3	36653	CO,UR,CR5	Streptococcus
45628	3	36463	CO,UR,CR5	Streptococcus
45636	2	23831	CO,UR	Streptococcus pseudopneumoniae
45679	4	48090	CO,UR,CR8,CR5	Streptococcus
45682	2	24311	UR,CR8	Streptococcus
45694	1	12188	CR8	Streptococcus
45695	4	47817	CO,UR,CR8,CR5	Streptococcus
45720	4	48960	CO,UR,CR8,CR5	Streptococcus
45725	1	11469	UR	Streptococcus
45735	1	12240	CO	Clostridium
45744	4	48722	CO,UR,CR8,CR5	Streptococcus
45762	1	11657	CR8	Streptococcus cristatus
45786	2	25295	CO,UR	Streptococcus pseudopneumoniae
45803	1	12496	CO	Streptococcus pseudopneumoniae
45828	2	24177	CO,CR8	Streptococcus
45837	2	24203	CO,CR8	Streptococcus
45844	4	48844	CO,UR,CR8,CR5	Streptococcus
45855	1	12436	CR8	Streptococcus
45861	2	23972	CO,UR	Clostridium
45870	4	47237	CO,UR,CR8,CR5	Streptococcus
45878	3	37634	CO,UR,CR5	Streptococcus
45879	4	48196	CO,UR,CR8,CR5	Streptococcus
45887	2	24622	CO,UR	Streptococcus pseudopneumoniae
45888	1	12884	CR8	Streptococcus cristatus
45896	1	12596	CR8	Streptococcus
45900	1	13739	CR8	Staphylococcus
45914	2	27025	UR,CR5	Staphylococcus epidermidis
45951	3	40503	CO,UR,CR5	Staphylococcus epidermidis
45953	1	13416	CR8	Staphylococcus epidermidis
45955	2	25974	UR,CR5	Bacillus flexus
45974	1	13143	CR8	Staphylococcus
45988	2	25635	CO,CR8	Staphylococcus haemolyticus
46013	1	13805	CR8	Staphylococcus
46025	1	13711	CR8	Staphylococcus epidermidis

46067	2	26760	CO,CR8	Staphylococcus epidermidis
46074	2	26339	UR,CR8	Staphylococcus
46079	2	26762	UR,CR8	Brochothrix thermosphacta
46081	1	13588	CR8	Staphylococcus
46095	1	12366	CR8	Brochothrix
46119	1	13485	CR8	Staphylococcus pettenkoferi
46138	1	13575	UR	Staphylococcus epidermidis
46157	2	26060	CO,CR8	Staphylococcus
46162	3	40108	CO,UR,CR5	Staphylococcus epidermidis
46163	1	13920	CR8	Staphylococcus epidermidis
46167	2	27309	UR,CR8	Staphylococcus
46176	1	13662	CR8	Staphylococcus
46189	4	54025	CO,CO_PMA,CR8,CR5	Staphylococcus epidermidis
46190	3	41023	CO,UR,CR5	Staphylococcus
46215	2	25793	CO,CR8	Staphylococcus epidermidis
46217	1	13637	UR	Staphylococcus epidermidis
46226	1	13401	UR	Staphylococcus epidermidis
46228	1	13553	CR8	Staphylococcus
46243	1	13372	CR8	Staphylococcus epidermidis
46244	1	11713	CO_PMA	Staphylococcus epidermidis
46259	4	48598	CO,UR,CR8,CR5	Staphylococcus
46260	1	13941	CR8	Staphylococcus epidermidis
46278	1	13353	CR8	Staphylococcus
46293	1	12891	CO	Staphylococcus epidermidis
46329	1	13387	CR8	Staphylococcus
46342	1	12407	CO	Staphylococcus
46360	1	12802	UR	Staphylococcus
46362	1	12717	UR	Staphylococcus
46367	1	13335	UR	Staphylococcus
46372	3	37350	CO,UR,CR5	Staphylococcus epidermidis
46398	3	38257	CO,UR,CR5	Staphylococcus haemolyticus
46405	1	13493	CR8	Staphylococcus epidermidis
46417	1	13546	UR	Staphylococcus epidermidis
46420	2	24661	CO_PMA,CR8_PMA	Staphylococcus
46450	3	39851	CO,UR,CR8	Staphylococcus epidermidis
46451	1	13618	CR8	Staphylococcus epidermidis
46463	1	13109	CO	Staphylococcus
46464	3	40182	CO,UR,CR8	Staphylococcus
46484	1	12156	CR8	Staphylococcus
46516	1	12973	CO	Staphylococcus epidermidis
46520	1	12617	UR	Bacillus cytotoxicus
46527	1	11499	CO_PMA	Staphylococcus
46532	2	27173	UR,CR5	Staphylococcus
46539	1	13134	CR8	Staphylococcus
46548	2	23764	CR8,CR5	Staphylococcus
46562	3	41774	CO,UR,CR5	Staphylococcus epidermidis
46568	1	12272	CO	Staphylococcus epidermidis
46571	1	13576	UR	Staphylococcus epidermidis
46607	2	26323	CO,CR8	Staphylococcus epidermidis
46608	2	27364	UR,CR8	Staphylococcus

46611	1	13825	CR8	Staphylococcus epidermidis
46612	2	26983	CO,CR8	Staphylococcus epidermidis
46621	2	27109	UR,CR8	Staphylococcus
46645	1	13176	CR8	Staphylococcus
46661	3	38369	CO,UR,CR5	Staphylococcus
46665	1	12175	CR8	Staphylococcus
46667	1	13378	CO	Staphylococcus epidermidis
46668	1	11896	CR5	Staphylococcus
46682	2	26392	CO,CR8	Staphylococcus epidermidis
46684	2	27317	UR,CR8	Staphylococcus epidermidis
46706	3	38165	CO,UR,CR5	Staphylococcus haemolyticus
46712	1	13623	UR	Staphylococcus epidermidis
46718	1	12472	CO	Staphylococcus epidermidis
46720	3	39797	CO,UR,CR5	Staphylococcus
46733	2	27930	CR8,CR5	Staphylococcus
46738	3	37296	CO,UR,CR5	Staphylococcus
46752	1	13487	UR	Staphylococcus epidermidis
46766	2	26802	UR,CR8	Staphylococcus
46779	2	25508	CO,CR8	Staphylococcus epidermidis
46793	1	12676	CO_PMA	Staphylococcus
46794	1	12816	CR8	Staphylococcus epidermidis
46809	2	27082	UR,CR8	Staphylococcus
46819	1	13308	CR8	Staphylococcus epidermidis
46826	1	13571	UR	Staphylococcus epidermidis
46828	1	13033	CR8	Staphylococcus
46868	1	11845	CO	Geobacillus
46886	3	40593	CO,UR,CR5	Staphylococcus epidermidis
46891	2	27308	UR,CR5	Staphylococcus
46894	1	12714	CR8	Staphylococcus
46905	2	26977	UR,CR8	Staphylococcus
46913	3	38786	CO,UR,CR5	Staphylococcus epidermidis
46929	1	13040	CR8	Staphylococcus
46947	1	13760	CR8	Staphylococcus
46963	1	13597	CR8	Staphylococcus
46981	5	64736	CO,UR,CR8,CR5,CO_PMA	Staphylococcus
46998	1	13277	UR	Staphylococcus epidermidis
47000	1	12216	CR8	Staphylococcus sciuri
47019	2	26832	UR,CR8	Staphylococcus
47034	1	13650	CR5	Staphylococcus epidermidis
47078	1	12007	CR8	Bacillus
47101	1	12571	CR8	Staphylococcus
47109	2	27189	UR,CR8	Staphylococcus epidermidis
47114	2	26722	CR8,CR5	Staphylococcus
47128	1	13213	CO	Staphylococcus epidermidis
47129	2	27096	UR,CR8	Staphylococcus
47130	1	12852	CO	Staphylococcus epidermidis
47135	3	39204	CO,UR,CR5	Staphylococcus
47137	1	13114	CR8	Staphylococcus epidermidis
47152	1	13632	CR8	Staphylococcus
47173	1	12958	CR8	Staphylococcus epidermidis

47175	1	13644	CR5	Staphylococcus
47185	1	13636	CR8	Staphylococcus
47187	2	25292	UR,CR8	Staphylococcus
47188	2	26197	CR8,CR5	Staphylococcus
47200	2	27025	UR,CR8	Staphylococcus epidermidis
47243	1	13096	CO	Staphylococcus
47246	1	12887	CR8	Staphylococcus
47248	1	13299	CO	Staphylococcus
47276	2	25940	CO,CR8	Staphylococcus epidermidis
47296	2	27180	UR,CR8	Staphylococcus
47306	1	13338	CR8	Staphylococcus epidermidis
47320	1	12874	CO	Staphylococcus epidermidis
47327	1	13036	UR	Staphylococcus
47333	1	13856	CR8	Staphylococcus epidermidis
47338	2	27235	UR,CR8	Staphylococcus
47341	1	13891	CR8	Staphylococcus epidermidis
47345	1	12472	CO	Staphylococcus epidermidis
47357	2	26304	UR,CR8	Staphylococcus
47361	1	12795	CO	Staphylococcus epidermidis
47419	1	12346	CO	Staphylococcus epidermidis
47425	1	12711	CO	Staphylococcus epidermidis
47434	1	11657	CR8	Geobacillus
47445	3	40440	CO,UR,CR5	Staphylococcus epidermidis
47450	1	11544	CR8	Pontibacillus
47462	1	13599	CR8	Staphylococcus epidermidis
47476	2	26153	CR8,CR5	Staphylococcus
47479	1	13483	CR8	Staphylococcus
47519	1	13617	CR8	Staphylococcus
47528	1	11973	CO_PMA	Staphylococcus
47544	2	27143	UR,CR8	Staphylococcus
47551	1	12961	CO	Staphylococcus epidermidis
47552	2	27790	UR,CR8	Staphylococcus epidermidis
47564	1	12758	CO	Staphylococcus epidermidis
47616	4	51552	CO,UR,CR8,CR5	Staphylococcus epidermidis
47626	2	26030	CR8,CR5	Staphylococcus
47628	2	24716	CO,CR5	Geobacillus
47638	2	25646	CO,CR8	Staphylococcus haemolyticus
47663	2	26725	UR,CR8	Staphylococcus
47666	1	12519	CO	Staphylococcus epidermidis
47672	1	13157	CO	Staphylococcus epidermidis
47676	1	13422	CR8	Staphylococcus
47680	1	13487	UR	Staphylococcus epidermidis
47715	2	27383	UR,CR8	Staphylococcus epidermidis
47724	2	27391	CO,CR8	Staphylococcus epidermidis
47728	1	13460	UR	Staphylococcus
47738	1	11825	CR8	Staphylococcus sciuri
47756	2	26618	CO,CR5	Staphylococcus epidermidis
47761	1	12800	CO	Staphylococcus epidermidis
47766	1	13143	CR8	Staphylococcus
47792	1	13185	CR8	Staphylococcus epidermidis

47806	5	66097	CO,UR,CR8,CR5,CO_PMA	Staphylococcus epidermidis
47819	2	25013	UR,CO_PMA	Staphylococcus epidermidis
47821	1	13097	CO	Staphylococcus
47832	3	39998	CO,UR,CR5	Staphylococcus epidermidis
47841	3	37634	CO,UR,CR8	Staphylococcus
47862	1	13671	CR8	Staphylococcus
47869	1	13697	UR	Staphylococcus epidermidis
47878	3	40634	CO,UR,CR5	Staphylococcus epidermidis
47947	2	27067	UR,CR8	Staphylococcus
47960	1	12506	CR8	Brochothrix thermosphacta
47963	2	25929	CO,CR8	Staphylococcus haemolyticus
47993	1	13752	CR5	Staphylococcus epidermidis
48021	2	23791	UR,CR5	Staphylococcus arlettae
48027	1	12973	CO	Staphylococcus epidermidis
48039	2	25814	UR,CR8	Staphylococcus
48048	1	13429	UR	Staphylococcus
48075	4	51466	CO,UR,CR8,CR5	Staphylococcus epidermidis
48081	1	13438	UR	Staphylococcus epidermidis
48083	2	26820	UR,CR8	Staphylococcus epidermidis
48089	1	13370	CR8	Staphylococcus haemolyticus
48099	1	13229	CR8	Staphylococcus epidermidis
48122	1	13846	CR8	Staphylococcus epidermidis
48138	1	13155	CR8	Staphylococcus
48156	1	13239	CR8	Staphylococcus
48159	1	13465	CR8	Staphylococcus epidermidis
48186	1	13309	CR8	Staphylococcus epidermidis
48200	2	26327	UR,CR8	Staphylococcus
48213	2	26733	CO,CO_PMA	Bacillus
48215	1	13204	CO_PMA	Bacillus
48279	2	26844	CO,CO_PMA	Bacillus
48318	1	12927	CO_PMA	Bacillus
48364	1	13171	CO_PMA	Bacillus
48388	1	13471	CO_PMA	Bacillus
48423	2	26662	CO,CO_PMA	Bacillus
48440	1	13247	CO_PMA	Bacillus
48497	2	26338	CO,CO_PMA	Bacillus
48536	2	27046	CO,CO_PMA	Bacillus
48581	2	26582	CO,CO_PMA	Bacillus
48645	1	12513	CO_PMA	Bacillus
48660	1	12788	CO_PMA	Bacillus
48662	1	13373	CO_PMA	Bacillus
48675	1	13193	CO_PMA	Bacillus
48690	1	11855	CO_PMA	Bacillus
48691	1	13274	CO_PMA	Bacillus
48710	1	12836	CO	Bacillus
48776	1	12698	CR8_PMA	Bacillus
48784	1	12851	CO_PMA	Bacillus
48805	1	13152	CO_PMA	Bacillus
48819	2	25698	CO,CO_PMA	Bacillus
48821	2	26949	CO,CO_PMA	Bacillus

48825	1	11817	CO_PMA	Bacillus
48830	2	26537	UR,CR5	
48851	1	13156	CO_PMA	Bacillus
48861	1	13016	CO_PMA	Bacillus
48912	1	11896	CR5	Desulfotomaculum
48936	2	25491	CO,CO_PMA	Bacillus
48975	1	12973	CO_PMA	Bacillus
48981	1	12819	CO_PMA	Bacillus
49022	1	13029	CO_PMA	Bacillus
49050	1	12530	CO_PMA	Bacillus
49052	2	26593	CO,CO_PMA	Bacillus
49101	1	12163	CO	Thermoanaerobacterium saccharolyticum
49107	1	12819	CO_PMA	Bacillus
49129	1	13591	CO_PMA	Bacillus
49160	1	13405	CO_PMA	Bacillus
49167	2	26746	CO,CO_PMA	Bacillus
49168	1	13148	CO_PMA	Bacillus
49172	1	13538	CO_PMA	Bacillus
49178	2	27334	CO,CO_PMA	Bacillus
49187	2	26831	CO,CO_PMA	Bacillus
49213	1	11725	CO	Desulfotomaculum
49214	1	13583	CO_PMA	Bacillus
49265	1	13419	CO_PMA	Bacillus
49268	2	26440	CO,CO_PMA	Bacillus
49305	1	12680	CO_PMA	Bacillus
49312	1	11787	CO	Bacillus
49322	1	12183	CR8_PMA	Bacillus
49325	2	26619	CO,CO_PMA	Bacillus
49357	1	13373	CO_PMA	Bacillus
49365	1	13631	CO_PMA	Bacillus
49366	1	13371	CO_PMA	Bacillus
49395	2	26565	CO,CO_PMA	Bacillus
49397	1	12999	CR5	Thermicanus
49446	2	27046	CO,CO_PMA	Bacillus
49493	2	26856	CO,CO_PMA	Bacillus
49495	1	13413	CO_PMA	Bacillus
49516	1	12712	CR8	Bacillus acidicola
49532	2	25412	CO,CO_PMA	Bacillus
49591	1	13709	CO_PMA	Bacillus
49623	1	13216	CO_PMA	Bacillus
49637	2	27233	CO,CO_PMA	Bacillus
49665	1	11806	CR8	Bacillus acidicola
49669	1	13062	CO_PMA	Bacillus
49688	1	12845	CO_PMA	Bacillus
49766	1	13421	CO	Bacillus
49864	1	12941	CO_PMA	Bacillus
49874	1	12003	CO_PMA	Bacillus
49912	1	13176	CO_PMA	Bacillus
49937	1	13563	CO_PMA	Bacillus
49990	2	24741	CO,CR8	Veillonella

50031	2	24974	CR8,CR5	Paenibacillus
50043	1	11723	CO	Veillonella
50073	1	11880	CO	Veillonella
50100	1	11643	CR8	Paenibacillus
50102	2	24220	CO,CR8	Paenibacillus
50179	1	13058	CR8	Paenibacillus
50184	1	12641	CR5	Collinsella aerofaciens
50382	1	12298	CR8	Paenibacillus
50392	1	12794	CR8	Paenibacillus
50464	2	24918	CO,CR8	Veillonella
50477	1	12420	CR5	Collinsella aerofaciens
50564	1	12894	CO	
50569	2	24271	CO,CR8	Veillonella
50574	1	11930	CO	Veillonella dispar
50648	1	12225	CR8	Paenibacillus
50707	2	24821	CO,CR8	Veillonella
50725	2	23280	CO,CR8	Veillonella
50779	1	11394	CR8_PMA	Paenibacillus
50847	2	23884	CO,CR5	Collinsella aerofaciens
50857	1	12428	CR8	Paenibacillus
50864	1	13552	CR5	Adlercreutzia
50911	1	12707	CR5	Paenibacillus
51048	1	11946	CR8	
51431	1	12040	CO	sfF
51571	1	12088	CO	sfA
51676	1	12945	CR5	
51836	3	39653	CO,UR,CR5	sfF
51890	1	13778	CR8	
51929	1	11941	CO	
51982	1	13397	CR8	
52142	1	12072	CR8	
52201	1	13386	CR8	HN1-15
52294	1	11877	CR8	sfC
52305	1	11998	UR	Roseiflexus
52341	1	12251	CR8	sfAT
52380	1	11769	CR8	Syntrophus
52401	2	24890	CO,CR8	
52457	1	12155	CO	sfC
52703	1	11523	CR8	Desulfobacterium
52737	1	11812	CO	sfE
52805	1	11813	CR5	HN1-15
52824	0	0	COURCR8CR5CO_PMAUR_PMACR8_PMACR5_PMA	
52843	1	12482	CR8	sfD
52935	1	13210	CR5	Chroococcidiopsis
53292	1	12412	CO	Anabaena
53368	2	24003	CO,CR8	Fischerella muscicola
53583	1	12029	CO	Aphanizomenon issatschenkoi
53672	1	12785	CO	Halomicronema
53678	2	25875	CR8,CR5	Phormidium animale
53679	1	12312	CR5	Deinococcus

53690	1	11973	CR5_PMA	A4b
53704	1	12016	CO	
53750	1	12521	CR8	Deinococcus
53818	1	11986	CO	Symploca
53827	1	13152	CO	Deinococcus
53955	2	25889	UR,CR8	sfB
53967	2	25335	CR8,CR5	sfB
54147	1	12150	CR5	
54344	1	12186	CO_PMA	SHAG537
54684	1	13169	CR5	
54850	1	12133	CO	
54955	2	24180	CO,CR8	HA73
55241	1	11704	CR8	
55347	2	25426	CO,CR8	
55518	1	13217	CO	
55774	1	12582	CO	
55906	1	11537	CR8	
56113	1	13119	CO	
56168	1	12366	CO	sfB
56623	1	12435	CO	
56900	1	12992	CO_PMA	
57019	1	11877	CO	
57308	1	12503	CR8	Isosphaera
57341	1	12354	CR8	Desulfovibrio putealis
57357	4	52622	CO,UR,CR8,CR5	Planctomyces
57583	3	36177	CO,UR,CR5	
57616	1	12717	CO	
57707	1	12209	CO	
57878	1	12297	CR5	Gemmatimonas
57982	2	26638	CR8,CR5_PMA	sfM
58114	1	11983	CO	Cystobacter minus
58123	1	13022	CR5	
58316	1	12342	CR5	sfA
58369	2	24095	CO,CR5	
58422	1	12126	CO	sfA
58435	2	27111	CO_PMA,CR8_PMA	
58446	2	23922	CO,CR8	Cystobacter minus
58484	1	12626	CR5	sfF
58534	2	24108	CO,CR8	sfG
58535	1	12502	CO	
58556	1	11866	CO	Anaeromyxobacter sp. Fw109-5
58567	3	38739	CO,UR,CR5	
58579	1	12807	CO	sfC
58588	1	12352	CR5	
58597	1	12406	CO	sfC
58619	1	13227	CO	
58625	2	24658	CO,CR8	
58646	1	12482	CO	sfD
58667	1	11886	CO	sfA
58691	1	11751	CO	Archangium

58706	1	11611	CR8	Myxococcus
58728	1	11944	CR5	sfF
58818	1	12184	CR5	sfA
58886	2	24655	CO,CR8	Methylobacterium
58889	1	12244	CR8	Bosea
58959	2	23319	UR,CR8	Mycoplana
58971	3	38453	CO,UR,CR8	Brevundimonas diminuta
58976	1	12983	CR8	Bradyrhizobium canariense
58982	2	23362	CO,CR8	Brevundimonas
58987	1	12954	UR	Brevundimonas diminuta
58993	1	13402	CR5_PMA	Bradyrhizobium elkanii
59027	1	12273	CO	
59047	1	12124	CR5	Rhodoplanes
59053	1	12959	CR8	
59062	2	26033	UR,CR8	Brevundimonas
59070	1	12526	CO	sfA
59071	1	11912	CO	Brevundimonas diminuta
59087	1	11892	CR8	Methylobacterium adhaesivum
59105	1	11768	CO	
59127	2	25820	CO,CR8	
59143	2	24564	CR8_PMA,CR5_PMA	Bradyrhizobium
59158	1	12009	CR8	Brevundimonas diminuta
59178	2	25607	UR,CR8	Brevundimonas
59195	1	12148	CO	Brevundimonas diminuta
59196	1	12827	CR8	Brevundimonas diminuta
59198	2	25628	CO,CR8	
59212	1	11672	CO	Methylobacterium zatmanii
59224	1	12589	CR5	Rhodoplanes
59247	3	35332	CO,UR,CR5	Methylobacterium
59259	2	24035	CO,CR8	Methylobacterium komagatae
59279	2	25967	CO,CR8	
59284	1	12274	CO	Brevundimonas diminuta
59299	1	12394	CO	
59317	1	12156	CO	Brevundimonas diminuta
59318	2	24177	CR8,CR5	Mycoplana
59321	1	11750	CO	Caulobacter
59322	1	12164	CR8	Bosea
59373	1	12802	CR8	
59396	1	11675	CO	sfA
59404	3	35104	CO,UR,CR5	Methylobacterium
59410	1	11848	CR8	Methylobacterium
59417	1	11655	CR8	Methylobacterium
59420	2	24816	CO,CR8	Methylobacterium komagatae
59421	2	25782	CO,CR5	
59454	2	27106	CO,CR8	
59463	2	25845	CO,UR	Brevundimonas diminuta
59486	1	11956	CO	Bradyrhizobium
59524	1	13190	CR8_PMA	
59547	1	12773	CR5	Methylobacterium
59549	1	11726	CR8	Methylobacterium

59551	1	12493	CR8	Methylobacterium
59560	4	50130	CO,CO_PMA,CR8,CR5	Methylobacterium
59566	2	26231	CO,CR8	
59571	3	35818	CO,UR,CR5	Methylobacterium
59578	1	11535	CR8	
59636	1	12410	CO	Bradyrhizobium
59710	1	11816	CO	Methylobacterium
59718	1	12001	CO	Methylobacterium zatmanii
59725	3	38254	CO,UR,CR5	Methylobacterium
59740	3	35692	CO,UR,CR8	Brevundimonas
59754	1	11767	CO	Methylobacterium
59755	1	11993	CR8	
59760	3	37187	CO,UR,CR8	
59786	2	24758	CO,CR8	Methylobacterium fujisawaense
59791	1	12253	CO	Methylobacterium
59792	1	12367	CO_PMA	
59796	1	11722	CR5	Methylobacterium radiotolerans
59826	1	11904	CO	Methylosinus sporium
59837	3	37433	CO,UR,CR5	Skermanella
59875	4	51131	CO,UR,CR8,CR5	Brevundimonas
59890	1	11804	CO	Methylobacterium fujisawaense
59903	2	24822	CO,CR8	Methylobacterium komagatae
59904	2	25073	CO,CR8	Methylobacterium
59915	4	52135	CO,CO_PMA,CR8,CR5	Methylobacterium
59935	2	24399	UR,CR8	Bosea
59959	1	12717	CO	Caulobacter
59960	1	12494	CO	Brevundimonas diminuta
59983	1	12022	CO	Bradyrhizobium
59989	2	28391	CO_PMA,CR8_PMA	Afipia broomeae
60010	1	12948	UR	
60031	2	24437	CO,CR8	
60042	1	14106	CO_PMA	Afipia
60050	2	24451	CO,CR8	
60065	1	12116	CO	Brevundimonas diminuta
60071	2	25773	CO,CR8	
60072	2	24008	UR,CR8	Brevundimonas
60100	2	23760	CO,CR8	Brevundimonas
60105	1	11583	CR8	Skermanella
60119	4	49392	CO,UR,CR8,CR5	Brevundimonas
60205	3	36959	CO,UR,CR5	Sphingomonas wittichii
60211	2	23123	CO_PMA,CR8_PMA	Rhodoplanes
60231	1	11830	CR8	Roseomonas
60246	2	24965	CR8,CR5	Novosphingobium
60278	1	12120	CO	Sphingomonas wittichii
60279	1	12071	UR	
60281	4	51370	CO,UR,CR8,CR5	Sphingomonas
60291	1	12132	CR8	Sphingobium
60301	1	11764	CO	Sphingobium
60307	1	11854	CR5	Sphingomonas asaccharolytica
60315	5	61826		Sphingomonas

60328	1	12051	CR8	Devosia
60332	1	12147	CO_PMA	Sphingobium
60346	1	11607	CR8	Novosphingobium
60348	3	40032	CR5,CO_PMA,CR8_PMA	Sphingomonas
60351	2	23315	CO,CR8	Sphingomonas
60374	2	25385	CR8,CR5	Sphingobium herbicidovorans
60378	3	38603	CO,UR,CR5	
60395	3	38305	CR8,CO_PMA,CR8_PMA	Sphingobium
60398	2	24228	CO_PMA,CR8_PMA	Sphingomonas
60405	3	36320	CO,UR,CR5	
60421	1	12227	CR8_PMA	Sphingobium
60425	1	1	CR5_PMA	
60430	1	11969	CR8	Sphingomonas wittichii
60447	1	11765	CR8	Novosphingobium
60449	1	11943	CO	Sphingomonas
60467	3	39592	CO,UR,CR5	Roseomonas
60472	1	12846	CR8	Devosia
60475	3	38465	CO,UR,CR5	Roseomonas
60492	1	11714	CO	
60523	3	40082	CO,UR,CR5	
60538	1	12421	UR	Sphingobium
60557	1	12421	UR	Sphingobium
60560	2	23727	CO,CR8	Oceanicaulis
60564	1	11733	CR5	Sphingomonas
60565	1	11528	UR	Erythrobacter
60568	1	12338	CO_PMA	Sphingomonas
60579	2	23772	CO_PMA,CR8_PMA	Sphingobium
60608	1	12403	CR8	Novosphingobium
60638	1	12556	CR8	Novosphingobium
60672	2	24258	CO,CR8	Sphingomonas
60697	1	11763	UR	Sphingopyxis
60732	4	50432	CR8,CR5,CO_PMA,CR8_PMA	Sphingobium
60741	2	24161	CO,UR	sfZ
60758	1	12008	CR8	Sphingomonas
60769	1	11805	CO	Novosphingobium
60770	1	11769	CO_PMA	Sphingomonas
60808	2	25997	CO,CR5	Novosphingobium
60822	1	12438	UR	Sphingobium
60826	1	12131	CO_PMA	Sphingomonas
60827	4	50477		Sphingomonas
60837	3	36293	CO,UR,CR5	Novosphingobium
60840	1	11828	CO	
60851	1	11892	CO	Roseomonas
60858	1	11623	CR5	Sphingomonas
60902	1	11444	CO_PMA	Sphingobium
60910	1	12369	CO	
60916	2	25481	CO,CR5	Kaistobacter
60926	1	12743	CO	
60931	1	12597	CO_PMA	Sphingomonas
60961	1	12280	CO	Novosphingobium

60966	1	12317	CR8	Sphingomonas
60970	1	11765	CO_PMA	Sphingobium
61013	3	38693	CO,UR,CR5	Sphingobium
61046	1	12048	CR8	Novosphingobium
61060	3	37640	CO_PMA,CR8_PMA,CR5_PMA	Caulobacter leidyia
61062	2	24005	CO,CR8	Sphingomonas
61082	2	24146	CO,CR5	Acidisphaera
61087	1	13145	CO	Sphingomonas
61094	2	26626	CO,UR	
61101	1	12289	UR	Sphingobium
61102	1	11774	CO_PMA	Novosphingobium resinovorum
61105	3	36764	CO,UR,CR8	
61106	1	12469	CR8	Sphingomonas yabuuchiae
61127	1	12292	CO_PMA	Sphingobium
61133	3	35731	CO,UR,CR5	Novosphingobium
61158	5	62564	UR,CR8,CO_PMA,UR_PMA,CR8_PMA	Sphingomonas
61191	3	36925	CO,UR,CR5	Sphingomonas
61277	1	11944	CR8	
61285	1	12006	CR8	Devosia
61290	1	11746	CO	Sphingobium
61302	3	36253	CR8,CO_PMA,CR8_PMA	Sphingobium
61317	1	12861	UR	
61325	1	12813	CR5	
61327	2	23884	CO_PMA,CR8_PMA	Sphingobium
61333	1	11811	CO_PMA	
61336	1	12017	CO	Sphingomonas
61355	3	38669	CO,UR,CR5	Novosphingobium
61385	1	12087	CO	Rhizobium
61473	3	38711	CO,UR,CR5	Rubellimicrobium
61538	1	11845	CO	sfA
61557	1	11866	CO	Nordella
61612	1	13008	CO	
61677	1	13251	CR8	Rubellimicrobium
61693	1	12427	CO	Agrobacterium rubi
61699	1	13320	CO	
61764	1	12832	CO	Rhizobium
61774	1	12049	CO	Rhizobium
61785	1	12201	UR	Agrobacterium
61810	1	12886	CO	Rhizobium
61835	1	13260	CR5	Rubellimicrobium
61909	4	51899	CO,UR,CR8,CR5	Paracoccus aminovorans
61913	1	12070	CO	Rhizobium
61935	1	11484	UR	Shinella
61965	3	39310	CO,CO_PMA,CR8	Paracoccus
61966	1	13120	CO	
61984	1	11708	CR8	Paracoccus
62019	1	12797	CO	
62086	1	11861	CR8	Candidatus Liberibacter africanus
62194	1	12427	CO_PMA	
62220	1	11663	CO_PMA	Paracoccus

62280	1	13206	CO	Paracoccus
62320	1	12087	CO	Rhizobium
62348	1	13252	CO	Paracoccus
62371	1	12227	CO_PMA	Paracoccus
62391	1	12725	CO	Nautella italica
62417	1	12631	CO	
62419	1	11975	CO	Rhizobium
62424	1	12691	CO	Rhizobium
62439	2	24266	CO,CR5	Rhizobium
62450	1	13108	CO	Rhizobium
62476	2	27058	CO,CR8	Paracoccus
62483	2	2	CR8_PMA,CR5_PMA	
62496	2	25254	CO,CO_PMA	
62512	1	12153	CO	Agrobacterium rubi
62521	1	11849	CR8	Paracoccus aminovorans
62538	1	12500	CO	
62578	1	11694	CO_PMA	
62585	1	13139	CR8	Paracoccus yeei
62664	2	26935	CO,UR	sfA
62669	1	12090	CO	Rhizobium
62719	1	12276	CR8	Paracoccus
62724	1	11676	CO	
62734	1	13415	CR8	Paracoccus
62760	1	12302	CO	Rhizobium
62769	1	12723	CR5	Paracoccus aminovorans
62790	1	12321	CO	
62812	1	11918	CO	Rhizobium
62819	1	12130	CO	
62825	2	24549	CO,CR8	Rubellimicrobium
62853	1	12806	CR8	
62890	1	12316	CR8	Paracoccus
62927	1	11664	CR8	
63093	1	13161	CR8	
63161	1	11766	UR	Chryseobacterium
63182	1	12518	UR	
63187	1	11933	CO	Chryseobacterium
63286	1	12329	CR8	Chryseobacterium
63336	1	12522	CR8	
63400	1	11835	CR5	Chryseobacterium
63573	3	39301	CO,UR,CR5	Chryseobacterium
63711	1	12149	UR	Chryseobacterium
63864	2	25030	CR8,CR5	Chryseobacterium
64220	2	25144	CR8,CR5	Chryseobacterium
64568	2	24912	CO,CR8	
64623	1	11875	CR5	Chryseobacterium
64654	1	11751	CO	Chryseobacterium
64734	1	12371	CR5	Chryseobacterium
64986	1	12015	CR8	Chryseobacterium
65050	1	11923	CR8	Chryseobacterium
65435	1	12410	CR8	Chryseobacterium

65635	1	12926	CR8	Chryseobacterium
65807	1	12724	CR8	Chryseobacterium
65875	2	25512	CR8,CR5	
65912	1	11894	CO	Flavobacterium
66457	1	12322	UR	Wautersiella
66513	1	12685	UR	Wautersiella
66605	2	24064	CO,CR8	Flavobacterium columnare
66715	2	24424	CR8,CR5	Flavobacterium
66896	1	12297	UR	Sphingobacterium
67028	1	11625	CR8	Prevotella
67542	1	11698	CR5	Prevotella
67664	1	11728	CR8	Prevotella
68290	1	11782	CR8	
68573	1	12789	CR5	
69046	1	12758	CO	
69286	1	11760	CR5	
69319	1	12649	CO	
69417	1	12463	CO	
69519	1	12266	CO	
69633	1	11732	CO	
69681	2	23705	CO,CR5	
69770	2	27711	CO,CR5	
69931	3	38502	CO,UR,CR5	
69992	3	36059	CO,UR,CR8	Finegoldia
70043	1	12593	CR5	
70051	3	36427	CO,UR,CR8	Finegoldia magna
70082	2	26512	CO,CR8	Eubacterium
70118	2	24434	CO,CR8	Eubacterium
70179	2	25779	CR8,CR5	
70180	1	12690	CR5	Peptoniphilus
70289	1	12934	CO	Peptoniphilus
70417	1	12026	CR5	
70425	1	12662	CO	
70485	1	12711	CO	
70579	1	12663	CO	
70606	2	25566	CO,CR5	
70627	1	12484	CR8	Helcococcus
70639	2	24646	CO,CR8	
70669	2	25006	CO,CR8	Anaerococcus
70706	1	12182	CR8	Anaerococcus
70712	1	13083	CR5	Anaerococcus
70729	1	11956	CR8	
70775	1	11768	CR8	
70828	1	11793	CO_PMA	Spiroplasma
70838	1	12020	CO	
70850	3	38593	CO,UR,CR5	Anaerococcus
70892	1	12258	CO	
70902	1	11777	CO	
70911	3	39312	CO,UR,CR5	
70956	1	12007	CO	

70959	1	13196	CR5	Anaerococcus
70970	2	25024	CO,CR8	
71020	2	26145	CR8,CR5	Anaerococcus
71056	3	38021	CO,UR,CR5	
71075	2	26288	CR8,CR5	Anaerococcus
71089	1	11855	CR8	Anaerococcus hydrogenalis
71091	1	12535	CR8	Anaerococcus
71105	1	12160	CO	
71113	1	11714	CR8	Anaerococcus
71159	1	11871	CO	
71178	1	11819	CO	
71190	1	13010	CO	
71191	1	12668	CR8	Anaerococcus
71202	4	53724	CO,UR,CR8,CR5	Anaerococcus
71224	1	11898	CR8	Anaerococcus prevotii
71226	1	13106	CR8	Anaerococcus
71261	3	36617	CO,UR,CR8	
71274	3	36646	CO,UR,CR5	
71411	1	12388	CR8	sfA
71526	1	11881	CO	Zplanct13
71601	3	36162	CO,UR,CR5	sfA
71623	1	11738	CO	PSB-M-3
71820	2	25564	CR8,CR5	Lactobacillus crispatus
71863	1	12258	CR8	Leuconostoc citreum
71907	1	12044	CR8	Leuconostoc
71916	1	12106	CR8	Leuconostoc citreum
71919	3	35960	CO,UR,CR5	Leptotrichia
71936	1	11780	UR	Lactobacillus
71954	1	12580	UR	Lactobacillus
71968	1	11613	UR	Lactobacillus
72006	1	11728	CR5	Lactobacillus
72019	1	12021	UR	Lactobacillus panis
72042	1	12984	UR	Lactobacillus
72061	3	37244	CO,UR,CR5	Rickettsia australis
72132	1	13585	CR5	Lactobacillus
72147	2	25068	UR,CR8	Leuconostoc citreum
72160	1	13004	UR	Lactobacillus
72197	1	12573	CR5	Lactobacillus
72200	1	12776	CR8	Leuconostoc citreum
72201	2	25688	CO,CR5	Lactobacillus
72285	2	25839	CO,CR5	Lactobacillus
72329	1	12322	CO	Rickettsia
72453	1	12038	CR8	Leuconostoc citreum
72531	1	12811	CR8	Carnobacterium divergens
72563	2	24717	CR8,CR5	Lactobacillus
72582	1	12221	CR8	
72587	1	12305	CO	Clostridium perfringens
72592	1	13179	CR5	Rickettsia akari
72597	1	13251	UR	Lactobacillus
72610	1	12113	CR8	Leuconostoc citreum

72629	1	12301	CR8	Leuconostoc
72637	1	11639	CR8	Lactobacillus
72646	1	12113	CR8	Leuconostoc
72729	1	12371	CR8	Lactobacillus
72761	2	25742	CO,CR8	Lactobacillus
72795	1	11772	CR8	
72839	2	26649	CO,CR8	Lactobacillus
72855	1	12698	UR	Lactobacillus
72859	1	12055	UR	Lactobacillus crispatus
72884	2	25168	CR8,CR5	Lactobacillus
72916	1	12320	CR8	Lactobacillus
72927	1	12177	CR8	Leuconostoc
72956	1	12587	CR5	Rickettsia akari
72987	1	11823	CR8	Leuconostoc
73168	1	12009	CR5	Dyadobacter
73491	1	12616	CR5	Runella
73514	2	27198	CR8,CR5	Hymenobacter
73689	2	25281	UR,CR8	Cytophaga
73726	1	11764	CR8	Candidatus Odyssella
73871	1	12733	CR8	
73923	1	12801	CO	Fulvivirga
74031	4	49322	CO,UR,CR8,CR5	Hymenobacter
74065	2	24333	CR8,CR5	Hymenobacter
74285	2	25557	CO,CR5	Hymenobacter
74655	1	11981	CO	
74689	2	24092	CO,CR5	sfJ
74948	1	12550	CO	
75099	3	36716	CO,UR,CR5	sfA
75605	1	12253	CO	
75644	3	37266	CO,UR,CR5	sfA
75661	1	12101	CR8	sfJ
75676	1	11654	CR8	
75878	1	13002	CO	sfA
76572	1	12997	CO	Spirochaeta
CO	945	11918756		
CO_PMA	203	2404656		
CR5	639	8097025		
CR5_PMA	23	143800		
CR8	1011	12613814		
CR8_PMA	69	732870		
UR	513	6383769		
UR_PMA	2	25634		

Table S10.2. real edge table for rOTU network (based on PhyloChip data) and visualization in Cytoscape

from	to	eweight
CO	10116	12142
CO	10173	13212
CO	10596	12310
CO	10657	12765
CO	10737	11758
CO	10744	12389
CO	10760	12733
CO	10870	12572
CO	1095	12846
CO	10995	12305
CO	11150	13235
CO	11204	13581
CO	11208	12047
CO	11318	13008
CO	11490	12950
CO	11636	13283
CO	11657	13021
CO	11724	12904
CO	11897	11821
CO	11929	11737
CO	11980	12000
CO	12094	13336
CO	12175	12449
CO	12187	12139
CO	12211	11876
CO	12392	13042
CO	12745	12717
CO	12870	12851
CO	12882	12049
CO	12912	12554
CO	12928	11758
CO	12934	11996
CO	13091	12582
CO	13092	12017
CO	13106	12186
CO	13122	12581
CO	13274	12808
CO	13362	11753
CO	13882	12174
CO	14199	12840
CO	14292	12474
CO	14622	12390
CO	1481	12449
CO	14831	13172
CO	15016	12224
CO	15127	12926
CO	15459	12157
CO	15581	12883
CO	15621	12073
CO	15679	11703
CO	15719	12076
CO	15731	12129
CO	15831	12183
CO	15917	12449
CO	15920	12350
CO	15978	11716
CO	15987	12425
CO	16065	12179
CO	16161	12379
CO	16221	12675
CO	16340	12229
CO	16349	12525
CO	16376	12836

CO	16384	12538
CO	16425	12382
CO	16495	12702
CO	16687	13050
CO	16734	12643
CO	16819	12434
CO	16847	12169
CO	16916	12113
CO	16937	13983
CO	17357	12146
CO	17392	13006
CO	19752	11891
CO	20192	12194
CO	21155	11997
CO	22190	12305
CO	23140	11862
CO	23182	12434
CO	23349	12089
CO	23897	12552
CO	24119	12096
CO	24447	13098
CO	24455	11751
CO	24600	11860
CO	24602	12434
CO	24608	11753
CO	24612	11763
CO	24646	11747
CO	24700	11938
CO	24841	12027
CO	24857	12123
CO	24978	12300
CO	24996	12177
CO	25078	12295
CO	25126	11802
CO	25139	12505
CO	25273	11677
CO	25288	12453
CO	25291	11832
CO	25319	11767
CO	25341	13178
CO	25380	12952
CO	25499	12043
CO	25549	11785
CO	25558	12000
CO	25604	11867
CO	25719	11818
CO	25759	11729
CO	25804	11964
CO	25901	13219
CO	25950	12251
CO	25994	12652
CO	26041	12013
CO	26109	12075
CO	26129	11990
CO	26219	12034
CO	26227	12453
CO	26356	11745
CO	26371	12602
CO	26430	11954
CO	26462	12772
CO	26599	12082
CO	26611	11793
CO	26613	11866
CO	26744	11733
CO	26746	12132
CO	26768	12505
CO	26910	11990

CO	26984	11803
CO	28113	12974
CO	28664	12106
CO	28989	12163
CO	29901	12451
CO	29909	12436
CO	30017	13291
CO	30922	12691
CO	30996	14191
CO	31205	12816
CO	32200	13021
CO	33058	13426
CO	33700	13858
CO	34240	12813
CO	34464	13545
CO	34741	12471
CO	35227	12622
CO	35586	12847
CO	36142	13059
CO	36759	13071
CO	36763	13265
CO	36785	13243
CO	36787	12068
CO	36803	13635
CO	36823	12201
CO	36837	12727
CO	36911	13247
CO	36930	12302
CO	36931	11937
CO	36933	13277
CO	36937	13155
CO	36943	13112
CO	36983	12006
CO	37007	12430
CO	37025	12507
CO	37036	12285
CO	37061	12684
CO	37089	13166
CO	37107	13147
CO	37124	12637
CO	37125	13217
CO	37129	13302
CO	37161	12825
CO	37166	11848
CO	37178	13148
CO	37183	12620
CO	37193	13201
CO	37226	12388
CO	37268	12095
CO	37280	13406
CO	37293	12361
CO	37312	12668
CO	37315	13290
CO	37325	13116
CO	37390	11803
CO	37394	12674
CO	37398	12794
CO	37429	12905
CO	37506	13089
CO	37536	12604
CO	37549	12052
CO	37576	13109
CO	37598	13184
CO	37630	12109
CO	37671	12795
CO	37679	12538
CO	37688	12943

CO	37779	12834
CO	37783	13074
CO	37813	12917
CO	37814	12756
CO	37820	12910
CO	37835	13181
CO	37838	13036
CO	37868	12011
CO	37877	13600
CO	37907	12241
CO	37917	12018
CO	37921	12868
CO	37948	13049
CO	37955	11882
CO	37963	12125
CO	37984	12725
CO	38001	12709
CO	38072	12562
CO	38083	12068
CO	38099	12716
CO	38101	13302
CO	38164	13477
CO	38210	12716
CO	38238	11728
CO	38248	12888
CO	38266	13304
CO	38393	12895
CO	38427	12989
CO	38432	12969
CO	38433	12917
CO	38444	11712
CO	38524	13162
CO	38640	13368
CO	38646	13082
CO	38691	12262
CO	38721	12670
CO	38747	12222
CO	38755	12545
CO	38823	12702
CO	38848	12958
CO	38907	13685
CO	38920	12946
CO	38958	13215
CO	38996	13656
CO	39081	13791
CO	39084	11970
CO	39092	12732
CO	39094	11718
CO	39109	12597
CO	39124	13012
CO	39242	12308
CO	39259	12830
CO	39424	12729
CO	39435	12599
CO	39484	12949
CO	39541	12805
CO	39558	12953
CO	39602	12378
CO	39646	11672
CO	39650	13530
CO	39654	13181
CO	39661	13087
CO	39719	13211
CO	39733	13728
CO	39746	12532
CO	39802	12692
CO	39819	11771

CO	39857	12714
CO	39910	13699
CO	39946	13078
CO	39968	12773
CO	39989	12829
CO	40014	12335
CO	40039	12818
CO	40042	13654
CO	40049	11972
CO	40067	12822
CO	40117	12507
CO	40141	12935
CO	40148	13758
CO	40152	12855
CO	40171	13555
CO	40172	12583
CO	40175	13012
CO	40178	13205
CO	40188	12255
CO	40191	13438
CO	40193	12993
CO	40199	12662
CO	40202	13331
CO	40206	13049
CO	40210	13485
CO	40219	13412
CO	40222	12642
CO	40234	12052
CO	40235	13375
CO	40238	12554
CO	40241	12617
CO	40245	13658
CO	40262	12919
CO	40278	11781
CO	40289	13205
CO	40303	13136
CO	40314	13401
CO	40325	12579
CO	40334	12535
CO	40335	12774
CO	40340	13154
CO	40347	13413
CO	40352	13425
CO	40373	13286
CO	40375	12426
CO	40382	12976
CO	40399	12816
CO	40408	12026
CO	40412	12619
CO	40425	12900
CO	40426	11882
CO	40436	13343
CO	40442	12854
CO	40455	13034
CO	40474	13264
CO	40479	13337
CO	40484	13006
CO	40486	12465
CO	40488	13223
CO	40490	12449
CO	40495	13011
CO	40505	13513
CO	40510	13234
CO	40512	13181
CO	40522	12893
CO	40535	12662
CO	40549	12071

CO	40558	12871
CO	40568	12966
CO	40575	13213
CO	40586	13512
CO	40597	12877
CO	40610	13336
CO	40611	13356
CO	40619	12366
CO	40621	12827
CO	40629	13281
CO	40639	12950
CO	40640	12805
CO	40666	12936
CO	40678	13037
CO	40686	13526
CO	40696	12743
CO	40697	12803
CO	40718	13498
CO	40727	13070
CO	40740	13356
CO	40741	13149
CO	40743	12303
CO	40749	13506
CO	40753	12860
CO	40757	13000
CO	40778	13421
CO	40805	12721
CO	40827	12095
CO	40834	12603
CO	40846	12870
CO	40861	12831
CO	40875	13121
CO	40884	12838
CO	40890	12212
CO	40911	13182
CO	40912	12639
CO	40917	13240
CO	40919	12679
CO	40921	12976
CO	40925	13649
CO	40932	13690
CO	40960	12685
CO	40962	13163
CO	40968	13106
CO	40976	13653
CO	40984	12650
CO	40988	13545
CO	40992	13047
CO	41001	12814
CO	41006	13236
CO	41008	13095
CO	41013	12517
CO	41015	11989
CO	41016	12275
CO	41022	12304
CO	41035	13380
CO	41048	13162
CO	41049	12817
CO	41050	12547
CO	41054	13427
CO	41058	13221
CO	41060	13048
CO	41061	13512
CO	41068	13210
CO	41078	12782
CO	41079	12569
CO	41080	13477

CO	41088	12693
CO	41101	12812
CO	41105	12675
CO	41110	12431
CO	41114	12886
CO	41123	13359
CO	41163	11808
CO	41199	12681
CO	41209	12613
CO	41227	13035
CO	41235	13329
CO	41250	13077
CO	41275	13107
CO	41284	12914
CO	41285	13280
CO	41288	12791
CO	41293	13671
CO	41301	12540
CO	41305	13139
CO	41369	12796
CO	41371	12494
CO	41373	12499
CO	41399	13271
CO	41444	11725
CO	41480	12687
CO	41535	13018
CO	41546	14116
CO	41552	12088
CO	41578	12204
CO	41631	13200
CO	41636	12480
CO	41637	12381
CO	41639	12642
CO	41657	13326
CO	41730	12500
CO	41745	13357
CO	41755	12192
CO	41760	13196
CO	41779	12176
CO	41840	12732
CO	41854	12592
CO	41868	12853
CO	41881	12917
CO	41886	12640
CO	41904	13676
CO	41908	13243
CO	41923	13474
CO	41925	12951
CO	41931	13177
CO	41938	12899
CO	41946	13070
CO	41992	12070
CO	42065	12681
CO	42075	12617
CO	42081	12552
CO	42091	13690
CO	42095	12648
CO	42104	13062
CO	42131	13007
CO	42149	13535
CO	42150	11713
CO	42189	12147
CO	42236	12870
CO	42247	12505
CO	42358	12512
CO	42400	12024
CO	42425	12052

CO	42432	12851
CO	42436	12483
CO	42479	13126
CO	42505	12750
CO	42743	12203
CO	42814	12379
CO	42959	13637
CO	43227	12790
CO	43705	11875
CO	43780	12773
CO	43975	12216
CO	44153	12567
CO	44160	12502
CO	44167	12047
CO	44182	12198
CO	44212	12939
CO	44224	12760
CO	44235	12152
CO	44243	12453
CO	44257	12301
CO	44277	12482
CO	44280	12173
CO	44299	12763
CO	44308	12099
CO	44316	12298
CO	44346	12628
CO	44363	12314
CO	44383	12225
CO	44422	12217
CO	44432	12182
CO	44452	12258
CO	44482	12374
CO	44501	12226
CO	44511	12517
CO	44516	12020
CO	44533	12046
CO	44534	12433
CO	44552	12284
CO	44553	11846
CO	44554	12245
CO	44562	12470
CO	44565	12629
CO	44574	12023
CO	44590	12363
CO	44604	12248
CO	44608	12141
CO	44633	12070
CO	44644	11997
CO	44647	12387
CO	44651	11832
CO	44673	12244
CO	44681	12256
CO	44702	11721
CO	44703	12516
CO	44743	12437
CO	44744	11926
CO	44745	12663
CO	44751	12451
CO	44757	12376
CO	44767	12145
CO	44770	12280
CO	44778	12161
CO	44789	12610
CO	44792	12652
CO	44816	12506
CO	44823	12546
CO	44826	12119

CO	44829	11921
CO	44860	12672
CO	44896	12817
CO	44910	12287
CO	44911	12319
CO	44912	12006
CO	44928	12274
CO	44958	12234
CO	44969	12489
CO	44997	12458
CO	45005	12482
CO	45008	12481
CO	45015	11970
CO	45016	12162
CO	45052	12902
CO	45053	12157
CO	45105	12111
CO	45118	12389
CO	45130	12622
CO	45131	12324
CO	45158	12605
CO	45161	13211
CO	45167	12318
CO	45210	12518
CO	45228	12223
CO	45230	12363
CO	45238	12054
CO	45269	12645
CO	45286	12572
CO	45299	12570
CO	45300	12440
CO	45304	12425
CO	45308	12213
CO	45311	12826
CO	45320	12771
CO	45321	12448
CO	45324	12648
CO	45326	12284
CO	45343	12492
CO	45350	12091
CO	45352	12200
CO	45385	12428
CO	45404	12068
CO	45427	12385
CO	45438	13086
CO	45440	12290
CO	45474	13273
CO	45481	12407
CO	45483	11885
CO	45493	12348
CO	45500	12119
CO	45507	12387
CO	45508	12706
CO	45513	13096
CO	45523	12855
CO	45531	12753
CO	45536	12074
CO	45542	13549
CO	45543	12490
CO	45549	12360
CO	45563	12413
CO	45564	11767
CO	45587	12739
CO	45601	12312
CO	45626	12348
CO	45628	12270
CO	45636	12174

CO	45679	12330
CO	45695	12271
CO	45720	12515
CO	45735	12240
CO	45744	12485
CO	45786	12925
CO	45803	12496
CO	45828	12101
CO	45837	12155
CO	45844	12500
CO	45861	12172
CO	45870	12151
CO	45878	12671
CO	45879	12304
CO	45887	12563
CO	45988	12266
CO	46067	13288
CO	46157	12726
CO	46162	12962
CO	46189	13545
CO	46190	13167
CO	46215	12904
CO	46259	11886
CO	46293	12891
CO	46342	12407
CO	46372	12559
CO	46398	12435
CO	46450	12769
CO	46463	13109
CO	46464	12885
CO	46516	12973
CO	46562	13416
CO	46568	12272
CO	46607	12667
CO	46612	13407
CO	46667	13378
CO	46682	12682
CO	46706	12021
CO	46718	12472
CO	46720	12455
CO	46779	12462
CO	46868	11845
CO	46981	13048
CO	47128	13213
CO	47130	12852
CO	47243	13096
CO	47248	13299
CO	47276	12842
CO	47320	12874
CO	47345	12472
CO	47361	12795
CO	47419	12346
CO	47425	12711
CO	47551	12961
CO	47564	12758
CO	47616	12618
CO	47628	12487
CO	47638	12328
CO	47666	12519
CO	47672	13157
CO	47724	13431
CO	47756	13155
CO	47761	12800
CO	47806	13319
CO	47821	13097
CO	47832	12895
CO	47841	12035

CO	47878	13236
CO	47963	12490
CO	48027	12973
CO	48075	12919
CO	48213	13320
CO	48279	13411
CO	48423	13454
CO	48497	13147
CO	48536	13435
CO	48581	13218
CO	48710	12836
CO	48819	12711
CO	48821	13514
CO	48936	12800
CO	49052	13283
CO	49101	12163
CO	49167	13319
CO	49178	13574
CO	49187	13388
CO	49213	11725
CO	49268	13450
CO	49312	11787
CO	49325	13183
CO	49395	13255
CO	49446	13435
CO	49493	13309
CO	49532	12695
CO	49637	13541
CO	49766	13421
CO	49990	12485
CO	50043	11723
CO	50073	11880
CO	50102	11970
CO	50464	12720
CO	50564	12894
CO	50569	12351
CO	50574	11930
CO	507	12269
CO	50707	12704
CO	50725	11744
CO	50847	11787
CO	51431	12040
CO	51571	12088
CO	51836	13386
CO	51929	11941
CO	52401	12497
CO	52457	12155
CO	52737	11812
CO	53292	12412
CO	53368	12108
CO	53583	12029
CO	53672	12785
CO	53704	12016
CO	53818	11986
CO	53827	13152
CO	54850	12133
CO	54955	12330
CO	55347	13077
CO	5538	13273
CO	55518	13217
CO	55774	12582
CO	56113	13119
CO	56168	12366
CO	56623	12435
CO	57019	11877
CO	57357	13622
CO	57583	12210

CO	57616	12717
CO	57707	12209
CO	58114	11983
CO	58369	12294
CO	58422	12126
CO	58446	12192
CO	58534	12322
CO	58535	12502
CO	58556	11866
CO	58567	13345
CO	58579	12807
CO	58597	12406
CO	58619	13227
CO	58625	12922
CO	58646	12482
CO	58667	11886
CO	58691	11751
CO	58886	12494
CO	58971	12959
CO	58982	11777
CO	59027	12273
CO	59070	12526
CO	59071	11912
CO	59105	11768
CO	59127	13272
CO	59195	12148
CO	59198	13108
CO	59212	11672
CO	59247	11972
CO	59259	12141
CO	59279	13197
CO	59284	12274
CO	59299	12394
CO	59317	12156
CO	59321	11750
CO	59396	11675
CO	59404	11687
CO	59420	12488
CO	59421	13018
CO	59454	13467
CO	59463	13044
CO	59486	11956
CO	59560	12935
CO	59566	13225
CO	59571	12051
CO	59636	12410
CO	59710	11816
CO	59718	12001
CO	59725	12736
CO	59740	11984
CO	59754	11767
CO	59760	12537
CO	59786	12577
CO	59791	12253
CO	59826	11904
CO	59837	12457
CO	59875	12761
CO	59890	11804
CO	59903	12504
CO	59904	12822
CO	59915	13304
CO	59959	12717
CO	59960	12494
CO	59983	12022
CO	60031	12257
CO	60050	12359
CO	60065	12116

CO	60071	13263
CO	60100	12029
CO	60119	12429
CO	60205	12649
CO	60278	12120
CO	60281	12735
CO	60301	11764
CO	60315	12300
CO	60351	11741
CO	60378	13265
CO	60405	12440
CO	60449	11943
CO	60475	13166
CO	60492	11714
CO	60523	13594
CO	60560	12119
CO	60672	12229
CO	60741	12505
CO	60769	11805
CO	60808	12968
CO	60837	12344
CO	60840	11828
CO	60851	11892
CO	60910	12369
CO	60916	12827
CO	60926	12743
CO	60961	12280
CO	61013	12640
CO	61062	12162
CO	61082	12415
CO	61087	13145
CO	61094	13558
CO	61105	12622
CO	61133	12171
CO	61191	12449
CO	61290	11746
CO	61336	12017
CO	61355	13031
CO	61385	12087
CO	61473	13260
CO	61538	11845
CO	61557	11866
CO	61612	13008
CO	61693	12427
CO	61699	13320
CO	61764	12832
CO	61774	12049
CO	61810	12886
CO	61909	13285
CO	61913	12070
CO	61965	13793
CO	61966	13120
CO	62019	12797
CO	62280	13206
CO	62320	12087
CO	62348	13252
CO	62391	12725
CO	62417	12631
CO	62419	11975
CO	62424	12691
CO	62439	12604
CO	62450	13108
CO	62476	13629
CO	62496	13498
CO	62512	12153
CO	62538	12500
CO	62664	13797

CO	62669	12090
CO	62724	11676
CO	62760	12302
CO	62790	12321
CO	62812	11918
CO	62819	12130
CO	62825	12560
CO	63187	11933
CO	63573	13008
CO	64568	12633
CO	64654	11751
CO	65912	11894
CO	66605	12308
CO	69046	12758
CO	69319	12649
CO	69417	12463
CO	69519	12266
CO	69633	11732
CO	69681	11884
CO	69770	13752
CO	69992	11924
CO	70051	11899
CO	70082	13367
CO	70118	12482
CO	70289	12934
CO	70425	12662
CO	70485	12711
CO	70579	12663
CO	70606	12701
CO	70639	12613
CO	70669	12620
CO	70838	12020
CO	70892	12258
CO	70902	11777
CO	70911	13374
CO	70956	12007
CO	70970	12710
CO	71056	12838
CO	71105	12160
CO	71159	11871
CO	71178	11819
CO	71190	13010
CO	71202	13205
CO	71261	12419
CO	71526	11881
CO	71601	12271
CO	71623	11738
CO	71919	12018
CO	72061	12726
CO	72201	12664
CO	72285	12878
CO	72329	12322
CO	72587	12305
CO	72761	12711
CO	72839	13416
CO	73923	12801
CO	74031	12838
CO	74285	12986
CO	74655	11981
CO	74689	12095
CO	74948	12550
CO	75099	12674
CO	75605	12253
CO	75644	12807
CO	75878	13002
CO	76572	12997
CO	7917	12216

CO	8300	13253
CO	8663	12180
CO	9103	12759
CO	9140	12197
CO	9232	12743
CO	9359	12169
CO	9380	12967
CO	9418	13016
CO	9623	12772
CO	9681	12275
CO	9742	12943
CO_PMA	10173	11943
CO_PMA	10344	14123
CO_PMA	10400	11498
CO_PMA	10672	1
CO_PMA	10804	12146
CO_PMA	11137	12670
CO_PMA	11657	11894
CO_PMA	11757	13437
CO_PMA	12094	13115
CO_PMA	12326	1
CO_PMA	12529	1
CO_PMA	13088	11709
CO_PMA	13769	11523
CO_PMA	14055	11532
CO_PMA	14194	12055
CO_PMA	14545	11529
CO_PMA	16384	12412
CO_PMA	28504	13345
CO_PMA	28641	13073
CO_PMA	28691	12293
CO_PMA	28754	12589
CO_PMA	28961	11590
CO_PMA	29382	1
CO_PMA	29693	11892
CO_PMA	29743	12633
CO_PMA	29861	1
CO_PMA	30142	11929
CO_PMA	30177	12916
CO_PMA	30430	13197
CO_PMA	30513	12573
CO_PMA	30652	13013
CO_PMA	30734	12555
CO_PMA	30787	1
CO_PMA	30849	13287
CO_PMA	30996	12760
CO_PMA	31163	11612
CO_PMA	31218	12448
CO_PMA	31464	1
CO_PMA	31702	12178
CO_PMA	31823	13325
CO_PMA	32200	11894
CO_PMA	32344	13429
CO_PMA	32411	12582
CO_PMA	32497	1
CO_PMA	33258	12806
CO_PMA	33873	12643
CO_PMA	34161	1
CO_PMA	34318	11700
CO_PMA	34389	12911
CO_PMA	34438	12071
CO_PMA	34708	12920
CO_PMA	35009	12781
CO_PMA	35030	13334
CO_PMA	35042	1
CO_PMA	35066	12390
CO_PMA	35165	13478

CO_PMA	35235	13848
CO_PMA	35240	12368
CO_PMA	35254	12667
CO_PMA	35383	1
CO_PMA	35512	11867
CO_PMA	35819	13134
CO_PMA	35860	11402
CO_PMA	36035	11878
CO_PMA	36091	12653
CO_PMA	36137	13103
CO_PMA	36510	12104
CO_PMA	36693	11661
CO_PMA	37076	12146
CO_PMA	37097	11769
CO_PMA	37253	11950
CO_PMA	37566	12054
CO_PMA	39733	12133
CO_PMA	40158	13425
CO_PMA	40159	12610
CO_PMA	40162	12063
CO_PMA	40182	11464
CO_PMA	40264	13159
CO_PMA	40550	11425
CO_PMA	40685	13214
CO_PMA	40925	11566
CO_PMA	41012	12734
CO_PMA	46189	12280
CO_PMA	46244	11713
CO_PMA	46420	12849
CO_PMA	46527	11499
CO_PMA	46793	12676
CO_PMA	46981	11418
CO_PMA	47528	11973
CO_PMA	47806	12167
CO_PMA	47819	11462
CO_PMA	48213	13413
CO_PMA	48215	13204
CO_PMA	48279	13433
CO_PMA	48318	12927
CO_PMA	48364	13171
CO_PMA	48388	13471
CO_PMA	48423	13208
CO_PMA	48440	13247
CO_PMA	48497	13191
CO_PMA	48536	13611
CO_PMA	48581	13364
CO_PMA	48645	12513
CO_PMA	48660	12788
CO_PMA	48662	13373
CO_PMA	48675	13193
CO_PMA	48690	11855
CO_PMA	48691	13274
CO_PMA	48784	12851
CO_PMA	48805	13152
CO_PMA	48819	12987
CO_PMA	48821	13435
CO_PMA	48825	11817
CO_PMA	48851	13156
CO_PMA	48861	13016
CO_PMA	48936	12691
CO_PMA	48975	12973
CO_PMA	48981	12819
CO_PMA	49022	13029
CO_PMA	49050	12530
CO_PMA	49052	13310
CO_PMA	49107	12819
CO_PMA	49129	13591

CO_PMA	49160	13405
CO_PMA	49167	13427
CO_PMA	49168	13148
CO_PMA	49172	13538
CO_PMA	49178	13760
CO_PMA	49187	13443
CO_PMA	49214	13583
CO_PMA	49265	13419
CO_PMA	49268	12990
CO_PMA	49305	12680
CO_PMA	49325	13436
CO_PMA	49357	13373
CO_PMA	49365	13631
CO_PMA	49366	13371
CO_PMA	49395	13310
CO_PMA	49446	13611
CO_PMA	49493	13547
CO_PMA	49495	13413
CO_PMA	49532	12717
CO_PMA	49591	13709
CO_PMA	49623	13216
CO_PMA	49637	13692
CO_PMA	49669	13062
CO_PMA	49688	12845
CO_PMA	49864	12941
CO_PMA	49874	12003
CO_PMA	49912	13176
CO_PMA	49937	13563
CO_PMA	54344	12186
CO_PMA	56900	12992
CO_PMA	57982	12964
CO_PMA	58435	13602
CO_PMA	59560	11753
CO_PMA	59792	12367
CO_PMA	59915	12362
CO_PMA	59989	14218
CO_PMA	60042	14106
CO_PMA	60211	11809
CO_PMA	60315	12921
CO_PMA	60332	12147
CO_PMA	60348	13629
CO_PMA	60395	13261
CO_PMA	60398	12292
CO_PMA	60568	12338
CO_PMA	60579	12306
CO_PMA	60732	13065
CO_PMA	60770	11769
CO_PMA	60826	12131
CO_PMA	60827	13239
CO_PMA	60902	11444
CO_PMA	60931	12597
CO_PMA	60970	11765
CO_PMA	61060	13070
CO_PMA	61102	11774
CO_PMA	61127	12292
CO_PMA	61158	13249
CO_PMA	61302	12837
CO_PMA	61327	12426
CO_PMA	61333	11811
CO_PMA	61965	11843
CO_PMA	62194	12427
CO_PMA	62220	11663
CO_PMA	62371	12227
CO_PMA	62496	11756
CO_PMA	62578	11694
CO_PMA	70828	11793
CO_PMA	7682	13023

CO_PMA	7899	1
CO_PMA	8070	12447
CO_PMA	8086	13833
CO_PMA	8217	12738
CO_PMA	8385	13005
CO_PMA	8546	11761
CO_PMA	8845	11981
CO_PMA	9071	12533
CO_PMA	9082	12746
CO_PMA	9103	11818
CO_PMA	9176	1
CO_PMA	9579	11467
CO_PMA	9619	11578
CR5	10672	1
CR5	10744	12433
CR5	10870	12339
CR5	1095	12607
CR5	11150	13311
CR5	11657	13230
CR5	11724	12715
CR5	1193	11637
CR5	12217	13228
CR5	12326	1
CR5	12912	11779
CR5	1440	12117
CR5	14545	12214
CR5	15581	12476
CR5	15685	13705
CR5	160	11889
CR5	16030	11899
CR5	16221	12651
CR5	1638	11837
CR5	16384	12506
CR5	16495	11828
CR5	16819	11761
CR5	17229	12324
CR5	17427	11815
CR5	17447	12614
CR5	17661	11996
CR5	17677	12227
CR5	17726	12282
CR5	17744	12028
CR5	17765	13759
CR5	17792	11699
CR5	17828	12203
CR5	17852	12307
CR5	17893	12779
CR5	17943	11766
CR5	17964	12249
CR5	18113	11847
CR5	18175	12365
CR5	18222	12241
CR5	18252	12119
CR5	18254	12380
CR5	18281	11633
CR5	2058	11686
CR5	20652	12783
CR5	21155	11985
CR5	2281	12341
CR5	24427	12205
CR5	24509	13296
CR5	24700	12035
CR5	24841	11784
CR5	24857	12148
CR5	24978	12593
CR5	25077	11714
CR5	25139	12653

CR5	25637	12284
CR5	25901	13295
CR5	25914	12021
CR5	26109	11678
CR5	26227	12765
CR5	26269	11708
CR5	26288	13720
CR5	26371	12470
CR5	26599	12146
CR5	26611	11761
CR5	26768	11934
CR5	26984	12000
CR5	27392	12183
CR5	27536	12066
CR5	28159	12607
CR5	28655	13034
CR5	28664	12234
CR5	28754	14249
CR5	28768	12409
CR5	28989	12157
CR5	29901	13055
CR5	29909	13635
CR5	30016	12156
CR5	30017	13905
CR5	30421	13719
CR5	30787	1
CR5	30922	12886
CR5	31080	13365
CR5	31205	12316
CR5	32200	13230
CR5	32357	14157
CR5	32607	13698
CR5	33058	13828
CR5	33300	12993
CR5	33488	14094
CR5	3369	13326
CR5	33892	13248
CR5	34124	13744
CR5	34240	13491
CR5	34438	13979
CR5	34464	13993
CR5	34741	12656
CR5	3494	12242
CR5	35066	13975
CR5	35183	13995
CR5	35227	12539
CR5	35586	13729
CR5	35629	14189
CR5	35716	13095
CR5	36142	13725
CR5	36542	13663
CR5	36754	12844
CR5	36763	13539
CR5	36770	13549
CR5	36785	13892
CR5	36787	12524
CR5	36803	14195
CR5	36804	13711
CR5	36823	12223
CR5	36837	13007
CR5	36867	13843
CR5	36880	12178
CR5	36895	13758
CR5	36906	13474
CR5	36908	13104
CR5	36931	12166
CR5	36933	13808

CR5	36937	14042
CR5	36940	12317
CR5	36943	13956
CR5	36949	12791
CR5	36955	13365
CR5	36980	13797
CR5	37004	13481
CR5	37007	13113
CR5	37029	14079
CR5	37046	13568
CR5	37071	13709
CR5	37086	12254
CR5	37089	14041
CR5	37129	14166
CR5	37151	14019
CR5	37161	13747
CR5	37170	12791
CR5	37172	12043
CR5	37178	12966
CR5	37180	13117
CR5	37183	12931
CR5	3719	11769
CR5	37199	13051
CR5	37226	12884
CR5	37247	13381
CR5	37264	11960
CR5	37268	12108
CR5	37293	12607
CR5	37315	13958
CR5	37329	13165
CR5	37358	14085
CR5	3736	11651
CR5	37365	14230
CR5	37394	12630
CR5	37398	12567
CR5	37429	13296
CR5	37449	12985
CR5	37494	13110
CR5	37536	12357
CR5	37576	13205
CR5	37579	13213
CR5	37622	13291
CR5	37633	12562
CR5	37679	12116
CR5	37688	13196
CR5	37726	14145
CR5	37735	12476
CR5	37737	12680
CR5	3774	11859
CR5	37746	12487
CR5	37763	12610
CR5	37779	11821
CR5	37783	13396
CR5	37813	13058
CR5	37814	12241
CR5	37820	12856
CR5	37833	11831
CR5	37835	12522
CR5	37838	12801
CR5	37860	12184
CR5	37877	13816
CR5	37881	13071
CR5	37920	12410
CR5	37921	12530
CR5	37948	12689
CR5	37996	12979
CR5	38020	13720

CR5	38099	12238
CR5	38101	12772
CR5	38103	12303
CR5	38108	12313
CR5	38165	11737
CR5	38168	13445
CR5	38210	12774
CR5	38270	12408
CR5	38303	12851
CR5	38331	13622
CR5	38337	13512
CR5	38393	13134
CR5	38394	12461
CR5	38433	13058
CR5	38499	13084
CR5	38628	13179
CR5	38646	12389
CR5	38679	13169
CR5	38726	13190
CR5	38746	12279
CR5	38907	13296
CR5	38920	12216
CR5	38958	13267
CR5	38996	13100
CR5	39027	13571
CR5	39054	11943
CR5	39092	12347
CR5	39124	12676
CR5	39208	12921
CR5	3925	12673
CR5	39343	13344
CR5	39401	12215
CR5	39473	12919
CR5	39484	12664
CR5	39549	14089
CR5	39559	13023
CR5	39588	13429
CR5	39650	13622
CR5	39654	12855
CR5	39719	12786
CR5	39774	13430
CR5	39833	13070
CR5	39926	12904
CR5	40042	13083
CR5	40055	11890
CR5	40076	13114
CR5	40101	12776
CR5	4011	11886
CR5	40117	12099
CR5	40145	13128
CR5	40148	12805
CR5	40152	12749
CR5	40156	12898
CR5	40158	14650
CR5	40159	13918
CR5	40170	13964
CR5	40171	13931
CR5	40172	12651
CR5	40191	12843
CR5	40193	13193
CR5	40199	12352
CR5	40206	12170
CR5	40210	13829
CR5	40216	14071
CR5	40220	13517
CR5	40224	13064
CR5	40235	13743

CR5	40241	13214
CR5	40245	13293
CR5	40262	12837
CR5	40303	13255
CR5	40304	13035
CR5	40333	13988
CR5	40334	12826
CR5	40337	12401
CR5	40340	13119
CR5	40356	13734
CR5	40382	12136
CR5	40389	13564
CR5	40394	14376
CR5	40402	13394
CR5	40415	12432
CR5	40416	12230
CR5	40427	12620
CR5	40455	12708
CR5	40474	13426
CR5	40488	12444
CR5	40495	12244
CR5	40505	12998
CR5	40512	13124
CR5	40518	13980
CR5	40522	12516
CR5	40526	12725
CR5	40529	13716
CR5	40531	13710
CR5	40535	12383
CR5	40558	11822
CR5	40577	12978
CR5	40578	13781
CR5	40583	12401
CR5	40586	13249
CR5	40587	12452
CR5	40610	13139
CR5	40666	12574
CR5	40676	13104
CR5	40686	13744
CR5	40705	12571
CR5	40716	13168
CR5	40723	13523
CR5	40727	13146
CR5	40749	13192
CR5	40766	13966
CR5	40778	13085
CR5	40827	11876
CR5	40867	12832
CR5	40875	12361
CR5	40876	13194
CR5	40893	13890
CR5	40898	13254
CR5	40908	13753
CR5	40912	12952
CR5	40920	12006
CR5	4093	11784
CR5	40932	14061
CR5	40945	12822
CR5	40968	12546
CR5	40976	13355
CR5	40979	12814
CR5	40984	12263
CR5	40996	13785
CR5	41001	12151
CR5	41006	13460
CR5	41008	12225
CR5	41018	13583

CR5	41022	11748
CR5	41035	12591
CR5	41049	12942
CR5	41068	13589
CR5	41080	13145
CR5	41088	12480
CR5	41105	12145
CR5	41114	12443
CR5	41199	12446
CR5	41204	13300
CR5	41227	12928
CR5	41235	13021
CR5	41237	11907
CR5	41253	12238
CR5	41272	12436
CR5	41284	12408
CR5	41285	13001
CR5	41293	13291
CR5	41368	12732
CR5	41371	12099
CR5	41373	12255
CR5	41460	12261
CR5	41474	12364
CR5	41489	11768
CR5	41491	12087
CR5	41493	11925
CR5	41546	13948
CR5	41631	12681
CR5	41636	11839
CR5	41639	12484
CR5	41657	12799
CR5	41721	12864
CR5	41745	12836
CR5	41749	11768
CR5	41753	12956
CR5	41770	12958
CR5	41840	12559
CR5	41854	11918
CR5	41881	12818
CR5	41886	12388
CR5	41900	12147
CR5	41904	13376
CR5	41908	12603
CR5	41923	13423
CR5	41946	12568
CR5	42004	13007
CR5	42038	13551
CR5	42075	12780
CR5	42081	12759
CR5	42104	12091
CR5	42131	12597
CR5	42149	13014
CR5	42236	12409
CR5	42295	12879
CR5	42337	12576
CR5	42357	12667
CR5	42479	12754
CR5	42694	13307
CR5	42818	13240
CR5	42959	13063
CR5	43416	12642
CR5	43697	11613
CR5	43749	13210
CR5	43752	12456
CR5	43927	11774
CR5	44151	12162
CR5	44160	12269

CR5	44167	11881
CR5	4421	11876
CR5	44212	12770
CR5	44221	12409
CR5	44257	12212
CR5	44299	12630
CR5	44346	12334
CR5	44363	12153
CR5	44452	11930
CR5	44501	11829
CR5	44554	11852
CR5	44565	12397
CR5	44574	11729
CR5	44633	11745
CR5	44647	12130
CR5	44666	11943
CR5	4467	11825
CR5	44673	11954
CR5	44703	12216
CR5	44721	13272
CR5	44744	11634
CR5	44751	12203
CR5	44770	11953
CR5	44792	12450
CR5	44816	12337
CR5	44823	12273
CR5	44826	11970
CR5	44829	11745
CR5	44860	12392
CR5	44884	12309
CR5	44896	12636
CR5	44904	11728
CR5	44910	12302
CR5	44911	12080
CR5	44957	12102
CR5	44958	11786
CR5	4497	11830
CR5	45005	12244
CR5	45016	11837
CR5	45105	11727
CR5	45111	11740
CR5	45118	12280
CR5	45130	12468
CR5	45131	12061
CR5	45158	12366
CR5	45161	12849
CR5	45188	12749
CR5	45297	12070
CR5	45300	12198
CR5	45311	12256
CR5	45320	12542
CR5	45326	11931
CR5	45350	11699
CR5	45352	12018
CR5	45357	12550
CR5	45385	12376
CR5	45404	11762
CR5	45427	11966
CR5	45438	12892
CR5	45481	12110
CR5	45483	11624
CR5	45493	11962
CR5	45513	12814
CR5	45523	12683
CR5	45531	12549
CR5	45536	11840
CR5	45542	13683

CR5	45563	12258
CR5	45587	12528
CR5	45626	12067
CR5	45628	12060
CR5	45679	12008
CR5	45695	11951
CR5	45720	12217
CR5	45744	12149
CR5	45844	12235
CR5	45870	11856
CR5	45878	12446
CR5	45879	11974
CR5	45914	13629
CR5	45951	13456
CR5	45955	12891
CR5	46162	13683
CR5	46189	14186
CR5	46190	13862
CR5	46259	12316
CR5	46372	12106
CR5	46398	12696
CR5	46532	13722
CR5	46548	11770
CR5	46562	14152
CR5	46661	12840
CR5	46668	11896
CR5	46706	12987
CR5	46720	13629
CR5	46733	13839
CR5	46738	12586
CR5	46886	13635
CR5	46891	13753
CR5	46913	12994
CR5	46981	13494
CR5	47034	13650
CR5	47114	13306
CR5	47135	13068
CR5	47175	13644
CR5	47188	13025
CR5	47445	13431
CR5	47476	13066
CR5	47616	13128
CR5	47626	12778
CR5	47628	12229
CR5	47756	13463
CR5	47806	13596
CR5	47832	13596
CR5	47878	13680
CR5	47993	13752
CR5	48021	12225
CR5	48075	12811
CR5	48830	13302
CR5	48912	11896
CR5	49397	12999
CR5	4998	11749
CR5	50031	12286
CR5	50184	12641
CR5	50477	12420
CR5	507	11881
CR5	50847	12097
CR5	50864	13552
CR5	50911	12707
CR5	51676	12945
CR5	51836	13333
CR5	5272	12323
CR5	52805	11813
CR5	52935	13210

CR5	53678	13049
CR5	53679	12312
CR5	53967	12775
CR5	54147	12150
CR5	54684	13169
CR5	5538	13377
CR5	57357	13077
CR5	57583	11718
CR5	57878	12297
CR5	58123	13022
CR5	58316	12342
CR5	58369	11801
CR5	58484	12626
CR5	58567	13021
CR5	58588	12352
CR5	58728	11944
CR5	58818	12184
CR5	59047	12124
CR5	59224	12589
CR5	59247	11771
CR5	59318	11684
CR5	59404	11613
CR5	59421	12764
CR5	59547	12773
CR5	59560	12718
CR5	59571	11790
CR5	59725	12867
CR5	59796	11722
CR5	59837	12133
CR5	59875	12693
CR5	59915	13172
CR5	60119	12403
CR5	60205	12112
CR5	60246	12613
CR5	60281	12924
CR5	60307	11854
CR5	60348	13233
CR5	60374	12750
CR5	60378	12990
CR5	60405	11861
CR5	60467	13529
CR5	60475	12906
CR5	60523	13498
CR5	60564	11733
CR5	60732	12601
CR5	60808	13029
CR5	60837	12397
CR5	60858	11623
CR5	60916	12654
CR5	61013	12707
CR5	61082	11731
CR5	61133	11858
CR5	61191	12206
CR5	6129	12462
CR5	61325	12813
CR5	61355	12932
CR5	61473	12978
CR5	61835	13260
CR5	61909	12986
CR5	6203	11896
CR5	6211	12825
CR5	62439	11662
CR5	62769	12723
CR5	63400	11835
CR5	63573	13245
CR5	63864	12770
CR5	64220	12776

CR5	64623	11875
CR5	64734	12371
CR5	65875	12717
CR5	66715	12265
CR5	67542	11698
CR5	68573	12789
CR5	69286	11760
CR5	69681	11821
CR5	69770	13959
CR5	69931	12920
CR5	70043	12593
CR5	70179	13197
CR5	70180	12690
CR5	70417	12026
CR5	70606	12865
CR5	70712	13083
CR5	70850	13243
CR5	70911	13164
CR5	70959	13196
CR5	71020	13282
CR5	71056	12726
CR5	71075	13319
CR5	71202	13981
CR5	71274	12528
CR5	71601	12122
CR5	71820	12977
CR5	71919	12123
CR5	72006	11728
CR5	72061	12242
CR5	72132	13585
CR5	72197	12573
CR5	72201	13024
CR5	72285	12961
CR5	72563	12172
CR5	72592	13179
CR5	72884	12476
CR5	72956	12587
CR5	73168	12009
CR5	73491	12616
CR5	73514	13876
CR5	74031	12802
CR5	74065	12255
CR5	74285	12571
CR5	74689	11997
CR5	75099	12184
CR5	75644	12611
CR5	8071	13711
CR5	8300	13013
CR5	9872	13193
CR5_PMA	11853	12309
CR5_PMA	12326	1
CR5_PMA	12529	1
CR5_PMA	28504	12431
CR5_PMA	28961	10857
CR5_PMA	30513	11870
CR5_PMA	30787	1
CR5_PMA	30849	12411
CR5_PMA	31218	12087
CR5_PMA	31464	1
CR5_PMA	32497	1
CR5_PMA	33895	10972
CR5_PMA	34161	1
CR5_PMA	35042	1
CR5_PMA	40182	11706
CR5_PMA	53690	11973
CR5_PMA	58993	13402
CR5_PMA	59143	11905

CR5_PMA	60425	1
CR5_PMA	61060	11866
CR5_PMA	62483	1
CR5_PMA	8961	1
CR5_PMA	9176	1
CR8	1007	12220
CR8	10344	13227
CR8	10400	12726
CR8	10409	12246
CR8	10657	12620
CR8	10672	1
CR8	10744	13124
CR8	10760	11845
CR8	10851	12033
CR8	10870	12642
CR8	1095	12643
CR8	11150	12923
CR8	11204	13854
CR8	11543	12126
CR8	11612	12635
CR8	11636	12534
CR8	11657	12706
CR8	11724	12931
CR8	11897	11565
CR8	11964	13913
CR8	12094	12822
CR8	12146	12123
CR8	12175	11581
CR8	12187	11584
CR8	12217	13125
CR8	12326	1
CR8	12392	12634
CR8	12476	13037
CR8	12530	12429
CR8	1257	11740
CR8	12654	12277
CR8	12745	11821
CR8	1277	12278
CR8	12809	12727
CR8	12870	12117
CR8	12912	11870
CR8	1302	12212
CR8	13162	13191
CR8	13274	12256
CR8	134	11756
CR8	13400	12774
CR8	13402	13124
CR8	13681	11532
CR8	13763	12079
CR8	13882	11616
CR8	13991	12708
CR8	14121	12523
CR8	14183	12913
CR8	14194	12634
CR8	14199	12391
CR8	1444	11758
CR8	14477	12274
CR8	14545	12124
CR8	14597	13266
CR8	14641	12678
CR8	14754	12870
CR8	14779	13275
CR8	1481	11749
CR8	15016	11841
CR8	15035	12532
CR8	15292	12519
CR8	15342	12373

CR8	15459	13207
CR8	15508	12327
CR8	15533	12038
CR8	15566	12049
CR8	15570	11656
CR8	15581	12914
CR8	15605	12317
CR8	15647	11763
CR8	1567	12909
CR8	15685	13938
CR8	15731	12091
CR8	158	12369
CR8	15831	11642
CR8	160	11937
CR8	16000	12480
CR8	1638	11662
CR8	16384	12601
CR8	16717	11539
CR8	17015	12326
CR8	1738	11618
CR8	17519	11988
CR8	17765	13155
CR8	17852	11557
CR8	1796	11889
CR8	18137	11744
CR8	182	11718
CR8	1844	12972
CR8	18761	11860
CR8	1884	12643
CR8	1891	12019
CR8	19470	12693
CR8	2022	11733
CR8	20652	12065
CR8	20782	12016
CR8	22140	12225
CR8	2281	11957
CR8	22993	12214
CR8	24363	12731
CR8	24509	12508
CR8	24544	12347
CR8	24602	12142
CR8	24700	11632
CR8	24978	12319
CR8	24986	12312
CR8	24996	11749
CR8	25078	11726
CR8	25221	12482
CR8	25325	11524
CR8	25341	12852
CR8	25425	12205
CR8	25637	11676
CR8	25654	11727
CR8	25901	12988
CR8	25950	11704
CR8	25994	12238
CR8	26041	11554
CR8	26288	12988
CR8	26371	11913
CR8	26393	11615
CR8	26462	12681
CR8	26514	11555
CR8	26768	11815
CR8	26984	11541
CR8	27392	11852
CR8	27490	12561
CR8	28113	12571
CR8	28754	13853

CR8	28819	13820
CR8	28952	13251
CR8	29901	12004
CR8	30017	13344
CR8	30645	12818
CR8	30787	1
CR8	30996	14124
CR8	31080	12367
CR8	31163	13327
CR8	31205	12185
CR8	31464	1
CR8	31479	13557
CR8	31655	13168
CR8	32200	12706
CR8	32607	12646
CR8	32857	13228
CR8	33058	13207
CR8	33570	11937
CR8	33576	13463
CR8	33646	12764
CR8	33688	13395
CR8	3369	13323
CR8	33847	13074
CR8	33873	13846
CR8	34240	12536
CR8	34246	13293
CR8	34438	13659
CR8	34464	13516
CR8	34741	12312
CR8	34803	13149
CR8	35066	13649
CR8	35183	12666
CR8	35716	12249
CR8	36089	13245
CR8	36142	12886
CR8	36231	12989
CR8	36381	13495
CR8	36762	13526
CR8	36772	13104
CR8	36773	12416
CR8	36785	13192
CR8	36787	11678
CR8	36803	13699
CR8	36804	12640
CR8	36834	12612
CR8	36837	12458
CR8	36867	12947
CR8	36869	12469
CR8	36895	12843
CR8	36900	13361
CR8	36906	12971
CR8	36930	12488
CR8	36937	13317
CR8	36943	13278
CR8	36957	12861
CR8	36960	13304
CR8	36983	11671
CR8	36992	13529
CR8	37046	12654
CR8	37071	13181
CR8	37089	13393
CR8	37097	14195
CR8	37107	13190
CR8	37124	12411
CR8	37125	13196
CR8	37129	13518
CR8	37151	13452

CR8	37166	11678
CR8	37170	12578
CR8	37172	12224
CR8	37178	12877
CR8	3719	11756
CR8	37193	13088
CR8	37198	13283
CR8	37199	12078
CR8	37201	12505
CR8	37226	12323
CR8	37232	13298
CR8	37247	12362
CR8	37255	13203
CR8	37259	12811
CR8	37268	11636
CR8	37280	13671
CR8	37293	12128
CR8	37300	13734
CR8	37315	13320
CR8	3736	11591
CR8	37364	11905
CR8	37365	13709
CR8	37393	12410
CR8	37394	12023
CR8	37403	12059
CR8	37407	12491
CR8	37429	12575
CR8	37536	11838
CR8	37562	12139
CR8	37576	12758
CR8	37609	11658
CR8	37611	12146
CR8	37619	13158
CR8	37630	11603
CR8	37671	12268
CR8	37679	11827
CR8	37688	12548
CR8	37691	12018
CR8	37726	13741
CR8	37735	12191
CR8	37737	12473
CR8	37768	12698
CR8	37779	11609
CR8	37813	12416
CR8	37835	12237
CR8	37841	11991
CR8	37860	11634
CR8	37868	12409
CR8	37877	13386
CR8	37902	12364
CR8	37907	12035
CR8	37921	12362
CR8	37924	11731
CR8	37943	12518
CR8	37955	11592
CR8	37984	11849
CR8	37996	12604
CR8	38001	13109
CR8	38020	13243
CR8	38094	12286
CR8	38115	12462
CR8	38137	12146
CR8	38210	12172
CR8	38272	12169
CR8	38281	12718
CR8	38349	12678
CR8	38371	12120

CR8	38393	12431
CR8	38432	13021
CR8	38433	12416
CR8	38438	12208
CR8	38444	11565
CR8	38449	12037
CR8	38506	13262
CR8	38524	12749
CR8	38628	13015
CR8	38640	12574
CR8	38646	12174
CR8	38726	12880
CR8	38808	12074
CR8	38848	12300
CR8	38920	12392
CR8	38958	12835
CR8	38959	11853
CR8	38996	13148
CR8	39019	11609
CR8	39027	13280
CR8	39060	11871
CR8	39092	11844
CR8	39112	12098
CR8	39124	12350
CR8	39208	12660
CR8	39225	12125
CR8	39242	11683
CR8	39248	12340
CR8	3925	12473
CR8	39401	12158
CR8	39415	12279
CR8	39446	12880
CR8	39484	12317
CR8	39549	13548
CR8	39559	12556
CR8	39588	12826
CR8	39606	12298
CR8	39607	12548
CR8	39644	12232
CR8	39650	13166
CR8	39654	12609
CR8	39661	11765
CR8	39733	13228
CR8	39746	11899
CR8	39909	12302
CR8	39910	12849
CR8	39926	12353
CR8	40014	11752
CR8	40042	13118
CR8	40055	12149
CR8	40076	12416
CR8	40126	12587
CR8	40148	12601
CR8	40156	12037
CR8	40158	14307
CR8	40159	13542
CR8	40164	11679
CR8	40170	13029
CR8	40172	12588
CR8	40191	12768
CR8	40193	13369
CR8	40199	12464
CR8	40206	11579
CR8	40209	12860
CR8	40210	13723
CR8	40220	12138
CR8	40241	12710

CR8	40245	13239
CR8	40262	12869
CR8	40264	14135
CR8	40278	12616
CR8	40286	12091
CR8	40303	13204
CR8	40333	14141
CR8	40334	12362
CR8	40335	12541
CR8	40356	13672
CR8	40358	12899
CR8	40378	12596
CR8	40382	11558
CR8	40394	14248
CR8	40407	12035
CR8	40408	12537
CR8	40413	12134
CR8	40419	12157
CR8	40426	12666
CR8	40443	11844
CR8	40488	12326
CR8	40492	12089
CR8	40495	12214
CR8	40503	11724
CR8	40505	12574
CR8	40512	12992
CR8	40518	14183
CR8	40522	11989
CR8	40549	11718
CR8	40592	11969
CR8	40597	13332
CR8	40620	12702
CR8	40651	11917
CR8	40676	12688
CR8	40678	11981
CR8	40686	13691
CR8	40716	13088
CR8	40719	13209
CR8	40733	12296
CR8	40741	12561
CR8	40749	12739
CR8	40774	11831
CR8	40779	12727
CR8	40792	13220
CR8	40797	11801
CR8	40805	12442
CR8	40827	11832
CR8	40838	11831
CR8	40853	13538
CR8	40875	11935
CR8	40882	12325
CR8	40892	12370
CR8	40893	13179
CR8	40898	13189
CR8	40912	13173
CR8	40919	12271
CR8	40920	11923
CR8	40929	12289
CR8	40932	13959
CR8	40958	12130
CR8	40968	12604
CR8	40983	12702
CR8	40984	11746
CR8	41001	11913
CR8	41006	13525
CR8	41008	12271
CR8	41012	13877

CR8	41016	12891
CR8	41048	12431
CR8	41050	12390
CR8	41058	12665
CR8	41068	13543
CR8	41079	12473
CR8	41080	13117
CR8	41085	13844
CR8	41088	12149
CR8	41101	12521
CR8	41103	11989
CR8	41114	12241
CR8	41119	11719
CR8	41171	12264
CR8	41227	12478
CR8	41275	12462
CR8	41284	12197
CR8	41285	13065
CR8	41293	12766
CR8	41305	12291
CR8	41373	12181
CR8	41425	12061
CR8	41462	12209
CR8	41474	12045
CR8	41490	13091
CR8	41491	12175
CR8	41493	11861
CR8	41499	11922
CR8	41500	13258
CR8	41502	12580
CR8	41517	12081
CR8	41531	13008
CR8	41535	12194
CR8	41546	13720
CR8	41601	11778
CR8	41631	12658
CR8	41652	12391
CR8	41657	12548
CR8	41712	12416
CR8	41721	12676
CR8	41733	11918
CR8	41745	12294
CR8	41749	11686
CR8	41753	12886
CR8	41755	11753
CR8	41840	12293
CR8	41851	12798
CR8	41853	11612
CR8	41854	11835
CR8	41881	12287
CR8	41886	11908
CR8	41904	12976
CR8	41908	12781
CR8	41923	13380
CR8	41938	11906
CR8	41946	12337
CR8	41978	12935
CR8	42038	12866
CR8	42065	11805
CR8	42075	11782
CR8	42081	12304
CR8	42097	12149
CR8	42104	11931
CR8	42131	12691
CR8	42149	12965
CR8	42173	13075
CR8	42189	11602

CR8	42208	11811
CR8	42218	12273
CR8	42236	12303
CR8	42253	11567
CR8	42295	12584
CR8	42417	12245
CR8	42426	11742
CR8	42432	11712
CR8	42470	12082
CR8	42472	11877
CR8	42479	12218
CR8	42497	11753
CR8	42505	11563
CR8	42582	11794
CR8	42622	12064
CR8	42676	13089
CR8	42818	13205
CR8	42908	11595
CR8	42959	13052
CR8	42997	11648
CR8	43095	13271
CR8	43227	12694
CR8	43697	11680
CR8	43705	11685
CR8	43780	12992
CR8	44033	13139
CR8	44151	12229
CR8	44160	12348
CR8	44162	12676
CR8	44165	12272
CR8	44182	12104
CR8	44212	12828
CR8	44221	12463
CR8	44257	12174
CR8	44277	12535
CR8	44280	11961
CR8	44288	12547
CR8	44299	12579
CR8	44308	11970
CR8	44316	12235
CR8	44346	12443
CR8	44363	12158
CR8	44396	11768
CR8	44452	12218
CR8	44460	12592
CR8	44466	12233
CR8	44501	12107
CR8	44511	12391
CR8	44516	11937
CR8	44533	11947
CR8	44552	12151
CR8	44565	12494
CR8	44574	11738
CR8	44590	12153
CR8	44602	12314
CR8	44604	12367
CR8	44608	11998
CR8	44612	12918
CR8	44623	11846
CR8	44633	12093
CR8	44647	12313
CR8	44651	11605
CR8	44655	12436
CR8	44673	12043
CR8	44680	12296
CR8	44681	12195
CR8	44688	11709

CR8	44703	12399
CR8	44751	12329
CR8	44767	12022
CR8	44770	12078
CR8	44778	12017
CR8	44792	12560
CR8	44813	12123
CR8	44816	12384
CR8	44820	11825
CR8	44823	12384
CR8	44826	11965
CR8	44829	11715
CR8	44837	12122
CR8	44852	12245
CR8	44860	12470
CR8	44896	12672
CR8	44910	12208
CR8	44911	12263
CR8	44912	11889
CR8	44957	12348
CR8	44958	12043
CR8	44969	12301
CR8	45016	11916
CR8	45053	11993
CR8	45070	12307
CR8	45084	12529
CR8	45097	11617
CR8	45101	12310
CR8	45105	12037
CR8	45111	11880
CR8	45118	12232
CR8	45130	12484
CR8	45131	12214
CR8	45158	12423
CR8	45161	13102
CR8	45167	12254
CR8	45179	12247
CR8	45188	12691
CR8	45210	12447
CR8	45221	11687
CR8	45225	12400
CR8	45228	12089
CR8	45230	12198
CR8	45238	11927
CR8	45246	11641
CR8	45297	12765
CR8	45300	12195
CR8	45307	12262
CR8	45308	12144
CR8	45311	12596
CR8	45320	12654
CR8	45324	12474
CR8	45326	12178
CR8	45350	11775
CR8	45352	12079
CR8	45357	12614
CR8	45382	12320
CR8	45385	12004
CR8	45389	11967
CR8	45402	11974
CR8	45404	11771
CR8	45427	12257
CR8	45438	12978
CR8	45481	12205
CR8	45484	13316
CR8	45500	11895
CR8	45513	12657

CR8	45523	12747
CR8	45529	12110
CR8	45536	11915
CR8	45542	13482
CR8	45549	12299
CR8	45556	11967
CR8	45563	12296
CR8	45578	12306
CR8	45587	12619
CR8	45593	12493
CR8	45601	12198
CR8	45626	12238
CR8	45628	12133
CR8	45679	12147
CR8	45682	12399
CR8	45694	12188
CR8	45695	12086
CR8	45720	12340
CR8	45744	12243
CR8	45762	11657
CR8	45828	12076
CR8	45837	12048
CR8	45844	12295
CR8	45855	12436
CR8	45870	11744
CR8	45878	12517
CR8	45879	11955
CR8	45888	12884
CR8	45896	12596
CR8	45900	13739
CR8	45951	13727
CR8	45953	13416
CR8	45974	13143
CR8	45988	13369
CR8	46013	13805
CR8	46025	13711
CR8	46067	13472
CR8	46074	13404
CR8	46079	13186
CR8	46081	13588
CR8	46095	12366
CR8	46119	13485
CR8	46157	13334
CR8	46163	13920
CR8	46167	13814
CR8	46176	13662
CR8	46189	14014
CR8	46190	13994
CR8	46215	12889
CR8	46228	13553
CR8	46243	13372
CR8	46259	12416
CR8	46260	13941
CR8	46278	13353
CR8	46329	13387
CR8	46372	12685
CR8	46398	13126
CR8	46405	13493
CR8	46450	13714
CR8	46451	13618
CR8	46464	13817
CR8	46484	12156
CR8	46539	13134
CR8	46548	11994
CR8	46562	14206
CR8	46607	13656
CR8	46608	13833

CR8	46611	13825
CR8	46612	13576
CR8	46621	13711
CR8	46645	13176
CR8	46661	13044
CR8	46665	12175
CR8	46682	13710
CR8	46684	13821
CR8	46706	13157
CR8	46720	13713
CR8	46733	14091
CR8	46738	12751
CR8	46766	13567
CR8	46779	13046
CR8	46794	12816
CR8	46809	13550
CR8	46819	13308
CR8	46828	13033
CR8	46886	13629
CR8	46894	12714
CR8	46905	13607
CR8	46913	13032
CR8	46929	13040
CR8	46947	13760
CR8	46963	13597
CR8	46981	13452
CR8	47000	12216
CR8	47019	13380
CR8	47078	12007
CR8	47101	12571
CR8	47109	13778
CR8	47114	13416
CR8	47129	13741
CR8	47135	13419
CR8	47137	13114
CR8	47152	13632
CR8	47173	12958
CR8	47185	13636
CR8	47187	12921
CR8	47188	13172
CR8	47200	13714
CR8	47246	12887
CR8	47276	13098
CR8	47296	13781
CR8	47306	13338
CR8	47333	13856
CR8	47338	13774
CR8	47341	13891
CR8	47357	13439
CR8	47434	11657
CR8	47445	13698
CR8	47450	11544
CR8	47462	13599
CR8	47476	13087
CR8	47479	13483
CR8	47519	13617
CR8	47544	13728
CR8	47552	14030
CR8	47616	13182
CR8	47626	13252
CR8	47638	13318
CR8	47663	13561
CR8	47676	13422
CR8	47715	13848
CR8	47724	13960
CR8	47738	11825
CR8	47766	13143

CR8	47792	13185
CR8	47806	13635
CR8	47841	13178
CR8	47862	13671
CR8	47878	13718
CR8	47947	13725
CR8	47960	12506
CR8	47963	13439
CR8	48039	13063
CR8	48075	13235
CR8	48083	13627
CR8	48089	13370
CR8	48099	13229
CR8	48122	13846
CR8	48138	13155
CR8	48156	13239
CR8	48159	13465
CR8	48186	13309
CR8	48200	13448
CR8	49516	12712
CR8	49665	11806
CR8	49990	12256
CR8	50031	12688
CR8	50100	11643
CR8	50102	12250
CR8	50179	13058
CR8	50382	12298
CR8	50392	12794
CR8	50464	12198
CR8	50569	11920
CR8	50648	12225
CR8	507	11848
CR8	50707	12117
CR8	50725	11536
CR8	508	11912
CR8	50857	12428
CR8	51048	11946
CR8	51836	12934
CR8	51890	13778
CR8	51982	13397
CR8	52142	12072
CR8	52201	13386
CR8	52294	11877
CR8	52341	12251
CR8	52380	11769
CR8	52401	12393
CR8	52703	11523
CR8	5272	11874
CR8	52843	12482
CR8	53368	11895
CR8	53678	12826
CR8	53750	12521
CR8	53955	12873
CR8	53967	12560
CR8	54955	11850
CR8	55241	11704
CR8	55347	12349
CR8	5538	13290
CR8	55906	11537
CR8	5687	11624
CR8	57308	12503
CR8	57341	12354
CR8	57357	13211
CR8	57583	12249
CR8	57982	13674
CR8	58446	11730
CR8	58534	11786

CR8	58567	12373
CR8	58625	11736
CR8	58706	11611
CR8	58886	12161
CR8	58889	12244
CR8	58959	11758
CR8	58971	12540
CR8	58976	12983
CR8	58982	11585
CR8	59053	12959
CR8	59062	12758
CR8	59087	11892
CR8	59127	12548
CR8	59158	12009
CR8	59178	12344
CR8	59196	12827
CR8	59198	12520
CR8	59247	11589
CR8	59259	11894
CR8	59279	12770
CR8	59318	12493
CR8	59322	12164
CR8	59373	12802
CR8	59404	11804
CR8	59410	11848
CR8	59417	11655
CR8	59420	12328
CR8	59454	13639
CR8	59549	11726
CR8	59551	12493
CR8	59560	12724
CR8	59566	13006
CR8	59571	11977
CR8	59578	11535
CR8	59725	12651
CR8	59740	11666
CR8	59755	11993
CR8	59760	12389
CR8	59786	12181
CR8	59837	12843
CR8	59875	12695
CR8	59903	12318
CR8	59904	12251
CR8	59915	13297
CR8	59935	12171
CR8	60031	12180
CR8	60050	12092
CR8	60071	12510
CR8	60072	11656
CR8	60100	11731
CR8	60105	11583
CR8	60119	12119
CR8	60205	12198
CR8	60231	11830
CR8	60246	12352
CR8	60281	12911
CR8	60291	12132
CR8	60315	12144
CR8	60328	12051
CR8	60346	11607
CR8	60351	11574
CR8	60374	12635
CR8	60378	12348
CR8	60395	12515
CR8	60430	11969
CR8	60447	11765
CR8	60467	13189

CR8	60472	12846
CR8	60475	12393
CR8	60523	12990
CR8	60560	11608
CR8	60608	12403
CR8	60638	12556
CR8	60672	12029
CR8	60732	12350
CR8	60758	12008
CR8	60827	12350
CR8	60837	11552
CR8	6094	11888
CR8	60966	12317
CR8	61046	12048
CR8	61062	11843
CR8	61105	12121
CR8	61106	12469
CR8	61133	11702
CR8	61158	12066
CR8	61191	12270
CR8	61277	11944
CR8	61285	12006
CR8	61302	11634
CR8	61355	12706
CR8	61473	12473
CR8	61677	13251
CR8	61909	13123
CR8	61965	13674
CR8	61984	11708
CR8	62086	11861
CR8	62476	13429
CR8	62521	11849
CR8	62585	13139
CR8	62719	12276
CR8	62734	13415
CR8	62825	11989
CR8	62853	12806
CR8	62890	12316
CR8	62927	11664
CR8	63093	13161
CR8	63286	12329
CR8	63336	12522
CR8	63573	13048
CR8	63864	12260
CR8	64220	12368
CR8	64568	12279
CR8	64986	12015
CR8	65050	11923
CR8	65435	12410
CR8	65635	12926
CR8	65807	12724
CR8	65875	12795
CR8	66605	11756
CR8	66715	12159
CR8	67028	11625
CR8	67664	11728
CR8	68290	11782
CR8	69931	12608
CR8	69992	12487
CR8	70051	12750
CR8	70082	13145
CR8	70118	11952
CR8	70179	12582
CR8	70627	12484
CR8	70639	12033
CR8	70669	12386
CR8	70706	12182

CR8	70729	11956
CR8	70775	11768
CR8	70850	12876
CR8	70911	12774
CR8	70970	12314
CR8	71020	12863
CR8	71056	12457
CR8	71075	12969
CR8	71089	11855
CR8	71091	12535
CR8	71113	11714
CR8	71191	12668
CR8	71202	13255
CR8	71224	11898
CR8	71226	13106
CR8	71261	12054
CR8	71274	12198
CR8	71411	12388
CR8	71601	11769
CR8	71820	12587
CR8	71863	12258
CR8	71907	12044
CR8	71916	12106
CR8	71919	11819
CR8	72061	12276
CR8	72147	12368
CR8	72200	12776
CR8	72453	12038
CR8	72531	12811
CR8	72563	12545
CR8	72582	12221
CR8	72610	12113
CR8	72629	12301
CR8	72637	11639
CR8	72646	12113
CR8	72729	12371
CR8	72761	13031
CR8	72795	11772
CR8	72839	13233
CR8	72884	12692
CR8	72916	12320
CR8	72927	12177
CR8	72987	11823
CR8	73514	13322
CR8	73689	12866
CR8	73726	11764
CR8	73871	12733
CR8	74031	12097
CR8	74065	12078
CR8	75099	11858
CR8	75644	11848
CR8	75661	12101
CR8	75676	11654
CR8	7636	12962
CR8	7833	12497
CR8	7871	12339
CR8	8038	12896
CR8	8070	12653
CR8	8071	13323
CR8	8247	13453
CR8	8300	13178
CR8	8316	12351
CR8	841	11549
CR8	8437	12023
CR8	8529	12536
CR8	8546	12733
CR8	8593	11565

CR8	8663	11573
CR8	8808	11601
CR8	8842	11984
CR8	8983	11840
CR8	9103	11857
CR8	9182	12656
CR8	9232	12212
CR8	9253	11752
CR8	9571	12984
CR8	9917	11769
CR8	9938	12942
CR8_PMA	10362	12653
CR8_PMA	10672	1
CR8_PMA	10804	11997
CR8_PMA	11137	12472
CR8_PMA	11657	11410
CR8_PMA	11745	12263
CR8_PMA	11853	13156
CR8_PMA	12094	13015
CR8_PMA	12326	1
CR8_PMA	13093	11390
CR8_PMA	13769	11294
CR8_PMA	14055	11504
CR8_PMA	14137	11444
CR8_PMA	14194	12314
CR8_PMA	28504	12771
CR8_PMA	29382	1
CR8_PMA	30430	12391
CR8_PMA	30513	11975
CR8_PMA	30787	1
CR8_PMA	30996	12292
CR8_PMA	31218	12025
CR8_PMA	31307	12838
CR8_PMA	31464	1
CR8_PMA	32200	11410
CR8_PMA	32497	1
CR8_PMA	33258	11219
CR8_PMA	33873	12338
CR8_PMA	34161	1
CR8_PMA	34266	11950
CR8_PMA	35042	1
CR8_PMA	35066	11683
CR8_PMA	35235	13269
CR8_PMA	35254	12305
CR8_PMA	36035	11682
CR8_PMA	36693	11202
CR8_PMA	37076	11997
CR8_PMA	37253	11400
CR8_PMA	46420	11812
CR8_PMA	48776	12698
CR8_PMA	49322	12183
CR8_PMA	50779	11394
CR8_PMA	58435	13509
CR8_PMA	59143	12659
CR8_PMA	59524	13190
CR8_PMA	59989	14173
CR8_PMA	60211	11314
CR8_PMA	60315	12605
CR8_PMA	60348	13170
CR8_PMA	60395	12529
CR8_PMA	60398	11936
CR8_PMA	60421	12227
CR8_PMA	60579	11466
CR8_PMA	60732	12416
CR8_PMA	60827	12876
CR8_PMA	61060	12704
CR8_PMA	61158	12898

CR8_PMA	61302	11782
CR8_PMA	61327	11458
CR8_PMA	62483	1
CR8_PMA	7682	12870
CR8_PMA	7690	11898
CR8_PMA	8217	12490
CR8_PMA	9071	12133
CR8_PMA	9082	12341
CR8_PMA	9425	12368
CR8_PMA	9427	12207
CR8_PMA	9474	11626
CR8_PMA	9615	12633
CR8_PMA	9619	11637
UR	10744	14178
UR	10760	12342
UR	10870	13439
UR	10995	11770
UR	11025	12890
UR	11150	12918
UR	11543	12319
UR	11897	12530
UR	12211	11720
UR	12217	13629
UR	12326	1
UR	12333	11483
UR	12392	12649
UR	12912	12325
UR	13091	11831
UR	13092	11598
UR	13106	11712
UR	13274	12284
UR	1331	11775
UR	14199	12650
UR	1440	11851
UR	14477	12871
UR	14545	12233
UR	14599	13755
UR	14633	11705
UR	14678	11741
UR	1481	11778
UR	15035	13022
UR	15249	13637
UR	15292	13212
UR	15342	13951
UR	1566	11458
UR	15908	11656
UR	15941	12165
UR	16096	12437
UR	16161	12369
UR	16250	12327
UR	1638	11615
UR	16425	12209
UR	1650	12284
UR	16520	12313
UR	16522	12324
UR	16590	12709
UR	16847	11844
UR	16916	12393
UR	16971	12652
UR	17051	12867
UR	17322	12943
UR	17348	12006
UR	17519	11737
UR	20652	11900
UR	20782	11789
UR	24509	12475
UR	25139	11930

UR	25425	11993
UR	25471	13159
UR	25637	11781
UR	26227	12068
UR	26288	12945
UR	28064	12041
UR	29061	12804
UR	30017	13203
UR	30787	1
UR	30882	12450
UR	31080	12274
UR	31616	13126
UR	32357	13063
UR	33058	13189
UR	33189	11653
UR	3351	13676
UR	3360	13234
UR	339	11707
UR	34240	12755
UR	34464	13530
UR	3464	13020
UR	34741	12347
UR	3482	13627
UR	35586	12940
UR	35629	13246
UR	3571	13256
UR	36142	12990
UR	3624	13699
UR	36381	13476
UR	3647	11952
UR	3672	13279
UR	36762	13306
UR	36785	12933
UR	36803	13686
UR	36867	12814
UR	36899	12809
UR	36937	13066
UR	36943	13146
UR	37002	13462
UR	37007	12364
UR	37071	12761
UR	37089	13332
UR	37107	12883
UR	37124	12484
UR	37125	13031
UR	37129	13456
UR	37166	11907
UR	37178	12537
UR	37193	12884
UR	37198	13083
UR	37226	12098
UR	37293	12037
UR	37300	13657
UR	37312	12206
UR	37315	13024
UR	37316	12420
UR	37329	12241
UR	3746	13481
UR	3752	12865
UR	37566	13224
UR	37653	12508
UR	3771	12595
UR	37726	13759
UR	37735	12046
UR	37877	13309
UR	37920	11824
UR	37948	12100

UR	3796	12346
UR	38001	12698
UR	38303	12520
UR	38331	12700
UR	38400	12315
UR	38432	12948
UR	38449	11771
UR	38463	12218
UR	38519	12476
UR	3871	13515
UR	38770	13104
UR	38813	13115
UR	39044	12499
UR	39106	12415
UR	39124	11796
UR	3925	12331
UR	39321	11812
UR	39484	11762
UR	39676	11957
UR	39684	12217
UR	39809	12091
UR	39869	11625
UR	39936	11742
UR	40158	14303
UR	40172	12318
UR	40178	13115
UR	40202	12934
UR	40219	13186
UR	40222	12527
UR	40238	12471
UR	40241	12436
UR	40245	13259
UR	40263	11933
UR	40264	14181
UR	40286	12002
UR	40289	12805
UR	40314	12962
UR	40322	11898
UR	40325	12256
UR	40334	12126
UR	40347	13195
UR	40373	12899
UR	40376	12403
UR	40389	12616
UR	4039	13741
UR	40391	12807
UR	40399	12556
UR	40407	11817
UR	40408	12564
UR	40412	11713
UR	40426	12332
UR	40436	13243
UR	40443	11586
UR	40473	13405
UR	40476	12173
UR	40479	13164
UR	40484	12573
UR	40492	11669
UR	40510	12857
UR	4055	13401
UR	40550	14147
UR	40574	12647
UR	40575	13045
UR	40577	12467
UR	40621	12777
UR	40640	12691
UR	40651	11540

UR	40679	12962
UR	40686	13627
UR	40696	12558
UR	40697	12583
UR	40716	13215
UR	40718	13336
UR	40733	12059
UR	40740	12924
UR	40753	12129
UR	40779	12720
UR	4078	12716
UR	40819	12356
UR	40856	12192
UR	40861	12562
UR	40882	12329
UR	40884	12720
UR	40889	12775
UR	40892	11990
UR	40898	13027
UR	40899	12675
UR	40911	12695
UR	4093	13049
UR	40930	11866
UR	40932	13939
UR	40943	13117
UR	40945	11910
UR	40958	11764
UR	40960	12459
UR	40968	12614
UR	40992	12782
UR	41001	12103
UR	41006	13327
UR	41008	12509
UR	41016	12508
UR	41018	12929
UR	4102	13526
UR	41035	11926
UR	41054	13266
UR	41061	13316
UR	41068	13199
UR	41080	12957
UR	41088	11887
UR	41123	12709
UR	41194	12667
UR	41227	12439
UR	41284	12041
UR	41332	11947
UR	41373	12008
UR	41428	11631
UR	41456	12858
UR	41462	12245
UR	41491	12018
UR	41499	11886
UR	41542	11896
UR	41589	12010
UR	41631	12554
UR	41639	11747
UR	41745	12456
UR	41749	11599
UR	41840	12179
UR	41854	11712
UR	41881	12197
UR	41886	12247
UR	41904	12906
UR	41908	12447
UR	41938	11737
UR	41964	11688

UR	42065	11988
UR	421	11633
UR	42104	11806
UR	42129	12397
UR	42285	12062
UR	42295	12660
UR	42310	11820
UR	42479	12610
UR	42511	14498
UR	42514	11721
UR	42609	12178
UR	42616	12325
UR	42672	11761
UR	42684	11540
UR	42706	11683
UR	4273	12884
UR	42748	11914
UR	42889	11841
UR	42933	12585
UR	42959	12747
UR	43361	11661
UR	43461	11885
UR	43496	12012
UR	43507	11565
UR	43571	12476
UR	43709	11820
UR	43740	11721
UR	43755	11959
UR	43982	12136
UR	44057	11681
UR	44069	11658
UR	44153	11986
UR	44212	12457
UR	44224	12112
UR	44243	12115
UR	44277	12110
UR	44299	12226
UR	44346	11660
UR	44371	12325
UR	44380	12471
UR	44422	11631
UR	44432	11455
UR	44482	11611
UR	44534	11682
UR	44562	11806
UR	44565	11923
UR	44623	11560
UR	44647	11693
UR	44673	11794
UR	44677	11600
UR	44680	11694
UR	44681	11747
UR	44688	11652
UR	44702	11542
UR	44743	11805
UR	44744	11569
UR	44745	11919
UR	44751	11748
UR	44770	11545
UR	44789	11996
UR	44792	11875
UR	44816	12025
UR	44820	11669
UR	44823	11937
UR	44860	11965
UR	44896	12163
UR	44910	11504

UR	44976	12207
UR	45005	11501
UR	45008	11922
UR	45016	11571
UR	45052	12272
UR	45118	11502
UR	45131	11663
UR	45151	11828
UR	45158	11499
UR	45161	12375
UR	45210	12053
UR	45230	11800
UR	45269	11985
UR	45286	11957
UR	45297	12096
UR	45299	11838
UR	45300	11498
UR	45304	12107
UR	45320	12106
UR	45324	12193
UR	45343	11932
UR	45402	11586
UR	45405	11595
UR	45438	12473
UR	45440	11634
UR	45481	11817
UR	45487	12578
UR	45507	11458
UR	45508	12047
UR	45513	12557
UR	45523	12350
UR	45529	11594
UR	45542	13447
UR	45563	11647
UR	45587	12054
UR	45592	11891
UR	45595	13056
UR	45636	11657
UR	45679	11605
UR	45682	11912
UR	45695	11509
UR	45720	11888
UR	45725	11469
UR	45744	11845
UR	45786	12370
UR	45844	11814
UR	45861	11800
UR	45870	11486
UR	45879	11963
UR	45887	12059
UR	45914	13396
UR	45951	13320
UR	45955	13083
UR	46074	12935
UR	46079	13576
UR	46138	13575
UR	46162	13463
UR	46167	13495
UR	46217	13637
UR	46226	13401
UR	46259	11980
UR	46360	12802
UR	46362	12717
UR	46367	13335
UR	46417	13546
UR	46450	13368
UR	46464	13480

UR	46520	12617
UR	46532	13451
UR	46571	13576
UR	46608	13531
UR	46621	13398
UR	46661	12485
UR	46684	13496
UR	46712	13623
UR	46738	11959
UR	46752	13487
UR	46766	13235
UR	46809	13532
UR	46826	13571
UR	46886	13329
UR	46891	13555
UR	46905	13370
UR	46913	12760
UR	46981	13324
UR	46998	13277
UR	47019	13452
UR	47109	13411
UR	47129	13355
UR	47135	12717
UR	47187	12371
UR	47200	13311
UR	47296	13399
UR	47327	13036
UR	47338	13461
UR	47357	12865
UR	47445	13311
UR	47544	13415
UR	47552	13760
UR	47616	12624
UR	47663	13164
UR	47680	13487
UR	47715	13535
UR	47728	13460
UR	47806	13380
UR	47819	13551
UR	47832	13507
UR	47841	12421
UR	47869	13697
UR	47947	13342
UR	48021	11566
UR	48039	12751
UR	48048	13429
UR	48075	12501
UR	48081	13438
UR	48083	13193
UR	48200	12879
UR	48830	13235
UR	52305	11998
UR	53955	13016
UR	5538	12798
UR	57357	12712
UR	58959	11561
UR	58971	12954
UR	58987	12954
UR	59062	13275
UR	59178	13263
UR	59463	12801
UR	59740	12042
UR	59760	12261
UR	59875	12982
UR	59935	12228
UR	60010	12948
UR	60072	12352

UR	60119	12441
UR	60279	12071
UR	60281	12800
UR	60315	11856
UR	60405	12019
UR	60467	12874
UR	60538	12421
UR	60557	12421
UR	60565	11528
UR	60697	11763
UR	60741	11656
UR	60822	12438
UR	60827	12012
UR	61013	13346
UR	61094	13068
UR	61101	12289
UR	61105	12021
UR	61158	11513
UR	61317	12861
UR	61785	12201
UR	61909	12505
UR	61935	11484
UR	62664	13138
UR	63161	11766
UR	63182	12518
UR	63711	12149
UR	66457	12322
UR	66513	12685
UR	66896	12297
UR	69931	12974
UR	69992	11648
UR	70051	11778
UR	70850	12474
UR	71202	13283
UR	71261	12144
UR	71274	11920
UR	71936	11780
UR	71954	12580
UR	71968	11613
UR	72019	12021
UR	72042	12984
UR	72147	12700
UR	72160	13004
UR	72597	13251
UR	72855	12698
UR	72859	12055
UR	73689	12415
UR	74031	11585
UR	7871	12775
UR	8249	12712
UR	8368	12049
UR	8808	11905
UR	8983	13070
UR	9232	11832
UR	9872	12987
UR	9921	12107
UR	9931	12580
UR_PMA	15495	12796
UR_PMA	61158	12838
